# Supplementary figures and images for: Global Geographic and Temporal Analysis of SARS-CoV-2 Haplotypes Normalized by COVID-19 Cases During the Pandemic
Source: Front Microbiol. 2021 Feb 17;12:612432. doi: 10.3389/fmicb.2021.612432 (PMC7971176; doi:10.3389/fmicb.2021.612432)

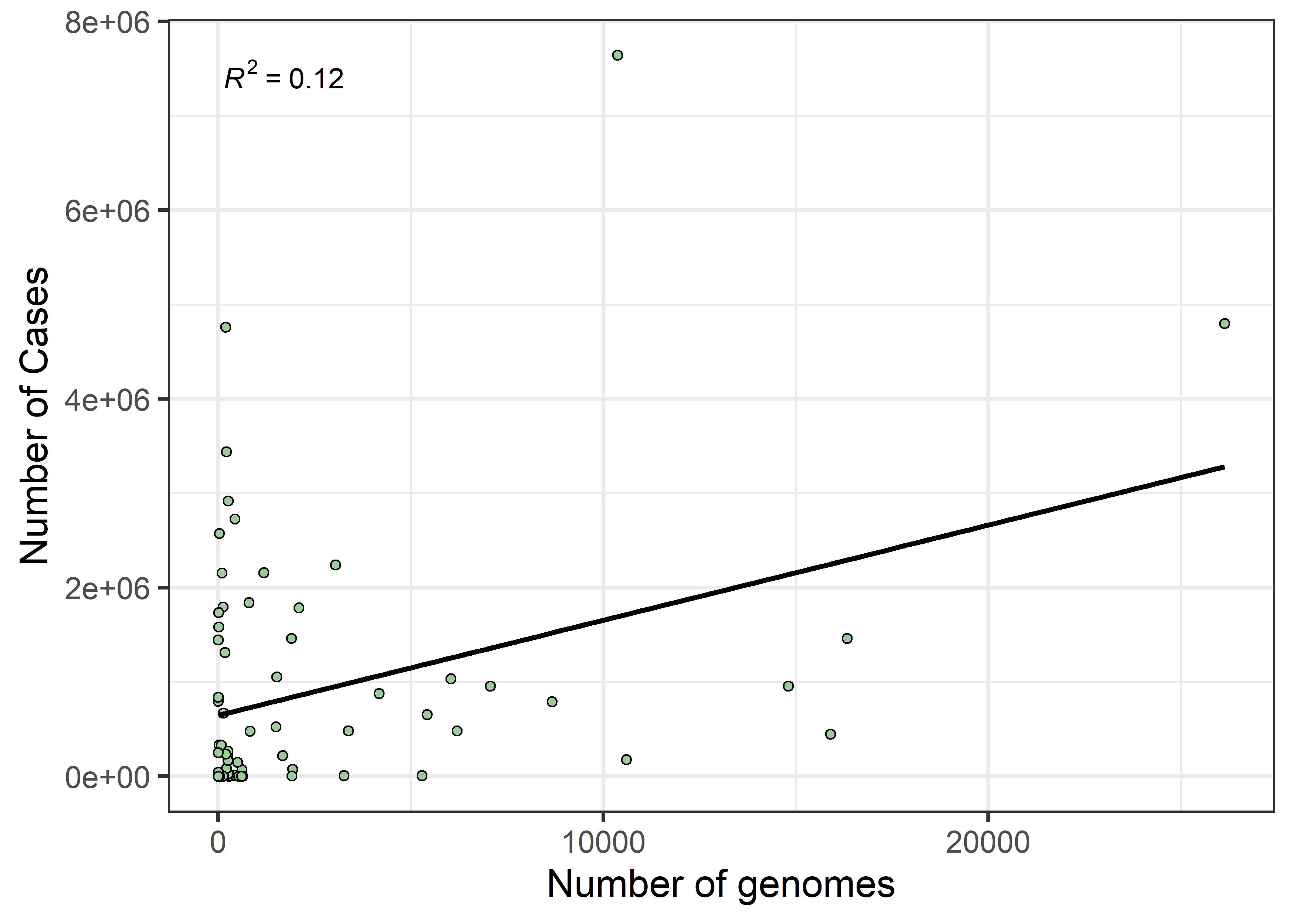

Supplement: Supplementary file 1 [file Data_Sheet_1.zip › Figure_S1.jpg]

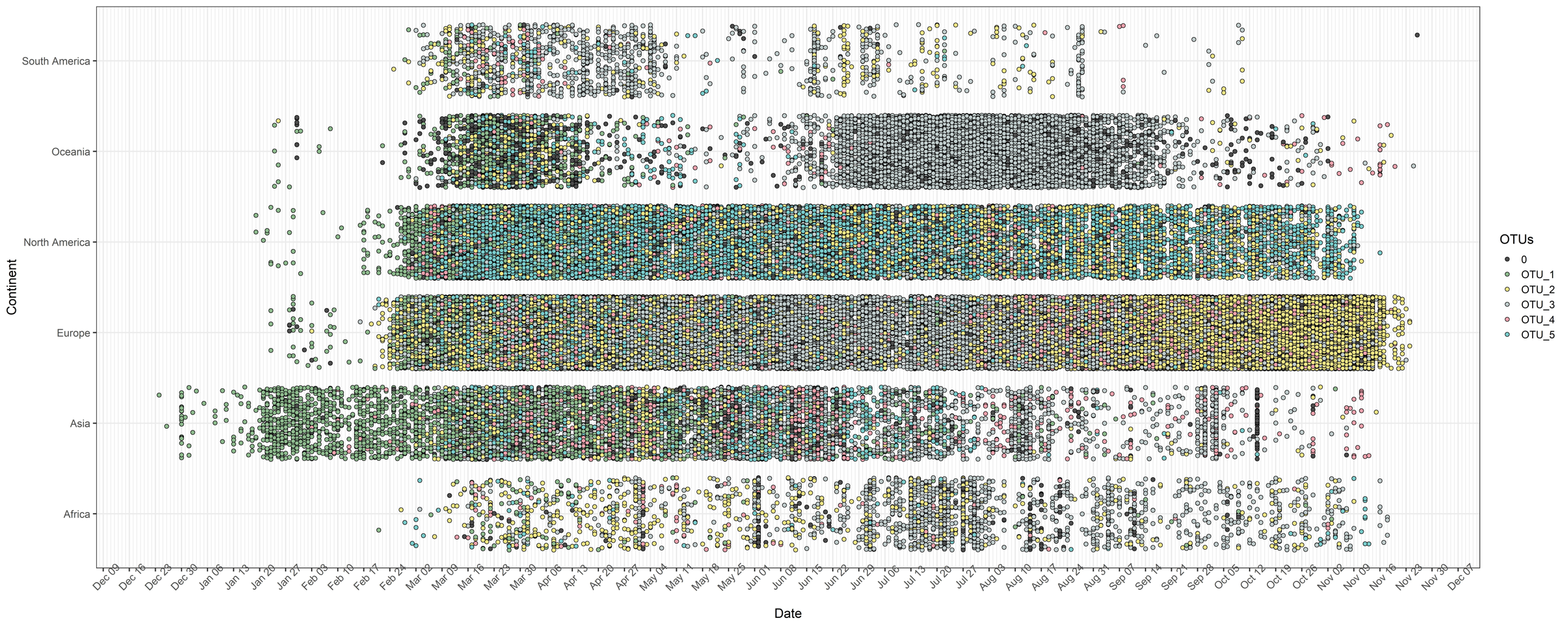

Supplement: Supplementary file 1 [file Data_Sheet_1.zip › Figure_S3.jpg]

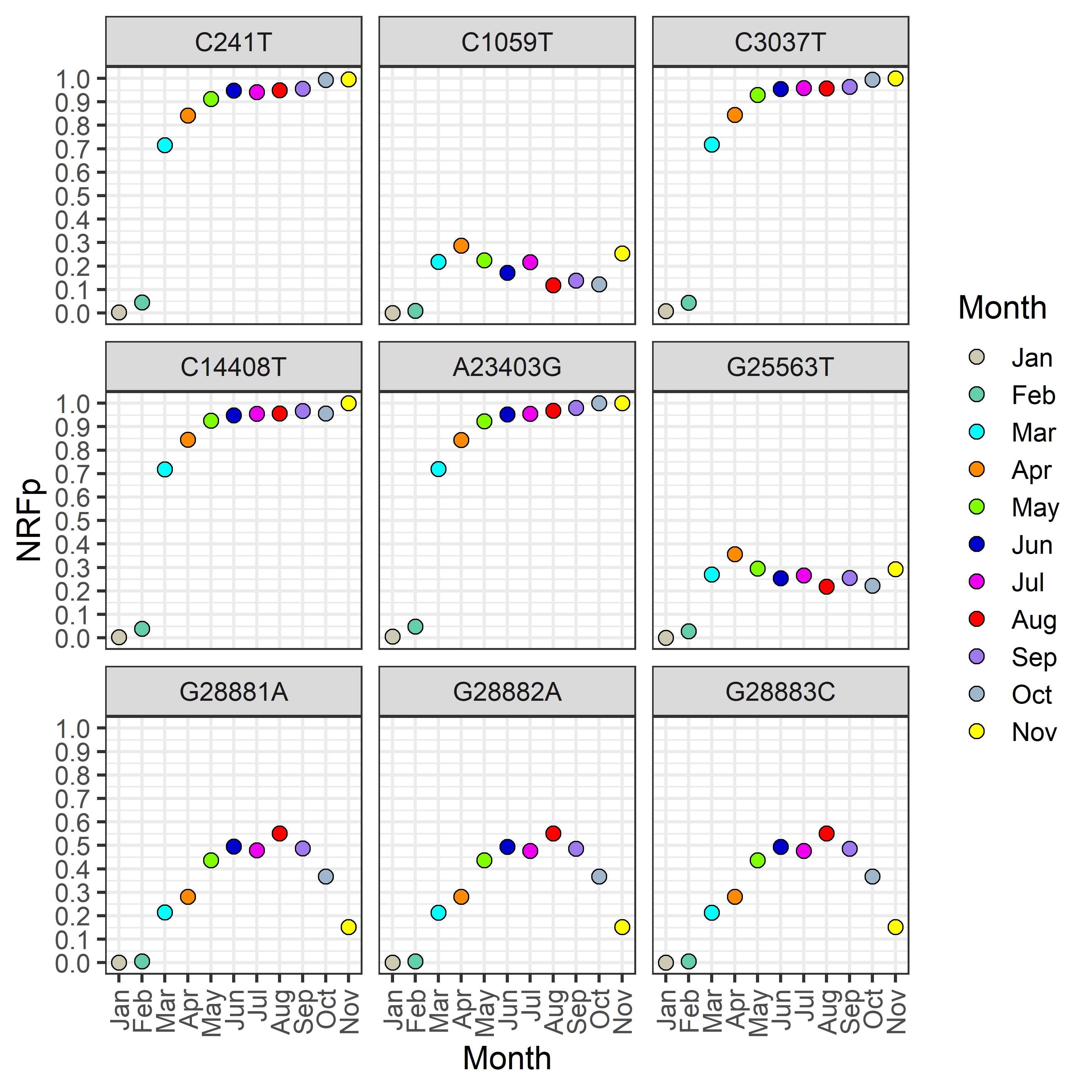

Supplement: Supplementary file 1 [file Data_Sheet_1.zip › Figure_S4.jpg]

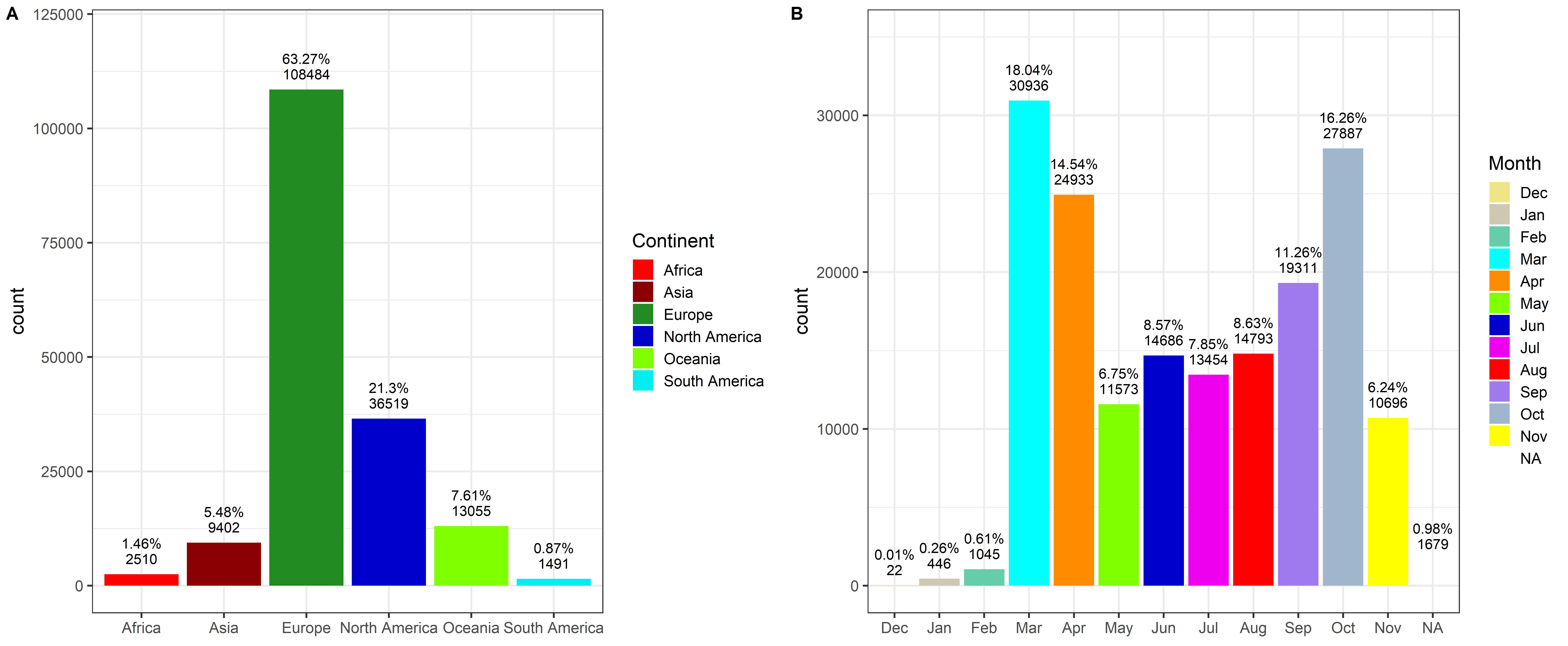

Supplement: Supplementary file 1 [file Data_Sheet_1.zip › Figure_S5.jpg]

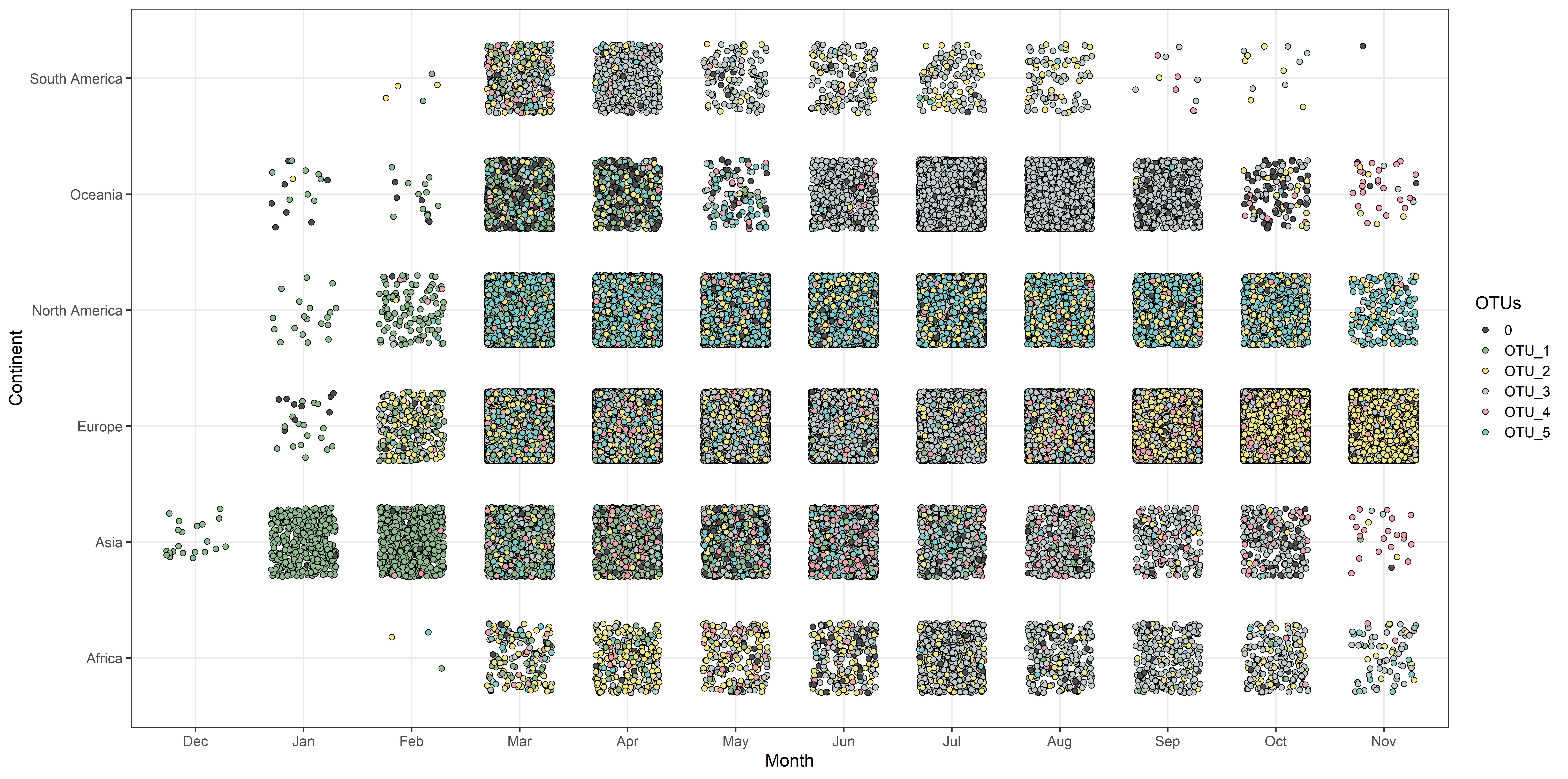

Supplement: Supplementary file 1 [file Data_Sheet_1.zip › Figure_S6.jpg]

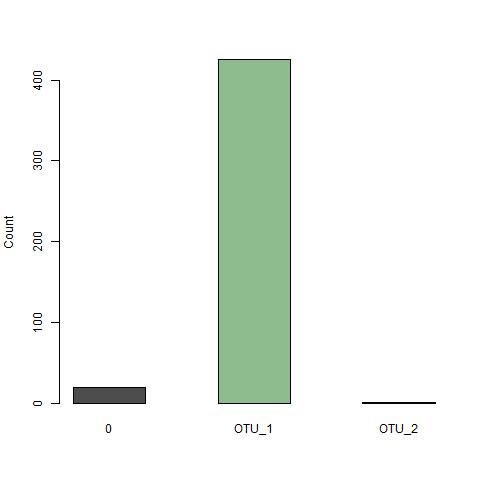

Supplement: Supplementary file 1 [file Data_Sheet_1.zip › Figure_S7.jpg]

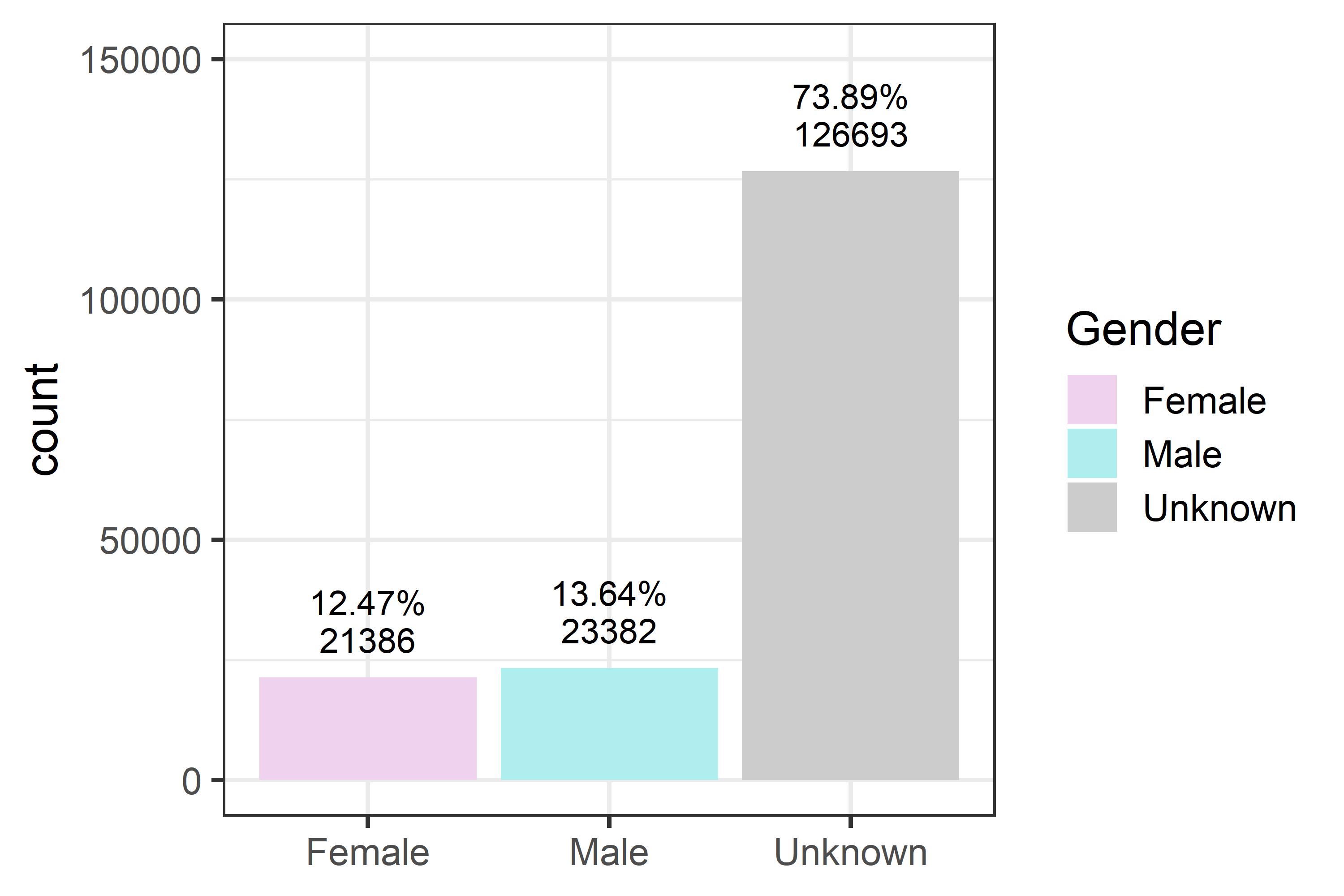

Supplement: Supplementary file 1 [file Data_Sheet_1.zip › Figure_S8.jpg]

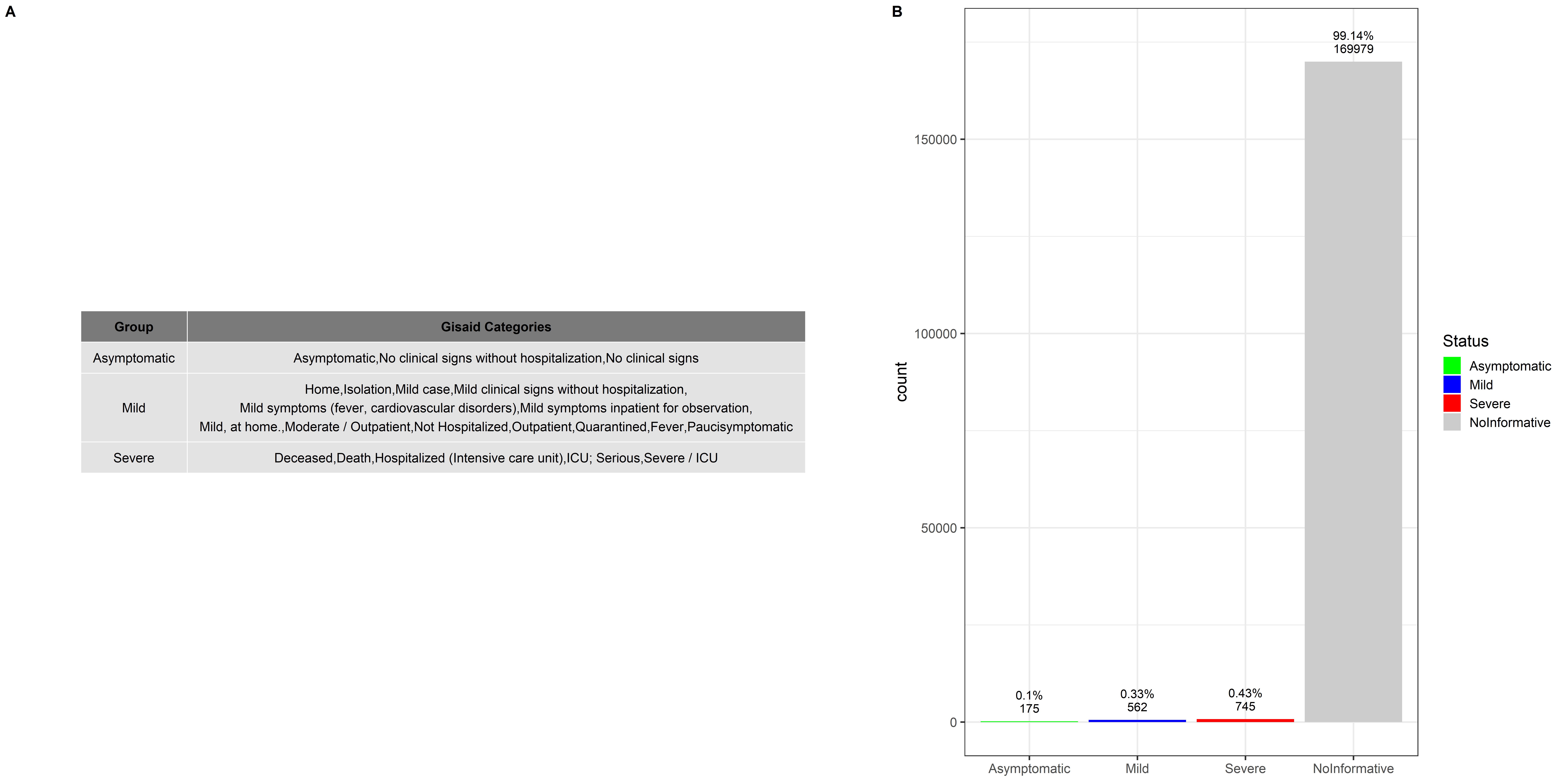

Supplement: Supplementary file 1 [file Data_Sheet_1.zip › Figure_S9.jpg]

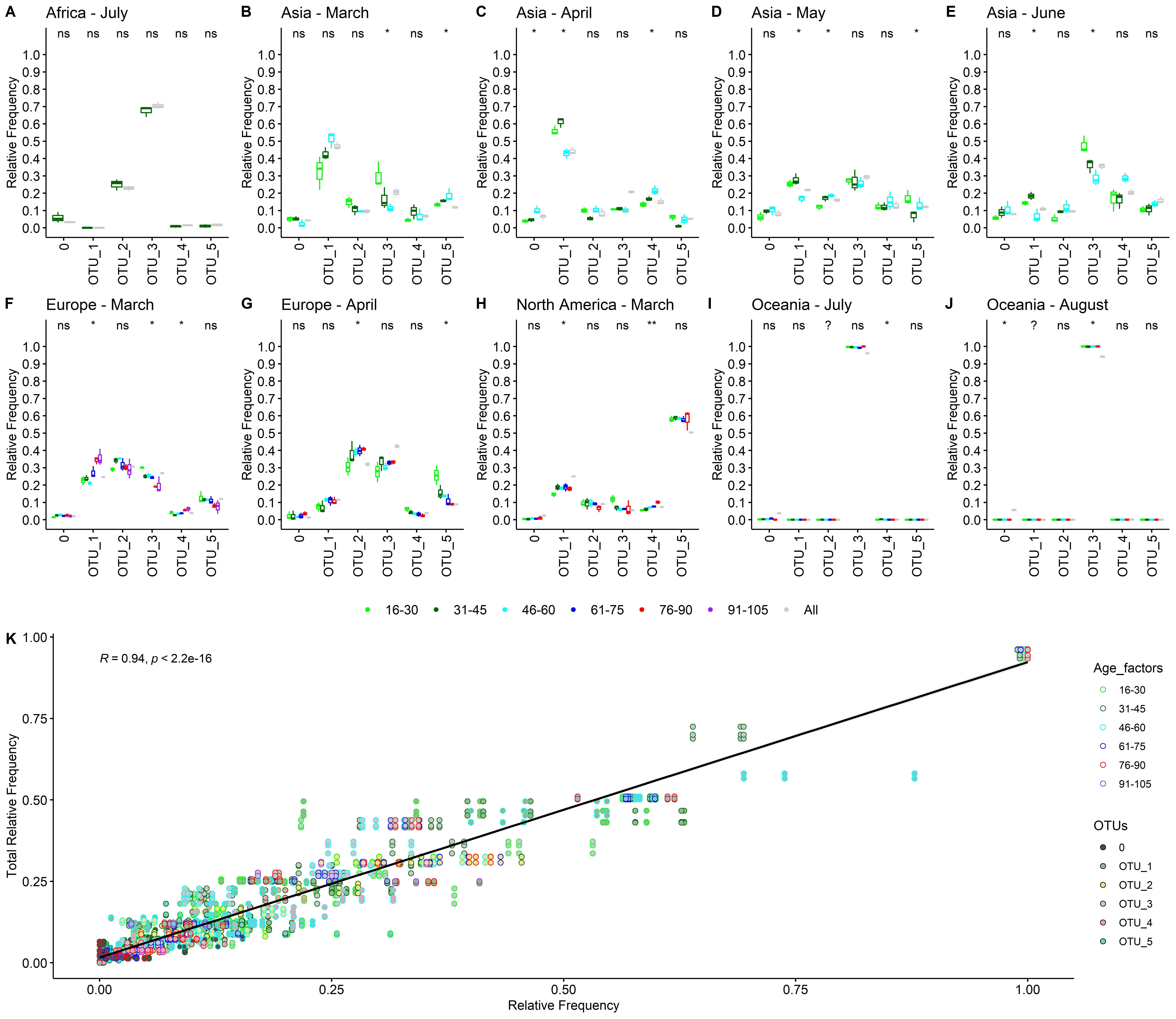

Supplement: Supplementary file 1 [file Data_Sheet_1.zip › Figure_S10.jpg]

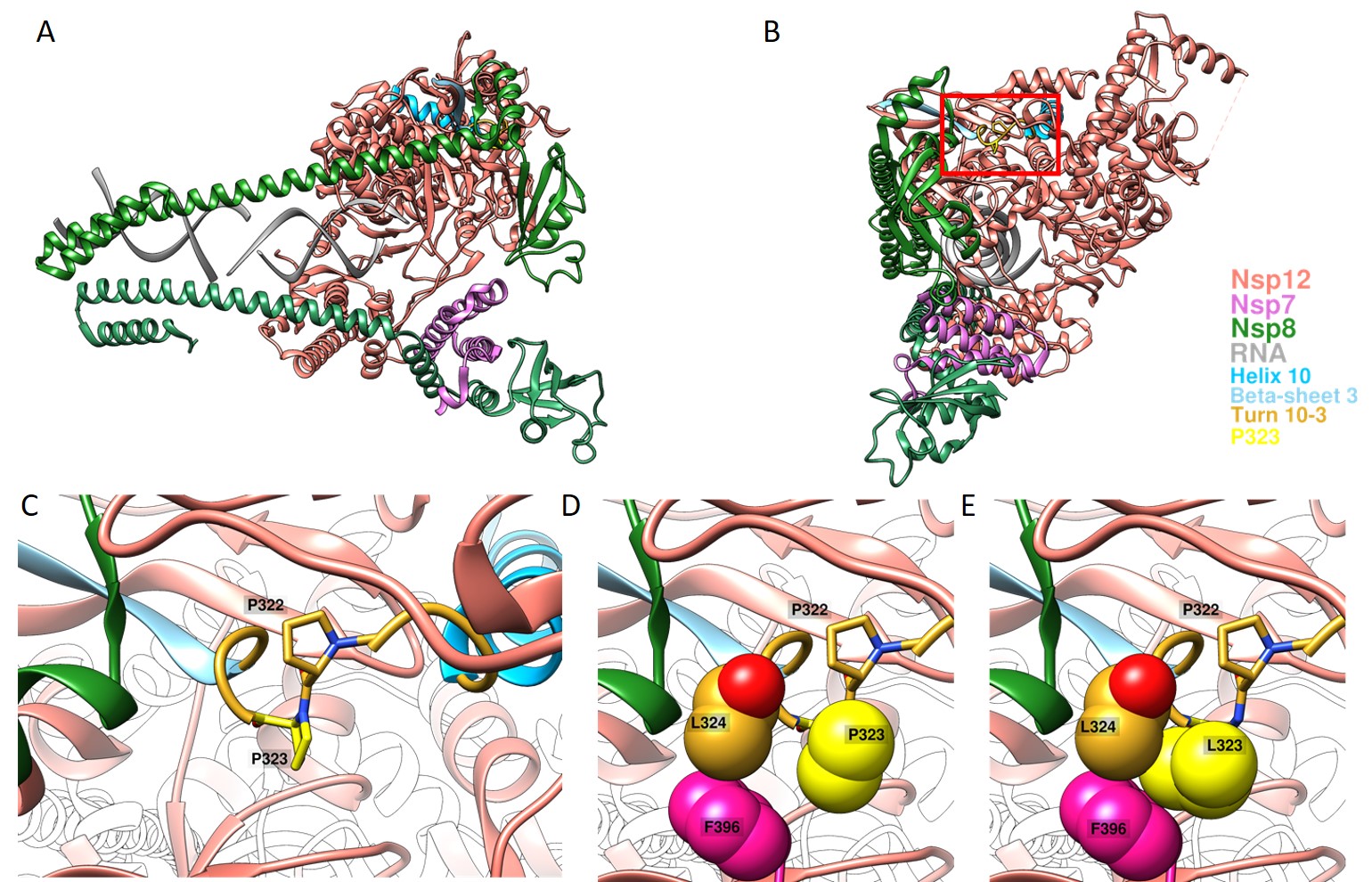

Supplement: Supplementary file 1 [file Data_Sheet_1.zip › Figure_S12.jpg]

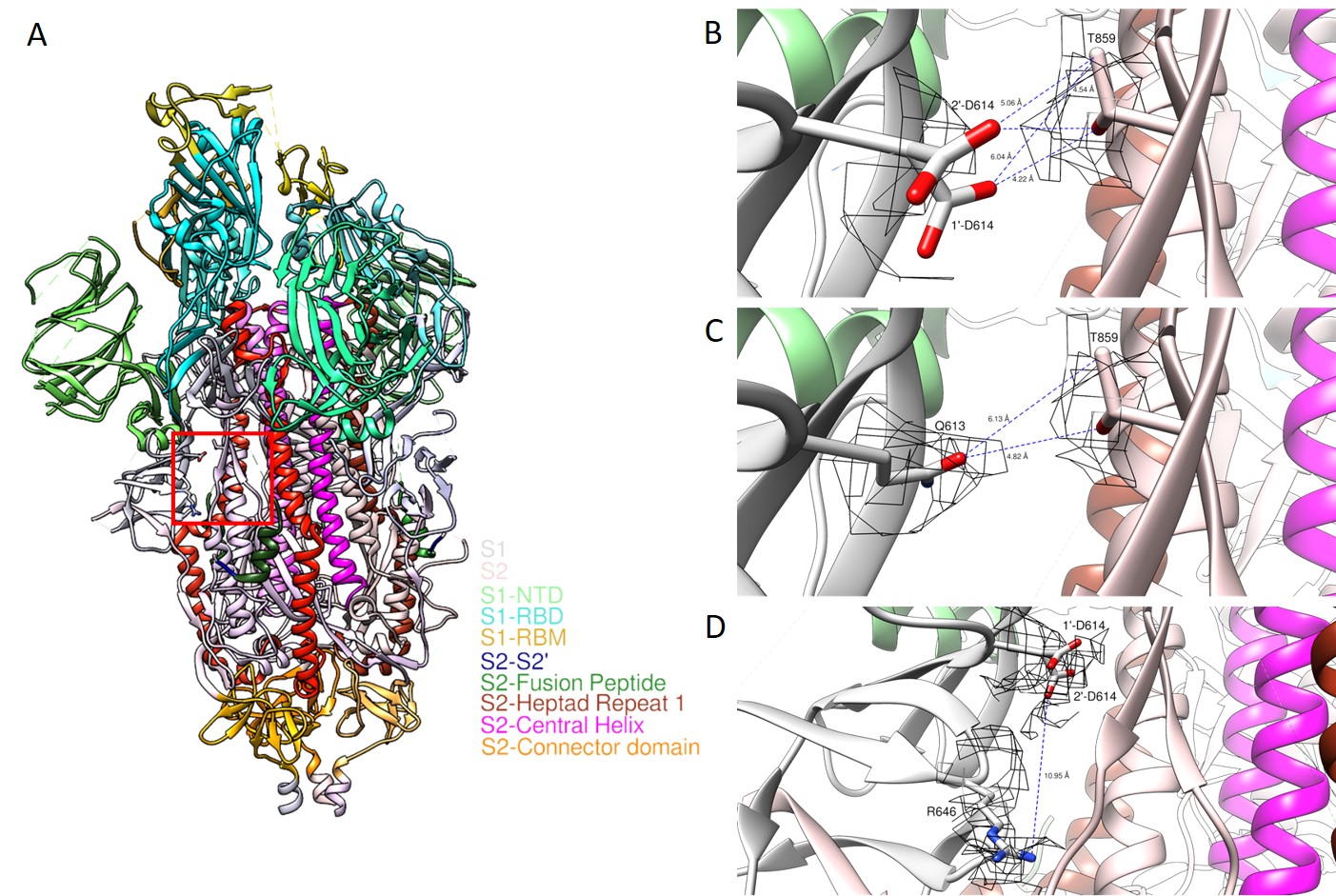

Supplement: Supplementary file 1 [file Data_Sheet_1.zip › Figure_S13.jpg]

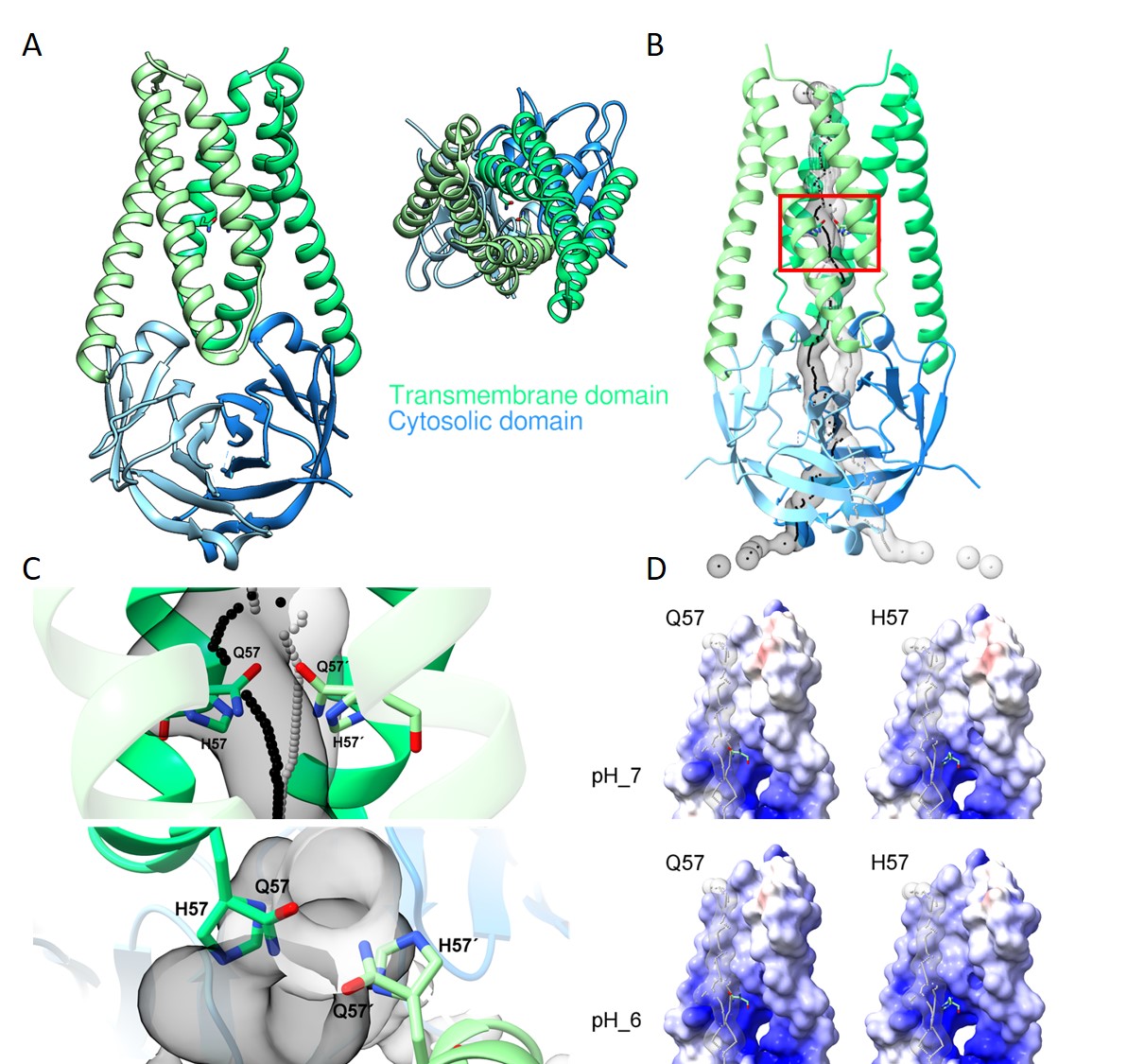

Supplement: Supplementary file 1 [file Data_Sheet_1.zip › Figure_S14.jpg]

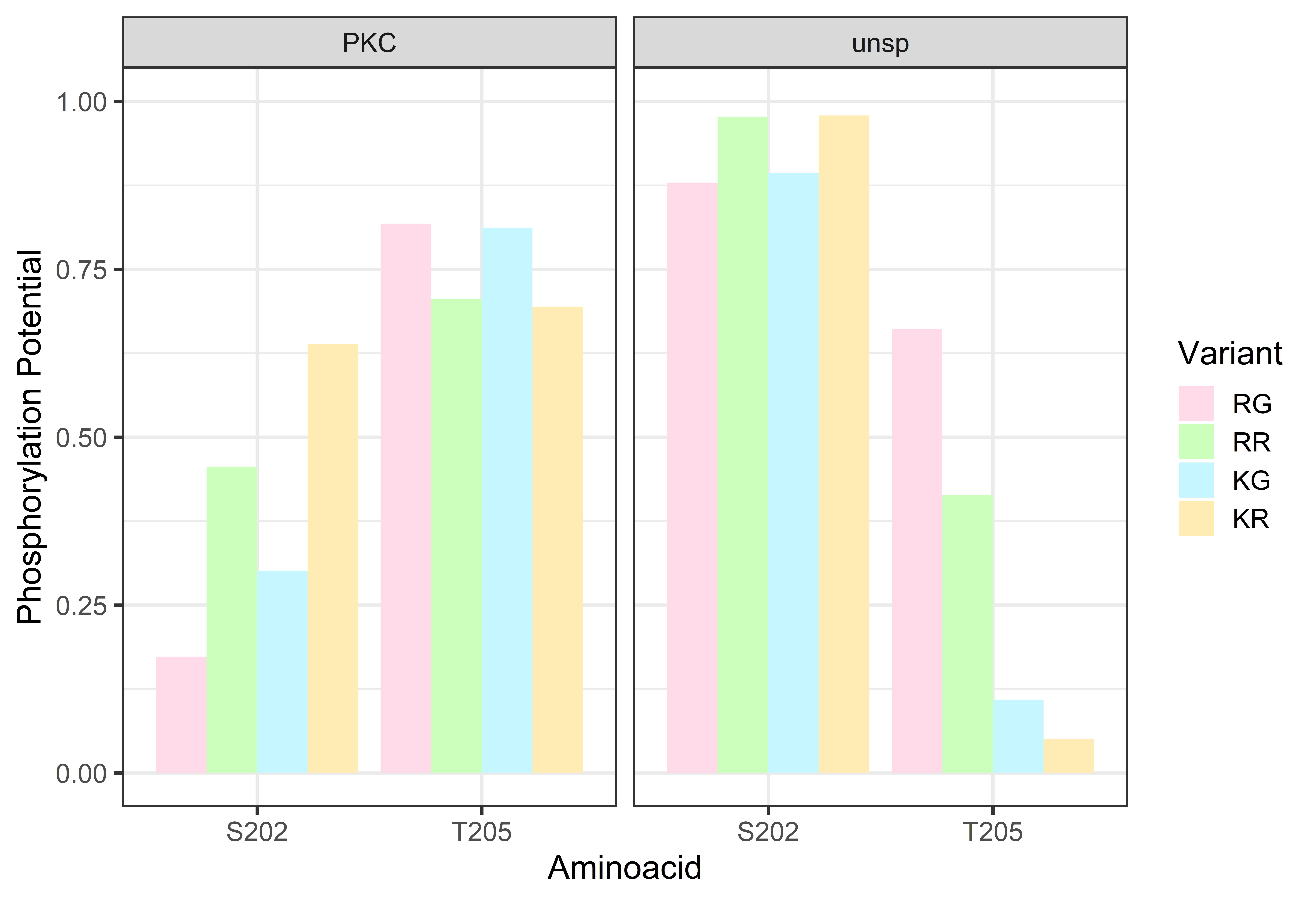

Supplement: Supplementary file 1 [file Data_Sheet_1.zip › Figure_S15.jpg]

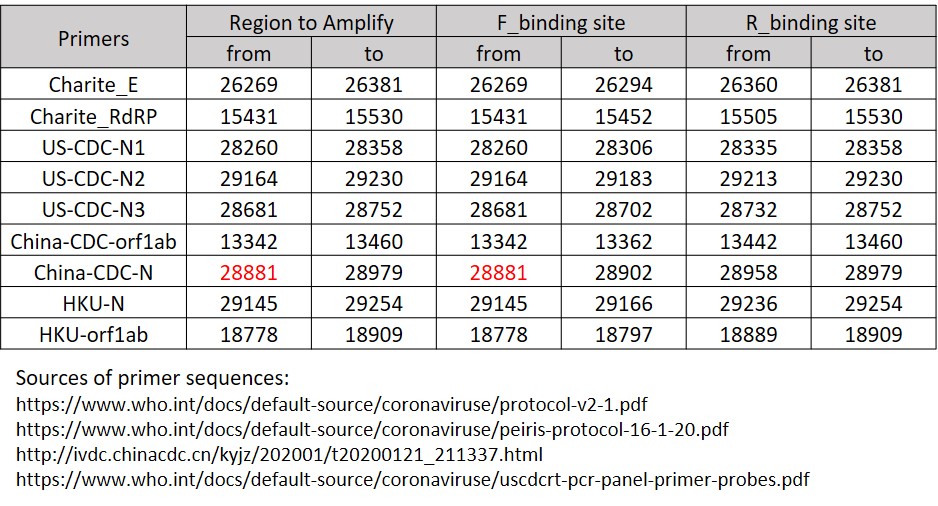

Supplement: Supplementary file 1 [file Data_Sheet_1.zip › Table_S1.jpg]

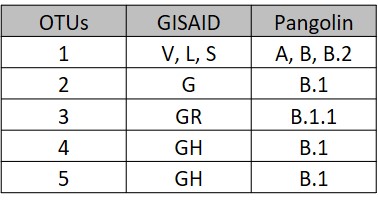

Supplement: Supplementary file 1 [file Data_Sheet_1.zip › Table_S2.jpg]
