# Supplementary material for: Global Geographic and Temporal Analysis of SARS-CoV-2 Haplotypes Normalized by COVID-19 Cases During the Pandemic
Source: Front Microbiol. 2021 Feb 17;12:612432. doi: 10.3389/fmicb.2021.612432 (PMC7971176; doi:10.3389/fmicb.2021.612432)
Supplement: Supplementary file 2 [file Data_Sheet_2.zip › 16_11-05_to_11-08.pdf]

We gratefully acknowledge the following Authors from the Originating laboratories responsible for obtaining the specimens, as well as the Submitting laboratories where the genome data were generated and shared via GISAID, on which this research is based.

All Submitters of data may be contacted directly via www.gisaid.org

Accession ID	Originating Laboratory	Submitting Laboratory	Authors
EPI_ISL_614294	Maimonides Medical Center	New York City Public Health Laboratory	Jade Wang, et al.
EPI_ISL_614298, EPI_ISL_614304, EPI_ISL_614307, EPI_ISL_614308, EPI_ISL_614310, EPI_ISL_614311, EPI_ISL_614312	Faroese National Reference Laboratory for Fish and Animal Diseases	Faroese National Reference Laboratory for Fish and Animal Diseases	Maria Marjunardóttir Dahl, Petra Elisabeth Petersen, Debes Hammershaimb Christiansen
EPI_ISL_614347, EPI_ISL_614348, EPI_ISL_614349, EPI_ISL_614351, EPI_ISL_614352, EPI_ISL_614353, EPI_ISL_614354, EPI_ISL_614355, EPI_ISL_614356, EPI_ISL_614357, EPI_ISL_614358, EPI_ISL_614359, EPI_ISL_614360, EPI_ISL_614361, EPI_ISL_614362, EPI_ISL_614363, EPI_ISL_614364, EPI_ISL_614365, EPI_ISL_614366, EPI_ISL_614367, EPI_ISL_614368, EPI_ISL_614371, EPI_ISL_614372, EPI_ISL_614375, EPI_ISL_614376, EPI_ISL_614377, EPI_ISL_614379, EPI_ISL_614380, EPI_ISL_614381, EPI_ISL_614384, EPI_ISL_614386, EPI_ISL_614387, EPI_ISL_614388, EPI_ISL_614391, EPI_ISL_614393			
see above	Molecular diagnostic unit for viral haemorrhagic fevers and emerging viruses, Bouaké CHU Laboratory	Project group Epidemology of Highly Pathogenic Microorganisms, Robert Koch-Institute	Chantal Akoua-Koffi, Diané Bamourou, Etilé Anoh, Essia Belarbi, Safiatou Karidioula, Grit Schubert, Adjaratou Traoré, Soundélé Maïté, Monemo Pacome, Coulibaly Mbegnan, Bamba Fatoumata Touré, Kra Ouffoué, Fabian Leendertz
EPI_ISL_614396, EPI_ISL_614397, EPI_ISL_614398	Laboratorio Biologia Molecolare Sars Cov2 - UOC Laboratorio Analisi - Servizio Medicina di Laboratorio, Ospedale "San Francesco" - ATS-ASSL Nuoro	Laboratorio specialistico UOC Ematologia - Ospedale "San Francesco" - ATS-ASSL Nuoro	Piras Giovanna, Fancello Tatiana, Asproni Rosanna, Fiamma Maura, Monne Maria Itria, Toja Alessandro, Sanna Filomena, Floris Anna Rita, Sulis Vincenzo, Palmas Angelo Domenico, Casu Gavino, Lo Maglio Iana, Mameli Giuseppe
EPI_ISL_614419, EPI_ISL_614427, EPI_ISL_614428, EPI_ISL_614429, EPI_ISL_614430, EPI_ISL_614431, EPI_ISL_614432, EPI_ISL_614433, EPI_ISL_614434, EPI_ISL_614435, EPI_ISL_614436, EPI_ISL_614437, EPI_ISL_614438, EPI_ISL_614439, EPI_ISL_614440, EPI_ISL_614441, EPI_ISL_614442, EPI_ISL_614443, EPI_ISL_614444, EPI_ISL_614448, EPI_ISL_614449, EPI_ISL_614450, EPI_ISL_614451, EPI_ISL_614452, EPI_ISL_614453, EPI_ISL_614454, EPI_ISL_614455, EPI_ISL_614456, EPI_ISL_614457, EPI_ISL_614458, EPI_ISL_614459, EPI_ISL_614460, EPI_ISL_614461, EPI_ISL_614462, EPI_ISL_614463, EPI_ISL_614464, EPI_ISL_614465, EPI_ISL_614466, EPI_ISL_614467, EPI_ISL_614468, EPI_ISL_614469, EPI_ISL_614470, EPI_ISL_614471, EPI_ISL_614472, EPI_ISL_614473, EPI_ISL_614474, EPI_ISL_614475, EPI_ISL_614476, EPI_ISL_614477, EPI_ISL_614478, EPI_ISL_614479, EPI_ISL_614480, EPI_ISL_614481, EPI_ISL_614482, EPI_ISL_614483, EPI_ISL_614484, EPI_ISL_614485, EPI_ISL_614486, EPI_ISL_614487, EPI_ISL_614488, EPI_ISL_614489, EPI_ISL_614490, EPI_ISL_614491, EPI_ISL_614492, EPI_ISL_614493, EPI_ISL_614494, EPI_ISL_614495, EPI_ISL_614496, EPI_ISL_614497, EPI_ISL_614498, EPI_ISL_614499, EPI_ISL_614500, EPI_ISL_614501, EPI_ISL_614502, EPI_ISL_614503, EPI_ISL_614504, EPI_ISL_614505, EPI_ISL_614506, EPI_ISL_614507, EPI_ISL_614508, EPI_ISL_614509, EPI_ISL_614510, EPI_ISL_614511, EPI_ISL_614512, EPI_ISL_614513, EPI_ISL_614514, EPI_ISL_614515, EPI_ISL_614516, EPI_ISL_614517, EPI_ISL_614518, EPI_ISL_614519, EPI_ISL_614520, EPI_ISL_614521, EPI_ISL_614522, EPI_ISL_614523, EPI_ISL_614524, EPI_ISL_614525, EPI_ISL_614526, EPI_ISL_614527, EPI_ISL_614528, EPI_ISL_614529, EPI_ISL_614530, EPI_ISL_614531, EPI_ISL_614532, EPI_ISL_614533, EPI_ISL_614534, EPI_ISL_614535, EPI_ISL_614536, EPI_ISL_614537, EPI_ISL_614538, EPI_ISL_614539, EPI_ISL_614540, EPI_ISL_614541, EPI_ISL_614542, EPI_ISL_614543, EPI_ISL_614544, EPI_ISL_614545, EPI_ISL_614546, EPI_ISL_614547, EPI_ISL_614548, EPI_ISL_614549, EPI_ISL_614550, EPI_ISL_614551, EPI_ISL_614552, EPI_ISL_614553, EPI_ISL_614554, EPI_ISL_614555, EPI_ISL_614556, EPI_ISL_614557, EPI_ISL_614558, EPI_ISL_614559, EPI_ISL_614560, EPI_ISL_614561, EPI_ISL_614562, EPI_ISL_614563, EPI_ISL_614564, EPI_ISL_614565, EPI_ISL_614566, EPI_ISL_614567, EPI_ISL_614568, EPI_ISL_614569, EPI_ISL_614570, EPI_ISL_614571, EPI_ISL_614572, EPI_ISL_614573, EPI_ISL_614574, EPI_ISL_614575, EPI_ISL_614576, EPI_ISL_614577, EPI_ISL_614578, EPI_ISL_614579, EPI_ISL_614580, EPI_ISL_614581, EPI_ISL_614582, EPI_ISL_614583, EPI_ISL_614584, EPI_ISL_614585, EPI_ISL_614586, EPI_ISL_614587, EPI_ISL_614588, EPI_ISL_614589, EPI_ISL_614590, EPI_ISL_614591, EPI_ISL_614592, EPI_ISL_614593, EPI_ISL_614594, EPI_ISL_614595, EPI_ISL_614596, EPI_ISL_614597, EPI_ISL_614598, EPI_ISL_614599, EPI_ISL_614600, EPI_ISL_614601, EPI_ISL_614602, EPI_ISL_614603, EPI_ISL_614604, EPI_ISL_614605, EPI_ISL_614606, EPI_ISL_614607, EPI_ISL_614608, EPI_ISL_614609, EPI_ISL_614610, EPI_ISL_614611, EPI_ISL_614612, EPI_ISL_614613, EPI_ISL_614614, EPI_ISL_614615, EPI_ISL_614616, EPI_ISL_614617, EPI_ISL_614618, EPI_ISL_614619, EPI_ISL_614620, EPI_ISL_614621, EPI_ISL_614622, EPI_ISL_614623, EPI_ISL_614624, EPI_ISL_614625, EPI_ISL_614626, EPI_ISL_614627, EPI_ISL_614628, EPI_ISL_614629, EPI_ISL_614630, EPI_ISL_614631, EPI_ISL_614632, EPI_ISL_614633, EPI_ISL_614634, EPI_ISL_614635, EPI_ISL_614636, EPI_ISL_614637, EPI_ISL_614638, EPI_ISL_614639, EPI_ISL_614640, EPI_ISL_614641, EPI_ISL_614642, EPI_ISL_614643, EPI_ISL_614644, EPI_ISL_614645, EPI_ISL_614646, EPI_ISL_614647, EPI_ISL_614648, EPI_ISL_614649, EPI_ISL_614650, EPI_ISL_614651, EPI_ISL_614652, EPI_ISL_614653, EPI_ISL_614654, EPI_ISL_614655, EPI_ISL_614656, EPI_ISL_614657, EPI_ISL_614658, EPI_ISL_614659, EPI_ISL_614660, EPI_ISL_614661, EPI_ISL_614662, EPI_ISL_614663, EPI_ISL_614664, EPI_ISL_614665, EPI_ISL_614666, EPI_ISL_614667, EPI_ISL_614668, EPI_ISL_614669, EPI_ISL_614670, EPI_ISL_614671, EPI_ISL_614672, EPI_ISL_614673, EPI_ISL_614674, EPI_ISL_614675, EPI_ISL_614676, EPI_ISL_614677, EPI_ISL_614678, EPI_ISL_614679, EPI_ISL_614680, EPI_ISL_614681, EPI_ISL_614682, EPI_ISL_614683, EPI_ISL_614684, EPI_ISL_614685, EPI_ISL_614686, EPI_ISL_614687, EPI_ISL_614688, EPI_ISL_614689, EPI_ISL_614690, EPI_ISL_614691, EPI_ISL_614692, EPI_ISL_614693, EPI_ISL_614694, EPI_ISL_614695, EPI_ISL_614696, EPI_ISL_614697, EPI_ISL_614698, EPI_ISL_614699, EPI_ISL_614700, EPI_ISL_614701, EPI_ISL_614702, EPI_ISL_614703, EPI_ISL_614704, EPI_ISL_614705, EPI_ISL_614706, EPI_ISL_614707, EPI_ISL_614708, EPI_ISL_614709, EPI_ISL_614710, EPI_ISL_614711, EPI_ISL_614712, EPI_ISL_614713, EPI_ISL_614714, EPI_ISL_614715, EPI_ISL_614716, EPI_ISL_614717, EPI_ISL_614718, EPI_ISL_614719, EPI_ISL_614720, EPI_ISL_614721, EPI_ISL_614722, EPI_ISL_614723, EPI_ISL_614724, EPI_ISL_614725, EPI_ISL_614726, EPI_ISL_614727, EPI_ISL_614728, EPI_ISL_614729, EPI_ISL_614730, EPI_ISL_614731, EPI_ISL_614732, EPI_ISL_614733, EPI_ISL_614734, EPI_ISL_614735, EPI_ISL_614736, EPI_ISL_614737, EPI_ISL_614738, EPI_ISL_614739, EPI_ISL_614740, EPI_ISL_614741, EPI_ISL_614742, EPI_ISL_614743, EPI_ISL_614744, EPI_ISL_614745, EPI_ISL_614746, EPI_ISL_614747, EPI_ISL_614748, EPI_ISL_614749, EPI_ISL_614750, EPI_ISL_614751, EPI_ISL_614752, EPI_ISL_614753, EPI_ISL_614754, EPI_ISL_614755, EPI_ISL_614756, EPI_ISL_614757, EPI_ISL_614758, EPI_ISL_614759, EPI_ISL_614760, EPI_ISL_614761, EPI_ISL_614762, EPI_ISL_614764, EPI_ISL_614765, EPI_ISL_614766, EPI_ISL_614767, EPI_ISL_614768, EPI_ISL_614769, EPI_ISL_614770, EPI_ISL_614771, EPI_ISL_614772, EPI_ISL_614773, EPI_ISL_614774, EPI_ISL_614775, EPI_ISL_614776, EPI_ISL_614777, EPI_ISL_614778, EPI_ISL_614779, EPI_ISL_614780, EPI_ISL_614781, EPI_ISL_614782, EPI_ISL_614783, EPI_ISL_614784, EPI_ISL_614785, EPI_ISL_614786, EPI_ISL_614787, EPI_ISL_614788, EPI_ISL_614789, EPI_ISL_614790, EPI_ISL_614791, EPI_ISL_614792, EPI_ISL_614793, EPI_ISL_614794, EPI_ISL_614795, EPI_ISL_614796, EPI_ISL_614797, EPI_ISL_614798, EPI_ISL_614799, EPI_ISL_614800, EPI_ISL_614801, EPI_ISL_614802, EPI_ISL_614803, EPI_ISL_614804, EPI_ISL_614805, EPI_ISL_614806, EPI_ISL_614807, EPI_ISL_614808, EPI_ISL_614809, EPI_ISL_614810, EPI_ISL_614811, EPI_ISL_614812, EPI_ISL_614813, EPI_ISL_614814, EPI_ISL_614815, EPI_ISL_614816, EPI_ISL_614817, EPI_ISL_614818, EPI_ISL_614819, EPI_ISL_614820, EPI_ISL_614821, EPI_ISL_614822, EPI_ISL_614823, EPI_ISL_614824, EPI_ISL_614825, EPI_ISL_614826, EPI_ISL_614827, EPI_ISL_614828, EPI_ISL_614829, EPI_ISL_614830, EPI_ISL_614831, EPI_ISL_614832, EPI_ISL_614833, EPI_ISL_614834, EPI_ISL_614835, EPI_ISL_614836, EPI_ISL_614837, EPI_ISL_614838, EPI_ISL_614839, EPI_ISL_614840, EPI_ISL_614841, EPI_ISL_614842, EPI_ISL_614843, EPI_ISL_614844, EPI_ISL_614845, EPI_ISL_614846, EPI_ISL_614847, EPI_ISL_614848, EPI_ISL_614849, EPI_ISL_614850, EPI_ISL_614851, EPI_ISL_614852, EPI_ISL_614853, EPI_ISL_614854, EPI_ISL_614855, EPI_ISL_614856, EPI_ISL_614857, EPI_ISL_614858, EPI_ISL_614859, EPI_ISL_614860, EPI_ISL_614861, EPI_ISL_614862, EPI_ISL_614863, EPI_ISL_614864, EPI_ISL_614865, EPI_ISL_614866, EPI_ISL_614867, EPI_ISL_614868, EPI_ISL_614869, EPI_ISL_614870, EPI_ISL_614871, EPI_ISL_614872, EPI_ISL_614873, EPI_ISL_614874, EPI_ISL_614875, EPI_ISL_614876, EPI_ISL_614877, EPI_ISL_614878, EPI_ISL_614879, EPI_ISL_614880, EPI_ISL_614881, EPI_ISL_614882, EPI_ISL_614883, EPI_ISL_614884, EPI_ISL_614885, EPI_ISL_614886, EPI_ISL_614887, EPI_ISL_614888			
see above	Department of Virus and Microbiological Special Diagnostics, Statens Serum Institut, Denmark	Albertsen lab, Department of Chemistry and Bioscience, Aalborg University, Denmark	Danish Covid-19 Genome Consortia
EPI_ISL_614889	Laboratorio Biologia Molecolare Sars Cov2 - UOC Laboratorio Analisi - Servizio Medicina di Laboratorio, Ospedale "San Francesco" - ATS-ASSL Nuoro	Laboratorio specialistico UOC Ematologia - Ospedale "San Francesco" - ATS-ASSL Nuoro	Piras Giovanna, Fancello Tatiana, Asproni Rosanna, Fiamma Maura, Monne Maria Itria, Toja Alessandro, Sanna Filomena, Floris Anna Rita, Sulis Vincenzo, Palmas Angelo Domenico, Casu Gavino, Lo Maglio Iana, Mameli Giuseppe
EPI_ISL_614893, EPI_ISL_614894, EPI_ISL_614895, EPI_ISL_614896, EPI_ISL_614897, EPI_ISL_614898, EPI_ISL_614899, EPI_ISL_614900, EPI_ISL_614901, EPI_ISL_614902, EPI_ISL_614903, EPI_ISL_614904, EPI_ISL_614905, EPI_ISL_614906, EPI_ISL_614907, EPI_ISL_614908, EPI_ISL_614909, EPI_ISL_614910, EPI_ISL_614911, EPI_ISL_614912, EPI_ISL_614913, EPI_ISL_614914, EPI_ISL_614915, EPI_ISL_614916, EPI_ISL_614917, EPI_ISL_614918, EPI_ISL_614919, EPI_ISL_614920, EPI_ISL_614921, EPI_ISL_614922, EPI_ISL_614923, EPI_ISL_614924, EPI_ISL_614925, EPI_ISL_614926, EPI_ISL_614927, EPI_ISL_614928, EPI_ISL_614930, EPI_ISL_614931, EPI_ISL_614932, EPI_ISL_614933, EPI_ISL_614934, EPI_ISL_614935, EPI_ISL_614936, EPI_ISL_614937, EPI_ISL_614938, EPI_ISL_614939, EPI_ISL_614940, EPI_ISL_614941, EPI_ISL_614942, EPI_ISL_614943, EPI_ISL_614944, EPI_ISL_614945, EPI_ISL_614946, EPI_ISL_614947, EPI_ISL_614948, EPI_ISL_614949, EPI_ISL_614950, EPI_ISL_614951, EPI_ISL_614952, EPI_ISL_614953, EPI_ISL_614954, EPI_ISL_614955, EPI_ISL_614956, EPI_ISL_614957, EPI_ISL_614958, EPI_ISL_614959, EPI_ISL_614960, EPI_ISL_614961, EPI_ISL_614962, EPI_ISL_614963, EPI_ISL_614964, EPI_ISL_614965, EPI_ISL_614966, EPI_ISL_614967, EPI_ISL_614968, EPI_ISL_614969, EPI_ISL_614970, EPI_ISL_614971, EPI_ISL_614972, EPI_ISL_614974, EPI_ISL_614975, EPI_ISL_614976, EPI_ISL_614977, EPI_ISL_614978, EPI_ISL_614979			
see above	Viollier AG	Department of Biosystems Science and Engineering, ETH Zürich	Christian Beisel, Sarah Nadeau, Ivan Topolsky, Pedro Ferreira, Philipp Jablonski, Susana Posada-Céspedes, Tobias Schär, Ina Nissen, Natascha Santacroce, Elodie Burcklen, Christiane Beckmann, Maurice Redondo, Olivier Kobel, Christoph Noppen, Sophie Seidel, Noemie Santamaria de Souza, Chaoran Chen, Niko Beerenwinkel, Tanja Stadler
EPI_ISL_614982, EPI_ISL_614983, EPI_ISL_614984, EPI_ISL_614985, EPI_ISL_614986, EPI_ISL_614987, EPI_ISL_614988, EPI_ISL_614989, EPI_ISL_614990, EPI_ISL_614991, EPI_ISL_614992, EPI_ISL_614993, EPI_ISL_614994, EPI_ISL_614997, EPI_ISL_614998, EPI_ISL_614999, EPI_ISL_615000, EPI_ISL_615001, EPI_ISL_615002, EPI_ISL_615003, EPI_ISL_615005, EPI_ISL_615006, EPI_ISL_615007, EPI_ISL_615008, EPI_ISL_615009, EPI_ISL_615010, EPI_ISL_615011, EPI_ISL_615012, EPI_ISL_615013, EPI_ISL_615014, EPI_ISL_615015, EPI_ISL_615016, EPI_ISL_615017, EPI_ISL_615018, EPI_ISL_615019, EPI_ISL_615020, EPI_ISL_615021, EPI_ISL_615023, EPI_ISL_615024, EPI_ISL_615025, EPI_ISL_615026, EPI_ISL_615027, EPI_ISL_615028, EPI_ISL_615029, EPI_ISL_615030, EPI_ISL_615031, EPI_ISL_615032, EPI_ISL_615033, EPI_ISL_615034, EPI_ISL_615035, EPI_ISL_615037, EPI_ISL_615038, EPI_ISL_615039, EPI_ISL_615040, EPI_ISL_615041, EPI_ISL_615042, EPI_ISL_615044, EPI_ISL_615045, EPI_ISL_615046, EPI_ISL_615047, EPI_ISL_615048, EPI_ISL_615049, EPI_ISL_615051, EPI_ISL_615053, EPI_ISL_615054, EPI_ISL_615055, EPI_ISL_615056, EPI_ISL_615057, EPI_ISL_615058, EPI_ISL_615059, EPI_ISL_615061, EPI_ISL_615062			
see above	Microbiology, Department of Pathology, St. Bernard's Hospital, Gibraltar Health Authority	Respiratory Virus Unit, Microbiology Services Colindale, Public Health England	PHE Covid Sequencing Team, Dr Nicholas Cortes (Gibraltar), Charlotte Gillborn-Jones (Gibraltar)
EPI_ISL_615076, EPI_ISL_615077, EPI_ISL_615078	Uppsala klinisk mikrobiologi	The Public Health Agency of Sweden	Anna-Malin Linde, Maria Lind Karlberg, Mattias Haukland, Reza Advani, Olov Svartstrom, Oskar Karlsson Lindsjo, Sandra Broddesson, Petra Edquist, Mia Brytting, Anna Risberg, Karin Tegmark-Wisell
EPI_ISL_615079	Orebro klinisk mikrobiologi	The Public Health Agency of Sweden	Anna-Malin Linde, Maria Lind Karlberg, Mattias Haukland, Reza Advani, Olov Svartstrom, Oskar Karlsson Lindsjo, Sandra Broddesson, Petra Edquist, Mia Brytting, Anna Risberg, Karin Tegmark-Wisell
EPI_ISL_615080, EPI_ISL_615081, EPI_ISL_615082, EPI_ISL_615083, EPI_ISL_615084, EPI_ISL_615085	Klinisk mikrobiologi, Skanes universitetssjukhus, Lund	The Public Health Agency of Sweden	Anna-Malin Linde, Maria Lind Karlberg, Mattias Haukland, Reza Advani, Olov Svartstrom, Oskar Karlsson Lindsjo, Sandra Broddesson, Petra Edquist, Mia Brytting, Anna Risberg, Karin Tegmark-Wisell
EPI_ISL_615086, EPI_ISL_615087, EPI_ISL_615088, EPI_ISL_615089	Karolinska Universitetslaboratoriet	The Public Health Agency of Sweden	Anna-Malin Linde, Maria Lind Karlberg, Mattias Haukland, Reza Advani, Olov Svartstrom, Oskar Karlsson Lindsjo, Sandra Broddesson, Petra Edquist, Mia Brytting, Anna Risberg, Karin Tegmark-Wisell
EPI_ISL_615090, EPI_ISL_615091	Klinisk mikrobiologi NAL Trollhattan	The Public Health Agency of Sweden	Anna-Malin Linde, Maria Lind Karlberg, Mattias Haukland, Reza Advani, Olov Svartstrom, Oskar Karlsson Lindsjo, Sandra Broddesson, Petra Edquist, Mia

			Brytting, Anna Risberg, Karin Tegmark-Wisell
EPI_ISL_615092, EPI_ISL_615093, EPI_ISL_615094, EPI_ISL_615095, EPI_ISL_615096	Umea klinisk mikrobiologi	The Public Health Agency of Sweden	Anna-Malin Linde, Maria Lind Karlberg, Mattias Haukland, Reza Advani, Olov Svartstrom, Oskar Karlsson Lindsjo, Sandra Broddesson, Petra Edquist, Mia Brytting, Anna Risberg, Karin Tegmark-Wisell
EPI_ISL_615097, EPI_ISL_615098, EPI_ISL_615099, EPI_ISL_615100	Klinisk mikrobiologi Lanssjukhuset Ryhov, Jonkoping	The Public Health Agency of Sweden	Anna-Malin Linde, Maria Lind Karlberg, Mattias Haukland, Reza Advani, Olov Svartstrom, Oskar Karlsson Lindsjo, Sandra Broddesson, Petra Edquist, Mia Brytting, Anna Risberg, Karin Tegmark-Wisell
EPI_ISL_615101, EPI_ISL_615102, EPI_ISL_615103	Gavle klinisk mikrobiologi	The Public Health Agency of Sweden	Anna-Malin Linde, Maria Lind Karlberg, Mattias Haukland, Reza Advani, Olov Svartstrom, Oskar Karlsson Lindsjo, Sandra Broddesson, Petra Edquist, Mia Brytting, Anna Risberg, Karin Tegmark-Wisell
EPI_ISL_615104, EPI_ISL_615105, EPI_ISL_615106, EPI_ISL_615107	Halmstad klinisk mikrobiologi	The Public Health Agency of Sweden	Anna-Malin Linde, Maria Lind Karlberg, Mattias Haukland, Reza Advani, Olov Svartstrom, Oskar Karlsson Lindsjo, Sandra Broddesson, Petra Edquist, Mia Brytting, Anna Risberg, Karin Tegmark-Wisell
EPI_ISL_615108	Klinisk Mikrobiologi	The Public Health Agency of Sweden	Anna-Malin Linde, Maria Lind Karlberg, Mattias Haukland, Reza Advani, Olov Svartstrom, Oskar Karlsson Lindsjo, Sandra Broddesson, Petra Edquist, Mia Brytting, Anna Risberg, Karin Tegmark-Wisell
EPI_ISL_615109	Klinisk mikrobiologi NAL Trollhattan	The Public Health Agency of Sweden	Anna-Malin Linde, Maria Lind Karlberg, Mattias Haukland, Reza Advani, Olov Svartstrom, Oskar Karlsson Lindsjo, Sandra Broddesson, Petra Edquist, Mia Brytting, Anna Risberg, Karin Tegmark-Wisell
EPI_ISL_615110	Klinsisk mikrobiologi Linkoping	The Public Health Agency of Sweden	Anna-Malin Linde, Maria Lind Karlberg, Mattias Haukland, Reza Advani, Olov Svartstrom, Oskar Karlsson Lindsjo, Sandra Broddesson, Petra Edquist, Mia Brytting, Anna Risberg, Karin Tegmark-Wisell
EPI_ISL_615111, EPI_ISL_615112, EPI_ISL_615113, EPI_ISL_615114	Orebro klinisk mikrobiologi	The Public Health Agency of Sweden	Anna-Malin Linde, Maria Lind Karlberg, Mattias Haukland, Reza Advani, Olov Svartstrom, Oskar Karlsson Lindsjo, Sandra Broddesson, Petra Edquist, Mia Brytting, Anna Risberg, Karin Tegmark-Wisell
EPI_ISL_615115, EPI_ISL_615116	Skovde/Unilabs	The Public Health Agency of Sweden	Anna-Malin Linde, Maria Lind Karlberg, Mattias Haukland, Reza Advani, Olov Svartstrom, Oskar Karlsson Lindsjo, Sandra Broddesson, Petra Edquist, Mia Brytting, Anna Risberg, Karin Tegmark-Wisell
EPI_ISL_615117, EPI_ISL_615118, EPI_ISL_615119	Klinsisk mikrobiologi Linkoping	The Public Health Agency of Sweden	Anna-Malin Linde, Maria Lind Karlberg, Mattias Haukland, Reza Advani, Olov Svartstrom, Oskar Karlsson Lindsjo, Sandra Broddesson, Petra Edquist, Mia Brytting, Anna Risberg, Karin Tegmark-Wisell
EPI_ISL_615120	Orebro klinisk mikrobiologi	The Public Health Agency of Sweden	Anna-Malin Linde, Maria Lind Karlberg, Mattias Haukland, Reza Advani, Olov Svartstrom, Oskar Karlsson Lindsjo, Sandra Broddesson, Petra Edquist, Mia Brytting, Anna Risberg, Karin Tegmark-Wisell
EPI_ISL_615121	Hospital de Pediatría "Prof. Dr. Juan P Garrahan"	Héritas	Cristian Rohr, Bianca Brun, Dalmacio Pereyra, Priscila Aldabe, Andrea Mangano, Maria Florencia Fernandez, Fabian Fay, Martin Vazquez
EPI_ISL_615139, EPI_ISL_615158	Texas Department of State Health Services	Texas Department of State Health Services	Rashmi Tuladhar, Bonnie Oh, Jenny Zhang, Maliha Rahman, Anita Pokharel, Myong Koag, Chung Wang, Rachel Lee, Grace Kubin, Mayela Pedrueza

EPI_ISL_615166, EPI_ISL_615167, EPI_ISL_615168, EPI_ISL_615169, EPI_ISL_615170, EPI_ISL_615171, EPI_ISL_615172, EPI_ISL_615173, EPI_ISL_615174, EPI_ISL_615175, EPI_ISL_615176, EPI_ISL_615177, EPI_ISL_615178, EPI_ISL_615179, EPI_ISL_615180, EPI_ISL_615181, EPI_ISL_615182, EPI_ISL_615183,
EPI_ISL_615184, EPI_ISL_615185, EPI_ISL_615186, EPI_ISL_615187, EPI_ISL_615188, EPI_ISL_615189, EPI_ISL_615190, EPI_ISL_615191, EPI_ISL_615192, EPI_ISL_615193, EPI_ISL_615194, EPI_ISL_615195, EPI_ISL_615196, EPI_ISL_615197, EPI_ISL_615198, EPI_ISL_615199, EPI_ISL_615200, EPI_ISL_615201,
EPI_ISL_615202, EPI_ISL_615203, EPI_ISL_615204, EPI_ISL_615205, EPI_ISL_615206, EPI_ISL_615207, EPI_ISL_615208, EPI_ISL_615209, EPI_ISL_615210, EPI_ISL_615211, EPI_ISL_615212, EPI_ISL_615213, EPI_ISL_615214, EPI_ISL_615215, EPI_ISL_615216, EPI_ISL_615217, EPI_ISL_615220, EPI_ISL_615221,
EPI_ISL_615222, EPI_ISL_615223, EPI_ISL_615224, EPI_ISL_615225, EPI_ISL_615226, EPI_ISL_615227, EPI_ISL_615228, EPI_ISL_615229, EPI_ISL_615230, EPI_ISL_615231, EPI_ISL_615232, EPI_ISL_615233, EPI_ISL_615234, EPI_ISL_615235, EPI_ISL_615236, EPI_ISL_615237, EPI_ISL_615238, EPI_ISL_615239,
EPI_ISL_615241, EPI_ISL_615242, EPI_ISL_615243, EPI_ISL_615244, EPI_ISL_615246, EPI_ISL_615247, EPI_ISL_615248, EPI_ISL_615249, EPI_ISL_615250, EPI_ISL_615251, EPI_ISL_615252, EPI_ISL_615253, EPI_ISL_615254, EPI_ISL_615255, EPI_ISL_615256, EPI_ISL_615257, EPI_ISL_615258, EPI_ISL_615259,
EPI_ISL_615260, EPI_ISL_615261, EPI_ISL_615262, EPI_ISL_615264, EPI_ISL_615265, EPI_ISL_615266, EPI_ISL_615267, EPI_ISL_615268, EPI_ISL_615269, EPI_ISL_615270, EPI_ISL_615272, EPI_ISL_615273, EPI_ISL_615274, EPI_ISL_615275, EPI_ISL_615276, EPI_ISL_615277, EPI_ISL_615278, EPI_ISL_615279,
EPI_ISL_615280, EPI_ISL_615281, EPI_ISL_615282, EPI_ISL_615283, EPI_ISL_615284, EPI_ISL_615285, EPI_ISL_615286, EPI_ISL_615287, EPI_ISL_615288, EPI_ISL_615289, EPI_ISL_615290, EPI_ISL_615291, EPI_ISL_615292, EPI_ISL_615293, EPI_ISL_615294, EPI_ISL_615295, EPI_ISL_615297, EPI_ISL_615299,
EPI_ISL_615302, EPI_ISL_615303, EPI_ISL_615304, EPI_ISL_615305, EPI_ISL_615306, EPI_ISL_615307, EPI_ISL_615308, EPI_ISL_615309, EPI_ISL_615310, EPI_ISL_615311, EPI_ISL_615312, EPI_ISL_615313, EPI_ISL_615315, EPI_ISL_615316, EPI_ISL_615317, EPI_ISL_615319, EPI_ISL_615320, EPI_ISL_615322,
EPI_ISL_615323, EPI_ISL_615324, EPI_ISL_615325, EPI_ISL_615326, EPI_ISL_615327, EPI_ISL_615328, EPI_ISL_615329, EPI_ISL_615330, EPI_ISL_615331, EPI_ISL_615332, EPI_ISL_615333, EPI_ISL_615334, EPI_ISL_615336, EPI_ISL_615337, EPI_ISL_615338, EPI_ISL_615339, EPI_ISL_615340, EPI_ISL_615341,
EPI_ISL_615342, EPI_ISL_615343, EPI_ISL_615344, EPI_ISL_615346, EPI_ISL_615347, EPI_ISL_615348, EPI_ISL_615349, EPI_ISL_615350, EPI_ISL_615351, EPI_ISL_615352, EPI_ISL_615353, EPI_ISL_615354, EPI_ISL_615355, EPI_ISL_615356, EPI_ISL_615357, EPI_ISL_615358, EPI_ISL_615359, EPI_ISL_615361,
EPI_ISL_615362, EPI_ISL_615363, EPI_ISL_615364, EPI_ISL_615365, EPI_ISL_615366, EPI_ISL_615367, EPI_ISL_615368, EPI_ISL_615369, EPI_ISL_615370, EPI_ISL_615371, EPI_ISL_615372, EPI_ISL_615373, EPI_ISL_615374, EPI_ISL_615375, EPI_ISL_615376, EPI_ISL_615377, EPI_ISL_615378, EPI_ISL_615379,
EPI_ISL_615380, EPI_ISL_615381, EPI_ISL_615382, EPI_ISL_615383, EPI_ISL_615384, EPI_ISL_615385, EPI_ISL_615386, EPI_ISL_615387, EPI_ISL_615388, EPI_ISL_615389, EPI_ISL_615390, EPI_ISL_615391, EPI_ISL_615392, EPI_ISL_615394, EPI_ISL_615395, EPI_ISL_615396, EPI_ISL_615398, EPI_ISL_615399,
EPI_ISL_615400, EPI_ISL_615401, EPI_ISL_615402, EPI_ISL_615403, EPI_ISL_615404, EPI_ISL_615405, EPI_ISL_615406, EPI_ISL_615407, EPI_ISL_615408, EPI_ISL_615409, EPI_ISL_615410, EPI_ISL_615411, EPI_ISL_615412, EPI_ISL_615413, EPI_ISL_615414, EPI_ISL_615415, EPI_ISL_615416, EPI_ISL_615417,
EPI_ISL_615418, EPI_ISL_615419, EPI_ISL_615420, EPI_ISL_615421, EPI_ISL_615422, EPI_ISL_615423, EPI_ISL_615424, EPI_ISL_615425, EPI_ISL_615426, EPI_ISL_615427, EPI_ISL_615428, EPI_ISL_615429, EPI_ISL_615430, EPI_ISL_615431, EPI_ISL_615432, EPI_ISL_615433, EPI_ISL_615434, EPI_ISL_615435,
EPI_ISL_615437, EPI_ISL_615439, EPI_ISL_615440, EPI_ISL_615441, EPI_ISL_615442, EPI_ISL_615443, EPI_ISL_615444, EPI_ISL_615445, EPI_ISL_615446, EPI_ISL_615447, EPI_ISL_615450, EPI_ISL_615451, EPI_ISL_615452, EPI_ISL_615453, EPI_ISL_615454, EPI_ISL_615455, EPI_ISL_615457, EPI_ISL_615458,
EPI_ISL_615459, EPI_ISL_615460, EPI_ISL_615461, EPI_ISL_615463, EPI_ISL_615464, EPI_ISL_615465, EPI_ISL_615466, EPI_ISL_615467, EPI_ISL_615468, EPI_ISL_615470, EPI_ISL_615471, EPI_ISL_615472, EPI_ISL_615473, EPI_ISL_615474, EPI_ISL_615475, EPI_ISL_615477, EPI_ISL_615478, EPI_ISL_615479,
EPI_ISL_615480, EPI_ISL_615481, EPI_ISL_615482, EPI_ISL_615483, EPI_ISL_615484, EPI_ISL_615485, EPI_ISL_615486, EPI_ISL_615487, EPI_ISL_615488, EPI_ISL_615489, EPI_ISL_615490, EPI_ISL_615491, EPI_ISL_615492, EPI_ISL_615493, EPI_ISL_615494, EPI_ISL_615495, EPI_ISL_615496, EPI_ISL_615497,
EPI_ISL_615498, EPI_ISL_615499, EPI_ISL_615501, EPI_ISL_615502, EPI_ISL_615503, EPI_ISL_615504, EPI_ISL_615505, EPI_ISL_615507, EPI_ISL_615508, EPI_ISL_615509, EPI_ISL_615510, EPI_ISL_615511, EPI_ISL_615512, EPI_ISL_615513, EPI_ISL_615514, EPI_ISL_615515, EPI_ISL_615516, EPI_ISL_615517,
EPI_ISL_615518, EPI_ISL_615519, EPI_ISL_615520, EPI_ISL_615521, EPI_ISL_615522, EPI_ISL_615523, EPI_ISL_615524, EPI_ISL_615525, EPI_ISL_615526, EPI_ISL_615527, EPI_ISL_615528, EPI_ISL_615530, EPI_ISL_615531, EPI_ISL_615532, EPI_ISL_615533, EPI_ISL_615534, EPI_ISL_615535, EPI_ISL_615536,
EPI_ISL_615537, EPI_ISL_615538, EPI_ISL_615539, EPI_ISL_615540, EPI_ISL_615541, EPI_ISL_615542, EPI_ISL_615544, EPI_ISL_615545, EPI_ISL_615546, EPI_ISL_615547, EPI_ISL_615548, EPI_ISL_615549, EPI_ISL_615550, EPI_ISL_615552, EPI_ISL_615553, EPI_ISL_615554, EPI_ISL_615555, EPI_ISL_615556,
EPI_ISL_615557, EPI_ISL_615558, EPI_ISL_615561, EPI_ISL_615562, EPI_ISL_615563, EPI_ISL_615564, EPI_ISL_615565, EPI_ISL_615566, EPI_ISL_615569, EPI_ISL_615571, EPI_ISL_615572, EPI_ISL_615574, EPI_ISL_615575, EPI_ISL_615576, EPI_ISL_615577, EPI_ISL_615578, EPI_ISL_615579, EPI_ISL_615580,
EPI_ISL_615581, EPI_ISL_615582, EPI_ISL_615583, EPI_ISL_615584, EPI_ISL_615586, EPI_ISL_615587, EPI_ISL_615588, EPI_ISL_615589, EPI_ISL_615590, EPI_ISL_615591, EPI_ISL_615592, EPI_ISL_615594, EPI_ISL_615595, EPI_ISL_615596, EPI_ISL_615597, EPI_ISL_615598, EPI_ISL_615599, EPI_ISL_615600,
EPI_ISL_615601, EPI_ISL_615602, EPI_ISL_615603, EPI_ISL_615604, EPI_ISL_615605, EPI_ISL_615608, EPI_ISL_615609, EPI_ISL_615610, EPI_ISL_615611, EPI_ISL_615613, EPI_ISL_615614, EPI_ISL_615615, EPI_ISL_615616, EPI_ISL_615617, EPI_ISL_615618, EPI_ISL_615620, EPI_ISL_615621, EPI_ISL_615622,
EPI_ISL_615623, EPI_ISL_615624, EPI_ISL_615625, EPI_ISL_615626, EPI_ISL_615627, EPI_ISL_615628, EPI_ISL_615629, EPI_ISL_615630, EPI_ISL_615631, EPI_ISL_615632, EPI_ISL_615633, EPI_ISL_615634, EPI_ISL_615635, EPI_ISL_615637, EPI_ISL_615638, EPI_ISL_615639, EPI_ISL_615640, EPI_ISL_615641,
EPI_ISL_615642, EPI_ISL_615643, EPI_ISL_615644, EPI_ISL_615645, EPI_ISL_615646, EPI_ISL_615647, EPI_ISL_615648, EPI_ISL_615649, EPI_ISL_615650, EPI_ISL_615654, EPI_ISL_615655, EPI_ISL_615656, EPI_ISL_615658, EPI_ISL_615659, EPI_ISL_615660, EPI_ISL_615662, EPI_ISL_615663, EPI_ISL_615664,
EPI_ISL_615665, EPI_ISL_615666, EPI_ISL_615668, EPI_ISL_615669, EPI_ISL_615670, EPI_ISL_615671, EPI_ISL_615672, EPI_ISL_615674, EPI_ISL_615675, EPI_ISL_615676, EPI_ISL_615677, EPI_ISL_615679, EPI_ISL_615680, EPI_ISL_615681, EPI_ISL_615682, EPI_ISL_615683, EPI_ISL_615685, EPI_ISL_615686,
EPI_ISL_615687, EPI_ISL_615689, EPI_ISL_615690, EPI_ISL_615691, EPI_ISL_615692, EPI_ISL_615693, EPI_ISL_615694, EPI_ISL_615695, EPI_ISL_615699, EPI_ISL_615700, EPI_ISL_615701, EPI_ISL_615703, EPI_ISL_615704, EPI_ISL_615705, EPI_ISL_615706, EPI_ISL_615707, EPI_ISL_615708, EPI_ISL_615710,
EPI_ISL_615711, EPI_ISL_615712, EPI_ISL_615714, EPI_ISL_615715, EPI_ISL_615716, EPI_ISL_615717, EPI_ISL_615719, EPI_ISL_615720, EPI_ISL_615721, EPI_ISL_615722, EPI_ISL_615723, EPI_ISL_615724, EPI_ISL_615725, EPI_ISL_615727, EPI_ISL_615728, EPI_ISL_615730, EPI_ISL_615731, EPI_ISL_615732,
EPI_ISL_615733, EPI_ISL_615734, EPI_ISL_615735, EPI_ISL_615736, EPI_ISL_615737, EPI_ISL_615739, EPI_ISL_615740, EPI_ISL_615742, EPI_ISL_615744, EPI_ISL_615745, EPI_ISL_615746, EPI_ISL_615747, EPI_ISL_615748, EPI_ISL_615749, EPI_ISL_615750, EPI_ISL_615751, EPI_ISL_615752, EPI_ISL_615754,
EPI_ISL_615755, EPI_ISL_615756, EPI_ISL_615757, EPI_ISL_615758, EPI_ISL_615759, EPI_ISL_615760, EPI_ISL_615761, EPI_ISL_615762, EPI_ISL_615763, EPI_ISL_615764, EPI_ISL_615765, EPI_ISL_615766, EPI_ISL_615767, EPI_ISL_615768, EPI_ISL_615769, EPI_ISL_615770, EPI_ISL_615771, EPI_ISL_615772,
EPI_ISL_615773, EPI_ISL_615774, EPI_ISL_615776, EPI_ISL_615777, EPI_ISL_615779, EPI_ISL_615780, EPI_ISL_615781, EPI_ISL_615782, EPI_ISL_615783, EPI_ISL_615784, EPI_ISL_615785, EPI_ISL_615786, EPI_ISL_615787, EPI_ISL_615788, EPI_ISL_615789, EPI_ISL_615790, EPI_ISL_615791, EPI_ISL_615792,
EPI_ISL_615793, EPI_ISL_615794, EPI_ISL_615795, EPI_ISL_615796, EPI_ISL_615797, EPI_ISL_615798, EPI_ISL_615799, EPI_ISL_615800, EPI_ISL_615801, EPI_ISL_615802, EPI_ISL_615803, EPI_ISL_615804, EPI_ISL_615805, EPI_ISL_615806, EPI_ISL_615808, EPI_ISL_615809, EPI_ISL_615810, EPI_ISL_615811,
EPI_ISL_615812, EPI_ISL_615813, EPI_ISL_615814, EPI_ISL_615815, EPI_ISL_615816, EPI_ISL_615817, EPI_ISL_615818, EPI_ISL_615819, EPI_ISL_615820, EPI_ISL_615821, EPI_ISL_615822, EPI_ISL_615823, EPI_ISL_615824, EPI_ISL_615825, EPI_ISL_615826, EPI_ISL_615827, EPI_ISL_615828, EPI_ISL_615829,
EPI_ISL_615830, EPI_ISL_615831, EPI_ISL_615832, EPI_ISL_615833, EPI_ISL_615834, EPI_ISL_615835, EPI_ISL_615836, EPI_ISL_615837, EPI_ISL_615838, EPI_ISL_615840, EPI_ISL_615841, EPI_ISL_615842, EPI_ISL_615844, EPI_ISL_615845, EPI_ISL_615846, EPI_ISL_615847, EPI_ISL_615848, EPI_ISL_615849,
EPI_ISL_615850, EPI_ISL_615851, EPI_ISL_615852, EPI_ISL_615853, EPI_ISL_615854, EPI_ISL_615855, EPI_ISL_615856, EPI_ISL_615857, EPI_ISL_615858, EPI_ISL_615860, EPI_ISL_615861, EPI_ISL_615862, EPI_ISL_615863, EPI_ISL_615864, EPI_ISL_615865, EPI_ISL_615866, EPI_ISL_615867, EPI_ISL_615868,
EPI_ISL_615869, EPI_ISL_615870, EPI_ISL_615871, EPI_ISL_615872, EPI_ISL_615873, EPI_ISL_615875, EPI_ISL_615876, EPI_ISL_615879, EPI_ISL_615880, EPI_ISL_615881, EPI_ISL_615882, EPI_ISL_615883, EPI_ISL_615884, EPI_ISL_615885, EPI_ISL_615886, EPI_ISL_615887, EPI_ISL_615888, EPI_ISL_615889,
EPI_ISL_615890, EPI_ISL_615891, EPI_ISL_615892, EPI_ISL_615893, EPI_ISL_615894, EPI_ISL_615895, EPI_ISL_615896, EPI_ISL_615897, EPI_ISL_615898, EPI_ISL_615899, EPI_ISL_615900, EPI_ISL_615901, EPI_ISL_615902, EPI_ISL_615903, EPI_ISL_615904, EPI_ISL_615905, EPI_ISL_615907, EPI_ISL_615908,
EPI_ISL_615909, EPI_ISL_615910, EPI_ISL_615911, EPI_ISL_615912, EPI_ISL_615913, EPI_ISL_615914, EPI_ISL_615917, EPI_ISL_615918, EPI_ISL_615919, EPI_ISL_615920, EPI_ISL_615921, EPI_ISL_615923, EPI_ISL_615925, EPI_ISL_615926, EPI_ISL_615927, EPI_ISL_615928, EPI_ISL_615929, EPI_ISL_615930,
EPI_ISL_615931, EPI_ISL_615932, EPI_ISL_615933, EPI_ISL_615934, EPI_ISL_615937, EPI_ISL_615938, EPI_ISL_615939, EPI_ISL_615940, EPI_ISL_615941, EPI_ISL_615942, EPI_ISL_615943, EPI_ISL_615944, EPI_ISL_615945, EPI_ISL_615946, EPI_ISL_615947, EPI_ISL_615948, EPI_ISL_615949, EPI_ISL_615950,
EPI_ISL_615951, EPI_ISL_615952, EPI_ISL_615953, EPI_ISL_615954, EPI_ISL_615955, EPI_ISL_615956, EPI_ISL_615957, EPI_ISL_615959, EPI_ISL_615960, EPI_ISL_615961, EPI_ISL_615962, EPI_ISL_615965, EPI_ISL_615966, EPI_ISL_615967, EPI_ISL_615968, EPI_ISL_615969, EPI_ISL_615970, EPI_ISL_615971,
EPI_ISL_615973, EPI_ISL_615974, EPI_ISL_615975, EPI_ISL_615976, EPI_ISL_615977, EPI_ISL_615978, EPI_ISL_615979, EPI_ISL_615981, EPI_ISL_615982, EPI_ISL_615984, EPI_ISL_615985, EPI_ISL_615986, EPI_ISL_615987, EPI_ISL_615989, EPI_ISL_615991, EPI_ISL_615992, EPI_ISL_615993, EPI_ISL_615994,
EPI_ISL_615995, EPI_ISL_615997, EPI_ISL_615998, EPI_ISL_615999, EPI_ISL_616000, EPI_ISL_616001, EPI_ISL_616002, EPI_ISL_616003, EPI_ISL_616004, EPI_ISL_616005, EPI_ISL_616006, EPI_ISL_616007, EPI_ISL_616008, EPI_ISL_616009, EPI_ISL_616010, EPI_ISL_616012, EPI_ISL_616013, EPI_ISL_616014,
EPI_ISL_616015, EPI_ISL_616016, EPI_ISL_616017, EPI_ISL_616018, EPI_ISL_616019, EPI_ISL_616020, EPI_ISL_616021, EPI_ISL_616022, EPI_ISL_616023, EPI_ISL_616024, EPI_ISL_616025, EPI_ISL_616026, EPI_ISL_616027, EPI_ISL_616028, EPI_ISL_616029, EPI_ISL_616030, EPI_ISL_616031, EPI_ISL_616032,
EPI_ISL_616033, EPI_ISL_616035, EPI_ISL_616036, EPI_ISL_616037, EPI_ISL_616038, EPI_ISL_616039, EPI_ISL_616040, EPI_ISL_616041, EPI_ISL_616042, EPI_ISL_616043, EPI_ISL_616044, EPI_ISL_616045, EPI_ISL_616046, EPI_ISL_616047, EPI_ISL_616048, EPI_ISL_616050, EPI_ISL_616051, EPI_ISL_616052,
EPI_ISL_616053, EPI_ISL_616054, EPI_ISL_616055, EPI_ISL_616056, EPI_ISL_616057, EPI_ISL_616058, EPI_ISL_616059, EPI_ISL_616060, EPI_ISL_616061, EPI_ISL_616062, EPI_ISL_616063, EPI_ISL_616064, EPI_ISL_616066, EPI_ISL_616068, EPI_ISL_616069, EPI_ISL_616070, EPI_ISL_616071, EPI_ISL_616072,
EPI_ISL_616073, EPI_ISL_616074, EPI_ISL_616075, EPI_ISL_616076, EPI_ISL_616077, EPI_ISL_616078, EPI_ISL_616079, EPI_ISL_616080, EPI_ISL_616081, EPI_ISL_616082, EPI_ISL_616083, EPI_ISL_616084, EPI_ISL_616085, EPI_ISL_616086, EPI_ISL_616087, EPI_ISL_616088, EPI_ISL_616089, EPI_ISL_616090,
EPI_ISL_616091, EPI_ISL_616092, EPI_ISL_616093, EPI_ISL_616094, EPI_ISL_616095, EPI_ISL_616096, EPI_ISL_616097, EPI_ISL_616099, EPI_ISL_616100, EPI_ISL_616101, EPI_ISL_616102, EPI_ISL_616103, EPI_ISL_616104, EPI_ISL_616105, EPI_ISL_616106, EPI_ISL_616108, EPI_ISL_616109, EPI_ISL_616110,
EPI_ISL_616111, EPI_ISL_616112, EPI_ISL_616113, EPI_ISL_616114, EPI_ISL_616115, EPI_ISL_616116, EPI_ISL_616117, EPI_ISL_616118, EPI_ISL_616119, EPI_ISL_616120, EPI_ISL_616121, EPI_ISL_616122, EPI_ISL_616123, EPI_ISL_616124, EPI_ISL_616125, EPI_ISL_616126, EPI_ISL_616127, EPI_ISL_616128,
EPI_ISL_616129, EPI_ISL_616130, EPI_ISL_616131, EPI_ISL_616132, EPI_ISL_616133, EPI_ISL_616134, EPI_ISL_616135, EPI_ISL_616136, EPI_ISL_616137, EPI_ISL_616138, EPI_ISL_616139, EPI_ISL_616140, EPI_ISL_616141, EPI_ISL_616142, EPI_ISL_616143, EPI_ISL_616144, EPI_ISL_616145, EPI_ISL_616146,
EPI_ISL_616147, EPI_ISL_616148, EPI_ISL_616149, EPI_ISL_616150, EPI_ISL_616151, EPI_ISL_616152, EPI_ISL_616153, EPI_ISL_616154, EPI_ISL_616155, EPI_ISL_616156, EPI_ISL_616157, EPI_ISL_616158, EPI_ISL_616159, EPI_ISL_616160, EPI_ISL_616161, EPI_ISL_616162, EPI_ISL_616163, EPI_ISL_616164,
EPI_ISL_616165, EPI_ISL_616166, EPI_ISL_616167, EPI_ISL_616168, EPI_ISL_616169, EPI_ISL_616170, EPI_ISL_616171, EPI_ISL_616172, EPI_ISL_616173, EPI_ISL_616174, EPI_ISL_616176, EPI_ISL_616177, EPI_ISL_616178, EPI_ISL_616179, EPI_ISL_616180, EPI_ISL_616182, EPI_ISL_616183, EPI_ISL_616184,
EPI_ISL_616185, EPI_ISL_616186, EPI_ISL_616187, EPI_ISL_616188, EPI_ISL_616189, EPI_ISL_616192, EPI_ISL_616194, EPI_ISL_616195, EPI_ISL_616196, EPI_ISL_616197, EPI_ISL_616198, EPI_ISL_616199, EPI_ISL_616200, EPI_ISL_616201, EPI_ISL_616203, EPI_ISL_616204, EPI_ISL_616205, EPI_ISL_616206,
EPI_ISL_616207, EPI_ISL_616208, EPI_ISL_616209, EPI_ISL_616210, EPI_ISL_616211, EPI_ISL_616212, EPI_ISL_616213, EPI_ISL_616214, EPI_ISL_616215, EPI_ISL_616216, EPI_ISL_616217, EPI_ISL_616218, EPI_ISL_616219, EPI_ISL_616220, EPI_ISL_616221, EPI_ISL_616222, EPI_ISL_616223, EPI_ISL_616224,
EPI_ISL_616225, EPI_ISL_616226, EPI_ISL_616227, EPI_ISL_616228, EPI_ISL_616230, EPI_ISL_616231, EPI_ISL_616232, EPI_ISL_616233, EPI_ISL_616234, EPI_ISL_616235, EPI_ISL_616236, EPI_ISL_616237, EPI_ISL_616239, EPI_ISL_616240, EPI_ISL_616241, EPI_ISL_616242, EPI_ISL_616243, EPI_ISL_616244,
EPI_ISL_616245, EPI_ISL_616246, EPI_ISL_616247, EPI_ISL_616248, EPI_ISL_616249, EPI_ISL_616250, EPI_ISL_616251, EPI_ISL_616253, EPI_ISL_616254, EPI_ISL_616255, EPI_ISL_616256, EPI_ISL_616257, EPI_ISL_616258, EPI_ISL_616259, EPI_ISL_616260, EPI_ISL_616261, EPI_ISL_616262, EPI_ISL_616263,

EPI_ISL_616264, EPI_ISL_616265, EPI_ISL_616266, EPI_ISL_616267, EPI_ISL_616268, EPI_ISL_616270, EPI_ISL_616271, EPI_ISL_616272, EPI_ISL_616273, EPI_ISL_616274, EPI_ISL_616275, EPI_ISL_616276, EPI_ISL_616277, EPI_ISL_616278, EPI_ISL_616279, EPI_ISL_616280, EPI_ISL_616282, EPI_ISL_616283,
EPI_ISL_616284, EPI_ISL_616285, EPI_ISL_616286, EPI_ISL_616287, EPI_ISL_616288, EPI_ISL_616289, EPI_ISL_616290, EPI_ISL_616291, EPI_ISL_616293, EPI_ISL_616294, EPI_ISL_616296, EPI_ISL_616297, EPI_ISL_616298, EPI_ISL_616299, EPI_ISL_616300, EPI_ISL_616301, EPI_ISL_616303, EPI_ISL_616304,
EPI_ISL_616305, EPI_ISL_616306, EPI_ISL_616307, EPI_ISL_616308, EPI_ISL_616309, EPI_ISL_616310, EPI_ISL_616311, EPI_ISL_616312, EPI_ISL_616314, EPI_ISL_616315, EPI_ISL_616316, EPI_ISL_616317, EPI_ISL_616318, EPI_ISL_616319, EPI_ISL_616320, EPI_ISL_616321, EPI_ISL_616322, EPI_ISL_616323,
EPI_ISL_616324, EPI_ISL_616325, EPI_ISL_616326, EPI_ISL_616327, EPI_ISL_616328, EPI_ISL_616329, EPI_ISL_616330, EPI_ISL_616331, EPI_ISL_616332, EPI_ISL_616333, EPI_ISL_616334, EPI_ISL_616336, EPI_ISL_616337, EPI_ISL_616338, EPI_ISL_616339, EPI_ISL_616340, EPI_ISL_616341, EPI_ISL_616342,
EPI_ISL_616343, EPI_ISL_616345, EPI_ISL_616346, EPI_ISL_616347, EPI_ISL_616348, EPI_ISL_616349, EPI_ISL_616350, EPI_ISL_616351, EPI_ISL_616352, EPI_ISL_616353, EPI_ISL_616354, EPI_ISL_616356, EPI_ISL_616355, EPI_ISL_616356, EPI_ISL_616358, EPI_ISL_616359, EPI_ISL_616360, EPI_ISL_616361, EPI_ISL_616362,
EPI_ISL_616363, EPI_ISL_616364, EPI_ISL_616365, EPI_ISL_616366, EPI_ISL_616367, EPI_ISL_616368, EPI_ISL_616370, EPI_ISL_616371, EPI_ISL_616372, EPI_ISL_616373, EPI_ISL_616374, EPI_ISL_616375, EPI_ISL_616376, EPI_ISL_616377, EPI_ISL_616378, EPI_ISL_616379, EPI_ISL_616380, EPI_ISL_616381,
EPI_ISL_616382, EPI_ISL_616383, EPI_ISL_616384, EPI_ISL_616385, EPI_ISL_616386, EPI_ISL_616387, EPI_ISL_616388, EPI_ISL_616389, EPI_ISL_616390, EPI_ISL_616391, EPI_ISL_616392, EPI_ISL_616393, EPI_ISL_616394, EPI_ISL_616395, EPI_ISL_616396, EPI_ISL_616397, EPI_ISL_616398, EPI_ISL_616399,
EPI_ISL_616400, EPI_ISL_616401, EPI_ISL_616403, EPI_ISL_616404, EPI_ISL_616405, EPI_ISL_616406, EPI_ISL_616407, EPI_ISL_616409, EPI_ISL_616410, EPI_ISL_616411, EPI_ISL_616412, EPI_ISL_616413, EPI_ISL_616414, EPI_ISL_616415, EPI_ISL_616416, EPI_ISL_616417, EPI_ISL_616418, EPI_ISL_616419,
EPI_ISL_616420, EPI_ISL_616421, EPI_ISL_616422, EPI_ISL_616423, EPI_ISL_616424, EPI_ISL_616425, EPI_ISL_616426, EPI_ISL_616428, EPI_ISL_616429, EPI_ISL_616430, EPI_ISL_616432, EPI_ISL_616434, EPI_ISL_616435, EPI_ISL_616436, EPI_ISL_616437, EPI_ISL_616438, EPI_ISL_616439, EPI_ISL_616440,
EPI_ISL_616441, EPI_ISL_616443, EPI_ISL_616444, EPI_ISL_616445, EPI_ISL_616446, EPI_ISL_616447, EPI_ISL_616448, EPI_ISL_616449, EPI_ISL_616450, EPI_ISL_616451, EPI_ISL_616452, EPI_ISL_616453, EPI_ISL_616454, EPI_ISL_616455, EPI_ISL_616456, EPI_ISL_616457, EPI_ISL_616458, EPI_ISL_616460,
EPI_ISL_616461, EPI_ISL_616462, EPI_ISL_616463, EPI_ISL_616464, EPI_ISL_616465, EPI_ISL_616466, EPI_ISL_616467, EPI_ISL_616468, EPI_ISL_616470, EPI_ISL_616471, EPI_ISL_616472, EPI_ISL_616473, EPI_ISL_616474, EPI_ISL_616475, EPI_ISL_616477, EPI_ISL_616478, EPI_ISL_616479, EPI_ISL_616481,
EPI_ISL_616482, EPI_ISL_616483, EPI_ISL_616484, EPI_ISL_616485, EPI_ISL_616486, EPI_ISL_616487, EPI_ISL_616488, EPI_ISL_616489, EPI_ISL_616490, EPI_ISL_616491, EPI_ISL_616492, EPI_ISL_616493, EPI_ISL_616494, EPI_ISL_616495, EPI_ISL_616496, EPI_ISL_616497, EPI_ISL_616499, EPI_ISL_616500,
EPI_ISL_616502, EPI_ISL_616503, EPI_ISL_616504, EPI_ISL_616505, EPI_ISL_616506, EPI_ISL_616507, EPI_ISL_616508, EPI_ISL_616509, EPI_ISL_616510, EPI_ISL_616511, EPI_ISL_616512, EPI_ISL_616513, EPI_ISL_616514, EPI_ISL_616515, EPI_ISL_616516, EPI_ISL_616517, EPI_ISL_616518, EPI_ISL_616519,
EPI_ISL_616520, EPI_ISL_616521, EPI_ISL_616522, EPI_ISL_616523, EPI_ISL_616524, EPI_ISL_616525, EPI_ISL_616526, EPI_ISL_616527, EPI_ISL_616528, EPI_ISL_616529, EPI_ISL_616530, EPI_ISL_616531, EPI_ISL_616532, EPI_ISL_616534, EPI_ISL_616535, EPI_ISL_616536, EPI_ISL_616537, EPI_ISL_616538,
EPI_ISL_616540, EPI_ISL_616541, EPI_ISL_616542, EPI_ISL_616543, EPI_ISL_616545, EPI_ISL_616546, EPI_ISL_616547, EPI_ISL_616548, EPI_ISL_616549, EPI_ISL_616550, EPI_ISL_616551, EPI_ISL_616552, EPI_ISL_616553, EPI_ISL_616554, EPI_ISL_616555, EPI_ISL_616556, EPI_ISL_616557, EPI_ISL_616558,
EPI_ISL_616559, EPI_ISL_616560, EPI_ISL_616561, EPI_ISL_616562, EPI_ISL_616563, EPI_ISL_616564, EPI_ISL_616565, EPI_ISL_616566, EPI_ISL_616567, EPI_ISL_616568, EPI_ISL_616569, EPI_ISL_616570, EPI_ISL_616571, EPI_ISL_616572, EPI_ISL_616573, EPI_ISL_616574, EPI_ISL_616575, EPI_ISL_616576,
EPI_ISL_616577, EPI_ISL_616578, EPI_ISL_616579, EPI_ISL_616580, EPI_ISL_616581, EPI_ISL_616582, EPI_ISL_616585, EPI_ISL_616586, EPI_ISL_616587, EPI_ISL_616588, EPI_ISL_616589, EPI_ISL_616590, EPI_ISL_616591, EPI_ISL_616592, EPI_ISL_616593, EPI_ISL_616594, EPI_ISL_616595, EPI_ISL_616596,
EPI_ISL_616597, EPI_ISL_616598, EPI_ISL_616599, EPI_ISL_616600, EPI_ISL_616601, EPI_ISL_616602, EPI_ISL_616603, EPI_ISL_616604, EPI_ISL_616605, EPI_ISL_616606, EPI_ISL_616607, EPI_ISL_616608, EPI_ISL_616609, EPI_ISL_616610, EPI_ISL_616611, EPI_ISL_616612, EPI_ISL_616613, EPI_ISL_616615,
EPI_ISL_616616, EPI_ISL_616617, EPI_ISL_616618, EPI_ISL_616619, EPI_ISL_616620, EPI_ISL_616621, EPI_ISL_616622, EPI_ISL_616623, EPI_ISL_616624, EPI_ISL_616625, EPI_ISL_616626, EPI_ISL_616627, EPI_ISL_616628, EPI_ISL_616629, EPI_ISL_616630, EPI_ISL_616631, EPI_ISL_616632, EPI_ISL_616633,
EPI_ISL_616634, EPI_ISL_616635, EPI_ISL_616636, EPI_ISL_616638, EPI_ISL_616639, EPI_ISL_616640, EPI_ISL_616641, EPI_ISL_616642, EPI_ISL_616643, EPI_ISL_616644, EPI_ISL_616645, EPI_ISL_616646, EPI_ISL_616649, EPI_ISL_616650, EPI_ISL_616651, EPI_ISL_616652, EPI_ISL_616653, EPI_ISL_616654,
EPI_ISL_616655, EPI_ISL_616656, EPI_ISL_616657, EPI_ISL_616658, EPI_ISL_616659, EPI_ISL_616660, EPI_ISL_616661, EPI_ISL_616662, EPI_ISL_616663, EPI_ISL_616664, EPI_ISL_616665, EPI_ISL_616666, EPI_ISL_616667, EPI_ISL_616668, EPI_ISL_616669, EPI_ISL_616670, EPI_ISL_616671, EPI_ISL_616672,
EPI_ISL_616673, EPI_ISL_616674, EPI_ISL_616675, EPI_ISL_616676, EPI_ISL_616677, EPI_ISL_616678, EPI_ISL_616679, EPI_ISL_616680, EPI_ISL_616681, EPI_ISL_616682, EPI_ISL_616683, EPI_ISL_616684, EPI_ISL_616685, EPI_ISL_616686, EPI_ISL_616687, EPI_ISL_616689, EPI_ISL_616690, EPI_ISL_616691,
EPI_ISL_616692, EPI_ISL_616694, EPI_ISL_616696, EPI_ISL_616698, EPI_ISL_616699, EPI_ISL_616700, EPI_ISL_616701, EPI_ISL_616703, EPI_ISL_616704, EPI_ISL_616705, EPI_ISL_616706, EPI_ISL_616707, EPI_ISL_616708, EPI_ISL_616709, EPI_ISL_616710, EPI_ISL_616711, EPI_ISL_616712, EPI_ISL_616713,
EPI_ISL_616715, EPI_ISL_616716, EPI_ISL_616717, EPI_ISL_616718, EPI_ISL_616719, EPI_ISL_616720, EPI_ISL_616721, EPI_ISL_616722, EPI_ISL_616723, EPI_ISL_616725, EPI_ISL_616726, EPI_ISL_616728, EPI_ISL_616729, EPI_ISL_616731, EPI_ISL_616732, EPI_ISL_616733, EPI_ISL_616734, EPI_ISL_616735,
EPI_ISL_616736, EPI_ISL_616737, EPI_ISL_616738, EPI_ISL_616739, EPI_ISL_616740, EPI_ISL_616742, EPI_ISL_616743, EPI_ISL_616744, EPI_ISL_616747, EPI_ISL_616750, EPI_ISL_616752, EPI_ISL_616753, EPI_ISL_616754, EPI_ISL_616755, EPI_ISL_616756, EPI_ISL_616758, EPI_ISL_616759, EPI_ISL_616763,
EPI_ISL_616764, EPI_ISL_616765, EPI_ISL_616766, EPI_ISL_616767, EPI_ISL_616768, EPI_ISL_616769, EPI_ISL_616770, EPI_ISL_616772, EPI_ISL_616773, EPI_ISL_616774, EPI_ISL_616777, EPI_ISL_616779, EPI_ISL_616781, EPI_ISL_616783, EPI_ISL_616784, EPI_ISL_616785, EPI_ISL_616786, EPI_ISL_616787,
EPI_ISL_616789, EPI_ISL_616790, EPI_ISL_616791, EPI_ISL_616792, EPI_ISL_616793, EPI_ISL_616794, EPI_ISL_616795, EPI_ISL_616796, EPI_ISL_616797, EPI_ISL_616798, EPI_ISL_616799, EPI_ISL_616801, EPI_ISL_616803, EPI_ISL_616805, EPI_ISL_616806, EPI_ISL_616807, EPI_ISL_616809, EPI_ISL_616810,
EPI_ISL_616811, EPI_ISL_616812, EPI_ISL_616813, EPI_ISL_616814, EPI_ISL_616816, EPI_ISL_616817, EPI_ISL_616818, EPI_ISL_616820, EPI_ISL_616821, EPI_ISL_616822, EPI_ISL_616823, EPI_ISL_616824, EPI_ISL_616825, EPI_ISL_616827, EPI_ISL_616829, EPI_ISL_616830, EPI_ISL_616831, EPI_ISL_616832,
EPI_ISL_616833, EPI_ISL_616834, EPI_ISL_616835, EPI_ISL_616836, EPI_ISL_616837, EPI_ISL_616838, EPI_ISL_616839, EPI_ISL_616840, EPI_ISL_616841, EPI_ISL_616842, EPI_ISL_616843, EPI_ISL_616844, EPI_ISL_616845, EPI_ISL_616846, EPI_ISL_616847, EPI_ISL_616848, EPI_ISL_616849, EPI_ISL_616850,
EPI_ISL_616851, EPI_ISL_616852, EPI_ISL_616853, EPI_ISL_616854, EPI_ISL_616855, EPI_ISL_616856, EPI_ISL_616857, EPI_ISL_616858, EPI_ISL_616860, EPI_ISL_616861, EPI_ISL_616863, EPI_ISL_616865, EPI_ISL_616866, EPI_ISL_616867, EPI_ISL_616868, EPI_ISL_616869, EPI_ISL_616870, EPI_ISL_616871,
EPI_ISL_616872, EPI_ISL_616873, EPI_ISL_616875, EPI_ISL_616876, EPI_ISL_616877, EPI_ISL_616878, EPI_ISL_616879, EPI_ISL_616880, EPI_ISL_616881, EPI_ISL_616882, EPI_ISL_616883, EPI_ISL_616884, EPI_ISL_616885, EPI_ISL_616886, EPI_ISL_616887, EPI_ISL_616888, EPI_ISL_616889, EPI_ISL_616890,
EPI_ISL_616891, EPI_ISL_616892, EPI_ISL_616893, EPI_ISL_616894, EPI_ISL_616895, EPI_ISL_616896, EPI_ISL_616897, EPI_ISL_616898, EPI_ISL_616899, EPI_ISL_616900, EPI_ISL_616901, EPI_ISL_616902, EPI_ISL_616903, EPI_ISL_616905, EPI_ISL_616951, EPI_ISL_616952, EPI_ISL_616953, EPI_ISL_616954,
EPI_ISL_616955, EPI_ISL_616956, EPI_ISL_616976, EPI_ISL_616977, EPI_ISL_616978, EPI_ISL_616979, EPI_ISL_616980, EPI_ISL_616981, EPI_ISL_616982, EPI_ISL_616983, EPI_ISL_616984, EPI_ISL_616985, EPI_ISL_616986, EPI_ISL_616987, EPI_ISL_616988, EPI_ISL_616989, EPI_ISL_616990, EPI_ISL_616991,
EPI_ISL_616992, EPI_ISL_616993, EPI_ISL_616994, EPI_ISL_616995, EPI_ISL_616996, EPI_ISL_616997, EPI_ISL_616998, EPI_ISL_616999, EPI_ISL_617000, EPI_ISL_617001, EPI_ISL_617002, EPI_ISL_617003, EPI_ISL_617004, EPI_ISL_617005, EPI_ISL_617006, EPI_ISL_617007, EPI_ISL_617008, EPI_ISL_617009,
EPI_ISL_617010, EPI_ISL_617011, EPI_ISL_617012, EPI_ISL_617013, EPI_ISL_617014, EPI_ISL_617015, EPI_ISL_617016, EPI_ISL_617017, EPI_ISL_617018, EPI_ISL_617019, EPI_ISL_617020, EPI_ISL_617021, EPI_ISL_617022, EPI_ISL_617023, EPI_ISL_617024, EPI_ISL_617025, EPI_ISL_617026, EPI_ISL_617027,
EPI_ISL_617028, EPI_ISL_617029, EPI_ISL_617030, EPI_ISL_617031, EPI_ISL_617032, EPI_ISL_617033, EPI_ISL_617034, EPI_ISL_617035, EPI_ISL_617036, EPI_ISL_617037, EPI_ISL_617038, EPI_ISL_617039, EPI_ISL_617040, EPI_ISL_617041, EPI_ISL_617042, EPI_ISL_617043, EPI_ISL_617044, EPI_ISL_617045,
EPI_ISL_617046, EPI_ISL_617047, EPI_ISL_617048, EPI_ISL_617049, EPI_ISL_617050, EPI_ISL_617051, EPI_ISL_617052, EPI_ISL_617053, EPI_ISL_617054, EPI_ISL_617077, EPI_ISL_617078, EPI_ISL_617096, EPI_ISL_617097, EPI_ISL_617098, EPI_ISL_617099, EPI_ISL_617100, EPI_ISL_617101, EPI_ISL_617102,
EPI_ISL_617103, EPI_ISL_617104, EPI_ISL_617105, EPI_ISL_617106, EPI_ISL_617107, EPI_ISL_617108, EPI_ISL_617109, EPI_ISL_617110, EPI_ISL_617111, EPI_ISL_617112, EPI_ISL_617113, EPI_ISL_617114, EPI_ISL_617115, EPI_ISL_617116, EPI_ISL_617117, EPI_ISL_617118, EPI_ISL_617119, EPI_ISL_617120,
EPI_ISL_617121, EPI_ISL_617122, EPI_ISL_617123, EPI_ISL_617124, EPI_ISL_617125, EPI_ISL_617126, EPI_ISL_617127, EPI_ISL_617128, EPI_ISL_617129, EPI_ISL_617130, EPI_ISL_617131, EPI_ISL_617132, EPI_ISL_617133, EPI_ISL_617134, EPI_ISL_617135, EPI_ISL_617136, EPI_ISL_617137, EPI_ISL_617138,
EPI_ISL_617139, EPI_ISL_617140, EPI_ISL_617141, EPI_ISL_617142, EPI_ISL_617143, EPI_ISL_617144, EPI_ISL_617145, EPI_ISL_617146, EPI_ISL_617147, EPI_ISL_617148, EPI_ISL_617149, EPI_ISL_617150, EPI_ISL_617151, EPI_ISL_617152, EPI_ISL_617153, EPI_ISL_617154, EPI_ISL_617155, EPI_ISL_617156,
EPI_ISL_617157, EPI_ISL_617158, EPI_ISL_617159, EPI_ISL_617160, EPI_ISL_617161, EPI_ISL_617162, EPI_ISL_617163, EPI_ISL_617164, EPI_ISL_617165, EPI_ISL_617166, EPI_ISL_617167, EPI_ISL_617168, EPI_ISL_617169, EPI_ISL_617170, EPI_ISL_617171, EPI_ISL_617172, EPI_ISL_617173, EPI_ISL_617174,
EPI_ISL_617175, EPI_ISL_617176, EPI_ISL_617177, EPI_ISL_617178, EPI_ISL_617179, EPI_ISL_617180, EPI_ISL_617181, EPI_ISL_617182, EPI_ISL_617183, EPI_ISL_617184, EPI_ISL_617185, EPI_ISL_617186, EPI_ISL_617187, EPI_ISL_617188, EPI_ISL_617189, EPI_ISL_617190, EPI_ISL_617191, EPI_ISL_617192,
EPI_ISL_617193, EPI_ISL_617194, EPI_ISL_617195, EPI_ISL_617196, EPI_ISL_617197, EPI_ISL_617198, EPI_ISL_617199, EPI_ISL_617200, EPI_ISL_617201, EPI_ISL_617202, EPI_ISL_617203, EPI_ISL_617204, EPI_ISL_617205, EPI_ISL_617206, EPI_ISL_617207, EPI_ISL_617208, EPI_ISL_617209, EPI_ISL_617210,
EPI_ISL_617211, EPI_ISL_617212, EPI_ISL_617213, EPI_ISL_617214, EPI_ISL_617215, EPI_ISL_617216, EPI_ISL_617217, EPI_ISL_617218, EPI_ISL_617219, EPI_ISL_617220, EPI_ISL_617221, EPI_ISL_617222, EPI_ISL_617223, EPI_ISL_617224, EPI_ISL_617225, EPI_ISL_617226, EPI_ISL_617227, EPI_ISL_617228,
EPI_ISL_617229, EPI_ISL_617230, EPI_ISL_617231, EPI_ISL_617232, EPI_ISL_617233, EPI_ISL_617234, EPI_ISL_617235, EPI_ISL_617236, EPI_ISL_617237, EPI_ISL_617238, EPI_ISL_617239, EPI_ISL_617240, EPI_ISL_617241, EPI_ISL_617242, EPI_ISL_617243, EPI_ISL_617244, EPI_ISL_617245, EPI_ISL_617246,
EPI_ISL_617247, EPI_ISL_617248, EPI_ISL_617249, EPI_ISL_617250, EPI_ISL_617251, EPI_ISL_617252, EPI_ISL_617253, EPI_ISL_617254, EPI_ISL_617255, EPI_ISL_617256, EPI_ISL_617257, EPI_ISL_617258, EPI_ISL_617259, EPI_ISL_617260, EPI_ISL_617261, EPI_ISL_617262, EPI_ISL_617263, EPI_ISL_617264,
EPI_ISL_617265, EPI_ISL_617266, EPI_ISL_617267, EPI_ISL_617268, EPI_ISL_617269, EPI_ISL_617270, EPI_ISL_617271, EPI_ISL_617272, EPI_ISL_617273, EPI_ISL_617274, EPI_ISL_617275, EPI_ISL_617276, EPI_ISL_617277, EPI_ISL_617278, EPI_ISL_617279, EPI_ISL_617280, EPI_ISL_617281, EPI_ISL_617282,
EPI_ISL_617283, EPI_ISL_617284, EPI_ISL_617285, EPI_ISL_617286, EPI_ISL_617287, EPI_ISL_617288, EPI_ISL_617289, EPI_ISL_617290, EPI_ISL_617291, EPI_ISL_617292, EPI_ISL_617293, EPI_ISL_617294, EPI_ISL_617295, EPI_ISL_617296, EPI_ISL_617297, EPI_ISL_617298, EPI_ISL_617299, EPI_ISL_617300,
EPI_ISL_617301, EPI_ISL_617302, EPI_ISL_617303, EPI_ISL_617304, EPI_ISL_617305, EPI_ISL_617306, EPI_ISL_617307, EPI_ISL_617308, EPI_ISL_617309, EPI_ISL_617310, EPI_ISL_617311, EPI_ISL_617312, EPI_ISL_617313, EPI_ISL_617314, EPI_ISL_617315, EPI_ISL_617316, EPI_ISL_617317, EPI_ISL_617318,
EPI_ISL_617319, EPI_ISL_617320, EPI_ISL_617321, EPI_ISL_617322, EPI_ISL_617323, EPI_ISL_617324, EPI_ISL_617325, EPI_ISL_617326, EPI_ISL_617327, EPI_ISL_617328, EPI_ISL_617329, EPI_ISL_617330, EPI_ISL_617331, EPI_ISL_617332, EPI_ISL_617333, EPI_ISL_617334, EPI_ISL_617335, EPI_ISL_617336,
EPI_ISL_617337, EPI_ISL_617338, EPI_ISL_617339, EPI_ISL_617340, EPI_ISL_617341, EPI_ISL_617342, EPI_ISL_617343, EPI_ISL_617344, EPI_ISL_617345, EPI_ISL_617346, EPI_ISL_617347, EPI_ISL_617348, EPI_ISL_617349, EPI_ISL_617350, EPI_ISL_617351, EPI_ISL_617352, EPI_ISL_617353, EPI_ISL_617354,
EPI_ISL_617355, EPI_ISL_617356, EPI_ISL_617357, EPI_ISL_617358, EPI_ISL_617359, EPI_ISL_617360, EPI_ISL_617361, EPI_ISL_617362, EPI_ISL_617363, EPI_ISL_617364, EPI_ISL_617365, EPI_ISL_617366, EPI_ISL_617367, EPI_ISL_617368, EPI_ISL_617369, EPI_ISL_617370, EPI_ISL_617371, EPI_ISL_617372,
EPI_ISL_617373, EPI_ISL_617374, EPI_ISL_617375, EPI_ISL_617376, EPI_ISL_617377, EPI_ISL_617378, EPI_ISL_617379, EPI_ISL_617380, EPI_ISL_617381, EPI_ISL_617382, EPI_ISL_617383, EPI_ISL_617384, EPI_ISL_617385, EPI_ISL_617386, EPI_ISL_617387, EPI_ISL_617388, EPI_ISL_617389, EPI_ISL_617390,
EPI_ISL_617391, EPI_ISL_617392, EPI_ISL_617393, EPI_ISL_617394, EPI_ISL_617395, EPI_ISL_617396, EPI_ISL_617397, EPI_ISL_617398, EPI_ISL_617399, EPI_ISL_617400, EPI_ISL_617401, EPI_ISL_617402, EPI_ISL_617403, EPI_ISL_617404, EPI_ISL_617405, EPI_ISL_617406, EPI_ISL_617407, EPI_ISL_617408,
EPI_ISL_617409, EPI_ISL_617410, EPI_ISL_617411, EPI_ISL_617412, EPI_ISL_617413, EPI_ISL_617414, EPI_ISL_617416, EPI_ISL_617417, EPI_ISL_617419, EPI_ISL_617420, EPI_ISL_617422, EPI_ISL_617424, EPI_ISL_617425, EPI_ISL_617426, EPI_ISL_617427, EPI_ISL_617428, EPI_ISL_617429, EPI_ISL_617430,
EPI_ISL_617431, EPI_ISL_617432, EPI_ISL_617433, EPI_ISL_617434, EPI_ISL_617435, EPI_ISL_617436, EPI_ISL_617437, EPI_ISL_617438, EPI_ISL_617439, EPI_ISL_617440, EPI_ISL_617441, EPI_ISL_617443, EPI_ISL_617446, EPI_ISL_617447, EPI_ISL_617448, EPI_ISL_617449, EPI_ISL_617450, EPI_ISL_617451,
EPI_ISL_617452, EPI_ISL_617453, EPI_ISL_617454, EPI_ISL_617455, EPI_ISL_617456, EPI_ISL_617458, EPI_ISL_617459, EPI_ISL_617460, EPI_ISL_617461, EPI_ISL_617462, EPI_ISL_617463, EPI_ISL_617464, EPI_ISL_617465, EPI_ISL_617467, EPI_ISL_617469, EPI_ISL_617470, EPI_ISL_617471, EPI_ISL_617472,
EPI_ISL_617473, EPI_ISL_617475, EPI_ISL_617476, EPI_ISL_617477, EPI_ISL_617478, EPI_ISL_617479, EPI_ISL_617480, EPI_ISL_617481, EPI_ISL_617482, EPI_ISL_617483, EPI_ISL_617484, EPI_ISL_617485, EPI_ISL_617486, EPI_ISL_617487, EPI_ISL_617488, EPI_ISL_617489, EPI_ISL_617490, EPI_ISL_617492,
EPI_ISL_617493, EPI_ISL_617494, EPI_ISL_617495, EPI_ISL_617496, EPI_ISL_617498, EPI_ISL_617499, EPI_ISL_617500, EPI_ISL_617501, EPI_ISL_617502, EPI_ISL_617503, EPI_ISL_617505, EPI_ISL_617506, EPI_ISL_617507, EPI_ISL_617509, EPI_ISL_617510, EPI_ISL_617511, EPI_ISL_617512, EPI_ISL_617513,
EPI_ISL_617514, EPI_ISL_617515, EPI_ISL_617516, EPI_ISL_617517, EPI_ISL_617518, EPI_ISL_617519, EPI_ISL_617520, EPI_ISL_617521, EPI_ISL_617522, EPI_ISL_617524, EPI_ISL_617525, EPI_ISL_617526, EPI_ISL_617527, EPI_ISL_617528, EPI_ISL_617530, EPI_ISL_617531, EPI_ISL_617532, EPI_ISL_617533,
EPI_ISL_617534, EPI_ISL_617535, EPI_ISL_617537, EPI_ISL_617538, EPI_ISL_617539, EPI_ISL_617541, EPI_ISL_617542, EPI_ISL_617543, EPI_ISL_617544, EPI_ISL_617545, EPI_ISL_617546, EPI_ISL_617547, EPI_ISL_617548, EPI_ISL_617549, EPI_ISL_617550, EPI_ISL_617551, EPI_ISL_617552, EPI_ISL_617553,
EPI_ISL_617554, EPI_ISL_617555, EPI_ISL_617556, EPI_ISL_617557, EPI_ISL_617558, EPI_ISL_617559, EPI_ISL_617560, EPI_ISL_617561, EPI_ISL_617562, EPI_ISL_617563, EPI_ISL_617565, EPI_ISL_617566, EPI_ISL_617567, EPI_ISL_617568, EPI_ISL_617569, EPI_ISL_617570, EPI_ISL_617571, EPI_ISL_617572,
EPI_ISL_617574, EPI_ISL_617575, EPI_ISL_617577, EPI_ISL_617579, EPI_ISL_617581, EPI_ISL_617582, EPI_ISL_617583, EPI_ISL_617584, EPI_ISL_617585, EPI_ISL_617586, EPI_ISL_617587, EPI_ISL_617588, EPI_ISL_617589, EPI_ISL_617590, EPI_ISL_617591, EPI_ISL_617592, EPI_ISL_617593, EPI_ISL_617594,
EPI_ISL_617595, EPI_ISL_617596, EPI_ISL_617597, EPI_ISL_617598, EPI_ISL_617600, EPI_ISL_617601, EPI_ISL_617602, EPI_ISL_617603, EPI_ISL_617604, EPI_ISL_617605, EPI_ISL_617606, EPI_ISL_617607, EPI_ISL_617608, EPI_ISL_617609, EPI_ISL_617610, EPI_ISL_617611, EPI_ISL_617612, EPI_ISL_617613,
EPI_ISL_617614, EPI_ISL_617615, EPI_ISL_617616, EPI_ISL_617617, EPI_ISL_617619, EPI_ISL_617620, EPI_ISL_617621, EPI_ISL_617622, EPI_ISL_617623, EPI_ISL_617624, EPI_ISL_617625, EPI_ISL_617626, EPI_ISL_617627, EPI_ISL_617628, EPI_ISL_617629, EPI_ISL_617630, EPI_ISL_617631, EPI_ISL_617632,
EPI_ISL_617633, EPI_ISL_617634, EPI_ISL_617635, EPI_ISL_617636, EPI_ISL_617637, EPI_ISL_617639, EPI_ISL_617640, EPI_ISL_617641, EPI_ISL_617642, EPI_ISL_617644, EPI_ISL_617645, EPI_ISL_617646, EPI_ISL_617647, EPI_ISL_617648, EPI_ISL_617649, EPI_ISL_617650, EPI_ISL_617651, EPI_ISL_617653,
EPI_ISL_617654, EPI_ISL_617656, EPI_ISL_617657, EPI_ISL_617659, EPI_ISL_617661, EPI_ISL_617662, EPI_ISL_617663, EPI_ISL_617664, EPI_ISL_617665, EPI_ISL_617666, EPI_ISL_617667, EPI_ISL_617668, EPI_ISL_617669, EPI_ISL_617670, EPI_ISL_617671, EPI_ISL_617672, EPI_ISL_617673, EPI_ISL_617674,
EPI_ISL_617675, EPI_ISL_617676, EPI_ISL_617677, EPI_ISL_617678, EPI_ISL_617679, EPI_ISL_617680, EPI_ISL_617681, EPI_ISL_617682, EPI_ISL_617683, EPI_ISL_617684, EPI_ISL_617685, EPI_ISL_617686, EPI_ISL_617687, EPI_ISL_617688, EPI_ISL_617689, EPI_ISL_617690, EPI_ISL_617691, EPI_ISL_617692,
EPI_ISL_617693, EPI_ISL_617694, EPI_ISL_617695, EPI_ISL_617696, EPI_ISL_617698, EPI_ISL_617699, EPI_ISL_617700, EPI_ISL_617701, EPI_ISL_617702, EPI_ISL_617703, EPI_ISL_617704, EPI_ISL_617705, EPI_ISL_617706, EPI_ISL_617707, EPI_ISL_617708, EPI_ISL_617709, EPI_ISL_617710, EPI_ISL_617711,
EPI_ISL_617712, EPI_ISL_617713, EPI_ISL_617714, EPI_ISL_617715, EPI_ISL_617716, EPI_ISL_617717, EPI_ISL_617718, EPI_ISL_617719, EPI_ISL_617720, EPI_ISL_617722, EPI_ISL_617723, EPI_ISL_617724, EPI_ISL_617725, EPI_ISL_617726, EPI_ISL_617727, EPI_ISL_617728, EPI_ISL_617729, EPI_ISL_617730,
EPI_ISL_617731, EPI_ISL_617733, EPI_ISL_617734, EPI_ISL_617735, EPI_ISL_617736, EPI_ISL_617737, EPI_ISL_617738, EPI_ISL_617739, EPI_ISL_617740, EPI_ISL_617741, EPI_ISL_617742, EPI_ISL_617743, EPI_ISL_617744, EPI_ISL_617745, EPI_ISL_617746, EPI_ISL_617747, EPI_ISL_617748, EPI_ISL_617749,
EPI_ISL_617750, EPI_ISL_617752, EPI_ISL_617753, EPI_ISL_617754, EPI_ISL_617755, EPI_ISL_617756, EPI_ISL_617757, EPI_ISL_617758, EPI_ISL_617759, EPI_ISL_617760, EPI_ISL_617761, EPI_ISL_617762, EPI_ISL_617763, EPI_ISL_617764, EPI_ISL_617765, EPI_ISL_617766, EPI_ISL_617767, EPI_ISL_617768,
EPI_ISL_617769, EPI_ISL_617770, EPI_ISL_617771, EPI_ISL_617772, EPI_ISL_617773, EPI_ISL_617774, EPI_ISL_617775, EPI_ISL_617776, EPI_ISL_617777, EPI_ISL_617778, EPI_ISL_617779, EPI_ISL_617780, EPI_ISL_617781, EPI_ISL_617782, EPI_ISL_617783, EPI_ISL_617784, EPI_ISL_617785, EPI_ISL_617786,
EPI_ISL_617787, EPI_ISL_617788, EPI_ISL_617789, EPI_ISL_617791, EPI_ISL_617792, EPI_ISL_617793, EPI_ISL_617794, EPI_ISL_617795, EPI_ISL_617797, EPI_ISL_617798, EPI_ISL_617799, EPI_ISL_617800, EPI_ISL_617801, EPI_ISL_617802, EPI_ISL_617803, EPI_ISL_617804, EPI_ISL_617805, EPI_ISL_617806,
EPI_ISL_617807, EPI_ISL_617808, EPI_ISL_617809, EPI_ISL_617810, EPI_ISL_617811, EPI_ISL_617812, EPI_ISL_617813, EPI_ISL_617814, EPI_ISL_617815, EPI_ISL_617816, EPI_ISL_617817, EPI_ISL_617818, EPI_ISL_617819, EPI_ISL_617820, EPI_ISL_617821, EPI_ISL_617822, EPI_ISL_617823, EPI_ISL_617824,
EPI_ISL_617825, EPI_ISL_617826, EPI_ISL_617827, EPI_ISL_617828, EPI_ISL_617829, EPI_ISL_617830, EPI_ISL_617831, EPI_ISL_617832, EPI_ISL_617833, EPI_ISL_617834, EPI_ISL_617835, EPI_ISL_617836, EPI_ISL_617837, EPI_ISL_617838, EPI_ISL_617839, EPI_ISL_617840, EPI_ISL_617841, EPI_ISL_617842,
EPI_ISL_617843, EPI_ISL_617844, EPI_ISL_617845, EPI_ISL_617846, EPI_ISL_617847, EPI_ISL_617848, EPI_ISL_617849, EPI_ISL_617851, EPI_ISL_617852, EPI_ISL_617853, EPI_ISL_617855, EPI_ISL_617856, EPI_ISL_617857, EPI_ISL_617858, EPI_ISL_617859, EPI_ISL_617860, EPI_ISL_617861, EPI_ISL_617862,
EPI_ISL_617863, EPI_ISL_617864, EPI_ISL_617865, EPI_ISL_617866, EPI_ISL_617868, EPI_ISL_617869, EPI_ISL_617871, EPI_ISL_617872, EPI_ISL_617873, EPI_ISL_617874, EPI_ISL_617875, EPI_ISL_617876, EPI_ISL_617877, EPI_ISL_617879, EPI_ISL_617881, EPI_ISL_617882, EPI_ISL_617883, EPI_ISL_617884,
EPI_ISL_617885, EPI_ISL_617886, EPI_ISL_617887, EPI_ISL_617888, EPI_ISL_617889, EPI_ISL_617890, EPI_ISL_617891, EPI_ISL_617892, EPI_ISL_617893, EPI_ISL_617894, EPI_ISL_617895, EPI_ISL_617896, EPI_ISL_617897, EPI_ISL_617898, EPI_ISL_617899, EPI_ISL_617900, EPI_ISL_617901, EPI_ISL_617902,
EPI_ISL_617903, EPI_ISL_617904, EPI_ISL_617905, EPI_ISL_617906, EPI_ISL_617908, EPI_ISL_617909, EPI_ISL_617910, EPI_ISL_617911, EPI_ISL_617912, EPI_ISL_617913, EPI_ISL_617914, EPI_ISL_617915, EPI_ISL_617916, EPI_ISL_617917, EPI_ISL_617920, EPI_ISL_617921, EPI_ISL_617922, EPI_ISL_617923,
EPI_ISL_617926, EPI_ISL_617927, EPI_ISL_617928, EPI_ISL_617929, EPI_ISL_617930, EPI_ISL_617931, EPI_ISL_617933, EPI_ISL_617935, EPI_ISL_617936, EPI_ISL_617937, EPI_ISL_617938, EPI_ISL_617939, EPI_ISL_617944, EPI_ISL_617945, EPI_ISL_617946, EPI_ISL_617947, EPI_ISL_617948, EPI_ISL_617949,
EPI_ISL_617950, EPI_ISL_617951, EPI_ISL_617952, EPI_ISL_617954, EPI_ISL_617955, EPI_ISL_617956, EPI_ISL_617957, EPI_ISL_617958, EPI_ISL_617962, EPI_ISL_617963, EPI_ISL_617964, EPI_ISL_617966, EPI_ISL_617967, EPI_ISL_617968, EPI_ISL_617970, EPI_ISL_617971, EPI_ISL_617972, EPI_ISL_617973,
EPI_ISL_617974, EPI_ISL_617976, EPI_ISL_617977, EPI_ISL_617979, EPI_ISL_617981, EPI_ISL_617982, EPI_ISL_617983, EPI_ISL_617984, EPI_ISL_617985, EPI_ISL_617986, EPI_ISL_617987, EPI_ISL_617989, EPI_ISL_617990, EPI_ISL_617991, EPI_ISL_617992, EPI_ISL_617993, EPI_ISL_617994, EPI_ISL_617995,
EPI_ISL_617996, EPI_ISL_617997, EPI_ISL_617998, EPI_ISL_617999, EPI_ISL_618000, EPI_ISL_618001, EPI_ISL_618002, EPI_ISL_618003, EPI_ISL_618004, EPI_ISL_618005, EPI_ISL_618006, EPI_ISL_618008, EPI_ISL_618009, EPI_ISL_618010, EPI_ISL_618011, EPI_ISL_618012, EPI_ISL_618014, EPI_ISL_618015,
EPI_ISL_618016, EPI_ISL_618017, EPI_ISL_618019, EPI_ISL_618020, EPI_ISL_618021, EPI_ISL_618022, EPI_ISL_618023, EPI_ISL_618024, EPI_ISL_618025, EPI_ISL_618026, EPI_ISL_618027, EPI_ISL_618028, EPI_ISL_618029, EPI_ISL_618030, EPI_ISL_618031, EPI_ISL_618032, EPI_ISL_618036, EPI_ISL_618037,
EPI_ISL_618039, EPI_ISL_618040, EPI_ISL_618041, EPI_ISL_618043, EPI_ISL_618045, EPI_ISL_618046, EPI_ISL_618047, EPI_ISL_618048, EPI_ISL_618049, EPI_ISL_618050, EPI_ISL_618051, EPI_ISL_618052, EPI_ISL_618053, EPI_ISL_618054, EPI_ISL_618055, EPI_ISL_618056, EPI_ISL_618057, EPI_ISL_618058,
EPI_ISL_618059, EPI_ISL_618062, EPI_ISL_618063, EPI_ISL_618064, EPI_ISL_618065, EPI_ISL_618066, EPI_ISL_618067, EPI_ISL_618068, EPI_ISL_618069, EPI_ISL_618070, EPI_ISL_618071, EPI_ISL_618072, EPI_ISL_618073, EPI_ISL_618074, EPI_ISL_618076, EPI_ISL_618077, EPI_ISL_618078, EPI_ISL_618079,

EPI_ISL_618080, EPI_ISL_618081, EPI_ISL_618082, EPI_ISL_618083, EPI_ISL_618084, EPI_ISL_618085, EPI_ISL_618086, EPI_ISL_618087, EPI_ISL_618088, EPI_ISL_618089, EPI_ISL_618090, EPI_ISL_618091, EPI_ISL_618092, EPI_ISL_618093, EPI_ISL_618094, EPI_ISL_618095, EPI_ISL_618096, EPI_ISL_618097,
EPI_ISL_618098, EPI_ISL_618099, EPI_ISL_618100, EPI_ISL_618101, EPI_ISL_618102, EPI_ISL_618103, EPI_ISL_618104, EPI_ISL_618105, EPI_ISL_618108, EPI_ISL_618109, EPI_ISL_618110, EPI_ISL_618111, EPI_ISL_618112, EPI_ISL_618113, EPI_ISL_618114, EPI_ISL_618115, EPI_ISL_618116, EPI_ISL_618117,
EPI_ISL_618118, EPI_ISL_618120, EPI_ISL_618121, EPI_ISL_618122, EPI_ISL_618124, EPI_ISL_618125, EPI_ISL_618128, EPI_ISL_618129, EPI_ISL_618130, EPI_ISL_618132, EPI_ISL_618133, EPI_ISL_618134, EPI_ISL_618135, EPI_ISL_618136, EPI_ISL_618137, EPI_ISL_618139, EPI_ISL_618144, EPI_ISL_618145,
EPI_ISL_618146, EPI_ISL_618147, EPI_ISL_618148, EPI_ISL_618149, EPI_ISL_618150, EPI_ISL_618154, EPI_ISL_618155, EPI_ISL_618156, EPI_ISL_618157, EPI_ISL_618158, EPI_ISL_618159, EPI_ISL_618160, EPI_ISL_618161, EPI_ISL_618162, EPI_ISL_618163, EPI_ISL_618164, EPI_ISL_618165, EPI_ISL_618166,
EPI_ISL_618167, EPI_ISL_618169, EPI_ISL_618170, EPI_ISL_618171, EPI_ISL_618173, EPI_ISL_618174, EPI_ISL_618175, EPI_ISL_618176, EPI_ISL_618177, EPI_ISL_618178, EPI_ISL_618179, EPI_ISL_618180, EPI_ISL_618181, EPI_ISL_618182, EPI_ISL_618183, EPI_ISL_618184, EPI_ISL_618185, EPI_ISL_618186,
EPI_ISL_618187, EPI_ISL_618188, EPI_ISL_618189, EPI_ISL_618190, EPI_ISL_618191, EPI_ISL_618192, EPI_ISL_618194, EPI_ISL_618195, EPI_ISL_618196, EPI_ISL_618197, EPI_ISL_618198, EPI_ISL_618199, EPI_ISL_618200, EPI_ISL_618201, EPI_ISL_618202, EPI_ISL_618203, EPI_ISL_618204, EPI_ISL_618205,
EPI_ISL_618206, EPI_ISL_618207, EPI_ISL_618208, EPI_ISL_618209, EPI_ISL_618210, EPI_ISL_618211, EPI_ISL_618212, EPI_ISL_618213, EPI_ISL_618214, EPI_ISL_618215, EPI_ISL_618217, EPI_ISL_618218, EPI_ISL_618219, EPI_ISL_618220, EPI_ISL_618221, EPI_ISL_618222, EPI_ISL_618224, EPI_ISL_618225,
EPI_ISL_618226, EPI_ISL_618227, EPI_ISL_618228, EPI_ISL_618229, EPI_ISL_618230, EPI_ISL_618231, EPI_ISL_618232, EPI_ISL_618233, EPI_ISL_618234, EPI_ISL_618235, EPI_ISL_618236, EPI_ISL_618237, EPI_ISL_618238, EPI_ISL_618239, EPI_ISL_618240, EPI_ISL_618241, EPI_ISL_618242, EPI_ISL_618243,
EPI_ISL_618244, EPI_ISL_618246, EPI_ISL_618247, EPI_ISL_618248, EPI_ISL_618249, EPI_ISL_618250, EPI_ISL_618251, EPI_ISL_618252, EPI_ISL_618253, EPI_ISL_618254, EPI_ISL_618255, EPI_ISL_618256, EPI_ISL_618257, EPI_ISL_618258, EPI_ISL_618259, EPI_ISL_618260, EPI_ISL_618261, EPI_ISL_618262,
EPI_ISL_618263, EPI_ISL_618264, EPI_ISL_618266, EPI_ISL_618267, EPI_ISL_618268, EPI_ISL_618270, EPI_ISL_618271, EPI_ISL_618272, EPI_ISL_618273, EPI_ISL_618275, EPI_ISL_618276, EPI_ISL_618277, EPI_ISL_618278, EPI_ISL_618279, EPI_ISL_618280, EPI_ISL_618281, EPI_ISL_618282, EPI_ISL_618283,
EPI_ISL_618284, EPI_ISL_618285, EPI_ISL_618286, EPI_ISL_618287, EPI_ISL_618288, EPI_ISL_618289, EPI_ISL_618290, EPI_ISL_618291, EPI_ISL_618292, EPI_ISL_618294, EPI_ISL_618295, EPI_ISL_618298, EPI_ISL_618299, EPI_ISL_618300, EPI_ISL_618301, EPI_ISL_618302, EPI_ISL_618303, EPI_ISL_618304,
EPI_ISL_618305, EPI_ISL_618306, EPI_ISL_618307, EPI_ISL_618308, EPI_ISL_618309, EPI_ISL_618310, EPI_ISL_618311, EPI_ISL_618312, EPI_ISL_618313, EPI_ISL_618314, EPI_ISL_618315, EPI_ISL_618316, EPI_ISL_618317, EPI_ISL_618318, EPI_ISL_618319, EPI_ISL_618320, EPI_ISL_618321, EPI_ISL_618322,
EPI_ISL_618323, EPI_ISL_618324, EPI_ISL_618325, EPI_ISL_618326, EPI_ISL_618327, EPI_ISL_618328, EPI_ISL_618329, EPI_ISL_618331, EPI_ISL_618333, EPI_ISL_618334, EPI_ISL_618335, EPI_ISL_618336, EPI_ISL_618337, EPI_ISL_618338, EPI_ISL_618339, EPI_ISL_618340, EPI_ISL_618341, EPI_ISL_618342,
EPI_ISL_618343, EPI_ISL_618344, EPI_ISL_618345, EPI_ISL_618346, EPI_ISL_618347, EPI_ISL_618348, EPI_ISL_618350, EPI_ISL_618351, EPI_ISL_618352, EPI_ISL_618353, EPI_ISL_618354, EPI_ISL_618355, EPI_ISL_618356, EPI_ISL_618357, EPI_ISL_618358, EPI_ISL_618359, EPI_ISL_618360, EPI_ISL_618361,
EPI_ISL_618363, EPI_ISL_618364, EPI_ISL_618365, EPI_ISL_618366, EPI_ISL_618367, EPI_ISL_618368, EPI_ISL_618369, EPI_ISL_618370, EPI_ISL_618371, EPI_ISL_618372, EPI_ISL_618374, EPI_ISL_618375, EPI_ISL_618376, EPI_ISL_618377, EPI_ISL_618378, EPI_ISL_618379, EPI_ISL_618380, EPI_ISL_618381,
EPI_ISL_618382, EPI_ISL_618383, EPI_ISL_618384, EPI_ISL_618385, EPI_ISL_618386, EPI_ISL_618387, EPI_ISL_618389, EPI_ISL_618390, EPI_ISL_618391, EPI_ISL_618392, EPI_ISL_618393, EPI_ISL_618394, EPI_ISL_618395, EPI_ISL_618396, EPI_ISL_618397, EPI_ISL_618398, EPI_ISL_618399, EPI_ISL_618400,
EPI_ISL_618402, EPI_ISL_618403, EPI_ISL_618404, EPI_ISL_618405, EPI_ISL_618406, EPI_ISL_618407, EPI_ISL_618408, EPI_ISL_618410, EPI_ISL_618411, EPI_ISL_618412, EPI_ISL_618414, EPI_ISL_618415, EPI_ISL_618416, EPI_ISL_618417, EPI_ISL_618418, EPI_ISL_618419, EPI_ISL_618421, EPI_ISL_618422,
EPI_ISL_618424, EPI_ISL_618425, EPI_ISL_618427, EPI_ISL_618428, EPI_ISL_618429, EPI_ISL_618430, EPI_ISL_618431, EPI_ISL_618433, EPI_ISL_618434, EPI_ISL_618435, EPI_ISL_618436, EPI_ISL_618437, EPI_ISL_618438, EPI_ISL_618439, EPI_ISL_618440, EPI_ISL_618442, EPI_ISL_618443, EPI_ISL_618444,
EPI_ISL_618446, EPI_ISL_618447, EPI_ISL_618448, EPI_ISL_618449, EPI_ISL_618450, EPI_ISL_618451, EPI_ISL_618452, EPI_ISL_618453, EPI_ISL_618454, EPI_ISL_618455, EPI_ISL_618456, EPI_ISL_618457, EPI_ISL_618458, EPI_ISL_618459, EPI_ISL_618460, EPI_ISL_618461, EPI_ISL_618462, EPI_ISL_618463,
EPI_ISL_618465, EPI_ISL_618467, EPI_ISL_618468, EPI_ISL_618470, EPI_ISL_618471, EPI_ISL_618475, EPI_ISL_618476, EPI_ISL_618477, EPI_ISL_618478, EPI_ISL_618479, EPI_ISL_618480, EPI_ISL_618481, EPI_ISL_618482, EPI_ISL_618485, EPI_ISL_618487, EPI_ISL_618489, EPI_ISL_618490, EPI_ISL_618491,
EPI_ISL_618492, EPI_ISL_618493, EPI_ISL_618495, EPI_ISL_618496, EPI_ISL_618497, EPI_ISL_618498, EPI_ISL_618499, EPI_ISL_618500, EPI_ISL_618501, EPI_ISL_618502, EPI_ISL_618503, EPI_ISL_618505, EPI_ISL_618507, EPI_ISL_618508, EPI_ISL_618510, EPI_ISL_618511, EPI_ISL_618513, EPI_ISL_618514,
EPI_ISL_618515, EPI_ISL_618516, EPI_ISL_618517, EPI_ISL_618518, EPI_ISL_618520, EPI_ISL_618522, EPI_ISL_618524, EPI_ISL_618525, EPI_ISL_618526, EPI_ISL_618527, EPI_ISL_618528, EPI_ISL_618530, EPI_ISL_618531, EPI_ISL_618532, EPI_ISL_618534, EPI_ISL_618536, EPI_ISL_618537, EPI_ISL_618538,
EPI_ISL_618539, EPI_ISL_618540, EPI_ISL_618544, EPI_ISL_618545, EPI_ISL_618547, EPI_ISL_618548, EPI_ISL_618549, EPI_ISL_618550, EPI_ISL_618552, EPI_ISL_618553, EPI_ISL_618555, EPI_ISL_618556, EPI_ISL_618557, EPI_ISL_618558, EPI_ISL_618559, EPI_ISL_618560, EPI_ISL_618561, EPI_ISL_618562,
EPI_ISL_618563, EPI_ISL_618564, EPI_ISL_618565, EPI_ISL_618566, EPI_ISL_618567, EPI_ISL_618568, EPI_ISL_618569, EPI_ISL_618570, EPI_ISL_618573, EPI_ISL_618574, EPI_ISL_618575, EPI_ISL_618576, EPI_ISL_618577, EPI_ISL_618578, EPI_ISL_618579, EPI_ISL_618580, EPI_ISL_618581, EPI_ISL_618582,
EPI_ISL_618583, EPI_ISL_618584, EPI_ISL_618585, EPI_ISL_618586, EPI_ISL_618587, EPI_ISL_618590, EPI_ISL_618591, EPI_ISL_618592, EPI_ISL_618593, EPI_ISL_618594, EPI_ISL_618595, EPI_ISL_618596, EPI_ISL_618597, EPI_ISL_618598, EPI_ISL_618600, EPI_ISL_618601, EPI_ISL_618602, EPI_ISL_618603,
EPI_ISL_618604, EPI_ISL_618605, EPI_ISL_618606, EPI_ISL_618607, EPI_ISL_618608, EPI_ISL_618609, EPI_ISL_618610, EPI_ISL_618611, EPI_ISL_618612, EPI_ISL_618613, EPI_ISL_618614, EPI_ISL_618615, EPI_ISL_618616, EPI_ISL_618617, EPI_ISL_618619, EPI_ISL_618620, EPI_ISL_618622, EPI_ISL_618625,
EPI_ISL_618626, EPI_ISL_618627, EPI_ISL_618628, EPI_ISL_618629, EPI_ISL_618630, EPI_ISL_618631, EPI_ISL_618633, EPI_ISL_618634, EPI_ISL_618635, EPI_ISL_618637, EPI_ISL_618638, EPI_ISL_618639, EPI_ISL_618640, EPI_ISL_618641, EPI_ISL_618642, EPI_ISL_618643, EPI_ISL_618644, EPI_ISL_618645,
EPI_ISL_618647, EPI_ISL_618648, EPI_ISL_618649, EPI_ISL_618650, EPI_ISL_618651, EPI_ISL_618652, EPI_ISL_618653, EPI_ISL_618654, EPI_ISL_618655, EPI_ISL_618656, EPI_ISL_618657, EPI_ISL_618659, EPI_ISL_618661, EPI_ISL_618662, EPI_ISL_618663, EPI_ISL_618664, EPI_ISL_618665, EPI_ISL_618666,
EPI_ISL_618668, EPI_ISL_618669, EPI_ISL_618671, EPI_ISL_618673, EPI_ISL_618675, EPI_ISL_618676, EPI_ISL_618677, EPI_ISL_618678, EPI_ISL_618679, EPI_ISL_618680, EPI_ISL_618682, EPI_ISL_618683, EPI_ISL_618684, EPI_ISL_618685, EPI_ISL_618686, EPI_ISL_618688, EPI_ISL_618689, EPI_ISL_618690,
EPI_ISL_618691, EPI_ISL_618692, EPI_ISL_618693, EPI_ISL_618694, EPI_ISL_618696, EPI_ISL_618698, EPI_ISL_618701, EPI_ISL_618703, EPI_ISL_618704, EPI_ISL_618705, EPI_ISL_618706, EPI_ISL_618707, EPI_ISL_618709, EPI_ISL_618710, EPI_ISL_618711, EPI_ISL_618712, EPI_ISL_618714, EPI_ISL_618715,
EPI_ISL_618716, EPI_ISL_618717, EPI_ISL_618718, EPI_ISL_618719, EPI_ISL_618720, EPI_ISL_618722, EPI_ISL_618725, EPI_ISL_618726, EPI_ISL_618728, EPI_ISL_618729, EPI_ISL_618730, EPI_ISL_618731, EPI_ISL_618732, EPI_ISL_618733, EPI_ISL_618734, EPI_ISL_618735, EPI_ISL_618736, EPI_ISL_618737,
EPI_ISL_618738, EPI_ISL_618739, EPI_ISL_618740, EPI_ISL_618741, EPI_ISL_618742, EPI_ISL_618744, EPI_ISL_618745, EPI_ISL_618746, EPI_ISL_618747, EPI_ISL_618748, EPI_ISL_618749, EPI_ISL_618750, EPI_ISL_618752, EPI_ISL_618754, EPI_ISL_618756, EPI_ISL_618757, EPI_ISL_618759, EPI_ISL_618760,
EPI_ISL_618761, EPI_ISL_618762, EPI_ISL_618763, EPI_ISL_618770, EPI_ISL_618772, EPI_ISL_618774, EPI_ISL_618775, EPI_ISL_618776, EPI_ISL_618777, EPI_ISL_618778, EPI_ISL_618779, EPI_ISL_618780, EPI_ISL_618781, EPI_ISL_618783, EPI_ISL_618784, EPI_ISL_618786, EPI_ISL_618787, EPI_ISL_618791,
EPI_ISL_618792, EPI_ISL_618793, EPI_ISL_618794, EPI_ISL_618795, EPI_ISL_618796, EPI_ISL_618798, EPI_ISL_618801, EPI_ISL_618802, EPI_ISL_618803, EPI_ISL_618804, EPI_ISL_618805, EPI_ISL_618806, EPI_ISL_618807, EPI_ISL_618808, EPI_ISL_618809, EPI_ISL_618811, EPI_ISL_618812, EPI_ISL_618813,
EPI_ISL_618814, EPI_ISL_618815, EPI_ISL_618817, EPI_ISL_618818, EPI_ISL_618819, EPI_ISL_618820, EPI_ISL_618821, EPI_ISL_618822, EPI_ISL_618823, EPI_ISL_618824, EPI_ISL_618825, EPI_ISL_618827, EPI_ISL_618829, EPI_ISL_618830, EPI_ISL_618831, EPI_ISL_618832, EPI_ISL_618833, EPI_ISL_618834,
EPI_ISL_618835, EPI_ISL_618836, EPI_ISL_618837, EPI_ISL_618838, EPI_ISL_618839, EPI_ISL_618840, EPI_ISL_618842, EPI_ISL_618843, EPI_ISL_618844, EPI_ISL_618845, EPI_ISL_618846, EPI_ISL_618847, EPI_ISL_618849, EPI_ISL_618850, EPI_ISL_618851, EPI_ISL_618852, EPI_ISL_618853, EPI_ISL_618854,
EPI_ISL_618855, EPI_ISL_618856, EPI_ISL_618857, EPI_ISL_618858, EPI_ISL_618859, EPI_ISL_618860, EPI_ISL_618861, EPI_ISL_618862, EPI_ISL_618863, EPI_ISL_618864, EPI_ISL_618865, EPI_ISL_618866, EPI_ISL_618867, EPI_ISL_618868, EPI_ISL_618869, EPI_ISL_618870, EPI_ISL_618871, EPI_ISL_618872,
EPI_ISL_618873, EPI_ISL_618874, EPI_ISL_618875, EPI_ISL_618876, EPI_ISL_618877, EPI_ISL_618878, EPI_ISL_618879, EPI_ISL_618880, EPI_ISL_618881, EPI_ISL_618882, EPI_ISL_618884, EPI_ISL_618885, EPI_ISL_618886, EPI_ISL_618887, EPI_ISL_618888, EPI_ISL_618889, EPI_ISL_618890, EPI_ISL_618891,
EPI_ISL_618892, EPI_ISL_618893, EPI_ISL_618894, EPI_ISL_618895, EPI_ISL_618896, EPI_ISL_618897, EPI_ISL_618898, EPI_ISL_618899, EPI_ISL_618900, EPI_ISL_618901, EPI_ISL_618902, EPI_ISL_618903, EPI_ISL_618904, EPI_ISL_618957, EPI_ISL_618958, EPI_ISL_618959, EPI_ISL_618960, EPI_ISL_618961,
EPI_ISL_618974, EPI_ISL_618975, EPI_ISL_618976, EPI_ISL_618977, EPI_ISL_618978, EPI_ISL_618979, EPI_ISL_618980, EPI_ISL_618981, EPI_ISL_618982, EPI_ISL_618983, EPI_ISL_618984, EPI_ISL_618985, EPI_ISL_618986, EPI_ISL_618987, EPI_ISL_618988, EPI_ISL_618989, EPI_ISL_618990, EPI_ISL_618991,
EPI_ISL_618992, EPI_ISL_618993, EPI_ISL_618994, EPI_ISL_618995, EPI_ISL_618996, EPI_ISL_618997, EPI_ISL_618998, EPI_ISL_618999, EPI_ISL_619000, EPI_ISL_619001, EPI_ISL_619002, EPI_ISL_619003, EPI_ISL_619004, EPI_ISL_619005, EPI_ISL_619006, EPI_ISL_619007, EPI_ISL_619008, EPI_ISL_619009,
EPI_ISL_619010, EPI_ISL_619011, EPI_ISL_619012, EPI_ISL_619013, EPI_ISL_619014, EPI_ISL_619015, EPI_ISL_619016, EPI_ISL_619017, EPI_ISL_619018, EPI_ISL_619019, EPI_ISL_619020, EPI_ISL_619021, EPI_ISL_619022, EPI_ISL_619023, EPI_ISL_619024, EPI_ISL_619025, EPI_ISL_619026, EPI_ISL_619027,
EPI_ISL_619028, EPI_ISL_619029, EPI_ISL_619030, EPI_ISL_619031, EPI_ISL_619032, EPI_ISL_619033, EPI_ISL_619034, EPI_ISL_619035, EPI_ISL_619036, EPI_ISL_619037, EPI_ISL_619038, EPI_ISL_619039, EPI_ISL_619040, EPI_ISL_619041, EPI_ISL_619042, EPI_ISL_619043, EPI_ISL_619044, EPI_ISL_619045,
EPI_ISL_619046, EPI_ISL_619047, EPI_ISL_619048, EPI_ISL_619049, EPI_ISL_619050, EPI_ISL_619051, EPI_ISL_619053, EPI_ISL_619054, EPI_ISL_619055, EPI_ISL_619056, EPI_ISL_619057, EPI_ISL_619058, EPI_ISL_619059, EPI_ISL_619083, EPI_ISL_619084, EPI_ISL_619085, EPI_ISL_619086, EPI_ISL_619087,
EPI_ISL_619088, EPI_ISL_619089, EPI_ISL_619090, EPI_ISL_619091, EPI_ISL_619112, EPI_ISL_619113, EPI_ISL_619114, EPI_ISL_619115, EPI_ISL_619116, EPI_ISL_619117, EPI_ISL_619118, EPI_ISL_619119, EPI_ISL_619120, EPI_ISL_619121, EPI_ISL_619122, EPI_ISL_619123, EPI_ISL_619124, EPI_ISL_619125,
EPI_ISL_619126, EPI_ISL_619127, EPI_ISL_619128, EPI_ISL_619129, EPI_ISL_619130, EPI_ISL_619131, EPI_ISL_619132, EPI_ISL_619133, EPI_ISL_619134, EPI_ISL_619135, EPI_ISL_619136, EPI_ISL_619137, EPI_ISL_619138, EPI_ISL_619139, EPI_ISL_619140, EPI_ISL_619141, EPI_ISL_619142, EPI_ISL_619143,
EPI_ISL_619144, EPI_ISL_619145, EPI_ISL_619146, EPI_ISL_619147, EPI_ISL_619148, EPI_ISL_619149, EPI_ISL_619150, EPI_ISL_619151, EPI_ISL_619152, EPI_ISL_619153, EPI_ISL_619154, EPI_ISL_619155, EPI_ISL_619156, EPI_ISL_619157, EPI_ISL_619158, EPI_ISL_619159, EPI_ISL_619160, EPI_ISL_619161,
EPI_ISL_619162, EPI_ISL_619163, EPI_ISL_619164, EPI_ISL_619165, EPI_ISL_619166, EPI_ISL_619167, EPI_ISL_619168, EPI_ISL_619169, EPI_ISL_619170, EPI_ISL_619171, EPI_ISL_619172, EPI_ISL_619173, EPI_ISL_619174, EPI_ISL_619175, EPI_ISL_619176, EPI_ISL_619177, EPI_ISL_619178, EPI_ISL_619179,
EPI_ISL_619180, EPI_ISL_619181, EPI_ISL_619182, EPI_ISL_619183, EPI_ISL_619184, EPI_ISL_619185, EPI_ISL_619186, EPI_ISL_619187, EPI_ISL_619188, EPI_ISL_619189, EPI_ISL_619190, EPI_ISL_619191, EPI_ISL_619192, EPI_ISL_619193, EPI_ISL_619194, EPI_ISL_619195, EPI_ISL_619196, EPI_ISL_619197,
EPI_ISL_619198, EPI_ISL_619199, EPI_ISL_619200, EPI_ISL_619201, EPI_ISL_619202, EPI_ISL_619203, EPI_ISL_619204, EPI_ISL_619205, EPI_ISL_619206, EPI_ISL_619207, EPI_ISL_619208, EPI_ISL_619209, EPI_ISL_619210, EPI_ISL_619211, EPI_ISL_619212, EPI_ISL_619213, EPI_ISL_619214, EPI_ISL_619215,
EPI_ISL_619216, EPI_ISL_619217, EPI_ISL_619218, EPI_ISL_619219, EPI_ISL_619220, EPI_ISL_619221, EPI_ISL_619222, EPI_ISL_619223, EPI_ISL_619224, EPI_ISL_619225, EPI_ISL_619226, EPI_ISL_619227, EPI_ISL_619228, EPI_ISL_619229, EPI_ISL_619230, EPI_ISL_619231, EPI_ISL_619232, EPI_ISL_619233,
EPI_ISL_619234, EPI_ISL_619235, EPI_ISL_619236, EPI_ISL_619237, EPI_ISL_619238, EPI_ISL_619239, EPI_ISL_619240, EPI_ISL_619241, EPI_ISL_619242, EPI_ISL_619243, EPI_ISL_619244, EPI_ISL_619245, EPI_ISL_619246, EPI_ISL_619247, EPI_ISL_619248, EPI_ISL_619249, EPI_ISL_619250, EPI_ISL_619251,
EPI_ISL_619252, EPI_ISL_619253, EPI_ISL_619254, EPI_ISL_619255, EPI_ISL_619256, EPI_ISL_619257, EPI_ISL_619258, EPI_ISL_619259, EPI_ISL_619260, EPI_ISL_619261, EPI_ISL_619262, EPI_ISL_619263, EPI_ISL_619264, EPI_ISL_619265, EPI_ISL_619266, EPI_ISL_619267, EPI_ISL_619268, EPI_ISL_619269,
EPI_ISL_619270, EPI_ISL_619271, EPI_ISL_619272, EPI_ISL_619273, EPI_ISL_619274, EPI_ISL_619275, EPI_ISL_619276, EPI_ISL_619277, EPI_ISL_619278, EPI_ISL_619279, EPI_ISL_619280, EPI_ISL_619281, EPI_ISL_619282, EPI_ISL_619283, EPI_ISL_619284, EPI_ISL_619285, EPI_ISL_619286, EPI_ISL_619287,
EPI_ISL_619288, EPI_ISL_619289, EPI_ISL_619290, EPI_ISL_619291, EPI_ISL_619292, EPI_ISL_619293, EPI_ISL_619294, EPI_ISL_619295, EPI_ISL_619296, EPI_ISL_619297, EPI_ISL_619298, EPI_ISL_619299, EPI_ISL_619300, EPI_ISL_619301, EPI_ISL_619302, EPI_ISL_619303, EPI_ISL_619304, EPI_ISL_619305,
EPI_ISL_619306, EPI_ISL_619307, EPI_ISL_619308, EPI_ISL_619309, EPI_ISL_619310, EPI_ISL_619311, EPI_ISL_619312, EPI_ISL_619313, EPI_ISL_619314, EPI_ISL_619315, EPI_ISL_619316, EPI_ISL_619317, EPI_ISL_619318, EPI_ISL_619319, EPI_ISL_619320, EPI_ISL_619321, EPI_ISL_619322, EPI_ISL_619323,
EPI_ISL_619324, EPI_ISL_619325, EPI_ISL_619326, EPI_ISL_619327, EPI_ISL_619328, EPI_ISL_619329, EPI_ISL_619330, EPI_ISL_619331, EPI_ISL_619332, EPI_ISL_619333, EPI_ISL_619334, EPI_ISL_619335, EPI_ISL_619336, EPI_ISL_619337, EPI_ISL_619338, EPI_ISL_619339, EPI_ISL_619340, EPI_ISL_619341,
EPI_ISL_619342, EPI_ISL_619343, EPI_ISL_619344, EPI_ISL_619345, EPI_ISL_619346, EPI_ISL_619347, EPI_ISL_619348, EPI_ISL_619349, EPI_ISL_619350, EPI_ISL_619351, EPI_ISL_619352, EPI_ISL_619353, EPI_ISL_619354, EPI_ISL_619355, EPI_ISL_619356, EPI_ISL_619357, EPI_ISL_619358, EPI_ISL_619359,
EPI_ISL_619360, EPI_ISL_619361, EPI_ISL_619362, EPI_ISL_619363, EPI_ISL_619364, EPI_ISL_619365, EPI_ISL_619366, EPI_ISL_619367, EPI_ISL_619368, EPI_ISL_619369, EPI_ISL_619370, EPI_ISL_619371, EPI_ISL_619372, EPI_ISL_619373, EPI_ISL_619374, EPI_ISL_619375, EPI_ISL_619376, EPI_ISL_619377,
EPI_ISL_619378, EPI_ISL_619379, EPI_ISL_619380, EPI_ISL_619381, EPI_ISL_619382, EPI_ISL_619383, EPI_ISL_619384, EPI_ISL_619385, EPI_ISL_619386, EPI_ISL_619387, EPI_ISL_619388, EPI_ISL_619389, EPI_ISL_619390, EPI_ISL_619391, EPI_ISL_619392, EPI_ISL_619393, EPI_ISL_619394, EPI_ISL_619395,
EPI_ISL_619396, EPI_ISL_619397, EPI_ISL_619398, EPI_ISL_619399, EPI_ISL_619400, EPI_ISL_619401, EPI_ISL_619402, EPI_ISL_619403, EPI_ISL_619404, EPI_ISL_619405, EPI_ISL_619406, EPI_ISL_619407, EPI_ISL_619408, EPI_ISL_619409, EPI_ISL_619410, EPI_ISL_619411, EPI_ISL_619412, EPI_ISL_619413,
EPI_ISL_619414, EPI_ISL_619416, EPI_ISL_619420, EPI_ISL_619422, EPI_ISL_619423, EPI_ISL_619425, EPI_ISL_619426, EPI_ISL_619427, EPI_ISL_619428, EPI_ISL_619429, EPI_ISL_619430, EPI_ISL_619431, EPI_ISL_619432, EPI_ISL_619433, EPI_ISL_619434, EPI_ISL_619435, EPI_ISL_619436, EPI_ISL_619437,
EPI_ISL_619438, EPI_ISL_619440, EPI_ISL_619442, EPI_ISL_619444, EPI_ISL_619445, EPI_ISL_619447, EPI_ISL_619450, EPI_ISL_619451, EPI_ISL_619452, EPI_ISL_619453, EPI_ISL_619454, EPI_ISL_619455, EPI_ISL_619456, EPI_ISL_619459, EPI_ISL_619461, EPI_ISL_619462, EPI_ISL_619465, EPI_ISL_619466,
EPI_ISL_619468, EPI_ISL_619471, EPI_ISL_619472, EPI_ISL_619475, EPI_ISL_619476, EPI_ISL_619477, EPI_ISL_619478, EPI_ISL_619484, EPI_ISL_619485, EPI_ISL_619486, EPI_ISL_619489, EPI_ISL_619491, EPI_ISL_619492, EPI_ISL_619493, EPI_ISL_619494, EPI_ISL_619495, EPI_ISL_619496, EPI_ISL_619497,
EPI_ISL_619498, EPI_ISL_619499, EPI_ISL_619500, EPI_ISL_619501, EPI_ISL_619502, EPI_ISL_619503, EPI_ISL_619504, EPI_ISL_619505, EPI_ISL_619508, EPI_ISL_619509, EPI_ISL_619510, EPI_ISL_619513, EPI_ISL_619515, EPI_ISL_619517, EPI_ISL_619519, EPI_ISL_619520, EPI_ISL_619521, EPI_ISL_619522,
EPI_ISL_619523, EPI_ISL_619524, EPI_ISL_619526, EPI_ISL_619528, EPI_ISL_619529, EPI_ISL_619530, EPI_ISL_619531, EPI_ISL_619533, EPI_ISL_619534, EPI_ISL_619536, EPI_ISL_619537, EPI_ISL_619538, EPI_ISL_619539, EPI_ISL_619540, EPI_ISL_619541, EPI_ISL_619542, EPI_ISL_619543, EPI_ISL_619544,
EPI_ISL_619545, EPI_ISL_619546, EPI_ISL_619547, EPI_ISL_619548, EPI_ISL_619549, EPI_ISL_619550, EPI_ISL_619551, EPI_ISL_619552, EPI_ISL_619553, EPI_ISL_619554, EPI_ISL_619555, EPI_ISL_619556, EPI_ISL_619558, EPI_ISL_619559, EPI_ISL_619560, EPI_ISL_619561, EPI_ISL_619562, EPI_ISL_619563,
EPI_ISL_619564, EPI_ISL_619565, EPI_ISL_619566, EPI_ISL_619567, EPI_ISL_619568, EPI_ISL_619569, EPI_ISL_619570, EPI_ISL_619571, EPI_ISL_619572, EPI_ISL_619573, EPI_ISL_619574, EPI_ISL_619575, EPI_ISL_619576, EPI_ISL_619577, EPI_ISL_619579, EPI_ISL_619581, EPI_ISL_619582, EPI_ISL_619583,
EPI_ISL_619585, EPI_ISL_619586, EPI_ISL_619587, EPI_ISL_619588, EPI_ISL_619589, EPI_ISL_619590, EPI_ISL_619591, EPI_ISL_619592, EPI_ISL_619593, EPI_ISL_619594, EPI_ISL_619599, EPI_ISL_619600, EPI_ISL_619603, EPI_ISL_619604, EPI_ISL_619605, EPI_ISL_619606, EPI_ISL_619610, EPI_ISL_619613,
EPI_ISL_619614, EPI_ISL_619615, EPI_ISL_619616, EPI_ISL_619619, EPI_ISL_619621, EPI_ISL_619622, EPI_ISL_619623, EPI_ISL_619624, EPI_ISL_619625, EPI_ISL_619626, EPI_ISL_619627, EPI_ISL_619628, EPI_ISL_619629, EPI_ISL_619630, EPI_ISL_619631, EPI_ISL_619632, EPI_ISL_619634, EPI_ISL_619635,
EPI_ISL_619636, EPI_ISL_619637, EPI_ISL_619638, EPI_ISL_619639, EPI_ISL_619640, EPI_ISL_619645, EPI_ISL_619646, EPI_ISL_619647, EPI_ISL_619648, EPI_ISL_619649, EPI_ISL_619650, EPI_ISL_619652, EPI_ISL_619654, EPI_ISL_619655, EPI_ISL_619660, EPI_ISL_619662, EPI_ISL_619663, EPI_ISL_619664,
EPI_ISL_619665, EPI_ISL_619666, EPI_ISL_619667, EPI_ISL_619668, EPI_ISL_619670, EPI_ISL_619673, EPI_ISL_619674, EPI_ISL_619675, EPI_ISL_619676, EPI_ISL_619677, EPI_ISL_619678, EPI_ISL_619679, EPI_ISL_619680, EPI_ISL_619682, EPI_ISL_619684, EPI_ISL_619685, EPI_ISL_619686, EPI_ISL_619689,
EPI_ISL_619690, EPI_ISL_619691, EPI_ISL_619692, EPI_ISL_619693, EPI_ISL_619694, EPI_ISL_619695, EPI_ISL_619696, EPI_ISL_619697, EPI_ISL_619700, EPI_ISL_619701, EPI_ISL_619702, EPI_ISL_619704, EPI_ISL_619706, EPI_ISL_619707, EPI_ISL_619709, EPI_ISL_619712, EPI_ISL_619713, EPI_ISL_619714,
EPI_ISL_619715, EPI_ISL_619716, EPI_ISL_619717, EPI_ISL_619718, EPI_ISL_619719, EPI_ISL_619720, EPI_ISL_619721, EPI_ISL_619722, EPI_ISL_619723, EPI_ISL_619726, EPI_ISL_619727, EPI_ISL_619728, EPI_ISL_619729, EPI_ISL_619730, EPI_ISL_619731, EPI_ISL_619732, EPI_ISL_619733, EPI_ISL_619734,
EPI_ISL_619735, EPI_ISL_619739, EPI_ISL_619740, EPI_ISL_619742, EPI_ISL_619744, EPI_ISL_619745, EPI_ISL_619746, EPI_ISL_619747, EPI_ISL_619748, EPI_ISL_619750, EPI_ISL_619752, EPI_ISL_619753, EPI_ISL_619754, EPI_ISL_619755, EPI_ISL_619756, EPI_ISL_619758, EPI_ISL_619759, EPI_ISL_619760,
EPI_ISL_619761, EPI_ISL_619763, EPI_ISL_619765, EPI_ISL_619766, EPI_ISL_619767, EPI_ISL_619769, EPI_ISL_619771, EPI_ISL_619772, EPI_ISL_619773, EPI_ISL_619774, EPI_ISL_619775, EPI_ISL_619776, EPI_ISL_619778, EPI_ISL_619779, EPI_ISL_619780, EPI_ISL_619781, EPI_ISL_619782, EPI_ISL_619783,
EPI_ISL_619784, EPI_ISL_619785, EPI_ISL_619786, EPI_ISL_619787, EPI_ISL_619789, EPI_ISL_619791, EPI_ISL_619792, EPI_ISL_619793, EPI_ISL_619794, EPI_ISL_619795, EPI_ISL_619796, EPI_ISL_619798, EPI_ISL_619799, EPI_ISL_619800, EPI_ISL_619801, EPI_ISL_619802, EPI_ISL_619803, EPI_ISL_619804,
EPI_ISL_619805, EPI_ISL_619806, EPI_ISL_619808, EPI_ISL_619813, EPI_ISL_619814, EPI_ISL_619816, EPI_ISL_619817, EPI_ISL_619818, EPI_ISL_619819, EPI_ISL_619820, EPI_ISL_619821, EPI_ISL_619822, EPI_ISL_619823, EPI_ISL_619824, EPI_ISL_619825, EPI_ISL_619826, EPI_ISL_619827, EPI_ISL_619828,
EPI_ISL_619830, EPI_ISL_619831, EPI_ISL_619833, EPI_ISL_619834, EPI_ISL_619835, EPI_ISL_619836, EPI_ISL_619837, EPI_ISL_619839, EPI_ISL_619840, EPI_ISL_619842, EPI_ISL_619843, EPI_ISL_619844, EPI_ISL_619845, EPI_ISL_619846, EPI_ISL_619847, EPI_ISL_619848, EPI_ISL_619849, EPI_ISL_619850,
EPI_ISL_619851, EPI_ISL_619853, EPI_ISL_619854, EPI_ISL_619855, EPI_ISL_619856, EPI_ISL_619857, EPI_ISL_619858, EPI_ISL_619859, EPI_ISL_619860, EPI_ISL_619861, EPI_ISL_619862, EPI_ISL_619864, EPI_ISL_619865, EPI_ISL_619866, EPI_ISL_619868, EPI_ISL_619869, EPI_ISL_619870, EPI_ISL_619871,
EPI_ISL_619872, EPI_ISL_619873, EPI_ISL_619877, EPI_ISL_619879, EPI_ISL_619880, EPI_ISL_619881, EPI_ISL_619882, EPI_ISL_619885, EPI_ISL_619886, EPI_ISL_619887, EPI_ISL_619889, EPI_ISL_619891, EPI_ISL_619893, EPI_ISL_619894, EPI_ISL_619895, EPI_ISL_619897, EPI_ISL_619898, EPI_ISL_619899,
EPI_ISL_619902, EPI_ISL_619903, EPI_ISL_619905, EPI_ISL_619906, EPI_ISL_619907, EPI_ISL_619908, EPI_ISL_619909, EPI_ISL_619910, EPI_ISL_619911, EPI_ISL_619912, EPI_ISL_619914, EPI_ISL_619915, EPI_ISL_619916, EPI_ISL_619917, EPI_ISL_619918, EPI_ISL_619919, EPI_ISL_619920, EPI_ISL_619921,
EPI_ISL_619922, EPI_ISL_619923, EPI_ISL_619924, EPI_ISL_619925, EPI_ISL_619926, EPI_ISL_619927, EPI_ISL_619928, EPI_ISL_619929, EPI_ISL_619930, EPI_ISL_619931, EPI_ISL_619932, EPI_ISL_619933, EPI_ISL_619934, EPI_ISL_619935, EPI_ISL_619936, EPI_ISL_619937, EPI_ISL_619938, EPI_ISL_619939,
EPI_ISL_619940, EPI_ISL_619941, EPI_ISL_619943, EPI_ISL_619944, EPI_ISL_619945, EPI_ISL_619946, EPI_ISL_619949, EPI_ISL_619950, EPI_ISL_619951, EPI_ISL_619952, EPI_ISL_619954, EPI_ISL_619955, EPI_ISL_619956, EPI_ISL_619957, EPI_ISL_619958, EPI_ISL_619959, EPI_ISL_619960, EPI_ISL_619961,
EPI_ISL_619962, EPI_ISL_619964, EPI_ISL_619965, EPI_ISL_619967, EPI_ISL_619968, EPI_ISL_619969, EPI_ISL_619970, EPI_ISL_619971, EPI_ISL_619972, EPI_ISL_619973, EPI_ISL_619975, EPI_ISL_619976, EPI_ISL_619978, EPI_ISL_619979, EPI_ISL_619980, EPI_ISL_619981, EPI_ISL_619982, EPI_ISL_619983,
EPI_ISL_619986, EPI_ISL_619989, EPI_ISL_619991, EPI_ISL_619992, EPI_ISL_619993, EPI_ISL_619994, EPI_ISL_619995, EPI_ISL_619996, EPI_ISL_619997, EPI_ISL_619999, EPI_ISL_620000, EPI_ISL_620001, EPI_ISL_620003, EPI_ISL_620004, EPI_ISL_620006, EPI_ISL_620010, EPI_ISL_620011, EPI_ISL_620012,
EPI_ISL_620013, EPI_ISL_620014, EPI_ISL_620015, EPI_ISL_620016, EPI_ISL_620017, EPI_ISL_620018, EPI_ISL_620019, EPI_ISL_620020, EPI_ISL_620021, EPI_ISL_620022, EPI_ISL_620023, EPI_ISL_620024, EPI_ISL_620025, EPI_ISL_620026, EPI_ISL_620027, EPI_ISL_620028, EPI_ISL_620029, EPI_ISL_620030,

EPI_ISL_620032, EPI_ISL_620033, EPI_ISL_620034, EPI_ISL_620035, EPI_ISL_620036, EPI_ISL_620037, EPI_ISL_620038, EPI_ISL_620039, EPI_ISL_620040, EPI_ISL_620045, EPI_ISL_620046, EPI_ISL_620047, EPI_ISL_620048, EPI_ISL_620049, EPI_ISL_620052, EPI_ISL_620054, EPI_ISL_620055, EPI_ISL_620056,
EPI_ISL_620058, EPI_ISL_620059, EPI_ISL_620060, EPI_ISL_620061, EPI_ISL_620062, EPI_ISL_620063, EPI_ISL_620064, EPI_ISL_620065, EPI_ISL_620066, EPI_ISL_620067, EPI_ISL_620068, EPI_ISL_620069, EPI_ISL_620070, EPI_ISL_620071, EPI_ISL_620072, EPI_ISL_620073, EPI_ISL_620074, EPI_ISL_620076,
EPI_ISL_620078, EPI_ISL_620079, EPI_ISL_620081, EPI_ISL_620082, EPI_ISL_620083, EPI_ISL_620084, EPI_ISL_620085, EPI_ISL_620086, EPI_ISL_620087, EPI_ISL_620088, EPI_ISL_620089, EPI_ISL_620090, EPI_ISL_620091, EPI_ISL_620093, EPI_ISL_620094, EPI_ISL_620097, EPI_ISL_620098, EPI_ISL_620100,
EPI_ISL_620101, EPI_ISL_620102, EPI_ISL_620103, EPI_ISL_620104, EPI_ISL_620105, EPI_ISL_620107, EPI_ISL_620109, EPI_ISL_620110, EPI_ISL_620111, EPI_ISL_620112, EPI_ISL_620113, EPI_ISL_620114, EPI_ISL_620115, EPI_ISL_620120, EPI_ISL_620123, EPI_ISL_620124, EPI_ISL_620125, EPI_ISL_620126,
EPI_ISL_620127, EPI_ISL_620128, EPI_ISL_620130, EPI_ISL_620132, EPI_ISL_620133, EPI_ISL_620134, EPI_ISL_620135, EPI_ISL_620136, EPI_ISL_620139, EPI_ISL_620140, EPI_ISL_620141, EPI_ISL_620142, EPI_ISL_620145, EPI_ISL_620146, EPI_ISL_620148, EPI_ISL_620149, EPI_ISL_620150, EPI_ISL_620151,
EPI_ISL_620153, EPI_ISL_620154, EPI_ISL_620155, EPI_ISL_620156, EPI_ISL_620157, EPI_ISL_620158, EPI_ISL_620159, EPI_ISL_620160, EPI_ISL_620161, EPI_ISL_620162, EPI_ISL_620163, EPI_ISL_620167, EPI_ISL_620169, EPI_ISL_620170, EPI_ISL_620171, EPI_ISL_620172, EPI_ISL_620173, EPI_ISL_620174,
EPI_ISL_620176, EPI_ISL_620177, EPI_ISL_620178, EPI_ISL_620179, EPI_ISL_620180, EPI_ISL_620183, EPI_ISL_620184, EPI_ISL_620185, EPI_ISL_620186, EPI_ISL_620187, EPI_ISL_620189, EPI_ISL_620192, EPI_ISL_620193, EPI_ISL_620194, EPI_ISL_620195, EPI_ISL_620196, EPI_ISL_620197, EPI_ISL_620198,
EPI_ISL_620200, EPI_ISL_620201, EPI_ISL_620202, EPI_ISL_620203, EPI_ISL_620204, EPI_ISL_620205, EPI_ISL_620206, EPI_ISL_620207, EPI_ISL_620208, EPI_ISL_620209, EPI_ISL_620210, EPI_ISL_620211, EPI_ISL_620213, EPI_ISL_620214, EPI_ISL_620215, EPI_ISL_620216, EPI_ISL_620217, EPI_ISL_620218,
EPI_ISL_620219, EPI_ISL_620220, EPI_ISL_620221, EPI_ISL_620222, EPI_ISL_620223, EPI_ISL_620224, EPI_ISL_620225, EPI_ISL_620226, EPI_ISL_620227, EPI_ISL_620228, EPI_ISL_620229, EPI_ISL_620231, EPI_ISL_620236, EPI_ISL_620237, EPI_ISL_620238, EPI_ISL_620239, EPI_ISL_620240, EPI_ISL_620241,
EPI_ISL_620242, EPI_ISL_620243, EPI_ISL_620244, EPI_ISL_620245, EPI_ISL_620246, EPI_ISL_620247, EPI_ISL_620248, EPI_ISL_620249, EPI_ISL_620250, EPI_ISL_620251, EPI_ISL_620252, EPI_ISL_620254, EPI_ISL_620255, EPI_ISL_620256, EPI_ISL_620257, EPI_ISL_620258, EPI_ISL_620259, EPI_ISL_620260,
EPI_ISL_620261, EPI_ISL_620262, EPI_ISL_620263, EPI_ISL_620264, EPI_ISL_620266, EPI_ISL_620268, EPI_ISL_620269, EPI_ISL_620270, EPI_ISL_620271, EPI_ISL_620272, EPI_ISL_620273, EPI_ISL_620274, EPI_ISL_620275, EPI_ISL_620277, EPI_ISL_620278, EPI_ISL_620279, EPI_ISL_620280, EPI_ISL_620281,
EPI_ISL_620282, EPI_ISL_620283, EPI_ISL_620284, EPI_ISL_620286, EPI_ISL_620287, EPI_ISL_620288, EPI_ISL_620289, EPI_ISL_620290, EPI_ISL_620292, EPI_ISL_620293, EPI_ISL_620294, EPI_ISL_620295, EPI_ISL_620296, EPI_ISL_620298, EPI_ISL_620299, EPI_ISL_620300, EPI_ISL_620301, EPI_ISL_620303,
EPI_ISL_620305, EPI_ISL_620306, EPI_ISL_620307, EPI_ISL_620308, EPI_ISL_620309, EPI_ISL_620310, EPI_ISL_620311, EPI_ISL_620312, EPI_ISL_620313, EPI_ISL_620314, EPI_ISL_620315, EPI_ISL_620316, EPI_ISL_620317, EPI_ISL_620318, EPI_ISL_620319, EPI_ISL_620320, EPI_ISL_620321, EPI_ISL_620322,
EPI_ISL_620323, EPI_ISL_620324, EPI_ISL_620325, EPI_ISL_620326, EPI_ISL_620328, EPI_ISL_620329, EPI_ISL_620330, EPI_ISL_620331, EPI_ISL_620333, EPI_ISL_620335, EPI_ISL_620336, EPI_ISL_620337, EPI_ISL_620338, EPI_ISL_620339, EPI_ISL_620340, EPI_ISL_620341, EPI_ISL_620342, EPI_ISL_620343,
EPI_ISL_620344, EPI_ISL_620348, EPI_ISL_620350, EPI_ISL_620351, EPI_ISL_620352, EPI_ISL_620354, EPI_ISL_620355, EPI_ISL_620356, EPI_ISL_620357, EPI_ISL_620358, EPI_ISL_620359, EPI_ISL_620360, EPI_ISL_620361, EPI_ISL_620362, EPI_ISL_620363, EPI_ISL_620364, EPI_ISL_620365, EPI_ISL_620366,
EPI_ISL_620367, EPI_ISL_620368, EPI_ISL_620369, EPI_ISL_620370, EPI_ISL_620371, EPI_ISL_620372, EPI_ISL_620374, EPI_ISL_620376, EPI_ISL_620377, EPI_ISL_620378, EPI_ISL_620379, EPI_ISL_620380, EPI_ISL_620381, EPI_ISL_620383, EPI_ISL_620384, EPI_ISL_620385, EPI_ISL_620386, EPI_ISL_620389,
EPI_ISL_620391, EPI_ISL_620392, EPI_ISL_620393, EPI_ISL_620396, EPI_ISL_620397, EPI_ISL_620398, EPI_ISL_620399, EPI_ISL_620400, EPI_ISL_620401, EPI_ISL_620403, EPI_ISL_620404, EPI_ISL_620762, EPI_ISL_620763, EPI_ISL_620764, EPI_ISL_620765, EPI_ISL_620766, EPI_ISL_620767, EPI_ISL_620768,
EPI_ISL_620769, EPI_ISL_620770, EPI_ISL_620771, EPI_ISL_620772, EPI_ISL_620773, EPI_ISL_620774, EPI_ISL_620775, EPI_ISL_620776, EPI_ISL_620777, EPI_ISL_620778, EPI_ISL_620780, EPI_ISL_620781, EPI_ISL_620782, EPI_ISL_620783, EPI_ISL_620784, EPI_ISL_620785, EPI_ISL_620786, EPI_ISL_620787,
EPI_ISL_620788, EPI_ISL_620789, EPI_ISL_620790, EPI_ISL_620791, EPI_ISL_620792, EPI_ISL_620793, EPI_ISL_620794, EPI_ISL_620795, EPI_ISL_620796, EPI_ISL_620797, EPI_ISL_620800, EPI_ISL_620801, EPI_ISL_620802, EPI_ISL_620803, EPI_ISL_620809, EPI_ISL_620810, EPI_ISL_620811, EPI_ISL_620812,
EPI_ISL_620813, EPI_ISL_620814, EPI_ISL_620815, EPI_ISL_620816, EPI_ISL_620817, EPI_ISL_620818, EPI_ISL_620819, EPI_ISL_620820, EPI_ISL_620821, EPI_ISL_620822, EPI_ISL_620823, EPI_ISL_620824, EPI_ISL_620825, EPI_ISL_620826, EPI_ISL_620827, EPI_ISL_620828, EPI_ISL_620829, EPI_ISL_620830,
EPI_ISL_620831, EPI_ISL_620832, EPI_ISL_620833, EPI_ISL_620834, EPI_ISL_620835, EPI_ISL_620836, EPI_ISL_620837, EPI_ISL_620838, EPI_ISL_620839, EPI_ISL_620840, EPI_ISL_620841, EPI_ISL_620842, EPI_ISL_620843, EPI_ISL_620845, EPI_ISL_620846, EPI_ISL_620847, EPI_ISL_620848, EPI_ISL_620849,
EPI_ISL_620850, EPI_ISL_620851, EPI_ISL_620852, EPI_ISL_620853, EPI_ISL_620854, EPI_ISL_620855, EPI_ISL_620856, EPI_ISL_620857, EPI_ISL_620858, EPI_ISL_620859, EPI_ISL_620860, EPI_ISL_620861, EPI_ISL_620862, EPI_ISL_620863, EPI_ISL_620864, EPI_ISL_620865, EPI_ISL_620866, EPI_ISL_620867,
EPI_ISL_620868, EPI_ISL_620869, EPI_ISL_620877, EPI_ISL_620879, EPI_ISL_620880, EPI_ISL_620881, EPI_ISL_620882, EPI_ISL_620883, EPI_ISL_620884, EPI_ISL_620885, EPI_ISL_620886, EPI_ISL_620887, EPI_ISL_620888, EPI_ISL_620889, EPI_ISL_620890, EPI_ISL_620892, EPI_ISL_620893, EPI_ISL_620894,
EPI_ISL_620895, EPI_ISL_620896, EPI_ISL_620897, EPI_ISL_620898, EPI_ISL_620899, EPI_ISL_620900, EPI_ISL_620901, EPI_ISL_620902, EPI_ISL_620903, EPI_ISL_620904, EPI_ISL_620905, EPI_ISL_620906, EPI_ISL_620908, EPI_ISL_620909, EPI_ISL_620910, EPI_ISL_620911, EPI_ISL_620912, EPI_ISL_620914,
EPI_ISL_620915, EPI_ISL_620916, EPI_ISL_620917, EPI_ISL_620918, EPI_ISL_620919, EPI_ISL_620920, EPI_ISL_620921, EPI_ISL_620922, EPI_ISL_620924, EPI_ISL_620925, EPI_ISL_620926, EPI_ISL_620927, EPI_ISL_620928, EPI_ISL_620929, EPI_ISL_620930, EPI_ISL_620932, EPI_ISL_620933, EPI_ISL_620934,
EPI_ISL_620935, EPI_ISL_620936, EPI_ISL_620937, EPI_ISL_620938, EPI_ISL_620939, EPI_ISL_620940, EPI_ISL_620941, EPI_ISL_620942, EPI_ISL_620943, EPI_ISL_620944, EPI_ISL_620945, EPI_ISL_620946, EPI_ISL_620947, EPI_ISL_620948, EPI_ISL_620949, EPI_ISL_620950, EPI_ISL_620951, EPI_ISL_620952,
EPI_ISL_620954, EPI_ISL_620955, EPI_ISL_620956, EPI_ISL_620957, EPI_ISL_620959, EPI_ISL_620960, EPI_ISL_620961, EPI_ISL_620962, EPI_ISL_620963, EPI_ISL_620964, EPI_ISL_620965, EPI_ISL_620966, EPI_ISL_620967, EPI_ISL_620968, EPI_ISL_620969, EPI_ISL_620970, EPI_ISL_620971, EPI_ISL_620973,
EPI_ISL_620974, EPI_ISL_620976, EPI_ISL_620977, EPI_ISL_620978, EPI_ISL_620979, EPI_ISL_620981, EPI_ISL_620983, EPI_ISL_620984, EPI_ISL_620985, EPI_ISL_620986, EPI_ISL_620987, EPI_ISL_620988, EPI_ISL_620989, EPI_ISL_620990, EPI_ISL_620991, EPI_ISL_620992, EPI_ISL_620993, EPI_ISL_620994,
EPI_ISL_620995, EPI_ISL_620996, EPI_ISL_620997, EPI_ISL_620999, EPI_ISL_621001, EPI_ISL_621002, EPI_ISL_621003, EPI_ISL_621004, EPI_ISL_621005, EPI_ISL_621006, EPI_ISL_621009, EPI_ISL_621010, EPI_ISL_621012, EPI_ISL_621013, EPI_ISL_621014, EPI_ISL_621015, EPI_ISL_621016, EPI_ISL_621018,
EPI_ISL_621019, EPI_ISL_621021, EPI_ISL_621022, EPI_ISL_621023, EPI_ISL_621024, EPI_ISL_621025, EPI_ISL_621026, EPI_ISL_621027, EPI_ISL_621029, EPI_ISL_621030, EPI_ISL_621031, EPI_ISL_621032, EPI_ISL_621033, EPI_ISL_621034, EPI_ISL_621035, EPI_ISL_621036, EPI_ISL_621037, EPI_ISL_621038,
EPI_ISL_621040, EPI_ISL_621041, EPI_ISL_621042, EPI_ISL_621043, EPI_ISL_621044, EPI_ISL_621045, EPI_ISL_621046, EPI_ISL_621047, EPI_ISL_621049, EPI_ISL_621050, EPI_ISL_621051, EPI_ISL_621052, EPI_ISL_621053, EPI_ISL_621054, EPI_ISL_621056, EPI_ISL_621057, EPI_ISL_621058, EPI_ISL_621059,
EPI_ISL_621060, EPI_ISL_621061, EPI_ISL_621062, EPI_ISL_621063, EPI_ISL_621064, EPI_ISL_621065, EPI_ISL_621066, EPI_ISL_621067, EPI_ISL_621068, EPI_ISL_621069, EPI_ISL_621070, EPI_ISL_621071, EPI_ISL_621072, EPI_ISL_621073, EPI_ISL_621074, EPI_ISL_621075, EPI_ISL_621076, EPI_ISL_621077,
EPI_ISL_621078, EPI_ISL_621079, EPI_ISL_621080, EPI_ISL_621081, EPI_ISL_621082, EPI_ISL_621083, EPI_ISL_621086, EPI_ISL_621087, EPI_ISL_621088, EPI_ISL_621089, EPI_ISL_621090, EPI_ISL_621091, EPI_ISL_621092, EPI_ISL_621093, EPI_ISL_621094, EPI_ISL_621095, EPI_ISL_621096, EPI_ISL_621097,
EPI_ISL_621098, EPI_ISL_621099, EPI_ISL_621100, EPI_ISL_621101, EPI_ISL_621102, EPI_ISL_621103, EPI_ISL_621104, EPI_ISL_621105, EPI_ISL_621106, EPI_ISL_621107, EPI_ISL_621108, EPI_ISL_621109, EPI_ISL_621110, EPI_ISL_621111, EPI_ISL_621112, EPI_ISL_621113, EPI_ISL_621114, EPI_ISL_621115,
EPI_ISL_621116, EPI_ISL_621117, EPI_ISL_621118, EPI_ISL_621120, EPI_ISL_621121, EPI_ISL_621122, EPI_ISL_621123, EPI_ISL_621124, EPI_ISL_621125, EPI_ISL_621126, EPI_ISL_621129, EPI_ISL_621130, EPI_ISL_621131, EPI_ISL_621133, EPI_ISL_621134, EPI_ISL_621135, EPI_ISL_621136, EPI_ISL_621137,
EPI_ISL_621138, EPI_ISL_621139, EPI_ISL_621140, EPI_ISL_621141, EPI_ISL_621142, EPI_ISL_621143, EPI_ISL_621144, EPI_ISL_621145, EPI_ISL_621146, EPI_ISL_621147, EPI_ISL_621150, EPI_ISL_621151, EPI_ISL_621153, EPI_ISL_621154, EPI_ISL_621155, EPI_ISL_621156, EPI_ISL_621157, EPI_ISL_621158,
EPI_ISL_621159, EPI_ISL_621160, EPI_ISL_621161, EPI_ISL_621162, EPI_ISL_621163, EPI_ISL_621164, EPI_ISL_621165, EPI_ISL_621166, EPI_ISL_621167, EPI_ISL_621168, EPI_ISL_621169, EPI_ISL_621170, EPI_ISL_621171, EPI_ISL_621172, EPI_ISL_621173, EPI_ISL_621174, EPI_ISL_621175, EPI_ISL_621176,
EPI_ISL_621177, EPI_ISL_621178, EPI_ISL_621179, EPI_ISL_621180, EPI_ISL_621181, EPI_ISL_621182, EPI_ISL_621183, EPI_ISL_621184, EPI_ISL_621185, EPI_ISL_621186, EPI_ISL_621187, EPI_ISL_621191, EPI_ISL_621192, EPI_ISL_621194, EPI_ISL_621195, EPI_ISL_621196, EPI_ISL_621197, EPI_ISL_621198,
EPI_ISL_621199, EPI_ISL_621200, EPI_ISL_621202, EPI_ISL_621203, EPI_ISL_621204, EPI_ISL_621205, EPI_ISL_621206, EPI_ISL_621207, EPI_ISL_621208, EPI_ISL_621209, EPI_ISL_621210, EPI_ISL_621211, EPI_ISL_621212, EPI_ISL_621213, EPI_ISL_621214, EPI_ISL_621215, EPI_ISL_621216, EPI_ISL_621217,
EPI_ISL_621218, EPI_ISL_621219, EPI_ISL_621220, EPI_ISL_621221, EPI_ISL_621222, EPI_ISL_621223, EPI_ISL_621224, EPI_ISL_621225, EPI_ISL_621226, EPI_ISL_621227, EPI_ISL_621228, EPI_ISL_621229, EPI_ISL_621230, EPI_ISL_621231, EPI_ISL_621232, EPI_ISL_621233, EPI_ISL_621234, EPI_ISL_621235,
EPI_ISL_621236, EPI_ISL_621237, EPI_ISL_621238, EPI_ISL_621239, EPI_ISL_621240, EPI_ISL_621241, EPI_ISL_621242, EPI_ISL_621243, EPI_ISL_621244, EPI_ISL_621245, EPI_ISL_621246, EPI_ISL_621247, EPI_ISL_621248, EPI_ISL_621249, EPI_ISL_621250, EPI_ISL_621251, EPI_ISL_621252, EPI_ISL_621253,
EPI_ISL_621254, EPI_ISL_621255, EPI_ISL_621256, EPI_ISL_621257, EPI_ISL_621258, EPI_ISL_621259, EPI_ISL_621260, EPI_ISL_621261, EPI_ISL_621262, EPI_ISL_621263, EPI_ISL_621264, EPI_ISL_621265, EPI_ISL_621266, EPI_ISL_621267, EPI_ISL_621268, EPI_ISL_621269, EPI_ISL_621270, EPI_ISL_621271,
EPI_ISL_621272, EPI_ISL_621273, EPI_ISL_621274, EPI_ISL_621275, EPI_ISL_621276, EPI_ISL_621277, EPI_ISL_621278, EPI_ISL_621279, EPI_ISL_621280, EPI_ISL_621281, EPI_ISL_621282, EPI_ISL_621283, EPI_ISL_621284, EPI_ISL_621287, EPI_ISL_621288, EPI_ISL_621289, EPI_ISL_621290, EPI_ISL_621292,
EPI_ISL_621293, EPI_ISL_621294, EPI_ISL_621295, EPI_ISL_621296, EPI_ISL_621297, EPI_ISL_621298, EPI_ISL_621299, EPI_ISL_621300, EPI_ISL_621301, EPI_ISL_621302, EPI_ISL_621303, EPI_ISL_621304, EPI_ISL_621305, EPI_ISL_621306, EPI_ISL_621307, EPI_ISL_621308, EPI_ISL_621309, EPI_ISL_621310,
EPI_ISL_621312, EPI_ISL_621313, EPI_ISL_621314, EPI_ISL_621315, EPI_ISL_621316, EPI_ISL_621317, EPI_ISL_621318, EPI_ISL_621319, EPI_ISL_621320, EPI_ISL_621321, EPI_ISL_621322, EPI_ISL_621323, EPI_ISL_621324, EPI_ISL_621325, EPI_ISL_621326, EPI_ISL_621327, EPI_ISL_621328, EPI_ISL_621329,
EPI_ISL_621330, EPI_ISL_621331, EPI_ISL_621332, EPI_ISL_621333, EPI_ISL_621334, EPI_ISL_621335, EPI_ISL_621336, EPI_ISL_621337, EPI_ISL_621338, EPI_ISL_621339, EPI_ISL_621340, EPI_ISL_621341, EPI_ISL_621342, EPI_ISL_621343, EPI_ISL_621344, EPI_ISL_621345, EPI_ISL_621346, EPI_ISL_621347,
EPI_ISL_621348, EPI_ISL_621350, EPI_ISL_621351, EPI_ISL_621352, EPI_ISL_621353, EPI_ISL_621354, EPI_ISL_621355, EPI_ISL_621356, EPI_ISL_621357, EPI_ISL_621358, EPI_ISL_621359, EPI_ISL_621360, EPI_ISL_621361, EPI_ISL_621362, EPI_ISL_621363, EPI_ISL_621364, EPI_ISL_621365, EPI_ISL_621366,
EPI_ISL_621367, EPI_ISL_621369, EPI_ISL_621370, EPI_ISL_621373, EPI_ISL_621374, EPI_ISL_621375, EPI_ISL_621376, EPI_ISL_621377, EPI_ISL_621378, EPI_ISL_621379, EPI_ISL_621380, EPI_ISL_621381, EPI_ISL_621383, EPI_ISL_621384, EPI_ISL_621385, EPI_ISL_621386, EPI_ISL_621387, EPI_ISL_621388,
EPI_ISL_621389, EPI_ISL_621390, EPI_ISL_621391, EPI_ISL_621392, EPI_ISL_621393, EPI_ISL_621394, EPI_ISL_621395, EPI_ISL_621396, EPI_ISL_621397, EPI_ISL_621398, EPI_ISL_621399, EPI_ISL_621400, EPI_ISL_621401, EPI_ISL_621402, EPI_ISL_621403, EPI_ISL_621404, EPI_ISL_621405, EPI_ISL_621406,
EPI_ISL_621407, EPI_ISL_621408, EPI_ISL_621409, EPI_ISL_621411, EPI_ISL_621412, EPI_ISL_621413, EPI_ISL_621414, EPI_ISL_621416, EPI_ISL_621417, EPI_ISL_621418, EPI_ISL_621419, EPI_ISL_621420, EPI_ISL_621422, EPI_ISL_621423, EPI_ISL_621424, EPI_ISL_621426, EPI_ISL_621428, EPI_ISL_621429,
EPI_ISL_621430, EPI_ISL_621431, EPI_ISL_621432, EPI_ISL_621433, EPI_ISL_621434, EPI_ISL_621435, EPI_ISL_621436, EPI_ISL_621438, EPI_ISL_621439, EPI_ISL_621440, EPI_ISL_621441, EPI_ISL_621442, EPI_ISL_621443, EPI_ISL_621444, EPI_ISL_621445, EPI_ISL_621446, EPI_ISL_621447, EPI_ISL_621448,
EPI_ISL_621449, EPI_ISL_621450, EPI_ISL_621451, EPI_ISL_621452, EPI_ISL_621453, EPI_ISL_621455, EPI_ISL_621456, EPI_ISL_621457, EPI_ISL_621458, EPI_ISL_621459, EPI_ISL_621460, EPI_ISL_621461, EPI_ISL_621462, EPI_ISL_621463, EPI_ISL_621464, EPI_ISL_621465, EPI_ISL_621466, EPI_ISL_621468,
EPI_ISL_621469, EPI_ISL_621470, EPI_ISL_621471, EPI_ISL_621472, EPI_ISL_621473, EPI_ISL_621474, EPI_ISL_621475, EPI_ISL_621476, EPI_ISL_621477, EPI_ISL_621478, EPI_ISL_621479, EPI_ISL_621480, EPI_ISL_621481, EPI_ISL_621482, EPI_ISL_621483, EPI_ISL_621484, EPI_ISL_621485, EPI_ISL_621486,
EPI_ISL_621487, EPI_ISL_621489, EPI_ISL_621490, EPI_ISL_621491, EPI_ISL_621492, EPI_ISL_621493, EPI_ISL_621494, EPI_ISL_621495, EPI_ISL_621496, EPI_ISL_621497, EPI_ISL_621498, EPI_ISL_621499, EPI_ISL_621500, EPI_ISL_621501, EPI_ISL_621502, EPI_ISL_621503, EPI_ISL_621504, EPI_ISL_621505,
EPI_ISL_621506, EPI_ISL_621507, EPI_ISL_621508, EPI_ISL_621509, EPI_ISL_621510, EPI_ISL_621511, EPI_ISL_621512, EPI_ISL_621513, EPI_ISL_621514, EPI_ISL_621515, EPI_ISL_621516, EPI_ISL_621517, EPI_ISL_621518, EPI_ISL_621519, EPI_ISL_621520, EPI_ISL_621521, EPI_ISL_621522, EPI_ISL_621523,
EPI_ISL_621524, EPI_ISL_621525, EPI_ISL_621526, EPI_ISL_621527, EPI_ISL_621528, EPI_ISL_621529, EPI_ISL_621530, EPI_ISL_621531, EPI_ISL_621532, EPI_ISL_621535, EPI_ISL_621536, EPI_ISL_621537, EPI_ISL_621538, EPI_ISL_621539, EPI_ISL_621540, EPI_ISL_621541, EPI_ISL_621542, EPI_ISL_621543,
EPI_ISL_621545, EPI_ISL_621546, EPI_ISL_621547, EPI_ISL_621548, EPI_ISL_621549, EPI_ISL_621550, EPI_ISL_621551, EPI_ISL_621552, EPI_ISL_621553, EPI_ISL_621554, EPI_ISL_621555, EPI_ISL_621556, EPI_ISL_621557, EPI_ISL_621558, EPI_ISL_621559, EPI_ISL_621560, EPI_ISL_621562, EPI_ISL_621563,
EPI_ISL_621564, EPI_ISL_621565, EPI_ISL_621566, EPI_ISL_621567, EPI_ISL_621568, EPI_ISL_621569, EPI_ISL_621570, EPI_ISL_621571, EPI_ISL_621572, EPI_ISL_621573, EPI_ISL_621574, EPI_ISL_621575, EPI_ISL_621576, EPI_ISL_621577, EPI_ISL_621578, EPI_ISL_621579, EPI_ISL_621580, EPI_ISL_621581,
EPI_ISL_621582, EPI_ISL_621583, EPI_ISL_621584, EPI_ISL_621585, EPI_ISL_621586, EPI_ISL_621587, EPI_ISL_621588, EPI_ISL_621589, EPI_ISL_621590, EPI_ISL_621591, EPI_ISL_621594, EPI_ISL_621595, EPI_ISL_621597, EPI_ISL_621598, EPI_ISL_621599, EPI_ISL_621600, EPI_ISL_621601, EPI_ISL_621602,
EPI_ISL_621603, EPI_ISL_621604, EPI_ISL_621605, EPI_ISL_621608, EPI_ISL_621609, EPI_ISL_621610, EPI_ISL_621613, EPI_ISL_621614, EPI_ISL_621616, EPI_ISL_621617, EPI_ISL_621618, EPI_ISL_621619, EPI_ISL_621620, EPI_ISL_621621, EPI_ISL_621623, EPI_ISL_621624, EPI_ISL_621625, EPI_ISL_621626,
EPI_ISL_621627, EPI_ISL_621628, EPI_ISL_621629, EPI_ISL_621631, EPI_ISL_621632, EPI_ISL_621633, EPI_ISL_621634, EPI_ISL_621635, EPI_ISL_621636, EPI_ISL_621638, EPI_ISL_621639, EPI_ISL_621640, EPI_ISL_621641, EPI_ISL_621642, EPI_ISL_621644, EPI_ISL_621645, EPI_ISL_621646, EPI_ISL_621648,
EPI_ISL_621649, EPI_ISL_621650, EPI_ISL_621651, EPI_ISL_621652, EPI_ISL_621653, EPI_ISL_621654, EPI_ISL_621655, EPI_ISL_621656, EPI_ISL_621657, EPI_ISL_621658, EPI_ISL_621659, EPI_ISL_621661, EPI_ISL_621662, EPI_ISL_621664, EPI_ISL_621665, EPI_ISL_621667, EPI_ISL_621668, EPI_ISL_621669,
EPI_ISL_621670, EPI_ISL_621671, EPI_ISL_621672, EPI_ISL_621673, EPI_ISL_621674, EPI_ISL_621675, EPI_ISL_621677, EPI_ISL_621678, EPI_ISL_621679, EPI_ISL_621680, EPI_ISL_621681, EPI_ISL_621682, EPI_ISL_621683, EPI_ISL_621684, EPI_ISL_621686, EPI_ISL_621687, EPI_ISL_621688, EPI_ISL_621689,
EPI_ISL_621690, EPI_ISL_621691, EPI_ISL_621692, EPI_ISL_621693, EPI_ISL_621694, EPI_ISL_621695, EPI_ISL_621697, EPI_ISL_621698, EPI_ISL_621699, EPI_ISL_621700, EPI_ISL_621701, EPI_ISL_621702, EPI_ISL_621703, EPI_ISL_621704, EPI_ISL_621705, EPI_ISL_621706, EPI_ISL_621707, EPI_ISL_621709,
EPI_ISL_621710, EPI_ISL_621711, EPI_ISL_621712, EPI_ISL_621713, EPI_ISL_621714, EPI_ISL_621715, EPI_ISL_621719, EPI_ISL_621720, EPI_ISL_621721, EPI_ISL_621722, EPI_ISL_621724, EPI_ISL_621725, EPI_ISL_621726, EPI_ISL_621727, EPI_ISL_621728, EPI_ISL_621729, EPI_ISL_621730, EPI_ISL_621731,
EPI_ISL_621732, EPI_ISL_621733, EPI_ISL_621734, EPI_ISL_621735, EPI_ISL_621737, EPI_ISL_621738, EPI_ISL_621739, EPI_ISL_621740, EPI_ISL_621741, EPI_ISL_621742, EPI_ISL_621743, EPI_ISL_621744, EPI_ISL_621747, EPI_ISL_621748, EPI_ISL_621749, EPI_ISL_621750, EPI_ISL_621751, EPI_ISL_621752,
EPI_ISL_621753, EPI_ISL_621755, EPI_ISL_621756, EPI_ISL_621757, EPI_ISL_621758, EPI_ISL_621759, EPI_ISL_621760, EPI_ISL_621761, EPI_ISL_621762, EPI_ISL_621763, EPI_ISL_621764, EPI_ISL_621765, EPI_ISL_621766, EPI_ISL_621767, EPI_ISL_621768, EPI_ISL_621769, EPI_ISL_621770, EPI_ISL_621771,
EPI_ISL_621772, EPI_ISL_621773, EPI_ISL_621774, EPI_ISL_621775, EPI_ISL_621776, EPI_ISL_621777, EPI_ISL_621778, EPI_ISL_621779, EPI_ISL_621780, EPI_ISL_621781, EPI_ISL_621782, EPI_ISL_621783, EPI_ISL_621784, EPI_ISL_621785, EPI_ISL_621786, EPI_ISL_621787, EPI_ISL_621788, EPI_ISL_621789,
EPI_ISL_621790, EPI_ISL_621791, EPI_ISL_621792, EPI_ISL_621793, EPI_ISL_621794, EPI_ISL_621795, EPI_ISL_621796, EPI_ISL_621797, EPI_ISL_621798, EPI_ISL_621799, EPI_ISL_621800, EPI_ISL_621801, EPI_ISL_621802, EPI_ISL_621803, EPI_ISL_621804, EPI_ISL_621805, EPI_ISL_621806, EPI_ISL_621807,
EPI_ISL_621808, EPI_ISL_621809, EPI_ISL_621810, EPI_ISL_621811, EPI_ISL_621812, EPI_ISL_621814, EPI_ISL_621816, EPI_ISL_621817, EPI_ISL_621818, EPI_ISL_621819, EPI_ISL_621820, EPI_ISL_621821, EPI_ISL_621822, EPI_ISL_621823, EPI_ISL_621824, EPI_ISL_621825, EPI_ISL_621826, EPI_ISL_621827,
EPI_ISL_621828, EPI_ISL_621829, EPI_ISL_621830, EPI_ISL_621832, EPI_ISL_621833, EPI_ISL_621834, EPI_ISL_621835, EPI_ISL_621836, EPI_ISL_621837, EPI_ISL_621838, EPI_ISL_621839, EPI_ISL_621840, EPI_ISL_621841, EPI_ISL_621842, EPI_ISL_621843, EPI_ISL_621844, EPI_ISL_621845, EPI_ISL_621846,
EPI_ISL_621847, EPI_ISL_621848, EPI_ISL_621849, EPI_ISL_621850, EPI_ISL_621851, EPI_ISL_621852, EPI_ISL_621855, EPI_ISL_621857, EPI_ISL_621858, EPI_ISL_621859, EPI_ISL_621860, EPI_ISL_621861, EPI_ISL_621862, EPI_ISL_621863, EPI_ISL_621864, EPI_ISL_621865, EPI_ISL_621866, EPI_ISL_621867,
EPI_ISL_621868, EPI_ISL_621869, EPI_ISL_621870, EPI_ISL_621871, EPI_ISL_621872, EPI_ISL_621873, EPI_ISL_621874, EPI_ISL_621875, EPI_ISL_621876, EPI_ISL_621877, EPI_ISL_621878, EPI_ISL_621879, EPI_ISL_621880, EPI_ISL_621882, EPI_ISL_621884, EPI_ISL_621885, EPI_ISL_621886, EPI_ISL_621887,
EPI_ISL_621888, EPI_ISL_621890, EPI_ISL_621891, EPI_ISL_621892, EPI_ISL_621893, EPI_ISL_621894, EPI_ISL_621895, EPI_ISL_621896, EPI_ISL_621897, EPI_ISL_621899, EPI_ISL_621900, EPI_ISL_621901, EPI_ISL_621902, EPI_ISL_621903, EPI_ISL_621904, EPI_ISL_621905, EPI_ISL_621906, EPI_ISL_621907,
EPI_ISL_621908, EPI_ISL_621909, EPI_ISL_621910, EPI_ISL_621911, EPI_ISL_621912, EPI_ISL_621913, EPI_ISL_621914, EPI_ISL_621915, EPI_ISL_621916, EPI_ISL_621918, EPI_ISL_621919, EPI_ISL_621920, EPI_ISL_621921, EPI_ISL_621922, EPI_ISL_621923, EPI_ISL_621924, EPI_ISL_621925, EPI_ISL_621926,
EPI_ISL_621927, EPI_ISL_621928, EPI_ISL_621932, EPI_ISL_621933, EPI_ISL_621935, EPI_ISL_621936, EPI_ISL_621941, EPI_ISL_621942, EPI_ISL_621944, EPI_ISL_621945, EPI_ISL_621947, EPI_ISL_621948, EPI_ISL_621949, EPI_ISL_621950, EPI_ISL_621951, EPI_ISL_621952, EPI_ISL_621953, EPI_ISL_621954,
EPI_ISL_621955, EPI_ISL_621956, EPI_ISL_621959, EPI_ISL_621960, EPI_ISL_621961, EPI_ISL_621962, EPI_ISL_621963, EPI_ISL_621964, EPI_ISL_621965, EPI_ISL_621966, EPI_ISL_621967, EPI_ISL_621969, EPI_ISL_621970, EPI_ISL_621971, EPI_ISL_621972, EPI_ISL_621974, EPI_ISL_621975, EPI_ISL_621976,
EPI_ISL_621977, EPI_ISL_621978, EPI_ISL_621979, EPI_ISL_621980, EPI_ISL_621982, EPI_ISL_621983, EPI_ISL_621984, EPI_ISL_621985, EPI_ISL_621986, EPI_ISL_621987, EPI_ISL_621988, EPI_ISL_621989, EPI_ISL_621990, EPI_ISL_621992, EPI_ISL_621993, EPI_ISL_621995, EPI_ISL_621996, EPI_ISL_621997,
EPI_ISL_621998, EPI_ISL_621999, EPI_ISL_622000, EPI_ISL_622001, EPI_ISL_622003, EPI_ISL_622004, EPI_ISL_622005, EPI_ISL_622006, EPI_ISL_622007, EPI_ISL_622008, EPI_ISL_622010, EPI_ISL_622011, EPI_ISL_622012, EPI_ISL_622014, EPI_ISL_622015, EPI_ISL_622016, EPI_ISL_622018, EPI_ISL_622019,
EPI_ISL_622020, EPI_ISL_622021, EPI_ISL_622022, EPI_ISL_622024, EPI_ISL_622025, EPI_ISL_622026, EPI_ISL_622027, EPI_ISL_622028, EPI_ISL_622030, EPI_ISL_622031, EPI_ISL_622032, EPI_ISL_622033, EPI_ISL_622034, EPI_ISL_622035, EPI_ISL_622036, EPI_ISL_622037, EPI_ISL_622038, EPI_ISL_622039,
EPI_ISL_622040, EPI_ISL_622041, EPI_ISL_622042, EPI_ISL_622043, EPI_ISL_622044, EPI_ISL_622045, EPI_ISL_622046, EPI_ISL_622047, EPI_ISL_622048, EPI_ISL_622049, EPI_ISL_622050, EPI_ISL_622051, EPI_ISL_622052, EPI_ISL_622053, EPI_ISL_622054, EPI_ISL_622055, EPI_ISL_622056, EPI_ISL_622059,
EPI_ISL_622060, EPI_ISL_622062, EPI_ISL_622063, EPI_ISL_622064, EPI_ISL_622066, EPI_ISL_622067, EPI_ISL_622068, EPI_ISL_622070, EPI_ISL_622071, EPI_ISL_622072, EPI_ISL_622073, EPI_ISL_622074, EPI_ISL_622075, EPI_ISL_622077, EPI_ISL_622078, EPI_ISL_622079, EPI_ISL_622080, EPI_ISL_622081,
EPI_ISL_622082, EPI_ISL_622083, EPI_ISL_622084, EPI_ISL_622085, EPI_ISL_622086, EPI_ISL_622087, EPI_ISL_622088, EPI_ISL_622089, EPI_ISL_622090, EPI_ISL_622091, EPI_ISL_622092, EPI_ISL_622093, EPI_ISL_622094, EPI_ISL_622095, EPI_ISL_622096, EPI_ISL_622097, EPI_ISL_622098, EPI_ISL_622099,
EPI_ISL_622100, EPI_ISL_622101, EPI_ISL_622103, EPI_ISL_622104, EPI_ISL_622105, EPI_ISL_622106, EPI_ISL_622107, EPI_ISL_622108, EPI_ISL_622109, EPI_ISL_622110, EPI_ISL_622111, EPI_ISL_622112, EPI_ISL_622113, EPI_ISL_622114, EPI_ISL_622115, EPI_ISL_622116, EPI_ISL_622117, EPI_ISL_622118,
EPI_ISL_622119, EPI_ISL_622120, EPI_ISL_622121, EPI_ISL_622122, EPI_ISL_622123, EPI_ISL_622124, EPI_ISL_622125, EPI_ISL_622126, EPI_ISL_622127, EPI_ISL_622129, EPI_ISL_622130, EPI_ISL_622132, EPI_ISL_622133, EPI_ISL_622134, EPI_ISL_622135, EPI_ISL_622136, EPI_ISL_622137, EPI_ISL_622138,
EPI_ISL_622139, EPI_ISL_622140, EPI_ISL_622141, EPI_ISL_622142, EPI_ISL_622143, EPI_ISL_622144, EPI_ISL_622145, EPI_ISL_622146, EPI_ISL_622147, EPI_ISL_622148, EPI_ISL_622149, EPI_ISL_622150, EPI_ISL_622151, EPI_ISL_622152, EPI_ISL_622153, EPI_ISL_622154, EPI_ISL_622155, EPI_ISL_622156,
EPI_ISL_622157, EPI_ISL_622158, EPI_ISL_622159, EPI_ISL_622161, EPI_ISL_622162, EPI_ISL_622163, EPI_ISL_622164, EPI_ISL_622165, EPI_ISL_622166, EPI_ISL_622167, EPI_ISL_622168, EPI_ISL_622169, EPI_ISL_622170, EPI_ISL_622171, EPI_ISL_622172, EPI_ISL_622173, EPI_ISL_622174, EPI_ISL_622177,

EPI_ISL_622178, EPI_ISL_622179, EPI_ISL_622180, EPI_ISL_622181, EPI_ISL_622182, EPI_ISL_622183, EPI_ISL_622185, EPI_ISL_622186, EPI_ISL_622187, EPI_ISL_622188, EPI_ISL_622189, EPI_ISL_622190, EPI_ISL_622191, EPI_ISL_622192, EPI_ISL_622193, EPI_ISL_622194, EPI_ISL_622196, EPI_ISL_622197, EPI_ISL_622198, EPI_ISL_622199, EPI_ISL_622200, EPI_ISL_622201, EPI_ISL_622202, EPI_ISL_622204, EPI_ISL_622205, EPI_ISL_622206, EPI_ISL_622207, EPI_ISL_622208, EPI_ISL_622209, EPI_ISL_622210, EPI_ISL_622211, EPI_ISL_622212, EPI_ISL_622213, EPI_ISL_622214, EPI_ISL_622215, EPI_ISL_622217, EPI_ISL_622218, EPI_ISL_622220, EPI_ISL_622221, EPI_ISL_622222, EPI_ISL_622223, EPI_ISL_622224, EPI_ISL_622225, EPI_ISL_622226, EPI_ISL_622229, EPI_ISL_622231, EPI_ISL_622232, EPI_ISL_622233, EPI_ISL_622234, EPI_ISL_622235, EPI_ISL_622236, EPI_ISL_622237, EPI_ISL_622238, EPI_ISL_622239, EPI_ISL_622240, EPI_ISL_622241, EPI_ISL_622242, EPI_ISL_622243, EPI_ISL_622244, EPI_ISL_622245, EPI_ISL_622246, EPI_ISL_622247, EPI_ISL_622248, EPI_ISL_622249, EPI_ISL_622250, EPI_ISL_622251, EPI_ISL_622252, EPI_ISL_622253, EPI_ISL_622254, EPI_ISL_622255, EPI_ISL_622257, EPI_ISL_622258, EPI_ISL_622259, EPI_ISL_622260, EPI_ISL_622262, EPI_ISL_622263, EPI_ISL_622264, EPI_ISL_622265, EPI_ISL_622266, EPI_ISL_622267, EPI_ISL_622268, EPI_ISL_622269, EPI_ISL_622270, EPI_ISL_622271, EPI_ISL_622272, EPI_ISL_622273, EPI_ISL_622274, EPI_ISL_622275, EPI_ISL_622276, EPI_ISL_622277, EPI_ISL_622278, EPI_ISL_622279, EPI_ISL_622280, EPI_ISL_622281, EPI_ISL_622282, EPI_ISL_622283, EPI_ISL_622284, EPI_ISL_622285, EPI_ISL_622286, EPI_ISL_622287, EPI_ISL_622288, EPI_ISL_622289, EPI_ISL_622290, EPI_ISL_622291, EPI_ISL_622292, EPI_ISL_622293, EPI_ISL_622294, EPI_ISL_622295, EPI_ISL_622296, EPI_ISL_622297, EPI_ISL_622298, EPI_ISL_622299, EPI_ISL_622300, EPI_ISL_622301, EPI_ISL_622302, EPI_ISL_622303, EPI_ISL_622304, EPI_ISL_622305, EPI_ISL_622306, EPI_ISL_622307, EPI_ISL_622308, EPI_ISL_622309, EPI_ISL_622310, EPI_ISL_622311, EPI_ISL_622312, EPI_ISL_622313, EPI_ISL_622314, EPI_ISL_622315, EPI_ISL_622316, EPI_ISL_622317, EPI_ISL_622320, EPI_ISL_622321, EPI_ISL_622322, EPI_ISL_622323, EPI_ISL_622324, EPI_ISL_622325, EPI_ISL_622326, EPI_ISL_622327, EPI_ISL_622328, EPI_ISL_622329, EPI_ISL_622330, EPI_ISL_622331, EPI_ISL_622332, EPI_ISL_622333, EPI_ISL_622334, EPI_ISL_622335, EPI_ISL_622336, EPI_ISL_622337, EPI_ISL_622338, EPI_ISL_622339, EPI_ISL_622340, EPI_ISL_622341, EPI_ISL_622343, EPI_ISL_622344, EPI_ISL_622345, EPI_ISL_622346, EPI_ISL_622347, EPI_ISL_622348, EPI_ISL_622349, EPI_ISL_622350, EPI_ISL_622351, EPI_ISL_622352, EPI_ISL_622353, EPI_ISL_622354, EPI_ISL_622356, EPI_ISL_622357, EPI_ISL_622358, EPI_ISL_622360, EPI_ISL_622361, EPI_ISL_622362, EPI_ISL_622364, EPI_ISL_622367, EPI_ISL_622368, EPI_ISL_622369, EPI_ISL_622370, EPI_ISL_622371, EPI_ISL_622373, EPI_ISL_622374, EPI_ISL_622376, EPI_ISL_622377, EPI_ISL_622378, EPI_ISL_622379, EPI_ISL_622380, EPI_ISL_622381, EPI_ISL_622383, EPI_ISL_622384, EPI_ISL_622385, EPI_ISL_622386, EPI_ISL_622387, EPI_ISL_622388, EPI_ISL_622389, EPI_ISL_622390, EPI_ISL_622391, EPI_ISL_622392, EPI_ISL_622393, EPI_ISL_622394, EPI_ISL_622395, EPI_ISL_622396, EPI_ISL_622398, EPI_ISL_622400, EPI_ISL_622401, EPI_ISL_622402, EPI_ISL_622403, EPI_ISL_622407, EPI_ISL_622408, EPI_ISL_622410, EPI_ISL_622411, EPI_ISL_622412, EPI_ISL_622413, EPI_ISL_622414, EPI_ISL_622415, EPI_ISL_622416, EPI_ISL_622418, EPI_ISL_622419, EPI_ISL_622420, EPI_ISL_622421, EPI_ISL_622422, EPI_ISL_622423, EPI_ISL_622424, EPI_ISL_622425, EPI_ISL_622426, EPI_ISL_622427, EPI_ISL_622428, EPI_ISL_622429, EPI_ISL_622430, EPI_ISL_622431, EPI_ISL_622432, EPI_ISL_622433, EPI_ISL_622434, EPI_ISL_622435, EPI_ISL_622436, EPI_ISL_622437, EPI_ISL_622438, EPI_ISL_622439, EPI_ISL_622440, EPI_ISL_622441, EPI_ISL_622442, EPI_ISL_622444, EPI_ISL_622445, EPI_ISL_622446, EPI_ISL_622447, EPI_ISL_622448, EPI_ISL_622450, EPI_ISL_622451, EPI_ISL_622452, EPI_ISL_622453, EPI_ISL_622454, EPI_ISL_622455, EPI_ISL_622456, EPI_ISL_622457, EPI_ISL_622458, EPI_ISL_622459, EPI_ISL_622460, EPI_ISL_622462, EPI_ISL_622463, EPI_ISL_622464, EPI_ISL_622465, EPI_ISL_622467, EPI_ISL_622468, EPI_ISL_622469, EPI_ISL_622470, EPI_ISL_622471, EPI_ISL_622472, EPI_ISL_622473, EPI_ISL_622474, EPI_ISL_622475, EPI_ISL_622476, EPI_ISL_622477, EPI_ISL_622478, EPI_ISL_622479, EPI_ISL_622480, EPI_ISL_622481, EPI_ISL_622482, EPI_ISL_622483, EPI_ISL_622484, EPI_ISL_622485, EPI_ISL_622486, EPI_ISL_622487, EPI_ISL_622488, EPI_ISL_622491, EPI_ISL_622492, EPI_ISL_622493, EPI_ISL_622494, EPI_ISL_622495, EPI_ISL_622496, EPI_ISL_622498, EPI_ISL_622499, EPI_ISL_622500, EPI_ISL_622501, EPI_ISL_622502, EPI_ISL_622503, EPI_ISL_622504, EPI_ISL_622505, EPI_ISL_622506, EPI_ISL_622507, EPI_ISL_622508, EPI_ISL_622509, EPI_ISL_622510, EPI_ISL_622511, EPI_ISL_622512, EPI_ISL_622513, EPI_ISL_622514, EPI_ISL_622516, EPI_ISL_622517, EPI_ISL_622518, EPI_ISL_622519, EPI_ISL_622520, EPI_ISL_622521, EPI_ISL_622522, EPI_ISL_622523, EPI_ISL_622524, EPI_ISL_622525, EPI_ISL_622526, EPI_ISL_622527, EPI_ISL_622528, EPI_ISL_622529, EPI_ISL_622530, EPI_ISL_622531, EPI_ISL_622532, EPI_ISL_622533, EPI_ISL_622534, EPI_ISL_622535, EPI_ISL_622536, EPI_ISL_622537, EPI_ISL_622539, EPI_ISL_622540, EPI_ISL_622541, EPI_ISL_622542, EPI_ISL_622543, EPI_ISL_622544, EPI_ISL_622545, EPI_ISL_622546, EPI_ISL_622549, EPI_ISL_622550, EPI_ISL_622551, EPI_ISL_622552, EPI_ISL_622553, EPI_ISL_622554, EPI_ISL_622555, EPI_ISL_622556, EPI_ISL_622557, EPI_ISL_622558, EPI_ISL_622559, EPI_ISL_622560, EPI_ISL_622561, EPI_ISL_622562, EPI_ISL_622563, EPI_ISL_622564, EPI_ISL_622565, EPI_ISL_622566, EPI_ISL_622567, EPI_ISL_622568, EPI_ISL_622569, EPI_ISL_622570, EPI_ISL_622571, EPI_ISL_622572, EPI_ISL_622573, EPI_ISL_622574, EPI_ISL_622575, EPI_ISL_622576, EPI_ISL_622577, EPI_ISL_622578, EPI_ISL_622579, EPI_ISL_622580, EPI_ISL_622581, EPI_ISL_622583, EPI_ISL_622584, EPI_ISL_622585, EPI_ISL_622586, EPI_ISL_622587, EPI_ISL_622588, EPI_ISL_622589, EPI_ISL_622590, EPI_ISL_622591, EPI_ISL_622592, EPI_ISL_622593, EPI_ISL_622594, EPI_ISL_622595, EPI_ISL_622596, EPI_ISL_622597, EPI_ISL_622599, EPI_ISL_622600, EPI_ISL_622601, EPI_ISL_622602, EPI_ISL_622603, EPI_ISL_622604, EPI_ISL_622605, EPI_ISL_622606, EPI_ISL_622607, EPI_ISL_622608, EPI_ISL_622609, EPI_ISL_622610, EPI_ISL_622612, EPI_ISL_622613, EPI_ISL_622614, EPI_ISL_622615, EPI_ISL_622616, EPI_ISL_622617, EPI_ISL_622618, EPI_ISL_622619, EPI_ISL_622620, EPI_ISL_622621, EPI_ISL_622622, EPI_ISL_622623, EPI_ISL_622624, EPI_ISL_622625, EPI_ISL_622626, EPI_ISL_622627, EPI_ISL_622628, EPI_ISL_622629, EPI_ISL_622630, EPI_ISL_622631, EPI_ISL_622633, EPI_ISL_622634, EPI_ISL_622635, EPI_ISL_622636, EPI_ISL_622637, EPI_ISL_622638, EPI_ISL_622639, EPI_ISL_622640, EPI_ISL_622641, EPI_ISL_622642, EPI_ISL_622644, EPI_ISL_622645, EPI_ISL_622646, EPI_ISL_622647, EPI_ISL_622648, EPI_ISL_622650, EPI_ISL_622652, EPI_ISL_622653, EPI_ISL_622654, EPI_ISL_622655, EPI_ISL_622656, EPI_ISL_622657, EPI_ISL_622658, EPI_ISL_622659, EPI_ISL_622660, EPI_ISL_622661, EPI_ISL_622662, EPI_ISL_622663, EPI_ISL_622664, EPI_ISL_622665, EPI_ISL_622666, EPI_ISL_622667, EPI_ISL_622668, EPI_ISL_622671, EPI_ISL_622673, EPI_ISL_622674, EPI_ISL_622675, EPI_ISL_622677, EPI_ISL_622678, EPI_ISL_622679, EPI_ISL_622680, EPI_ISL_622681, EPI_ISL_622682, EPI_ISL_622683, EPI_ISL_622684, EPI_ISL_622685, EPI_ISL_622686, EPI_ISL_622687, EPI_ISL_622688, EPI_ISL_622689, EPI_ISL_622690, EPI_ISL_622691, EPI_ISL_622692, EPI_ISL_622693, EPI_ISL_622695, EPI_ISL_622696, EPI_ISL_622697, EPI_ISL_622698, EPI_ISL_622699, EPI_ISL_622700, EPI_ISL_622702, EPI_ISL_622703, EPI_ISL_622704, EPI_ISL_622705, EPI_ISL_622706, EPI_ISL_622707, EPI_ISL_622708, EPI_ISL_622709, EPI_ISL_622710, EPI_ISL_622711, EPI_ISL_622712, EPI_ISL_622714, EPI_ISL_622715, EPI_ISL_622716, EPI_ISL_622717, EPI_ISL_622718, EPI_ISL_622719, EPI_ISL_622721, EPI_ISL_622722, EPI_ISL_622723, EPI_ISL_622724, EPI_ISL_622725, EPI_ISL_622726, EPI_ISL_622727, EPI_ISL_622728, EPI_ISL_622729, EPI_ISL_622730, EPI_ISL_622731, EPI_ISL_622732, EPI_ISL_622733, EPI_ISL_622734, EPI_ISL_622735, EPI_ISL_622736, EPI_ISL_622737, EPI_ISL_622738, EPI_ISL_622739, EPI_ISL_622741, EPI_ISL_622742, EPI_ISL_622743, EPI_ISL_622744, EPI_ISL_622745, EPI_ISL_622746, EPI_ISL_622747, EPI_ISL_622748, EPI_ISL_622749, EPI_ISL_622750, EPI_ISL_622751, EPI_ISL_622752, EPI_ISL_622753, EPI_ISL_622754, EPI_ISL_622755, EPI_ISL_622756, EPI_ISL_622757, EPI_ISL_622758, EPI_ISL_622759, EPI_ISL_622760			
see above	Department of Virus and Microbiological Special Diagnostics, Statens Serum Institut, Denmark	Albertsen lab, Department of Chemistry and Bioscience, Aalborg University, Denmark	Danish Covid-19 Genome Consortia
EPI_ISL_622762, EPI_ISL_622763, EPI_ISL_622764, EPI_ISL_622765, EPI_ISL_622766	SA Pathology	SA Pathology	Lex Leong, Julien Soubrier, Chuan Kok Lim, Song Gao, Mark Turra, Karin Kassahn, Ivan Bastian, Geoff Higgins
EPI_ISL_622787, EPI_ISL_622792	Middlemore Hospital	Institute of Environmental Science and Research (ESR)	Xiaoyun Ren, Matt Storey, Nikki Freed, Muhammad Faisal, Jing Wang, Hermes Perez, Anja Werno, Antje van der Linden, Arlo Upton, Chris Mansell, David Hammer, Dragana Drinkovic, Gary McAuliffe, Hana Sofia Andersson, James Ussher, Jill Sherwood, Josh Freeman, Julia Howard, Juliet Elvy, Mary DeAlmeida, Matt Blakiston, Matthew Rogers, Max Bloomfield, Michael Addidle, Michelle Balm, Sally Roberts, Sarah Jefferies, Sharmini Muttaiyah, Susan Morpeth, Susan Taylor, Timothy Blackmore, Vani Sathyendran, Veronica Playle, Virginia Hope, Erasmus Smit, Lauren Jelly, Olin Silander, Joep de Ligt
EPI_ISL_622794, EPI_ISL_622795, EPI_ISL_622796, EPI_ISL_622797, EPI_ISL_622798, EPI_ISL_622799, EPI_ISL_622800, EPI_ISL_622801	Canterbury Health Laboratories	Institute of Environmental Science and Research (ESR)	Xiaoyun Ren, Matt Storey, Nikki Freed, Muhammad Faisal, Jing Wang, Hermes Perez, Anja Werno, Antje van der Linden, Arlo Upton, Chris Mansell, David Hammer, Dragana Drinkovic, Gary McAuliffe, Hana Sofia Andersson, James Ussher, Jill Sherwood, Josh Freeman, Julia Howard, Juliet Elvy, Mary DeAlmeida, Matt Blakiston, Matthew Rogers, Max Bloomfield, Michael Addidle, Michelle Balm, Sally Roberts, Sarah Jefferies, Sharmini Muttaiyah, Susan Morpeth, Susan Taylor, Timothy Blackmore, Vani Sathyendran, Veronica Playle, Virginia Hope, Erasmus Smit, Lauren Jelly, Olin Silander, Joep de Ligt
EPI_ISL_622802	Middlemore Hospital	Institute of Environmental Science and Research (ESR)	Xiaoyun Ren, Matt Storey, Nikki Freed, Muhammad Faisal, Jing Wang, Hermes Perez, Anja Werno, Antje van der Linden, Arlo Upton, Chris Mansell, David Hammer, Dragana Drinkovic, Gary McAuliffe, Hana Sofia Andersson, James Ussher, Jill Sherwood, Josh Freeman, Julia Howard, Juliet Elvy, Mary DeAlmeida, Matt Blakiston, Matthew Rogers, Max Bloomfield, Michael Addidle, Michelle Balm, Sally Roberts, Sarah Jefferies, Sharmini Muttaiyah, Susan Morpeth, Susan Taylor, Timothy Blackmore, Vani Sathyendran, Veronica Playle, Virginia Hope, Erasmus Smit, Lauren Jelly, Olin Silander, Joep de Ligt
EPI_ISL_622803	LabPLUS	Institute of Environmental Science and Research (ESR)	Xiaoyun Ren, Matt Storey, Nikki Freed, Muhammad Faisal, Jing Wang, Hermes Perez, Anja Werno, Antje van der Linden, Arlo Upton, Chris Mansell, David Hammer, Dragana Drinkovic, Gary McAuliffe, Hana Sofia Andersson, James Ussher, Jill Sherwood, Josh Freeman, Julia Howard, Juliet Elvy, Mary DeAlmeida, Matt Blakiston, Matthew Rogers, Max Bloomfield, Michael Addidle, Michelle Balm, Sally Roberts, Sarah Jefferies, Sharmini Muttaiyah, Susan Morpeth, Susan Taylor, Timothy Blackmore, Vani Sathyendran, Veronica Playle, Virginia Hope, Erasmus Smit, Lauren Jelly, Olin Silander, Joep de Ligt
EPI_ISL_622804, EPI_ISL_622805, EPI_ISL_622806	PathLab Bay of Plenty	Institute of Environmental Science and Research (ESR)	Xiaoyun Ren, Matt Storey, Nikki Freed, Muhammad Faisal, Jing Wang, Hermes Perez, Anja Werno, Antje van der Linden, Arlo Upton, Chris Mansell, David Hammer, Dragana Drinkovic, Gary McAuliffe, Hana Sofia Andersson, James Ussher, Jill Sherwood, Josh Freeman, Julia Howard, Juliet Elvy, Mary DeAlmeida, Matt Blakiston, Matthew Rogers, Max Bloomfield, Michael Addidle, Michelle Balm, Sally Roberts, Sarah Jefferies, Sharmini Muttaiyah, Susan Morpeth, Susan Taylor, Timothy Blackmore, Vani Sathyendran, Veronica Playle, Virginia Hope, Erasmus Smit, Lauren Jelly, Olin Silander, Joep de Ligt
EPI_ISL_622807	LabPLUS	Institute of Environmental Science and Research (ESR)	Xiaoyun Ren, Matt Storey, Nikki Freed, Muhammad Faisal, Jing Wang, Hermes Perez, Anja Werno, Antje van der Linden, Arlo Upton, Chris Mansell, David Hammer, Dragana Drinkovic, Gary McAuliffe, Hana Sofia Andersson, James Ussher, Jill Sherwood, Josh Freeman, Julia Howard, Juliet Elvy, Mary DeAlmeida, Matt Blakiston, Matthew Rogers, Max Bloomfield, Michael Addidle, Michelle Balm, Sally Roberts, Sarah Jefferies, Sharmini Muttaiyah, Susan Morpeth, Susan Taylor, Timothy Blackmore, Vani Sathyendran, Veronica Playle, Virginia Hope, Erasmus Smit, Lauren Jelly, Olin Silander, Joep de Ligt
EPI_ISL_622808, EPI_ISL_622809, EPI_ISL_622810, EPI_ISL_622811, EPI_ISL_622812, EPI_ISL_622813	Canterbury Health Laboratories	Institute of Environmental Science and Research (ESR)	Xiaoyun Ren, Matt Storey, Nikki Freed, Muhammad Faisal, Jing Wang, Hermes Perez, Anja Werno, Antje van der Linden, Arlo Upton, Chris Mansell, David Hammer, Dragana Drinkovic, Gary McAuliffe, Hana Sofia Andersson, James Ussher, Jill Sherwood, Josh Freeman, Julia Howard, Juliet Elvy, Mary DeAlmeida, Matt Blakiston, Matthew Rogers, Max Bloomfield, Michael Addidle, Michelle Balm, Sally Roberts, Sarah Jefferies, Sharmini Muttaiyah, Susan Morpeth, Susan Taylor, Timothy Blackmore, Vani Sathyendran, Veronica Playle, Virginia Hope, Erasmus Smit, Lauren Jelly, Olin Silander, Joep de Ligt
EPI_ISL_622814, EPI_ISL_622815, EPI_ISL_622816	LabPLUS	Institute of Environmental Science and Research (ESR)	Xiaoyun Ren, Matt Storey, Nikki Freed, Muhammad Faisal, Jing Wang, Hermes Perez, Anja Werno, Antje van der Linden, Arlo Upton, Chris Mansell, David Hammer, Dragana Drinkovic, Gary McAuliffe, Hana Sofia Andersson, James Ussher, Jill Sherwood, Josh Freeman, Julia Howard, Juliet Elvy, Mary DeAlmeida, Matt Blakiston, Matthew Rogers, Max Bloomfield, Michael Addidle, Michelle Balm, Sally Roberts, Sarah Jefferies, Sharmini Muttaiyah, Susan Morpeth, Susan Taylor, Timothy Blackmore, Vani Sathyendran, Veronica Playle, Virginia Hope, Erasmus Smit, Lauren Jelly, Olin Silander, Joep de Ligt
EPI_ISL_622817, EPI_ISL_622818, EPI_ISL_622819, EPI_ISL_622820	Middlemore Hospital	Institute of Environmental Science and Research (ESR)	Xiaoyun Ren, Matt Storey, Nikki Freed, Muhammad Faisal, Jing Wang, Hermes Perez, Anja Werno, Antje van der Linden, Arlo Upton, Chris Mansell, David Hammer, Dragana Drinkovic, Gary McAuliffe, Hana Sofia Andersson, James Ussher, Jill Sherwood, Josh Freeman, Julia Howard, Juliet Elvy, Mary DeAlmeida, Matt Blakiston, Matthew Rogers, Max Bloomfield, Michael Addidle, Michelle Balm, Sally Roberts, Sarah Jefferies, Sharmini Muttaiyah, Susan Morpeth, Susan Taylor, Timothy Blackmore, Vani Sathyendran, Veronica Playle, Virginia Hope, Erasmus Smit, Lauren Jelly, Olin Silander, Joep de Ligt
EPI_ISL_622821, EPI_ISL_622822, EPI_ISL_622823	LabPLUS	Institute of Environmental Science and Research (ESR)	Xiaoyun Ren, Matt Storey, Nikki Freed, Muhammad Faisal, Jing Wang, Hermes Perez, Anja Werno, Antje van der Linden, Arlo Upton, Chris Mansell, David Hammer, Dragana Drinkovic, Gary McAuliffe, Hana Sofia Andersson, James Ussher, Jill Sherwood, Josh Freeman, Julia Howard, Juliet Elvy, Mary DeAlmeida, Matt Blakiston, Matthew Rogers, Max Bloomfield, Michael Addidle, Michelle Balm, Sally Roberts, Sarah Jefferies, Sharmini Muttaiyah, Susan Morpeth, Susan Taylor, Timothy Blackmore, Vani Sathyendran, Veronica Playle, Virginia Hope, Erasmus Smit, Lauren Jelly, Olin Silander, Joep de Ligt
EPI_ISL_622824, EPI_ISL_622825, EPI_ISL_622826, EPI_ISL_622827, EPI_ISL_622828, EPI_ISL_622829, EPI_ISL_622830	Canterbury Health Laboratories	Institute of Environmental Science and Research (ESR)	Xiaoyun Ren, Matt Storey, Nikki Freed, Muhammad Faisal, Jing Wang, Hermes Perez, Anja Werno, Antje van der Linden, Arlo Upton, Chris Mansell, David Hammer, Dragana Drinkovic, Gary McAuliffe, Hana Sofia Andersson, James Ussher, Jill Sherwood, Josh Freeman, Julia Howard, Juliet Elvy, Mary DeAlmeida, Matt Blakiston, Matthew Rogers, Max Bloomfield, Michael Addidle, Michelle Balm, Sally Roberts, Sarah Jefferies, Sharmini Muttaiyah, Susan Morpeth, Susan Taylor, Timothy Blackmore, Vani Sathyendran, Veronica Playle, Virginia Hope, Erasmus Smit, Lauren Jelly, Olin Silander, Joep de Ligt
EPI_ISL_622831, EPI_ISL_622832	Middlemore Hospital	Institute of Environmental Science and Research (ESR)	Xiaoyun Ren, Matt Storey, Nikki Freed, Muhammad Faisal, Jing Wang, Hermes Perez, Anja Werno, Antje van der Linden, Arlo Upton, Chris Mansell, David Hammer, Dragana Drinkovic, Gary McAuliffe, Hana Sofia Andersson, James Ussher, Jill Sherwood, Josh Freeman, Julia Howard, Juliet Elvy, Mary

			DeAlmeida, Matt Blakiston, Matthew Rogers, Max Bloomfield, Michael Addidle, Michelle Balm, Sally Roberts, Sarah Jefferies, Sharmini Muttaiyah, Susan Morpeth, Susan Taylor, Timothy Blackmore, Vani Sathyendran, Veronica Playle, Virginia Hope, Erasmus Smit, Lauren Jelly, Olin Silander, Joep de Ligt
EPI_ISL_622833, EPI_ISL_622835, EPI_ISL_622836, EPI_ISL_622837, EPI_ISL_622838, EPI_ISL_622839, EPI_ISL_622840, EPI_ISL_622841, EPI_ISL_622842, EPI_ISL_622843, EPI_ISL_622844, EPI_ISL_622845, EPI_ISL_622846, EPI_ISL_622847, EPI_ISL_622848, EPI_ISL_622849, EPI_ISL_622850, EPI_ISL_622851, EPI_ISL_622852, EPI_ISL_622853, EPI_ISL_622854, EPI_ISL_622855, EPI_ISL_622856, EPI_ISL_622857, EPI_ISL_622858, EPI_ISL_622859, EPI_ISL_622860, EPI_ISL_622861, EPI_ISL_622862, EPI_ISL_622863, EPI_ISL_622864, EPI_ISL_622865, EPI_ISL_622866, EPI_ISL_622867, EPI_ISL_622868, EPI_ISL_622869, EPI_ISL_622870, EPI_ISL_622871, EPI_ISL_622872, EPI_ISL_622873, EPI_ISL_622874, EPI_ISL_622875, EPI_ISL_622877, EPI_ISL_622878, EPI_ISL_622881, EPI_ISL_622882, EPI_ISL_622883, EPI_ISL_622884, EPI_ISL_622885, EPI_ISL_622886, EPI_ISL_622887, EPI_ISL_622888, EPI_ISL_622889, EPI_ISL_622890, EPI_ISL_622892			
see above	Respiratory Virus Unit, Microbiology Services Colindale, Public Health England	Respiratory Virus Unit, Microbiology Services Colindale, Public Health England	PHE Covid Sequencing Team
EPI_ISL_622922, EPI_ISL_622934, EPI_ISL_622935, EPI_ISL_622936, EPI_ISL_622937, EPI_ISL_622938, EPI_ISL_622939, EPI_ISL_622940, EPI_ISL_622941	National Institute for Communicable Diseases of the National Health Laboratory Service	National Institute for Communicable Diseases of the National Health Laboratory Service	Allam M, Ismail A, Khumalo Z, Kwenda S, Mtshali P, Mnyameni F, Mohale T, Subramoney K, Bhiman JN
EPI_ISL_623073	Lancet Laboratories	National Institute for Communicable Diseases of the National Health Laboratory Service	Allam M, Ismail A, Khumalo Z, Kwenda S, Mtshali P, Mnyameni F, Mohale T, Subramoney K, Bhiman JN
EPI_ISL_623076, EPI_ISL_623077, EPI_ISL_623078, EPI_ISL_623079	Uppsala klinisk mikrobiologi	The Public Health Agency of Sweden	Anna-Malin Linde, Maria Lind Karlberg, Mattias Haukland, Reza Advani, Olov Svartstrom, Oskar Karlsson Lindsjo, Sandra Broddesson, Petra Edquist, Mia Brytting, Anna Risberg, Karin Tegmark-Wisell
EPI_ISL_623080, EPI_ISL_623081, EPI_ISL_623082, EPI_ISL_623083, EPI_ISL_623084, EPI_ISL_623085	Klinisk mikrobiologi, Skanes universitetssjukhus, Lund	The Public Health Agency of Sweden	Anna-Malin Linde, Maria Lind Karlberg, Mattias Haukland, Reza Advani, Olov Svartstrom, Oskar Karlsson Lindsjo, Sandra Broddesson, Petra Edquist, Mia Brytting, Anna Risberg, Karin Tegmark-Wisell
EPI_ISL_623086	Halmstad klinisk mikrobiologi	The Public Health Agency of Sweden	Anna-Malin Linde, Maria Lind Karlberg, Mattias Haukland, Reza Advani, Olov Svartstrom, Oskar Karlsson Lindsjo, Sandra Broddesson, Petra Edquist, Mia Brytting, Anna Risberg, Karin Tegmark-Wisell
EPI_ISL_623088	Klinisk mikrobiologi NAL Trollhattan	The Public Health Agency of Sweden	Anna-Malin Linde, Maria Lind Karlberg, Mattias Haukland, Reza Advani, Olov Svartstrom, Oskar Karlsson Lindsjo, Sandra Broddesson, Petra Edquist, Mia Brytting, Anna Risberg, Karin Tegmark-Wisell
EPI_ISL_623089	Klinisk mikrobiologi Linkoping	The Public Health Agency of Sweden	Anna-Malin Linde, Maria Lind Karlberg, Mattias Haukland, Reza Advani, Olov Svartstrom, Oskar Karlsson Lindsjo, Sandra Broddesson, Petra Edquist, Mia Brytting, Anna Risberg, Karin Tegmark-Wisell
EPI_ISL_623090	Klinisk mikrobiologi Vasternorrland	The Public Health Agency of Sweden	Anna-Malin Linde, Maria Lind Karlberg, Mattias Haukland, Reza Advani, Olov Svartstrom, Oskar Karlsson Lindsjo, Sandra Broddesson, Petra Edquist, Mia Brytting, Anna Risberg, Karin Tegmark-Wisell
EPI_ISL_623091	Kalmar klinisk mikrobiologi	The Public Health Agency of Sweden	Anna-Malin Linde, Maria Lind Karlberg, Mattias Haukland, Reza Advani, Olov Svartstrom, Oskar Karlsson Lindsjo, Sandra Broddesson, Petra Edquist, Mia Brytting, Anna Risberg, Karin Tegmark-Wisell
EPI_ISL_623092, EPI_ISL_623093, EPI_ISL_623094	Klinisk mikrobiologi Lanssjukhuset Ryhov, Jonkoping	The Public Health Agency of Sweden	Anna-Malin Linde, Maria Lind Karlberg, Mattias Haukland, Reza Advani, Olov Svartstrom, Oskar Karlsson Lindsjo, Sandra Broddesson, Petra Edquist, Mia Brytting, Anna Risberg, Karin Tegmark-Wisell
EPI_ISL_623095	Gavle klinisk mikrobiologi	The Public Health Agency of Sweden	Anna-Malin Linde, Maria Lind Karlberg, Mattias Haukland, Reza Advani, Olov Svartstrom, Oskar Karlsson Lindsjo, Sandra Broddesson, Petra Edquist, Mia Brytting, Anna Risberg, Karin Tegmark-Wisell
EPI_ISL_623096, EPI_ISL_623097	General practitioner	National Reference Center for Viruses of Respiratory Infections, Institut Pasteur, Paris	Marion Barbet, Sylvie Behillil, Méline Bizard, Angela Brisebarre, Camille Capel, Etienne Simon-Lorière, Vincent Enouf, Maud Vanpeene, Sylvie van der Werf
EPI_ISL_623099, EPI_ISL_623100, EPI_ISL_623101, EPI_ISL_623102	CNR Virus des Infections Respiratoires - France SUD	CNR Virus des Infections Respiratoires - France SUD	Antonin Bal, Gregory Destras, Gwendolyne Burfin, Hadrien Règue, Alexandre Gaymard, Maude Bouscambert-Duchamp, Florence Morfin-Sherpa, Martine Valette, Bruno Lina, Laurence Josset
EPI_ISL_623108, EPI_ISL_623110, EPI_ISL_623112, EPI_ISL_623114, EPI_ISL_623116, EPI_ISL_623118, EPI_ISL_623119, EPI_ISL_623120, EPI_ISL_623124, EPI_ISL_623126, EPI_ISL_623129, EPI_ISL_623130, EPI_ISL_623132, EPI_ISL_623134, EPI_ISL_623136, EPI_ISL_623138, EPI_ISL_623140, EPI_ISL_623142, EPI_ISL_623143, EPI_ISL_623144, EPI_ISL_623145, EPI_ISL_623146, EPI_ISL_623147, EPI_ISL_623148, EPI_ISL_623149, EPI_ISL_623152, EPI_ISL_623154, EPI_ISL_623157, EPI_ISL_623158, EPI_ISL_623160, EPI_ISL_623161, EPI_ISL_623162, EPI_ISL_623164, EPI_ISL_623165, EPI_ISL_623166, EPI_ISL_623168			
see above	Laboratorio de Virologia Molecular / UFRJ	Bioinformatics Laboratory / LNCC	Carolina M Voloch, Ronaldo S Francisco Jr, Luiz G P de Almeida, Otavio J. Brustolini, Cynthia C Cardoso, Alexandra L Gerber, Ana Paula de C Guimarães, Diana Mariani, Covid19-UFRJ Workgroup, Luís Cristóvão Pôrto, Renato S Aguiar, Terezinha M P P Castiñeiras, Orlando C. Ferreira, Amilcar Tanuri, Ana Tereza R de Vasconcelos
EPI_ISL_623171, EPI_ISL_623172, EPI_ISL_623174, EPI_ISL_623175, EPI_ISL_623176, EPI_ISL_623177, EPI_ISL_623178, EPI_ISL_623179, EPI_ISL_623180, EPI_ISL_623182, EPI_ISL_623183, EPI_ISL_623185, EPI_ISL_623186, EPI_ISL_623188, EPI_ISL_623189, EPI_ISL_623190, EPI_ISL_623191, EPI_ISL_623192, EPI_ISL_623193, EPI_ISL_623195, EPI_ISL_623196, EPI_ISL_623197, EPI_ISL_623199, EPI_ISL_623200, EPI_ISL_623202, EPI_ISL_623203, EPI_ISL_623205, EPI_ISL_623207, EPI_ISL_623208			
see above	Utah Public Health Laboratory	Utah Public Health Laboratory	Erin Young, Kelly Oakeson
EPI_ISL_623211, EPI_ISL_623212, EPI_ISL_623213, EPI_ISL_623215, EPI_ISL_623216, EPI_ISL_623217, EPI_ISL_623218, EPI_ISL_623219	Lighthouse Lab in Milton Keynes	Wellcome Sanger Institute for the COVID-19 Genomics UK (COG-UK) consortium	The Lighthouse Lab in Milton Keynes and Alex Alderton, Roberto Amato, Sonia Goncalves, Ewan Harrison, David K. Jackson, Ian Johnston, Dominic Kwiatkowski, Cordelia Langford, John Sillitoe on behalf of the Wellcome Sanger Institute COVID-19 Surveillance Team (http://www.sanger.ac.uk/covid-team)
EPI_ISL_623220	Lighthouse Lab in Cambridge	Wellcome Sanger Institute for the COVID-19 Genomics UK (COG-UK) consortium	Rob Howes, The Lighthouse Lab in Cambridge and Alex Alderton, Roberto Amato, Sonia Goncalves, Ewan Harrison, David K. Jackson, Ian Johnston, Dominic Kwiatkowski, Cordelia Langford, John Sillitoe on behalf of the Wellcome Sanger Institute COVID-19 Surveillance Team (http://www.sanger.ac.uk/covid-team)
EPI_ISL_623221, EPI_ISL_623223, EPI_ISL_623225, EPI_ISL_623226, EPI_ISL_623227, EPI_ISL_623228, EPI_ISL_623229, EPI_ISL_623230, EPI_ISL_623231, EPI_ISL_623232, EPI_ISL_623233, EPI_ISL_623236, EPI_ISL_623237, EPI_ISL_623239, EPI_ISL_623240, EPI_ISL_623241, EPI_ISL_623242, EPI_ISL_623243, EPI_ISL_623244, EPI_ISL_623246			
see above	Lighthouse Lab in Milton Keynes	Wellcome Sanger Institute for the COVID-19 Genomics UK (COG-UK) consortium	The Lighthouse Lab in Milton Keynes and Alex Alderton, Roberto Amato, Sonia Goncalves, Ewan Harrison, David K. Jackson, Ian Johnston, Dominic Kwiatkowski, Cordelia Langford, John Sillitoe on behalf of the Wellcome Sanger Institute COVID-19 Surveillance Team (http://www.sanger.ac.uk/covid-team)
EPI_ISL_623247, EPI_ISL_623248	Lighthouse Lab in Cambridge	Wellcome Sanger Institute for the COVID-19 Genomics UK (COG-UK) consortium	Rob Howes, The Lighthouse Lab in Cambridge and Alex Alderton, Roberto Amato, Sonia Goncalves, Ewan Harrison, David K. Jackson, Ian Johnston, Dominic Kwiatkowski, Cordelia Langford, John Sillitoe on behalf of the Wellcome Sanger Institute COVID-19 Surveillance Team (http://www.sanger.ac.uk/covid-team)
EPI_ISL_623249, EPI_ISL_623250, EPI_ISL_623251, EPI_ISL_623253, EPI_ISL_623254, EPI_ISL_623255	Lighthouse Lab in Milton Keynes	Wellcome Sanger Institute for the COVID-19 Genomics UK (COG-UK) consortium	The Lighthouse Lab in Milton Keynes and Alex Alderton, Roberto Amato, Sonia Goncalves, Ewan Harrison, David K. Jackson, Ian Johnston, Dominic Kwiatkowski, Cordelia Langford, John Sillitoe on behalf of the Wellcome Sanger Institute COVID-19 Surveillance Team (http://www.sanger.ac.uk/covid-team)
EPI_ISL_623256	Lighthouse Lab in Cambridge	Wellcome Sanger Institute for the COVID-19 Genomics UK (COG-UK) consortium	Rob Howes, The Lighthouse Lab in Cambridge and Alex Alderton, Roberto Amato, Sonia Goncalves, Ewan Harrison, David K. Jackson, Ian Johnston, Dominic Kwiatkowski, Cordelia Langford, John Sillitoe on behalf of the Wellcome Sanger Institute COVID-19 Surveillance Team (http://www.sanger.ac.uk/covid-team)
EPI_ISL_623258, EPI_ISL_623261, EPI_ISL_623263, EPI_ISL_623265	Lighthouse Lab in Milton Keynes	Wellcome Sanger Institute for the COVID-19 Genomics UK (COG-UK) consortium	The Lighthouse Lab in Milton Keynes and Alex Alderton, Roberto Amato, Sonia Goncalves, Ewan Harrison, David K. Jackson, Ian Johnston, Dominic Kwiatkowski, Cordelia Langford, John Sillitoe on behalf of the Wellcome Sanger Institute COVID-19 Surveillance Team (http://www.sanger.ac.uk/covid-team)
EPI_ISL_623268	Lighthouse Lab in Cambridge	Wellcome Sanger Institute for the COVID-19 Genomics UK (COG-UK) consortium	Rob Howes, The Lighthouse Lab in Cambridge and Alex Alderton, Roberto Amato, Sonia Goncalves, Ewan Harrison, David K. Jackson, Ian Johnston, Dominic Kwiatkowski, Cordelia Langford, John Sillitoe on behalf of the Wellcome Sanger Institute COVID-19 Surveillance Team (http://www.sanger.ac.uk/covid-team)
EPI_ISL_623269, EPI_ISL_623270, EPI_ISL_623271	Lighthouse Lab in Milton Keynes	Wellcome Sanger Institute for the COVID-19 Genomics UK (COG-UK) consortium	The Lighthouse Lab in Milton Keynes and Alex Alderton, Roberto Amato, Sonia Goncalves, Ewan Harrison, David K. Jackson, Ian Johnston, Dominic Kwiatkowski, Cordelia Langford, John Sillitoe on behalf of the Wellcome Sanger Institute COVID-19 Surveillance Team (http://www.sanger.ac.uk/covid-team)
EPI_ISL_623273	Lighthouse Lab in Cambridge	Wellcome Sanger Institute for the COVID-19 Genomics	Rob Howes, The Lighthouse Lab in Cambridge and Alex Alderton, Roberto Amato, Sonia Goncalves, Ewan Harrison, David K. Jackson, Ian Johnston,

		UK (COG-UK) consortium	Dominic Kwiatkowski, Cordelia Langford, John Sillitoe on behalf of the Wellcome Sanger Institute COVID-19 Surveillance Team (http://www.sanger.ac.uk/covid-team)
EPI_ISL_623274, EPI_ISL_623275	Lighthouse Lab in Milton Keynes	Wellcome Sanger Institute for the COVID-19 Genomics UK (COG-UK) consortium	The Lighthouse Lab in Milton Keynes and Alex Alderton, Roberto Amato, Sonia Goncalves, Ewan Harrison, David K. Jackson, Ian Johnston, Dominic Kwiatkowski, Cordelia Langford, John Sillitoe on behalf of the Wellcome Sanger Institute COVID-19 Surveillance Team (http://www.sanger.ac.uk/covid-team)
EPI_ISL_623276	Lighthouse Lab in Cambridge	Wellcome Sanger Institute for the COVID-19 Genomics UK (COG-UK) consortium	Rob Howes, The Lighthouse Lab in Cambridge and Alex Alderton, Roberto Amato, Sonia Goncalves, Ewan Harrison, David K. Jackson, Ian Johnston, Dominic Kwiatkowski, Cordelia Langford, John Sillitoe on behalf of the Wellcome Sanger Institute COVID-19 Surveillance Team (http://www.sanger.ac.uk/covid-team)
EPI_ISL_623277, EPI_ISL_623279, EPI_ISL_623280	Lighthouse Lab in Milton Keynes	Wellcome Sanger Institute for the COVID-19 Genomics UK (COG-UK) consortium	The Lighthouse Lab in Milton Keynes and Alex Alderton, Roberto Amato, Sonia Goncalves, Ewan Harrison, David K. Jackson, Ian Johnston, Dominic Kwiatkowski, Cordelia Langford, John Sillitoe on behalf of the Wellcome Sanger Institute COVID-19 Surveillance Team (http://www.sanger.ac.uk/covid-team)
EPI_ISL_623281	Lighthouse Lab in Cambridge	Wellcome Sanger Institute for the COVID-19 Genomics UK (COG-UK) consortium	Rob Howes, The Lighthouse Lab in Cambridge and Alex Alderton, Roberto Amato, Sonia Goncalves, Ewan Harrison, David K. Jackson, Ian Johnston, Dominic Kwiatkowski, Cordelia Langford, John Sillitoe on behalf of the Wellcome Sanger Institute COVID-19 Surveillance Team (http://www.sanger.ac.uk/covid-team)
EPI_ISL_623282, EPI_ISL_623283, EPI_ISL_623284, EPI_ISL_623285	Lighthouse Lab in Milton Keynes	Wellcome Sanger Institute for the COVID-19 Genomics UK (COG-UK) consortium	The Lighthouse Lab in Milton Keynes and Alex Alderton, Roberto Amato, Sonia Goncalves, Ewan Harrison, David K. Jackson, Ian Johnston, Dominic Kwiatkowski, Cordelia Langford, John Sillitoe on behalf of the Wellcome Sanger Institute COVID-19 Surveillance Team (http://www.sanger.ac.uk/covid-team)
EPI_ISL_623286	Lighthouse Lab in Cambridge	Wellcome Sanger Institute for the COVID-19 Genomics UK (COG-UK) consortium	Rob Howes, The Lighthouse Lab in Cambridge and Alex Alderton, Roberto Amato, Sonia Goncalves, Ewan Harrison, David K. Jackson, Ian Johnston, Dominic Kwiatkowski, Cordelia Langford, John Sillitoe on behalf of the Wellcome Sanger Institute COVID-19 Surveillance Team (http://www.sanger.ac.uk/covid-team)
EPI_ISL_623287	Lighthouse Lab in Milton Keynes	Wellcome Sanger Institute for the COVID-19 Genomics UK (COG-UK) consortium	The Lighthouse Lab in Milton Keynes and Alex Alderton, Roberto Amato, Sonia Goncalves, Ewan Harrison, David K. Jackson, Ian Johnston, Dominic Kwiatkowski, Cordelia Langford, John Sillitoe on behalf of the Wellcome Sanger Institute COVID-19 Surveillance Team (http://www.sanger.ac.uk/covid-team)
EPI_ISL_623288	Lighthouse Lab in Cambridge	Wellcome Sanger Institute for the COVID-19 Genomics UK (COG-UK) consortium	Rob Howes, The Lighthouse Lab in Cambridge and Alex Alderton, Roberto Amato, Sonia Goncalves, Ewan Harrison, David K. Jackson, Ian Johnston, Dominic Kwiatkowski, Cordelia Langford, John Sillitoe on behalf of the Wellcome Sanger Institute COVID-19 Surveillance Team (http://www.sanger.ac.uk/covid-team)
EPI_ISL_623289, EPI_ISL_623290, EPI_ISL_623291, EPI_ISL_623292, EPI_ISL_623293, EPI_ISL_623294, EPI_ISL_623296, EPI_ISL_623297, EPI_ISL_623299, EPI_ISL_623300	Lighthouse Lab in Milton Keynes	Wellcome Sanger Institute for the COVID-19 Genomics UK (COG-UK) consortium	The Lighthouse Lab in Milton Keynes and Alex Alderton, Roberto Amato, Sonia Goncalves, Ewan Harrison, David K. Jackson, Ian Johnston, Dominic Kwiatkowski, Cordelia Langford, John Sillitoe on behalf of the Wellcome Sanger Institute COVID-19 Surveillance Team (http://www.sanger.ac.uk/covid-team)
EPI_ISL_623301	Lighthouse Lab in Cambridge	Wellcome Sanger Institute for the COVID-19 Genomics UK (COG-UK) consortium	Rob Howes, The Lighthouse Lab in Cambridge and Alex Alderton, Roberto Amato, Sonia Goncalves, Ewan Harrison, David K. Jackson, Ian Johnston, Dominic Kwiatkowski, Cordelia Langford, John Sillitoe on behalf of the Wellcome Sanger Institute COVID-19 Surveillance Team (http://www.sanger.ac.uk/covid-team)
EPI_ISL_623302, EPI_ISL_623303, EPI_ISL_623304, EPI_ISL_623305, EPI_ISL_623306, EPI_ISL_623307	Lighthouse Lab in Milton Keynes	Wellcome Sanger Institute for the COVID-19 Genomics UK (COG-UK) consortium	The Lighthouse Lab in Milton Keynes and Alex Alderton, Roberto Amato, Sonia Goncalves, Ewan Harrison, David K. Jackson, Ian Johnston, Dominic Kwiatkowski, Cordelia Langford, John Sillitoe on behalf of the Wellcome Sanger Institute COVID-19 Surveillance Team (http://www.sanger.ac.uk/covid-team)
EPI_ISL_623308	Lighthouse Lab in Cambridge	Wellcome Sanger Institute for the COVID-19 Genomics UK (COG-UK) consortium	Rob Howes, The Lighthouse Lab in Cambridge and Alex Alderton, Roberto Amato, Sonia Goncalves, Ewan Harrison, David K. Jackson, Ian Johnston, Dominic Kwiatkowski, Cordelia Langford, John Sillitoe on behalf of the Wellcome Sanger Institute COVID-19 Surveillance Team (http://www.sanger.ac.uk/covid-team)
EPI_ISL_623309, EPI_ISL_623310	Lighthouse Lab in Milton Keynes	Wellcome Sanger Institute for the COVID-19 Genomics UK (COG-UK) consortium	The Lighthouse Lab in Milton Keynes and Alex Alderton, Roberto Amato, Sonia Goncalves, Ewan Harrison, David K. Jackson, Ian Johnston, Dominic Kwiatkowski, Cordelia Langford, John Sillitoe on behalf of the Wellcome Sanger Institute COVID-19 Surveillance Team (http://www.sanger.ac.uk/covid-team)
EPI_ISL_623312	Lighthouse Lab in Cambridge	Wellcome Sanger Institute for the COVID-19 Genomics UK (COG-UK) consortium	Rob Howes, The Lighthouse Lab in Cambridge and Alex Alderton, Roberto Amato, Sonia Goncalves, Ewan Harrison, David K. Jackson, Ian Johnston, Dominic Kwiatkowski, Cordelia Langford, John Sillitoe on behalf of the Wellcome Sanger Institute COVID-19 Surveillance Team (http://www.sanger.ac.uk/covid-team)
EPI_ISL_623314, EPI_ISL_623315, EPI_ISL_623316, EPI_ISL_623317, EPI_ISL_623318, EPI_ISL_623319, EPI_ISL_623320	Lighthouse Lab in Milton Keynes	Wellcome Sanger Institute for the COVID-19 Genomics UK (COG-UK) consortium	The Lighthouse Lab in Milton Keynes and Alex Alderton, Roberto Amato, Sonia Goncalves, Ewan Harrison, David K. Jackson, Ian Johnston, Dominic Kwiatkowski, Cordelia Langford, John Sillitoe on behalf of the Wellcome Sanger Institute COVID-19 Surveillance Team (http://www.sanger.ac.uk/covid-team)
EPI_ISL_623321	Lighthouse Lab in Cambridge	Wellcome Sanger Institute for the COVID-19 Genomics UK (COG-UK) consortium	Rob Howes, The Lighthouse Lab in Cambridge and Alex Alderton, Roberto Amato, Sonia Goncalves, Ewan Harrison, David K. Jackson, Ian Johnston, Dominic Kwiatkowski, Cordelia Langford, John Sillitoe on behalf of the Wellcome Sanger Institute COVID-19 Surveillance Team (http://www.sanger.ac.uk/covid-team)
EPI_ISL_623322	Lighthouse Lab in Milton Keynes	Wellcome Sanger Institute for the COVID-19 Genomics UK (COG-UK) consortium	The Lighthouse Lab in Milton Keynes and Alex Alderton, Roberto Amato, Sonia Goncalves, Ewan Harrison, David K. Jackson, Ian Johnston, Dominic Kwiatkowski, Cordelia Langford, John Sillitoe on behalf of the Wellcome Sanger Institute COVID-19 Surveillance Team (http://www.sanger.ac.uk/covid-team)
EPI_ISL_623323	Lighthouse Lab in Cambridge	Wellcome Sanger Institute for the COVID-19 Genomics UK (COG-UK) consortium	Rob Howes, The Lighthouse Lab in Cambridge and Alex Alderton, Roberto Amato, Sonia Goncalves, Ewan Harrison, David K. Jackson, Ian Johnston, Dominic Kwiatkowski, Cordelia Langford, John Sillitoe on behalf of the Wellcome Sanger Institute COVID-19 Surveillance Team (http://www.sanger.ac.uk/covid-team)
EPI_ISL_623324, EPI_ISL_623327, EPI_ISL_623328, EPI_ISL_623329	Lighthouse Lab in Milton Keynes	Wellcome Sanger Institute for the COVID-19 Genomics UK (COG-UK) consortium	The Lighthouse Lab in Milton Keynes and Alex Alderton, Roberto Amato, Sonia Goncalves, Ewan Harrison, David K. Jackson, Ian Johnston, Dominic Kwiatkowski, Cordelia Langford, John Sillitoe on behalf of the Wellcome Sanger Institute COVID-19 Surveillance Team (http://www.sanger.ac.uk/covid-team)
EPI_ISL_623330	Lighthouse Lab in Cambridge	Wellcome Sanger Institute for the COVID-19 Genomics UK (COG-UK) consortium	Rob Howes, The Lighthouse Lab in Cambridge and Alex Alderton, Roberto Amato, Sonia Goncalves, Ewan Harrison, David K. Jackson, Ian Johnston, Dominic Kwiatkowski, Cordelia Langford, John Sillitoe on behalf of the Wellcome Sanger Institute COVID-19 Surveillance Team (http://www.sanger.ac.uk/covid-team)
EPI_ISL_623331, EPI_ISL_623332	Lighthouse Lab in Milton Keynes	Wellcome Sanger Institute for the COVID-19 Genomics UK (COG-UK) consortium	The Lighthouse Lab in Milton Keynes and Alex Alderton, Roberto Amato, Sonia Goncalves, Ewan Harrison, David K. Jackson, Ian Johnston, Dominic Kwiatkowski, Cordelia Langford, John Sillitoe on behalf of the Wellcome Sanger Institute COVID-19 Surveillance Team (http://www.sanger.ac.uk/covid-team)
EPI_ISL_623333, EPI_ISL_623334	Lighthouse Lab in Cambridge	Wellcome Sanger Institute for the COVID-19 Genomics UK (COG-UK) consortium	Rob Howes, The Lighthouse Lab in Cambridge and Alex Alderton, Roberto Amato, Sonia Goncalves, Ewan Harrison, David K. Jackson, Ian Johnston, Dominic Kwiatkowski, Cordelia Langford, John Sillitoe on behalf of the Wellcome Sanger Institute COVID-19 Surveillance Team (http://www.sanger.ac.uk/covid-team)
EPI_ISL_623335, EPI_ISL_623336, EPI_ISL_623338, EPI_ISL_623339, EPI_ISL_623340, EPI_ISL_623341, EPI_ISL_623342	Lighthouse Lab in Milton Keynes	Wellcome Sanger Institute for the COVID-19 Genomics UK (COG-UK) consortium	The Lighthouse Lab in Milton Keynes and Alex Alderton, Roberto Amato, Sonia Goncalves, Ewan Harrison, David K. Jackson, Ian Johnston, Dominic Kwiatkowski, Cordelia Langford, John Sillitoe on behalf of the Wellcome Sanger Institute COVID-19 Surveillance Team (http://www.sanger.ac.uk/covid-team)
EPI_ISL_623344, EPI_ISL_623345, EPI_ISL_623346, EPI_ISL_623347	Lighthouse Lab in Cambridge	Wellcome Sanger Institute for the COVID-19 Genomics UK (COG-UK) consortium	Rob Howes, The Lighthouse Lab in Cambridge and Alex Alderton, Roberto Amato, Sonia Goncalves, Ewan Harrison, David K. Jackson, Ian Johnston, Dominic Kwiatkowski, Cordelia Langford, John Sillitoe on behalf of the Wellcome Sanger Institute COVID-19 Surveillance Team (http://www.sanger.ac.uk/covid-team)

EPI_ISL_623350, EPI_ISL_623351, EPI_ISL_623352, EPI_ISL_623353, EPI_ISL_623354, EPI_ISL_623355, EPI_ISL_623356, EPI_ISL_623357, EPI_ISL_623358	Lighthouse Lab in Milton Keynes	Wellcome Sanger Institute for the COVID-19 Genomics UK (COG-UK) consortium	The Lighthouse Lab in Milton Keynes and Alex Alderton, Roberto Amato, Sonia Goncalves, Ewan Harrison, David K. Jackson, Ian Johnston, Dominic Kwiatkowski, Cordelia Langford, John Sillitoe on behalf of the Wellcome Sanger Institute COVID-19 Surveillance Team (http://www.sanger.ac.uk/covid-team)
EPI_ISL_623359, EPI_ISL_623360	Lighthouse Lab in Cambridge	Wellcome Sanger Institute for the COVID-19 Genomics UK (COG-UK) consortium	Rob Howes, The Lighthouse Lab in Cambridge and Alex Alderton, Roberto Amato, Sonia Goncalves, Ewan Harrison, David K. Jackson, Ian Johnston, Dominic Kwiatkowski, Cordelia Langford, John Sillitoe on behalf of the Wellcome Sanger Institute COVID-19 Surveillance Team (http://www.sanger.ac.uk/covid-team)
EPI_ISL_623361, EPI_ISL_623362	Lighthouse Lab in Milton Keynes	Wellcome Sanger Institute for the COVID-19 Genomics UK (COG-UK) consortium	The Lighthouse Lab in Milton Keynes and Alex Alderton, Roberto Amato, Sonia Goncalves, Ewan Harrison, David K. Jackson, Ian Johnston, Dominic Kwiatkowski, Cordelia Langford, John Sillitoe on behalf of the Wellcome Sanger Institute COVID-19 Surveillance Team (http://www.sanger.ac.uk/covid-team)
EPI_ISL_623363, EPI_ISL_623364	Lighthouse Lab in Cambridge	Wellcome Sanger Institute for the COVID-19 Genomics UK (COG-UK) consortium	Rob Howes, The Lighthouse Lab in Cambridge and Alex Alderton, Roberto Amato, Sonia Goncalves, Ewan Harrison, David K. Jackson, Ian Johnston, Dominic Kwiatkowski, Cordelia Langford, John Sillitoe on behalf of the Wellcome Sanger Institute COVID-19 Surveillance Team (http://www.sanger.ac.uk/covid-team)
EPI_ISL_623365	Lighthouse Lab in Milton Keynes	Wellcome Sanger Institute for the COVID-19 Genomics UK (COG-UK) consortium	The Lighthouse Lab in Milton Keynes and Alex Alderton, Roberto Amato, Sonia Goncalves, Ewan Harrison, David K. Jackson, Ian Johnston, Dominic Kwiatkowski, Cordelia Langford, John Sillitoe on behalf of the Wellcome Sanger Institute COVID-19 Surveillance Team (http://www.sanger.ac.uk/covid-team)
EPI_ISL_623366	Lighthouse Lab in Cambridge	Wellcome Sanger Institute for the COVID-19 Genomics UK (COG-UK) consortium	Rob Howes, The Lighthouse Lab in Cambridge and Alex Alderton, Roberto Amato, Sonia Goncalves, Ewan Harrison, David K. Jackson, Ian Johnston, Dominic Kwiatkowski, Cordelia Langford, John Sillitoe on behalf of the Wellcome Sanger Institute COVID-19 Surveillance Team (http://www.sanger.ac.uk/covid-team)
EPI_ISL_623367, EPI_ISL_623368, EPI_ISL_623369	Lighthouse Lab in Milton Keynes	Wellcome Sanger Institute for the COVID-19 Genomics UK (COG-UK) consortium	The Lighthouse Lab in Milton Keynes and Alex Alderton, Roberto Amato, Sonia Goncalves, Ewan Harrison, David K. Jackson, Ian Johnston, Dominic Kwiatkowski, Cordelia Langford, John Sillitoe on behalf of the Wellcome Sanger Institute COVID-19 Surveillance Team (http://www.sanger.ac.uk/covid-team)
EPI_ISL_623370	Lighthouse Lab in Cambridge	Wellcome Sanger Institute for the COVID-19 Genomics UK (COG-UK) consortium	Rob Howes, The Lighthouse Lab in Cambridge and Alex Alderton, Roberto Amato, Sonia Goncalves, Ewan Harrison, David K. Jackson, Ian Johnston, Dominic Kwiatkowski, Cordelia Langford, John Sillitoe on behalf of the Wellcome Sanger Institute COVID-19 Surveillance Team (http://www.sanger.ac.uk/covid-team)
EPI_ISL_623372, EPI_ISL_623373, EPI_ISL_623375, EPI_ISL_623376, EPI_ISL_623377, EPI_ISL_623378, EPI_ISL_623379, EPI_ISL_623380, EPI_ISL_623381, EPI_ISL_623382, EPI_ISL_623383, EPI_ISL_623384			
see above	Lighthouse Lab in Milton Keynes	Wellcome Sanger Institute for the COVID-19 Genomics UK (COG-UK) consortium	The Lighthouse Lab in Milton Keynes and Alex Alderton, Roberto Amato, Sonia Goncalves, Ewan Harrison, David K. Jackson, Ian Johnston, Dominic Kwiatkowski, Cordelia Langford, John Sillitoe on behalf of the Wellcome Sanger Institute COVID-19 Surveillance Team (http://www.sanger.ac.uk/covid-team)
EPI_ISL_623385, EPI_ISL_623386	Lighthouse Lab in Cambridge	Wellcome Sanger Institute for the COVID-19 Genomics UK (COG-UK) consortium	Rob Howes, The Lighthouse Lab in Cambridge and Alex Alderton, Roberto Amato, Sonia Goncalves, Ewan Harrison, David K. Jackson, Ian Johnston, Dominic Kwiatkowski, Cordelia Langford, John Sillitoe on behalf of the Wellcome Sanger Institute COVID-19 Surveillance Team (http://www.sanger.ac.uk/covid-team)
EPI_ISL_623389	Lighthouse Lab in Milton Keynes	Wellcome Sanger Institute for the COVID-19 Genomics UK (COG-UK) consortium	The Lighthouse Lab in Milton Keynes and Alex Alderton, Roberto Amato, Sonia Goncalves, Ewan Harrison, David K. Jackson, Ian Johnston, Dominic Kwiatkowski, Cordelia Langford, John Sillitoe on behalf of the Wellcome Sanger Institute COVID-19 Surveillance Team (http://www.sanger.ac.uk/covid-team)
EPI_ISL_623390, EPI_ISL_623392, EPI_ISL_623393	Lighthouse Lab in Cambridge	Wellcome Sanger Institute for the COVID-19 Genomics UK (COG-UK) consortium	Rob Howes, The Lighthouse Lab in Cambridge and Alex Alderton, Roberto Amato, Sonia Goncalves, Ewan Harrison, David K. Jackson, Ian Johnston, Dominic Kwiatkowski, Cordelia Langford, John Sillitoe on behalf of the Wellcome Sanger Institute COVID-19 Surveillance Team (http://www.sanger.ac.uk/covid-team)
EPI_ISL_623394, EPI_ISL_623395, EPI_ISL_623396	Lighthouse Lab in Milton Keynes	Wellcome Sanger Institute for the COVID-19 Genomics UK (COG-UK) consortium	The Lighthouse Lab in Milton Keynes and Alex Alderton, Roberto Amato, Sonia Goncalves, Ewan Harrison, David K. Jackson, Ian Johnston, Dominic Kwiatkowski, Cordelia Langford, John Sillitoe on behalf of the Wellcome Sanger Institute COVID-19 Surveillance Team (http://www.sanger.ac.uk/covid-team)
EPI_ISL_623397, EPI_ISL_623398	Lighthouse Lab in Cambridge	Wellcome Sanger Institute for the COVID-19 Genomics UK (COG-UK) consortium	Rob Howes, The Lighthouse Lab in Cambridge and Alex Alderton, Roberto Amato, Sonia Goncalves, Ewan Harrison, David K. Jackson, Ian Johnston, Dominic Kwiatkowski, Cordelia Langford, John Sillitoe on behalf of the Wellcome Sanger Institute COVID-19 Surveillance Team (http://www.sanger.ac.uk/covid-team)
EPI_ISL_623400, EPI_ISL_623401, EPI_ISL_623402, EPI_ISL_623403	Lighthouse Lab in Milton Keynes	Wellcome Sanger Institute for the COVID-19 Genomics UK (COG-UK) consortium	The Lighthouse Lab in Milton Keynes and Alex Alderton, Roberto Amato, Sonia Goncalves, Ewan Harrison, David K. Jackson, Ian Johnston, Dominic Kwiatkowski, Cordelia Langford, John Sillitoe on behalf of the Wellcome Sanger Institute COVID-19 Surveillance Team (http://www.sanger.ac.uk/covid-team)
EPI_ISL_623404	Lighthouse Lab in Cambridge	Wellcome Sanger Institute for the COVID-19 Genomics UK (COG-UK) consortium	Rob Howes, The Lighthouse Lab in Cambridge and Alex Alderton, Roberto Amato, Sonia Goncalves, Ewan Harrison, David K. Jackson, Ian Johnston, Dominic Kwiatkowski, Cordelia Langford, John Sillitoe on behalf of the Wellcome Sanger Institute COVID-19 Surveillance Team (http://www.sanger.ac.uk/covid-team)
EPI_ISL_623405, EPI_ISL_623406, EPI_ISL_623407, EPI_ISL_623408	Lighthouse Lab in Milton Keynes	Wellcome Sanger Institute for the COVID-19 Genomics UK (COG-UK) consortium	The Lighthouse Lab in Milton Keynes and Alex Alderton, Roberto Amato, Sonia Goncalves, Ewan Harrison, David K. Jackson, Ian Johnston, Dominic Kwiatkowski, Cordelia Langford, John Sillitoe on behalf of the Wellcome Sanger Institute COVID-19 Surveillance Team (http://www.sanger.ac.uk/covid-team)
EPI_ISL_623409	Lighthouse Lab in Cambridge	Wellcome Sanger Institute for the COVID-19 Genomics UK (COG-UK) consortium	Rob Howes, The Lighthouse Lab in Cambridge and Alex Alderton, Roberto Amato, Sonia Goncalves, Ewan Harrison, David K. Jackson, Ian Johnston, Dominic Kwiatkowski, Cordelia Langford, John Sillitoe on behalf of the Wellcome Sanger Institute COVID-19 Surveillance Team (http://www.sanger.ac.uk/covid-team)
EPI_ISL_623410	Lighthouse Lab in Milton Keynes	Wellcome Sanger Institute for the COVID-19 Genomics UK (COG-UK) consortium	The Lighthouse Lab in Milton Keynes and Alex Alderton, Roberto Amato, Sonia Goncalves, Ewan Harrison, David K. Jackson, Ian Johnston, Dominic Kwiatkowski, Cordelia Langford, John Sillitoe on behalf of the Wellcome Sanger Institute COVID-19 Surveillance Team (http://www.sanger.ac.uk/covid-team)
EPI_ISL_623411	Lighthouse Lab in Cambridge	Wellcome Sanger Institute for the COVID-19 Genomics UK (COG-UK) consortium	Rob Howes, The Lighthouse Lab in Cambridge and Alex Alderton, Roberto Amato, Sonia Goncalves, Ewan Harrison, David K. Jackson, Ian Johnston, Dominic Kwiatkowski, Cordelia Langford, John Sillitoe on behalf of the Wellcome Sanger Institute COVID-19 Surveillance Team (http://www.sanger.ac.uk/covid-team)
EPI_ISL_623412, EPI_ISL_623413, EPI_ISL_623415, EPI_ISL_623416, EPI_ISL_623419, EPI_ISL_623420, EPI_ISL_623422, EPI_ISL_623423	Lighthouse Lab in Milton Keynes	Wellcome Sanger Institute for the COVID-19 Genomics UK (COG-UK) consortium	The Lighthouse Lab in Milton Keynes and Alex Alderton, Roberto Amato, Sonia Goncalves, Ewan Harrison, David K. Jackson, Ian Johnston, Dominic Kwiatkowski, Cordelia Langford, John Sillitoe on behalf of the Wellcome Sanger Institute COVID-19 Surveillance Team (http://www.sanger.ac.uk/covid-team)
EPI_ISL_623424, EPI_ISL_623425, EPI_ISL_623426, EPI_ISL_623427	Lighthouse Lab in Cambridge	Wellcome Sanger Institute for the COVID-19 Genomics UK (COG-UK) consortium	Rob Howes, The Lighthouse Lab in Cambridge and Alex Alderton, Roberto Amato, Sonia Goncalves, Ewan Harrison, David K. Jackson, Ian Johnston, Dominic Kwiatkowski, Cordelia Langford, John Sillitoe on behalf of the Wellcome Sanger Institute COVID-19 Surveillance Team (http://www.sanger.ac.uk/covid-team)
EPI_ISL_623429, EPI_ISL_623430, EPI_ISL_623431, EPI_ISL_623432	Lighthouse Lab in Milton Keynes	Wellcome Sanger Institute for the COVID-19 Genomics UK (COG-UK) consortium	The Lighthouse Lab in Milton Keynes and Alex Alderton, Roberto Amato, Sonia Goncalves, Ewan Harrison, David K. Jackson, Ian Johnston, Dominic Kwiatkowski, Cordelia Langford, John Sillitoe on behalf of the Wellcome Sanger Institute COVID-19 Surveillance Team (http://www.sanger.ac.uk/covid-team)
EPI_ISL_623433	Lighthouse Lab in Cambridge	Wellcome Sanger Institute for the COVID-19 Genomics UK (COG-UK) consortium	Rob Howes, The Lighthouse Lab in Cambridge and Alex Alderton, Roberto Amato, Sonia Goncalves, Ewan Harrison, David K. Jackson, Ian Johnston, Dominic Kwiatkowski, Cordelia Langford, John Sillitoe on behalf of the Wellcome Sanger Institute COVID-19 Surveillance Team (http://www.sanger.ac.uk/covid-team)
EPI_ISL_623434, EPI_ISL_623435, EPI_ISL_623436	Lighthouse Lab in Milton Keynes	Wellcome Sanger Institute for the COVID-19 Genomics UK (COG-UK) consortium	The Lighthouse Lab in Milton Keynes and Alex Alderton, Roberto Amato, Sonia Goncalves, Ewan Harrison, David K. Jackson, Ian Johnston, Dominic Kwiatkowski, Cordelia Langford, John Sillitoe on behalf of the Wellcome Sanger Institute COVID-19 Surveillance Team

			(http://www.sanger.ac.uk/covid-team)
EPI_ISL_623437	Lighthouse Lab in Cambridge	Wellcome Sanger Institute for the COVID-19 Genomics UK (COG-UK) consortium	Rob Howes, The Lighthouse Lab in Cambridge and Alex Alderton, Roberto Amato, Sonia Goncalves, Ewan Harrison, David K. Jackson, Ian Johnston, Dominic Kwiatkowski, Cordelia Langford, John Sillitoe on behalf of the Wellcome Sanger Institute COVID-19 Surveillance Team (http://www.sanger.ac.uk/covid-team)
EPI_ISL_623439	Lighthouse Lab in Milton Keynes	Wellcome Sanger Institute for the COVID-19 Genomics UK (COG-UK) consortium	The Lighthouse Lab in Milton Keynes and Alex Alderton, Roberto Amato, Sonia Goncalves, Ewan Harrison, David K. Jackson, Ian Johnston, Dominic Kwiatkowski, Cordelia Langford, John Sillitoe on behalf of the Wellcome Sanger Institute COVID-19 Surveillance Team (http://www.sanger.ac.uk/covid-team)
EPI_ISL_623440	Lighthouse Lab in Cambridge	Wellcome Sanger Institute for the COVID-19 Genomics UK (COG-UK) consortium	Rob Howes, The Lighthouse Lab in Cambridge and Alex Alderton, Roberto Amato, Sonia Goncalves, Ewan Harrison, David K. Jackson, Ian Johnston, Dominic Kwiatkowski, Cordelia Langford, John Sillitoe on behalf of the Wellcome Sanger Institute COVID-19 Surveillance Team (http://www.sanger.ac.uk/covid-team)
EPI_ISL_623442, EPI_ISL_623443	Lighthouse Lab in Milton Keynes	Wellcome Sanger Institute for the COVID-19 Genomics UK (COG-UK) consortium	The Lighthouse Lab in Milton Keynes and Alex Alderton, Roberto Amato, Sonia Goncalves, Ewan Harrison, David K. Jackson, Ian Johnston, Dominic Kwiatkowski, Cordelia Langford, John Sillitoe on behalf of the Wellcome Sanger Institute COVID-19 Surveillance Team (http://www.sanger.ac.uk/covid-team)
EPI_ISL_623444, EPI_ISL_623445	Lighthouse Lab in Cambridge	Wellcome Sanger Institute for the COVID-19 Genomics UK (COG-UK) consortium	Rob Howes, The Lighthouse Lab in Cambridge and Alex Alderton, Roberto Amato, Sonia Goncalves, Ewan Harrison, David K. Jackson, Ian Johnston, Dominic Kwiatkowski, Cordelia Langford, John Sillitoe on behalf of the Wellcome Sanger Institute COVID-19 Surveillance Team (http://www.sanger.ac.uk/covid-team)
EPI_ISL_623446, EPI_ISL_623447	Lighthouse Lab in Milton Keynes	Wellcome Sanger Institute for the COVID-19 Genomics UK (COG-UK) consortium	The Lighthouse Lab in Milton Keynes and Alex Alderton, Roberto Amato, Sonia Goncalves, Ewan Harrison, David K. Jackson, Ian Johnston, Dominic Kwiatkowski, Cordelia Langford, John Sillitoe on behalf of the Wellcome Sanger Institute COVID-19 Surveillance Team (http://www.sanger.ac.uk/covid-team)
EPI_ISL_623448, EPI_ISL_623449, EPI_ISL_623451	Lighthouse Lab in Cambridge	Wellcome Sanger Institute for the COVID-19 Genomics UK (COG-UK) consortium	Rob Howes, The Lighthouse Lab in Cambridge and Alex Alderton, Roberto Amato, Sonia Goncalves, Ewan Harrison, David K. Jackson, Ian Johnston, Dominic Kwiatkowski, Cordelia Langford, John Sillitoe on behalf of the Wellcome Sanger Institute COVID-19 Surveillance Team (http://www.sanger.ac.uk/covid-team)
EPI_ISL_623452, EPI_ISL_623454, EPI_ISL_623455, EPI_ISL_623457, EPI_ISL_623459, EPI_ISL_623460, EPI_ISL_623461, EPI_ISL_623462, EPI_ISL_623464, EPI_ISL_623465, EPI_ISL_623466, EPI_ISL_623467, EPI_ISL_623469, EPI_ISL_623470, EPI_ISL_623471, EPI_ISL_623472, EPI_ISL_623473			
see above	Lighthouse Lab in Milton Keynes	Wellcome Sanger Institute for the COVID-19 Genomics UK (COG-UK) consortium	The Lighthouse Lab in Milton Keynes and Alex Alderton, Roberto Amato, Sonia Goncalves, Ewan Harrison, David K. Jackson, Ian Johnston, Dominic Kwiatkowski, Cordelia Langford, John Sillitoe on behalf of the Wellcome Sanger Institute COVID-19 Surveillance Team (http://www.sanger.ac.uk/covid-team)
EPI_ISL_623474	Lighthouse Lab in Cambridge	Wellcome Sanger Institute for the COVID-19 Genomics UK (COG-UK) consortium	Rob Howes, The Lighthouse Lab in Cambridge and Alex Alderton, Roberto Amato, Sonia Goncalves, Ewan Harrison, David K. Jackson, Ian Johnston, Dominic Kwiatkowski, Cordelia Langford, John Sillitoe on behalf of the Wellcome Sanger Institute COVID-19 Surveillance Team (http://www.sanger.ac.uk/covid-team)
EPI_ISL_623478, EPI_ISL_623479, EPI_ISL_623480, EPI_ISL_623481	Lighthouse Lab in Milton Keynes	Wellcome Sanger Institute for the COVID-19 Genomics UK (COG-UK) consortium	The Lighthouse Lab in Milton Keynes and Alex Alderton, Roberto Amato, Sonia Goncalves, Ewan Harrison, David K. Jackson, Ian Johnston, Dominic Kwiatkowski, Cordelia Langford, John Sillitoe on behalf of the Wellcome Sanger Institute COVID-19 Surveillance Team (http://www.sanger.ac.uk/covid-team)
EPI_ISL_623482	Lighthouse Lab in Cambridge	Wellcome Sanger Institute for the COVID-19 Genomics UK (COG-UK) consortium	Rob Howes, The Lighthouse Lab in Cambridge and Alex Alderton, Roberto Amato, Sonia Goncalves, Ewan Harrison, David K. Jackson, Ian Johnston, Dominic Kwiatkowski, Cordelia Langford, John Sillitoe on behalf of the Wellcome Sanger Institute COVID-19 Surveillance Team (http://www.sanger.ac.uk/covid-team)
EPI_ISL_623483	Lighthouse Lab in Milton Keynes	Wellcome Sanger Institute for the COVID-19 Genomics UK (COG-UK) consortium	The Lighthouse Lab in Milton Keynes and Alex Alderton, Roberto Amato, Sonia Goncalves, Ewan Harrison, David K. Jackson, Ian Johnston, Dominic Kwiatkowski, Cordelia Langford, John Sillitoe on behalf of the Wellcome Sanger Institute COVID-19 Surveillance Team (http://www.sanger.ac.uk/covid-team)
EPI_ISL_623485	Lighthouse Lab in Cambridge	Wellcome Sanger Institute for the COVID-19 Genomics UK (COG-UK) consortium	Rob Howes, The Lighthouse Lab in Cambridge and Alex Alderton, Roberto Amato, Sonia Goncalves, Ewan Harrison, David K. Jackson, Ian Johnston, Dominic Kwiatkowski, Cordelia Langford, John Sillitoe on behalf of the Wellcome Sanger Institute COVID-19 Surveillance Team (http://www.sanger.ac.uk/covid-team)
EPI_ISL_623487	Lighthouse Lab in Milton Keynes	Wellcome Sanger Institute for the COVID-19 Genomics UK (COG-UK) consortium	The Lighthouse Lab in Milton Keynes and Alex Alderton, Roberto Amato, Sonia Goncalves, Ewan Harrison, David K. Jackson, Ian Johnston, Dominic Kwiatkowski, Cordelia Langford, John Sillitoe on behalf of the Wellcome Sanger Institute COVID-19 Surveillance Team (http://www.sanger.ac.uk/covid-team)
EPI_ISL_623488, EPI_ISL_623489, EPI_ISL_623490	Lighthouse Lab in Cambridge	Wellcome Sanger Institute for the COVID-19 Genomics UK (COG-UK) consortium	Rob Howes, The Lighthouse Lab in Cambridge and Alex Alderton, Roberto Amato, Sonia Goncalves, Ewan Harrison, David K. Jackson, Ian Johnston, Dominic Kwiatkowski, Cordelia Langford, John Sillitoe on behalf of the Wellcome Sanger Institute COVID-19 Surveillance Team (http://www.sanger.ac.uk/covid-team)
EPI_ISL_623491, EPI_ISL_623492, EPI_ISL_623493	Lighthouse Lab in Milton Keynes	Wellcome Sanger Institute for the COVID-19 Genomics UK (COG-UK) consortium	The Lighthouse Lab in Milton Keynes and Alex Alderton, Roberto Amato, Sonia Goncalves, Ewan Harrison, David K. Jackson, Ian Johnston, Dominic Kwiatkowski, Cordelia Langford, John Sillitoe on behalf of the Wellcome Sanger Institute COVID-19 Surveillance Team (http://www.sanger.ac.uk/covid-team)
EPI_ISL_623494, EPI_ISL_623495	Lighthouse Lab in Cambridge	Wellcome Sanger Institute for the COVID-19 Genomics UK (COG-UK) consortium	Rob Howes, The Lighthouse Lab in Cambridge and Alex Alderton, Roberto Amato, Sonia Goncalves, Ewan Harrison, David K. Jackson, Ian Johnston, Dominic Kwiatkowski, Cordelia Langford, John Sillitoe on behalf of the Wellcome Sanger Institute COVID-19 Surveillance Team (http://www.sanger.ac.uk/covid-team)
EPI_ISL_623496, EPI_ISL_623499, EPI_ISL_623500, EPI_ISL_623501, EPI_ISL_623502, EPI_ISL_623503, EPI_ISL_623504, EPI_ISL_623505, EPI_ISL_623507, EPI_ISL_623509, EPI_ISL_623510, EPI_ISL_623511, EPI_ISL_623512, EPI_ISL_623513, EPI_ISL_623514, EPI_ISL_623516, EPI_ISL_623517, EPI_ISL_623519			
see above	Lighthouse Lab in Milton Keynes	Wellcome Sanger Institute for the COVID-19 Genomics UK (COG-UK) consortium	The Lighthouse Lab in Milton Keynes and Alex Alderton, Roberto Amato, Sonia Goncalves, Ewan Harrison, David K. Jackson, Ian Johnston, Dominic Kwiatkowski, Cordelia Langford, John Sillitoe on behalf of the Wellcome Sanger Institute COVID-19 Surveillance Team (http://www.sanger.ac.uk/covid-team)
EPI_ISL_623520	Lighthouse Lab in Cambridge	Wellcome Sanger Institute for the COVID-19 Genomics UK (COG-UK) consortium	Rob Howes, The Lighthouse Lab in Cambridge and Alex Alderton, Roberto Amato, Sonia Goncalves, Ewan Harrison, David K. Jackson, Ian Johnston, Dominic Kwiatkowski, Cordelia Langford, John Sillitoe on behalf of the Wellcome Sanger Institute COVID-19 Surveillance Team (http://www.sanger.ac.uk/covid-team)
EPI_ISL_623521, EPI_ISL_623523, EPI_ISL_623524, EPI_ISL_623525	Lighthouse Lab in Milton Keynes	Wellcome Sanger Institute for the COVID-19 Genomics UK (COG-UK) consortium	The Lighthouse Lab in Milton Keynes and Alex Alderton, Roberto Amato, Sonia Goncalves, Ewan Harrison, David K. Jackson, Ian Johnston, Dominic Kwiatkowski, Cordelia Langford, John Sillitoe on behalf of the Wellcome Sanger Institute COVID-19 Surveillance Team (http://www.sanger.ac.uk/covid-team)
EPI_ISL_623527	Lighthouse Lab in Cambridge	Wellcome Sanger Institute for the COVID-19 Genomics UK (COG-UK) consortium	Rob Howes, The Lighthouse Lab in Cambridge and Alex Alderton, Roberto Amato, Sonia Goncalves, Ewan Harrison, David K. Jackson, Ian Johnston, Dominic Kwiatkowski, Cordelia Langford, John Sillitoe on behalf of the Wellcome Sanger Institute COVID-19 Surveillance Team (http://www.sanger.ac.uk/covid-team)
EPI_ISL_623529	Lighthouse Lab in Milton Keynes	Wellcome Sanger Institute for the COVID-19 Genomics UK (COG-UK) consortium	The Lighthouse Lab in Milton Keynes and Alex Alderton, Roberto Amato, Sonia Goncalves, Ewan Harrison, David K. Jackson, Ian Johnston, Dominic Kwiatkowski, Cordelia Langford, John Sillitoe on behalf of the Wellcome Sanger Institute COVID-19 Surveillance Team (http://www.sanger.ac.uk/covid-team)
EPI_ISL_623531	Lighthouse Lab in Cambridge	Wellcome Sanger Institute for the COVID-19 Genomics UK (COG-UK) consortium	Rob Howes, The Lighthouse Lab in Cambridge and Alex Alderton, Roberto Amato, Sonia Goncalves, Ewan Harrison, David K. Jackson, Ian Johnston, Dominic Kwiatkowski, Cordelia Langford, John Sillitoe on behalf of the Wellcome Sanger Institute COVID-19 Surveillance Team (http://www.sanger.ac.uk/covid-team)
EPI_ISL_623534, EPI_ISL_623535, EPI_ISL_623536	Lighthouse Lab in Milton Keynes	Wellcome Sanger Institute for the COVID-19 Genomics UK (COG-UK) consortium	The Lighthouse Lab in Milton Keynes and Alex Alderton, Roberto Amato, Sonia Goncalves, Ewan Harrison, David K. Jackson, Ian Johnston, Dominic Kwiatkowski, Cordelia Langford, John Sillitoe on behalf of the Wellcome Sanger Institute COVID-19 Surveillance Team

			(http://www.sanger.ac.uk/covid-team)
EPI_ISL_623537, EPI_ISL_623539	Lighthouse Lab in Cambridge	Wellcome Sanger Institute for the COVID-19 Genomics UK (COG-UK) consortium	Rob Howes, The Lighthouse Lab in Cambridge and Alex Alderton, Roberto Amato, Sonia Goncalves, Ewan Harrison, David K. Jackson, Ian Johnston, Dominic Kwiatkowski, Cordelia Langford, John Sillitoe on behalf of the Wellcome Sanger Institute COVID-19 Surveillance Team (http://www.sanger.ac.uk/covid-team)
EPI_ISL_623540, EPI_ISL_623541, EPI_ISL_623542	Lighthouse Lab in Milton Keynes	Wellcome Sanger Institute for the COVID-19 Genomics UK (COG-UK) consortium	The Lighthouse Lab in Milton Keynes and Alex Alderton, Roberto Amato, Sonia Goncalves, Ewan Harrison, David K. Jackson, Ian Johnston, Dominic Kwiatkowski, Cordelia Langford, John Sillitoe on behalf of the Wellcome Sanger Institute COVID-19 Surveillance Team (http://www.sanger.ac.uk/covid-team)
EPI_ISL_623545, EPI_ISL_623547, EPI_ISL_623548	Lighthouse Lab in Cambridge	Wellcome Sanger Institute for the COVID-19 Genomics UK (COG-UK) consortium	Rob Howes, The Lighthouse Lab in Cambridge and Alex Alderton, Roberto Amato, Sonia Goncalves, Ewan Harrison, David K. Jackson, Ian Johnston, Dominic Kwiatkowski, Cordelia Langford, John Sillitoe on behalf of the Wellcome Sanger Institute COVID-19 Surveillance Team (http://www.sanger.ac.uk/covid-team)
EPI_ISL_623549	Lighthouse Lab in Milton Keynes	Wellcome Sanger Institute for the COVID-19 Genomics UK (COG-UK) consortium	The Lighthouse Lab in Milton Keynes and Alex Alderton, Roberto Amato, Sonia Goncalves, Ewan Harrison, David K. Jackson, Ian Johnston, Dominic Kwiatkowski, Cordelia Langford, John Sillitoe on behalf of the Wellcome Sanger Institute COVID-19 Surveillance Team (http://www.sanger.ac.uk/covid-team)
EPI_ISL_623550	Lighthouse Lab in Cambridge	Wellcome Sanger Institute for the COVID-19 Genomics UK (COG-UK) consortium	Rob Howes, The Lighthouse Lab in Cambridge and Alex Alderton, Roberto Amato, Sonia Goncalves, Ewan Harrison, David K. Jackson, Ian Johnston, Dominic Kwiatkowski, Cordelia Langford, John Sillitoe on behalf of the Wellcome Sanger Institute COVID-19 Surveillance Team (http://www.sanger.ac.uk/covid-team)
EPI_ISL_623552, EPI_ISL_623553	Lighthouse Lab in Milton Keynes	Wellcome Sanger Institute for the COVID-19 Genomics UK (COG-UK) consortium	The Lighthouse Lab in Milton Keynes and Alex Alderton, Roberto Amato, Sonia Goncalves, Ewan Harrison, David K. Jackson, Ian Johnston, Dominic Kwiatkowski, Cordelia Langford, John Sillitoe on behalf of the Wellcome Sanger Institute COVID-19 Surveillance Team (http://www.sanger.ac.uk/covid-team)
EPI_ISL_623556, EPI_ISL_623557, EPI_ISL_623559	Lighthouse Lab in Cambridge	Wellcome Sanger Institute for the COVID-19 Genomics UK (COG-UK) consortium	Rob Howes, The Lighthouse Lab in Cambridge and Alex Alderton, Roberto Amato, Sonia Goncalves, Ewan Harrison, David K. Jackson, Ian Johnston, Dominic Kwiatkowski, Cordelia Langford, John Sillitoe on behalf of the Wellcome Sanger Institute COVID-19 Surveillance Team (http://www.sanger.ac.uk/covid-team)
EPI_ISL_623561, EPI_ISL_623563, EPI_ISL_623564, EPI_ISL_623565, EPI_ISL_623566	Lighthouse Lab in Milton Keynes	Wellcome Sanger Institute for the COVID-19 Genomics UK (COG-UK) consortium	The Lighthouse Lab in Milton Keynes and Alex Alderton, Roberto Amato, Sonia Goncalves, Ewan Harrison, David K. Jackson, Ian Johnston, Dominic Kwiatkowski, Cordelia Langford, John Sillitoe on behalf of the Wellcome Sanger Institute COVID-19 Surveillance Team (http://www.sanger.ac.uk/covid-team)
EPI_ISL_623568, EPI_ISL_623569, EPI_ISL_623570, EPI_ISL_623572	Lighthouse Lab in Cambridge	Wellcome Sanger Institute for the COVID-19 Genomics UK (COG-UK) consortium	Rob Howes, The Lighthouse Lab in Cambridge and Alex Alderton, Roberto Amato, Sonia Goncalves, Ewan Harrison, David K. Jackson, Ian Johnston, Dominic Kwiatkowski, Cordelia Langford, John Sillitoe on behalf of the Wellcome Sanger Institute COVID-19 Surveillance Team (http://www.sanger.ac.uk/covid-team)
EPI_ISL_623573	Lighthouse Lab in Milton Keynes	Wellcome Sanger Institute for the COVID-19 Genomics UK (COG-UK) consortium	The Lighthouse Lab in Milton Keynes and Alex Alderton, Roberto Amato, Sonia Goncalves, Ewan Harrison, David K. Jackson, Ian Johnston, Dominic Kwiatkowski, Cordelia Langford, John Sillitoe on behalf of the Wellcome Sanger Institute COVID-19 Surveillance Team (http://www.sanger.ac.uk/covid-team)
EPI_ISL_623574, EPI_ISL_623575, EPI_ISL_623576	Lighthouse Lab in Cambridge	Wellcome Sanger Institute for the COVID-19 Genomics UK (COG-UK) consortium	Rob Howes, The Lighthouse Lab in Cambridge and Alex Alderton, Roberto Amato, Sonia Goncalves, Ewan Harrison, David K. Jackson, Ian Johnston, Dominic Kwiatkowski, Cordelia Langford, John Sillitoe on behalf of the Wellcome Sanger Institute COVID-19 Surveillance Team (http://www.sanger.ac.uk/covid-team)
EPI_ISL_623577, EPI_ISL_623579, EPI_ISL_623580, EPI_ISL_623582, EPI_ISL_623583	Lighthouse Lab in Milton Keynes	Wellcome Sanger Institute for the COVID-19 Genomics UK (COG-UK) consortium	The Lighthouse Lab in Milton Keynes and Alex Alderton, Roberto Amato, Sonia Goncalves, Ewan Harrison, David K. Jackson, Ian Johnston, Dominic Kwiatkowski, Cordelia Langford, John Sillitoe on behalf of the Wellcome Sanger Institute COVID-19 Surveillance Team (http://www.sanger.ac.uk/covid-team)
EPI_ISL_623584	Lighthouse Lab in Cambridge	Wellcome Sanger Institute for the COVID-19 Genomics UK (COG-UK) consortium	Rob Howes, The Lighthouse Lab in Cambridge and Alex Alderton, Roberto Amato, Sonia Goncalves, Ewan Harrison, David K. Jackson, Ian Johnston, Dominic Kwiatkowski, Cordelia Langford, John Sillitoe on behalf of the Wellcome Sanger Institute COVID-19 Surveillance Team (http://www.sanger.ac.uk/covid-team)
EPI_ISL_623586	Lighthouse Lab in Milton Keynes	Wellcome Sanger Institute for the COVID-19 Genomics UK (COG-UK) consortium	The Lighthouse Lab in Milton Keynes and Alex Alderton, Roberto Amato, Sonia Goncalves, Ewan Harrison, David K. Jackson, Ian Johnston, Dominic Kwiatkowski, Cordelia Langford, John Sillitoe on behalf of the Wellcome Sanger Institute COVID-19 Surveillance Team (http://www.sanger.ac.uk/covid-team)
EPI_ISL_623587	Lighthouse Lab in Cambridge	Wellcome Sanger Institute for the COVID-19 Genomics UK (COG-UK) consortium	Rob Howes, The Lighthouse Lab in Cambridge and Alex Alderton, Roberto Amato, Sonia Goncalves, Ewan Harrison, David K. Jackson, Ian Johnston, Dominic Kwiatkowski, Cordelia Langford, John Sillitoe on behalf of the Wellcome Sanger Institute COVID-19 Surveillance Team (http://www.sanger.ac.uk/covid-team)
EPI_ISL_623588, EPI_ISL_623591	Lighthouse Lab in Milton Keynes	Wellcome Sanger Institute for the COVID-19 Genomics UK (COG-UK) consortium	The Lighthouse Lab in Milton Keynes and Alex Alderton, Roberto Amato, Sonia Goncalves, Ewan Harrison, David K. Jackson, Ian Johnston, Dominic Kwiatkowski, Cordelia Langford, John Sillitoe on behalf of the Wellcome Sanger Institute COVID-19 Surveillance Team (http://www.sanger.ac.uk/covid-team)
EPI_ISL_623593, EPI_ISL_623595	Lighthouse Lab in Cambridge	Wellcome Sanger Institute for the COVID-19 Genomics UK (COG-UK) consortium	Rob Howes, The Lighthouse Lab in Cambridge and Alex Alderton, Roberto Amato, Sonia Goncalves, Ewan Harrison, David K. Jackson, Ian Johnston, Dominic Kwiatkowski, Cordelia Langford, John Sillitoe on behalf of the Wellcome Sanger Institute COVID-19 Surveillance Team (http://www.sanger.ac.uk/covid-team)
EPI_ISL_623596, EPI_ISL_623598, EPI_ISL_623599	Lighthouse Lab in Milton Keynes	Wellcome Sanger Institute for the COVID-19 Genomics UK (COG-UK) consortium	The Lighthouse Lab in Milton Keynes and Alex Alderton, Roberto Amato, Sonia Goncalves, Ewan Harrison, David K. Jackson, Ian Johnston, Dominic Kwiatkowski, Cordelia Langford, John Sillitoe on behalf of the Wellcome Sanger Institute COVID-19 Surveillance Team (http://www.sanger.ac.uk/covid-team)
EPI_ISL_623600	Lighthouse Lab in Cambridge	Wellcome Sanger Institute for the COVID-19 Genomics UK (COG-UK) consortium	Rob Howes, The Lighthouse Lab in Cambridge and Alex Alderton, Roberto Amato, Sonia Goncalves, Ewan Harrison, David K. Jackson, Ian Johnston, Dominic Kwiatkowski, Cordelia Langford, John Sillitoe on behalf of the Wellcome Sanger Institute COVID-19 Surveillance Team (http://www.sanger.ac.uk/covid-team)
EPI_ISL_623601, EPI_ISL_623603, EPI_ISL_623604	Lighthouse Lab in Milton Keynes	Wellcome Sanger Institute for the COVID-19 Genomics UK (COG-UK) consortium	The Lighthouse Lab in Milton Keynes and Alex Alderton, Roberto Amato, Sonia Goncalves, Ewan Harrison, David K. Jackson, Ian Johnston, Dominic Kwiatkowski, Cordelia Langford, John Sillitoe on behalf of the Wellcome Sanger Institute COVID-19 Surveillance Team (http://www.sanger.ac.uk/covid-team)
EPI_ISL_623605	Lighthouse Lab in Cambridge	Wellcome Sanger Institute for the COVID-19 Genomics UK (COG-UK) consortium	Rob Howes, The Lighthouse Lab in Cambridge and Alex Alderton, Roberto Amato, Sonia Goncalves, Ewan Harrison, David K. Jackson, Ian Johnston, Dominic Kwiatkowski, Cordelia Langford, John Sillitoe on behalf of the Wellcome Sanger Institute COVID-19 Surveillance Team (http://www.sanger.ac.uk/covid-team)
EPI_ISL_623606, EPI_ISL_623607	Lighthouse Lab in Milton Keynes	Wellcome Sanger Institute for the COVID-19 Genomics UK (COG-UK) consortium	The Lighthouse Lab in Milton Keynes and Alex Alderton, Roberto Amato, Sonia Goncalves, Ewan Harrison, David K. Jackson, Ian Johnston, Dominic Kwiatkowski, Cordelia Langford, John Sillitoe on behalf of the Wellcome Sanger Institute COVID-19 Surveillance Team (http://www.sanger.ac.uk/covid-team)
EPI_ISL_623608, EPI_ISL_623609	Lighthouse Lab in Cambridge	Wellcome Sanger Institute for the COVID-19 Genomics UK (COG-UK) consortium	Rob Howes, The Lighthouse Lab in Cambridge and Alex Alderton, Roberto Amato, Sonia Goncalves, Ewan Harrison, David K. Jackson, Ian Johnston, Dominic Kwiatkowski, Cordelia Langford, John Sillitoe on behalf of the Wellcome Sanger Institute COVID-19 Surveillance Team (http://www.sanger.ac.uk/covid-team)
EPI_ISL_623610	Lighthouse Lab in Milton Keynes	Wellcome Sanger Institute for the COVID-19 Genomics UK (COG-UK) consortium	The Lighthouse Lab in Milton Keynes and Alex Alderton, Roberto Amato, Sonia Goncalves, Ewan Harrison, David K. Jackson, Ian Johnston, Dominic Kwiatkowski, Cordelia Langford, John Sillitoe on behalf of the Wellcome Sanger Institute COVID-19 Surveillance Team (http://www.sanger.ac.uk/covid-team)
EPI_ISL_623611, EPI_ISL_623612	Lighthouse Lab in Cambridge	Wellcome Sanger Institute for the COVID-19 Genomics UK (COG-UK) consortium	Rob Howes, The Lighthouse Lab in Cambridge and Alex Alderton, Roberto Amato, Sonia Goncalves, Ewan Harrison, David K. Jackson, Ian Johnston, Dominic Kwiatkowski, Cordelia Langford, John Sillitoe on behalf of the Wellcome Sanger Institute COVID-19 Surveillance Team

			(http://www.sanger.ac.uk/covid-team)
EPI_ISL_623614, EPI_ISL_623615	Lighthouse Lab in Milton Keynes	Wellcome Sanger Institute for the COVID-19 Genomics UK (COG-UK) consortium	The Lighthouse Lab in Milton Keynes and Alex Alderton, Roberto Amato, Sonia Goncalves, Ewan Harrison, David K. Jackson, Ian Johnston, Dominic Kwiatkowski, Cordelia Langford, John Sillitoe on behalf of the Wellcome Sanger Institute COVID-19 Surveillance Team (http://www.sanger.ac.uk/covid-team)
EPI_ISL_623616, EPI_ISL_623617, EPI_ISL_623619	Lighthouse Lab in Cambridge	Wellcome Sanger Institute for the COVID-19 Genomics UK (COG-UK) consortium	Rob Howes, The Lighthouse Lab in Cambridge and Alex Alderton, Roberto Amato, Sonia Goncalves, Ewan Harrison, David K. Jackson, Ian Johnston, Dominic Kwiatkowski, Cordelia Langford, John Sillitoe on behalf of the Wellcome Sanger Institute COVID-19 Surveillance Team (http://www.sanger.ac.uk/covid-team)
EPI_ISL_623620, EPI_ISL_623621, EPI_ISL_623622, EPI_ISL_623623, EPI_ISL_623625	Lighthouse Lab in Milton Keynes	Wellcome Sanger Institute for the COVID-19 Genomics UK (COG-UK) consortium	The Lighthouse Lab in Milton Keynes and Alex Alderton, Roberto Amato, Sonia Goncalves, Ewan Harrison, David K. Jackson, Ian Johnston, Dominic Kwiatkowski, Cordelia Langford, John Sillitoe on behalf of the Wellcome Sanger Institute COVID-19 Surveillance Team (http://www.sanger.ac.uk/covid-team)
EPI_ISL_623626	Lighthouse Lab in Cambridge	Wellcome Sanger Institute for the COVID-19 Genomics UK (COG-UK) consortium	Rob Howes, The Lighthouse Lab in Cambridge and Alex Alderton, Roberto Amato, Sonia Goncalves, Ewan Harrison, David K. Jackson, Ian Johnston, Dominic Kwiatkowski, Cordelia Langford, John Sillitoe on behalf of the Wellcome Sanger Institute COVID-19 Surveillance Team (http://www.sanger.ac.uk/covid-team)
EPI_ISL_623627, EPI_ISL_623628, EPI_ISL_623629, EPI_ISL_623631, EPI_ISL_623632, EPI_ISL_623634, EPI_ISL_623636, EPI_ISL_623637, EPI_ISL_623638	Lighthouse Lab in Milton Keynes	Wellcome Sanger Institute for the COVID-19 Genomics UK (COG-UK) consortium	The Lighthouse Lab in Milton Keynes and Alex Alderton, Roberto Amato, Sonia Goncalves, Ewan Harrison, David K. Jackson, Ian Johnston, Dominic Kwiatkowski, Cordelia Langford, John Sillitoe on behalf of the Wellcome Sanger Institute COVID-19 Surveillance Team (http://www.sanger.ac.uk/covid-team)
EPI_ISL_623640, EPI_ISL_623641	Lighthouse Lab in Cambridge	Wellcome Sanger Institute for the COVID-19 Genomics UK (COG-UK) consortium	Rob Howes, The Lighthouse Lab in Cambridge and Alex Alderton, Roberto Amato, Sonia Goncalves, Ewan Harrison, David K. Jackson, Ian Johnston, Dominic Kwiatkowski, Cordelia Langford, John Sillitoe on behalf of the Wellcome Sanger Institute COVID-19 Surveillance Team (http://www.sanger.ac.uk/covid-team)
EPI_ISL_623642	Lighthouse Lab in Milton Keynes	Wellcome Sanger Institute for the COVID-19 Genomics UK (COG-UK) consortium	The Lighthouse Lab in Milton Keynes and Alex Alderton, Roberto Amato, Sonia Goncalves, Ewan Harrison, David K. Jackson, Ian Johnston, Dominic Kwiatkowski, Cordelia Langford, John Sillitoe on behalf of the Wellcome Sanger Institute COVID-19 Surveillance Team (http://www.sanger.ac.uk/covid-team)
EPI_ISL_623643, EPI_ISL_623644, EPI_ISL_623645	Lighthouse Lab in Cambridge	Wellcome Sanger Institute for the COVID-19 Genomics UK (COG-UK) consortium	Rob Howes, The Lighthouse Lab in Cambridge and Alex Alderton, Roberto Amato, Sonia Goncalves, Ewan Harrison, David K. Jackson, Ian Johnston, Dominic Kwiatkowski, Cordelia Langford, John Sillitoe on behalf of the Wellcome Sanger Institute COVID-19 Surveillance Team (http://www.sanger.ac.uk/covid-team)
EPI_ISL_623646	Lighthouse Lab in Milton Keynes	Wellcome Sanger Institute for the COVID-19 Genomics UK (COG-UK) consortium	The Lighthouse Lab in Milton Keynes and Alex Alderton, Roberto Amato, Sonia Goncalves, Ewan Harrison, David K. Jackson, Ian Johnston, Dominic Kwiatkowski, Cordelia Langford, John Sillitoe on behalf of the Wellcome Sanger Institute COVID-19 Surveillance Team (http://www.sanger.ac.uk/covid-team)
EPI_ISL_623647	Lighthouse Lab in Cambridge	Wellcome Sanger Institute for the COVID-19 Genomics UK (COG-UK) consortium	Rob Howes, The Lighthouse Lab in Cambridge and Alex Alderton, Roberto Amato, Sonia Goncalves, Ewan Harrison, David K. Jackson, Ian Johnston, Dominic Kwiatkowski, Cordelia Langford, John Sillitoe on behalf of the Wellcome Sanger Institute COVID-19 Surveillance Team (http://www.sanger.ac.uk/covid-team)
EPI_ISL_623648, EPI_ISL_623649	Lighthouse Lab in Milton Keynes	Wellcome Sanger Institute for the COVID-19 Genomics UK (COG-UK) consortium	The Lighthouse Lab in Milton Keynes and Alex Alderton, Roberto Amato, Sonia Goncalves, Ewan Harrison, David K. Jackson, Ian Johnston, Dominic Kwiatkowski, Cordelia Langford, John Sillitoe on behalf of the Wellcome Sanger Institute COVID-19 Surveillance Team (http://www.sanger.ac.uk/covid-team)
EPI_ISL_623650, EPI_ISL_623651, EPI_ISL_623652, EPI_ISL_623654	Lighthouse Lab in Cambridge	Wellcome Sanger Institute for the COVID-19 Genomics UK (COG-UK) consortium	Rob Howes, The Lighthouse Lab in Cambridge and Alex Alderton, Roberto Amato, Sonia Goncalves, Ewan Harrison, David K. Jackson, Ian Johnston, Dominic Kwiatkowski, Cordelia Langford, John Sillitoe on behalf of the Wellcome Sanger Institute COVID-19 Surveillance Team (http://www.sanger.ac.uk/covid-team)
EPI_ISL_623656	Lighthouse Lab in Milton Keynes	Wellcome Sanger Institute for the COVID-19 Genomics UK (COG-UK) consortium	The Lighthouse Lab in Milton Keynes and Alex Alderton, Roberto Amato, Sonia Goncalves, Ewan Harrison, David K. Jackson, Ian Johnston, Dominic Kwiatkowski, Cordelia Langford, John Sillitoe on behalf of the Wellcome Sanger Institute COVID-19 Surveillance Team (http://www.sanger.ac.uk/covid-team)
EPI_ISL_623657, EPI_ISL_623658	Lighthouse Lab in Cambridge	Wellcome Sanger Institute for the COVID-19 Genomics UK (COG-UK) consortium	Rob Howes, The Lighthouse Lab in Cambridge and Alex Alderton, Roberto Amato, Sonia Goncalves, Ewan Harrison, David K. Jackson, Ian Johnston, Dominic Kwiatkowski, Cordelia Langford, John Sillitoe on behalf of the Wellcome Sanger Institute COVID-19 Surveillance Team (http://www.sanger.ac.uk/covid-team)
EPI_ISL_623659, EPI_ISL_623660	Lighthouse Lab in Milton Keynes	Wellcome Sanger Institute for the COVID-19 Genomics UK (COG-UK) consortium	The Lighthouse Lab in Milton Keynes and Alex Alderton, Roberto Amato, Sonia Goncalves, Ewan Harrison, David K. Jackson, Ian Johnston, Dominic Kwiatkowski, Cordelia Langford, John Sillitoe on behalf of the Wellcome Sanger Institute COVID-19 Surveillance Team (http://www.sanger.ac.uk/covid-team)
EPI_ISL_623662, EPI_ISL_623663, EPI_ISL_623665, EPI_ISL_623666, EPI_ISL_623667	Lighthouse Lab in Cambridge	Wellcome Sanger Institute for the COVID-19 Genomics UK (COG-UK) consortium	Rob Howes, The Lighthouse Lab in Cambridge and Alex Alderton, Roberto Amato, Sonia Goncalves, Ewan Harrison, David K. Jackson, Ian Johnston, Dominic Kwiatkowski, Cordelia Langford, John Sillitoe on behalf of the Wellcome Sanger Institute COVID-19 Surveillance Team (http://www.sanger.ac.uk/covid-team)
EPI_ISL_623668, EPI_ISL_623669, EPI_ISL_623670, EPI_ISL_623671, EPI_ISL_623672, EPI_ISL_623673	Lighthouse Lab in Milton Keynes	Wellcome Sanger Institute for the COVID-19 Genomics UK (COG-UK) consortium	The Lighthouse Lab in Milton Keynes and Alex Alderton, Roberto Amato, Sonia Goncalves, Ewan Harrison, David K. Jackson, Ian Johnston, Dominic Kwiatkowski, Cordelia Langford, John Sillitoe on behalf of the Wellcome Sanger Institute COVID-19 Surveillance Team (http://www.sanger.ac.uk/covid-team)
EPI_ISL_623675	Lighthouse Lab in Cambridge	Wellcome Sanger Institute for the COVID-19 Genomics UK (COG-UK) consortium	Rob Howes, The Lighthouse Lab in Cambridge and Alex Alderton, Roberto Amato, Sonia Goncalves, Ewan Harrison, David K. Jackson, Ian Johnston, Dominic Kwiatkowski, Cordelia Langford, John Sillitoe on behalf of the Wellcome Sanger Institute COVID-19 Surveillance Team (http://www.sanger.ac.uk/covid-team)
EPI_ISL_623676	Lighthouse Lab in Milton Keynes	Wellcome Sanger Institute for the COVID-19 Genomics UK (COG-UK) consortium	The Lighthouse Lab in Milton Keynes and Alex Alderton, Roberto Amato, Sonia Goncalves, Ewan Harrison, David K. Jackson, Ian Johnston, Dominic Kwiatkowski, Cordelia Langford, John Sillitoe on behalf of the Wellcome Sanger Institute COVID-19 Surveillance Team (http://www.sanger.ac.uk/covid-team)
EPI_ISL_623679, EPI_ISL_623680	Lighthouse Lab in Cambridge	Wellcome Sanger Institute for the COVID-19 Genomics UK (COG-UK) consortium	Rob Howes, The Lighthouse Lab in Cambridge and Alex Alderton, Roberto Amato, Sonia Goncalves, Ewan Harrison, David K. Jackson, Ian Johnston, Dominic Kwiatkowski, Cordelia Langford, John Sillitoe on behalf of the Wellcome Sanger Institute COVID-19 Surveillance Team (http://www.sanger.ac.uk/covid-team)
EPI_ISL_623681, EPI_ISL_623682	Lighthouse Lab in Milton Keynes	Wellcome Sanger Institute for the COVID-19 Genomics UK (COG-UK) consortium	The Lighthouse Lab in Milton Keynes and Alex Alderton, Roberto Amato, Sonia Goncalves, Ewan Harrison, David K. Jackson, Ian Johnston, Dominic Kwiatkowski, Cordelia Langford, John Sillitoe on behalf of the Wellcome Sanger Institute COVID-19 Surveillance Team (http://www.sanger.ac.uk/covid-team)
EPI_ISL_623683	Lighthouse Lab in Cambridge	Wellcome Sanger Institute for the COVID-19 Genomics UK (COG-UK) consortium	Rob Howes, The Lighthouse Lab in Cambridge and Alex Alderton, Roberto Amato, Sonia Goncalves, Ewan Harrison, David K. Jackson, Ian Johnston, Dominic Kwiatkowski, Cordelia Langford, John Sillitoe on behalf of the Wellcome Sanger Institute COVID-19 Surveillance Team (http://www.sanger.ac.uk/covid-team)
EPI_ISL_623684, EPI_ISL_623686, EPI_ISL_623687	Lighthouse Lab in Milton Keynes	Wellcome Sanger Institute for the COVID-19 Genomics UK (COG-UK) consortium	The Lighthouse Lab in Milton Keynes and Alex Alderton, Roberto Amato, Sonia Goncalves, Ewan Harrison, David K. Jackson, Ian Johnston, Dominic Kwiatkowski, Cordelia Langford, John Sillitoe on behalf of the Wellcome Sanger Institute COVID-19 Surveillance Team (http://www.sanger.ac.uk/covid-team)
EPI_ISL_623688	Lighthouse Lab in Cambridge	Wellcome Sanger Institute for the COVID-19 Genomics UK (COG-UK) consortium	Rob Howes, The Lighthouse Lab in Cambridge and Alex Alderton, Roberto Amato, Sonia Goncalves, Ewan Harrison, David K. Jackson, Ian Johnston, Dominic Kwiatkowski, Cordelia Langford, John Sillitoe on behalf of the Wellcome Sanger Institute COVID-19 Surveillance Team (http://www.sanger.ac.uk/covid-team)
EPI_ISL_623689	Lighthouse Lab in Milton Keynes	Wellcome Sanger Institute for the COVID-19 Genomics UK (COG-UK) consortium	The Lighthouse Lab in Milton Keynes and Alex Alderton, Roberto Amato, Sonia Goncalves, Ewan Harrison, David K. Jackson, Ian Johnston, Dominic Kwiatkowski, Cordelia Langford, John Sillitoe on behalf of the Wellcome Sanger Institute COVID-19 Surveillance Team

			(http://www.sanger.ac.uk/covid-team)
EPI_ISL_623690	Lighthouse Lab in Cambridge	Wellcome Sanger Institute for the COVID-19 Genomics UK (COG-UK) consortium	Rob Howes, The Lighthouse Lab in Cambridge and Alex Alderton, Roberto Amato, Sonia Goncalves, Ewan Harrison, David K. Jackson, Ian Johnston, Dominic Kwiatkowski, Cordelia Langford, John Sillitoe on behalf of the Wellcome Sanger Institute COVID-19 Surveillance Team (http://www.sanger.ac.uk/covid-team)
EPI_ISL_623691, EPI_ISL_623692, EPI_ISL_623693	Lighthouse Lab in Milton Keynes	Wellcome Sanger Institute for the COVID-19 Genomics UK (COG-UK) consortium	The Lighthouse Lab in Milton Keynes and Alex Alderton, Roberto Amato, Sonia Goncalves, Ewan Harrison, David K. Jackson, Ian Johnston, Dominic Kwiatkowski, Cordelia Langford, John Sillitoe on behalf of the Wellcome Sanger Institute COVID-19 Surveillance Team (http://www.sanger.ac.uk/covid-team)
EPI_ISL_623694	Lighthouse Lab in Cambridge	Wellcome Sanger Institute for the COVID-19 Genomics UK (COG-UK) consortium	Rob Howes, The Lighthouse Lab in Cambridge and Alex Alderton, Roberto Amato, Sonia Goncalves, Ewan Harrison, David K. Jackson, Ian Johnston, Dominic Kwiatkowski, Cordelia Langford, John Sillitoe on behalf of the Wellcome Sanger Institute COVID-19 Surveillance Team (http://www.sanger.ac.uk/covid-team)
EPI_ISL_623695, EPI_ISL_623697, EPI_ISL_623698, EPI_ISL_623699	Lighthouse Lab in Milton Keynes	Wellcome Sanger Institute for the COVID-19 Genomics UK (COG-UK) consortium	The Lighthouse Lab in Milton Keynes and Alex Alderton, Roberto Amato, Sonia Goncalves, Ewan Harrison, David K. Jackson, Ian Johnston, Dominic Kwiatkowski, Cordelia Langford, John Sillitoe on behalf of the Wellcome Sanger Institute COVID-19 Surveillance Team (http://www.sanger.ac.uk/covid-team)
EPI_ISL_623702, EPI_ISL_623703	Lighthouse Lab in Cambridge	Wellcome Sanger Institute for the COVID-19 Genomics UK (COG-UK) consortium	Rob Howes, The Lighthouse Lab in Cambridge and Alex Alderton, Roberto Amato, Sonia Goncalves, Ewan Harrison, David K. Jackson, Ian Johnston, Dominic Kwiatkowski, Cordelia Langford, John Sillitoe on behalf of the Wellcome Sanger Institute COVID-19 Surveillance Team (http://www.sanger.ac.uk/covid-team)
EPI_ISL_623704, EPI_ISL_623705, EPI_ISL_623706, EPI_ISL_623707, EPI_ISL_623708, EPI_ISL_623709, EPI_ISL_623711	Lighthouse Lab in Milton Keynes	Wellcome Sanger Institute for the COVID-19 Genomics UK (COG-UK) consortium	The Lighthouse Lab in Milton Keynes and Alex Alderton, Roberto Amato, Sonia Goncalves, Ewan Harrison, David K. Jackson, Ian Johnston, Dominic Kwiatkowski, Cordelia Langford, John Sillitoe on behalf of the Wellcome Sanger Institute COVID-19 Surveillance Team (http://www.sanger.ac.uk/covid-team)
EPI_ISL_623712	Lighthouse Lab in Cambridge	Wellcome Sanger Institute for the COVID-19 Genomics UK (COG-UK) consortium	Rob Howes, The Lighthouse Lab in Cambridge and Alex Alderton, Roberto Amato, Sonia Goncalves, Ewan Harrison, David K. Jackson, Ian Johnston, Dominic Kwiatkowski, Cordelia Langford, John Sillitoe on behalf of the Wellcome Sanger Institute COVID-19 Surveillance Team (http://www.sanger.ac.uk/covid-team)
EPI_ISL_623714, EPI_ISL_623715	Lighthouse Lab in Milton Keynes	Wellcome Sanger Institute for the COVID-19 Genomics UK (COG-UK) consortium	The Lighthouse Lab in Milton Keynes and Alex Alderton, Roberto Amato, Sonia Goncalves, Ewan Harrison, David K. Jackson, Ian Johnston, Dominic Kwiatkowski, Cordelia Langford, John Sillitoe on behalf of the Wellcome Sanger Institute COVID-19 Surveillance Team (http://www.sanger.ac.uk/covid-team)
EPI_ISL_623717	Lighthouse Lab in Cambridge	Wellcome Sanger Institute for the COVID-19 Genomics UK (COG-UK) consortium	Rob Howes, The Lighthouse Lab in Cambridge and Alex Alderton, Roberto Amato, Sonia Goncalves, Ewan Harrison, David K. Jackson, Ian Johnston, Dominic Kwiatkowski, Cordelia Langford, John Sillitoe on behalf of the Wellcome Sanger Institute COVID-19 Surveillance Team (http://www.sanger.ac.uk/covid-team)
EPI_ISL_623718, EPI_ISL_623720	Lighthouse Lab in Milton Keynes	Wellcome Sanger Institute for the COVID-19 Genomics UK (COG-UK) consortium	The Lighthouse Lab in Milton Keynes and Alex Alderton, Roberto Amato, Sonia Goncalves, Ewan Harrison, David K. Jackson, Ian Johnston, Dominic Kwiatkowski, Cordelia Langford, John Sillitoe on behalf of the Wellcome Sanger Institute COVID-19 Surveillance Team (http://www.sanger.ac.uk/covid-team)
EPI_ISL_623721	Lighthouse Lab in Cambridge	Wellcome Sanger Institute for the COVID-19 Genomics UK (COG-UK) consortium	Rob Howes, The Lighthouse Lab in Cambridge and Alex Alderton, Roberto Amato, Sonia Goncalves, Ewan Harrison, David K. Jackson, Ian Johnston, Dominic Kwiatkowski, Cordelia Langford, John Sillitoe on behalf of the Wellcome Sanger Institute COVID-19 Surveillance Team (http://www.sanger.ac.uk/covid-team)
EPI_ISL_623722, EPI_ISL_623723	Lighthouse Lab in Milton Keynes	Wellcome Sanger Institute for the COVID-19 Genomics UK (COG-UK) consortium	The Lighthouse Lab in Milton Keynes and Alex Alderton, Roberto Amato, Sonia Goncalves, Ewan Harrison, David K. Jackson, Ian Johnston, Dominic Kwiatkowski, Cordelia Langford, John Sillitoe on behalf of the Wellcome Sanger Institute COVID-19 Surveillance Team (http://www.sanger.ac.uk/covid-team)
EPI_ISL_623725, EPI_ISL_623726	Lighthouse Lab in Cambridge	Wellcome Sanger Institute for the COVID-19 Genomics UK (COG-UK) consortium	Rob Howes, The Lighthouse Lab in Cambridge and Alex Alderton, Roberto Amato, Sonia Goncalves, Ewan Harrison, David K. Jackson, Ian Johnston, Dominic Kwiatkowski, Cordelia Langford, John Sillitoe on behalf of the Wellcome Sanger Institute COVID-19 Surveillance Team (http://www.sanger.ac.uk/covid-team)
EPI_ISL_623727, EPI_ISL_623728, EPI_ISL_623729	Lighthouse Lab in Milton Keynes	Wellcome Sanger Institute for the COVID-19 Genomics UK (COG-UK) consortium	The Lighthouse Lab in Milton Keynes and Alex Alderton, Roberto Amato, Sonia Goncalves, Ewan Harrison, David K. Jackson, Ian Johnston, Dominic Kwiatkowski, Cordelia Langford, John Sillitoe on behalf of the Wellcome Sanger Institute COVID-19 Surveillance Team (http://www.sanger.ac.uk/covid-team)
EPI_ISL_623731, EPI_ISL_623733	Lighthouse Lab in Cambridge	Wellcome Sanger Institute for the COVID-19 Genomics UK (COG-UK) consortium	Rob Howes, The Lighthouse Lab in Cambridge and Alex Alderton, Roberto Amato, Sonia Goncalves, Ewan Harrison, David K. Jackson, Ian Johnston, Dominic Kwiatkowski, Cordelia Langford, John Sillitoe on behalf of the Wellcome Sanger Institute COVID-19 Surveillance Team (http://www.sanger.ac.uk/covid-team)
EPI_ISL_623734, EPI_ISL_623737, EPI_ISL_623739	Lighthouse Lab in Milton Keynes	Wellcome Sanger Institute for the COVID-19 Genomics UK (COG-UK) consortium	The Lighthouse Lab in Milton Keynes and Alex Alderton, Roberto Amato, Sonia Goncalves, Ewan Harrison, David K. Jackson, Ian Johnston, Dominic Kwiatkowski, Cordelia Langford, John Sillitoe on behalf of the Wellcome Sanger Institute COVID-19 Surveillance Team (http://www.sanger.ac.uk/covid-team)
EPI_ISL_623740, EPI_ISL_623742	Lighthouse Lab in Cambridge	Wellcome Sanger Institute for the COVID-19 Genomics UK (COG-UK) consortium	Rob Howes, The Lighthouse Lab in Cambridge and Alex Alderton, Roberto Amato, Sonia Goncalves, Ewan Harrison, David K. Jackson, Ian Johnston, Dominic Kwiatkowski, Cordelia Langford, John Sillitoe on behalf of the Wellcome Sanger Institute COVID-19 Surveillance Team (http://www.sanger.ac.uk/covid-team)
EPI_ISL_623744, EPI_ISL_623745	Lighthouse Lab in Milton Keynes	Wellcome Sanger Institute for the COVID-19 Genomics UK (COG-UK) consortium	The Lighthouse Lab in Milton Keynes and Alex Alderton, Roberto Amato, Sonia Goncalves, Ewan Harrison, David K. Jackson, Ian Johnston, Dominic Kwiatkowski, Cordelia Langford, John Sillitoe on behalf of the Wellcome Sanger Institute COVID-19 Surveillance Team (http://www.sanger.ac.uk/covid-team)
EPI_ISL_623746	Lighthouse Lab in Cambridge	Wellcome Sanger Institute for the COVID-19 Genomics UK (COG-UK) consortium	Rob Howes, The Lighthouse Lab in Cambridge and Alex Alderton, Roberto Amato, Sonia Goncalves, Ewan Harrison, David K. Jackson, Ian Johnston, Dominic Kwiatkowski, Cordelia Langford, John Sillitoe on behalf of the Wellcome Sanger Institute COVID-19 Surveillance Team (http://www.sanger.ac.uk/covid-team)
EPI_ISL_623748	Lighthouse Lab in Milton Keynes	Wellcome Sanger Institute for the COVID-19 Genomics UK (COG-UK) consortium	The Lighthouse Lab in Milton Keynes and Alex Alderton, Roberto Amato, Sonia Goncalves, Ewan Harrison, David K. Jackson, Ian Johnston, Dominic Kwiatkowski, Cordelia Langford, John Sillitoe on behalf of the Wellcome Sanger Institute COVID-19 Surveillance Team (http://www.sanger.ac.uk/covid-team)
EPI_ISL_623749, EPI_ISL_623750, EPI_ISL_623751	Lighthouse Lab in Cambridge	Wellcome Sanger Institute for the COVID-19 Genomics UK (COG-UK) consortium	Rob Howes, The Lighthouse Lab in Cambridge and Alex Alderton, Roberto Amato, Sonia Goncalves, Ewan Harrison, David K. Jackson, Ian Johnston, Dominic Kwiatkowski, Cordelia Langford, John Sillitoe on behalf of the Wellcome Sanger Institute COVID-19 Surveillance Team (http://www.sanger.ac.uk/covid-team)
EPI_ISL_623752, EPI_ISL_623753	Lighthouse Lab in Milton Keynes	Wellcome Sanger Institute for the COVID-19 Genomics UK (COG-UK) consortium	The Lighthouse Lab in Milton Keynes and Alex Alderton, Roberto Amato, Sonia Goncalves, Ewan Harrison, David K. Jackson, Ian Johnston, Dominic Kwiatkowski, Cordelia Langford, John Sillitoe on behalf of the Wellcome Sanger Institute COVID-19 Surveillance Team (http://www.sanger.ac.uk/covid-team)
EPI_ISL_623754	Lighthouse Lab in Cambridge	Wellcome Sanger Institute for the COVID-19 Genomics UK (COG-UK) consortium	Rob Howes, The Lighthouse Lab in Cambridge and Alex Alderton, Roberto Amato, Sonia Goncalves, Ewan Harrison, David K. Jackson, Ian Johnston, Dominic Kwiatkowski, Cordelia Langford, John Sillitoe on behalf of the Wellcome Sanger Institute COVID-19 Surveillance Team (http://www.sanger.ac.uk/covid-team)
EPI_ISL_623755, EPI_ISL_623756, EPI_ISL_623757	Lighthouse Lab in Milton Keynes	Wellcome Sanger Institute for the COVID-19 Genomics UK (COG-UK) consortium	The Lighthouse Lab in Milton Keynes and Alex Alderton, Roberto Amato, Sonia Goncalves, Ewan Harrison, David K. Jackson, Ian Johnston, Dominic Kwiatkowski, Cordelia Langford, John Sillitoe on behalf of the Wellcome Sanger Institute COVID-19 Surveillance Team (http://www.sanger.ac.uk/covid-team)
EPI_ISL_623758, EPI_ISL_623759	Lighthouse Lab in Cambridge	Wellcome Sanger Institute for the COVID-19 Genomics UK (COG-UK) consortium	Rob Howes, The Lighthouse Lab in Cambridge and Alex Alderton, Roberto Amato, Sonia Goncalves, Ewan Harrison, David K. Jackson, Ian Johnston, Dominic Kwiatkowski, Cordelia Langford, John Sillitoe on behalf of the Wellcome Sanger Institute COVID-19 Surveillance Team

			(http://www.sanger.ac.uk/covid-team)
EPI_ISL_623760, EPI_ISL_623761	Lighthouse Lab in Milton Keynes	Wellcome Sanger Institute for the COVID-19 Genomics UK (COG-UK) consortium	The Lighthouse Lab in Milton Keynes and Alex Alderton, Roberto Amato, Sonia Goncalves, Ewan Harrison, David K. Jackson, Ian Johnston, Dominic Kwiatkowski, Cordelia Langford, John Sillitoe on behalf of the Wellcome Sanger Institute COVID-19 Surveillance Team (http://www.sanger.ac.uk/covid-team)
EPI_ISL_623762, EPI_ISL_623763, EPI_ISL_623764	Lighthouse Lab in Cambridge	Wellcome Sanger Institute for the COVID-19 Genomics UK (COG-UK) consortium	Rob Howes, The Lighthouse Lab in Cambridge and Alex Alderton, Roberto Amato, Sonia Goncalves, Ewan Harrison, David K. Jackson, Ian Johnston, Dominic Kwiatkowski, Cordelia Langford, John Sillitoe on behalf of the Wellcome Sanger Institute COVID-19 Surveillance Team (http://www.sanger.ac.uk/covid-team)
EPI_ISL_623766, EPI_ISL_623767, EPI_ISL_623768, EPI_ISL_623769, EPI_ISL_623770, EPI_ISL_623773	Lighthouse Lab in Milton Keynes	Wellcome Sanger Institute for the COVID-19 Genomics UK (COG-UK) consortium	The Lighthouse Lab in Milton Keynes and Alex Alderton, Roberto Amato, Sonia Goncalves, Ewan Harrison, David K. Jackson, Ian Johnston, Dominic Kwiatkowski, Cordelia Langford, John Sillitoe on behalf of the Wellcome Sanger Institute COVID-19 Surveillance Team (http://www.sanger.ac.uk/covid-team)
EPI_ISL_623774	Lighthouse Lab in Cambridge	Wellcome Sanger Institute for the COVID-19 Genomics UK (COG-UK) consortium	Rob Howes, The Lighthouse Lab in Cambridge and Alex Alderton, Roberto Amato, Sonia Goncalves, Ewan Harrison, David K. Jackson, Ian Johnston, Dominic Kwiatkowski, Cordelia Langford, John Sillitoe on behalf of the Wellcome Sanger Institute COVID-19 Surveillance Team (http://www.sanger.ac.uk/covid-team)
EPI_ISL_623777, EPI_ISL_623778, EPI_ISL_623780, EPI_ISL_623782, EPI_ISL_623783, EPI_ISL_623784	Lighthouse Lab in Milton Keynes	Wellcome Sanger Institute for the COVID-19 Genomics UK (COG-UK) consortium	The Lighthouse Lab in Milton Keynes and Alex Alderton, Roberto Amato, Sonia Goncalves, Ewan Harrison, David K. Jackson, Ian Johnston, Dominic Kwiatkowski, Cordelia Langford, John Sillitoe on behalf of the Wellcome Sanger Institute COVID-19 Surveillance Team (http://www.sanger.ac.uk/covid-team)
EPI_ISL_623785	Lighthouse Lab in Cambridge	Wellcome Sanger Institute for the COVID-19 Genomics UK (COG-UK) consortium	Rob Howes, The Lighthouse Lab in Cambridge and Alex Alderton, Roberto Amato, Sonia Goncalves, Ewan Harrison, David K. Jackson, Ian Johnston, Dominic Kwiatkowski, Cordelia Langford, John Sillitoe on behalf of the Wellcome Sanger Institute COVID-19 Surveillance Team (http://www.sanger.ac.uk/covid-team)
EPI_ISL_623786, EPI_ISL_623787	Lighthouse Lab in Milton Keynes	Wellcome Sanger Institute for the COVID-19 Genomics UK (COG-UK) consortium	The Lighthouse Lab in Milton Keynes and Alex Alderton, Roberto Amato, Sonia Goncalves, Ewan Harrison, David K. Jackson, Ian Johnston, Dominic Kwiatkowski, Cordelia Langford, John Sillitoe on behalf of the Wellcome Sanger Institute COVID-19 Surveillance Team (http://www.sanger.ac.uk/covid-team)
EPI_ISL_623788	Lighthouse Lab in Cambridge	Wellcome Sanger Institute for the COVID-19 Genomics UK (COG-UK) consortium	Rob Howes, The Lighthouse Lab in Cambridge and Alex Alderton, Roberto Amato, Sonia Goncalves, Ewan Harrison, David K. Jackson, Ian Johnston, Dominic Kwiatkowski, Cordelia Langford, John Sillitoe on behalf of the Wellcome Sanger Institute COVID-19 Surveillance Team (http://www.sanger.ac.uk/covid-team)
EPI_ISL_623789	Lighthouse Lab in Milton Keynes	Wellcome Sanger Institute for the COVID-19 Genomics UK (COG-UK) consortium	The Lighthouse Lab in Milton Keynes and Alex Alderton, Roberto Amato, Sonia Goncalves, Ewan Harrison, David K. Jackson, Ian Johnston, Dominic Kwiatkowski, Cordelia Langford, John Sillitoe on behalf of the Wellcome Sanger Institute COVID-19 Surveillance Team (http://www.sanger.ac.uk/covid-team)
EPI_ISL_623790, EPI_ISL_623791	Lighthouse Lab in Cambridge	Wellcome Sanger Institute for the COVID-19 Genomics UK (COG-UK) consortium	Rob Howes, The Lighthouse Lab in Cambridge and Alex Alderton, Roberto Amato, Sonia Goncalves, Ewan Harrison, David K. Jackson, Ian Johnston, Dominic Kwiatkowski, Cordelia Langford, John Sillitoe on behalf of the Wellcome Sanger Institute COVID-19 Surveillance Team (http://www.sanger.ac.uk/covid-team)
EPI_ISL_623792	Lighthouse Lab in Milton Keynes	Wellcome Sanger Institute for the COVID-19 Genomics UK (COG-UK) consortium	The Lighthouse Lab in Milton Keynes and Alex Alderton, Roberto Amato, Sonia Goncalves, Ewan Harrison, David K. Jackson, Ian Johnston, Dominic Kwiatkowski, Cordelia Langford, John Sillitoe on behalf of the Wellcome Sanger Institute COVID-19 Surveillance Team (http://www.sanger.ac.uk/covid-team)
EPI_ISL_623793, EPI_ISL_623794	Lighthouse Lab in Cambridge	Wellcome Sanger Institute for the COVID-19 Genomics UK (COG-UK) consortium	Rob Howes, The Lighthouse Lab in Cambridge and Alex Alderton, Roberto Amato, Sonia Goncalves, Ewan Harrison, David K. Jackson, Ian Johnston, Dominic Kwiatkowski, Cordelia Langford, John Sillitoe on behalf of the Wellcome Sanger Institute COVID-19 Surveillance Team (http://www.sanger.ac.uk/covid-team)
EPI_ISL_623795	Lighthouse Lab in Milton Keynes	Wellcome Sanger Institute for the COVID-19 Genomics UK (COG-UK) consortium	The Lighthouse Lab in Milton Keynes and Alex Alderton, Roberto Amato, Sonia Goncalves, Ewan Harrison, David K. Jackson, Ian Johnston, Dominic Kwiatkowski, Cordelia Langford, John Sillitoe on behalf of the Wellcome Sanger Institute COVID-19 Surveillance Team (http://www.sanger.ac.uk/covid-team)
EPI_ISL_623797, EPI_ISL_623798, EPI_ISL_623800, EPI_ISL_623802	Lighthouse Lab in Cambridge	Wellcome Sanger Institute for the COVID-19 Genomics UK (COG-UK) consortium	Rob Howes, The Lighthouse Lab in Cambridge and Alex Alderton, Roberto Amato, Sonia Goncalves, Ewan Harrison, David K. Jackson, Ian Johnston, Dominic Kwiatkowski, Cordelia Langford, John Sillitoe on behalf of the Wellcome Sanger Institute COVID-19 Surveillance Team (http://www.sanger.ac.uk/covid-team)
EPI_ISL_623803, EPI_ISL_623804, EPI_ISL_623805	Lighthouse Lab in Milton Keynes	Wellcome Sanger Institute for the COVID-19 Genomics UK (COG-UK) consortium	The Lighthouse Lab in Milton Keynes and Alex Alderton, Roberto Amato, Sonia Goncalves, Ewan Harrison, David K. Jackson, Ian Johnston, Dominic Kwiatkowski, Cordelia Langford, John Sillitoe on behalf of the Wellcome Sanger Institute COVID-19 Surveillance Team (http://www.sanger.ac.uk/covid-team)
EPI_ISL_623806	Lighthouse Lab in Cambridge	Wellcome Sanger Institute for the COVID-19 Genomics UK (COG-UK) consortium	Rob Howes, The Lighthouse Lab in Cambridge and Alex Alderton, Roberto Amato, Sonia Goncalves, Ewan Harrison, David K. Jackson, Ian Johnston, Dominic Kwiatkowski, Cordelia Langford, John Sillitoe on behalf of the Wellcome Sanger Institute COVID-19 Surveillance Team (http://www.sanger.ac.uk/covid-team)
EPI_ISL_623807	Lighthouse Lab in Milton Keynes	Wellcome Sanger Institute for the COVID-19 Genomics UK (COG-UK) consortium	The Lighthouse Lab in Milton Keynes and Alex Alderton, Roberto Amato, Sonia Goncalves, Ewan Harrison, David K. Jackson, Ian Johnston, Dominic Kwiatkowski, Cordelia Langford, John Sillitoe on behalf of the Wellcome Sanger Institute COVID-19 Surveillance Team (http://www.sanger.ac.uk/covid-team)
EPI_ISL_623808, EPI_ISL_623811, EPI_ISL_623812	Lighthouse Lab in Cambridge	Wellcome Sanger Institute for the COVID-19 Genomics UK (COG-UK) consortium	Rob Howes, The Lighthouse Lab in Cambridge and Alex Alderton, Roberto Amato, Sonia Goncalves, Ewan Harrison, David K. Jackson, Ian Johnston, Dominic Kwiatkowski, Cordelia Langford, John Sillitoe on behalf of the Wellcome Sanger Institute COVID-19 Surveillance Team (http://www.sanger.ac.uk/covid-team)
EPI_ISL_623813, EPI_ISL_623814	Lighthouse Lab in Milton Keynes	Wellcome Sanger Institute for the COVID-19 Genomics UK (COG-UK) consortium	The Lighthouse Lab in Milton Keynes and Alex Alderton, Roberto Amato, Sonia Goncalves, Ewan Harrison, David K. Jackson, Ian Johnston, Dominic Kwiatkowski, Cordelia Langford, John Sillitoe on behalf of the Wellcome Sanger Institute COVID-19 Surveillance Team (http://www.sanger.ac.uk/covid-team)
EPI_ISL_623815, EPI_ISL_623816	Lighthouse Lab in Cambridge	Wellcome Sanger Institute for the COVID-19 Genomics UK (COG-UK) consortium	Rob Howes, The Lighthouse Lab in Cambridge and Alex Alderton, Roberto Amato, Sonia Goncalves, Ewan Harrison, David K. Jackson, Ian Johnston, Dominic Kwiatkowski, Cordelia Langford, John Sillitoe on behalf of the Wellcome Sanger Institute COVID-19 Surveillance Team (http://www.sanger.ac.uk/covid-team)
EPI_ISL_623817, EPI_ISL_623818, EPI_ISL_623819, EPI_ISL_623820, EPI_ISL_623821	Lighthouse Lab in Milton Keynes	Wellcome Sanger Institute for the COVID-19 Genomics UK (COG-UK) consortium	The Lighthouse Lab in Milton Keynes and Alex Alderton, Roberto Amato, Sonia Goncalves, Ewan Harrison, David K. Jackson, Ian Johnston, Dominic Kwiatkowski, Cordelia Langford, John Sillitoe on behalf of the Wellcome Sanger Institute COVID-19 Surveillance Team (http://www.sanger.ac.uk/covid-team)
EPI_ISL_623823	Lighthouse Lab in Cambridge	Wellcome Sanger Institute for the COVID-19 Genomics UK (COG-UK) consortium	Rob Howes, The Lighthouse Lab in Cambridge and Alex Alderton, Roberto Amato, Sonia Goncalves, Ewan Harrison, David K. Jackson, Ian Johnston, Dominic Kwiatkowski, Cordelia Langford, John Sillitoe on behalf of the Wellcome Sanger Institute COVID-19 Surveillance Team (http://www.sanger.ac.uk/covid-team)
EPI_ISL_623824, EPI_ISL_623825, EPI_ISL_623826	Lighthouse Lab in Milton Keynes	Wellcome Sanger Institute for the COVID-19 Genomics UK (COG-UK) consortium	The Lighthouse Lab in Milton Keynes and Alex Alderton, Roberto Amato, Sonia Goncalves, Ewan Harrison, David K. Jackson, Ian Johnston, Dominic Kwiatkowski, Cordelia Langford, John Sillitoe on behalf of the Wellcome Sanger Institute COVID-19 Surveillance Team (http://www.sanger.ac.uk/covid-team)
EPI_ISL_623829, EPI_ISL_623831, EPI_ISL_623832, EPI_ISL_623833, EPI_ISL_623834	Lighthouse Lab in Cambridge	Wellcome Sanger Institute for the COVID-19 Genomics UK (COG-UK) consortium	Rob Howes, The Lighthouse Lab in Cambridge and Alex Alderton, Roberto Amato, Sonia Goncalves, Ewan Harrison, David K. Jackson, Ian Johnston, Dominic Kwiatkowski, Cordelia Langford, John Sillitoe on behalf of the Wellcome Sanger Institute COVID-19 Surveillance Team (http://www.sanger.ac.uk/covid-team)
EPI_ISL_623835, EPI_ISL_623836, EPI_ISL_623837	Lighthouse Lab in Milton Keynes	Wellcome Sanger Institute for the COVID-19 Genomics UK (COG-UK) consortium	The Lighthouse Lab in Milton Keynes and Alex Alderton, Roberto Amato, Sonia Goncalves, Ewan Harrison, David K. Jackson, Ian Johnston, Dominic Kwiatkowski, Cordelia Langford, John Sillitoe on behalf of the Wellcome Sanger Institute COVID-19 Surveillance Team

			(http://www.sanger.ac.uk/covid-team)
EPI_ISL_623838, EPI_ISL_623841	Lighthouse Lab in Cambridge	Wellcome Sanger Institute for the COVID-19 Genomics UK (COG-UK) consortium	Rob Howes, The Lighthouse Lab in Cambridge and Alex Alderton, Roberto Amato, Sonia Goncalves, Ewan Harrison, David K. Jackson, Ian Johnston, Dominic Kwiatkowski, Cordelia Langford, John Sillitoe on behalf of the Wellcome Sanger Institute COVID-19 Surveillance Team (http://www.sanger.ac.uk/covid-team)
EPI_ISL_623842	Lighthouse Lab in Milton Keynes	Wellcome Sanger Institute for the COVID-19 Genomics UK (COG-UK) consortium	The Lighthouse Lab in Milton Keynes and Alex Alderton, Roberto Amato, Sonia Goncalves, Ewan Harrison, David K. Jackson, Ian Johnston, Dominic Kwiatkowski, Cordelia Langford, John Sillitoe on behalf of the Wellcome Sanger Institute COVID-19 Surveillance Team (http://www.sanger.ac.uk/covid-team)
EPI_ISL_623844	Lighthouse Lab in Cambridge	Wellcome Sanger Institute for the COVID-19 Genomics UK (COG-UK) consortium	Rob Howes, The Lighthouse Lab in Cambridge and Alex Alderton, Roberto Amato, Sonia Goncalves, Ewan Harrison, David K. Jackson, Ian Johnston, Dominic Kwiatkowski, Cordelia Langford, John Sillitoe on behalf of the Wellcome Sanger Institute COVID-19 Surveillance Team (http://www.sanger.ac.uk/covid-team)
EPI_ISL_623845	Lighthouse Lab in Milton Keynes	Wellcome Sanger Institute for the COVID-19 Genomics UK (COG-UK) consortium	The Lighthouse Lab in Milton Keynes and Alex Alderton, Roberto Amato, Sonia Goncalves, Ewan Harrison, David K. Jackson, Ian Johnston, Dominic Kwiatkowski, Cordelia Langford, John Sillitoe on behalf of the Wellcome Sanger Institute COVID-19 Surveillance Team (http://www.sanger.ac.uk/covid-team)
EPI_ISL_623846, EPI_ISL_623847, EPI_ISL_623849	Lighthouse Lab in Cambridge	Wellcome Sanger Institute for the COVID-19 Genomics UK (COG-UK) consortium	Rob Howes, The Lighthouse Lab in Cambridge and Alex Alderton, Roberto Amato, Sonia Goncalves, Ewan Harrison, David K. Jackson, Ian Johnston, Dominic Kwiatkowski, Cordelia Langford, John Sillitoe on behalf of the Wellcome Sanger Institute COVID-19 Surveillance Team (http://www.sanger.ac.uk/covid-team)
EPI_ISL_623851, EPI_ISL_623852, EPI_ISL_623853	Lighthouse Lab in Milton Keynes	Wellcome Sanger Institute for the COVID-19 Genomics UK (COG-UK) consortium	The Lighthouse Lab in Milton Keynes and Alex Alderton, Roberto Amato, Sonia Goncalves, Ewan Harrison, David K. Jackson, Ian Johnston, Dominic Kwiatkowski, Cordelia Langford, John Sillitoe on behalf of the Wellcome Sanger Institute COVID-19 Surveillance Team (http://www.sanger.ac.uk/covid-team)
EPI_ISL_623854, EPI_ISL_623856, EPI_ISL_623857	Lighthouse Lab in Cambridge	Wellcome Sanger Institute for the COVID-19 Genomics UK (COG-UK) consortium	Rob Howes, The Lighthouse Lab in Cambridge and Alex Alderton, Roberto Amato, Sonia Goncalves, Ewan Harrison, David K. Jackson, Ian Johnston, Dominic Kwiatkowski, Cordelia Langford, John Sillitoe on behalf of the Wellcome Sanger Institute COVID-19 Surveillance Team (http://www.sanger.ac.uk/covid-team)
EPI_ISL_623858, EPI_ISL_623859, EPI_ISL_623860, EPI_ISL_623862	Lighthouse Lab in Alderley Park	Wellcome Sanger Institute for the COVID-19 Genomics UK (COG-UK) consortium	Jacquelyn Wynn, Mairead Hyland, The Lighthouse Lab in Alderley Park and Alex Alderton, Roberto Amato, Sonia Goncalves, Ewan Harrison, David K. Jackson, Ian Johnston, Dominic Kwiatkowski, Cordelia Langford, John Sillitoe on behalf of the Wellcome Sanger Institute COVID-19 Surveillance Team (http://www.sanger.ac.uk/covid-team)
EPI_ISL_623863, EPI_ISL_623865	Lighthouse Lab in Glasgow	Wellcome Sanger Institute for the COVID-19 Genomics UK (COG-UK) consortium	Harper VanSteenhouse, Yumi Kasai, David Gray, Carol Clugston, Anna Dominiczak and Alex Alderton, Roberto Amato, Sonia Goncalves, Ewan Harrison, David K. Jackson, Ian Johnston, Dominic Kwiatkowski, Cordelia Langford, John Sillitoe on behalf of the Wellcome Sanger Institute COVID-19 Surveillance Team (http://www.sanger.ac.uk/covid-team)
EPI_ISL_623866, EPI_ISL_623867	Lighthouse Lab in Alderley Park	Wellcome Sanger Institute for the COVID-19 Genomics UK (COG-UK) consortium	Jacquelyn Wynn, Mairead Hyland, The Lighthouse Lab in Alderley Park and Alex Alderton, Roberto Amato, Sonia Goncalves, Ewan Harrison, David K. Jackson, Ian Johnston, Dominic Kwiatkowski, Cordelia Langford, John Sillitoe on behalf of the Wellcome Sanger Institute COVID-19 Surveillance Team (http://www.sanger.ac.uk/covid-team)
EPI_ISL_623868	Lighthouse Lab in Glasgow	Wellcome Sanger Institute for the COVID-19 Genomics UK (COG-UK) consortium	Harper VanSteenhouse, Yumi Kasai, David Gray, Carol Clugston, Anna Dominiczak and Alex Alderton, Roberto Amato, Sonia Goncalves, Ewan Harrison, David K. Jackson, Ian Johnston, Dominic Kwiatkowski, Cordelia Langford, John Sillitoe on behalf of the Wellcome Sanger Institute COVID-19 Surveillance Team (http://www.sanger.ac.uk/covid-team)
EPI_ISL_623869, EPI_ISL_623870, EPI_ISL_623871	Lighthouse Lab in Alderley Park	Wellcome Sanger Institute for the COVID-19 Genomics UK (COG-UK) consortium	Jacquelyn Wynn, Mairead Hyland, The Lighthouse Lab in Alderley Park and Alex Alderton, Roberto Amato, Sonia Goncalves, Ewan Harrison, David K. Jackson, Ian Johnston, Dominic Kwiatkowski, Cordelia Langford, John Sillitoe on behalf of the Wellcome Sanger Institute COVID-19 Surveillance Team (http://www.sanger.ac.uk/covid-team)
EPI_ISL_623873	Lighthouse Lab in Glasgow	Wellcome Sanger Institute for the COVID-19 Genomics UK (COG-UK) consortium	Harper VanSteenhouse, Yumi Kasai, David Gray, Carol Clugston, Anna Dominiczak and Alex Alderton, Roberto Amato, Sonia Goncalves, Ewan Harrison, David K. Jackson, Ian Johnston, Dominic Kwiatkowski, Cordelia Langford, John Sillitoe on behalf of the Wellcome Sanger Institute COVID-19 Surveillance Team (http://www.sanger.ac.uk/covid-team)
EPI_ISL_623875	Lighthouse Lab in Alderley Park	Wellcome Sanger Institute for the COVID-19 Genomics UK (COG-UK) consortium	Jacquelyn Wynn, Mairead Hyland, The Lighthouse Lab in Alderley Park and Alex Alderton, Roberto Amato, Sonia Goncalves, Ewan Harrison, David K. Jackson, Ian Johnston, Dominic Kwiatkowski, Cordelia Langford, John Sillitoe on behalf of the Wellcome Sanger Institute COVID-19 Surveillance Team (http://www.sanger.ac.uk/covid-team)
EPI_ISL_623876	Lighthouse Lab in Glasgow	Wellcome Sanger Institute for the COVID-19 Genomics UK (COG-UK) consortium	Harper VanSteenhouse, Yumi Kasai, David Gray, Carol Clugston, Anna Dominiczak and Alex Alderton, Roberto Amato, Sonia Goncalves, Ewan Harrison, David K. Jackson, Ian Johnston, Dominic Kwiatkowski, Cordelia Langford, John Sillitoe on behalf of the Wellcome Sanger Institute COVID-19 Surveillance Team (http://www.sanger.ac.uk/covid-team)
EPI_ISL_623877, EPI_ISL_623879, EPI_ISL_623880, EPI_ISL_623881, EPI_ISL_623882	Lighthouse Lab in Alderley Park	Wellcome Sanger Institute for the COVID-19 Genomics UK (COG-UK) consortium	Jacquelyn Wynn, Mairead Hyland, The Lighthouse Lab in Alderley Park and Alex Alderton, Roberto Amato, Sonia Goncalves, Ewan Harrison, David K. Jackson, Ian Johnston, Dominic Kwiatkowski, Cordelia Langford, John Sillitoe on behalf of the Wellcome Sanger Institute COVID-19 Surveillance Team (http://www.sanger.ac.uk/covid-team)
EPI_ISL_623883, EPI_ISL_623884	Lighthouse Lab in Glasgow	Wellcome Sanger Institute for the COVID-19 Genomics UK (COG-UK) consortium	Harper VanSteenhouse, Yumi Kasai, David Gray, Carol Clugston, Anna Dominiczak and Alex Alderton, Roberto Amato, Sonia Goncalves, Ewan Harrison, David K. Jackson, Ian Johnston, Dominic Kwiatkowski, Cordelia Langford, John Sillitoe on behalf of the Wellcome Sanger Institute COVID-19 Surveillance Team (http://www.sanger.ac.uk/covid-team)
EPI_ISL_623885	Lighthouse Lab in Alderley Park	Wellcome Sanger Institute for the COVID-19 Genomics UK (COG-UK) consortium	Jacquelyn Wynn, Mairead Hyland, The Lighthouse Lab in Alderley Park and Alex Alderton, Roberto Amato, Sonia Goncalves, Ewan Harrison, David K. Jackson, Ian Johnston, Dominic Kwiatkowski, Cordelia Langford, John Sillitoe on behalf of the Wellcome Sanger Institute COVID-19 Surveillance Team (http://www.sanger.ac.uk/covid-team)
EPI_ISL_623886	Lighthouse Lab in Glasgow	Wellcome Sanger Institute for the COVID-19 Genomics UK (COG-UK) consortium	Harper VanSteenhouse, Yumi Kasai, David Gray, Carol Clugston, Anna Dominiczak and Alex Alderton, Roberto Amato, Sonia Goncalves, Ewan Harrison, David K. Jackson, Ian Johnston, Dominic Kwiatkowski, Cordelia Langford, John Sillitoe on behalf of the Wellcome Sanger Institute COVID-19 Surveillance Team (http://www.sanger.ac.uk/covid-team)
EPI_ISL_623887, EPI_ISL_623888, EPI_ISL_623889, EPI_ISL_623890, EPI_ISL_623891, EPI_ISL_623892	Lighthouse Lab in Alderley Park	Wellcome Sanger Institute for the COVID-19 Genomics UK (COG-UK) consortium	Jacquelyn Wynn, Mairead Hyland, The Lighthouse Lab in Alderley Park and Alex Alderton, Roberto Amato, Sonia Goncalves, Ewan Harrison, David K. Jackson, Ian Johnston, Dominic Kwiatkowski, Cordelia Langford, John Sillitoe on behalf of the Wellcome Sanger Institute COVID-19 Surveillance Team (http://www.sanger.ac.uk/covid-team)
EPI_ISL_623893	Lighthouse Lab in Glasgow	Wellcome Sanger Institute for the COVID-19 Genomics UK (COG-UK) consortium	Harper VanSteenhouse, Yumi Kasai, David Gray, Carol Clugston, Anna Dominiczak and Alex Alderton, Roberto Amato, Sonia Goncalves, Ewan Harrison, David K. Jackson, Ian Johnston, Dominic Kwiatkowski, Cordelia Langford, John Sillitoe on behalf of the Wellcome Sanger Institute COVID-19 Surveillance Team (http://www.sanger.ac.uk/covid-team)
EPI_ISL_623894	Lighthouse Lab in Alderley Park	Wellcome Sanger Institute for the COVID-19 Genomics UK (COG-UK) consortium	Jacquelyn Wynn, Mairead Hyland, The Lighthouse Lab in Alderley Park and Alex Alderton, Roberto Amato, Sonia Goncalves, Ewan Harrison, David K. Jackson, Ian Johnston, Dominic Kwiatkowski, Cordelia Langford, John Sillitoe on behalf of the Wellcome Sanger Institute COVID-19 Surveillance Team (http://www.sanger.ac.uk/covid-team)
EPI_ISL_623895	Lighthouse Lab in Glasgow	Wellcome Sanger Institute for the COVID-19 Genomics UK (COG-UK) consortium	Harper VanSteenhouse, Yumi Kasai, David Gray, Carol Clugston, Anna Dominiczak and Alex Alderton, Roberto Amato, Sonia Goncalves, Ewan Harrison, David K. Jackson, Ian Johnston, Dominic Kwiatkowski, Cordelia Langford, John Sillitoe on behalf of the Wellcome Sanger Institute COVID-19 Surveillance Team (http://www.sanger.ac.uk/covid-team)
EPI_ISL_623896, EPI_ISL_623897, EPI_ISL_623898, EPI_ISL_623899, EPI_ISL_623900, EPI_ISL_623902	Lighthouse Lab in Alderley Park	Wellcome Sanger Institute for the COVID-19 Genomics UK (COG-UK) consortium	Jacquelyn Wynn, Mairead Hyland, The Lighthouse Lab in Alderley Park and Alex Alderton, Roberto Amato, Sonia Goncalves, Ewan Harrison, David K. Jackson, Ian Johnston, Dominic Kwiatkowski, Cordelia Langford, John Sillitoe on behalf of the Wellcome Sanger Institute COVID-19 Surveillance Team (http://www.sanger.ac.uk/covid-team)
EPI_ISL_623903	Lighthouse Lab in Glasgow	Wellcome Sanger Institute for the COVID-19 Genomics UK (COG-UK) consortium	Harper VanSteenhouse, Yumi Kasai, David Gray, Carol Clugston, Anna Dominiczak and Alex Alderton, Roberto Amato, Sonia Goncalves, Ewan Harrison, David K. Jackson, Ian Johnston, Dominic Kwiatkowski, Cordelia Langford, John Sillitoe on behalf of the Wellcome Sanger Institute COVID-19 Surveillance

			Team (http://www.sanger.ac.uk/covid-team)
EPI_ISL_623905, EPI_ISL_623906	Lighthouse Lab in Alderley Park	Wellcome Sanger Institute for the COVID-19 Genomics UK (COG-UK) consortium	Jacquelyn Wynn, Mairead Hyland, The Lighthouse Lab in Alderley Park and Alex Alderton, Roberto Amato, Sonia Goncalves, Ewan Harrison, David K. Jackson, Ian Johnston, Dominic Kwiatkowski, Cordelia Langford, John Sillitoe on behalf of the Wellcome Sanger Institute COVID-19 Surveillance Team (http://www.sanger.ac.uk/covid-team)
EPI_ISL_623907, EPI_ISL_623908	Lighthouse Lab in Glasgow	Wellcome Sanger Institute for the COVID-19 Genomics UK (COG-UK) consortium	Harper VanSteenhouse, Yumi Kasai, David Gray, Carol Clugston, Anna Dominiczak and Alex Alderton, Roberto Amato, Sonia Goncalves, Ewan Harrison, David K. Jackson, Ian Johnston, Dominic Kwiatkowski, Cordelia Langford, John Sillitoe on behalf of the Wellcome Sanger Institute COVID-19 Surveillance Team (http://www.sanger.ac.uk/covid-team)
EPI_ISL_623909	Lighthouse Lab in Alderley Park	Wellcome Sanger Institute for the COVID-19 Genomics UK (COG-UK) consortium	Jacquelyn Wynn, Mairead Hyland, The Lighthouse Lab in Alderley Park and Alex Alderton, Roberto Amato, Sonia Goncalves, Ewan Harrison, David K. Jackson, Ian Johnston, Dominic Kwiatkowski, Cordelia Langford, John Sillitoe on behalf of the Wellcome Sanger Institute COVID-19 Surveillance Team (http://www.sanger.ac.uk/covid-team)
EPI_ISL_623911	Lighthouse Lab in Glasgow	Wellcome Sanger Institute for the COVID-19 Genomics UK (COG-UK) consortium	Harper VanSteenhouse, Yumi Kasai, David Gray, Carol Clugston, Anna Dominiczak and Alex Alderton, Roberto Amato, Sonia Goncalves, Ewan Harrison, David K. Jackson, Ian Johnston, Dominic Kwiatkowski, Cordelia Langford, John Sillitoe on behalf of the Wellcome Sanger Institute COVID-19 Surveillance Team (http://www.sanger.ac.uk/covid-team)
EPI_ISL_623912, EPI_ISL_623913, EPI_ISL_623914, EPI_ISL_623915	Lighthouse Lab in Alderley Park	Wellcome Sanger Institute for the COVID-19 Genomics UK (COG-UK) consortium	Jacquelyn Wynn, Mairead Hyland, The Lighthouse Lab in Alderley Park and Alex Alderton, Roberto Amato, Sonia Goncalves, Ewan Harrison, David K. Jackson, Ian Johnston, Dominic Kwiatkowski, Cordelia Langford, John Sillitoe on behalf of the Wellcome Sanger Institute COVID-19 Surveillance Team (http://www.sanger.ac.uk/covid-team)
EPI_ISL_623916, EPI_ISL_623917	Lighthouse Lab in Glasgow	Wellcome Sanger Institute for the COVID-19 Genomics UK (COG-UK) consortium	Harper VanSteenhouse, Yumi Kasai, David Gray, Carol Clugston, Anna Dominiczak and Alex Alderton, Roberto Amato, Sonia Goncalves, Ewan Harrison, David K. Jackson, Ian Johnston, Dominic Kwiatkowski, Cordelia Langford, John Sillitoe on behalf of the Wellcome Sanger Institute COVID-19 Surveillance Team (http://www.sanger.ac.uk/covid-team)
EPI_ISL_623918, EPI_ISL_623919, EPI_ISL_623920	Lighthouse Lab in Alderley Park	Wellcome Sanger Institute for the COVID-19 Genomics UK (COG-UK) consortium	Jacquelyn Wynn, Mairead Hyland, The Lighthouse Lab in Alderley Park and Alex Alderton, Roberto Amato, Sonia Goncalves, Ewan Harrison, David K. Jackson, Ian Johnston, Dominic Kwiatkowski, Cordelia Langford, John Sillitoe on behalf of the Wellcome Sanger Institute COVID-19 Surveillance Team (http://www.sanger.ac.uk/covid-team)
EPI_ISL_623921	Lighthouse Lab in Glasgow	Wellcome Sanger Institute for the COVID-19 Genomics UK (COG-UK) consortium	Harper VanSteenhouse, Yumi Kasai, David Gray, Carol Clugston, Anna Dominiczak and Alex Alderton, Roberto Amato, Sonia Goncalves, Ewan Harrison, David K. Jackson, Ian Johnston, Dominic Kwiatkowski, Cordelia Langford, John Sillitoe on behalf of the Wellcome Sanger Institute COVID-19 Surveillance Team (http://www.sanger.ac.uk/covid-team)
EPI_ISL_623922	Lighthouse Lab in Alderley Park	Wellcome Sanger Institute for the COVID-19 Genomics UK (COG-UK) consortium	Jacquelyn Wynn, Mairead Hyland, The Lighthouse Lab in Alderley Park and Alex Alderton, Roberto Amato, Sonia Goncalves, Ewan Harrison, David K. Jackson, Ian Johnston, Dominic Kwiatkowski, Cordelia Langford, John Sillitoe on behalf of the Wellcome Sanger Institute COVID-19 Surveillance Team (http://www.sanger.ac.uk/covid-team)
EPI_ISL_623924	Lighthouse Lab in Glasgow	Wellcome Sanger Institute for the COVID-19 Genomics UK (COG-UK) consortium	Harper VanSteenhouse, Yumi Kasai, David Gray, Carol Clugston, Anna Dominiczak and Alex Alderton, Roberto Amato, Sonia Goncalves, Ewan Harrison, David K. Jackson, Ian Johnston, Dominic Kwiatkowski, Cordelia Langford, John Sillitoe on behalf of the Wellcome Sanger Institute COVID-19 Surveillance Team (http://www.sanger.ac.uk/covid-team)
EPI_ISL_623925, EPI_ISL_623927, EPI_ISL_623928, EPI_ISL_623929	Lighthouse Lab in Alderley Park	Wellcome Sanger Institute for the COVID-19 Genomics UK (COG-UK) consortium	Jacquelyn Wynn, Mairead Hyland, The Lighthouse Lab in Alderley Park and Alex Alderton, Roberto Amato, Sonia Goncalves, Ewan Harrison, David K. Jackson, Ian Johnston, Dominic Kwiatkowski, Cordelia Langford, John Sillitoe on behalf of the Wellcome Sanger Institute COVID-19 Surveillance Team (http://www.sanger.ac.uk/covid-team)
EPI_ISL_623930	Lighthouse Lab in Glasgow	Wellcome Sanger Institute for the COVID-19 Genomics UK (COG-UK) consortium	Harper VanSteenhouse, Yumi Kasai, David Gray, Carol Clugston, Anna Dominiczak and Alex Alderton, Roberto Amato, Sonia Goncalves, Ewan Harrison, David K. Jackson, Ian Johnston, Dominic Kwiatkowski, Cordelia Langford, John Sillitoe on behalf of the Wellcome Sanger Institute COVID-19 Surveillance Team (http://www.sanger.ac.uk/covid-team)
EPI_ISL_623931, EPI_ISL_623932	Lighthouse Lab in Alderley Park	Wellcome Sanger Institute for the COVID-19 Genomics UK (COG-UK) consortium	Jacquelyn Wynn, Mairead Hyland, The Lighthouse Lab in Alderley Park and Alex Alderton, Roberto Amato, Sonia Goncalves, Ewan Harrison, David K. Jackson, Ian Johnston, Dominic Kwiatkowski, Cordelia Langford, John Sillitoe on behalf of the Wellcome Sanger Institute COVID-19 Surveillance Team (http://www.sanger.ac.uk/covid-team)
EPI_ISL_623933, EPI_ISL_623934	Lighthouse Lab in Glasgow	Wellcome Sanger Institute for the COVID-19 Genomics UK (COG-UK) consortium	Harper VanSteenhouse, Yumi Kasai, David Gray, Carol Clugston, Anna Dominiczak and Alex Alderton, Roberto Amato, Sonia Goncalves, Ewan Harrison, David K. Jackson, Ian Johnston, Dominic Kwiatkowski, Cordelia Langford, John Sillitoe on behalf of the Wellcome Sanger Institute COVID-19 Surveillance Team (http://www.sanger.ac.uk/covid-team)
EPI_ISL_623935, EPI_ISL_623936, EPI_ISL_623937, EPI_ISL_623938, EPI_ISL_623940, EPI_ISL_623941	Lighthouse Lab in Alderley Park	Wellcome Sanger Institute for the COVID-19 Genomics UK (COG-UK) consortium	Jacquelyn Wynn, Mairead Hyland, The Lighthouse Lab in Alderley Park and Alex Alderton, Roberto Amato, Sonia Goncalves, Ewan Harrison, David K. Jackson, Ian Johnston, Dominic Kwiatkowski, Cordelia Langford, John Sillitoe on behalf of the Wellcome Sanger Institute COVID-19 Surveillance Team (http://www.sanger.ac.uk/covid-team)
EPI_ISL_623942	Lighthouse Lab in Glasgow	Wellcome Sanger Institute for the COVID-19 Genomics UK (COG-UK) consortium	Harper VanSteenhouse, Yumi Kasai, David Gray, Carol Clugston, Anna Dominiczak and Alex Alderton, Roberto Amato, Sonia Goncalves, Ewan Harrison, David K. Jackson, Ian Johnston, Dominic Kwiatkowski, Cordelia Langford, John Sillitoe on behalf of the Wellcome Sanger Institute COVID-19 Surveillance Team (http://www.sanger.ac.uk/covid-team)
EPI_ISL_623943	Lighthouse Lab in Alderley Park	Wellcome Sanger Institute for the COVID-19 Genomics UK (COG-UK) consortium	Jacquelyn Wynn, Mairead Hyland, The Lighthouse Lab in Alderley Park and Alex Alderton, Roberto Amato, Sonia Goncalves, Ewan Harrison, David K. Jackson, Ian Johnston, Dominic Kwiatkowski, Cordelia Langford, John Sillitoe on behalf of the Wellcome Sanger Institute COVID-19 Surveillance Team (http://www.sanger.ac.uk/covid-team)
EPI_ISL_623944	Lighthouse Lab in Glasgow	Wellcome Sanger Institute for the COVID-19 Genomics UK (COG-UK) consortium	Harper VanSteenhouse, Yumi Kasai, David Gray, Carol Clugston, Anna Dominiczak and Alex Alderton, Roberto Amato, Sonia Goncalves, Ewan Harrison, David K. Jackson, Ian Johnston, Dominic Kwiatkowski, Cordelia Langford, John Sillitoe on behalf of the Wellcome Sanger Institute COVID-19 Surveillance Team (http://www.sanger.ac.uk/covid-team)
EPI_ISL_623945, EPI_ISL_623946, EPI_ISL_623947, EPI_ISL_623948	Lighthouse Lab in Alderley Park	Wellcome Sanger Institute for the COVID-19 Genomics UK (COG-UK) consortium	Jacquelyn Wynn, Mairead Hyland, The Lighthouse Lab in Alderley Park and Alex Alderton, Roberto Amato, Sonia Goncalves, Ewan Harrison, David K. Jackson, Ian Johnston, Dominic Kwiatkowski, Cordelia Langford, John Sillitoe on behalf of the Wellcome Sanger Institute COVID-19 Surveillance Team (http://www.sanger.ac.uk/covid-team)
EPI_ISL_623949	Lighthouse Lab in Glasgow	Wellcome Sanger Institute for the COVID-19 Genomics UK (COG-UK) consortium	Harper VanSteenhouse, Yumi Kasai, David Gray, Carol Clugston, Anna Dominiczak and Alex Alderton, Roberto Amato, Sonia Goncalves, Ewan Harrison, David K. Jackson, Ian Johnston, Dominic Kwiatkowski, Cordelia Langford, John Sillitoe on behalf of the Wellcome Sanger Institute COVID-19 Surveillance Team (http://www.sanger.ac.uk/covid-team)
EPI_ISL_623950, EPI_ISL_623951, EPI_ISL_623952, EPI_ISL_623953	Lighthouse Lab in Alderley Park	Wellcome Sanger Institute for the COVID-19 Genomics UK (COG-UK) consortium	Jacquelyn Wynn, Mairead Hyland, The Lighthouse Lab in Alderley Park and Alex Alderton, Roberto Amato, Sonia Goncalves, Ewan Harrison, David K. Jackson, Ian Johnston, Dominic Kwiatkowski, Cordelia Langford, John Sillitoe on behalf of the Wellcome Sanger Institute COVID-19 Surveillance Team (http://www.sanger.ac.uk/covid-team)
EPI_ISL_623954	Lighthouse Lab in Glasgow	Wellcome Sanger Institute for the COVID-19 Genomics UK (COG-UK) consortium	Harper VanSteenhouse, Yumi Kasai, David Gray, Carol Clugston, Anna Dominiczak and Alex Alderton, Roberto Amato, Sonia Goncalves, Ewan Harrison, David K. Jackson, Ian Johnston, Dominic Kwiatkowski, Cordelia Langford, John Sillitoe on behalf of the Wellcome Sanger Institute COVID-19 Surveillance Team (http://www.sanger.ac.uk/covid-team)
EPI_ISL_623955	Lighthouse Lab in Alderley Park	Wellcome Sanger Institute for the COVID-19 Genomics UK (COG-UK) consortium	Jacquelyn Wynn, Mairead Hyland, The Lighthouse Lab in Alderley Park and Alex Alderton, Roberto Amato, Sonia Goncalves, Ewan Harrison, David K. Jackson, Ian Johnston, Dominic Kwiatkowski, Cordelia Langford, John Sillitoe on behalf of the Wellcome Sanger Institute COVID-19 Surveillance Team (http://www.sanger.ac.uk/covid-team)
EPI_ISL_623956	Lighthouse Lab in Glasgow	Wellcome Sanger Institute for the COVID-19 Genomics UK (COG-UK) consortium	Harper VanSteenhouse, Yumi Kasai, David Gray, Carol Clugston, Anna Dominiczak and Alex Alderton, Roberto Amato, Sonia Goncalves, Ewan Harrison, David K. Jackson, Ian Johnston, Dominic Kwiatkowski, Cordelia Langford, John Sillitoe on behalf of the Wellcome Sanger Institute COVID-19 Surveillance Team (http://www.sanger.ac.uk/covid-team)
EPI_ISL_623957, EPI_ISL_623958	Lighthouse Lab in Alderley Park	Wellcome Sanger Institute for the COVID-19 Genomics UK (COG-UK) consortium	Jacquelyn Wynn, Mairead Hyland, The Lighthouse Lab in Alderley Park and Alex Alderton, Roberto Amato, Sonia Goncalves, Ewan Harrison, David K. Jackson, Ian Johnston, Dominic Kwiatkowski, Cordelia Langford, John Sillitoe on behalf of the Wellcome Sanger Institute COVID-19 Surveillance Team

			(http://www.sanger.ac.uk/covid-team)
EPI_ISL_623959	Lighthouse Lab in Glasgow	Wellcome Sanger Institute for the COVID-19 Genomics UK (COG-UK) consortium	Harper VanSteenhouse, Yumi Kasai, David Gray, Carol Clugston, Anna Dominiczak and Alex Alderton, Roberto Amato, Sonia Goncalves, Ewan Harrison, David K. Jackson, Ian Johnston, Dominic Kwiatkowski, Cordelia Langford, John Sillitoe on behalf of the Wellcome Sanger Institute COVID-19 Surveillance Team (http://www.sanger.ac.uk/covid-team)
EPI_ISL_623961, EPI_ISL_623962	Lighthouse Lab in Alderley Park	Wellcome Sanger Institute for the COVID-19 Genomics UK (COG-UK) consortium	Jacquelyn Wynn, Mairead Hyland, The Lighthouse Lab in Alderley Park and Alex Alderton, Roberto Amato, Sonia Goncalves, Ewan Harrison, David K. Jackson, Ian Johnston, Dominic Kwiatkowski, Cordelia Langford, John Sillitoe on behalf of the Wellcome Sanger Institute COVID-19 Surveillance Team (http://www.sanger.ac.uk/covid-team)
EPI_ISL_623963	Lighthouse Lab in Glasgow	Wellcome Sanger Institute for the COVID-19 Genomics UK (COG-UK) consortium	Harper VanSteenhouse, Yumi Kasai, David Gray, Carol Clugston, Anna Dominiczak and Alex Alderton, Roberto Amato, Sonia Goncalves, Ewan Harrison, David K. Jackson, Ian Johnston, Dominic Kwiatkowski, Cordelia Langford, John Sillitoe on behalf of the Wellcome Sanger Institute COVID-19 Surveillance Team (http://www.sanger.ac.uk/covid-team)
EPI_ISL_623964, EPI_ISL_623965, EPI_ISL_623967, EPI_ISL_623969, EPI_ISL_623970, EPI_ISL_623971, EPI_ISL_623973	Lighthouse Lab in Alderley Park	Wellcome Sanger Institute for the COVID-19 Genomics UK (COG-UK) consortium	Jacquelyn Wynn, Mairead Hyland, The Lighthouse Lab in Alderley Park and Alex Alderton, Roberto Amato, Sonia Goncalves, Ewan Harrison, David K. Jackson, Ian Johnston, Dominic Kwiatkowski, Cordelia Langford, John Sillitoe on behalf of the Wellcome Sanger Institute COVID-19 Surveillance Team (http://www.sanger.ac.uk/covid-team)
EPI_ISL_623975, EPI_ISL_623976, EPI_ISL_623977	Lighthouse Lab in Glasgow	Wellcome Sanger Institute for the COVID-19 Genomics UK (COG-UK) consortium	Harper VanSteenhouse, Yumi Kasai, David Gray, Carol Clugston, Anna Dominiczak and Alex Alderton, Roberto Amato, Sonia Goncalves, Ewan Harrison, David K. Jackson, Ian Johnston, Dominic Kwiatkowski, Cordelia Langford, John Sillitoe on behalf of the Wellcome Sanger Institute COVID-19 Surveillance Team (http://www.sanger.ac.uk/covid-team)
EPI_ISL_623978, EPI_ISL_623981, EPI_ISL_623982, EPI_ISL_623984, EPI_ISL_623985	Lighthouse Lab in Alderley Park	Wellcome Sanger Institute for the COVID-19 Genomics UK (COG-UK) consortium	Jacquelyn Wynn, Mairead Hyland, The Lighthouse Lab in Alderley Park and Alex Alderton, Roberto Amato, Sonia Goncalves, Ewan Harrison, David K. Jackson, Ian Johnston, Dominic Kwiatkowski, Cordelia Langford, John Sillitoe on behalf of the Wellcome Sanger Institute COVID-19 Surveillance Team (http://www.sanger.ac.uk/covid-team)
EPI_ISL_623986	Lighthouse Lab in Glasgow	Wellcome Sanger Institute for the COVID-19 Genomics UK (COG-UK) consortium	Harper VanSteenhouse, Yumi Kasai, David Gray, Carol Clugston, Anna Dominiczak and Alex Alderton, Roberto Amato, Sonia Goncalves, Ewan Harrison, David K. Jackson, Ian Johnston, Dominic Kwiatkowski, Cordelia Langford, John Sillitoe on behalf of the Wellcome Sanger Institute COVID-19 Surveillance Team (http://www.sanger.ac.uk/covid-team)
EPI_ISL_623987, EPI_ISL_623988, EPI_ISL_623989	Lighthouse Lab in Alderley Park	Wellcome Sanger Institute for the COVID-19 Genomics UK (COG-UK) consortium	Jacquelyn Wynn, Mairead Hyland, The Lighthouse Lab in Alderley Park and Alex Alderton, Roberto Amato, Sonia Goncalves, Ewan Harrison, David K. Jackson, Ian Johnston, Dominic Kwiatkowski, Cordelia Langford, John Sillitoe on behalf of the Wellcome Sanger Institute COVID-19 Surveillance Team (http://www.sanger.ac.uk/covid-team)
EPI_ISL_623990, EPI_ISL_623991	Lighthouse Lab in Glasgow	Wellcome Sanger Institute for the COVID-19 Genomics UK (COG-UK) consortium	Harper VanSteenhouse, Yumi Kasai, David Gray, Carol Clugston, Anna Dominiczak and Alex Alderton, Roberto Amato, Sonia Goncalves, Ewan Harrison, David K. Jackson, Ian Johnston, Dominic Kwiatkowski, Cordelia Langford, John Sillitoe on behalf of the Wellcome Sanger Institute COVID-19 Surveillance Team (http://www.sanger.ac.uk/covid-team)
EPI_ISL_623992, EPI_ISL_623993	Lighthouse Lab in Alderley Park	Wellcome Sanger Institute for the COVID-19 Genomics UK (COG-UK) consortium	Jacquelyn Wynn, Mairead Hyland, The Lighthouse Lab in Alderley Park and Alex Alderton, Roberto Amato, Sonia Goncalves, Ewan Harrison, David K. Jackson, Ian Johnston, Dominic Kwiatkowski, Cordelia Langford, John Sillitoe on behalf of the Wellcome Sanger Institute COVID-19 Surveillance Team (http://www.sanger.ac.uk/covid-team)
EPI_ISL_623994, EPI_ISL_623995	Lighthouse Lab in Glasgow	Wellcome Sanger Institute for the COVID-19 Genomics UK (COG-UK) consortium	Harper VanSteenhouse, Yumi Kasai, David Gray, Carol Clugston, Anna Dominiczak and Alex Alderton, Roberto Amato, Sonia Goncalves, Ewan Harrison, David K. Jackson, Ian Johnston, Dominic Kwiatkowski, Cordelia Langford, John Sillitoe on behalf of the Wellcome Sanger Institute COVID-19 Surveillance Team (http://www.sanger.ac.uk/covid-team)
EPI_ISL_623996, EPI_ISL_623997, EPI_ISL_623998, EPI_ISL_623999, EPI_ISL_624000, EPI_ISL_624003, EPI_ISL_624004, EPI_ISL_624005, EPI_ISL_624006, EPI_ISL_624008, EPI_ISL_624009			
see above	Lighthouse Lab in Alderley Park	Wellcome Sanger Institute for the COVID-19 Genomics UK (COG-UK) consortium	Jacquelyn Wynn, Mairead Hyland, The Lighthouse Lab in Alderley Park and Alex Alderton, Roberto Amato, Sonia Goncalves, Ewan Harrison, David K. Jackson, Ian Johnston, Dominic Kwiatkowski, Cordelia Langford, John Sillitoe on behalf of the Wellcome Sanger Institute COVID-19 Surveillance Team (http://www.sanger.ac.uk/covid-team)
EPI_ISL_624010	Lighthouse Lab in Glasgow	Wellcome Sanger Institute for the COVID-19 Genomics UK (COG-UK) consortium	Harper VanSteenhouse, Yumi Kasai, David Gray, Carol Clugston, Anna Dominiczak and Alex Alderton, Roberto Amato, Sonia Goncalves, Ewan Harrison, David K. Jackson, Ian Johnston, Dominic Kwiatkowski, Cordelia Langford, John Sillitoe on behalf of the Wellcome Sanger Institute COVID-19 Surveillance Team (http://www.sanger.ac.uk/covid-team)
EPI_ISL_624011, EPI_ISL_624013, EPI_ISL_624014, EPI_ISL_624015, EPI_ISL_624016, EPI_ISL_624017	Lighthouse Lab in Alderley Park	Wellcome Sanger Institute for the COVID-19 Genomics UK (COG-UK) consortium	Jacquelyn Wynn, Mairead Hyland, The Lighthouse Lab in Alderley Park and Alex Alderton, Roberto Amato, Sonia Goncalves, Ewan Harrison, David K. Jackson, Ian Johnston, Dominic Kwiatkowski, Cordelia Langford, John Sillitoe on behalf of the Wellcome Sanger Institute COVID-19 Surveillance Team (http://www.sanger.ac.uk/covid-team)
EPI_ISL_624018, EPI_ISL_624019	Lighthouse Lab in Glasgow	Wellcome Sanger Institute for the COVID-19 Genomics UK (COG-UK) consortium	Harper VanSteenhouse, Yumi Kasai, David Gray, Carol Clugston, Anna Dominiczak and Alex Alderton, Roberto Amato, Sonia Goncalves, Ewan Harrison, David K. Jackson, Ian Johnston, Dominic Kwiatkowski, Cordelia Langford, John Sillitoe on behalf of the Wellcome Sanger Institute COVID-19 Surveillance Team (http://www.sanger.ac.uk/covid-team)
EPI_ISL_624020, EPI_ISL_624021	Lighthouse Lab in Alderley Park	Wellcome Sanger Institute for the COVID-19 Genomics UK (COG-UK) consortium	Jacquelyn Wynn, Mairead Hyland, The Lighthouse Lab in Alderley Park and Alex Alderton, Roberto Amato, Sonia Goncalves, Ewan Harrison, David K. Jackson, Ian Johnston, Dominic Kwiatkowski, Cordelia Langford, John Sillitoe on behalf of the Wellcome Sanger Institute COVID-19 Surveillance Team (http://www.sanger.ac.uk/covid-team)
EPI_ISL_624022	Lighthouse Lab in Glasgow	Wellcome Sanger Institute for the COVID-19 Genomics UK (COG-UK) consortium	Harper VanSteenhouse, Yumi Kasai, David Gray, Carol Clugston, Anna Dominiczak and Alex Alderton, Roberto Amato, Sonia Goncalves, Ewan Harrison, David K. Jackson, Ian Johnston, Dominic Kwiatkowski, Cordelia Langford, John Sillitoe on behalf of the Wellcome Sanger Institute COVID-19 Surveillance Team (http://www.sanger.ac.uk/covid-team)
EPI_ISL_624023, EPI_ISL_624025, EPI_ISL_624026	Lighthouse Lab in Alderley Park	Wellcome Sanger Institute for the COVID-19 Genomics UK (COG-UK) consortium	Jacquelyn Wynn, Mairead Hyland, The Lighthouse Lab in Alderley Park and Alex Alderton, Roberto Amato, Sonia Goncalves, Ewan Harrison, David K. Jackson, Ian Johnston, Dominic Kwiatkowski, Cordelia Langford, John Sillitoe on behalf of the Wellcome Sanger Institute COVID-19 Surveillance Team (http://www.sanger.ac.uk/covid-team)
EPI_ISL_624027, EPI_ISL_624028	Lighthouse Lab in Glasgow	Wellcome Sanger Institute for the COVID-19 Genomics UK (COG-UK) consortium	Harper VanSteenhouse, Yumi Kasai, David Gray, Carol Clugston, Anna Dominiczak and Alex Alderton, Roberto Amato, Sonia Goncalves, Ewan Harrison, David K. Jackson, Ian Johnston, Dominic Kwiatkowski, Cordelia Langford, John Sillitoe on behalf of the Wellcome Sanger Institute COVID-19 Surveillance Team (http://www.sanger.ac.uk/covid-team)
EPI_ISL_624030, EPI_ISL_624031	Lighthouse Lab in Alderley Park	Wellcome Sanger Institute for the COVID-19 Genomics UK (COG-UK) consortium	Jacquelyn Wynn, Mairead Hyland, The Lighthouse Lab in Alderley Park and Alex Alderton, Roberto Amato, Sonia Goncalves, Ewan Harrison, David K. Jackson, Ian Johnston, Dominic Kwiatkowski, Cordelia Langford, John Sillitoe on behalf of the Wellcome Sanger Institute COVID-19 Surveillance Team (http://www.sanger.ac.uk/covid-team)
EPI_ISL_624032, EPI_ISL_624034	Lighthouse Lab in Glasgow	Wellcome Sanger Institute for the COVID-19 Genomics UK (COG-UK) consortium	Harper VanSteenhouse, Yumi Kasai, David Gray, Carol Clugston, Anna Dominiczak and Alex Alderton, Roberto Amato, Sonia Goncalves, Ewan Harrison, David K. Jackson, Ian Johnston, Dominic Kwiatkowski, Cordelia Langford, John Sillitoe on behalf of the Wellcome Sanger Institute COVID-19 Surveillance Team (http://www.sanger.ac.uk/covid-team)
EPI_ISL_624035, EPI_ISL_624036, EPI_ISL_624037, EPI_ISL_624038, EPI_ISL_624039, EPI_ISL_624040, EPI_ISL_624041	Lighthouse Lab in Alderley Park	Wellcome Sanger Institute for the COVID-19 Genomics UK (COG-UK) consortium	Jacquelyn Wynn, Mairead Hyland, The Lighthouse Lab in Alderley Park and Alex Alderton, Roberto Amato, Sonia Goncalves, Ewan Harrison, David K. Jackson, Ian Johnston, Dominic Kwiatkowski, Cordelia Langford, John Sillitoe on behalf of the Wellcome Sanger Institute COVID-19 Surveillance Team (http://www.sanger.ac.uk/covid-team)
EPI_ISL_624043	Lighthouse Lab in Glasgow	Wellcome Sanger Institute for the COVID-19 Genomics UK (COG-UK) consortium	Harper VanSteenhouse, Yumi Kasai, David Gray, Carol Clugston, Anna Dominiczak and Alex Alderton, Roberto Amato, Sonia Goncalves, Ewan Harrison, David K. Jackson, Ian Johnston, Dominic Kwiatkowski, Cordelia Langford, John Sillitoe on behalf of the Wellcome Sanger Institute COVID-19 Surveillance Team (http://www.sanger.ac.uk/covid-team)
EPI_ISL_624047, EPI_ISL_624048	Lighthouse Lab in Alderley Park	Wellcome Sanger Institute for the COVID-19 Genomics UK (COG-UK) consortium	Jacquelyn Wynn, Mairead Hyland, The Lighthouse Lab in Alderley Park and Alex Alderton, Roberto Amato, Sonia Goncalves, Ewan Harrison, David K. Jackson, Ian Johnston, Dominic Kwiatkowski, Cordelia Langford, John Sillitoe on behalf of the Wellcome Sanger Institute COVID-19 Surveillance Team (http://www.sanger.ac.uk/covid-team)

EPI_ISL_624049	Lighthouse Lab in Glasgow	Wellcome Sanger Institute for the COVID-19 Genomics UK (COG-UK) consortium	Harper VanSteenhouse, Yumi Kasai, David Gray, Carol Clugston, Anna Dominiczak and Alex Alderton, Roberto Amato, Sonia Goncalves, Ewan Harrison, David K. Jackson, Ian Johnston, Dominic Kwiatkowski, Cordelia Langford, John Sillitoe on behalf of the Wellcome Sanger Institute COVID-19 Surveillance Team (http://www.sanger.ac.uk/covid-team)
EPI_ISL_624050, EPI_ISL_624051, EPI_ISL_624052, EPI_ISL_624053, EPI_ISL_624054, EPI_ISL_624055, EPI_ISL_624056, EPI_ISL_624058, EPI_ISL_624059, EPI_ISL_624060, EPI_ISL_624062, EPI_ISL_624063, EPI_ISL_624064, EPI_ISL_624066			
see above	Lighthouse Lab in Alderley Park	Wellcome Sanger Institute for the COVID-19 Genomics UK (COG-UK) consortium	Jacquelyn Wynn, Mairead Hyland, The Lighthouse Lab in Alderley Park and Alex Alderton, Roberto Amato, Sonia Goncalves, Ewan Harrison, David K. Jackson, Ian Johnston, Dominic Kwiatkowski, Cordelia Langford, John Sillitoe on behalf of the Wellcome Sanger Institute COVID-19 Surveillance Team (http://www.sanger.ac.uk/covid-team)
EPI_ISL_624067	Lighthouse Lab in Glasgow	Wellcome Sanger Institute for the COVID-19 Genomics UK (COG-UK) consortium	Harper VanSteenhouse, Yumi Kasai, David Gray, Carol Clugston, Anna Dominiczak and Alex Alderton, Roberto Amato, Sonia Goncalves, Ewan Harrison, David K. Jackson, Ian Johnston, Dominic Kwiatkowski, Cordelia Langford, John Sillitoe on behalf of the Wellcome Sanger Institute COVID-19 Surveillance Team (http://www.sanger.ac.uk/covid-team)
EPI_ISL_624068, EPI_ISL_624069, EPI_ISL_624070, EPI_ISL_624072	Lighthouse Lab in Alderley Park	Wellcome Sanger Institute for the COVID-19 Genomics UK (COG-UK) consortium	Jacquelyn Wynn, Mairead Hyland, The Lighthouse Lab in Alderley Park and Alex Alderton, Roberto Amato, Sonia Goncalves, Ewan Harrison, David K. Jackson, Ian Johnston, Dominic Kwiatkowski, Cordelia Langford, John Sillitoe on behalf of the Wellcome Sanger Institute COVID-19 Surveillance Team (http://www.sanger.ac.uk/covid-team)
EPI_ISL_624073	Lighthouse Lab in Glasgow	Wellcome Sanger Institute for the COVID-19 Genomics UK (COG-UK) consortium	Harper VanSteenhouse, Yumi Kasai, David Gray, Carol Clugston, Anna Dominiczak and Alex Alderton, Roberto Amato, Sonia Goncalves, Ewan Harrison, David K. Jackson, Ian Johnston, Dominic Kwiatkowski, Cordelia Langford, John Sillitoe on behalf of the Wellcome Sanger Institute COVID-19 Surveillance Team (http://www.sanger.ac.uk/covid-team)
EPI_ISL_624074, EPI_ISL_624075, EPI_ISL_624076	Lighthouse Lab in Alderley Park	Wellcome Sanger Institute for the COVID-19 Genomics UK (COG-UK) consortium	Jacquelyn Wynn, Mairead Hyland, The Lighthouse Lab in Alderley Park and Alex Alderton, Roberto Amato, Sonia Goncalves, Ewan Harrison, David K. Jackson, Ian Johnston, Dominic Kwiatkowski, Cordelia Langford, John Sillitoe on behalf of the Wellcome Sanger Institute COVID-19 Surveillance Team (http://www.sanger.ac.uk/covid-team)
EPI_ISL_624077	Lighthouse Lab in Glasgow	Wellcome Sanger Institute for the COVID-19 Genomics UK (COG-UK) consortium	Harper VanSteenhouse, Yumi Kasai, David Gray, Carol Clugston, Anna Dominiczak and Alex Alderton, Roberto Amato, Sonia Goncalves, Ewan Harrison, David K. Jackson, Ian Johnston, Dominic Kwiatkowski, Cordelia Langford, John Sillitoe on behalf of the Wellcome Sanger Institute COVID-19 Surveillance Team (http://www.sanger.ac.uk/covid-team)
EPI_ISL_624080, EPI_ISL_624082, EPI_ISL_624083, EPI_ISL_624084, EPI_ISL_624085, EPI_ISL_624086, EPI_ISL_624087, EPI_ISL_624088, EPI_ISL_624089, EPI_ISL_624090, EPI_ISL_624091			
see above	Lighthouse Lab in Alderley Park	Wellcome Sanger Institute for the COVID-19 Genomics UK (COG-UK) consortium	Jacquelyn Wynn, Mairead Hyland, The Lighthouse Lab in Alderley Park and Alex Alderton, Roberto Amato, Sonia Goncalves, Ewan Harrison, David K. Jackson, Ian Johnston, Dominic Kwiatkowski, Cordelia Langford, John Sillitoe on behalf of the Wellcome Sanger Institute COVID-19 Surveillance Team (http://www.sanger.ac.uk/covid-team)
EPI_ISL_624092	Lighthouse Lab in Glasgow	Wellcome Sanger Institute for the COVID-19 Genomics UK (COG-UK) consortium	Harper VanSteenhouse, Yumi Kasai, David Gray, Carol Clugston, Anna Dominiczak and Alex Alderton, Roberto Amato, Sonia Goncalves, Ewan Harrison, David K. Jackson, Ian Johnston, Dominic Kwiatkowski, Cordelia Langford, John Sillitoe on behalf of the Wellcome Sanger Institute COVID-19 Surveillance Team (http://www.sanger.ac.uk/covid-team)
EPI_ISL_624093, EPI_ISL_624096, EPI_ISL_624097, EPI_ISL_624098, EPI_ISL_624099, EPI_ISL_624100, EPI_ISL_624102, EPI_ISL_624103, EPI_ISL_624104	Lighthouse Lab in Alderley Park	Wellcome Sanger Institute for the COVID-19 Genomics UK (COG-UK) consortium	Jacquelyn Wynn, Mairead Hyland, The Lighthouse Lab in Alderley Park and Alex Alderton, Roberto Amato, Sonia Goncalves, Ewan Harrison, David K. Jackson, Ian Johnston, Dominic Kwiatkowski, Cordelia Langford, John Sillitoe on behalf of the Wellcome Sanger Institute COVID-19 Surveillance Team (http://www.sanger.ac.uk/covid-team)
EPI_ISL_624105	Lighthouse Lab in Glasgow	Wellcome Sanger Institute for the COVID-19 Genomics UK (COG-UK) consortium	Harper VanSteenhouse, Yumi Kasai, David Gray, Carol Clugston, Anna Dominiczak and Alex Alderton, Roberto Amato, Sonia Goncalves, Ewan Harrison, David K. Jackson, Ian Johnston, Dominic Kwiatkowski, Cordelia Langford, John Sillitoe on behalf of the Wellcome Sanger Institute COVID-19 Surveillance Team (http://www.sanger.ac.uk/covid-team)
EPI_ISL_624106, EPI_ISL_624107, EPI_ISL_624108, EPI_ISL_624109, EPI_ISL_624110, EPI_ISL_624111	Lighthouse Lab in Alderley Park	Wellcome Sanger Institute for the COVID-19 Genomics UK (COG-UK) consortium	Jacquelyn Wynn, Mairead Hyland, The Lighthouse Lab in Alderley Park and Alex Alderton, Roberto Amato, Sonia Goncalves, Ewan Harrison, David K. Jackson, Ian Johnston, Dominic Kwiatkowski, Cordelia Langford, John Sillitoe on behalf of the Wellcome Sanger Institute COVID-19 Surveillance Team (http://www.sanger.ac.uk/covid-team)
EPI_ISL_624112	Lighthouse Lab in Glasgow	Wellcome Sanger Institute for the COVID-19 Genomics UK (COG-UK) consortium	Harper VanSteenhouse, Yumi Kasai, David Gray, Carol Clugston, Anna Dominiczak and Alex Alderton, Roberto Amato, Sonia Goncalves, Ewan Harrison, David K. Jackson, Ian Johnston, Dominic Kwiatkowski, Cordelia Langford, John Sillitoe on behalf of the Wellcome Sanger Institute COVID-19 Surveillance Team (http://www.sanger.ac.uk/covid-team)
EPI_ISL_624113	Lighthouse Lab in Alderley Park	Wellcome Sanger Institute for the COVID-19 Genomics UK (COG-UK) consortium	Jacquelyn Wynn, Mairead Hyland, The Lighthouse Lab in Alderley Park and Alex Alderton, Roberto Amato, Sonia Goncalves, Ewan Harrison, David K. Jackson, Ian Johnston, Dominic Kwiatkowski, Cordelia Langford, John Sillitoe on behalf of the Wellcome Sanger Institute COVID-19 Surveillance Team (http://www.sanger.ac.uk/covid-team)
EPI_ISL_624114	Lighthouse Lab in Glasgow	Wellcome Sanger Institute for the COVID-19 Genomics UK (COG-UK) consortium	Harper VanSteenhouse, Yumi Kasai, David Gray, Carol Clugston, Anna Dominiczak and Alex Alderton, Roberto Amato, Sonia Goncalves, Ewan Harrison, David K. Jackson, Ian Johnston, Dominic Kwiatkowski, Cordelia Langford, John Sillitoe on behalf of the Wellcome Sanger Institute COVID-19 Surveillance Team (http://www.sanger.ac.uk/covid-team)
EPI_ISL_624115, EPI_ISL_624116, EPI_ISL_624117, EPI_ISL_624118	Lighthouse Lab in Alderley Park	Wellcome Sanger Institute for the COVID-19 Genomics UK (COG-UK) consortium	Jacquelyn Wynn, Mairead Hyland, The Lighthouse Lab in Alderley Park and Alex Alderton, Roberto Amato, Sonia Goncalves, Ewan Harrison, David K. Jackson, Ian Johnston, Dominic Kwiatkowski, Cordelia Langford, John Sillitoe on behalf of the Wellcome Sanger Institute COVID-19 Surveillance Team (http://www.sanger.ac.uk/covid-team)
EPI_ISL_624120	Lighthouse Lab in Glasgow	Wellcome Sanger Institute for the COVID-19 Genomics UK (COG-UK) consortium	Harper VanSteenhouse, Yumi Kasai, David Gray, Carol Clugston, Anna Dominiczak and Alex Alderton, Roberto Amato, Sonia Goncalves, Ewan Harrison, David K. Jackson, Ian Johnston, Dominic Kwiatkowski, Cordelia Langford, John Sillitoe on behalf of the Wellcome Sanger Institute COVID-19 Surveillance Team (http://www.sanger.ac.uk/covid-team)
EPI_ISL_624121	Lighthouse Lab in Alderley Park	Wellcome Sanger Institute for the COVID-19 Genomics UK (COG-UK) consortium	Jacquelyn Wynn, Mairead Hyland, The Lighthouse Lab in Alderley Park and Alex Alderton, Roberto Amato, Sonia Goncalves, Ewan Harrison, David K. Jackson, Ian Johnston, Dominic Kwiatkowski, Cordelia Langford, John Sillitoe on behalf of the Wellcome Sanger Institute COVID-19 Surveillance Team (http://www.sanger.ac.uk/covid-team)
EPI_ISL_624122	Lighthouse Lab in Glasgow	Wellcome Sanger Institute for the COVID-19 Genomics UK (COG-UK) consortium	Harper VanSteenhouse, Yumi Kasai, David Gray, Carol Clugston, Anna Dominiczak and Alex Alderton, Roberto Amato, Sonia Goncalves, Ewan Harrison, David K. Jackson, Ian Johnston, Dominic Kwiatkowski, Cordelia Langford, John Sillitoe on behalf of the Wellcome Sanger Institute COVID-19 Surveillance Team (http://www.sanger.ac.uk/covid-team)
EPI_ISL_624124, EPI_ISL_624125, EPI_ISL_624126, EPI_ISL_624127, EPI_ISL_624129, EPI_ISL_624130	Lighthouse Lab in Alderley Park	Wellcome Sanger Institute for the COVID-19 Genomics UK (COG-UK) consortium	Jacquelyn Wynn, Mairead Hyland, The Lighthouse Lab in Alderley Park and Alex Alderton, Roberto Amato, Sonia Goncalves, Ewan Harrison, David K. Jackson, Ian Johnston, Dominic Kwiatkowski, Cordelia Langford, John Sillitoe on behalf of the Wellcome Sanger Institute COVID-19 Surveillance Team (http://www.sanger.ac.uk/covid-team)
EPI_ISL_624132	Lighthouse Lab in Glasgow	Wellcome Sanger Institute for the COVID-19 Genomics UK (COG-UK) consortium	Harper VanSteenhouse, Yumi Kasai, David Gray, Carol Clugston, Anna Dominiczak and Alex Alderton, Roberto Amato, Sonia Goncalves, Ewan Harrison, David K. Jackson, Ian Johnston, Dominic Kwiatkowski, Cordelia Langford, John Sillitoe on behalf of the Wellcome Sanger Institute COVID-19 Surveillance Team (http://www.sanger.ac.uk/covid-team)
EPI_ISL_624134, EPI_ISL_624136, EPI_ISL_624137, EPI_ISL_624138, EPI_ISL_624139	Lighthouse Lab in Alderley Park	Wellcome Sanger Institute for the COVID-19 Genomics UK (COG-UK) consortium	Jacquelyn Wynn, Mairead Hyland, The Lighthouse Lab in Alderley Park and Alex Alderton, Roberto Amato, Sonia Goncalves, Ewan Harrison, David K. Jackson, Ian Johnston, Dominic Kwiatkowski, Cordelia Langford, John Sillitoe on behalf of the Wellcome Sanger Institute COVID-19 Surveillance Team (http://www.sanger.ac.uk/covid-team)
EPI_ISL_624140	Lighthouse Lab in Glasgow	Wellcome Sanger Institute for the COVID-19 Genomics UK (COG-UK) consortium	Harper VanSteenhouse, Yumi Kasai, David Gray, Carol Clugston, Anna Dominiczak and Alex Alderton, Roberto Amato, Sonia Goncalves, Ewan Harrison, David K. Jackson, Ian Johnston, Dominic Kwiatkowski, Cordelia Langford, John Sillitoe on behalf of the Wellcome Sanger Institute COVID-19 Surveillance Team (http://www.sanger.ac.uk/covid-team)
EPI_ISL_624141, EPI_ISL_624142, EPI_ISL_624143, EPI_ISL_624144	Lighthouse Lab in Alderley Park	Wellcome Sanger Institute for the COVID-19 Genomics UK (COG-UK) consortium	Jacquelyn Wynn, Mairead Hyland, The Lighthouse Lab in Alderley Park and Alex Alderton, Roberto Amato, Sonia Goncalves, Ewan Harrison, David K. Jackson, Ian Johnston, Dominic Kwiatkowski, Cordelia Langford, John Sillitoe on behalf of the Wellcome Sanger Institute COVID-19 Surveillance Team (http://www.sanger.ac.uk/covid-team)

EPI_ISL_624145	Lighthouse Lab in Glasgow	Wellcome Sanger Institute for the COVID-19 Genomics UK (COG-UK) consortium	Harper VanSteenhouse, Yumi Kasai, David Gray, Carol Clugston, Anna Dominiczak and Alex Alderton, Roberto Amato, Sonia Goncalves, Ewan Harrison, David K. Jackson, Ian Johnston, Dominic Kwiatkowski, Cordelia Langford, John Sillitoe on behalf of the Wellcome Sanger Institute COVID-19 Surveillance Team (http://www.sanger.ac.uk/covid-team)
EPI_ISL_624146	Lighthouse Lab in Alderley Park	Wellcome Sanger Institute for the COVID-19 Genomics UK (COG-UK) consortium	Jacquelyn Wynn, Mairead Hyland, The Lighthouse Lab in Alderley Park and Alex Alderton, Roberto Amato, Sonia Goncalves, Ewan Harrison, David K. Jackson, Ian Johnston, Dominic Kwiatkowski, Cordelia Langford, John Sillitoe on behalf of the Wellcome Sanger Institute COVID-19 Surveillance Team (http://www.sanger.ac.uk/covid-team)
EPI_ISL_624147, EPI_ISL_624148	Lighthouse Lab in Glasgow	Wellcome Sanger Institute for the COVID-19 Genomics UK (COG-UK) consortium	Harper VanSteenhouse, Yumi Kasai, David Gray, Carol Clugston, Anna Dominiczak and Alex Alderton, Roberto Amato, Sonia Goncalves, Ewan Harrison, David K. Jackson, Ian Johnston, Dominic Kwiatkowski, Cordelia Langford, John Sillitoe on behalf of the Wellcome Sanger Institute COVID-19 Surveillance Team (http://www.sanger.ac.uk/covid-team)
EPI_ISL_624149, EPI_ISL_624150, EPI_ISL_624151, EPI_ISL_624152, EPI_ISL_624153, EPI_ISL_624154, EPI_ISL_624155, EPI_ISL_624156, EPI_ISL_624157, EPI_ISL_624159, EPI_ISL_624160, EPI_ISL_624161, EPI_ISL_624162			
see above	Lighthouse Lab in Alderley Park	Wellcome Sanger Institute for the COVID-19 Genomics UK (COG-UK) consortium	Jacquelyn Wynn, Mairead Hyland, The Lighthouse Lab in Alderley Park and Alex Alderton, Roberto Amato, Sonia Goncalves, Ewan Harrison, David K. Jackson, Ian Johnston, Dominic Kwiatkowski, Cordelia Langford, John Sillitoe on behalf of the Wellcome Sanger Institute COVID-19 Surveillance Team (http://www.sanger.ac.uk/covid-team)
EPI_ISL_624164, EPI_ISL_624165	Lighthouse Lab in Glasgow	Wellcome Sanger Institute for the COVID-19 Genomics UK (COG-UK) consortium	Harper VanSteenhouse, Yumi Kasai, David Gray, Carol Clugston, Anna Dominiczak and Alex Alderton, Roberto Amato, Sonia Goncalves, Ewan Harrison, David K. Jackson, Ian Johnston, Dominic Kwiatkowski, Cordelia Langford, John Sillitoe on behalf of the Wellcome Sanger Institute COVID-19 Surveillance Team (http://www.sanger.ac.uk/covid-team)
EPI_ISL_624167, EPI_ISL_624168, EPI_ISL_624169, EPI_ISL_624170, EPI_ISL_624171, EPI_ISL_624172, EPI_ISL_624175	Lighthouse Lab in Alderley Park	Wellcome Sanger Institute for the COVID-19 Genomics UK (COG-UK) consortium	Jacquelyn Wynn, Mairead Hyland, The Lighthouse Lab in Alderley Park and Alex Alderton, Roberto Amato, Sonia Goncalves, Ewan Harrison, David K. Jackson, Ian Johnston, Dominic Kwiatkowski, Cordelia Langford, John Sillitoe on behalf of the Wellcome Sanger Institute COVID-19 Surveillance Team (http://www.sanger.ac.uk/covid-team)
EPI_ISL_624176	Lighthouse Lab in Glasgow	Wellcome Sanger Institute for the COVID-19 Genomics UK (COG-UK) consortium	Harper VanSteenhouse, Yumi Kasai, David Gray, Carol Clugston, Anna Dominiczak and Alex Alderton, Roberto Amato, Sonia Goncalves, Ewan Harrison, David K. Jackson, Ian Johnston, Dominic Kwiatkowski, Cordelia Langford, John Sillitoe on behalf of the Wellcome Sanger Institute COVID-19 Surveillance Team (http://www.sanger.ac.uk/covid-team)
EPI_ISL_624177, EPI_ISL_624178, EPI_ISL_624179	Lighthouse Lab in Alderley Park	Wellcome Sanger Institute for the COVID-19 Genomics UK (COG-UK) consortium	Jacquelyn Wynn, Mairead Hyland, The Lighthouse Lab in Alderley Park and Alex Alderton, Roberto Amato, Sonia Goncalves, Ewan Harrison, David K. Jackson, Ian Johnston, Dominic Kwiatkowski, Cordelia Langford, John Sillitoe on behalf of the Wellcome Sanger Institute COVID-19 Surveillance Team (http://www.sanger.ac.uk/covid-team)
EPI_ISL_624181	Lighthouse Lab in Glasgow	Wellcome Sanger Institute for the COVID-19 Genomics UK (COG-UK) consortium	Harper VanSteenhouse, Yumi Kasai, David Gray, Carol Clugston, Anna Dominiczak and Alex Alderton, Roberto Amato, Sonia Goncalves, Ewan Harrison, David K. Jackson, Ian Johnston, Dominic Kwiatkowski, Cordelia Langford, John Sillitoe on behalf of the Wellcome Sanger Institute COVID-19 Surveillance Team (http://www.sanger.ac.uk/covid-team)
EPI_ISL_624182, EPI_ISL_624183, EPI_ISL_624184, EPI_ISL_624185	Lighthouse Lab in Alderley Park	Wellcome Sanger Institute for the COVID-19 Genomics UK (COG-UK) consortium	Jacquelyn Wynn, Mairead Hyland, The Lighthouse Lab in Alderley Park and Alex Alderton, Roberto Amato, Sonia Goncalves, Ewan Harrison, David K. Jackson, Ian Johnston, Dominic Kwiatkowski, Cordelia Langford, John Sillitoe on behalf of the Wellcome Sanger Institute COVID-19 Surveillance Team (http://www.sanger.ac.uk/covid-team)
EPI_ISL_624188, EPI_ISL_624189	Lighthouse Lab in Glasgow	Wellcome Sanger Institute for the COVID-19 Genomics UK (COG-UK) consortium	Harper VanSteenhouse, Yumi Kasai, David Gray, Carol Clugston, Anna Dominiczak and Alex Alderton, Roberto Amato, Sonia Goncalves, Ewan Harrison, David K. Jackson, Ian Johnston, Dominic Kwiatkowski, Cordelia Langford, John Sillitoe on behalf of the Wellcome Sanger Institute COVID-19 Surveillance Team (http://www.sanger.ac.uk/covid-team)
EPI_ISL_624190, EPI_ISL_624191, EPI_ISL_624193, EPI_ISL_624194, EPI_ISL_624195, EPI_ISL_624198, EPI_ISL_624201, EPI_ISL_624202, EPI_ISL_624203, EPI_ISL_624204, EPI_ISL_624205, EPI_ISL_624206, EPI_ISL_624207, EPI_ISL_624208, EPI_ISL_624209, EPI_ISL_624210, EPI_ISL_624211, EPI_ISL_624213, EPI_ISL_624214, EPI_ISL_624215, EPI_ISL_624216, EPI_ISL_624217, EPI_ISL_624218, EPI_ISL_624219, EPI_ISL_624220, EPI_ISL_624221, EPI_ISL_624222, EPI_ISL_624223, EPI_ISL_624224, EPI_ISL_624225, EPI_ISL_624227, EPI_ISL_624228, EPI_ISL_624229, EPI_ISL_624230, EPI_ISL_624231, EPI_ISL_624232, EPI_ISL_624233, EPI_ISL_624234, EPI_ISL_624235, EPI_ISL_624236, EPI_ISL_624237, EPI_ISL_624238, EPI_ISL_624239, EPI_ISL_624240, EPI_ISL_624241, EPI_ISL_624242, EPI_ISL_624243, EPI_ISL_624245, EPI_ISL_624247, EPI_ISL_624249, EPI_ISL_624250, EPI_ISL_624251, EPI_ISL_624253, EPI_ISL_624255, EPI_ISL_624256, EPI_ISL_624257, EPI_ISL_624258, EPI_ISL_624259, EPI_ISL_624260, EPI_ISL_624261, EPI_ISL_624262, EPI_ISL_624263, EPI_ISL_624264, EPI_ISL_624265, EPI_ISL_624267, EPI_ISL_624268, EPI_ISL_624269, EPI_ISL_624270, EPI_ISL_624271, EPI_ISL_624272, EPI_ISL_624273, EPI_ISL_624274, EPI_ISL_624275, EPI_ISL_624276, EPI_ISL_624277, EPI_ISL_624278, EPI_ISL_624279, EPI_ISL_624280, EPI_ISL_624281, EPI_ISL_624283, EPI_ISL_624284, EPI_ISL_624285, EPI_ISL_624286, EPI_ISL_624287, EPI_ISL_624288, EPI_ISL_624289, EPI_ISL_624290, EPI_ISL_624292, EPI_ISL_624293, EPI_ISL_624294, EPI_ISL_624295, EPI_ISL_624296, EPI_ISL_624297, EPI_ISL_624298, EPI_ISL_624300, EPI_ISL_624301, EPI_ISL_624302, EPI_ISL_624304, EPI_ISL_624305, EPI_ISL_624306, EPI_ISL_624308, EPI_ISL_624309, EPI_ISL_624310, EPI_ISL_624311, EPI_ISL_624312, EPI_ISL_624313, EPI_ISL_624314, EPI_ISL_624315, EPI_ISL_624316, EPI_ISL_624317, EPI_ISL_624318, EPI_ISL_624319, EPI_ISL_624320, EPI_ISL_624321, EPI_ISL_624322, EPI_ISL_624323, EPI_ISL_624324, EPI_ISL_624325, EPI_ISL_624326, EPI_ISL_624327, EPI_ISL_624328, EPI_ISL_624330, EPI_ISL_624331, EPI_ISL_624332, EPI_ISL_624333, EPI_ISL_624334, EPI_ISL_624336, EPI_ISL_624337, EPI_ISL_624338, EPI_ISL_624339, EPI_ISL_624340, EPI_ISL_624341, EPI_ISL_624342, EPI_ISL_624343, EPI_ISL_624344, EPI_ISL_624345, EPI_ISL_624346, EPI_ISL_624347, EPI_ISL_624348, EPI_ISL_624349, EPI_ISL_624351, EPI_ISL_624352, EPI_ISL_624353, EPI_ISL_624355, EPI_ISL_624357, EPI_ISL_624358, EPI_ISL_624359, EPI_ISL_624360, EPI_ISL_624361, EPI_ISL_624362, EPI_ISL_624363, EPI_ISL_624364, EPI_ISL_624365, EPI_ISL_624366, EPI_ISL_624367, EPI_ISL_624368, EPI_ISL_624369, EPI_ISL_624370, EPI_ISL_624371, EPI_ISL_624373, EPI_ISL_624374, EPI_ISL_624375, EPI_ISL_624376, EPI_ISL_624377, EPI_ISL_624378, EPI_ISL_624379, EPI_ISL_624380, EPI_ISL_624381, EPI_ISL_624382, EPI_ISL_624383, EPI_ISL_624384, EPI_ISL_624385, EPI_ISL_624386, EPI_ISL_624387, EPI_ISL_624388, EPI_ISL_624389, EPI_ISL_624390, EPI_ISL_624391, EPI_ISL_624392, EPI_ISL_624393, EPI_ISL_624394, EPI_ISL_624395, EPI_ISL_624396, EPI_ISL_624397, EPI_ISL_624398, EPI_ISL_624399, EPI_ISL_624400, EPI_ISL_624401, EPI_ISL_624402, EPI_ISL_624403, EPI_ISL_624404, EPI_ISL_624405, EPI_ISL_624406, EPI_ISL_624407, EPI_ISL_624408, EPI_ISL_624409, EPI_ISL_624410, EPI_ISL_624411, EPI_ISL_624412, EPI_ISL_624413, EPI_ISL_624414, EPI_ISL_624415, EPI_ISL_624416, EPI_ISL_624417, EPI_ISL_624419, EPI_ISL_624420, EPI_ISL_624421, EPI_ISL_624422, EPI_ISL_624423, EPI_ISL_624424, EPI_ISL_624425, EPI_ISL_624426, EPI_ISL_624427, EPI_ISL_624428, EPI_ISL_624429, EPI_ISL_624430, EPI_ISL_624431, EPI_ISL_624432, EPI_ISL_624433, EPI_ISL_624434, EPI_ISL_624435, EPI_ISL_624436, EPI_ISL_624437, EPI_ISL_624438, EPI_ISL_624439, EPI_ISL_624440, EPI_ISL_624441, EPI_ISL_624442, EPI_ISL_624443, EPI_ISL_624444, EPI_ISL_624446, EPI_ISL_624448, EPI_ISL_624449, EPI_ISL_624450, EPI_ISL_624451, EPI_ISL_624453, EPI_ISL_624454, EPI_ISL_624455, EPI_ISL_624456, EPI_ISL_624457, EPI_ISL_624458, EPI_ISL_624459, EPI_ISL_624460, EPI_ISL_624461, EPI_ISL_624462, EPI_ISL_624463, EPI_ISL_624464, EPI_ISL_624465, EPI_ISL_624466, EPI_ISL_624467, EPI_ISL_624468, EPI_ISL_624469, EPI_ISL_624470, EPI_ISL_624471, EPI_ISL_624472, EPI_ISL_624473, EPI_ISL_624474, EPI_ISL_624475, EPI_ISL_624476, EPI_ISL_624477, EPI_ISL_624478, EPI_ISL_624479, EPI_ISL_624480, EPI_ISL_624481, EPI_ISL_624482, EPI_ISL_624483, EPI_ISL_624485, EPI_ISL_624486, EPI_ISL_624487, EPI_ISL_624489, EPI_ISL_624490, EPI_ISL_624491, EPI_ISL_624492, EPI_ISL_624493, EPI_ISL_624494, EPI_ISL_624495, EPI_ISL_624496, EPI_ISL_624497, EPI_ISL_624498, EPI_ISL_624500, EPI_ISL_624501, EPI_ISL_624502, EPI_ISL_624504, EPI_ISL_624505, EPI_ISL_624506, EPI_ISL_624507, EPI_ISL_624508, EPI_ISL_624509, EPI_ISL_624510, EPI_ISL_624511, EPI_ISL_624512, EPI_ISL_624513, EPI_ISL_624515, EPI_ISL_624516, EPI_ISL_624518, EPI_ISL_624520, EPI_ISL_624521, EPI_ISL_624522, EPI_ISL_624523, EPI_ISL_624524, EPI_ISL_624525, EPI_ISL_624526, EPI_ISL_624527, EPI_ISL_624528, EPI_ISL_624529, EPI_ISL_624531, EPI_ISL_624532, EPI_ISL_624533, EPI_ISL_624534, EPI_ISL_624535, EPI_ISL_624536, EPI_ISL_624537, EPI_ISL_624539, EPI_ISL_624540, EPI_ISL_624541, EPI_ISL_624542, EPI_ISL_624543, EPI_ISL_624544, EPI_ISL_624545, EPI_ISL_624547, EPI_ISL_624548, EPI_ISL_624549, EPI_ISL_624550, EPI_ISL_624551, EPI_ISL_624552, EPI_ISL_624553, EPI_ISL_624554, EPI_ISL_624555, EPI_ISL_624556, EPI_ISL_624557, EPI_ISL_624559, EPI_ISL_624560, EPI_ISL_624561, EPI_ISL_624563, EPI_ISL_624564, EPI_ISL_624565, EPI_ISL_624566, EPI_ISL_624567, EPI_ISL_624568, EPI_ISL_624569, EPI_ISL_624570, EPI_ISL_624571, EPI_ISL_624572, EPI_ISL_624573, EPI_ISL_624574, EPI_ISL_624575, EPI_ISL_624576, EPI_ISL_624578, EPI_ISL_624579, EPI_ISL_624580, EPI_ISL_624581, EPI_ISL_624582, EPI_ISL_624583, EPI_ISL_624584, EPI_ISL_624585, EPI_ISL_624586, EPI_ISL_624587, EPI_ISL_624588, EPI_ISL_624589, EPI_ISL_624590, EPI_ISL_624591, EPI_ISL_624592, EPI_ISL_624594, EPI_ISL_624595, EPI_ISL_624596, EPI_ISL_624597, EPI_ISL_624599, EPI_ISL_624600, EPI_ISL_624601, EPI_ISL_624603, EPI_ISL_624604, EPI_ISL_624605, EPI_ISL_624606, EPI_ISL_624607, EPI_ISL_624608, EPI_ISL_624609, EPI_ISL_624610, EPI_ISL_624611, EPI_ISL_624612, EPI_ISL_624614, EPI_ISL_624615, EPI_ISL_624616, EPI_ISL_624620, EPI_ISL_624621, EPI_ISL_624622, EPI_ISL_624623, EPI_ISL_624624, EPI_ISL_624625, EPI_ISL_624626, EPI_ISL_624627, EPI_ISL_624628, EPI_ISL_624630, EPI_ISL_624631, EPI_ISL_624632, EPI_ISL_624633, EPI_ISL_624635, EPI_ISL_624637, EPI_ISL_624638, EPI_ISL_624639, EPI_ISL_624640, EPI_ISL_624641, EPI_ISL_624642, EPI_ISL_624643, EPI_ISL_624644, EPI_ISL_624647, EPI_ISL_624648, EPI_ISL_624650, EPI_ISL_624651, EPI_ISL_624652, EPI_ISL_624653, EPI_ISL_624654, EPI_ISL_624655, EPI_ISL_624656, EPI_ISL_624658, EPI_ISL_624659, EPI_ISL_624660, EPI_ISL_624661, EPI_ISL_624662, EPI_ISL_624663, EPI_ISL_624664, EPI_ISL_624665, EPI_ISL_624666, EPI_ISL_624667, EPI_ISL_624668, EPI_ISL_624669, EPI_ISL_624670, EPI_ISL_624672, EPI_ISL_624673, EPI_ISL_624674, EPI_ISL_624675, EPI_ISL_624676, EPI_ISL_624677, EPI_ISL_624678, EPI_ISL_624679, EPI_ISL_624680, EPI_ISL_624681, EPI_ISL_624682, EPI_ISL_624683, EPI_ISL_624684, EPI_ISL_624686, EPI_ISL_624688, EPI_ISL_624689, EPI_ISL_624690, EPI_ISL_624691, EPI_ISL_624692, EPI_ISL_624694, EPI_ISL_624696, EPI_ISL_624697, EPI_ISL_624698, EPI_ISL_624700, EPI_ISL_624701, EPI_ISL_624702, EPI_ISL_624703, EPI_ISL_624704, EPI_ISL_624705, EPI_ISL_624706, EPI_ISL_624707, EPI_ISL_624708, EPI_ISL_624709, EPI_ISL_624710, EPI_ISL_624711, EPI_ISL_624712, EPI_ISL_624713, EPI_ISL_624716, EPI_ISL_624717, EPI_ISL_624718, EPI_ISL_624719, EPI_ISL_624720, EPI_ISL_624721, EPI_ISL_624722, EPI_ISL_624723, EPI_ISL_624725, EPI_ISL_624726, EPI_ISL_624727, EPI_ISL_624729, EPI_ISL_624730, EPI_ISL_624731, EPI_ISL_624732, EPI_ISL_624733, EPI_ISL_624734, EPI_ISL_624735, EPI_ISL_624736, EPI_ISL_624737, EPI_ISL_624738, EPI_ISL_624739, EPI_ISL_624741, EPI_ISL_624743, EPI_ISL_624744, EPI_ISL_624745, EPI_ISL_624747, EPI_ISL_624748, EPI_ISL_624749, EPI_ISL_624750, EPI_ISL_624751, EPI_ISL_624752, EPI_ISL_624753, EPI_ISL_624754, EPI_ISL_624755, EPI_ISL_624756, EPI_ISL_624757, EPI_ISL_624759, EPI_ISL_624760, EPI_ISL_624762, EPI_ISL_624763, EPI_ISL_624764, EPI_ISL_624765, EPI_ISL_624766, EPI_ISL_624767, EPI_ISL_624768, EPI_ISL_624769, EPI_ISL_624771, EPI_ISL_624772, EPI_ISL_624773, EPI_ISL_624774, EPI_ISL_624776, EPI_ISL_624777, EPI_ISL_624779, EPI_ISL_624780, EPI_ISL_624781, EPI_ISL_624782, EPI_ISL_624783, EPI_ISL_624784, EPI_ISL_624785, EPI_ISL_624786, EPI_ISL_624787, EPI_ISL_624789, EPI_ISL_624790, EPI_ISL_624791, EPI_ISL_624792, EPI_ISL_624793, EPI_ISL_624795, EPI_ISL_624796, EPI_ISL_624797, EPI_ISL_624800, EPI_ISL_624801, EPI_ISL_624802, EPI_ISL_624803, EPI_ISL_624804, EPI_ISL_624805, EPI_ISL_624806, EPI_ISL_624808, EPI_ISL_624809, EPI_ISL_624810, EPI_ISL_624811, EPI_ISL_624812, EPI_ISL_624813, EPI_ISL_624814, EPI_ISL_624815, EPI_ISL_624816, EPI_ISL_624817, EPI_ISL_624819, EPI_ISL_624820, EPI_ISL_624821, EPI_ISL_624823, EPI_ISL_624824, EPI_ISL_624825, EPI_ISL_624826, EPI_ISL_624827, EPI_ISL_624828, EPI_ISL_624829, EPI_ISL_624830, EPI_ISL_624831, EPI_ISL_624832, EPI_ISL_624833, EPI_ISL_624834, EPI_ISL_624836, EPI_ISL_624837, EPI_ISL_624838, EPI_ISL_624839, EPI_ISL_624840, EPI_ISL_624841, EPI_ISL_624847, EPI_ISL_624848, EPI_ISL_624849, EPI_ISL_624851, EPI_ISL_624852, EPI_ISL_624853, EPI_ISL_624854, EPI_ISL_624855, EPI_ISL_624856, EPI_ISL_624858, EPI_ISL_624859, EPI_ISL_624860, EPI_ISL_624861, EPI_ISL_624862, EPI_ISL_624863, EPI_ISL_624865, EPI_ISL_624866, EPI_ISL_624867, EPI_ISL_624868, EPI_ISL_624869, EPI_ISL_624870, EPI_ISL_624872, EPI_ISL_624873, EPI_ISL_624874, EPI_ISL_624875, EPI_ISL_624876, EPI_ISL_624877, EPI_ISL_624878, EPI_ISL_624879, EPI_ISL_624880, EPI_ISL_624881, EPI_ISL_624882, EPI_ISL_624883, EPI_ISL_624884, EPI_ISL_624886, EPI_ISL_624887, EPI_ISL_624888, EPI_ISL_624889, EPI_ISL_624890, EPI_ISL_624891, EPI_ISL_624892, EPI_ISL_624893, EPI_ISL_624894, EPI_ISL_624895, EPI_ISL_624896, EPI_ISL_624898, EPI_ISL_624899, EPI_ISL_624900, EPI_ISL_624901, EPI_ISL_624902, EPI_ISL_624904, EPI_ISL_624905, EPI_ISL_624906, EPI_ISL_624908, EPI_ISL_624910, EPI_ISL_624912, EPI_ISL_624913, EPI_ISL_624914, EPI_ISL_624915, EPI_ISL_624916, EPI_ISL_624917, EPI_ISL_624918, EPI_ISL_624919, EPI_ISL_624921, EPI_ISL_624922, EPI_ISL_624923, EPI_ISL_624924, EPI_ISL_624925, EPI_ISL_624926, EPI_ISL_624927, EPI_ISL_624928, EPI_ISL_624929, EPI_ISL_624930, EPI_ISL_624931, EPI_ISL_624932, EPI_ISL_624933, EPI_ISL_624934, EPI_ISL_624935, EPI_ISL_624936, EPI_ISL_624937, EPI_ISL_624938, EPI_ISL_624939, EPI_ISL_624940, EPI_ISL_624942, EPI_ISL_624943, EPI_ISL_624944, EPI_ISL_624945, EPI_ISL_624946, EPI_ISL_624947, EPI_ISL_624949, EPI_ISL_624950, EPI_ISL_624951, EPI_ISL_624952, EPI_ISL_624953, EPI_ISL_624955, EPI_ISL_624956, EPI_ISL_624957, EPI_ISL_624958, EPI_ISL_624959, EPI_ISL_624960, EPI_ISL_624961, EPI_ISL_624962, EPI_ISL_624963, EPI_ISL_624964, EPI_ISL_624965, EPI_ISL_624966, EPI_ISL_624967, EPI_ISL_624968, EPI_ISL_624969, EPI_ISL_624970, EPI_ISL_624971, EPI_ISL_624972, EPI_ISL_624973, EPI_ISL_624974, EPI_ISL_624975, EPI_ISL_624976, EPI_ISL_624977, EPI_ISL_624978, EPI_ISL_624980, EPI_ISL_624981, EPI_ISL_624982, EPI_ISL_624983, EPI_ISL_624984, EPI_ISL_624985, EPI_ISL_624986, EPI_ISL_624988, EPI_ISL_624989, EPI_ISL_624990, EPI_ISL_624991, EPI_ISL_624992, EPI_ISL_624994, EPI_ISL_624996, EPI_ISL_624997, EPI_ISL_624998, EPI_ISL_624999, EPI_ISL_625000, EPI_ISL_625001, EPI_ISL_625002, EPI_ISL_625004, EPI_ISL_625005, EPI_ISL_625007, EPI_ISL_625008, EPI_ISL_625009, EPI_ISL_625010, EPI_ISL_625011, EPI_ISL_625012, EPI_ISL_625014, EPI_ISL_625015, EPI_ISL_625016, EPI_ISL_625019, EPI_ISL_625020, EPI_ISL_625021, EPI_ISL_625022, EPI_ISL_625023, EPI_ISL_625024, EPI_ISL_625025, EPI_ISL_625027, EPI_ISL_625028, EPI_ISL_625029, EPI_ISL_625030, EPI_ISL_625031, EPI_ISL_625032, EPI_ISL_625033, EPI_ISL_625034, EPI_ISL_625035, EPI_ISL_625036, EPI_ISL_625037, EPI_ISL_625038, EPI_ISL_625039, EPI_ISL_625040, EPI_ISL_625041, EPI_ISL_625042, EPI_ISL_625043, EPI_ISL_625044, EPI_ISL_625045, EPI_ISL_625046, EPI_ISL_625047, EPI_ISL_625048, EPI_ISL_625050, EPI_ISL_625051, EPI_ISL_625052, EPI_ISL_625053, EPI_ISL_625054, EPI_ISL_625055, EPI_ISL_625056, EPI_ISL_625057, EPI_ISL_625058, EPI_ISL_625059, EPI_ISL_625060, EPI_ISL_625061, EPI_ISL_625062, EPI_ISL_625063, EPI_ISL_625064, EPI_ISL_625065, EPI_ISL_625067, EPI_ISL_625068, EPI_ISL_625069, EPI_ISL_625070, EPI_ISL_625071, EPI_ISL_625072, EPI_ISL_625073, EPI_ISL_625074, EPI_ISL_625075, EPI_ISL_625076, EPI_ISL_625077, EPI_ISL_625078, EPI_ISL_625079, EPI_ISL_625080, EPI_ISL_625081, EPI_ISL_625082, EPI_ISL_625083, EPI_ISL_625084, EPI_ISL_625085, EPI_ISL_625086, EPI_ISL_625087, EPI_ISL_625088, EPI_ISL_625090, EPI_ISL_625091, EPI_ISL_625093, EPI_ISL_625094, EPI_ISL_625095, EPI_ISL_625096, EPI_ISL_625097, EPI_ISL_625098, EPI_ISL_625099, EPI_ISL_625100, EPI_ISL_625101, EPI_ISL_625102, EPI_ISL_625103, EPI_ISL_625104, EPI_ISL_625105, EPI_ISL_625106, EPI_ISL_625107, EPI_ISL_625108, EPI_ISL_625109, EPI_ISL_625110, EPI_ISL_625111, EPI_ISL_625112, EPI_ISL_625113, EPI_ISL_625114, EPI_ISL_625115, EPI_ISL_625116, EPI_ISL_625117, EPI_ISL_625118, EPI_ISL_625119, EPI_ISL_625120, EPI_ISL_625122, EPI_ISL_625123, EPI_ISL_625124, EPI_ISL_625125, EPI_ISL_625126, EPI_ISL_625127, EPI_ISL_625129, EPI_ISL_625130, EPI_ISL_625131, EPI_ISL_625132, EPI_ISL_625134, EPI_ISL_625135, EPI_ISL_625136, EPI_ISL_625137, EPI_ISL_625138, EPI_ISL_625139, EPI_ISL_625140, EPI_ISL_625141, EPI_ISL_625142, EPI_ISL_625143, EPI_ISL_625144, EPI_ISL_625147, EPI_ISL_625148, EPI_ISL_625149, EPI_ISL_625150, EPI_ISL_625152, EPI_ISL_625153, EPI_ISL_625154, EPI_ISL_625155,			

EPI_ISL_625156, EPI_ISL_625157, EPI_ISL_625158, EPI_ISL_625159, EPI_ISL_625160, EPI_ISL_625161, EPI_ISL_625162, EPI_ISL_625163, EPI_ISL_625164, EPI_ISL_625165, EPI_ISL_625166, EPI_ISL_625167, EPI_ISL_625169, EPI_ISL_625170, EPI_ISL_625171, EPI_ISL_625172, EPI_ISL_625173, EPI_ISL_625174, EPI_ISL_625175, EPI_ISL_625176, EPI_ISL_625177, EPI_ISL_625178, EPI_ISL_625179, EPI_ISL_625181, EPI_ISL_625183, EPI_ISL_625184, EPI_ISL_625186, EPI_ISL_625187, EPI_ISL_625188, EPI_ISL_625189, EPI_ISL_625190, EPI_ISL_625191, EPI_ISL_625192, EPI_ISL_625193, EPI_ISL_625194, EPI_ISL_625196, EPI_ISL_625197, EPI_ISL_625198, EPI_ISL_625199, EPI_ISL_625200, EPI_ISL_625201, EPI_ISL_625202, EPI_ISL_625203, EPI_ISL_625204, EPI_ISL_625205, EPI_ISL_625206, EPI_ISL_625207, EPI_ISL_625208, EPI_ISL_625209, EPI_ISL_625210, EPI_ISL_625211, EPI_ISL_625212, EPI_ISL_625214, EPI_ISL_625215, EPI_ISL_625216, EPI_ISL_625217, EPI_ISL_625218, EPI_ISL_625219, EPI_ISL_625220, EPI_ISL_625221, EPI_ISL_625223, EPI_ISL_625224, EPI_ISL_625226, EPI_ISL_625227, EPI_ISL_625228, EPI_ISL_625229, EPI_ISL_625230, EPI_ISL_625231, EPI_ISL_625232, EPI_ISL_625233, EPI_ISL_625234, EPI_ISL_625236, EPI_ISL_625237, EPI_ISL_625238, EPI_ISL_625239, EPI_ISL_625241, EPI_ISL_625242, EPI_ISL_625243, EPI_ISL_625244, EPI_ISL_625245, EPI_ISL_625246, EPI_ISL_625248, EPI_ISL_625249, EPI_ISL_625250, EPI_ISL_625251, EPI_ISL_625252, EPI_ISL_625253, EPI_ISL_625254, EPI_ISL_625255, EPI_ISL_625257, EPI_ISL_625258, EPI_ISL_625259, EPI_ISL_625260, EPI_ISL_625261, EPI_ISL_625262, EPI_ISL_625263, EPI_ISL_625264, EPI_ISL_625265, EPI_ISL_625266, EPI_ISL_625267, EPI_ISL_625268, EPI_ISL_625269, EPI_ISL_625270, EPI_ISL_625271, EPI_ISL_625272, EPI_ISL_625273, EPI_ISL_625274, EPI_ISL_625275, EPI_ISL_625276, EPI_ISL_625277, EPI_ISL_625279, EPI_ISL_625280			
see above	Lighthouse Lab in Alderley Park	Wellcome Sanger Institute for the COVID-19 Genomics UK (COG-UK) consortium	Jacquelyn Wynn, Mairead Hyland, The Lighthouse Lab in Alderley Park and Alex Alderton, Roberto Amato, Sonia Goncalves, Ewan Harrison, David K. Jackson, Ian Johnston, Dominic Kwiatkowski, Cordelia Langford, John Sillitoe on behalf of the Wellcome Sanger Institute COVID-19 Surveillance Team (http://www.sanger.ac.uk/covid-team)
EPI_ISL_625281, EPI_ISL_625282, EPI_ISL_625283, EPI_ISL_625284, EPI_ISL_625285, EPI_ISL_625286, EPI_ISL_625287, EPI_ISL_625288, EPI_ISL_625290, EPI_ISL_625291, EPI_ISL_625292, EPI_ISL_625293, EPI_ISL_625294, EPI_ISL_625295, EPI_ISL_625296, EPI_ISL_625298, EPI_ISL_625299, EPI_ISL_625300, EPI_ISL_625301, EPI_ISL_625302, EPI_ISL_625303, EPI_ISL_625305, EPI_ISL_625306, EPI_ISL_625307, EPI_ISL_625308, EPI_ISL_625309, EPI_ISL_625310, EPI_ISL_625311, EPI_ISL_625313, EPI_ISL_625314			
see above	Lighthouse Lab in Cambridge	Wellcome Sanger Institute for the COVID-19 Genomics UK (COG-UK) consortium	Rob Howes, The Lighthouse Lab in Cambridge and Alex Alderton, Roberto Amato, Sonia Goncalves, Ewan Harrison, David K. Jackson, Ian Johnston, Dominic Kwiatkowski, Cordelia Langford, John Sillitoe on behalf of the Wellcome Sanger Institute COVID-19 Surveillance Team (http://www.sanger.ac.uk/covid-team)
EPI_ISL_625317, EPI_ISL_625319, EPI_ISL_625320, EPI_ISL_625321, EPI_ISL_625322, EPI_ISL_625323, EPI_ISL_625324, EPI_ISL_625325, EPI_ISL_625327, EPI_ISL_625329, EPI_ISL_625330, EPI_ISL_625332, EPI_ISL_625333, EPI_ISL_625335, EPI_ISL_625336, EPI_ISL_625337, EPI_ISL_625338, EPI_ISL_625339, EPI_ISL_625340, EPI_ISL_625341, EPI_ISL_625342, EPI_ISL_625343, EPI_ISL_625344, EPI_ISL_625345, EPI_ISL_625346, EPI_ISL_625347, EPI_ISL_625348, EPI_ISL_625349, EPI_ISL_625350, EPI_ISL_625351, EPI_ISL_625352, EPI_ISL_625353, EPI_ISL_625354, EPI_ISL_625355, EPI_ISL_625356, EPI_ISL_625357, EPI_ISL_625359, EPI_ISL_625361, EPI_ISL_625362, EPI_ISL_625363, EPI_ISL_625364, EPI_ISL_625366, EPI_ISL_625367, EPI_ISL_625368, EPI_ISL_625369, EPI_ISL_625370, EPI_ISL_625371, EPI_ISL_625372, EPI_ISL_625373, EPI_ISL_625374, EPI_ISL_625375, EPI_ISL_625377, EPI_ISL_625378, EPI_ISL_625379, EPI_ISL_625380, EPI_ISL_625381, EPI_ISL_625382, EPI_ISL_625383, EPI_ISL_625384, EPI_ISL_625385, EPI_ISL_625386, EPI_ISL_625387, EPI_ISL_625388, EPI_ISL_625389, EPI_ISL_625390, EPI_ISL_625391, EPI_ISL_625392, EPI_ISL_625393, EPI_ISL_625394, EPI_ISL_625395			
see above	Lighthouse Lab in Glasgow	Wellcome Sanger Institute for the COVID-19 Genomics UK (COG-UK) consortium	Harper VanSteenhouse, Yumi Kasai, David Gray, Carol Clugston, Anna Dominiczak and Alex Alderton, Roberto Amato, Sonia Goncalves, Ewan Harrison, David K. Jackson, Ian Johnston, Dominic Kwiatkowski, Cordelia Langford, John Sillitoe on behalf of the Wellcome Sanger Institute COVID-19 Surveillance Team (http://www.sanger.ac.uk/covid-team)
EPI_ISL_625396, EPI_ISL_625397, EPI_ISL_625398, EPI_ISL_625399, EPI_ISL_625400, EPI_ISL_625403	Lighthouse Lab in Alderley Park	Wellcome Sanger Institute for the COVID-19 Genomics UK (COG-UK) consortium	Jacquelyn Wynn, Mairead Hyland, The Lighthouse Lab in Alderley Park and Alex Alderton, Roberto Amato, Sonia Goncalves, Ewan Harrison, David K. Jackson, Ian Johnston, Dominic Kwiatkowski, Cordelia Langford, John Sillitoe on behalf of the Wellcome Sanger Institute COVID-19 Surveillance Team (http://www.sanger.ac.uk/covid-team)
EPI_ISL_625406, EPI_ISL_625407, EPI_ISL_625409, EPI_ISL_625410, EPI_ISL_625411, EPI_ISL_625412, EPI_ISL_625413, EPI_ISL_625414, EPI_ISL_625415, EPI_ISL_625416, EPI_ISL_625417, EPI_ISL_625418, EPI_ISL_625419, EPI_ISL_625420, EPI_ISL_625421			
see above	Lighthouse Lab in Milton Keynes	Wellcome Sanger Institute for the COVID-19 Genomics UK (COG-UK) consortium	The Lighthouse Lab in Milton Keynes and Alex Alderton, Roberto Amato, Sonia Goncalves, Ewan Harrison, David K. Jackson, Ian Johnston, Dominic Kwiatkowski, Cordelia Langford, John Sillitoe on behalf of the Wellcome Sanger Institute COVID-19 Surveillance Team (http://www.sanger.ac.uk/covid-team)
EPI_ISL_625422, EPI_ISL_625423	Lighthouse Lab in Alderley Park	Wellcome Sanger Institute for the COVID-19 Genomics UK (COG-UK) consortium	Jacquelyn Wynn, Mairead Hyland, The Lighthouse Lab in Alderley Park and Alex Alderton, Roberto Amato, Sonia Goncalves, Ewan Harrison, David K. Jackson, Ian Johnston, Dominic Kwiatkowski, Cordelia Langford, John Sillitoe on behalf of the Wellcome Sanger Institute COVID-19 Surveillance Team (http://www.sanger.ac.uk/covid-team)
EPI_ISL_625424	Lighthouse Lab in Milton Keynes	Wellcome Sanger Institute for the COVID-19 Genomics UK (COG-UK) consortium	The Lighthouse Lab in Milton Keynes and Alex Alderton, Roberto Amato, Sonia Goncalves, Ewan Harrison, David K. Jackson, Ian Johnston, Dominic Kwiatkowski, Cordelia Langford, John Sillitoe on behalf of the Wellcome Sanger Institute COVID-19 Surveillance Team (http://www.sanger.ac.uk/covid-team)
EPI_ISL_625425	Lighthouse Lab in Alderley Park	Wellcome Sanger Institute for the COVID-19 Genomics UK (COG-UK) consortium	Jacquelyn Wynn, Mairead Hyland, The Lighthouse Lab in Alderley Park and Alex Alderton, Roberto Amato, Sonia Goncalves, Ewan Harrison, David K. Jackson, Ian Johnston, Dominic Kwiatkowski, Cordelia Langford, John Sillitoe on behalf of the Wellcome Sanger Institute COVID-19 Surveillance Team (http://www.sanger.ac.uk/covid-team)
EPI_ISL_625426	Lighthouse Lab in Milton Keynes	Wellcome Sanger Institute for the COVID-19 Genomics UK (COG-UK) consortium	The Lighthouse Lab in Milton Keynes and Alex Alderton, Roberto Amato, Sonia Goncalves, Ewan Harrison, David K. Jackson, Ian Johnston, Dominic Kwiatkowski, Cordelia Langford, John Sillitoe on behalf of the Wellcome Sanger Institute COVID-19 Surveillance Team (http://www.sanger.ac.uk/covid-team)
EPI_ISL_625427, EPI_ISL_625429	Lighthouse Lab in Alderley Park	Wellcome Sanger Institute for the COVID-19 Genomics UK (COG-UK) consortium	Jacquelyn Wynn, Mairead Hyland, The Lighthouse Lab in Alderley Park and Alex Alderton, Roberto Amato, Sonia Goncalves, Ewan Harrison, David K. Jackson, Ian Johnston, Dominic Kwiatkowski, Cordelia Langford, John Sillitoe on behalf of the Wellcome Sanger Institute COVID-19 Surveillance Team (http://www.sanger.ac.uk/covid-team)
EPI_ISL_625430	Lighthouse Lab in Milton Keynes	Wellcome Sanger Institute for the COVID-19 Genomics UK (COG-UK) consortium	The Lighthouse Lab in Milton Keynes and Alex Alderton, Roberto Amato, Sonia Goncalves, Ewan Harrison, David K. Jackson, Ian Johnston, Dominic Kwiatkowski, Cordelia Langford, John Sillitoe on behalf of the Wellcome Sanger Institute COVID-19 Surveillance Team (http://www.sanger.ac.uk/covid-team)
EPI_ISL_625431	Lighthouse Lab in Alderley Park	Wellcome Sanger Institute for the COVID-19 Genomics UK (COG-UK) consortium	Jacquelyn Wynn, Mairead Hyland, The Lighthouse Lab in Alderley Park and Alex Alderton, Roberto Amato, Sonia Goncalves, Ewan Harrison, David K. Jackson, Ian Johnston, Dominic Kwiatkowski, Cordelia Langford, John Sillitoe on behalf of the Wellcome Sanger Institute COVID-19 Surveillance Team (http://www.sanger.ac.uk/covid-team)
EPI_ISL_625432	Lighthouse Lab in Cambridge	Wellcome Sanger Institute for the COVID-19 Genomics UK (COG-UK) consortium	Rob Howes, The Lighthouse Lab in Cambridge and Alex Alderton, Roberto Amato, Sonia Goncalves, Ewan Harrison, David K. Jackson, Ian Johnston, Dominic Kwiatkowski, Cordelia Langford, John Sillitoe on behalf of the Wellcome Sanger Institute COVID-19 Surveillance Team (http://www.sanger.ac.uk/covid-team)
EPI_ISL_625433, EPI_ISL_625434, EPI_ISL_625435, EPI_ISL_625436	Lighthouse Lab in Alderley Park	Wellcome Sanger Institute for the COVID-19 Genomics UK (COG-UK) consortium	Jacquelyn Wynn, Mairead Hyland, The Lighthouse Lab in Alderley Park and Alex Alderton, Roberto Amato, Sonia Goncalves, Ewan Harrison, David K. Jackson, Ian Johnston, Dominic Kwiatkowski, Cordelia Langford, John Sillitoe on behalf of the Wellcome Sanger Institute COVID-19 Surveillance Team (http://www.sanger.ac.uk/covid-team)
EPI_ISL_625437, EPI_ISL_625438, EPI_ISL_625439, EPI_ISL_625440, EPI_ISL_625441, EPI_ISL_625442, EPI_ISL_625443, EPI_ISL_625444, EPI_ISL_625446, EPI_ISL_625447, EPI_ISL_625448, EPI_ISL_625449, EPI_ISL_625450, EPI_ISL_625451, EPI_ISL_625452, EPI_ISL_625453			
see above	Lighthouse Lab in Cambridge	Wellcome Sanger Institute for the COVID-19 Genomics UK (COG-UK) consortium	Rob Howes, The Lighthouse Lab in Cambridge and Alex Alderton, Roberto Amato, Sonia Goncalves, Ewan Harrison, David K. Jackson, Ian Johnston, Dominic Kwiatkowski, Cordelia Langford, John Sillitoe on behalf of the Wellcome Sanger Institute COVID-19 Surveillance Team (http://www.sanger.ac.uk/covid-team)
EPI_ISL_625456	Virology Unit, Institut Pasteur de Madagascar	Virology Unit, Institut Pasteur de Madagascar	Christian Ranaivoson, Cara Brook, Norosoa Razanajatovo, Vida Ahyong, Tsiry Randriambolamanantsoa, Michelle Tan, Vololoniaina Raharinosy, Helisoa Razafimanjato, Cristina M. Tato, Joseph L. DeRisi, Soa Fy Andriamandimby, Jean-Michel Heraud, Philippe Dussart
EPI_ISL_625460, EPI_ISL_625461, EPI_ISL_625465, EPI_ISL_625467, EPI_ISL_625471, EPI_ISL_625473, EPI_ISL_625474, EPI_ISL_625475, EPI_ISL_625476, EPI_ISL_625477	Child Health Research Foundation	Child Health Research Foundation	Senjuti Saha, Md Saiful Islam Sajib, Nikkon Sarkar, Syed Muktadir Al Sium, Afroza Akter Tanni, Roly Malaker, Arif Mohammad Tanmoy, Md Hafizur Rahman, Samir K Saha
EPI_ISL_625478, EPI_ISL_625479, EPI_ISL_625480, EPI_ISL_625481, EPI_ISL_625482, EPI_ISL_625484, EPI_ISL_625485, EPI_ISL_625486, EPI_ISL_625487, EPI_ISL_625488, EPI_ISL_625489, EPI_ISL_625490, EPI_ISL_625492, EPI_ISL_625493, EPI_ISL_625494, EPI_ISL_625495, EPI_ISL_625496, EPI_ISL_625497, EPI_ISL_625498, EPI_ISL_625499			
see above	Santa Clara County Public Health Laboratory	Chan-Zuckerberg Biohub	CZB Cliahub Consortium
EPI_ISL_625500, EPI_ISL_625501, EPI_ISL_625502, EPI_ISL_625503, EPI_ISL_625506, EPI_ISL_625507, EPI_ISL_625508, EPI_ISL_625509, EPI_ISL_625510, EPI_ISL_625511, EPI_ISL_625512, EPI_ISL_625514, EPI_ISL_625515, EPI_ISL_625516, EPI_ISL_625517, EPI_ISL_625518, EPI_ISL_625519, EPI_ISL_625520, EPI_ISL_625521, EPI_ISL_625523, EPI_ISL_625524, EPI_ISL_625525, EPI_ISL_625526, EPI_ISL_625527, EPI_ISL_625528, EPI_ISL_625529, EPI_ISL_625530, EPI_ISL_625531, EPI_ISL_625532, EPI_ISL_625534, EPI_ISL_625535, EPI_ISL_625536, EPI_ISL_625537, EPI_ISL_625538, EPI_ISL_625539, EPI_ISL_625540, EPI_ISL_625541, EPI_ISL_625542, EPI_ISL_625543, EPI_ISL_625544, EPI_ISL_625545, EPI_ISL_625546			
see above	Alameda County Public Health Lab	Chan-Zuckerberg Biohub	CZB Cliahub Consortium
EPI_ISL_625548, EPI_ISL_625549	County of San Luis Obispo Public Health Laboratory	Chan-Zuckerberg Biohub	CZB Cliahub Consortium

EPI_ISL_625550, EPI_ISL_625551, EPI_ISL_625552, EPI_ISL_625553, EPI_ISL_625555, EPI_ISL_625556, EPI_ISL_625557, EPI_ISL_625558	UCSF Clinical Microbiology Laboratory	Chan-Zuckerberg Biohub	CZB Cliahub Consortium
EPI_ISL_625559, EPI_ISL_625560, EPI_ISL_625561, EPI_ISL_625562, EPI_ISL_625563, EPI_ISL_625564, EPI_ISL_625565, EPI_ISL_625566, EPI_ISL_625567, EPI_ISL_625568, EPI_ISL_625569, EPI_ISL_625570, EPI_ISL_625571, EPI_ISL_625572, EPI_ISL_625573, EPI_ISL_625574, EPI_ISL_625575, EPI_ISL_625576, EPI_ISL_625578, EPI_ISL_625579, EPI_ISL_625580, EPI_ISL_625581, EPI_ISL_625582, EPI_ISL_625583, EPI_ISL_625584, EPI_ISL_625585, EPI_ISL_625586, EPI_ISL_625587, EPI_ISL_625588, EPI_ISL_625589, EPI_ISL_625590, EPI_ISL_625591, EPI_ISL_625592			
see above	NaN	Chan-Zuckerberg Biohub	CZB Cliahub Consortium
EPI_ISL_625594, EPI_ISL_625595, EPI_ISL_625596, EPI_ISL_625597, EPI_ISL_625598, EPI_ISL_625599, EPI_ISL_625600, EPI_ISL_625601, EPI_ISL_625602, EPI_ISL_625603, EPI_ISL_625605, EPI_ISL_625606, EPI_ISL_625607, EPI_ISL_625608, EPI_ISL_625610			
see above	Santa Clara County Public Health Laboratory	Chan-Zuckerberg Biohub	CZB Cliahub Consortium
EPI_ISL_625611, EPI_ISL_625612, EPI_ISL_625613, EPI_ISL_625614, EPI_ISL_625616, EPI_ISL_625617, EPI_ISL_625619, EPI_ISL_625621, EPI_ISL_625622	Alameda County Public Health Lab	Chan-Zuckerberg Biohub	CZB Cliahub Consortium
EPI_ISL_625623, EPI_ISL_625625, EPI_ISL_625626	County of San Luis Obispo Public Health Laboratory	Chan-Zuckerberg Biohub	CZB Cliahub Consortium
EPI_ISL_625627, EPI_ISL_625628, EPI_ISL_625629, EPI_ISL_625630, EPI_ISL_625636, EPI_ISL_625641, EPI_ISL_625642, EPI_ISL_625643, EPI_ISL_625644, EPI_ISL_625645, EPI_ISL_625647, EPI_ISL_625648, EPI_ISL_625649, EPI_ISL_625650, EPI_ISL_625651, EPI_ISL_625652, EPI_ISL_625654, EPI_ISL_625655, EPI_ISL_625656, EPI_ISL_625657			
see above	Orange County Public Health Lab	Chan-Zuckerberg Biohub	CZB Cliahub Consortium
EPI_ISL_625660	San Joaquin County Public Health Lab	Chan-Zuckerberg Biohub	CZB Cliahub Consortium
EPI_ISL_625662, EPI_ISL_625663, EPI_ISL_625664, EPI_ISL_625665, EPI_ISL_625666, EPI_ISL_625667, EPI_ISL_625668, EPI_ISL_625669, EPI_ISL_625670	UCSF Clinical Microbiology Laboratory	Chan-Zuckerberg Biohub	CZB Cliahub Consortium
EPI_ISL_625673, EPI_ISL_625674, EPI_ISL_625675, EPI_ISL_625676, EPI_ISL_625677, EPI_ISL_625678, EPI_ISL_625679, EPI_ISL_625680, EPI_ISL_625681, EPI_ISL_625682	Laboratory of Molecular Medicine, University of Magallanes	Centro Asistencial Docente y de Investigacion, Universidad de Magallanes	Jorge Gonzalez, Jacqueline Aldridge, Diego Alvarez, Marcelo Navarrete
EPI_ISL_625684, EPI_ISL_625697, EPI_ISL_625703, EPI_ISL_625716, EPI_ISL_625732, EPI_ISL_625733, EPI_ISL_625734, EPI_ISL_625735, EPI_ISL_625736, EPI_ISL_625737, EPI_ISL_625738, EPI_ISL_625739, EPI_ISL_625740, EPI_ISL_625741, EPI_ISL_625746, EPI_ISL_625752, EPI_ISL_625756, EPI_ISL_625757, EPI_ISL_625758, EPI_ISL_625759, EPI_ISL_625760, EPI_ISL_625761, EPI_ISL_625762, EPI_ISL_625763, EPI_ISL_625764, EPI_ISL_625765, EPI_ISL_625766, EPI_ISL_625767, EPI_ISL_625768, EPI_ISL_625769, EPI_ISL_625770, EPI_ISL_625771, EPI_ISL_625772, EPI_ISL_625773, EPI_ISL_625774, EPI_ISL_625775, EPI_ISL_625776, EPI_ISL_625778, EPI_ISL_625779, EPI_ISL_625780, EPI_ISL_625781, EPI_ISL_625782, EPI_ISL_625783, EPI_ISL_625784, EPI_ISL_625785, EPI_ISL_625786, EPI_ISL_625787, EPI_ISL_625788, EPI_ISL_625789, EPI_ISL_625790, EPI_ISL_625791, EPI_ISL_625792, EPI_ISL_625793, EPI_ISL_625794, EPI_ISL_625795, EPI_ISL_625796, EPI_ISL_625797, EPI_ISL_625798, EPI_ISL_625799, EPI_ISL_625800, EPI_ISL_625801, EPI_ISL_625802, EPI_ISL_625803, EPI_ISL_625804, EPI_ISL_625805, EPI_ISL_625806, EPI_ISL_625807, EPI_ISL_625808, EPI_ISL_625809, EPI_ISL_625810, EPI_ISL_625811, EPI_ISL_625812, EPI_ISL_625813, EPI_ISL_625814, EPI_ISL_625815, EPI_ISL_625816, EPI_ISL_625817, EPI_ISL_625818, EPI_ISL_625819, EPI_ISL_625820, EPI_ISL_625821, EPI_ISL_625822, EPI_ISL_625823, EPI_ISL_625824, EPI_ISL_625825, EPI_ISL_625826, EPI_ISL_625827, EPI_ISL_625828, EPI_ISL_625829, EPI_ISL_625830, EPI_ISL_625831, EPI_ISL_625832, EPI_ISL_625833, EPI_ISL_625834, EPI_ISL_625835, EPI_ISL_625836, EPI_ISL_625837, EPI_ISL_625838, EPI_ISL_625839, EPI_ISL_625842, EPI_ISL_625844, EPI_ISL_625846, EPI_ISL_625847, EPI_ISL_625848, EPI_ISL_625857, EPI_ISL_625872, EPI_ISL_625873, EPI_ISL_625874, EPI_ISL_625875, EPI_ISL_625877, EPI_ISL_625878, EPI_ISL_625885, EPI_ISL_625889, EPI_ISL_625892, EPI_ISL_625893, EPI_ISL_625894, EPI_ISL_625895, EPI_ISL_625896, EPI_ISL_625897, EPI_ISL_625898, EPI_ISL_625899, EPI_ISL_625900, EPI_ISL_625901, EPI_ISL_625902, EPI_ISL_625903, EPI_ISL_625904, EPI_ISL_625905, EPI_ISL_625906, EPI_ISL_625907, EPI_ISL_625908, EPI_ISL_625909, EPI_ISL_625910, EPI_ISL_625911, EPI_ISL_625912, EPI_ISL_625913, EPI_ISL_625914, EPI_ISL_625915, EPI_ISL_625916, EPI_ISL_625917, EPI_ISL_625918, EPI_ISL_625919, EPI_ISL_625920, EPI_ISL_625921, EPI_ISL_625922, EPI_ISL_625923, EPI_ISL_625924, EPI_ISL_625925, EPI_ISL_625926, EPI_ISL_625927, EPI_ISL_625928, EPI_ISL_625929, EPI_ISL_625930, EPI_ISL_625931, EPI_ISL_625932, EPI_ISL_625934, EPI_ISL_625935, EPI_ISL_625936, EPI_ISL_625939, EPI_ISL_625940, EPI_ISL_625941, EPI_ISL_625942, EPI_ISL_625945, EPI_ISL_625946, EPI_ISL_625947, EPI_ISL_625948, EPI_ISL_625949, EPI_ISL_625950, EPI_ISL_625951, EPI_ISL_625953, EPI_ISL_625954, EPI_ISL_625956, EPI_ISL_625957, EPI_ISL_625958, EPI_ISL_625959, EPI_ISL_625961, EPI_ISL_625962, EPI_ISL_625963, EPI_ISL_625964, EPI_ISL_625965, EPI_ISL_625966, EPI_ISL_625969, EPI_ISL_625971, EPI_ISL_625976, EPI_ISL_625977, EPI_ISL_625978, EPI_ISL_625981, EPI_ISL_625982, EPI_ISL_625985, EPI_ISL_625986, EPI_ISL_625987, EPI_ISL_625988, EPI_ISL_625990, EPI_ISL_625991, EPI_ISL_625992, EPI_ISL_625993, EPI_ISL_625994, EPI_ISL_625995, EPI_ISL_625996, EPI_ISL_625997, EPI_ISL_625999, EPI_ISL_626000, EPI_ISL_626001, EPI_ISL_626002, EPI_ISL_626003, EPI_ISL_626004, EPI_ISL_626005, EPI_ISL_626006, EPI_ISL_626007, EPI_ISL_626008, EPI_ISL_626009, EPI_ISL_626011, EPI_ISL_626012, EPI_ISL_626013, EPI_ISL_626014, EPI_ISL_626015, EPI_ISL_626016, EPI_ISL_626017, EPI_ISL_626018, EPI_ISL_626019, EPI_ISL_626020, EPI_ISL_626021, EPI_ISL_626022, EPI_ISL_626023, EPI_ISL_626024, EPI_ISL_626025, EPI_ISL_626026, EPI_ISL_626027, EPI_ISL_626028, EPI_ISL_626029, EPI_ISL_626030, EPI_ISL_626031, EPI_ISL_626032, EPI_ISL_626033, EPI_ISL_626034, EPI_ISL_626035, EPI_ISL_626036, EPI_ISL_626037, EPI_ISL_626038, EPI_ISL_626039, EPI_ISL_626040, EPI_ISL_626041, EPI_ISL_626042, EPI_ISL_626043, EPI_ISL_626044, EPI_ISL_626045, EPI_ISL_626046, EPI_ISL_626047, EPI_ISL_626048, EPI_ISL_626049, EPI_ISL_626050, EPI_ISL_626051, EPI_ISL_626052, EPI_ISL_626053, EPI_ISL_626054, EPI_ISL_626055, EPI_ISL_626056, EPI_ISL_626057, EPI_ISL_626058, EPI_ISL_626059, EPI_ISL_626060, EPI_ISL_626061, EPI_ISL_626062, EPI_ISL_626063, EPI_ISL_626064, EPI_ISL_626065, EPI_ISL_626066, EPI_ISL_626067, EPI_ISL_626068, EPI_ISL_626069, EPI_ISL_626070, EPI_ISL_626071, EPI_ISL_626072, EPI_ISL_626073, EPI_ISL_626074, EPI_ISL_626075, EPI_ISL_626076, EPI_ISL_626077, EPI_ISL_626078, EPI_ISL_626079, EPI_ISL_626080, EPI_ISL_626081, EPI_ISL_626082, EPI_ISL_626083, EPI_ISL_626084, EPI_ISL_626085, EPI_ISL_626086, EPI_ISL_626087, EPI_ISL_626088, EPI_ISL_626089, EPI_ISL_626090, EPI_ISL_626091, EPI_ISL_626092, EPI_ISL_626093, EPI_ISL_626094, EPI_ISL_626095, EPI_ISL_626096, EPI_ISL_626097, EPI_ISL_626098, EPI_ISL_626099, EPI_ISL_626100, EPI_ISL_626101, EPI_ISL_626102, EPI_ISL_626103, EPI_ISL_626104, EPI_ISL_626105, EPI_ISL_626106, EPI_ISL_626107, EPI_ISL_626108, EPI_ISL_626109, EPI_ISL_626110, EPI_ISL_626111, EPI_ISL_626112, EPI_ISL_626113, EPI_ISL_626114, EPI_ISL_626115, EPI_ISL_626116, EPI_ISL_626117, EPI_ISL_626118, EPI_ISL_626119, EPI_ISL_626120, EPI_ISL_626121, EPI_ISL_626122, EPI_ISL_626123, EPI_ISL_626124, EPI_ISL_626125, EPI_ISL_626126, EPI_ISL_626127, EPI_ISL_626128, EPI_ISL_626129, EPI_ISL_626130, EPI_ISL_626131, EPI_ISL_626132, EPI_ISL_626133, EPI_ISL_626134, EPI_ISL_626135, EPI_ISL_626136, EPI_ISL_626137, EPI_ISL_626138, EPI_ISL_626139, EPI_ISL_626140, EPI_ISL_626141, EPI_ISL_626142, EPI_ISL_626143, EPI_ISL_626144, EPI_ISL_626145, EPI_ISL_626146, EPI_ISL_626147, EPI_ISL_626148, EPI_ISL_626149, EPI_ISL_626150, EPI_ISL_626151, EPI_ISL_626152, EPI_ISL_626153, EPI_ISL_626154, EPI_ISL_626155, EPI_ISL_626156, EPI_ISL_626157, EPI_ISL_626158, EPI_ISL_626159, EPI_ISL_626160, EPI_ISL_626161, EPI_ISL_626162, EPI_ISL_626163, EPI_ISL_626164, EPI_ISL_626165, EPI_ISL_626166, EPI_ISL_626167, EPI_ISL_626168, EPI_ISL_626169, EPI_ISL_626170, EPI_ISL_626171, EPI_ISL_626172, EPI_ISL_626173, EPI_ISL_626174, EPI_ISL_626175, EPI_ISL_626176, EPI_ISL_626177, EPI_ISL_626178, EPI_ISL_626179, EPI_ISL_626180, EPI_ISL_626181, EPI_ISL_626182, EPI_ISL_626183, EPI_ISL_626184, EPI_ISL_626185, EPI_ISL_626186, EPI_ISL_626187, EPI_ISL_626188, EPI_ISL_626189, EPI_ISL_626190, EPI_ISL_626191, EPI_ISL_626192, EPI_ISL_626193, EPI_ISL_626194, EPI_ISL_626195, EPI_ISL_626196, EPI_ISL_626197, EPI_ISL_626198, EPI_ISL_626199, EPI_ISL_626200, EPI_ISL_626201, EPI_ISL_626202, EPI_ISL_626203, EPI_ISL_626204, EPI_ISL_626205, EPI_ISL_626206, EPI_ISL_626207, EPI_ISL_626208, EPI_ISL_626209, EPI_ISL_626210			
see above	Department of Virus and Microbiological Special Diagnostics, Statens Serum Institut, Denmark	Albertsen lab, Department of Chemistry and Bioscience, Aalborg University, Denmark	Danish Covid-19 Genome Consortia
EPI_ISL_626211, EPI_ISL_626212, EPI_ISL_626214, EPI_ISL_626216, EPI_ISL_626217, EPI_ISL_626219, EPI_ISL_626220, EPI_ISL_626221, EPI_ISL_626222, EPI_ISL_626223, EPI_ISL_626230			
see above	Institute for Virology, University Hospital Essen	Center of Medical Microbiology, Virology, and Hospital Hygiene, University of Duesseldorf	Olympia E. Anastasiou, Ulf Dittmer, Maximilian Damagnez, Alexander Dilthey, Torsten Houwaart, Lisanna Hülse, Malte Kohns Vasconcelos, Nadine Lübke, Jessica Nicolai, Klaus Pfeffer, Daniel Strelow, Jörg Timm, Andreas Walker, Tobias Wienemann
EPI_ISL_626231, EPI_ISL_626232, EPI_ISL_626233, EPI_ISL_626234, EPI_ISL_626235, EPI_ISL_626236, EPI_ISL_626237, EPI_ISL_626238, EPI_ISL_626239, EPI_ISL_626240, EPI_ISL_626241, EPI_ISL_626242, EPI_ISL_626243, EPI_ISL_626244, EPI_ISL_626245, EPI_ISL_626246, EPI_ISL_626247, EPI_ISL_626248, EPI_ISL_626249, EPI_ISL_626250, EPI_ISL_626251, EPI_ISL_626252, EPI_ISL_626253, EPI_ISL_626254, EPI_ISL_626255, EPI_ISL_626256, EPI_ISL_626257, EPI_ISL_626258, EPI_ISL_626259, EPI_ISL_626260, EPI_ISL_626261, EPI_ISL_626262, EPI_ISL_626263, EPI_ISL_626265, EPI_ISL_626266, EPI_ISL_626267, EPI_ISL_626268, EPI_ISL_626269, EPI_ISL_626270, EPI_ISL_626271, EPI_ISL_626272, EPI_ISL_626273, EPI_ISL_626274, EPI_ISL_626275, EPI_ISL_626276, EPI_ISL_626277, EPI_ISL_626278, EPI_ISL_626279, EPI_ISL_626280, EPI_ISL_626281, EPI_ISL_626282, EPI_ISL_626283, EPI_ISL_626284, EPI_ISL_626285, EPI_ISL_626286, EPI_ISL_626287, EPI_ISL_626288, EPI_ISL_626289, EPI_ISL_626290, EPI_ISL_626291, EPI_ISL_626293, EPI_ISL_626294, EPI_ISL_626295, EPI_ISL_626296, EPI_ISL_626297, EPI_ISL_626299, EPI_ISL_626300, EPI_ISL_626301, EPI_ISL_626302, EPI_ISL_626303, EPI_ISL_626304, EPI_ISL_626305, EPI_ISL_626306, EPI_ISL_626307, EPI_ISL_626310, EPI_ISL_626311, EPI_ISL_626312, EPI_ISL_626314, EPI_ISL_626315, EPI_ISL_626316, EPI_ISL_626318, EPI_ISL_626319, EPI_ISL_626320, EPI_ISL_626321, EPI_ISL_626322, EPI_ISL_626323, EPI_ISL_626324, EPI_ISL_626325, EPI_ISL_626326, EPI_ISL_626327, EPI_ISL_626328, EPI_ISL_626330, EPI_ISL_626331, EPI_ISL_626332, EPI_ISL_626333, EPI_ISL_626334, EPI_ISL_626335, EPI_ISL_626336, EPI_ISL_626337, EPI_ISL_626338, EPI_ISL_626339			
see above	Department of Clinical Microbiology	GIGA Medical Genomics	Keith Durkin, Maria Artesi, Sébastien Bontems, Raphaël Boreux, Bouchra Boujemla, Cécile Meex, Pierrette Melin, Marie-Pierre Hayette, Vincent Bours
EPI_ISL_632284, EPI_ISL_632285	Communicable Disease Laboratory, Public Health Directorate	Communicable Disease Laboratory, Public Health Directorate	AlWasti,H., AlTaif,Z., AlHujairi,Z., AlAbbas,Z.
EPI_ISL_634976	Institute of Molecular Biology and Biotechnology, National Institutes of Health	Institute of Molecular Biology and Biotechnology, National Institutes of Health	Schwem,B., Arevalo,G., Dungca,N.T. and Salvana,E.M.
