# Supplementary material for: Global Geographic and Temporal Analysis of SARS-CoV-2 Haplotypes Normalized by COVID-19 Cases During the Pandemic
Source: Front Microbiol. 2021 Feb 17;12:612432. doi: 10.3389/fmicb.2021.612432 (PMC7971176; doi:10.3389/fmicb.2021.612432)
Supplement: Supplementary file 2 [file Data_Sheet_2.zip › 17_11-09_to_11-15.pdf]

We gratefully acknowledge the following Authors from the Originating laboratories responsible for obtaining the specimens, as well as the Submitting laboratories where the genome data were generated and shared via GISAID, on which this research is based.

All Submitters of data may be contacted directly via www.gisaid.org

Accession ID	Originating Laboratory	Submitting Laboratory	Authors
EPI_ISL_626352, EPI_ISL_626353, EPI_ISL_626354, EPI_ISL_626355, EPI_ISL_626356	LabPLUS	Institute of Environmental Science and Research (ESR)	Xiaoyun Ren, Matt Storey, Nikki Freed, Muhammad Faisal, Jing Wang, Hermes Perez, Anja Werno, Antje van der Linden, Arlo Upton, Chris Mansell, David Hammer, Dragana Drinkovic, Gary McAuliffe, Hana Sofia Andersson, James Ussher, Jill Sherwood, Josh Freeman, Julia Howard, Juliet Elvy, Mary DeAlmeida, Matt Blakiston, Matthew Rogers, Max Bloomfield, Michael Addidle, Michelle Balm, Sally Roberts, Sarah Jefferies, Sharmini Muttaiyah, Susan Morpeth, Susan Taylor, Timothy Blackmore, Vani Sathyendran, Veronica Playle, Virginia Hope, Erasmus Smit, Lauren Jelly, Olin Silander, Joep de Ligt
EPI_ISL_626359, EPI_ISL_626360, EPI_ISL_626364, EPI_ISL_626365, EPI_ISL_626366, EPI_ISL_626368, EPI_ISL_626370, EPI_ISL_626371, EPI_ISL_626372, EPI_ISL_626373, EPI_ISL_626374, EPI_ISL_626375, EPI_ISL_626376, EPI_ISL_626377, EPI_ISL_626378, EPI_ISL_626379, EPI_ISL_626381, EPI_ISL_626382, EPI_ISL_626383, EPI_ISL_626384, EPI_ISL_626385, EPI_ISL_626386, EPI_ISL_626387, EPI_ISL_626388, EPI_ISL_626389, EPI_ISL_626390, EPI_ISL_626391, EPI_ISL_626392, EPI_ISL_626393, EPI_ISL_626394, EPI_ISL_626395, EPI_ISL_626396, EPI_ISL_626397, EPI_ISL_626398, EPI_ISL_626399, EPI_ISL_626400, EPI_ISL_626401, EPI_ISL_626402, EPI_ISL_626403, EPI_ISL_626404, EPI_ISL_626405, EPI_ISL_626406, EPI_ISL_626407, EPI_ISL_626408, EPI_ISL_626409, EPI_ISL_626410, EPI_ISL_626415, EPI_ISL_626431, EPI_ISL_626438, EPI_ISL_626439, EPI_ISL_626442, EPI_ISL_626443, EPI_ISL_626445, EPI_ISL_626446, EPI_ISL_626447, EPI_ISL_626448, EPI_ISL_626449, EPI_ISL_626450, EPI_ISL_626451, EPI_ISL_626452, EPI_ISL_626453, EPI_ISL_626457, EPI_ISL_626458, EPI_ISL_626459, EPI_ISL_626460, EPI_ISL_626461, EPI_ISL_626464, EPI_ISL_626465, EPI_ISL_626466, EPI_ISL_626467, EPI_ISL_626468, EPI_ISL_626469, EPI_ISL_626470, EPI_ISL_626472, EPI_ISL_626473, EPI_ISL_626476, EPI_ISL_626478, EPI_ISL_626480, EPI_ISL_626485, EPI_ISL_626486, EPI_ISL_626487, EPI_ISL_626488, EPI_ISL_626489, EPI_ISL_626492, EPI_ISL_626493, EPI_ISL_626494, EPI_ISL_626495, EPI_ISL_626497, EPI_ISL_626498, EPI_ISL_626502, EPI_ISL_626503, EPI_ISL_626505, EPI_ISL_626506, EPI_ISL_626507, EPI_ISL_626508, EPI_ISL_626509, EPI_ISL_626511, EPI_ISL_626514, EPI_ISL_626515, EPI_ISL_626521, EPI_ISL_626522, EPI_ISL_626524, EPI_ISL_626525, EPI_ISL_626526, EPI_ISL_626527, EPI_ISL_626529, EPI_ISL_626532, EPI_ISL_626536, EPI_ISL_626538, EPI_ISL_626539, EPI_ISL_626540, EPI_ISL_626542			
see above	Northwestern Memorial Hospital	Ozer Lab	Ramon Lorenzo-Redondo, Hannah H. Nam, Scott C. Roberts, Lacy M. Simons, Chad J. Achenbach, Lawrence J. Jennings, Chao Qi, Alan R. Hauser, Michael G. Ison, Judd F. Hultquist, Egon A. Ozer
EPI_ISL_626549, EPI_ISL_626550, EPI_ISL_626551, EPI_ISL_626552, EPI_ISL_626553, EPI_ISL_626554, EPI_ISL_626555, EPI_ISL_626556, EPI_ISL_626557, EPI_ISL_626558, EPI_ISL_626559, EPI_ISL_626560, EPI_ISL_626561, EPI_ISL_626562, EPI_ISL_626563, EPI_ISL_626564, EPI_ISL_626565			
see above	Laboratorio de Biología Molecular, Facultad de Medicina, Universidad de Atacama, Copiapo, Chile/ FONDAP CRG, Universidad Andrés Bello, Santiago, Chile	Center for Mathematical Modeling and Center for Genome Regulation. Santiago, Chile	Echeverría C, Manríquez R, Bastias M, Sanhueza D, Travisany D, Allende ML, Maass A, González M, Montecino, M, Orellana A, Castro E, Meneses C.
EPI_ISL_626570, EPI_ISL_626571, EPI_ISL_626572, EPI_ISL_626573, EPI_ISL_626574, EPI_ISL_626575, EPI_ISL_626576, EPI_ISL_626577, EPI_ISL_626578, EPI_ISL_626579, EPI_ISL_626580, EPI_ISL_626581, EPI_ISL_626582, EPI_ISL_626583, EPI_ISL_626584, EPI_ISL_626585, EPI_ISL_626586, EPI_ISL_626587, EPI_ISL_626588, EPI_ISL_626589, EPI_ISL_626590, EPI_ISL_626591, EPI_ISL_626592, EPI_ISL_626593, EPI_ISL_626594, EPI_ISL_626595, EPI_ISL_626596, EPI_ISL_626597, EPI_ISL_626598, EPI_ISL_626599, EPI_ISL_626600, EPI_ISL_626601, EPI_ISL_626602, EPI_ISL_626603, EPI_ISL_626604, EPI_ISL_626605, EPI_ISL_626606, EPI_ISL_626607, EPI_ISL_626608, EPI_ISL_626609, EPI_ISL_626610, EPI_ISL_626611, EPI_ISL_626612, EPI_ISL_626613, EPI_ISL_626614, EPI_ISL_626615, EPI_ISL_626616, EPI_ISL_626617, EPI_ISL_626618, EPI_ISL_626619, EPI_ISL_626620, EPI_ISL_626621, EPI_ISL_626622, EPI_ISL_626623, EPI_ISL_626624, EPI_ISL_626625			
see above	The National Institute of Public Health	State Veterinary Institute Prague	Nagy,A;Jirincova,H;Novakova,L;Trnka,D;Vecerova,J
EPI_ISL_626628, EPI_ISL_626629, EPI_ISL_626630, EPI_ISL_626631, EPI_ISL_626632, EPI_ISL_626633, EPI_ISL_626634, EPI_ISL_626635, EPI_ISL_626639, EPI_ISL_626640, EPI_ISL_626641, EPI_ISL_626642, EPI_ISL_626643			
see above	National Public Health Laboratory, National Centre for Infectious Diseases	National Public Health Laboratory, National Centre for Infectious Diseases	Tze Minn Mak, Sophie Octavia, Zhenyang Zhou, Lin Cui, Raymond Tzer Pin Lin
EPI_ISL_627070	Regional Virus Laboratory, Belfast Health and Social Care Trust	COVID-19 Genomics UK (COG-UK) Consortium	Conall McCaughey, James McKenna, Tanya Curran, Susan Feeney, Alison Watt, Ciara Cox, Mairead Connor, Zoltan Molnar, David Simpson, Derek Fairley
EPI_ISL_627071, EPI_ISL_627072, EPI_ISL_627073, EPI_ISL_627074, EPI_ISL_627075, EPI_ISL_627076, EPI_ISL_627077	Quadram Institute Bioscience	COVID-19 Genomics UK (COG-UK) Consortium	Dave J. Baker, Gemma L. Kay, Alp Aydin, Thanh Le-Viet, Steven Rudder, Ana P. Tedim, Anastasia Kolyva, Maria Diaz, Leonardo de Oliveira Martins, Nabil-Fareed Alikhan, Lizzie Meadows, Rachael Stanley, Ngozi Elumogo, Muhammed Yasir, Nicholas M. Thomson, Alexander J Trotter, Rachel Gilroy, Samuel Bloomfield, Claire Stuart, Andrew Bell, Reenesh Prakash, Samir Dervisevic, Alison E. Mather, John Wain, Mark Webber, Andrew J. Page, Justin O'Grady
EPI_ISL_627078	Wales Specialist Virology Centre Sequencing lab: Pathogen Genomics Unit	COVID-19 Genomics UK (COG-UK) Consortium	Catherine Moore, Johnathan Evans, Laura Gifford, Malorie Perry, Simon Cottrell, Angela Marchbank, Alec Birchley, Alexander Adams, Amy Gaskin, Bree Gatica-Wilcox, Jason Coombes, Joel Southgate, Lauren Gilbert, Lee Graham, Nicole Pacchiarini, Sara Kumziene-Summerhayes, Sarah Taylor, Sophie Jones, Sara Rey, Matthew Bull, Joanne Watkins, Sally Corden, Tom Connor
EPI_ISL_627079	Oxford Viromics, NDM, University of Oxford; Oxford University Hospitals; Basingstoke and North Hampshire Hospital	COVID-19 Genomics UK (COG-UK) Consortium	Tanya Golubchik, David Bonsall, George Macintyre, Amy Trebes, Mariateresa de Cesare, Catrin Moore, Alex Mobbs, Anita Justice, Robert Shaw, Monique Andersson, Timothy Peto, Emma Wise, Nathan Moore, Jessica Lynch, Nick Cortes, Matilde Mori, Stephen Kidd, David Buck, John Todd, Christophe Fraser
EPI_ISL_627080, EPI_ISL_627081	Quadram Institute Bioscience	COVID-19 Genomics UK (COG-UK) Consortium	Dave J. Baker, Gemma L. Kay, Alp Aydin, Thanh Le-Viet, Steven Rudder, Ana P. Tedim, Anastasia Kolyva, Maria Diaz, Leonardo de Oliveira Martins, Nabil-Fareed Alikhan, Lizzie Meadows, Rachael Stanley, Ngozi Elumogo, Muhammed Yasir, Nicholas M. Thomson, Alexander J Trotter, Rachel Gilroy, Samuel Bloomfield, Claire Stuart, Andrew Bell, Reenesh Prakash, Samir Dervisevic, Alison E. Mather, John Wain, Mark Webber, Andrew J. Page, Justin O'Grady
EPI_ISL_627082	University College London, Great Ormond Street Hospital for Children NHS Foundation Trust, Imperial College Healthcare NHS Trust	COVID-19 Genomics UK (COG-UK) Consortium	Sergi Castellano, Rachel Williams, Mark Kristiansen, Paola Resende Silva, Sunando Roy, Tony Brooks, Helena Tutill, Paola Niola, Patricia Dyal, Charlotte Williams, Leysa Forrest, Yasmin Panchbhaya, Jacqueline Findlay, Samuel Weeks, Julianne Brown, Kathryn Harris, Paul Randell, James Price, Alison Holmes, Judith Breuer
EPI_ISL_627083, EPI_ISL_627084	Quadram Institute Bioscience	COVID-19 Genomics UK (COG-UK) Consortium	Dave J. Baker, Gemma L. Kay, Alp Aydin, Thanh Le-Viet, Steven Rudder, Ana P. Tedim, Anastasia Kolyva, Maria Diaz, Leonardo de Oliveira Martins, Nabil-Fareed Alikhan, Lizzie Meadows, Rachael Stanley, Ngozi Elumogo, Muhammed Yasir, Nicholas M. Thomson, Alexander J Trotter, Rachel Gilroy, Samuel Bloomfield, Claire Stuart, Andrew Bell, Reenesh Prakash, Samir Dervisevic, Alison E. Mather, John Wain, Mark Webber, Andrew J. Page, Justin O'Grady
EPI_ISL_627085	Oxford Viromics, NDM, University of Oxford; Oxford University Hospitals; Basingstoke and North Hampshire Hospital	COVID-19 Genomics UK (COG-UK) Consortium	Tanya Golubchik, David Bonsall, George Macintyre, Amy Trebes, Mariateresa de Cesare, Catrin Moore, Alex Mobbs, Anita Justice, Robert Shaw, Monique Andersson, Timothy Peto, Emma Wise, Nathan Moore, Jessica Lynch, Nick Cortes, Matilde Mori, Stephen Kidd, David Buck, John Todd, Christophe Fraser
EPI_ISL_627086, EPI_ISL_627087, EPI_ISL_627088, EPI_ISL_627089, EPI_ISL_627090, EPI_ISL_627091, EPI_ISL_627092, EPI_ISL_627093, EPI_ISL_627094, EPI_ISL_627095, EPI_ISL_627096, EPI_ISL_627097, EPI_ISL_627203, EPI_ISL_627204, EPI_ISL_627205, EPI_ISL_627206, EPI_ISL_627207			
see above	Wales Specialist Virology Centre Sequencing lab: Pathogen Genomics Unit	COVID-19 Genomics UK (COG-UK) Consortium	Catherine Moore, Johnathan Evans, Laura Gifford, Malorie Perry, Simon Cottrell, Angela Marchbank, Alec Birchley, Alexander Adams, Amy Gaskin, Bree Gatica-Wilcox, Jason Coombes, Joel Southgate, Lauren Gilbert, Lee Graham, Nicole Pacchiarini, Sara Kumziene-Summerhayes, Sarah Taylor, Sophie Jones, Sara Rey, Matthew Bull, Joanne Watkins, Sally Corden, Tom Connor
EPI_ISL_627208, EPI_ISL_627209	West of Scotland Specialist Virology Centre, NHSGGC / MRC-University of Glasgow Centre for Virus Research	COVID-19 Genomics UK (COG-UK) Consortium	Ana da Silva Filipe, Natasha Johnson, Kathy Smollett, Daniel Mair, Stephen Carmichael, Lily Tong, Jenna Nichols, Elihu Aranday-Cortes, Kyriaki Nomikou; Sarah McDonald, Marc Niebel, Patawee Asamaphan; Richard Orton, Joseph Hughes, Sreenu Vattipally, David L Robertson; Alasdair MacLean, Rory Gunson; Kathy Li, Igor Starinskij, Natasha Jesudason, Rajiv Shah, James Shepherd, Antonia Ho, Emma Thomson
EPI_ISL_627210	University of Exeter	COVID-19 Genomics UK (COG-UK) Consortium	Ben Temperton,Aaron Jeffries,Michelle Michelsen,Joanna Warwick-Dugdale,Audrey Farbos,Robyn Manley,Stephen Michell,Jane Masoli
EPI_ISL_627211	University College London, Great Ormond Street Hospital for Children NHS Foundation Trust, Imperial	COVID-19 Genomics UK (COG-UK) Consortium	Sergi Castellano, Rachel Williams, Mark Kristiansen, Paola Resende Silva, Sunando Roy, Tony Brooks, Helena Tutill, Paola Niola, Patricia Dyal, Charlotte Williams, Leysa Forrest, Yasmin Panchbhaya, Jacqueline Findlay, Samuel Weeks, Julianne Brown, Kathryn Harris, Paul Randell, James Price, Alison

	College Healthcare NHS Trust		Holmes, Judith Breuer
EPI_ISL_627212	Wales Specialist Virology Centre Sequencing lab: Pathogen Genomics Unit	COVID-19 Genomics UK (COG-UK) Consortium	Catherine Moore, Johnathan Evans, Laura Gifford, Malorie Perry, Simon Cottrell, Angela Marchbank, Alec Birchley, Alexander Adams, Amy Gaskin, Bree Gatica-Wilcox, Jason Coombes, Joel Southgate, Lauren Gilbert, Lee Graham, Nicole Pacchiarini, Sara Kumziene-Summerhayes, Sarah Taylor, Sophie Jones, Sara Rey, Matthew Bull, Joanne Watkins, Sally Corden, Tom Connor
EPI_ISL_627213, EPI_ISL_627214	Quadram Institute Bioscience	COVID-19 Genomics UK (COG-UK) Consortium	Dave J. Baker, Gemma L. Kay, Alp Aydin, Thanh Le-Viet, Steven Rudder, Ana P. Tedim, Anastasia Kolyva, Maria Diaz, Leonardo de Oliveira Martins, Nabil-Fareed Alikhan, Lizzie Meadows, Rachael Stanley, Ngozi Elumogo, Muhammed Yasir, Nicholas M. Thomson, Alexander J Trotter, Rachel Gilroy, Samuel Bloomfield, Claire Stuart, Andrew Bell, Reenesh Prakash, Samir Dervisevic, Alison E. Mather, John Wain, Mark Webber, Andrew J. Page, Justin O'Grady
EPI_ISL_627215, EPI_ISL_627216	Queens Medical Centre, Clinical Microbiology Department / DeepSeq Nottingham	COVID-19 Genomics UK (COG-UK) Consortium	Gemma Clark, Wendy Smith, Manjinder Khakh, Vicki M Fleming, Michelle M Lister, Hannah Howson-Wells, Jonathan Ball, Patrick McClure, Joseph Chappell, Theocharis Tsoleridis, Nadine Holmes, Matthew Carlisle, Christopher Moore, Fei Sang, Johnny Debebe, Victoria Wright, Matthew Loose
EPI_ISL_627217, EPI_ISL_627218, EPI_ISL_627219, EPI_ISL_627220, EPI_ISL_627221, EPI_ISL_627222, EPI_ISL_627223, EPI_ISL_627224, EPI_ISL_627225, EPI_ISL_627226, EPI_ISL_627227, EPI_ISL_627228, EPI_ISL_627229, EPI_ISL_627230, EPI_ISL_627231, EPI_ISL_627232, EPI_ISL_627233, EPI_ISL_627234, EPI_ISL_627235, EPI_ISL_627236, EPI_ISL_627237, EPI_ISL_627238, EPI_ISL_627239, EPI_ISL_627240, EPI_ISL_627241, EPI_ISL_627242, EPI_ISL_627243, EPI_ISL_627244, EPI_ISL_627245, EPI_ISL_627246, EPI_ISL_627247, EPI_ISL_627248, EPI_ISL_627249			
see above	Wales Specialist Virology Centre Sequencing lab: Pathogen Genomics Unit	COVID-19 Genomics UK (COG-UK) Consortium	Catherine Moore, Johnathan Evans, Laura Gifford, Malorie Perry, Simon Cottrell, Angela Marchbank, Alec Birchley, Alexander Adams, Amy Gaskin, Bree Gatica-Wilcox, Jason Coombes, Joel Southgate, Lauren Gilbert, Lee Graham, Nicole Pacchiarini, Sara Kumziene-Summerhayes, Sarah Taylor, Sophie Jones, Sara Rey, Matthew Bull, Joanne Watkins, Sally Corden, Tom Connor
EPI_ISL_627250, EPI_ISL_627251	Oxford Viromics, NDM, University of Oxford; Oxford University Hospitals; Basingstoke and North Hampshire Hospital	COVID-19 Genomics UK (COG-UK) Consortium	Tanya Golubchik, David Bonsall, George Macintyre, Amy Trebes, Mariateresa de Cesare, Catrin Moore, Alex Mobbs, Anita Justice, Robert Shaw, Monique Andersson, Timothy Peto, Emma Wise, Nathan Moore, Jessica Lynch, Nick Cortes, Matilde Mori, Stephen Kidd, David Buck, John Todd, Christophe Fraser
EPI_ISL_627252, EPI_ISL_627253, EPI_ISL_627254	Virology Department, Sheffield Teaching Hospitals NHS Foundation Trust/Department of Infection, Immunity and Cardiovascular Disease, The Medical School, University of Sheffield	COVID-19 Genomics UK (COG-UK) Consortium	Thushan de Silva, Matthew Parker, Nikki Smith, Adri Angyal, Rebecca Brown, Luke Green, Rachel Tucker, Paul Parsons, Danielle Groves, Katie Johnson, Laura Carrilero, Alex Keeley, Dave Partridge, Matthew Wyles, Benjamin Lindsey, Mehmet Yavuz, Mohammad Raza, Cariad Evans
EPI_ISL_627255, EPI_ISL_627256, EPI_ISL_627257, EPI_ISL_627258, EPI_ISL_627259, EPI_ISL_627260, EPI_ISL_627261, EPI_ISL_627262, EPI_ISL_627292	Oxford Viromics, NDM, University of Oxford; Oxford University Hospitals; Basingstoke and North Hampshire Hospital	COVID-19 Genomics UK (COG-UK) Consortium	Tanya Golubchik, David Bonsall, George Macintyre, Amy Trebes, Mariateresa de Cesare, Catrin Moore, Alex Mobbs, Anita Justice, Robert Shaw, Monique Andersson, Timothy Peto, Emma Wise, Nathan Moore, Jessica Lynch, Nick Cortes, Matilde Mori, Stephen Kidd, David Buck, John Todd, Christophe Fraser
EPI_ISL_627295, EPI_ISL_627296	Wales Specialist Virology Centre Sequencing lab: Pathogen Genomics Unit	COVID-19 Genomics UK (COG-UK) Consortium	Catherine Moore, Johnathan Evans, Laura Gifford, Malorie Perry, Simon Cottrell, Angela Marchbank, Alec Birchley, Alexander Adams, Amy Gaskin, Bree Gatica-Wilcox, Jason Coombes, Joel Southgate, Lauren Gilbert, Lee Graham, Nicole Pacchiarini, Sara Kumziene-Summerhayes, Sarah Taylor, Sophie Jones, Sara Rey, Matthew Bull, Joanne Watkins, Sally Corden, Tom Connor
EPI_ISL_627300	Quadram Institute Bioscience	COVID-19 Genomics UK (COG-UK) Consortium	Dave J. Baker, Gemma L. Kay, Alp Aydin, Thanh Le-Viet, Steven Rudder, Ana P. Tedim, Anastasia Kolyva, Maria Diaz, Leonardo de Oliveira Martins, Nabil-Fareed Alikhan, Lizzie Meadows, Rachael Stanley, Ngozi Elumogo, Muhammed Yasir, Nicholas M. Thomson, Alexander J Trotter, Rachel Gilroy, Samuel Bloomfield, Claire Stuart, Andrew Bell, Reenesh Prakash, Samir Dervisevic, Alison E. Mather, John Wain, Mark Webber, Andrew J. Page, Justin O'Grady
EPI_ISL_627301	Wales Specialist Virology Centre Sequencing lab: Pathogen Genomics Unit	COVID-19 Genomics UK (COG-UK) Consortium	Catherine Moore, Johnathan Evans, Laura Gifford, Malorie Perry, Simon Cottrell, Angela Marchbank, Alec Birchley, Alexander Adams, Amy Gaskin, Bree Gatica-Wilcox, Jason Coombes, Joel Southgate, Lauren Gilbert, Lee Graham, Nicole Pacchiarini, Sara Kumziene-Summerhayes, Sarah Taylor, Sophie Jones, Sara Rey, Matthew Bull, Joanne Watkins, Sally Corden, Tom Connor
EPI_ISL_627309, EPI_ISL_627310	Queens Medical Centre, Clinical Microbiology Department / DeepSeq Nottingham	COVID-19 Genomics UK (COG-UK) Consortium	Gemma Clark, Wendy Smith, Manjinder Khakh, Vicki M Fleming, Michelle M Lister, Hannah Howson-Wells, Jonathan Ball, Patrick McClure, Joseph Chappell, Theocharis Tsoleridis, Nadine Holmes, Matthew Carlisle, Christopher Moore, Fei Sang, Johnny Debebe, Victoria Wright, Matthew Loose
EPI_ISL_627314, EPI_ISL_627315, EPI_ISL_627316, EPI_ISL_627317, EPI_ISL_627318, EPI_ISL_627319, EPI_ISL_627320, EPI_ISL_627321, EPI_ISL_627322, EPI_ISL_627323, EPI_ISL_627324, EPI_ISL_627325, EPI_ISL_627326, EPI_ISL_627327, EPI_ISL_627328			
see above	University of Exeter	COVID-19 Genomics UK (COG-UK) Consortium	Ben Temperton,Aaron Jeffries,Michelle Michelsen,Joanna Warwick-Dugdale,Audrey Farbos,Robyn Manley,Stephen Michell,Jane Masoli
EPI_ISL_627329, EPI_ISL_627330, EPI_ISL_627331, EPI_ISL_627332, EPI_ISL_627333, EPI_ISL_627334, EPI_ISL_627335, EPI_ISL_627336, EPI_ISL_627337, EPI_ISL_627338, EPI_ISL_627339, EPI_ISL_627340, EPI_ISL_627341, EPI_ISL_627342, EPI_ISL_627343, EPI_ISL_627344, EPI_ISL_627345, EPI_ISL_627346, EPI_ISL_627347, EPI_ISL_627348, EPI_ISL_627349, EPI_ISL_627350, EPI_ISL_627351, EPI_ISL_627352, EPI_ISL_627353, EPI_ISL_627354, EPI_ISL_627355, EPI_ISL_627356, EPI_ISL_627357, EPI_ISL_627358, EPI_ISL_627359, EPI_ISL_627360, EPI_ISL_627361, EPI_ISL_627362, EPI_ISL_627363, EPI_ISL_627364, EPI_ISL_627365, EPI_ISL_627366, EPI_ISL_627367, EPI_ISL_627368, EPI_ISL_627369, EPI_ISL_627370, EPI_ISL_627371, EPI_ISL_627372, EPI_ISL_627373, EPI_ISL_627374, EPI_ISL_627375, EPI_ISL_627376, EPI_ISL_627377, EPI_ISL_627378, EPI_ISL_627379, EPI_ISL_627380, EPI_ISL_627381, EPI_ISL_627382, EPI_ISL_627383, EPI_ISL_627384, EPI_ISL_627385, EPI_ISL_627386, EPI_ISL_627387, EPI_ISL_627388, EPI_ISL_627389, EPI_ISL_627390, EPI_ISL_627391, EPI_ISL_627392, EPI_ISL_627393, EPI_ISL_627394, EPI_ISL_627395, EPI_ISL_627396, EPI_ISL_627397, EPI_ISL_627398, EPI_ISL_627399, EPI_ISL_627400, EPI_ISL_627401, EPI_ISL_627402, EPI_ISL_627403, EPI_ISL_627404, EPI_ISL_627405, EPI_ISL_627406, EPI_ISL_627407, EPI_ISL_627408, EPI_ISL_627409, EPI_ISL_627410, EPI_ISL_627411, EPI_ISL_627412, EPI_ISL_627413, EPI_ISL_627414, EPI_ISL_627415, EPI_ISL_627416, EPI_ISL_627417, EPI_ISL_627418, EPI_ISL_627419, EPI_ISL_627420, EPI_ISL_627421, EPI_ISL_627422, EPI_ISL_627423			
see above	West of Scotland Specialist Virology Centre, NHSGGC / MRC-University of Glasgow Centre for Virus Research	COVID-19 Genomics UK (COG-UK) Consortium	Ana da Silva Filipe, Natasha Johnson, Kathy Smollett, Daniel Mair, Stephen Carmichael, Lily Tong, Jenna Nichols, Elihu Aranday-Cortes, Kyriaki Nomikou; Sarah McDonald, Marc Niebel, Patawee Asamaphan; Richard Orton, Joseph Hughes, Sreenu Vattipally, David L Robertson; Alasdair MacLean, Rory Gunson; Kathy Li, Igor Starinskij, Natasha Jesudason, Rajiv Shah, James Shepherd, Antonia Ho, Emma Thomson
EPI_ISL_627424, EPI_ISL_627425, EPI_ISL_627426, EPI_ISL_627427, EPI_ISL_627428, EPI_ISL_627429, EPI_ISL_627430, EPI_ISL_627431, EPI_ISL_627432, EPI_ISL_627433, EPI_ISL_627434, EPI_ISL_627435, EPI_ISL_627436, EPI_ISL_627437, EPI_ISL_627438, EPI_ISL_627439, EPI_ISL_627440, EPI_ISL_627441, EPI_ISL_627442, EPI_ISL_627443, EPI_ISL_627444			
see above	University of Exeter	COVID-19 Genomics UK (COG-UK) Consortium	Ben Temperton,Aaron Jeffries,Michelle Michelsen,Joanna Warwick-Dugdale,Audrey Farbos,Robyn Manley,Stephen Michell,Jane Masoli
EPI_ISL_627445, EPI_ISL_627446, EPI_ISL_627447, EPI_ISL_627448, EPI_ISL_627449	Liverpool Clinical Laboratories	COVID-19 Genomics UK (COG-UK) Consortium	Sam Haldenby, Anita Lucaci, Steve Paterson, Julian Hiscox, Alistair Darby, M Almsaud, A Alrezaihi, Muhannad Alruwaili, Stuart D Armstrong, Jones Benjamin, Eleanor G Bentley, Anu Chawla, Jordan J Clark, Angela Cowell, Richard Eccles, Isabel García-Dorival, Matthew Gemmell, Alessandro Gerada, PKF Gilmore, Richard Gregory, Ximeng Han, Catherine Hartley, Margaret Hughes, Miren Iturriza-Gomara, James Johnson, L Luu, Jenifer Manson, Charlotte Nelson, Elaine O'Toole, Cassie Olateju, Rebekah Penrice-Randal , Lucille Rainbow, N.P Randle, Trevor Ian Robinson, Parul Sharma, Ghada T Shawli, James P Stewart, Neil Swainston, Ecaterina Vamos, Joanne Watts, Mark Whitehead
EPI_ISL_627450, EPI_ISL_627451, EPI_ISL_627452, EPI_ISL_627453, EPI_ISL_627454, EPI_ISL_627455, EPI_ISL_627456, EPI_ISL_627457, EPI_ISL_627458, EPI_ISL_627459, EPI_ISL_627460, EPI_ISL_627461, EPI_ISL_627462, EPI_ISL_627463, EPI_ISL_627464, EPI_ISL_627465, EPI_ISL_627466, EPI_ISL_627467, EPI_ISL_627468, EPI_ISL_627469, EPI_ISL_627470, EPI_ISL_627471, EPI_ISL_627472, EPI_ISL_627473, EPI_ISL_627474, EPI_ISL_627475, EPI_ISL_627476, EPI_ISL_627477, EPI_ISL_627478, EPI_ISL_627479, EPI_ISL_627480, EPI_ISL_627481			
see above	University College London, Great Ormond Street Hospital for Children NHS Foundation Trust, Imperial College Healthcare NHS Trust	COVID-19 Genomics UK (COG-UK) Consortium	Sergi Castellano, Rachel Williams, Mark Kristiansen, Paola Resende Silva, Sunando Roy, Tony Brooks, Helena Tutill, Paola Niola, Patricia Dyal, Charlotte Williams, Leysa Forrest, Yasmin Panchbhaya, Jacqueline Findlay, Samuel Weeks, Julianne Brown, Kathryn Harris, Paul Randell, James Price, Alison Holmes, Judith Breuer
EPI_ISL_627482, EPI_ISL_627483, EPI_ISL_627484, EPI_ISL_627485, EPI_ISL_627486, EPI_ISL_627487, EPI_ISL_627488, EPI_ISL_627489, EPI_ISL_627490	Wales Specialist Virology Centre Sequencing lab: Pathogen Genomics Unit	COVID-19 Genomics UK (COG-UK) Consortium	Catherine Moore, Johnathan Evans, Laura Gifford, Malorie Perry, Simon Cottrell, Angela Marchbank, Alec Birchley, Alexander Adams, Amy Gaskin, Bree Gatica-Wilcox, Jason Coombes, Joel Southgate, Lauren Gilbert, Lee Graham, Nicole Pacchiarini, Sara Kumziene-Summerhayes, Sarah Taylor, Sophie Jones, Sara Rey, Matthew Bull, Joanne Watkins, Sally Corden, Tom Connor
EPI_ISL_627491	University College London, Great Ormond Street Hospital for Children NHS Foundation Trust, Imperial College Healthcare NHS Trust	COVID-19 Genomics UK (COG-UK) Consortium	Sergi Castellano, Rachel Williams, Mark Kristiansen, Paola Resende Silva, Sunando Roy, Tony Brooks, Helena Tutill, Paola Niola, Patricia Dyal, Charlotte Williams, Leysa Forrest, Yasmin Panchbhaya, Jacqueline Findlay, Samuel Weeks, Julianne Brown, Kathryn Harris, Paul Randell, James Price, Alison Holmes, Judith Breuer
EPI_ISL_627492, EPI_ISL_627493, EPI_ISL_627494, EPI_ISL_627495, EPI_ISL_627496, EPI_ISL_627497, EPI_ISL_627498, EPI_ISL_627499, EPI_ISL_627500, EPI_ISL_627501, EPI_ISL_627502, EPI_ISL_627503, EPI_ISL_627504, EPI_ISL_627505, EPI_ISL_627506, EPI_ISL_627507, EPI_ISL_627508, EPI_ISL_627509, EPI_ISL_627510, EPI_ISL_627511, EPI_ISL_627512, EPI_ISL_627513, EPI_ISL_627514, EPI_ISL_627515, EPI_ISL_627516, EPI_ISL_627517			
see above	Virology Department, Sheffield Teaching Hospitals NHS Foundation Trust/Department of Infection, Immunity and Cardiovascular Disease, The Medical School, University of Sheffield	COVID-19 Genomics UK (COG-UK) Consortium	Thushan de Silva, Matthew Parker, Nikki Smith, Adri Angyal, Rebecca Brown, Luke Green, Rachel Tucker, Paul Parsons, Danielle Groves, Katie Johnson, Laura Carrilero, Alex Keeley, Dave Partridge, Matthew Wyles, Benjamin Lindsey, Mehmet Yavuz, Mohammad Raza, Cariad Evans
EPI_ISL_627518, EPI_ISL_627519, EPI_ISL_627520, EPI_ISL_627521, EPI_ISL_627522, EPI_ISL_627523, EPI_ISL_627524, EPI_ISL_627525, EPI_ISL_627526, EPI_ISL_627527, EPI_ISL_627528, EPI_ISL_627529, EPI_ISL_627530, EPI_ISL_627531, EPI_ISL_627532, EPI_ISL_627533, EPI_ISL_627534, EPI_ISL_627535,			

EPI_ISL_627536, EPI_ISL_627537, EPI_ISL_627538, EPI_ISL_627539, EPI_ISL_627540, EPI_ISL_627541, EPI_ISL_627542, EPI_ISL_627543, EPI_ISL_627544, EPI_ISL_627545, EPI_ISL_627546, EPI_ISL_627547, EPI_ISL_627548, EPI_ISL_627549, EPI_ISL_627550, EPI_ISL_627551, EPI_ISL_627552, EPI_ISL_627553, EPI_ISL_627554, EPI_ISL_627555, EPI_ISL_627556, EPI_ISL_627557, EPI_ISL_627558, EPI_ISL_627559, EPI_ISL_627560, EPI_ISL_627561, EPI_ISL_627562, EPI_ISL_627563, EPI_ISL_627564, EPI_ISL_627565, EPI_ISL_627566, EPI_ISL_627567, EPI_ISL_627568, EPI_ISL_627569			
see above	Regional Virus Laboratory, Belfast Health and Social Care Trust	COVID-19 Genomics UK (COG-UK) Consortium	Conall McCaughey, James McKenna, Tanya Curran, Susan Feeney, Alison Watt, Ciara Cox, Mairead Connor, Zoltan Molnar, David Simpson, Derek Fairley
EPI_ISL_627570, EPI_ISL_627571, EPI_ISL_627572, EPI_ISL_627573, EPI_ISL_627574, EPI_ISL_627575, EPI_ISL_627576, EPI_ISL_627577, EPI_ISL_627578, EPI_ISL_627579, EPI_ISL_627580, EPI_ISL_627581, EPI_ISL_627582, EPI_ISL_627583, EPI_ISL_627584, EPI_ISL_627585, EPI_ISL_627586, EPI_ISL_627587, EPI_ISL_627588, EPI_ISL_627589, EPI_ISL_627590, EPI_ISL_627591, EPI_ISL_627592, EPI_ISL_627593, EPI_ISL_627594, EPI_ISL_627595, EPI_ISL_627596, EPI_ISL_627597, EPI_ISL_627598, EPI_ISL_627599, EPI_ISL_627600, EPI_ISL_627601, EPI_ISL_627602, EPI_ISL_627603, EPI_ISL_627604, EPI_ISL_627605, EPI_ISL_627606, EPI_ISL_627607, EPI_ISL_627608, EPI_ISL_627609, EPI_ISL_627610, EPI_ISL_627611, EPI_ISL_627612, EPI_ISL_627613, EPI_ISL_627614, EPI_ISL_627615, EPI_ISL_627616, EPI_ISL_627617, EPI_ISL_627618, EPI_ISL_627619, EPI_ISL_627620, EPI_ISL_627621, EPI_ISL_627622, EPI_ISL_627623, EPI_ISL_627624, EPI_ISL_627625, EPI_ISL_627626, EPI_ISL_627627, EPI_ISL_627628, EPI_ISL_627629, EPI_ISL_627630, EPI_ISL_627631, EPI_ISL_627632, EPI_ISL_627633, EPI_ISL_627634, EPI_ISL_627635, EPI_ISL_627636, EPI_ISL_627637, EPI_ISL_627638, EPI_ISL_627639, EPI_ISL_627640, EPI_ISL_627641, EPI_ISL_627642, EPI_ISL_627643, EPI_ISL_627644, EPI_ISL_627645, EPI_ISL_627646, EPI_ISL_627647, EPI_ISL_627648, EPI_ISL_627649, EPI_ISL_627650, EPI_ISL_627651, EPI_ISL_627652, EPI_ISL_627653, EPI_ISL_627654, EPI_ISL_627655, EPI_ISL_627656, EPI_ISL_627657, EPI_ISL_627658, EPI_ISL_627659, EPI_ISL_627660, EPI_ISL_627661, EPI_ISL_627662, EPI_ISL_627663, EPI_ISL_627664, EPI_ISL_627665			
see above	Quadram Institute Bioscience	COVID-19 Genomics UK (COG-UK) Consortium	Dave J. Baker, Gemma L. Kay, Alp Aydin, Thanh Le-Viet, Steven Rudder, Ana P. Tedim, Anastasia Kolyva, Maria Diaz, Leonardo de Oliveira Martins, Nabil-Fareed Alikhan, Lizzie Meadows, Rachael Stanley, Ngozi Elumogo, Muhammed Yasir, Nicholas M. Thomson, Alexander J Trotter, Rachel Gilroy, Samuel Bloomfield, Claire Stuart, Andrew Bell, Reenesh Prakash, Samir Dervisevic, Alison E. Mather, John Wain, Mark Webber, Andrew J. Page, Justin O'Grady
EPI_ISL_627666, EPI_ISL_627667, EPI_ISL_627668, EPI_ISL_627669, EPI_ISL_627670, EPI_ISL_627671, EPI_ISL_627672, EPI_ISL_627673, EPI_ISL_627674, EPI_ISL_627675, EPI_ISL_627676, EPI_ISL_627677, EPI_ISL_627678, EPI_ISL_627679, EPI_ISL_627680, EPI_ISL_627681, EPI_ISL_627682, EPI_ISL_627683, EPI_ISL_627684, EPI_ISL_627685, EPI_ISL_627686, EPI_ISL_627687, EPI_ISL_627688, EPI_ISL_627689, EPI_ISL_627690, EPI_ISL_627691, EPI_ISL_627692, EPI_ISL_627693, EPI_ISL_627694, EPI_ISL_627695, EPI_ISL_627696, EPI_ISL_627697, EPI_ISL_627698, EPI_ISL_627699, EPI_ISL_627700, EPI_ISL_627701, EPI_ISL_627702, EPI_ISL_627703, EPI_ISL_627704, EPI_ISL_627705, EPI_ISL_627706, EPI_ISL_627707, EPI_ISL_627708, EPI_ISL_627709, EPI_ISL_627710, EPI_ISL_627711, EPI_ISL_627712, EPI_ISL_627713, EPI_ISL_627714, EPI_ISL_627715, EPI_ISL_627716, EPI_ISL_627717, EPI_ISL_627718, EPI_ISL_627719, EPI_ISL_627720, EPI_ISL_627721, EPI_ISL_627722, EPI_ISL_627723, EPI_ISL_627724, EPI_ISL_627725, EPI_ISL_627726, EPI_ISL_627727, EPI_ISL_627728, EPI_ISL_627729			
see above	Queens Medical Centre, Clinical Microbiology Department / DeepSeq Nottingham	COVID-19 Genomics UK (COG-UK) Consortium	Gemma Clark, Wendy Smith, Manjinder Khakh, Vicki M Fleming, Michelle M Lister, Hannah Howson-Wells, Jonathan Ball, Patrick McClure, Joseph Chappell, Theocharis Tsoleridis, Nadine Holmes, Matthew Carlisle, Christopher Moore, Fei Sang, Johnny Debebe, Victoria Wright, Matthew Loose
EPI_ISL_627730, EPI_ISL_627731, EPI_ISL_627732, EPI_ISL_627733, EPI_ISL_627734, EPI_ISL_627735, EPI_ISL_627736, EPI_ISL_627737, EPI_ISL_627738, EPI_ISL_627739, EPI_ISL_627740, EPI_ISL_627741, EPI_ISL_627742, EPI_ISL_627743, EPI_ISL_627744, EPI_ISL_627745, EPI_ISL_627746, EPI_ISL_627747, EPI_ISL_627748, EPI_ISL_627749, EPI_ISL_627750, EPI_ISL_627751, EPI_ISL_627752, EPI_ISL_627753, EPI_ISL_627754, EPI_ISL_627755, EPI_ISL_627756, EPI_ISL_627757, EPI_ISL_627758, EPI_ISL_627759, EPI_ISL_627760, EPI_ISL_627761, EPI_ISL_627762, EPI_ISL_627763, EPI_ISL_627764, EPI_ISL_627765, EPI_ISL_627766, EPI_ISL_627767, EPI_ISL_627768, EPI_ISL_627769, EPI_ISL_627770, EPI_ISL_627771, EPI_ISL_627772, EPI_ISL_627773, EPI_ISL_627774, EPI_ISL_627775, EPI_ISL_627776, EPI_ISL_627777, EPI_ISL_627778, EPI_ISL_627779, EPI_ISL_627780, EPI_ISL_627781, EPI_ISL_627782, EPI_ISL_627783, EPI_ISL_627784, EPI_ISL_627785, EPI_ISL_627786, EPI_ISL_627787, EPI_ISL_627788, EPI_ISL_627789, EPI_ISL_627790, EPI_ISL_627791, EPI_ISL_627792, EPI_ISL_627793, EPI_ISL_627794, EPI_ISL_627795, EPI_ISL_627796, EPI_ISL_627797, EPI_ISL_627798, EPI_ISL_627799, EPI_ISL_627800, EPI_ISL_627801, EPI_ISL_627802, EPI_ISL_627803, EPI_ISL_627804, EPI_ISL_627805, EPI_ISL_627806, EPI_ISL_627807, EPI_ISL_627808, EPI_ISL_627809, EPI_ISL_627810, EPI_ISL_627811, EPI_ISL_627812, EPI_ISL_627813, EPI_ISL_627814, EPI_ISL_627815, EPI_ISL_627816, EPI_ISL_627817, EPI_ISL_627818, EPI_ISL_627819, EPI_ISL_627820, EPI_ISL_627821, EPI_ISL_627822, EPI_ISL_627823, EPI_ISL_627824, EPI_ISL_627825, EPI_ISL_627826, EPI_ISL_627827, EPI_ISL_627828, EPI_ISL_627829, EPI_ISL_627830, EPI_ISL_627831, EPI_ISL_627832, EPI_ISL_627833, EPI_ISL_627834, EPI_ISL_627835, EPI_ISL_627836, EPI_ISL_627837, EPI_ISL_627838, EPI_ISL_627839, EPI_ISL_627840, EPI_ISL_627841, EPI_ISL_627842, EPI_ISL_627843, EPI_ISL_627844, EPI_ISL_627845, EPI_ISL_627846, EPI_ISL_627847, EPI_ISL_627848, EPI_ISL_627849, EPI_ISL_627850, EPI_ISL_627851, EPI_ISL_627852, EPI_ISL_627853, EPI_ISL_627854, EPI_ISL_627855, EPI_ISL_627856, EPI_ISL_627857, EPI_ISL_627858, EPI_ISL_627859, EPI_ISL_627860, EPI_ISL_627861, EPI_ISL_627862, EPI_ISL_627863, EPI_ISL_627864, EPI_ISL_627865, EPI_ISL_627866, EPI_ISL_627867, EPI_ISL_627868, EPI_ISL_627869, EPI_ISL_627870, EPI_ISL_627871, EPI_ISL_627872, EPI_ISL_627873, EPI_ISL_627874, EPI_ISL_627875, EPI_ISL_627876, EPI_ISL_627877, EPI_ISL_627878, EPI_ISL_627879, EPI_ISL_627880, EPI_ISL_627881, EPI_ISL_627882, EPI_ISL_627883, EPI_ISL_627884, EPI_ISL_627885, EPI_ISL_627886, EPI_ISL_627887, EPI_ISL_627888, EPI_ISL_627889, EPI_ISL_627890, EPI_ISL_627891, EPI_ISL_627892, EPI_ISL_627893, EPI_ISL_627894, EPI_ISL_627895, EPI_ISL_627896, EPI_ISL_627897, EPI_ISL_627898, EPI_ISL_627899, EPI_ISL_627900, EPI_ISL_627901, EPI_ISL_627902, EPI_ISL_627903, EPI_ISL_627904, EPI_ISL_627905, EPI_ISL_627906, EPI_ISL_627907, EPI_ISL_627908, EPI_ISL_627909, EPI_ISL_627910, EPI_ISL_627911, EPI_ISL_627912, EPI_ISL_627913, EPI_ISL_627914, EPI_ISL_627915, EPI_ISL_627916, EPI_ISL_627917, EPI_ISL_627918, EPI_ISL_627919, EPI_ISL_627920, EPI_ISL_627921, EPI_ISL_627922, EPI_ISL_627923, EPI_ISL_627924, EPI_ISL_627925, EPI_ISL_627926, EPI_ISL_627927, EPI_ISL_627928, EPI_ISL_627929, EPI_ISL_627930, EPI_ISL_627931, EPI_ISL_627932, EPI_ISL_627933, EPI_ISL_627934, EPI_ISL_627935, EPI_ISL_627936, EPI_ISL_627937, EPI_ISL_627938, EPI_ISL_627939, EPI_ISL_627940, EPI_ISL_627941, EPI_ISL_627942, EPI_ISL_627943, EPI_ISL_627944, EPI_ISL_627945, EPI_ISL_627946, EPI_ISL_627947, EPI_ISL_627948, EPI_ISL_627949, EPI_ISL_627950, EPI_ISL_627951, EPI_ISL_627952, EPI_ISL_627953, EPI_ISL_627954, EPI_ISL_627955, EPI_ISL_627956, EPI_ISL_627957, EPI_ISL_627958, EPI_ISL_627959, EPI_ISL_627960, EPI_ISL_627961, EPI_ISL_627962, EPI_ISL_627963, EPI_ISL_627964, EPI_ISL_627965, EPI_ISL_627966, EPI_ISL_627967, EPI_ISL_627968, EPI_ISL_627969, EPI_ISL_627970, EPI_ISL_627971, EPI_ISL_627972, EPI_ISL_627973, EPI_ISL_627974, EPI_ISL_627975, EPI_ISL_627976, EPI_ISL_627977, EPI_ISL_627978, EPI_ISL_627979, EPI_ISL_627980, EPI_ISL_627981, EPI_ISL_627982, EPI_ISL_627983, EPI_ISL_627984, EPI_ISL_627985, EPI_ISL_627986, EPI_ISL_627987, EPI_ISL_627988, EPI_ISL_627989, EPI_ISL_627990, EPI_ISL_627991, EPI_ISL_627992, EPI_ISL_627993, EPI_ISL_627994, EPI_ISL_627995, EPI_ISL_627996, EPI_ISL_627997, EPI_ISL_627998, EPI_ISL_627999, EPI_ISL_628000, EPI_ISL_628001, EPI_ISL_628002, EPI_ISL_628003, EPI_ISL_628004, EPI_ISL_628005, EPI_ISL_628006, EPI_ISL_628007, EPI_ISL_628008, EPI_ISL_628009, EPI_ISL_628010, EPI_ISL_628011, EPI_ISL_628012, EPI_ISL_628013, EPI_ISL_628014, EPI_ISL_628015, EPI_ISL_628016, EPI_ISL_628017, EPI_ISL_628018, EPI_ISL_628019, EPI_ISL_628020, EPI_ISL_628021, EPI_ISL_628022, EPI_ISL_628023, EPI_ISL_628024, EPI_ISL_628025, EPI_ISL_628026, EPI_ISL_628027, EPI_ISL_628028, EPI_ISL_628029, EPI_ISL_628030, EPI_ISL_628031, EPI_ISL_628032, EPI_ISL_628033, EPI_ISL_628034, EPI_ISL_628035, EPI_ISL_628036, EPI_ISL_628037, EPI_ISL_628038, EPI_ISL_628039, EPI_ISL_628040, EPI_ISL_628041, EPI_ISL_628042, EPI_ISL_628043, EPI_ISL_628044, EPI_ISL_628045, EPI_ISL_628046, EPI_ISL_628047, EPI_ISL_628048, EPI_ISL_628049, EPI_ISL_628050, EPI_ISL_628051, EPI_ISL_628052, EPI_ISL_628053, EPI_ISL_628054, EPI_ISL_628055, EPI_ISL_628056, EPI_ISL_628057, EPI_ISL_628058, EPI_ISL_628059, EPI_ISL_628060, EPI_ISL_628061, EPI_ISL_628062, EPI_ISL_628063, EPI_ISL_628064, EPI_ISL_628065, EPI_ISL_628066, EPI_ISL_628067, EPI_ISL_628068, EPI_ISL_628069, EPI_ISL_628070, EPI_ISL_628071, EPI_ISL_628072, EPI_ISL_628073, EPI_ISL_628074, EPI_ISL_628075, EPI_ISL_628076, EPI_ISL_628077, EPI_ISL_628078, EPI_ISL_628079, EPI_ISL_628080, EPI_ISL_628081, EPI_ISL_628082, EPI_ISL_628083, EPI_ISL_628084, EPI_ISL_628085, EPI_ISL_628086, EPI_ISL_628087, EPI_ISL_628088, EPI_ISL_628089, EPI_ISL_628090, EPI_ISL_628091, EPI_ISL_628092, EPI_ISL_628093, EPI_ISL_628094, EPI_ISL_628095, EPI_ISL_628096, EPI_ISL_628097, EPI_ISL_628098, EPI_ISL_628099, EPI_ISL_628100, EPI_ISL_628101, EPI_ISL_628102, EPI_ISL_628103, EPI_ISL_628104, EPI_ISL_628105, EPI_ISL_628106, EPI_ISL_628107, EPI_ISL_628108, EPI_ISL_628109, EPI_ISL_628110, EPI_ISL_628111, EPI_ISL_628112, EPI_ISL_628113, EPI_ISL_628114, EPI_ISL_628115, EPI_ISL_628116, EPI_ISL_628117, EPI_ISL_628118, EPI_ISL_628119, EPI_ISL_628120, EPI_ISL_628121, EPI_ISL_628122, EPI_ISL_628123, EPI_ISL_628124, EPI_ISL_628125, EPI_ISL_628126, EPI_ISL_628127, EPI_ISL_628128, EPI_ISL_628129, EPI_ISL_628130, EPI_ISL_628131, EPI_ISL_628132, EPI_ISL_628133, EPI_ISL_628134, EPI_ISL_628135, EPI_ISL_628136, EPI_ISL_628137, EPI_ISL_628138, EPI_ISL_628139, EPI_ISL_628140, EPI_ISL_628141, EPI_ISL_628142, EPI_ISL_628143, EPI_ISL_628144, EPI_ISL_628145, EPI_ISL_628146, EPI_ISL_628147, EPI_ISL_628148, EPI_ISL_628149, EPI_ISL_628150, EPI_ISL_628151, EPI_ISL_628152, EPI_ISL_628153, EPI_ISL_628154, EPI_ISL_628155, EPI_ISL_628156, EPI_ISL_628157, EPI_ISL_628158, EPI_ISL_628159, EPI_ISL_628160, EPI_ISL_628161, EPI_ISL_628162, EPI_ISL_628163, EPI_ISL_628164, EPI_ISL_628165, EPI_ISL_628166, EPI_ISL_628167, EPI_ISL_628168, EPI_ISL_628169, EPI_ISL_628170, EPI_ISL_628171, EPI_ISL_628172, EPI_ISL_628173, EPI_ISL_628174, EPI_ISL_628175, EPI_ISL_628176, EPI_ISL_628177, EPI_ISL_628178, EPI_ISL_628179, EPI_ISL_628180, EPI_ISL_628181, EPI_ISL_628182, EPI_ISL_628183, EPI_ISL_628184, EPI_ISL_628185, EPI_ISL_628186, EPI_ISL_628187, EPI_ISL_628188, EPI_ISL_628189, EPI_ISL_628190, EPI_ISL_628191, EPI_ISL_628192, EPI_ISL_628193, EPI_ISL_628194, EPI_ISL_628195, EPI_ISL_628196, EPI_ISL_628197, EPI_ISL_628198, EPI_ISL_628199, EPI_ISL_628200, EPI_ISL_628201, EPI_ISL_628202, EPI_ISL_628203, EPI_ISL_628204, EPI_ISL_628205, EPI_ISL_628206, EPI_ISL_628207, EPI_ISL_628208, EPI_ISL_628209, EPI_ISL_628210, EPI_ISL_628211, EPI_ISL_628212, EPI_ISL_628213, EPI_ISL_628214, EPI_ISL_628215, EPI_ISL_628216, EPI_ISL_628217, EPI_ISL_628218, EPI_ISL_628219, EPI_ISL_628220, EPI_ISL_628221, EPI_ISL_628222, EPI_ISL_628223, EPI_ISL_628224, EPI_ISL_628225, EPI_ISL_628226, EPI_ISL_628227, EPI_ISL_628228, EPI_ISL_628229, EPI_ISL_628230, EPI_ISL_628231, EPI_ISL_628232, EPI_ISL_628233, EPI_ISL_628234, EPI_ISL_628235, EPI_ISL_628236, EPI_ISL_628237, EPI_ISL_628238, EPI_ISL_628239, EPI_ISL_628240, EPI_ISL_628241, EPI_ISL_628242, EPI_ISL_628243, EPI_ISL_628244, EPI_ISL_628245, EPI_ISL_628246, EPI_ISL_628247, EPI_ISL_628248, EPI_ISL_628249, EPI_ISL_628250, EPI_ISL_628251, EPI_ISL_628252, EPI_ISL_628253, EPI_ISL_628254, EPI_ISL_628255, EPI_ISL_628256, EPI_ISL_628257, EPI_ISL_628258, EPI_ISL_628259, EPI_ISL_628260, EPI_ISL_628261, EPI_ISL_628262, EPI_ISL_628263, EPI_ISL_628264, EPI_ISL_628265, EPI_ISL_628266, EPI_ISL_628267, EPI_ISL_628268, EPI_ISL_628269, EPI_ISL_628270, EPI_ISL_628271, EPI_ISL_628272, EPI_ISL_628273, EPI_ISL_628274, EPI_ISL_628275, EPI_ISL_628276, EPI_ISL_628277, EPI_ISL_628278, EPI_ISL_628279, EPI_ISL_628280, EPI_ISL_628281, EPI_ISL_628282, EPI_ISL_628283, EPI_ISL_628284, EPI_ISL_628285, EPI_ISL_628286, EPI_ISL_628287, EPI_ISL_628288, EPI_ISL_628289, EPI_ISL_628290, EPI_ISL_628291, EPI_ISL_628292, EPI_ISL_628293, EPI_ISL_628294, EPI_ISL_628295, EPI_ISL_628296, EPI_ISL_628297, EPI_ISL_628298, EPI_ISL_628299, EPI_ISL_628300, EPI_ISL_628301, EPI_ISL_628302, EPI_ISL_628303, EPI_ISL_628304, EPI_ISL_628305, EPI_ISL_628306, EPI_ISL_628307, EPI_ISL_628308, EPI_ISL_628309, EPI_ISL_628310, EPI_ISL_628311			
see above	Wales Specialist Virology Centre Sequencing lab: Pathogen Genomics Unit	COVID-19 Genomics UK (COG-UK) Consortium	Catherine Moore, Johnathan Evans, Laura Gifford, Malorie Perry, Simon Cottrell, Angela Marchbank, Alec Birchley, Alexander Adams, Amy Gaskin, Bree Gatica-Wilcox, Jason Coombes, Joel Southgate, Lauren Gilbert, Lee Graham, Nicole Pacchiarini, Sara Kumziene-Summerhayes, Sarah Taylor, Sophie Jones, Sara Rey, Matthew Bull, Joanne Watkins, Sally Corden, Tom Connor
EPI_ISL_628312, EPI_ISL_628313, EPI_ISL_628314, EPI_ISL_628315, EPI_ISL_628316, EPI_ISL_628317, EPI_ISL_628318, EPI_ISL_628319, EPI_ISL_628320, EPI_ISL_628321, EPI_ISL_628322, EPI_ISL_628323, EPI_ISL_628324, EPI_ISL_628325, EPI_ISL_628326, EPI_ISL_628327, EPI_ISL_628328, EPI_ISL_628329, EPI_ISL_628330, EPI_ISL_628331, EPI_ISL_628332, EPI_ISL_628333			
see above	Centre for Enzyme Innovation, University of Portsmouth / Translational Research Laboratory, Portsmouth Hospitals NHS Trust	COVID-19 Genomics UK (COG-UK) Consortium	Angela Beckett,Yann Bourgeois,Garry Scarlett,Sharon Glaysher,Scott Elliott,Kelly Bicknell,Robert Impey,Allyson Lloyd,Sarah Wyllie,Ethan Butcher,Anoop Chauhan,Samuel Robson
EPI_ISL_628334, EPI_ISL_628335, EPI_ISL_628336, EPI_ISL_628337, EPI_ISL_628338, EPI_ISL_628339, EPI_ISL_628340, EPI_ISL_628341, EPI_ISL_628342, EPI_ISL_628343, EPI_ISL_628344, EPI_ISL_628345, EPI_ISL_628346, EPI_ISL_628347, EPI_ISL_628348, EPI_ISL_628349, EPI_ISL_628350, EPI_ISL_628351, EPI_ISL_628352, EPI_ISL_628353, EPI_ISL_628354, EPI_ISL_628355, EPI_ISL_628356, EPI_ISL_628357, EPI_ISL_628358, EPI_ISL_628359, EPI_ISL_628360, EPI_ISL_628361, EPI_ISL_628362, EPI_ISL_628363, EPI_ISL_628364, EPI_ISL_628365, EPI_ISL_628366, EPI_ISL_628367, EPI_ISL_628368			
see above	Oxford Viromics, NDM, University of Oxford; Oxford University Hospitals; Basingstoke and North Hampshire Hospital	COVID-19 Genomics UK (COG-UK) Consortium	Tanya Golubchik, David Bonsall, George Macintyre, Amy Trebes, Mariateresa de Cesare, Catrin Moore, Alex Mobbs, Anita Justice, Robert Shaw, Monique Andersson, Timothy Peto, Emma Wise, Nathan Moore, Jessica Lynch, Nick Cortes, Matilde Mori, Stephen Kidd, David Buck, John Todd, Christophe Fraser
EPI_ISL_628369, EPI_ISL_628370, EPI_ISL_628371, EPI_ISL_628372, EPI_ISL_628373, EPI_ISL_628374, EPI_ISL_628375, EPI_ISL_628376, EPI_ISL_628377, EPI_ISL_628378, EPI_ISL_628379, EPI_ISL_628380, EPI_ISL_628381, EPI_ISL_628382, EPI_ISL_628383, EPI_ISL_628384, EPI_ISL_628385, EPI_ISL_628386, EPI_ISL_628387, EPI_ISL_628388, EPI_ISL_628389, EPI_ISL_628390, EPI_ISL_628391, EPI_ISL_628392, EPI_ISL_628393, EPI_ISL_628394, EPI_ISL_628395, EPI_ISL_628396, EPI_ISL_628397, EPI_ISL_628398, EPI_ISL_628399, EPI_ISL_628400, EPI_ISL_628401, EPI_ISL_628402, EPI_ISL_628403, EPI_ISL_628404, EPI_ISL_628405, EPI_ISL_628406, EPI_ISL_628407, EPI_ISL_628408, EPI_ISL_628409, EPI_ISL_628410, EPI_ISL_628411, EPI_ISL_628412, EPI_ISL_628413			
see above	Virology Department, Sheffield Teaching Hospitals NHS Foundation Trust/Department of Infection, Immunity and Cardiovascular Disease, The Medical School, University of Sheffield	COVID-19 Genomics UK (COG-UK) Consortium	Thushan de Silva, Matthew Parker, Nikki Smith, Adri Angyal, Rebecca Brown, Luke Green, Rachel Tucker, Paul Parsons, Danielle Groves, Katie Johnson, Laura Carrilero, Alex Keeley, Dave Partridge, Matthew Wyles, Benjamin Lindsey, Mehmet Yavuz, Mohammad Raza, Cariad Evans
EPI_ISL_628414, EPI_ISL_628415, EPI_ISL_628416, EPI_ISL_628417, EPI_ISL_628418, EPI_ISL_628419, EPI_ISL_628420	Oxford Viromics, NDM, University of Oxford; Oxford University Hospitals; Basingstoke and North Hampshire Hospital	COVID-19 Genomics UK (COG-UK) Consortium	Tanya Golubchik, David Bonsall, George Macintyre, Amy Trebes, Mariateresa de Cesare, Catrin Moore, Alex Mobbs, Anita Justice, Robert Shaw, Monique Andersson, Timothy Peto, Emma Wise, Nathan Moore, Jessica Lynch, Nick Cortes, Matilde Mori, Stephen Kidd, David Buck, John Todd, Christophe Fraser
EPI_ISL_628421	Virology Department, Sheffield Teaching Hospitals NHS Foundation Trust/Department of Infection, Immunity and Cardiovascular Disease, The Medical School, University of Sheffield	COVID-19 Genomics UK (COG-UK) Consortium	Thushan de Silva, Matthew Parker, Nikki Smith, Adri Angyal, Rebecca Brown, Luke Green, Rachel Tucker, Paul Parsons, Danielle Groves, Katie Johnson, Laura Carrilero, Alex Keeley, Dave Partridge, Matthew Wyles, Benjamin Lindsey, Mehmet Yavuz, Mohammad Raza, Cariad Evans

EPI_ISL_628422, EPI_ISL_628423, EPI_ISL_628424, EPI_ISL_628425, EPI_ISL_628426, EPI_ISL_628427, EPI_ISL_628428, EPI_ISL_628429, EPI_ISL_628430, EPI_ISL_628431, EPI_ISL_628432, EPI_ISL_628433, EPI_ISL_628434, EPI_ISL_628435, EPI_ISL_628436, EPI_ISL_628437, EPI_ISL_628438, EPI_ISL_628439, EPI_ISL_628440, EPI_ISL_628441, EPI_ISL_628442, EPI_ISL_628443, EPI_ISL_628444, EPI_ISL_628445, EPI_ISL_628446, EPI_ISL_628447, EPI_ISL_628448, EPI_ISL_628449, EPI_ISL_628450, EPI_ISL_628451, EPI_ISL_628452, EPI_ISL_628453, EPI_ISL_628454, EPI_ISL_628455, EPI_ISL_628456, EPI_ISL_628457, EPI_ISL_628458, EPI_ISL_628459, EPI_ISL_628460, EPI_ISL_628461, EPI_ISL_628462, EPI_ISL_628463, EPI_ISL_628464, EPI_ISL_628465, EPI_ISL_628466, EPI_ISL_628467, EPI_ISL_628468, EPI_ISL_628469, EPI_ISL_628470, EPI_ISL_628471, EPI_ISL_628472, EPI_ISL_628473, EPI_ISL_628474, EPI_ISL_628475, EPI_ISL_628476, EPI_ISL_628477, EPI_ISL_628478, EPI_ISL_628479, EPI_ISL_628480, EPI_ISL_628481, EPI_ISL_628482, EPI_ISL_628483, EPI_ISL_628484, EPI_ISL_628485, EPI_ISL_628486, EPI_ISL_628487, EPI_ISL_628488, EPI_ISL_628489, EPI_ISL_628490, EPI_ISL_628491, EPI_ISL_628492, EPI_ISL_628493, EPI_ISL_628494, EPI_ISL_628495, EPI_ISL_628496, EPI_ISL_628497, EPI_ISL_628498, EPI_ISL_628499, EPI_ISL_628500, EPI_ISL_628501, EPI_ISL_628502, EPI_ISL_628503, EPI_ISL_628504, EPI_ISL_628505, EPI_ISL_628506, EPI_ISL_628507, EPI_ISL_628508, EPI_ISL_628509, EPI_ISL_628510, EPI_ISL_628511, EPI_ISL_628512, EPI_ISL_628513, EPI_ISL_628514, EPI_ISL_628515, EPI_ISL_628516, EPI_ISL_628517, EPI_ISL_628518, EPI_ISL_628519, EPI_ISL_628520, EPI_ISL_628521, EPI_ISL_628522, EPI_ISL_628523, EPI_ISL_628524, EPI_ISL_628525, EPI_ISL_628526, EPI_ISL_628527, EPI_ISL_628528, EPI_ISL_628529, EPI_ISL_628530, EPI_ISL_628531, EPI_ISL_628532, EPI_ISL_628533, EPI_ISL_628534, EPI_ISL_628535, EPI_ISL_628536, EPI_ISL_628537, EPI_ISL_628538, EPI_ISL_628539, EPI_ISL_628540, EPI_ISL_628541, EPI_ISL_628542, EPI_ISL_628543, EPI_ISL_628544, EPI_ISL_628545, EPI_ISL_628546, EPI_ISL_628547, EPI_ISL_628548, EPI_ISL_628549, EPI_ISL_628550, EPI_ISL_628551, EPI_ISL_628552, EPI_ISL_628553, EPI_ISL_628554, EPI_ISL_628555, EPI_ISL_628556, EPI_ISL_628557, EPI_ISL_628558, EPI_ISL_628559, EPI_ISL_628560, EPI_ISL_628561, EPI_ISL_628562, EPI_ISL_628563, EPI_ISL_628564, EPI_ISL_628565, EPI_ISL_628566, EPI_ISL_628567, EPI_ISL_628568, EPI_ISL_628569, EPI_ISL_628570, EPI_ISL_628571, EPI_ISL_628572, EPI_ISL_628573, EPI_ISL_628574, EPI_ISL_628575, EPI_ISL_628576, EPI_ISL_628577, EPI_ISL_628578, EPI_ISL_628579, EPI_ISL_628580, EPI_ISL_628581, EPI_ISL_628582, EPI_ISL_628583, EPI_ISL_628584, EPI_ISL_628585, EPI_ISL_628586, EPI_ISL_628587, EPI_ISL_628588, EPI_ISL_628589, EPI_ISL_628590, EPI_ISL_628591, EPI_ISL_628592, EPI_ISL_628593, EPI_ISL_628594, EPI_ISL_628595, EPI_ISL_628596, EPI_ISL_628597, EPI_ISL_628598, EPI_ISL_628599, EPI_ISL_628600, EPI_ISL_628601, EPI_ISL_628602, EPI_ISL_628603, EPI_ISL_628604, EPI_ISL_628605, EPI_ISL_628606, EPI_ISL_628607, EPI_ISL_628608, EPI_ISL_628609, EPI_ISL_628610, EPI_ISL_628611, EPI_ISL_628612, EPI_ISL_628613, EPI_ISL_628614, EPI_ISL_628615, EPI_ISL_628616, EPI_ISL_628617, EPI_ISL_628618, EPI_ISL_628619, EPI_ISL_628620, EPI_ISL_628621, EPI_ISL_628622, EPI_ISL_628623, EPI_ISL_628624, EPI_ISL_628625, EPI_ISL_628626, EPI_ISL_628627, EPI_ISL_628628, EPI_ISL_628629, EPI_ISL_628630, EPI_ISL_628631, EPI_ISL_628632, EPI_ISL_628633, EPI_ISL_628634, EPI_ISL_628635, EPI_ISL_628636, EPI_ISL_628637, EPI_ISL_628638, EPI_ISL_628639, EPI_ISL_628640, EPI_ISL_628641, EPI_ISL_628642, EPI_ISL_628643, EPI_ISL_628644, EPI_ISL_628645, EPI_ISL_628646, EPI_ISL_628647, EPI_ISL_628648, EPI_ISL_628649, EPI_ISL_628650, EPI_ISL_628651, EPI_ISL_628652, EPI_ISL_628653, EPI_ISL_628654, EPI_ISL_628655, EPI_ISL_628656, EPI_ISL_628657, EPI_ISL_628658, EPI_ISL_628659, EPI_ISL_628660, EPI_ISL_628661, EPI_ISL_628662, EPI_ISL_628663, EPI_ISL_628664, EPI_ISL_628665, EPI_ISL_628666, EPI_ISL_628667, EPI_ISL_628668, EPI_ISL_628669, EPI_ISL_628670, EPI_ISL_628671, EPI_ISL_628672, EPI_ISL_628673, EPI_ISL_628674, EPI_ISL_628675, EPI_ISL_628676, EPI_ISL_628677, EPI_ISL_628678, EPI_ISL_628679, EPI_ISL_628680, EPI_ISL_628681, EPI_ISL_628682, EPI_ISL_628683, EPI_ISL_628684, EPI_ISL_628685, EPI_ISL_628686, EPI_ISL_628687, EPI_ISL_628688, EPI_ISL_628689, EPI_ISL_628690, EPI_ISL_628691, EPI_ISL_628692, EPI_ISL_628693, EPI_ISL_628694, EPI_ISL_628695, EPI_ISL_628696, EPI_ISL_628697, EPI_ISL_628698, EPI_ISL_628699, EPI_ISL_628700			
see above	Oxford Viromics, NDM, University of Oxford; Oxford University Hospitals; Basingstoke and North Hampshire Hospital	COVID-19 Genomics UK (COG-UK) Consortium	Tanya Golubchik, David Bonsall, George Macintyre, Amy Trebes, Mariateresa de Cesare, Catrin Moore, Alex Mobbs, Anita Justice, Robert Shaw, Monique Andersson, Timothy Peto, Emma Wise, Nathan Moore, Jessica Lynch, Nick Cortes, Matilde Mori, Stephen Kidd, David Buck, John Todd, Christophe Fraser
EPI_ISL_628804, EPI_ISL_628805, EPI_ISL_628806, EPI_ISL_628807, EPI_ISL_628808, EPI_ISL_628809, EPI_ISL_628810, EPI_ISL_628811, EPI_ISL_628813, EPI_ISL_628814, EPI_ISL_628815, EPI_ISL_628816, EPI_ISL_628817, EPI_ISL_628818, EPI_ISL_628819, EPI_ISL_628820, EPI_ISL_628821, EPI_ISL_628822, EPI_ISL_628823, EPI_ISL_628824, EPI_ISL_628826, EPI_ISL_628828, EPI_ISL_628829, EPI_ISL_628830, EPI_ISL_628831, EPI_ISL_628832, EPI_ISL_628833, EPI_ISL_628834, EPI_ISL_628835, EPI_ISL_628836, EPI_ISL_628839, EPI_ISL_628840, EPI_ISL_628841, EPI_ISL_628842, EPI_ISL_628843, EPI_ISL_628844, EPI_ISL_628845, EPI_ISL_628846, EPI_ISL_628847, EPI_ISL_628848, EPI_ISL_628849, EPI_ISL_628851, EPI_ISL_628853, EPI_ISL_628856, EPI_ISL_628857, EPI_ISL_628859, EPI_ISL_628860, EPI_ISL_628861, EPI_ISL_628862, EPI_ISL_628863, EPI_ISL_628867, EPI_ISL_628869, EPI_ISL_628870, EPI_ISL_628873, EPI_ISL_628874, EPI_ISL_628875, EPI_ISL_628876, EPI_ISL_628878, EPI_ISL_628879, EPI_ISL_628880, EPI_ISL_628881, EPI_ISL_628882, EPI_ISL_628884			
see above	University of Michigan Clinical Microbiology Laboratory	Lauring Lab, University of Michigan, Department of Microbiology and Immunology	Valesano
EPI_ISL_628932, EPI_ISL_628933, EPI_ISL_628934, EPI_ISL_628935, EPI_ISL_628936, EPI_ISL_628937, EPI_ISL_628938, EPI_ISL_628939, EPI_ISL_628940, EPI_ISL_628941, EPI_ISL_628943, EPI_ISL_628945, EPI_ISL_628951, EPI_ISL_628953, EPI_ISL_628954, EPI_ISL_628955, EPI_ISL_628958, EPI_ISL_628959, EPI_ISL_628960, EPI_ISL_628961, EPI_ISL_628964, EPI_ISL_628967, EPI_ISL_628968, EPI_ISL_628969, EPI_ISL_628971, EPI_ISL_628972, EPI_ISL_628975, EPI_ISL_628976, EPI_ISL_628977, EPI_ISL_628978, EPI_ISL_628979, EPI_ISL_628981, EPI_ISL_628983, EPI_ISL_628984, EPI_ISL_628985, EPI_ISL_628986, EPI_ISL_628987, EPI_ISL_628989, EPI_ISL_628990, EPI_ISL_628991, EPI_ISL_628992, EPI_ISL_628993, EPI_ISL_628994, EPI_ISL_628996, EPI_ISL_628997, EPI_ISL_628998, EPI_ISL_628999			
see above	Utah Public Health Laboratory	Utah Public Health Laboratory	Erin Young, Kelly Oakeson
EPI_ISL_629001	South Eastern Area Laboratory Services (SEALS)	NSW Health Pathology - Institute of Clinical Pathology and Medical Research; Westmead Hospital; University of Sydney	CIDM-PH et al.
EPI_ISL_629002	Sydney South West Pathology Service (SSWPS) - Liverpool Hospital - NSW Health Pathology	NSW Health Pathology - Institute of Clinical Pathology and Medical Research; Westmead Hospital; University of Sydney	CIDM-PH et al.
EPI_ISL_629003, EPI_ISL_629004, EPI_ISL_629005, EPI_ISL_629006	South Eastern Area Laboratory Services (SEALS)	NSW Health Pathology - Institute of Clinical Pathology and Medical Research; Westmead Hospital; University of Sydney	CIDM-PH et al.
EPI_ISL_629007, EPI_ISL_629008, EPI_ISL_629009	Laverty Pathology	NSW Health Pathology - Institute of Clinical Pathology and Medical Research; Westmead Hospital; University of Sydney	CIDM-PH et al.
EPI_ISL_629010	Sydney South West Pathology Service (SSWPS) - Royal Prince Alfred Hospital - NSW Health Pathology	NSW Health Pathology - Institute of Clinical Pathology and Medical Research; Westmead Hospital; University of Sydney	CIDM-PH et al.
EPI_ISL_629011, EPI_ISL_629012, EPI_ISL_629013, EPI_ISL_629014, EPI_ISL_629015, EPI_ISL_629016, EPI_ISL_629017, EPI_ISL_629018, EPI_ISL_629019, EPI_ISL_629020, EPI_ISL_629021, EPI_ISL_629022, EPI_ISL_629023, EPI_ISL_629024, EPI_ISL_629025, EPI_ISL_629026, EPI_ISL_629027, EPI_ISL_629028			
see above	Centro de Biotecnología Vegetal, Universidad Andrés Bello, Center for Genome Regulation	Center for Mathematical Modeling and Center for Genome Regulation. Santiago, Chile	Bastias M, Sanhueza D, Travisany D, Allende ML, Maass A, González M, Bustos F, Arriagada G, Montecino, M, Orellana A, Castro E, Meneses C.
EPI_ISL_629029, EPI_ISL_629030	South Eastern Area Laboratory Services (SEALS)	NSW Health Pathology - Institute of Clinical Pathology and Medical Research; Westmead Hospital; University of Sydney	CIDM-PH et al.
EPI_ISL_629031, EPI_ISL_629032, EPI_ISL_629033, EPI_ISL_629034	Omsk Research Institute of Natural Focal Infections	WHO National Influenza Centre Russian Federation	Artem Fadeev, Ekaterina Gradoboeva, Ekaterina Savkina, Daria Nashatyreva, Elena Poleshchuk, Aleksei Vasilenko, Valery Yakimenko, Andrey Komissarov
EPI_ISL_629036, EPI_ISL_629037, EPI_ISL_629038, EPI_ISL_629039, EPI_ISL_629040, EPI_ISL_629041, EPI_ISL_629042, EPI_ISL_629043, EPI_ISL_629044, EPI_ISL_629045, EPI_ISL_629046, EPI_ISL_629047, EPI_ISL_629048, EPI_ISL_629049, EPI_ISL_629050, EPI_ISL_629051, EPI_ISL_629052, EPI_ISL_629053, EPI_ISL_629054, EPI_ISL_629055, EPI_ISL_629056, EPI_ISL_629057, EPI_ISL_629058, EPI_ISL_629059, EPI_ISL_629060, EPI_ISL_629061, EPI_ISL_629062, EPI_ISL_629063, EPI_ISL_629064, EPI_ISL_629065, EPI_ISL_629066, EPI_ISL_629067, EPI_ISL_629068, EPI_ISL_629069, EPI_ISL_629070, EPI_ISL_629071, EPI_ISL_629072, EPI_ISL_629073, EPI_ISL_629074, EPI_ISL_629075, EPI_ISL_629076, EPI_ISL_629077, EPI_ISL_629078, EPI_ISL_629079, EPI_ISL_629080			
see above	Maryland Public Health Laboratory	Maryland Public Health Laboratory	Maryland Department of Health Laboratories Administration
EPI_ISL_629081, EPI_ISL_629082, EPI_ISL_629083, EPI_ISL_629084, EPI_ISL_629085, EPI_ISL_629086, EPI_ISL_629088, EPI_ISL_629089, EPI_ISL_629090, EPI_ISL_629091, EPI_ISL_629092, EPI_ISL_629093, EPI_ISL_629094, EPI_ISL_629095, EPI_ISL_629096, EPI_ISL_629097, EPI_ISL_629098, EPI_ISL_629099, EPI_ISL_629101, EPI_ISL_629102, EPI_ISL_629103, EPI_ISL_629104, EPI_ISL_629105, EPI_ISL_629106, EPI_ISL_629107, EPI_ISL_629108			
see above	Laboratoire du Centre Hospitalier Annecy Genevois	CNR Virus des Infections Respiratoires - France SUD	Antonin Bal, Gregory Destras, Gwendolyne Burfin, Hadrien Règue, Quentin Semanas, Martine Valette, Bruno Lina, Hélène Petitprez, Bruno Chanzy, Laurence Josset
EPI_ISL_629109, EPI_ISL_629110, EPI_ISL_629111, EPI_ISL_629112, EPI_ISL_629113, EPI_ISL_629114, EPI_ISL_629115, EPI_ISL_629116, EPI_ISL_629117, EPI_ISL_629118, EPI_ISL_629119, EPI_ISL_629121, EPI_ISL_629122, EPI_ISL_629123, EPI_ISL_629124, EPI_ISL_629125, EPI_ISL_629126, EPI_ISL_629127, EPI_ISL_629128, EPI_ISL_629129, EPI_ISL_629130, EPI_ISL_629131, EPI_ISL_629132, EPI_ISL_629133, EPI_ISL_629134, EPI_ISL_629135, EPI_ISL_629136, EPI_ISL_629139, EPI_ISL_629140, EPI_ISL_629141, EPI_ISL_629142, EPI_ISL_629143, EPI_ISL_629144, EPI_ISL_629145, EPI_ISL_629146, EPI_ISL_629147, EPI_ISL_629148, EPI_ISL_629149, EPI_ISL_629150, EPI_ISL_629151, EPI_ISL_629152, EPI_ISL_629153, EPI_ISL_629154, EPI_ISL_629155, EPI_ISL_629156, EPI_ISL_629157, EPI_ISL_629158, EPI_ISL_629159, EPI_ISL_629160, EPI_ISL_629161, EPI_ISL_629162, EPI_ISL_629163, EPI_ISL_629164, EPI_ISL_629165, EPI_ISL_629166, EPI_ISL_629167, EPI_ISL_629168, EPI_ISL_629169, EPI_ISL_629170, EPI_ISL_629171, EPI_ISL_629172, EPI_ISL_629173, EPI_ISL_629174, EPI_ISL_629175, EPI_ISL_629176, EPI_ISL_629178, EPI_ISL_629179, EPI_ISL_629180, EPI_ISL_629181, EPI_ISL_629182, EPI_ISL_629183, EPI_ISL_629184, EPI_ISL_629185, EPI_ISL_629186, EPI_ISL_629187, EPI_ISL_629188, EPI_ISL_629189, EPI_ISL_629190, EPI_ISL_629191, EPI_ISL_629192, EPI_ISL_629193, EPI_ISL_629194, EPI_ISL_629195, EPI_ISL_629196, EPI_ISL_629197, EPI_ISL_629198, EPI_ISL_629200, EPI_ISL_629201, EPI_ISL_629202, EPI_ISL_629203, EPI_ISL_629204, EPI_ISL_629205, EPI_ISL_629206, EPI_ISL_629207, EPI_ISL_629208, EPI_ISL_629210, EPI_ISL_629211, EPI_ISL_629212, EPI_ISL_629213, EPI_ISL_629214, EPI_ISL_629215, EPI_ISL_629216, EPI_ISL_629218, EPI_ISL_629220, EPI_ISL_629221, EPI_ISL_629222, EPI_ISL_629223, EPI_ISL_629224, EPI_ISL_629225, EPI_ISL_629226, EPI_ISL_629227, EPI_ISL_629228, EPI_ISL_629230, EPI_ISL_629231, EPI_ISL_629232, EPI_ISL_629233, EPI_ISL_629234, EPI_ISL_629235, EPI_ISL_629236, EPI_ISL_629237, EPI_ISL_629238, EPI_ISL_629239, EPI_ISL_629240, EPI_ISL_629242, EPI_ISL_629243, EPI_ISL_629244, EPI_ISL_629246, EPI_ISL_629247, EPI_ISL_629248, EPI_ISL_629249, EPI_ISL_629250, EPI_ISL_629251, EPI_ISL_629253, EPI_ISL_629254, EPI_ISL_629255, EPI_ISL_629256, EPI_ISL_629257, EPI_ISL_629258, EPI_ISL_629259, EPI_ISL_629260, EPI_ISL_629261, EPI_ISL_629262, EPI_ISL_629263, EPI_ISL_629264, EPI_ISL_629265, EPI_ISL_629266, EPI_ISL_629267, EPI_ISL_629268, EPI_ISL_629269, EPI_ISL_629270, EPI_ISL_629271, EPI_ISL_629273, EPI_ISL_629274, EPI_ISL_629275, EPI_ISL_629278, EPI_ISL_629279, EPI_ISL_629280, EPI_ISL_629281, EPI_ISL_629282, EPI_ISL_629283, EPI_ISL_629284, EPI_ISL_629285, EPI_ISL_629286, EPI_ISL_629287, EPI_ISL_629288, EPI_ISL_629289, EPI_ISL_629290, EPI_ISL_629291, EPI_ISL_629292, EPI_ISL_629293, EPI_ISL_629294, EPI_ISL_629295, EPI_ISL_629296, EPI_ISL_629297, EPI_ISL_629298, EPI_ISL_629299, EPI_ISL_629300, EPI_ISL_629301, EPI_ISL_629302, EPI_ISL_629303, EPI_ISL_629304, EPI_ISL_629306, EPI_ISL_629307, EPI_ISL_629308, EPI_ISL_629310, EPI_ISL_629312, EPI_ISL_629313, EPI_ISL_629314, EPI_ISL_629315, EPI_ISL_629316, EPI_ISL_629317, EPI_ISL_629318, EPI_ISL_629319, EPI_ISL_629321, EPI_ISL_629322, EPI_ISL_629324, EPI_ISL_629325, EPI_ISL_629326, EPI_ISL_629327, EPI_ISL_629328, EPI_ISL_629329, EPI_ISL_629330, EPI_ISL_629332, EPI_ISL_629333, EPI_ISL_629334, EPI_ISL_629335, EPI_ISL_629336, EPI_ISL_629338, EPI_ISL_629339, EPI_ISL_629340, EPI_ISL_629341, EPI_ISL_629342, EPI_ISL_629343, EPI_ISL_629344, EPI_ISL_629345, EPI_ISL_629346, EPI_ISL_629348, EPI_ISL_629349, EPI_ISL_629350, EPI_ISL_629351, EPI_ISL_629352, EPI_ISL_629353, EPI_ISL_629354, EPI_ISL_629355, EPI_ISL_629356, EPI_ISL_629357, EPI_ISL_629358, EPI_ISL_629359, EPI_ISL_629360, EPI_ISL_629361, EPI_ISL_629362, EPI_ISL_629364, EPI_ISL_629365, EPI_ISL_629366, EPI_ISL_629367, EPI_ISL_629368, EPI_ISL_629369, EPI_ISL_629370, EPI_ISL_629371, EPI_ISL_629372, EPI_ISL_629373, EPI_ISL_629374, EPI_ISL_629375, EPI_ISL_629376, EPI_ISL_629377, EPI_ISL_629378, EPI_ISL_629379, EPI_ISL_629380, EPI_ISL_629381, EPI_ISL_629382, EPI_ISL_629383, EPI_ISL_629384, EPI_ISL_629385, EPI_ISL_629386, EPI_ISL_629387, EPI_ISL_629388, EPI_ISL_629389, EPI_ISL_629390, EPI_ISL_629391, EPI_ISL_629392, EPI_ISL_629393, EPI_ISL_629394, EPI_ISL_629395, EPI_ISL_629396, EPI_ISL_629397, EPI_ISL_629399, EPI_ISL_629400, EPI_ISL_629401, EPI_ISL_629402, EPI_ISL_629405, EPI_ISL_629406, EPI_ISL_629407, EPI_ISL_629410, EPI_ISL_629411, EPI_ISL_629412, EPI_ISL_629413, EPI_ISL_629414, EPI_ISL_629415, EPI_ISL_629416, EPI_ISL_629417, EPI_ISL_629418, EPI_ISL_629419, EPI_ISL_629420, EPI_ISL_629421, EPI_ISL_629422, EPI_ISL_629423, EPI_ISL_629424, EPI_ISL_629425, EPI_ISL_629426, EPI_ISL_629427, EPI_ISL_629429, EPI_ISL_629430, EPI_ISL_629431, EPI_ISL_629432, EPI_ISL_629433, EPI_ISL_629434, EPI_ISL_629435, EPI_ISL_629436, EPI_ISL_629437, EPI_ISL_629439, EPI_ISL_629441, EPI_ISL_629442, EPI_ISL_629443, EPI_ISL_629444, EPI_ISL_629445, EPI_ISL_629447, EPI_ISL_629448, EPI_ISL_629449, EPI_ISL_629451, EPI_ISL_629452, EPI_ISL_629453, EPI_ISL_629455, EPI_ISL_629456, EPI_ISL_629457, EPI_ISL_629458, EPI_ISL_629460, EPI_ISL_629461, EPI_ISL_629463, EPI_ISL_629464, EPI_ISL_629465, EPI_ISL_629467, EPI_ISL_629468, EPI_ISL_629469, EPI_ISL_629470,			

EPI_ISL_629471, EPI_ISL_629472, EPI_ISL_629473, EPI_ISL_629474, EPI_ISL_629475, EPI_ISL_629477, EPI_ISL_629480, EPI_ISL_629481, EPI_ISL_629482, EPI_ISL_629483, EPI_ISL_629485, EPI_ISL_629486, EPI_ISL_629487, EPI_ISL_629488, EPI_ISL_629489, EPI_ISL_629491, EPI_ISL_629492, EPI_ISL_629493, EPI_ISL_629494, EPI_ISL_629496, EPI_ISL_629497, EPI_ISL_629498, EPI_ISL_629500, EPI_ISL_629501, EPI_ISL_629502, EPI_ISL_629504, EPI_ISL_629505, EPI_ISL_629506, EPI_ISL_629507, EPI_ISL_629508, EPI_ISL_629510, EPI_ISL_629511, EPI_ISL_629513, EPI_ISL_629514, EPI_ISL_629515, EPI_ISL_629517, EPI_ISL_629518, EPI_ISL_629519, EPI_ISL_629521, EPI_ISL_629522, EPI_ISL_629523, EPI_ISL_629524, EPI_ISL_629525, EPI_ISL_629527, EPI_ISL_629529, EPI_ISL_629530, EPI_ISL_629531, EPI_ISL_629535, EPI_ISL_629536, EPI_ISL_629537, EPI_ISL_629538, EPI_ISL_629539, EPI_ISL_629540, EPI_ISL_629541, EPI_ISL_629542, EPI_ISL_629543, EPI_ISL_629545, EPI_ISL_629547, EPI_ISL_629550, EPI_ISL_629551, EPI_ISL_629552, EPI_ISL_629555, EPI_ISL_629556, EPI_ISL_629558, EPI_ISL_629560, EPI_ISL_629561, EPI_ISL_629564, EPI_ISL_629565, EPI_ISL_629566, EPI_ISL_629569, EPI_ISL_629571, EPI_ISL_629574, EPI_ISL_629575, EPI_ISL_629576, EPI_ISL_629577, EPI_ISL_629578, EPI_ISL_629579, EPI_ISL_629582, EPI_ISL_629584, EPI_ISL_629585, EPI_ISL_629586, EPI_ISL_629587, EPI_ISL_629588, EPI_ISL_629589, EPI_ISL_629594, EPI_ISL_629595, EPI_ISL_629596, EPI_ISL_629598, EPI_ISL_629601, EPI_ISL_629603, EPI_ISL_629604, EPI_ISL_629605, EPI_ISL_629606, EPI_ISL_629609, EPI_ISL_629610, EPI_ISL_629611, EPI_ISL_629612, EPI_ISL_629613, EPI_ISL_629614, EPI_ISL_629615, EPI_ISL_629616, EPI_ISL_629617, EPI_ISL_629618, EPI_ISL_629619, EPI_ISL_629621, EPI_ISL_629622, EPI_ISL_629623, EPI_ISL_629624, EPI_ISL_629625, EPI_ISL_629626, EPI_ISL_629627, EPI_ISL_629628, EPI_ISL_629629, EPI_ISL_629630, EPI_ISL_629631, EPI_ISL_629634, EPI_ISL_629635, EPI_ISL_629636, EPI_ISL_629637, EPI_ISL_629639, EPI_ISL_629640, EPI_ISL_629641, EPI_ISL_629642, EPI_ISL_629644, EPI_ISL_629646, EPI_ISL_629647, EPI_ISL_629648, EPI_ISL_629649, EPI_ISL_629650, EPI_ISL_629651, EPI_ISL_629652, EPI_ISL_629653, EPI_ISL_629655, EPI_ISL_629656, EPI_ISL_629657, EPI_ISL_629658, EPI_ISL_629659, EPI_ISL_629660, EPI_ISL_629661, EPI_ISL_629664, EPI_ISL_629667, EPI_ISL_629669, EPI_ISL_629670, EPI_ISL_629671, EPI_ISL_629673, EPI_ISL_629674, EPI_ISL_629675, EPI_ISL_629676, EPI_ISL_629677, EPI_ISL_629679, EPI_ISL_629680, EPI_ISL_629681, EPI_ISL_629682, EPI_ISL_629684, EPI_ISL_629685, EPI_ISL_629686, EPI_ISL_629687, EPI_ISL_629689, EPI_ISL_629691, EPI_ISL_629693, EPI_ISL_629694, EPI_ISL_629695, EPI_ISL_629696, EPI_ISL_629697, EPI_ISL_629698, EPI_ISL_629700, EPI_ISL_629704, EPI_ISL_629705, EPI_ISL_629707, EPI_ISL_629708, EPI_ISL_629709, EPI_ISL_629712, EPI_ISL_629713, EPI_ISL_629714, EPI_ISL_629715, EPI_ISL_629716, EPI_ISL_629717, EPI_ISL_629718, EPI_ISL_629719, EPI_ISL_629721, EPI_ISL_629722, EPI_ISL_629724, EPI_ISL_629726, EPI_ISL_629730, EPI_ISL_629731, EPI_ISL_629732, EPI_ISL_629734, EPI_ISL_629737, EPI_ISL_629738, EPI_ISL_629740, EPI_ISL_629743, EPI_ISL_629744, EPI_ISL_629745, EPI_ISL_629746, EPI_ISL_629748, EPI_ISL_629749, EPI_ISL_629750, EPI_ISL_629751, EPI_ISL_629752

see above	Lighthouse Lab in Milton Keynes	Wellcome Sanger Institute for the COVID-19 Genomics UK (COG-UK) consortium	The Lighthouse Lab in Milton Keynes and Alex Alderton, Roberto Amato, Sonia Goncalves, Ewan Harrison, David K. Jackson, Ian Johnston, Dominic Kwiatkowski, Cordelia Langford, John Sillitoe on behalf of the Wellcome Sanger Institute COVID-19 Surveillance Team

EPI_ISL_629755, EPI_ISL_629756, EPI_ISL_629757, EPI_ISL_629758, EPI_ISL_629759, EPI_ISL_629760, EPI_ISL_629761, EPI_ISL_629762, EPI_ISL_629763, EPI_ISL_629764, EPI_ISL_629765, EPI_ISL_629766, EPI_ISL_629767, EPI_ISL_629768, EPI_ISL_629769, EPI_ISL_629770, EPI_ISL_629771, EPI_ISL_629772, EPI_ISL_629773, EPI_ISL_629774, EPI_ISL_629775, EPI_ISL_629776, EPI_ISL_629777, EPI_ISL_629778, EPI_ISL_629779, EPI_ISL_629780, EPI_ISL_629781, EPI_ISL_629782, EPI_ISL_629785, EPI_ISL_629786, EPI_ISL_629787, EPI_ISL_629789, EPI_ISL_629790, EPI_ISL_629791, EPI_ISL_629792, EPI_ISL_629793, EPI_ISL_629794, EPI_ISL_629795, EPI_ISL_629796, EPI_ISL_629797, EPI_ISL_629798, EPI_ISL_629799, EPI_ISL_629800, EPI_ISL_629801, EPI_ISL_629802, EPI_ISL_629803, EPI_ISL_629804, EPI_ISL_629805, EPI_ISL_629806, EPI_ISL_629808, EPI_ISL_629809, EPI_ISL_629810, EPI_ISL_629811, EPI_ISL_629812, EPI_ISL_629813, EPI_ISL_629815, EPI_ISL_629816, EPI_ISL_629817, EPI_ISL_629818, EPI_ISL_629820, EPI_ISL_629821, EPI_ISL_629822, EPI_ISL_629824, EPI_ISL_629825, EPI_ISL_629826, EPI_ISL_629827, EPI_ISL_629828, EPI_ISL_629829, EPI_ISL_629830, EPI_ISL_629831, EPI_ISL_629832, EPI_ISL_629834, EPI_ISL_629835, EPI_ISL_629836, EPI_ISL_629837, EPI_ISL_629838, EPI_ISL_629840, EPI_ISL_629841, EPI_ISL_629842, EPI_ISL_629843, EPI_ISL_629844, EPI_ISL_629845, EPI_ISL_629846, EPI_ISL_629847, EPI_ISL_629848, EPI_ISL_629850, EPI_ISL_629851, EPI_ISL_629852, EPI_ISL_629853, EPI_ISL_629854, EPI_ISL_629855, EPI_ISL_629856, EPI_ISL_629857, EPI_ISL_629858, EPI_ISL_629859, EPI_ISL_629861, EPI_ISL_629862, EPI_ISL_629863, EPI_ISL_629864, EPI_ISL_629865, EPI_ISL_629867, EPI_ISL_629868, EPI_ISL_629869, EPI_ISL_629870, EPI_ISL_629871, EPI_ISL_629872, EPI_ISL_629873, EPI_ISL_629874, EPI_ISL_629875, EPI_ISL_629876, EPI_ISL_629878, EPI_ISL_629879, EPI_ISL_629880, EPI_ISL_629884, EPI_ISL_629885, EPI_ISL_629886, EPI_ISL_629889, EPI_ISL_629891, EPI_ISL_629892, EPI_ISL_629893, EPI_ISL_629894, EPI_ISL_629895, EPI_ISL_629896, EPI_ISL_629897, EPI_ISL_629898, EPI_ISL_629899, EPI_ISL_629900, EPI_ISL_629901, EPI_ISL_629903, EPI_ISL_629904, EPI_ISL_629905, EPI_ISL_629906, EPI_ISL_629908, EPI_ISL_629910, EPI_ISL_629911, EPI_ISL_629913, EPI_ISL_629915, EPI_ISL_629916, EPI_ISL_629917, EPI_ISL_629918, EPI_ISL_629919, EPI_ISL_629920, EPI_ISL_629921, EPI_ISL_629922, EPI_ISL_629923, EPI_ISL_629924, EPI_ISL_629925, EPI_ISL_629926, EPI_ISL_629927, EPI_ISL_629928, EPI_ISL_629929, EPI_ISL_629930, EPI_ISL_629931, EPI_ISL_629933, EPI_ISL_629934, EPI_ISL_629935, EPI_ISL_629936, EPI_ISL_629937, EPI_ISL_629938, EPI_ISL_629939, EPI_ISL_629941, EPI_ISL_629943, EPI_ISL_629944, EPI_ISL_629945, EPI_ISL_629946, EPI_ISL_629947, EPI_ISL_629948, EPI_ISL_629949, EPI_ISL_629950, EPI_ISL_629951, EPI_ISL_629952, EPI_ISL_629954, EPI_ISL_629955, EPI_ISL_629956, EPI_ISL_629957, EPI_ISL_629958, EPI_ISL_629959, EPI_ISL_629960, EPI_ISL_629961, EPI_ISL_629962, EPI_ISL_629963, EPI_ISL_629964, EPI_ISL_629965, EPI_ISL_629966, EPI_ISL_629967, EPI_ISL_629968, EPI_ISL_629969, EPI_ISL_629971, EPI_ISL_629972, EPI_ISL_629973, EPI_ISL_629975, EPI_ISL_629976, EPI_ISL_629979, EPI_ISL_629980, EPI_ISL_629981, EPI_ISL_629982, EPI_ISL_629983, EPI_ISL_629984, EPI_ISL_629985, EPI_ISL_629986, EPI_ISL_629988, EPI_ISL_629989, EPI_ISL_629990, EPI_ISL_629991, EPI_ISL_629992, EPI_ISL_629993, EPI_ISL_629995, EPI_ISL_629996, EPI_ISL_629997, EPI_ISL_629998, EPI_ISL_629999, EPI_ISL_630001, EPI_ISL_630002, EPI_ISL_630003, EPI_ISL_630004, EPI_ISL_630005, EPI_ISL_630006

see above	Lighthouse Lab in Glasgow	Wellcome Sanger Institute for the COVID-19 Genomics UK (COG-UK) consortium	Harper VanSteenhouse, Yumi Kasai, David Gray, Carol Clugston, Anna Dominiczak and Alex Alderton, Roberto Amato, Sonia Goncalves, Ewan Harrison, David K. Jackson, Ian Johnston, Dominic Kwiatkowski, Cordelia Langford, John Sillitoe on behalf of the Wellcome Sanger Institute COVID-19 Surveillance Team

EPI_ISL_630007, EPI_ISL_630008, EPI_ISL_630009, EPI_ISL_630011, EPI_ISL_630012, EPI_ISL_630013, EPI_ISL_630015, EPI_ISL_630016, EPI_ISL_630017, EPI_ISL_630018, EPI_ISL_630020, EPI_ISL_630021, EPI_ISL_630023, EPI_ISL_630024, EPI_ISL_630025, EPI_ISL_630027, EPI_ISL_630028, EPI_ISL_630029, EPI_ISL_630030, EPI_ISL_630031, EPI_ISL_630032, EPI_ISL_630033, EPI_ISL_630034, EPI_ISL_630035, EPI_ISL_630038, EPI_ISL_630039, EPI_ISL_630040, EPI_ISL_630041, EPI_ISL_630042, EPI_ISL_630043, EPI_ISL_630044, EPI_ISL_630045, EPI_ISL_630046, EPI_ISL_630047, EPI_ISL_630049, EPI_ISL_630050, EPI_ISL_630052, EPI_ISL_630053, EPI_ISL_630054, EPI_ISL_630055, EPI_ISL_630056, EPI_ISL_630057, EPI_ISL_630058, EPI_ISL_630059, EPI_ISL_630060, EPI_ISL_630061, EPI_ISL_630062, EPI_ISL_630063, EPI_ISL_630064, EPI_ISL_630065, EPI_ISL_630066, EPI_ISL_630067, EPI_ISL_630068, EPI_ISL_630069, EPI_ISL_630070, EPI_ISL_630071, EPI_ISL_630072, EPI_ISL_630073, EPI_ISL_630074, EPI_ISL_630075, EPI_ISL_630076, EPI_ISL_630077, EPI_ISL_630078, EPI_ISL_630079, EPI_ISL_630080, EPI_ISL_630081, EPI_ISL_630082, EPI_ISL_630083, EPI_ISL_630084, EPI_ISL_630085, EPI_ISL_630087, EPI_ISL_630088, EPI_ISL_630089, EPI_ISL_630090, EPI_ISL_630092, EPI_ISL_630093, EPI_ISL_630094, EPI_ISL_630096, EPI_ISL_630097, EPI_ISL_630098, EPI_ISL_630099, EPI_ISL_630100, EPI_ISL_630101, EPI_ISL_630102, EPI_ISL_630103, EPI_ISL_630104, EPI_ISL_630105, EPI_ISL_630106, EPI_ISL_630107, EPI_ISL_630108, EPI_ISL_630111, EPI_ISL_630112, EPI_ISL_630113, EPI_ISL_630115, EPI_ISL_630116, EPI_ISL_630117, EPI_ISL_630118, EPI_ISL_630119, EPI_ISL_630120, EPI_ISL_630121, EPI_ISL_630122, EPI_ISL_630123, EPI_ISL_630124, EPI_ISL_630125, EPI_ISL_630126, EPI_ISL_630127, EPI_ISL_630128, EPI_ISL_630130, EPI_ISL_630131, EPI_ISL_630132, EPI_ISL_630133, EPI_ISL_630134, EPI_ISL_630135, EPI_ISL_630136, EPI_ISL_630137, EPI_ISL_630138, EPI_ISL_630139, EPI_ISL_630140, EPI_ISL_630141, EPI_ISL_630142, EPI_ISL_630143, EPI_ISL_630144, EPI_ISL_630145, EPI_ISL_630146, EPI_ISL_630148, EPI_ISL_630149, EPI_ISL_630150, EPI_ISL_630152, EPI_ISL_630153, EPI_ISL_630154, EPI_ISL_630155, EPI_ISL_630156, EPI_ISL_630157, EPI_ISL_630158, EPI_ISL_630159, EPI_ISL_630160, EPI_ISL_630161, EPI_ISL_630163, EPI_ISL_630164, EPI_ISL_630165, EPI_ISL_630166, EPI_ISL_630168, EPI_ISL_630169, EPI_ISL_630171, EPI_ISL_630172, EPI_ISL_630173, EPI_ISL_630174, EPI_ISL_630175, EPI_ISL_630176, EPI_ISL_630177, EPI_ISL_630178, EPI_ISL_630180, EPI_ISL_630181, EPI_ISL_630182, EPI_ISL_630183, EPI_ISL_630184, EPI_ISL_630185, EPI_ISL_630186, EPI_ISL_630188, EPI_ISL_630190, EPI_ISL_630192, EPI_ISL_630193, EPI_ISL_630194, EPI_ISL_630196, EPI_ISL_630197, EPI_ISL_630198, EPI_ISL_630199, EPI_ISL_630200, EPI_ISL_630201, EPI_ISL_630202, EPI_ISL_630203, EPI_ISL_630204, EPI_ISL_630206, EPI_ISL_630207, EPI_ISL_630208, EPI_ISL_630209, EPI_ISL_630211, EPI_ISL_630212, EPI_ISL_630213, EPI_ISL_630214, EPI_ISL_630215, EPI_ISL_630216, EPI_ISL_630217, EPI_ISL_630218, EPI_ISL_630220, EPI_ISL_630221, EPI_ISL_630222, EPI_ISL_630223, EPI_ISL_630225, EPI_ISL_630226, EPI_ISL_630227, EPI_ISL_630228, EPI_ISL_630229, EPI_ISL_630230, EPI_ISL_630231, EPI_ISL_630233, EPI_ISL_630234, EPI_ISL_630236, EPI_ISL_630237, EPI_ISL_630240, EPI_ISL_630241, EPI_ISL_630242, EPI_ISL_630243, EPI_ISL_630246, EPI_ISL_630248, EPI_ISL_630250, EPI_ISL_630251, EPI_ISL_630253, EPI_ISL_630256, EPI_ISL_630257, EPI_ISL_630258, EPI_ISL_630259, EPI_ISL_630260, EPI_ISL_630262, EPI_ISL_630263, EPI_ISL_630264, EPI_ISL_630265, EPI_ISL_630267, EPI_ISL_630268, EPI_ISL_630269, EPI_ISL_630270, EPI_ISL_630271, EPI_ISL_630272, EPI_ISL_630273, EPI_ISL_630274, EPI_ISL_630275, EPI_ISL_630277, EPI_ISL_630278, EPI_ISL_630279, EPI_ISL_630280, EPI_ISL_630281, EPI_ISL_630282, EPI_ISL_630283, EPI_ISL_630284, EPI_ISL_630285, EPI_ISL_630286, EPI_ISL_630288, EPI_ISL_630289, EPI_ISL_630290, EPI_ISL_630291, EPI_ISL_630292, EPI_ISL_630293, EPI_ISL_630294, EPI_ISL_630295, EPI_ISL_630296, EPI_ISL_630297, EPI_ISL_630299, EPI_ISL_630300, EPI_ISL_630302, EPI_ISL_630303, EPI_ISL_630304, EPI_ISL_630305, EPI_ISL_630306, EPI_ISL_630307, EPI_ISL_630308, EPI_ISL_630309, EPI_ISL_630310, EPI_ISL_630311, EPI_ISL_630312, EPI_ISL_630313, EPI_ISL_630314, EPI_ISL_630315, EPI_ISL_630316, EPI_ISL_630317, EPI_ISL_630318, EPI_ISL_630320, EPI_ISL_630321, EPI_ISL_630322, EPI_ISL_630323, EPI_ISL_630324, EPI_ISL_630325, EPI_ISL_630326, EPI_ISL_630327, EPI_ISL_630328, EPI_ISL_630329, EPI_ISL_630330, EPI_ISL_630331, EPI_ISL_630333, EPI_ISL_630334, EPI_ISL_630335, EPI_ISL_630336, EPI_ISL_630337, EPI_ISL_630338, EPI_ISL_630339, EPI_ISL_630340, EPI_ISL_630341, EPI_ISL_630342, EPI_ISL_630344, EPI_ISL_630346, EPI_ISL_630347

see above	Lighthouse Lab in Milton Keynes	Wellcome Sanger Institute for the COVID-19 Genomics UK (COG-UK) consortium	The Lighthouse Lab in Milton Keynes and Alex Alderton, Roberto Amato, Sonia Goncalves, Ewan Harrison, David K. Jackson, Ian Johnston, Dominic Kwiatkowski, Cordelia Langford, John Sillitoe on behalf of the Wellcome Sanger Institute COVID-19 Surveillance Team

EPI_ISL_630348, EPI_ISL_630349, EPI_ISL_630350, EPI_ISL_630351, EPI_ISL_630352, EPI_ISL_630353, EPI_ISL_630354, EPI_ISL_630355, EPI_ISL_630356, EPI_ISL_630357, EPI_ISL_630358, EPI_ISL_630359, EPI_ISL_630360, EPI_ISL_630361, EPI_ISL_630362, EPI_ISL_630363, EPI_ISL_630364, EPI_ISL_630365, EPI_ISL_630366, EPI_ISL_630367, EPI_ISL_630368, EPI_ISL_630369, EPI_ISL_630370, EPI_ISL_630371, EPI_ISL_630372, EPI_ISL_630373, EPI_ISL_630374, EPI_ISL_630375, EPI_ISL_630376, EPI_ISL_630377, EPI_ISL_630378, EPI_ISL_630379, EPI_ISL_630380, EPI_ISL_630381, EPI_ISL_630382, EPI_ISL_630383, EPI_ISL_630384, EPI_ISL_630385, EPI_ISL_630386, EPI_ISL_630387, EPI_ISL_630388, EPI_ISL_630389, EPI_ISL_630390, EPI_ISL_630391, EPI_ISL_630392, EPI_ISL_630393, EPI_ISL_630394, EPI_ISL_630395, EPI_ISL_630396, EPI_ISL_630398, EPI_ISL_630399, EPI_ISL_630400, EPI_ISL_630401, EPI_ISL_630402, EPI_ISL_630403, EPI_ISL_630404, EPI_ISL_630405, EPI_ISL_630406, EPI_ISL_630407, EPI_ISL_630408, EPI_ISL_630409, EPI_ISL_630410, EPI_ISL_630411, EPI_ISL_630412, EPI_ISL_630413, EPI_ISL_630414, EPI_ISL_630415, EPI_ISL_630416, EPI_ISL_630417, EPI_ISL_630418, EPI_ISL_630419, EPI_ISL_630421, EPI_ISL_630422, EPI_ISL_630423, EPI_ISL_630424, EPI_ISL_630425, EPI_ISL_630426, EPI_ISL_630427, EPI_ISL_630428, EPI_ISL_630429, EPI_ISL_630430, EPI_ISL_630431, EPI_ISL_630432, EPI_ISL_630433, EPI_ISL_630434, EPI_ISL_630436, EPI_ISL_630437, EPI_ISL_630438, EPI_ISL_630439, EPI_ISL_630440, EPI_ISL_630441, EPI_ISL_630442, EPI_ISL_630444, EPI_ISL_630445, EPI_ISL_630446, EPI_ISL_630447, EPI_ISL_630448, EPI_ISL_630449, EPI_ISL_630450, EPI_ISL_630451, EPI_ISL_630452, EPI_ISL_630453, EPI_ISL_630454, EPI_ISL_630456, EPI_ISL_630457, EPI_ISL_630458, EPI_ISL_630459, EPI_ISL_630460, EPI_ISL_630461, EPI_ISL_630462, EPI_ISL_630463, EPI_ISL_630464, EPI_ISL_630465, EPI_ISL_630466, EPI_ISL_630467, EPI_ISL_630468, EPI_ISL_630469, EPI_ISL_630470, EPI_ISL_630471, EPI_ISL_630472, EPI_ISL_630473, EPI_ISL_630475, EPI_ISL_630476, EPI_ISL_630477, EPI_ISL_630478, EPI_ISL_630479, EPI_ISL_630480, EPI_ISL_630481, EPI_ISL_630482, EPI_ISL_630484, EPI_ISL_630485, EPI_ISL_630486, EPI_ISL_630487, EPI_ISL_630488, EPI_ISL_630490, EPI_ISL_630491, EPI_ISL_630492, EPI_ISL_630493, EPI_ISL_630494, EPI_ISL_630495, EPI_ISL_630496, EPI_ISL_630497, EPI_ISL_630498, EPI_ISL_630499, EPI_ISL_630500, EPI_ISL_630501, EPI_ISL_630502, EPI_ISL_630503, EPI_ISL_630504, EPI_ISL_630505, EPI_ISL_630506, EPI_ISL_630507, EPI_ISL_630509, EPI_ISL_630510, EPI_ISL_630511, EPI_ISL_630512, EPI_ISL_630514, EPI_ISL_630515, EPI_ISL_630516, EPI_ISL_630517, EPI_ISL_630518, EPI_ISL_630519, EPI_ISL_630520, EPI_ISL_630521, EPI_ISL_630522, EPI_ISL_630523, EPI_ISL_630524, EPI_ISL_630525, EPI_ISL_630526, EPI_ISL_630528, EPI_ISL_630530, EPI_ISL_630531, EPI_ISL_630532, EPI_ISL_630534, EPI_ISL_630535, EPI_ISL_630538, EPI_ISL_630539, EPI_ISL_630541, EPI_ISL_630542, EPI_ISL_630543, EPI_ISL_630544, EPI_ISL_630548, EPI_ISL_630549, EPI_ISL_630550, EPI_ISL_630551, EPI_ISL_630552, EPI_ISL_630553, EPI_ISL_630554, EPI_ISL_630555, EPI_ISL_630556, EPI_ISL_630557, EPI_ISL_630558, EPI_ISL_630559, EPI_ISL_630560, EPI_ISL_630561, EPI_ISL_630562, EPI_ISL_630563, EPI_ISL_630564, EPI_ISL_630566, EPI_ISL_630567, EPI_ISL_630568, EPI_ISL_630569, EPI_ISL_630570, EPI_ISL_630571, EPI_ISL_630572, EPI_ISL_630573, EPI_ISL_630574, EPI_ISL_630575, EPI_ISL_630577, EPI_ISL_630578, EPI_ISL_630579, EPI_ISL_630581, EPI_ISL_630582, EPI_ISL_630583, EPI_ISL_630584, EPI_ISL_630585, EPI_ISL_630586, EPI_ISL_630587, EPI_ISL_630588, EPI_ISL_630589, EPI_ISL_630590, EPI_ISL_630591, EPI_ISL_630592, EPI_ISL_630593, EPI_ISL_630594, EPI_ISL_630595, EPI_ISL_630596, EPI_ISL_630597, EPI_ISL_630598, EPI_ISL_630599, EPI_ISL_630601, EPI_ISL_630602, EPI_ISL_630603, EPI_ISL_630604, EPI_ISL_630605, EPI_ISL_630606, EPI_ISL_630607, EPI_ISL_630608, EPI_ISL_630610, EPI_ISL_630611, EPI_ISL_630612, EPI_ISL_630613, EPI_ISL_630614, EPI_ISL_630615, EPI_ISL_630616, EPI_ISL_630617, EPI_ISL_630618, EPI_ISL_630619, EPI_ISL_630620, EPI_ISL_630622, EPI_ISL_630623, EPI_ISL_630624, EPI_ISL_630625, EPI_ISL_630626, EPI_ISL_630627, EPI_ISL_630628, EPI_ISL_630629, EPI_ISL_630630, EPI_ISL_630631, EPI_ISL_630633, EPI_ISL_630635, EPI_ISL_630636, EPI_ISL_630637, EPI_ISL_630638, EPI_ISL_630639, EPI_ISL_630640, EPI_ISL_630641, EPI_ISL_630642, EPI_ISL_630643, EPI_ISL_630644, EPI_ISL_630645, EPI_ISL_630646, EPI_ISL_630647, EPI_ISL_630648, EPI_ISL_630649, EPI_ISL_630651, EPI_ISL_630652, EPI_ISL_630653, EPI_ISL_630654, EPI_ISL_630655, EPI_ISL_630656, EPI_ISL_630658, EPI_ISL_630659, EPI_ISL_630660, EPI_ISL_630661, EPI_ISL_630662, EPI_ISL_630663, EPI_ISL_630664, EPI_ISL_630665, EPI_ISL_630667, EPI_ISL_630668, EPI_ISL_630669, EPI_ISL_630670, EPI_ISL_630671, EPI_ISL_630672, EPI_ISL_630673, EPI_ISL_630674, EPI_ISL_630676, EPI_ISL_630678, EPI_ISL_630679, EPI_ISL_630680, EPI_ISL_630681, EPI_ISL_630682, EPI_ISL_630683, EPI_ISL_630684, EPI_ISL_630686, EPI_ISL_630687, EPI_ISL_630688, EPI_ISL_630689, EPI_ISL_630690, EPI_ISL_630691, EPI_ISL_630693, EPI_ISL_630694, EPI_ISL_630695, EPI_ISL_630696, EPI_ISL_630697, EPI_ISL_630698, EPI_ISL_630699, EPI_ISL_630700, EPI_ISL_630701

see above	Lighthouse Lab in Alderley Park	Wellcome Sanger Institute for the COVID-19 Genomics UK (COG-UK) consortium	Jacquelyn Wynn, Mairead Hyland, The Lighthouse Lab in Alderley Park and Alex Alderton, Roberto Amato, Sonia Goncalves, Ewan Harrison, David K. Jackson, Ian Johnston, Dominic Kwiatkowski, Cordelia Langford, John Sillitoe on behalf of the Wellcome Sanger Institute COVID-19 Surveillance Team
EPI_ISL_630702, EPI_ISL_630703, EPI_ISL_630704, EPI_ISL_630705	Lighthouse Lab in Glasgow	Wellcome Sanger Institute for the COVID-19 Genomics UK (COG-UK) consortium	Harper VanSteenhouse, Yumi Kasai, David Gray, Carol Clugston, Anna Dominiczak and Alex Alderton, Roberto Amato, Sonia Goncalves, Ewan Harrison, David K. Jackson, Ian Johnston, Dominic Kwiatkowski, Cordelia Langford, John Sillitoe on behalf of the Wellcome Sanger Institute COVID-19 Surveillance Team
EPI_ISL_630707, EPI_ISL_630708, EPI_ISL_630709, EPI_ISL_630713, EPI_ISL_630714, EPI_ISL_630715, EPI_ISL_630716, EPI_ISL_630717	Lighthouse Lab in Alderley Park	Wellcome Sanger Institute for the COVID-19 Genomics UK (COG-UK) consortium	Jacquelyn Wynn, Mairead Hyland, The Lighthouse Lab in Alderley Park and Alex Alderton, Roberto Amato, Sonia Goncalves, Ewan Harrison, David K. Jackson, Ian Johnston, Dominic Kwiatkowski, Cordelia Langford, John Sillitoe on behalf of the Wellcome Sanger Institute COVID-19 Surveillance Team
EPI_ISL_630718, EPI_ISL_630719	Lighthouse Lab in Glasgow	Wellcome Sanger Institute for the COVID-19 Genomics UK (COG-UK) consortium	Harper VanSteenhouse, Yumi Kasai, David Gray, Carol Clugston, Anna Dominiczak and Alex Alderton, Roberto Amato, Sonia Goncalves, Ewan Harrison, David K. Jackson, Ian Johnston, Dominic Kwiatkowski, Cordelia Langford, John Sillitoe on behalf of the Wellcome Sanger Institute COVID-19 Surveillance Team
EPI_ISL_630723, EPI_ISL_630724	Lighthouse Lab in Alderley Park	Wellcome Sanger Institute for the COVID-19 Genomics UK (COG-UK) consortium	Jacquelyn Wynn, Mairead Hyland, The Lighthouse Lab in Alderley Park and Alex Alderton, Roberto Amato, Sonia Goncalves, Ewan Harrison, David K. Jackson, Ian Johnston, Dominic Kwiatkowski, Cordelia Langford, John Sillitoe on behalf of the Wellcome Sanger Institute COVID-19 Surveillance Team
EPI_ISL_630725	Lighthouse Lab in Glasgow	Wellcome Sanger Institute for the COVID-19 Genomics	Harper VanSteenhouse, Yumi Kasai, David Gray, Carol Clugston, Anna Dominiczak and Alex Alderton, Roberto Amato, Sonia Goncalves, Ewan Harrison,

		UK (COG-UK) consortium	David K. Jackson, Ian Johnston, Dominic Kwiatkowski, Cordelia Langford, John Sillitoe on behalf of the Wellcome Sanger Institute COVID-19 Surveillance Team
EPI_ISL_630726, EPI_ISL_630727, EPI_ISL_630728, EPI_ISL_630729, EPI_ISL_630732	Lighthouse Lab in Alderley Park	Wellcome Sanger Institute for the COVID-19 Genomics UK (COG-UK) consortium	Jacquelyn Wynn, Mairead Hyland, The Lighthouse Lab in Alderley Park and Alex Alderton, Roberto Amato, Sonia Goncalves, Ewan Harrison, David K. Jackson, Ian Johnston, Dominic Kwiatkowski, Cordelia Langford, John Sillitoe on behalf of the Wellcome Sanger Institute COVID-19 Surveillance Team
EPI_ISL_630733	Lighthouse Lab in Glasgow	Wellcome Sanger Institute for the COVID-19 Genomics UK (COG-UK) consortium	Harper VanSteenhouse, Yumi Kasai, David Gray, Carol Clugston, Anna Dominiczak and Alex Alderton, Roberto Amato, Sonia Goncalves, Ewan Harrison, David K. Jackson, Ian Johnston, Dominic Kwiatkowski, Cordelia Langford, John Sillitoe on behalf of the Wellcome Sanger Institute COVID-19 Surveillance Team
EPI_ISL_630735, EPI_ISL_630736, EPI_ISL_630737, EPI_ISL_630739, EPI_ISL_630740, EPI_ISL_630743, EPI_ISL_630744, EPI_ISL_630746, EPI_ISL_630747, EPI_ISL_630748, EPI_ISL_630750, EPI_ISL_630751, EPI_ISL_630752, EPI_ISL_630753, EPI_ISL_630754, EPI_ISL_630755, EPI_ISL_630756, EPI_ISL_630757, EPI_ISL_630758, EPI_ISL_630759, EPI_ISL_630760			
see above	Lighthouse Lab in Alderley Park	Wellcome Sanger Institute for the COVID-19 Genomics UK (COG-UK) consortium	Jacquelyn Wynn, Mairead Hyland, The Lighthouse Lab in Alderley Park and Alex Alderton, Roberto Amato, Sonia Goncalves, Ewan Harrison, David K. Jackson, Ian Johnston, Dominic Kwiatkowski, Cordelia Langford, John Sillitoe on behalf of the Wellcome Sanger Institute COVID-19 Surveillance Team
EPI_ISL_630761	Lighthouse Lab in Glasgow	Wellcome Sanger Institute for the COVID-19 Genomics UK (COG-UK) consortium	Harper VanSteenhouse, Yumi Kasai, David Gray, Carol Clugston, Anna Dominiczak and Alex Alderton, Roberto Amato, Sonia Goncalves, Ewan Harrison, David K. Jackson, Ian Johnston, Dominic Kwiatkowski, Cordelia Langford, John Sillitoe on behalf of the Wellcome Sanger Institute COVID-19 Surveillance Team
EPI_ISL_630763	Lighthouse Lab in Alderley Park	Wellcome Sanger Institute for the COVID-19 Genomics UK (COG-UK) consortium	Jacquelyn Wynn, Mairead Hyland, The Lighthouse Lab in Alderley Park and Alex Alderton, Roberto Amato, Sonia Goncalves, Ewan Harrison, David K. Jackson, Ian Johnston, Dominic Kwiatkowski, Cordelia Langford, John Sillitoe on behalf of the Wellcome Sanger Institute COVID-19 Surveillance Team
EPI_ISL_630764	Lighthouse Lab in Glasgow	Wellcome Sanger Institute for the COVID-19 Genomics UK (COG-UK) consortium	Harper VanSteenhouse, Yumi Kasai, David Gray, Carol Clugston, Anna Dominiczak and Alex Alderton, Roberto Amato, Sonia Goncalves, Ewan Harrison, David K. Jackson, Ian Johnston, Dominic Kwiatkowski, Cordelia Langford, John Sillitoe on behalf of the Wellcome Sanger Institute COVID-19 Surveillance Team
EPI_ISL_630766, EPI_ISL_630767, EPI_ISL_630768, EPI_ISL_630769, EPI_ISL_630772, EPI_ISL_630773, EPI_ISL_630774	Lighthouse Lab in Alderley Park	Wellcome Sanger Institute for the COVID-19 Genomics UK (COG-UK) consortium	Jacquelyn Wynn, Mairead Hyland, The Lighthouse Lab in Alderley Park and Alex Alderton, Roberto Amato, Sonia Goncalves, Ewan Harrison, David K. Jackson, Ian Johnston, Dominic Kwiatkowski, Cordelia Langford, John Sillitoe on behalf of the Wellcome Sanger Institute COVID-19 Surveillance Team
EPI_ISL_630775	Lighthouse Lab in Glasgow	Wellcome Sanger Institute for the COVID-19 Genomics UK (COG-UK) consortium	Harper VanSteenhouse, Yumi Kasai, David Gray, Carol Clugston, Anna Dominiczak and Alex Alderton, Roberto Amato, Sonia Goncalves, Ewan Harrison, David K. Jackson, Ian Johnston, Dominic Kwiatkowski, Cordelia Langford, John Sillitoe on behalf of the Wellcome Sanger Institute COVID-19 Surveillance Team
EPI_ISL_630776, EPI_ISL_630777	Lighthouse Lab in Alderley Park	Wellcome Sanger Institute for the COVID-19 Genomics UK (COG-UK) consortium	Jacquelyn Wynn, Mairead Hyland, The Lighthouse Lab in Alderley Park and Alex Alderton, Roberto Amato, Sonia Goncalves, Ewan Harrison, David K. Jackson, Ian Johnston, Dominic Kwiatkowski, Cordelia Langford, John Sillitoe on behalf of the Wellcome Sanger Institute COVID-19 Surveillance Team
EPI_ISL_630778, EPI_ISL_630782	Lighthouse Lab in Glasgow	Wellcome Sanger Institute for the COVID-19 Genomics UK (COG-UK) consortium	Harper VanSteenhouse, Yumi Kasai, David Gray, Carol Clugston, Anna Dominiczak and Alex Alderton, Roberto Amato, Sonia Goncalves, Ewan Harrison, David K. Jackson, Ian Johnston, Dominic Kwiatkowski, Cordelia Langford, John Sillitoe on behalf of the Wellcome Sanger Institute COVID-19 Surveillance Team
EPI_ISL_630783, EPI_ISL_630784, EPI_ISL_630785	Lighthouse Lab in Alderley Park	Wellcome Sanger Institute for the COVID-19 Genomics UK (COG-UK) consortium	Jacquelyn Wynn, Mairead Hyland, The Lighthouse Lab in Alderley Park and Alex Alderton, Roberto Amato, Sonia Goncalves, Ewan Harrison, David K. Jackson, Ian Johnston, Dominic Kwiatkowski, Cordelia Langford, John Sillitoe on behalf of the Wellcome Sanger Institute COVID-19 Surveillance Team
EPI_ISL_630787	Lighthouse Lab in Glasgow	Wellcome Sanger Institute for the COVID-19 Genomics UK (COG-UK) consortium	Harper VanSteenhouse, Yumi Kasai, David Gray, Carol Clugston, Anna Dominiczak and Alex Alderton, Roberto Amato, Sonia Goncalves, Ewan Harrison, David K. Jackson, Ian Johnston, Dominic Kwiatkowski, Cordelia Langford, John Sillitoe on behalf of the Wellcome Sanger Institute COVID-19 Surveillance Team
EPI_ISL_630788, EPI_ISL_630790, EPI_ISL_630792, EPI_ISL_630793, EPI_ISL_630795, EPI_ISL_630796, EPI_ISL_630797, EPI_ISL_630798, EPI_ISL_630799, EPI_ISL_630800, EPI_ISL_630801, EPI_ISL_630802, EPI_ISL_630803			
see above	Lighthouse Lab in Alderley Park	Wellcome Sanger Institute for the COVID-19 Genomics UK (COG-UK) consortium	Jacquelyn Wynn, Mairead Hyland, The Lighthouse Lab in Alderley Park and Alex Alderton, Roberto Amato, Sonia Goncalves, Ewan Harrison, David K. Jackson, Ian Johnston, Dominic Kwiatkowski, Cordelia Langford, John Sillitoe on behalf of the Wellcome Sanger Institute COVID-19 Surveillance Team
EPI_ISL_630804	Lighthouse Lab in Glasgow	Wellcome Sanger Institute for the COVID-19 Genomics UK (COG-UK) consortium	Harper VanSteenhouse, Yumi Kasai, David Gray, Carol Clugston, Anna Dominiczak and Alex Alderton, Roberto Amato, Sonia Goncalves, Ewan Harrison, David K. Jackson, Ian Johnston, Dominic Kwiatkowski, Cordelia Langford, John Sillitoe on behalf of the Wellcome Sanger Institute COVID-19 Surveillance Team
EPI_ISL_630805, EPI_ISL_630807, EPI_ISL_630808, EPI_ISL_630809, EPI_ISL_630810, EPI_ISL_630811, EPI_ISL_630812	Lighthouse Lab in Alderley Park	Wellcome Sanger Institute for the COVID-19 Genomics UK (COG-UK) consortium	Jacquelyn Wynn, Mairead Hyland, The Lighthouse Lab in Alderley Park and Alex Alderton, Roberto Amato, Sonia Goncalves, Ewan Harrison, David K. Jackson, Ian Johnston, Dominic Kwiatkowski, Cordelia Langford, John Sillitoe on behalf of the Wellcome Sanger Institute COVID-19 Surveillance Team
EPI_ISL_630813	Lighthouse Lab in Glasgow	Wellcome Sanger Institute for the COVID-19 Genomics UK (COG-UK) consortium	Harper VanSteenhouse, Yumi Kasai, David Gray, Carol Clugston, Anna Dominiczak and Alex Alderton, Roberto Amato, Sonia Goncalves, Ewan Harrison, David K. Jackson, Ian Johnston, Dominic Kwiatkowski, Cordelia Langford, John Sillitoe on behalf of the Wellcome Sanger Institute COVID-19 Surveillance Team
EPI_ISL_630814	Lighthouse Lab in Alderley Park	Wellcome Sanger Institute for the COVID-19 Genomics UK (COG-UK) consortium	Jacquelyn Wynn, Mairead Hyland, The Lighthouse Lab in Alderley Park and Alex Alderton, Roberto Amato, Sonia Goncalves, Ewan Harrison, David K. Jackson, Ian Johnston, Dominic Kwiatkowski, Cordelia Langford, John Sillitoe on behalf of the Wellcome Sanger Institute COVID-19 Surveillance Team
EPI_ISL_630815	Lighthouse Lab in Glasgow	Wellcome Sanger Institute for the COVID-19 Genomics UK (COG-UK) consortium	Harper VanSteenhouse, Yumi Kasai, David Gray, Carol Clugston, Anna Dominiczak and Alex Alderton, Roberto Amato, Sonia Goncalves, Ewan Harrison, David K. Jackson, Ian Johnston, Dominic Kwiatkowski, Cordelia Langford, John Sillitoe on behalf of the Wellcome Sanger Institute COVID-19 Surveillance Team
EPI_ISL_630816, EPI_ISL_630817, EPI_ISL_630818, EPI_ISL_630819, EPI_ISL_630820, EPI_ISL_630821, EPI_ISL_630822, EPI_ISL_630823	Lighthouse Lab in Alderley Park	Wellcome Sanger Institute for the COVID-19 Genomics UK (COG-UK) consortium	Jacquelyn Wynn, Mairead Hyland, The Lighthouse Lab in Alderley Park and Alex Alderton, Roberto Amato, Sonia Goncalves, Ewan Harrison, David K. Jackson, Ian Johnston, Dominic Kwiatkowski, Cordelia Langford, John Sillitoe on behalf of the Wellcome Sanger Institute COVID-19 Surveillance Team
EPI_ISL_630824	Lighthouse Lab in Glasgow	Wellcome Sanger Institute for the COVID-19 Genomics UK (COG-UK) consortium	Harper VanSteenhouse, Yumi Kasai, David Gray, Carol Clugston, Anna Dominiczak and Alex Alderton, Roberto Amato, Sonia Goncalves, Ewan Harrison, David K. Jackson, Ian Johnston, Dominic Kwiatkowski, Cordelia Langford, John Sillitoe on behalf of the Wellcome Sanger Institute COVID-19 Surveillance Team
EPI_ISL_630825, EPI_ISL_630826	Lighthouse Lab in Alderley Park	Wellcome Sanger Institute for the COVID-19 Genomics UK (COG-UK) consortium	Jacquelyn Wynn, Mairead Hyland, The Lighthouse Lab in Alderley Park and Alex Alderton, Roberto Amato, Sonia Goncalves, Ewan Harrison, David K. Jackson, Ian Johnston, Dominic Kwiatkowski, Cordelia Langford, John Sillitoe on behalf of the Wellcome Sanger Institute COVID-19 Surveillance Team
EPI_ISL_630827	Lighthouse Lab in Glasgow	Wellcome Sanger Institute for the COVID-19 Genomics UK (COG-UK) consortium	Harper VanSteenhouse, Yumi Kasai, David Gray, Carol Clugston, Anna Dominiczak and Alex Alderton, Roberto Amato, Sonia Goncalves, Ewan Harrison, David K. Jackson, Ian Johnston, Dominic Kwiatkowski, Cordelia Langford, John Sillitoe on behalf of the Wellcome Sanger Institute COVID-19 Surveillance Team
EPI_ISL_630828, EPI_ISL_630829, EPI_ISL_630830, EPI_ISL_630831, EPI_ISL_630832	Lighthouse Lab in Alderley Park	Wellcome Sanger Institute for the COVID-19 Genomics UK (COG-UK) consortium	Jacquelyn Wynn, Mairead Hyland, The Lighthouse Lab in Alderley Park and Alex Alderton, Roberto Amato, Sonia Goncalves, Ewan Harrison, David K. Jackson, Ian Johnston, Dominic Kwiatkowski, Cordelia Langford, John Sillitoe on behalf of the Wellcome Sanger Institute COVID-19 Surveillance Team
EPI_ISL_630833	Lighthouse Lab in Glasgow	Wellcome Sanger Institute for the COVID-19 Genomics UK (COG-UK) consortium	Harper VanSteenhouse, Yumi Kasai, David Gray, Carol Clugston, Anna Dominiczak and Alex Alderton, Roberto Amato, Sonia Goncalves, Ewan Harrison, David K. Jackson, Ian Johnston, Dominic Kwiatkowski, Cordelia Langford, John Sillitoe on behalf of the Wellcome Sanger Institute COVID-19 Surveillance Team
EPI_ISL_630835, EPI_ISL_630836, EPI_ISL_630837, EPI_ISL_630840, EPI_ISL_630841, EPI_ISL_630842, EPI_ISL_630843, EPI_ISL_630844, EPI_ISL_630845, EPI_ISL_630847, EPI_ISL_630848			
see above	Lighthouse Lab in Alderley Park	Wellcome Sanger Institute for the COVID-19 Genomics UK (COG-UK) consortium	Jacquelyn Wynn, Mairead Hyland, The Lighthouse Lab in Alderley Park and Alex Alderton, Roberto Amato, Sonia Goncalves, Ewan Harrison, David K. Jackson, Ian Johnston, Dominic Kwiatkowski, Cordelia Langford, John Sillitoe on behalf of the Wellcome Sanger Institute COVID-19 Surveillance Team
EPI_ISL_630851	Lighthouse Lab in Glasgow	Wellcome Sanger Institute for the COVID-19 Genomics UK (COG-UK) consortium	Harper VanSteenhouse, Yumi Kasai, David Gray, Carol Clugston, Anna Dominiczak and Alex Alderton, Roberto Amato, Sonia Goncalves, Ewan Harrison, David K. Jackson, Ian Johnston, Dominic Kwiatkowski, Cordelia Langford, John Sillitoe on behalf of the Wellcome Sanger Institute COVID-19 Surveillance

			Team
EPI_ISL_630852	Lighthouse Lab in Alderley Park	Wellcome Sanger Institute for the COVID-19 Genomics UK (COG-UK) consortium	Jacquelyn Wynn, Mairead Hyland, The Lighthouse Lab in Alderley Park and Alex Alderton, Roberto Amato, Sonia Goncalves, Ewan Harrison, David K. Jackson, Ian Johnston, Dominic Kwiatkowski, Cordelia Langford, John Sillitoe on behalf of the Wellcome Sanger Institute COVID-19 Surveillance Team
EPI_ISL_630853	Lighthouse Lab in Glasgow	Wellcome Sanger Institute for the COVID-19 Genomics UK (COG-UK) consortium	Harper VanSteenhouse, Yumi Kasai, David Gray, Carol Clugston, Anna Dominiczak and Alex Alderton, Roberto Amato, Sonia Goncalves, Ewan Harrison, David K. Jackson, Ian Johnston, Dominic Kwiatkowski, Cordelia Langford, John Sillitoe on behalf of the Wellcome Sanger Institute COVID-19 Surveillance Team
EPI_ISL_630854, EPI_ISL_630855, EPI_ISL_630856, EPI_ISL_630857, EPI_ISL_630858	Lighthouse Lab in Alderley Park	Wellcome Sanger Institute for the COVID-19 Genomics UK (COG-UK) consortium	Jacquelyn Wynn, Mairead Hyland, The Lighthouse Lab in Alderley Park and Alex Alderton, Roberto Amato, Sonia Goncalves, Ewan Harrison, David K. Jackson, Ian Johnston, Dominic Kwiatkowski, Cordelia Langford, John Sillitoe on behalf of the Wellcome Sanger Institute COVID-19 Surveillance Team
EPI_ISL_630859	Lighthouse Lab in Glasgow	Wellcome Sanger Institute for the COVID-19 Genomics UK (COG-UK) consortium	Harper VanSteenhouse, Yumi Kasai, David Gray, Carol Clugston, Anna Dominiczak and Alex Alderton, Roberto Amato, Sonia Goncalves, Ewan Harrison, David K. Jackson, Ian Johnston, Dominic Kwiatkowski, Cordelia Langford, John Sillitoe on behalf of the Wellcome Sanger Institute COVID-19 Surveillance Team
EPI_ISL_630860, EPI_ISL_630863	Lighthouse Lab in Alderley Park	Wellcome Sanger Institute for the COVID-19 Genomics UK (COG-UK) consortium	Jacquelyn Wynn, Mairead Hyland, The Lighthouse Lab in Alderley Park and Alex Alderton, Roberto Amato, Sonia Goncalves, Ewan Harrison, David K. Jackson, Ian Johnston, Dominic Kwiatkowski, Cordelia Langford, John Sillitoe on behalf of the Wellcome Sanger Institute COVID-19 Surveillance Team
EPI_ISL_630864	Lighthouse Lab in Glasgow	Wellcome Sanger Institute for the COVID-19 Genomics UK (COG-UK) consortium	Harper VanSteenhouse, Yumi Kasai, David Gray, Carol Clugston, Anna Dominiczak and Alex Alderton, Roberto Amato, Sonia Goncalves, Ewan Harrison, David K. Jackson, Ian Johnston, Dominic Kwiatkowski, Cordelia Langford, John Sillitoe on behalf of the Wellcome Sanger Institute COVID-19 Surveillance Team
EPI_ISL_630865, EPI_ISL_630866, EPI_ISL_630867, EPI_ISL_630868, EPI_ISL_630869	Lighthouse Lab in Alderley Park	Wellcome Sanger Institute for the COVID-19 Genomics UK (COG-UK) consortium	Jacquelyn Wynn, Mairead Hyland, The Lighthouse Lab in Alderley Park and Alex Alderton, Roberto Amato, Sonia Goncalves, Ewan Harrison, David K. Jackson, Ian Johnston, Dominic Kwiatkowski, Cordelia Langford, John Sillitoe on behalf of the Wellcome Sanger Institute COVID-19 Surveillance Team
EPI_ISL_630871, EPI_ISL_630872, EPI_ISL_630873	Lighthouse Lab in Glasgow	Wellcome Sanger Institute for the COVID-19 Genomics UK (COG-UK) consortium	Harper VanSteenhouse, Yumi Kasai, David Gray, Carol Clugston, Anna Dominiczak and Alex Alderton, Roberto Amato, Sonia Goncalves, Ewan Harrison, David K. Jackson, Ian Johnston, Dominic Kwiatkowski, Cordelia Langford, John Sillitoe on behalf of the Wellcome Sanger Institute COVID-19 Surveillance Team
EPI_ISL_630874	Lighthouse Lab in Alderley Park	Wellcome Sanger Institute for the COVID-19 Genomics UK (COG-UK) consortium	Jacquelyn Wynn, Mairead Hyland, The Lighthouse Lab in Alderley Park and Alex Alderton, Roberto Amato, Sonia Goncalves, Ewan Harrison, David K. Jackson, Ian Johnston, Dominic Kwiatkowski, Cordelia Langford, John Sillitoe on behalf of the Wellcome Sanger Institute COVID-19 Surveillance Team
EPI_ISL_630875	Lighthouse Lab in Glasgow	Wellcome Sanger Institute for the COVID-19 Genomics UK (COG-UK) consortium	Harper VanSteenhouse, Yumi Kasai, David Gray, Carol Clugston, Anna Dominiczak and Alex Alderton, Roberto Amato, Sonia Goncalves, Ewan Harrison, David K. Jackson, Ian Johnston, Dominic Kwiatkowski, Cordelia Langford, John Sillitoe on behalf of the Wellcome Sanger Institute COVID-19 Surveillance Team
EPI_ISL_630877, EPI_ISL_630878, EPI_ISL_630879, EPI_ISL_630880, EPI_ISL_630881, EPI_ISL_630882	Lighthouse Lab in Alderley Park	Wellcome Sanger Institute for the COVID-19 Genomics UK (COG-UK) consortium	Jacquelyn Wynn, Mairead Hyland, The Lighthouse Lab in Alderley Park and Alex Alderton, Roberto Amato, Sonia Goncalves, Ewan Harrison, David K. Jackson, Ian Johnston, Dominic Kwiatkowski, Cordelia Langford, John Sillitoe on behalf of the Wellcome Sanger Institute COVID-19 Surveillance Team
EPI_ISL_630883	Lighthouse Lab in Glasgow	Wellcome Sanger Institute for the COVID-19 Genomics UK (COG-UK) consortium	Harper VanSteenhouse, Yumi Kasai, David Gray, Carol Clugston, Anna Dominiczak and Alex Alderton, Roberto Amato, Sonia Goncalves, Ewan Harrison, David K. Jackson, Ian Johnston, Dominic Kwiatkowski, Cordelia Langford, John Sillitoe on behalf of the Wellcome Sanger Institute COVID-19 Surveillance Team
EPI_ISL_630884, EPI_ISL_630885, EPI_ISL_630886, EPI_ISL_630887	Lighthouse Lab in Alderley Park	Wellcome Sanger Institute for the COVID-19 Genomics UK (COG-UK) consortium	Jacquelyn Wynn, Mairead Hyland, The Lighthouse Lab in Alderley Park and Alex Alderton, Roberto Amato, Sonia Goncalves, Ewan Harrison, David K. Jackson, Ian Johnston, Dominic Kwiatkowski, Cordelia Langford, John Sillitoe on behalf of the Wellcome Sanger Institute COVID-19 Surveillance Team
EPI_ISL_630888	Lighthouse Lab in Glasgow	Wellcome Sanger Institute for the COVID-19 Genomics UK (COG-UK) consortium	Harper VanSteenhouse, Yumi Kasai, David Gray, Carol Clugston, Anna Dominiczak and Alex Alderton, Roberto Amato, Sonia Goncalves, Ewan Harrison, David K. Jackson, Ian Johnston, Dominic Kwiatkowski, Cordelia Langford, John Sillitoe on behalf of the Wellcome Sanger Institute COVID-19 Surveillance Team
EPI_ISL_630889, EPI_ISL_630890, EPI_ISL_630892, EPI_ISL_630893, EPI_ISL_630896, EPI_ISL_630897, EPI_ISL_630898, EPI_ISL_630899	Lighthouse Lab in Alderley Park	Wellcome Sanger Institute for the COVID-19 Genomics UK (COG-UK) consortium	Jacquelyn Wynn, Mairead Hyland, The Lighthouse Lab in Alderley Park and Alex Alderton, Roberto Amato, Sonia Goncalves, Ewan Harrison, David K. Jackson, Ian Johnston, Dominic Kwiatkowski, Cordelia Langford, John Sillitoe on behalf of the Wellcome Sanger Institute COVID-19 Surveillance Team
EPI_ISL_630900	Lighthouse Lab in Glasgow	Wellcome Sanger Institute for the COVID-19 Genomics UK (COG-UK) consortium	Harper VanSteenhouse, Yumi Kasai, David Gray, Carol Clugston, Anna Dominiczak and Alex Alderton, Roberto Amato, Sonia Goncalves, Ewan Harrison, David K. Jackson, Ian Johnston, Dominic Kwiatkowski, Cordelia Langford, John Sillitoe on behalf of the Wellcome Sanger Institute COVID-19 Surveillance Team
EPI_ISL_630901, EPI_ISL_630902, EPI_ISL_630904, EPI_ISL_630905, EPI_ISL_630906, EPI_ISL_630907, EPI_ISL_630908, EPI_ISL_630909, EPI_ISL_630910, EPI_ISL_630912, EPI_ISL_630913			
see above	Lighthouse Lab in Alderley Park	Wellcome Sanger Institute for the COVID-19 Genomics UK (COG-UK) consortium	Jacquelyn Wynn, Mairead Hyland, The Lighthouse Lab in Alderley Park and Alex Alderton, Roberto Amato, Sonia Goncalves, Ewan Harrison, David K. Jackson, Ian Johnston, Dominic Kwiatkowski, Cordelia Langford, John Sillitoe on behalf of the Wellcome Sanger Institute COVID-19 Surveillance Team
EPI_ISL_630915	Lighthouse Lab in Glasgow	Wellcome Sanger Institute for the COVID-19 Genomics UK (COG-UK) consortium	Harper VanSteenhouse, Yumi Kasai, David Gray, Carol Clugston, Anna Dominiczak and Alex Alderton, Roberto Amato, Sonia Goncalves, Ewan Harrison, David K. Jackson, Ian Johnston, Dominic Kwiatkowski, Cordelia Langford, John Sillitoe on behalf of the Wellcome Sanger Institute COVID-19 Surveillance Team
EPI_ISL_630916, EPI_ISL_630917, EPI_ISL_630918, EPI_ISL_630920, EPI_ISL_630921, EPI_ISL_630922	Lighthouse Lab in Alderley Park	Wellcome Sanger Institute for the COVID-19 Genomics UK (COG-UK) consortium	Jacquelyn Wynn, Mairead Hyland, The Lighthouse Lab in Alderley Park and Alex Alderton, Roberto Amato, Sonia Goncalves, Ewan Harrison, David K. Jackson, Ian Johnston, Dominic Kwiatkowski, Cordelia Langford, John Sillitoe on behalf of the Wellcome Sanger Institute COVID-19 Surveillance Team
EPI_ISL_630923	Lighthouse Lab in Glasgow	Wellcome Sanger Institute for the COVID-19 Genomics UK (COG-UK) consortium	Harper VanSteenhouse, Yumi Kasai, David Gray, Carol Clugston, Anna Dominiczak and Alex Alderton, Roberto Amato, Sonia Goncalves, Ewan Harrison, David K. Jackson, Ian Johnston, Dominic Kwiatkowski, Cordelia Langford, John Sillitoe on behalf of the Wellcome Sanger Institute COVID-19 Surveillance Team
EPI_ISL_630924, EPI_ISL_630925	Lighthouse Lab in Alderley Park	Wellcome Sanger Institute for the COVID-19 Genomics UK (COG-UK) consortium	Jacquelyn Wynn, Mairead Hyland, The Lighthouse Lab in Alderley Park and Alex Alderton, Roberto Amato, Sonia Goncalves, Ewan Harrison, David K. Jackson, Ian Johnston, Dominic Kwiatkowski, Cordelia Langford, John Sillitoe on behalf of the Wellcome Sanger Institute COVID-19 Surveillance Team
EPI_ISL_630926	Lighthouse Lab in Glasgow	Wellcome Sanger Institute for the COVID-19 Genomics UK (COG-UK) consortium	Harper VanSteenhouse, Yumi Kasai, David Gray, Carol Clugston, Anna Dominiczak and Alex Alderton, Roberto Amato, Sonia Goncalves, Ewan Harrison, David K. Jackson, Ian Johnston, Dominic Kwiatkowski, Cordelia Langford, John Sillitoe on behalf of the Wellcome Sanger Institute COVID-19 Surveillance Team
EPI_ISL_630927, EPI_ISL_630928, EPI_ISL_630929, EPI_ISL_630930	Lighthouse Lab in Alderley Park	Wellcome Sanger Institute for the COVID-19 Genomics UK (COG-UK) consortium	Jacquelyn Wynn, Mairead Hyland, The Lighthouse Lab in Alderley Park and Alex Alderton, Roberto Amato, Sonia Goncalves, Ewan Harrison, David K. Jackson, Ian Johnston, Dominic Kwiatkowski, Cordelia Langford, John Sillitoe on behalf of the Wellcome Sanger Institute COVID-19 Surveillance Team
EPI_ISL_630931	Lighthouse Lab in Glasgow	Wellcome Sanger Institute for the COVID-19 Genomics UK (COG-UK) consortium	Harper VanSteenhouse, Yumi Kasai, David Gray, Carol Clugston, Anna Dominiczak and Alex Alderton, Roberto Amato, Sonia Goncalves, Ewan Harrison, David K. Jackson, Ian Johnston, Dominic Kwiatkowski, Cordelia Langford, John Sillitoe on behalf of the Wellcome Sanger Institute COVID-19 Surveillance Team
EPI_ISL_630932	Lighthouse Lab in Alderley Park	Wellcome Sanger Institute for the COVID-19 Genomics UK (COG-UK) consortium	Jacquelyn Wynn, Mairead Hyland, The Lighthouse Lab in Alderley Park and Alex Alderton, Roberto Amato, Sonia Goncalves, Ewan Harrison, David K. Jackson, Ian Johnston, Dominic Kwiatkowski, Cordelia Langford, John Sillitoe on behalf of the Wellcome Sanger Institute COVID-19 Surveillance Team
EPI_ISL_630933	Lighthouse Lab in Glasgow	Wellcome Sanger Institute for the COVID-19 Genomics UK (COG-UK) consortium	Harper VanSteenhouse, Yumi Kasai, David Gray, Carol Clugston, Anna Dominiczak and Alex Alderton, Roberto Amato, Sonia Goncalves, Ewan Harrison, David K. Jackson, Ian Johnston, Dominic Kwiatkowski, Cordelia Langford, John Sillitoe on behalf of the Wellcome Sanger Institute COVID-19 Surveillance Team
EPI_ISL_630934, EPI_ISL_630935, EPI_ISL_630938, EPI_ISL_630939, EPI_ISL_630940	Lighthouse Lab in Alderley Park	Wellcome Sanger Institute for the COVID-19 Genomics UK (COG-UK) consortium	Jacquelyn Wynn, Mairead Hyland, The Lighthouse Lab in Alderley Park and Alex Alderton, Roberto Amato, Sonia Goncalves, Ewan Harrison, David K. Jackson, Ian Johnston, Dominic Kwiatkowski, Cordelia Langford, John Sillitoe on behalf of the Wellcome Sanger Institute COVID-19 Surveillance Team
EPI_ISL_630941	Lighthouse Lab in Glasgow	Wellcome Sanger Institute for the COVID-19 Genomics UK (COG-UK) consortium	Harper VanSteenhouse, Yumi Kasai, David Gray, Carol Clugston, Anna Dominiczak and Alex Alderton, Roberto Amato, Sonia Goncalves, Ewan Harrison, David K. Jackson, Ian Johnston, Dominic Kwiatkowski, Cordelia Langford, John Sillitoe on behalf of the Wellcome Sanger Institute COVID-19 Surveillance Team

EPI_ISL_630942, EPI_ISL_630943, EPI_ISL_630944, EPI_ISL_630945, EPI_ISL_630946, EPI_ISL_630947	Lighthouse Lab in Alderley Park	Wellcome Sanger Institute for the COVID-19 Genomics UK (COG-UK) consortium	Jacquelyn Wynn, Mairead Hyland, The Lighthouse Lab in Alderley Park and Alex Alderton, Roberto Amato, Sonia Goncalves, Ewan Harrison, David K. Jackson, Ian Johnston, Dominic Kwiatkowski, Cordelia Langford, John Sillitoe on behalf of the Wellcome Sanger Institute COVID-19 Surveillance Team
EPI_ISL_630948, EPI_ISL_630949	Lighthouse Lab in Glasgow	Wellcome Sanger Institute for the COVID-19 Genomics UK (COG-UK) consortium	Harper VanSteenhouse, Yumi Kasai, David Gray, Carol Clugston, Anna Dominiczak and Alex Alderton, Roberto Amato, Sonia Goncalves, Ewan Harrison, David K. Jackson, Ian Johnston, Dominic Kwiatkowski, Cordelia Langford, John Sillitoe on behalf of the Wellcome Sanger Institute COVID-19 Surveillance Team
EPI_ISL_630950, EPI_ISL_630952, EPI_ISL_630954	Lighthouse Lab in Alderley Park	Wellcome Sanger Institute for the COVID-19 Genomics UK (COG-UK) consortium	Jacquelyn Wynn, Mairead Hyland, The Lighthouse Lab in Alderley Park and Alex Alderton, Roberto Amato, Sonia Goncalves, Ewan Harrison, David K. Jackson, Ian Johnston, Dominic Kwiatkowski, Cordelia Langford, John Sillitoe on behalf of the Wellcome Sanger Institute COVID-19 Surveillance Team
EPI_ISL_630955, EPI_ISL_630959	Lighthouse Lab in Glasgow	Wellcome Sanger Institute for the COVID-19 Genomics UK (COG-UK) consortium	Harper VanSteenhouse, Yumi Kasai, David Gray, Carol Clugston, Anna Dominiczak and Alex Alderton, Roberto Amato, Sonia Goncalves, Ewan Harrison, David K. Jackson, Ian Johnston, Dominic Kwiatkowski, Cordelia Langford, John Sillitoe on behalf of the Wellcome Sanger Institute COVID-19 Surveillance Team
EPI_ISL_630960, EPI_ISL_630961, EPI_ISL_630962, EPI_ISL_630963, EPI_ISL_630964	Lighthouse Lab in Alderley Park	Wellcome Sanger Institute for the COVID-19 Genomics UK (COG-UK) consortium	Jacquelyn Wynn, Mairead Hyland, The Lighthouse Lab in Alderley Park and Alex Alderton, Roberto Amato, Sonia Goncalves, Ewan Harrison, David K. Jackson, Ian Johnston, Dominic Kwiatkowski, Cordelia Langford, John Sillitoe on behalf of the Wellcome Sanger Institute COVID-19 Surveillance Team
EPI_ISL_630965	Lighthouse Lab in Glasgow	Wellcome Sanger Institute for the COVID-19 Genomics UK (COG-UK) consortium	Harper VanSteenhouse, Yumi Kasai, David Gray, Carol Clugston, Anna Dominiczak and Alex Alderton, Roberto Amato, Sonia Goncalves, Ewan Harrison, David K. Jackson, Ian Johnston, Dominic Kwiatkowski, Cordelia Langford, John Sillitoe on behalf of the Wellcome Sanger Institute COVID-19 Surveillance Team
EPI_ISL_630966, EPI_ISL_630968, EPI_ISL_630969, EPI_ISL_630970, EPI_ISL_630971	Lighthouse Lab in Alderley Park	Wellcome Sanger Institute for the COVID-19 Genomics UK (COG-UK) consortium	Jacquelyn Wynn, Mairead Hyland, The Lighthouse Lab in Alderley Park and Alex Alderton, Roberto Amato, Sonia Goncalves, Ewan Harrison, David K. Jackson, Ian Johnston, Dominic Kwiatkowski, Cordelia Langford, John Sillitoe on behalf of the Wellcome Sanger Institute COVID-19 Surveillance Team
EPI_ISL_630972	Lighthouse Lab in Cambridge	Wellcome Sanger Institute for the COVID-19 Genomics UK (COG-UK) consortium	Rob Howes, The Lighthouse Lab in Cambridge and Alex Alderton, Roberto Amato, Sonia Goncalves, Ewan Harrison, David K. Jackson, Ian Johnston, Dominic Kwiatkowski, Cordelia Langford, John Sillitoe on behalf of the Wellcome Sanger Institute COVID-19 Surveillance Team
EPI_ISL_630973, EPI_ISL_630974	Lighthouse Lab in Milton Keynes	Wellcome Sanger Institute for the COVID-19 Genomics UK (COG-UK) consortium	The Lighthouse Lab in Milton Keynes and Alex Alderton, Roberto Amato, Sonia Goncalves, Ewan Harrison, David K. Jackson, Ian Johnston, Dominic Kwiatkowski, Cordelia Langford, John Sillitoe on behalf of the Wellcome Sanger Institute COVID-19 Surveillance Team
EPI_ISL_630975, EPI_ISL_630976	Lighthouse Lab in Alderley Park	Wellcome Sanger Institute for the COVID-19 Genomics UK (COG-UK) consortium	Jacquelyn Wynn, Mairead Hyland, The Lighthouse Lab in Alderley Park and Alex Alderton, Roberto Amato, Sonia Goncalves, Ewan Harrison, David K. Jackson, Ian Johnston, Dominic Kwiatkowski, Cordelia Langford, John Sillitoe on behalf of the Wellcome Sanger Institute COVID-19 Surveillance Team
EPI_ISL_630977	Lighthouse Lab in Cambridge	Wellcome Sanger Institute for the COVID-19 Genomics UK (COG-UK) consortium	Rob Howes, The Lighthouse Lab in Cambridge and Alex Alderton, Roberto Amato, Sonia Goncalves, Ewan Harrison, David K. Jackson, Ian Johnston, Dominic Kwiatkowski, Cordelia Langford, John Sillitoe on behalf of the Wellcome Sanger Institute COVID-19 Surveillance Team
EPI_ISL_630978	Lighthouse Lab in Milton Keynes	Wellcome Sanger Institute for the COVID-19 Genomics UK (COG-UK) consortium	The Lighthouse Lab in Milton Keynes and Alex Alderton, Roberto Amato, Sonia Goncalves, Ewan Harrison, David K. Jackson, Ian Johnston, Dominic Kwiatkowski, Cordelia Langford, John Sillitoe on behalf of the Wellcome Sanger Institute COVID-19 Surveillance Team
EPI_ISL_630979	Lighthouse Lab in Cambridge	Wellcome Sanger Institute for the COVID-19 Genomics UK (COG-UK) consortium	Rob Howes, The Lighthouse Lab in Cambridge and Alex Alderton, Roberto Amato, Sonia Goncalves, Ewan Harrison, David K. Jackson, Ian Johnston, Dominic Kwiatkowski, Cordelia Langford, John Sillitoe on behalf of the Wellcome Sanger Institute COVID-19 Surveillance Team
EPI_ISL_630981	Lighthouse Lab in Milton Keynes	Wellcome Sanger Institute for the COVID-19 Genomics UK (COG-UK) consortium	The Lighthouse Lab in Milton Keynes and Alex Alderton, Roberto Amato, Sonia Goncalves, Ewan Harrison, David K. Jackson, Ian Johnston, Dominic Kwiatkowski, Cordelia Langford, John Sillitoe on behalf of the Wellcome Sanger Institute COVID-19 Surveillance Team
EPI_ISL_630982, EPI_ISL_630983	Lighthouse Lab in Alderley Park	Wellcome Sanger Institute for the COVID-19 Genomics UK (COG-UK) consortium	Jacquelyn Wynn, Mairead Hyland, The Lighthouse Lab in Alderley Park and Alex Alderton, Roberto Amato, Sonia Goncalves, Ewan Harrison, David K. Jackson, Ian Johnston, Dominic Kwiatkowski, Cordelia Langford, John Sillitoe on behalf of the Wellcome Sanger Institute COVID-19 Surveillance Team
EPI_ISL_630984	Lighthouse Lab in Cambridge	Wellcome Sanger Institute for the COVID-19 Genomics UK (COG-UK) consortium	Rob Howes, The Lighthouse Lab in Cambridge and Alex Alderton, Roberto Amato, Sonia Goncalves, Ewan Harrison, David K. Jackson, Ian Johnston, Dominic Kwiatkowski, Cordelia Langford, John Sillitoe on behalf of the Wellcome Sanger Institute COVID-19 Surveillance Team
EPI_ISL_630985	Lighthouse Lab in Alderley Park	Wellcome Sanger Institute for the COVID-19 Genomics UK (COG-UK) consortium	Jacquelyn Wynn, Mairead Hyland, The Lighthouse Lab in Alderley Park and Alex Alderton, Roberto Amato, Sonia Goncalves, Ewan Harrison, David K. Jackson, Ian Johnston, Dominic Kwiatkowski, Cordelia Langford, John Sillitoe on behalf of the Wellcome Sanger Institute COVID-19 Surveillance Team
EPI_ISL_630986	Lighthouse Lab in Milton Keynes	Wellcome Sanger Institute for the COVID-19 Genomics UK (COG-UK) consortium	The Lighthouse Lab in Milton Keynes and Alex Alderton, Roberto Amato, Sonia Goncalves, Ewan Harrison, David K. Jackson, Ian Johnston, Dominic Kwiatkowski, Cordelia Langford, John Sillitoe on behalf of the Wellcome Sanger Institute COVID-19 Surveillance Team
EPI_ISL_630988	Lighthouse Lab in Cambridge	Wellcome Sanger Institute for the COVID-19 Genomics UK (COG-UK) consortium	Rob Howes, The Lighthouse Lab in Cambridge and Alex Alderton, Roberto Amato, Sonia Goncalves, Ewan Harrison, David K. Jackson, Ian Johnston, Dominic Kwiatkowski, Cordelia Langford, John Sillitoe on behalf of the Wellcome Sanger Institute COVID-19 Surveillance Team
EPI_ISL_630990, EPI_ISL_630991, EPI_ISL_630993	Lighthouse Lab in Milton Keynes	Wellcome Sanger Institute for the COVID-19 Genomics UK (COG-UK) consortium	The Lighthouse Lab in Milton Keynes and Alex Alderton, Roberto Amato, Sonia Goncalves, Ewan Harrison, David K. Jackson, Ian Johnston, Dominic Kwiatkowski, Cordelia Langford, John Sillitoe on behalf of the Wellcome Sanger Institute COVID-19 Surveillance Team
EPI_ISL_630994	Lighthouse Lab in Alderley Park	Wellcome Sanger Institute for the COVID-19 Genomics UK (COG-UK) consortium	Jacquelyn Wynn, Mairead Hyland, The Lighthouse Lab in Alderley Park and Alex Alderton, Roberto Amato, Sonia Goncalves, Ewan Harrison, David K. Jackson, Ian Johnston, Dominic Kwiatkowski, Cordelia Langford, John Sillitoe on behalf of the Wellcome Sanger Institute COVID-19 Surveillance Team
EPI_ISL_630995	Lighthouse Lab in Milton Keynes	Wellcome Sanger Institute for the COVID-19 Genomics UK (COG-UK) consortium	The Lighthouse Lab in Milton Keynes and Alex Alderton, Roberto Amato, Sonia Goncalves, Ewan Harrison, David K. Jackson, Ian Johnston, Dominic Kwiatkowski, Cordelia Langford, John Sillitoe on behalf of the Wellcome Sanger Institute COVID-19 Surveillance Team
EPI_ISL_630996, EPI_ISL_630997	Lighthouse Lab in Cambridge	Wellcome Sanger Institute for the COVID-19 Genomics UK (COG-UK) consortium	Rob Howes, The Lighthouse Lab in Cambridge and Alex Alderton, Roberto Amato, Sonia Goncalves, Ewan Harrison, David K. Jackson, Ian Johnston, Dominic Kwiatkowski, Cordelia Langford, John Sillitoe on behalf of the Wellcome Sanger Institute COVID-19 Surveillance Team
EPI_ISL_630998	Lighthouse Lab in Milton Keynes	Wellcome Sanger Institute for the COVID-19 Genomics UK (COG-UK) consortium	The Lighthouse Lab in Milton Keynes and Alex Alderton, Roberto Amato, Sonia Goncalves, Ewan Harrison, David K. Jackson, Ian Johnston, Dominic Kwiatkowski, Cordelia Langford, John Sillitoe on behalf of the Wellcome Sanger Institute COVID-19 Surveillance Team
EPI_ISL_630999	Lighthouse Lab in Alderley Park	Wellcome Sanger Institute for the COVID-19 Genomics UK (COG-UK) consortium	Jacquelyn Wynn, Mairead Hyland, The Lighthouse Lab in Alderley Park and Alex Alderton, Roberto Amato, Sonia Goncalves, Ewan Harrison, David K. Jackson, Ian Johnston, Dominic Kwiatkowski, Cordelia Langford, John Sillitoe on behalf of the Wellcome Sanger Institute COVID-19 Surveillance Team
EPI_ISL_631000	Lighthouse Lab in Milton Keynes	Wellcome Sanger Institute for the COVID-19 Genomics UK (COG-UK) consortium	The Lighthouse Lab in Milton Keynes and Alex Alderton, Roberto Amato, Sonia Goncalves, Ewan Harrison, David K. Jackson, Ian Johnston, Dominic Kwiatkowski, Cordelia Langford, John Sillitoe on behalf of the Wellcome Sanger Institute COVID-19 Surveillance Team
EPI_ISL_631001	Lighthouse Lab in Cambridge	Wellcome Sanger Institute for the COVID-19 Genomics UK (COG-UK) consortium	Rob Howes, The Lighthouse Lab in Cambridge and Alex Alderton, Roberto Amato, Sonia Goncalves, Ewan Harrison, David K. Jackson, Ian Johnston, Dominic Kwiatkowski, Cordelia Langford, John Sillitoe on behalf of the Wellcome Sanger Institute COVID-19 Surveillance Team
EPI_ISL_631002	Lighthouse Lab in Milton Keynes	Wellcome Sanger Institute for the COVID-19 Genomics UK (COG-UK) consortium	The Lighthouse Lab in Milton Keynes and Alex Alderton, Roberto Amato, Sonia Goncalves, Ewan Harrison, David K. Jackson, Ian Johnston, Dominic Kwiatkowski, Cordelia Langford, John Sillitoe on behalf of the Wellcome Sanger Institute COVID-19 Surveillance Team
EPI_ISL_631003, EPI_ISL_631005	Lighthouse Lab in Cambridge	Wellcome Sanger Institute for the COVID-19 Genomics UK (COG-UK) consortium	Rob Howes, The Lighthouse Lab in Cambridge and Alex Alderton, Roberto Amato, Sonia Goncalves, Ewan Harrison, David K. Jackson, Ian Johnston, Dominic Kwiatkowski, Cordelia Langford, John Sillitoe on behalf of the Wellcome Sanger Institute COVID-19 Surveillance Team
EPI_ISL_631006, EPI_ISL_631007	Lighthouse Lab in Milton Keynes	Wellcome Sanger Institute for the COVID-19 Genomics UK (COG-UK) consortium	The Lighthouse Lab in Milton Keynes and Alex Alderton, Roberto Amato, Sonia Goncalves, Ewan Harrison, David K. Jackson, Ian Johnston, Dominic Kwiatkowski, Cordelia Langford, John Sillitoe on behalf of the Wellcome Sanger Institute COVID-19 Surveillance Team
EPI_ISL_631008	Lighthouse Lab in Cambridge	Wellcome Sanger Institute for the COVID-19 Genomics UK (COG-UK) consortium	Rob Howes, The Lighthouse Lab in Cambridge and Alex Alderton, Roberto Amato, Sonia Goncalves, Ewan Harrison, David K. Jackson, Ian Johnston, Dominic Kwiatkowski, Cordelia Langford, John Sillitoe on behalf of the Wellcome Sanger Institute COVID-19 Surveillance Team
EPI_ISL_631009, EPI_ISL_631010	Lighthouse Lab in Milton Keynes	Wellcome Sanger Institute for the COVID-19 Genomics UK (COG-UK) consortium	The Lighthouse Lab in Milton Keynes and Alex Alderton, Roberto Amato, Sonia Goncalves, Ewan Harrison, David K. Jackson, Ian Johnston, Dominic Kwiatkowski, Cordelia Langford, John Sillitoe on behalf of the Wellcome Sanger Institute COVID-19 Surveillance Team
EPI_ISL_631012	Lighthouse Lab in Alderley Park	Wellcome Sanger Institute for the COVID-19 Genomics UK (COG-UK) consortium	Jacquelyn Wynn, Mairead Hyland, The Lighthouse Lab in Alderley Park and Alex Alderton, Roberto Amato, Sonia Goncalves, Ewan Harrison, David K. Jackson, Ian Johnston, Dominic Kwiatkowski, Cordelia Langford, John Sillitoe on behalf of the Wellcome Sanger Institute COVID-19 Surveillance Team
EPI_ISL_631013	Lighthouse Lab in Milton Keynes	Wellcome Sanger Institute for the COVID-19 Genomics UK (COG-UK) consortium	The Lighthouse Lab in Milton Keynes and Alex Alderton, Roberto Amato, Sonia Goncalves, Ewan Harrison, David K. Jackson, Ian Johnston, Dominic Kwiatkowski, Cordelia Langford, John Sillitoe on behalf of the Wellcome Sanger Institute COVID-19 Surveillance Team

EPI_ISL_631014	Lighthouse Lab in Alderley Park	Wellcome Sanger Institute for the COVID-19 Genomics UK (COG-UK) consortium	Jacquelyn Wynn, Mairead Hyland, The Lighthouse Lab in Alderley Park and Alex Alderton, Roberto Amato, Sonia Goncalves, Ewan Harrison, David K. Jackson, Ian Johnston, Dominic Kwiatkowski, Cordelia Langford, John Sillitoe on behalf of the Wellcome Sanger Institute COVID-19 Surveillance Team
EPI_ISL_631015	Lighthouse Lab in Milton Keynes	Wellcome Sanger Institute for the COVID-19 Genomics UK (COG-UK) consortium	The Lighthouse Lab in Milton Keynes and Alex Alderton, Roberto Amato, Sonia Goncalves, Ewan Harrison, David K. Jackson, Ian Johnston, Dominic Kwiatkowski, Cordelia Langford, John Sillitoe on behalf of the Wellcome Sanger Institute COVID-19 Surveillance Team
EPI_ISL_631016	Lighthouse Lab in Cambridge	Wellcome Sanger Institute for the COVID-19 Genomics UK (COG-UK) consortium	Rob Howes, The Lighthouse Lab in Cambridge and Alex Alderton, Roberto Amato, Sonia Goncalves, Ewan Harrison, David K. Jackson, Ian Johnston, Dominic Kwiatkowski, Cordelia Langford, John Sillitoe on behalf of the Wellcome Sanger Institute COVID-19 Surveillance Team
EPI_ISL_631018	Lighthouse Lab in Alderley Park	Wellcome Sanger Institute for the COVID-19 Genomics UK (COG-UK) consortium	Jacquelyn Wynn, Mairead Hyland, The Lighthouse Lab in Alderley Park and Alex Alderton, Roberto Amato, Sonia Goncalves, Ewan Harrison, David K. Jackson, Ian Johnston, Dominic Kwiatkowski, Cordelia Langford, John Sillitoe on behalf of the Wellcome Sanger Institute COVID-19 Surveillance Team
EPI_ISL_631019, EPI_ISL_631020, EPI_ISL_631021, EPI_ISL_631022, EPI_ISL_631024	Lighthouse Lab in Milton Keynes	Wellcome Sanger Institute for the COVID-19 Genomics UK (COG-UK) consortium	The Lighthouse Lab in Milton Keynes and Alex Alderton, Roberto Amato, Sonia Goncalves, Ewan Harrison, David K. Jackson, Ian Johnston, Dominic Kwiatkowski, Cordelia Langford, John Sillitoe on behalf of the Wellcome Sanger Institute COVID-19 Surveillance Team
EPI_ISL_631025	Lighthouse Lab in Cambridge	Wellcome Sanger Institute for the COVID-19 Genomics UK (COG-UK) consortium	Rob Howes, The Lighthouse Lab in Cambridge and Alex Alderton, Roberto Amato, Sonia Goncalves, Ewan Harrison, David K. Jackson, Ian Johnston, Dominic Kwiatkowski, Cordelia Langford, John Sillitoe on behalf of the Wellcome Sanger Institute COVID-19 Surveillance Team
EPI_ISL_631026	Lighthouse Lab in Milton Keynes	Wellcome Sanger Institute for the COVID-19 Genomics UK (COG-UK) consortium	The Lighthouse Lab in Milton Keynes and Alex Alderton, Roberto Amato, Sonia Goncalves, Ewan Harrison, David K. Jackson, Ian Johnston, Dominic Kwiatkowski, Cordelia Langford, John Sillitoe on behalf of the Wellcome Sanger Institute COVID-19 Surveillance Team
EPI_ISL_631027	Lighthouse Lab in Alderley Park	Wellcome Sanger Institute for the COVID-19 Genomics UK (COG-UK) consortium	Jacquelyn Wynn, Mairead Hyland, The Lighthouse Lab in Alderley Park and Alex Alderton, Roberto Amato, Sonia Goncalves, Ewan Harrison, David K. Jackson, Ian Johnston, Dominic Kwiatkowski, Cordelia Langford, John Sillitoe on behalf of the Wellcome Sanger Institute COVID-19 Surveillance Team
EPI_ISL_631028	Lighthouse Lab in Cambridge	Wellcome Sanger Institute for the COVID-19 Genomics UK (COG-UK) consortium	Rob Howes, The Lighthouse Lab in Cambridge and Alex Alderton, Roberto Amato, Sonia Goncalves, Ewan Harrison, David K. Jackson, Ian Johnston, Dominic Kwiatkowski, Cordelia Langford, John Sillitoe on behalf of the Wellcome Sanger Institute COVID-19 Surveillance Team
EPI_ISL_631029, EPI_ISL_631030	Lighthouse Lab in Milton Keynes	Wellcome Sanger Institute for the COVID-19 Genomics UK (COG-UK) consortium	The Lighthouse Lab in Milton Keynes and Alex Alderton, Roberto Amato, Sonia Goncalves, Ewan Harrison, David K. Jackson, Ian Johnston, Dominic Kwiatkowski, Cordelia Langford, John Sillitoe on behalf of the Wellcome Sanger Institute COVID-19 Surveillance Team
EPI_ISL_631031	Lighthouse Lab in Alderley Park	Wellcome Sanger Institute for the COVID-19 Genomics UK (COG-UK) consortium	Jacquelyn Wynn, Mairead Hyland, The Lighthouse Lab in Alderley Park and Alex Alderton, Roberto Amato, Sonia Goncalves, Ewan Harrison, David K. Jackson, Ian Johnston, Dominic Kwiatkowski, Cordelia Langford, John Sillitoe on behalf of the Wellcome Sanger Institute COVID-19 Surveillance Team
EPI_ISL_631032	Lighthouse Lab in Milton Keynes	Wellcome Sanger Institute for the COVID-19 Genomics UK (COG-UK) consortium	The Lighthouse Lab in Milton Keynes and Alex Alderton, Roberto Amato, Sonia Goncalves, Ewan Harrison, David K. Jackson, Ian Johnston, Dominic Kwiatkowski, Cordelia Langford, John Sillitoe on behalf of the Wellcome Sanger Institute COVID-19 Surveillance Team
EPI_ISL_631033	Lighthouse Lab in Alderley Park	Wellcome Sanger Institute for the COVID-19 Genomics UK (COG-UK) consortium	Jacquelyn Wynn, Mairead Hyland, The Lighthouse Lab in Alderley Park and Alex Alderton, Roberto Amato, Sonia Goncalves, Ewan Harrison, David K. Jackson, Ian Johnston, Dominic Kwiatkowski, Cordelia Langford, John Sillitoe on behalf of the Wellcome Sanger Institute COVID-19 Surveillance Team
EPI_ISL_631034	Lighthouse Lab in Milton Keynes	Wellcome Sanger Institute for the COVID-19 Genomics UK (COG-UK) consortium	The Lighthouse Lab in Milton Keynes and Alex Alderton, Roberto Amato, Sonia Goncalves, Ewan Harrison, David K. Jackson, Ian Johnston, Dominic Kwiatkowski, Cordelia Langford, John Sillitoe on behalf of the Wellcome Sanger Institute COVID-19 Surveillance Team
EPI_ISL_631035	Lighthouse Lab in Alderley Park	Wellcome Sanger Institute for the COVID-19 Genomics UK (COG-UK) consortium	Jacquelyn Wynn, Mairead Hyland, The Lighthouse Lab in Alderley Park and Alex Alderton, Roberto Amato, Sonia Goncalves, Ewan Harrison, David K. Jackson, Ian Johnston, Dominic Kwiatkowski, Cordelia Langford, John Sillitoe on behalf of the Wellcome Sanger Institute COVID-19 Surveillance Team
EPI_ISL_631036, EPI_ISL_631037	Lighthouse Lab in Milton Keynes	Wellcome Sanger Institute for the COVID-19 Genomics UK (COG-UK) consortium	The Lighthouse Lab in Milton Keynes and Alex Alderton, Roberto Amato, Sonia Goncalves, Ewan Harrison, David K. Jackson, Ian Johnston, Dominic Kwiatkowski, Cordelia Langford, John Sillitoe on behalf of the Wellcome Sanger Institute COVID-19 Surveillance Team
EPI_ISL_631038, EPI_ISL_631039	Lighthouse Lab in Cambridge	Wellcome Sanger Institute for the COVID-19 Genomics UK (COG-UK) consortium	Rob Howes, The Lighthouse Lab in Cambridge and Alex Alderton, Roberto Amato, Sonia Goncalves, Ewan Harrison, David K. Jackson, Ian Johnston, Dominic Kwiatkowski, Cordelia Langford, John Sillitoe on behalf of the Wellcome Sanger Institute COVID-19 Surveillance Team
EPI_ISL_631040	Lighthouse Lab in Milton Keynes	Wellcome Sanger Institute for the COVID-19 Genomics UK (COG-UK) consortium	The Lighthouse Lab in Milton Keynes and Alex Alderton, Roberto Amato, Sonia Goncalves, Ewan Harrison, David K. Jackson, Ian Johnston, Dominic Kwiatkowski, Cordelia Langford, John Sillitoe on behalf of the Wellcome Sanger Institute COVID-19 Surveillance Team
EPI_ISL_631041	Lighthouse Lab in Alderley Park	Wellcome Sanger Institute for the COVID-19 Genomics UK (COG-UK) consortium	Jacquelyn Wynn, Mairead Hyland, The Lighthouse Lab in Alderley Park and Alex Alderton, Roberto Amato, Sonia Goncalves, Ewan Harrison, David K. Jackson, Ian Johnston, Dominic Kwiatkowski, Cordelia Langford, John Sillitoe on behalf of the Wellcome Sanger Institute COVID-19 Surveillance Team
EPI_ISL_631042, EPI_ISL_631043	Lighthouse Lab in Milton Keynes	Wellcome Sanger Institute for the COVID-19 Genomics UK (COG-UK) consortium	The Lighthouse Lab in Milton Keynes and Alex Alderton, Roberto Amato, Sonia Goncalves, Ewan Harrison, David K. Jackson, Ian Johnston, Dominic Kwiatkowski, Cordelia Langford, John Sillitoe on behalf of the Wellcome Sanger Institute COVID-19 Surveillance Team
EPI_ISL_631044	Lighthouse Lab in Alderley Park	Wellcome Sanger Institute for the COVID-19 Genomics UK (COG-UK) consortium	Jacquelyn Wynn, Mairead Hyland, The Lighthouse Lab in Alderley Park and Alex Alderton, Roberto Amato, Sonia Goncalves, Ewan Harrison, David K. Jackson, Ian Johnston, Dominic Kwiatkowski, Cordelia Langford, John Sillitoe on behalf of the Wellcome Sanger Institute COVID-19 Surveillance Team
EPI_ISL_631045	Lighthouse Lab in Cambridge	Wellcome Sanger Institute for the COVID-19 Genomics UK (COG-UK) consortium	Rob Howes, The Lighthouse Lab in Cambridge and Alex Alderton, Roberto Amato, Sonia Goncalves, Ewan Harrison, David K. Jackson, Ian Johnston, Dominic Kwiatkowski, Cordelia Langford, John Sillitoe on behalf of the Wellcome Sanger Institute COVID-19 Surveillance Team
EPI_ISL_631046	Lighthouse Lab in Alderley Park	Wellcome Sanger Institute for the COVID-19 Genomics UK (COG-UK) consortium	Jacquelyn Wynn, Mairead Hyland, The Lighthouse Lab in Alderley Park and Alex Alderton, Roberto Amato, Sonia Goncalves, Ewan Harrison, David K. Jackson, Ian Johnston, Dominic Kwiatkowski, Cordelia Langford, John Sillitoe on behalf of the Wellcome Sanger Institute COVID-19 Surveillance Team
EPI_ISL_631047, EPI_ISL_631048, EPI_ISL_631049	Lighthouse Lab in Milton Keynes	Wellcome Sanger Institute for the COVID-19 Genomics UK (COG-UK) consortium	The Lighthouse Lab in Milton Keynes and Alex Alderton, Roberto Amato, Sonia Goncalves, Ewan Harrison, David K. Jackson, Ian Johnston, Dominic Kwiatkowski, Cordelia Langford, John Sillitoe on behalf of the Wellcome Sanger Institute COVID-19 Surveillance Team
EPI_ISL_631050	Lighthouse Lab in Alderley Park	Wellcome Sanger Institute for the COVID-19 Genomics UK (COG-UK) consortium	Jacquelyn Wynn, Mairead Hyland, The Lighthouse Lab in Alderley Park and Alex Alderton, Roberto Amato, Sonia Goncalves, Ewan Harrison, David K. Jackson, Ian Johnston, Dominic Kwiatkowski, Cordelia Langford, John Sillitoe on behalf of the Wellcome Sanger Institute COVID-19 Surveillance Team
EPI_ISL_631051, EPI_ISL_631052	Lighthouse Lab in Milton Keynes	Wellcome Sanger Institute for the COVID-19 Genomics UK (COG-UK) consortium	The Lighthouse Lab in Milton Keynes and Alex Alderton, Roberto Amato, Sonia Goncalves, Ewan Harrison, David K. Jackson, Ian Johnston, Dominic Kwiatkowski, Cordelia Langford, John Sillitoe on behalf of the Wellcome Sanger Institute COVID-19 Surveillance Team
EPI_ISL_631053, EPI_ISL_631055	Lighthouse Lab in Cambridge	Wellcome Sanger Institute for the COVID-19 Genomics UK (COG-UK) consortium	Rob Howes, The Lighthouse Lab in Cambridge and Alex Alderton, Roberto Amato, Sonia Goncalves, Ewan Harrison, David K. Jackson, Ian Johnston, Dominic Kwiatkowski, Cordelia Langford, John Sillitoe on behalf of the Wellcome Sanger Institute COVID-19 Surveillance Team
EPI_ISL_631056, EPI_ISL_631057	Lighthouse Lab in Alderley Park	Wellcome Sanger Institute for the COVID-19 Genomics UK (COG-UK) consortium	Jacquelyn Wynn, Mairead Hyland, The Lighthouse Lab in Alderley Park and Alex Alderton, Roberto Amato, Sonia Goncalves, Ewan Harrison, David K. Jackson, Ian Johnston, Dominic Kwiatkowski, Cordelia Langford, John Sillitoe on behalf of the Wellcome Sanger Institute COVID-19 Surveillance Team
EPI_ISL_631058	Lighthouse Lab in Milton Keynes	Wellcome Sanger Institute for the COVID-19 Genomics UK (COG-UK) consortium	The Lighthouse Lab in Milton Keynes and Alex Alderton, Roberto Amato, Sonia Goncalves, Ewan Harrison, David K. Jackson, Ian Johnston, Dominic Kwiatkowski, Cordelia Langford, John Sillitoe on behalf of the Wellcome Sanger Institute COVID-19 Surveillance Team
EPI_ISL_631059, EPI_ISL_631060	Lighthouse Lab in Alderley Park	Wellcome Sanger Institute for the COVID-19 Genomics UK (COG-UK) consortium	Jacquelyn Wynn, Mairead Hyland, The Lighthouse Lab in Alderley Park and Alex Alderton, Roberto Amato, Sonia Goncalves, Ewan Harrison, David K. Jackson, Ian Johnston, Dominic Kwiatkowski, Cordelia Langford, John Sillitoe on behalf of the Wellcome Sanger Institute COVID-19 Surveillance Team
EPI_ISL_631061	Lighthouse Lab in Milton Keynes	Wellcome Sanger Institute for the COVID-19 Genomics UK (COG-UK) consortium	The Lighthouse Lab in Milton Keynes and Alex Alderton, Roberto Amato, Sonia Goncalves, Ewan Harrison, David K. Jackson, Ian Johnston, Dominic Kwiatkowski, Cordelia Langford, John Sillitoe on behalf of the Wellcome Sanger Institute COVID-19 Surveillance Team
EPI_ISL_631062, EPI_ISL_631063	Lighthouse Lab in Cambridge	Wellcome Sanger Institute for the COVID-19 Genomics UK (COG-UK) consortium	Rob Howes, The Lighthouse Lab in Cambridge and Alex Alderton, Roberto Amato, Sonia Goncalves, Ewan Harrison, David K. Jackson, Ian Johnston, Dominic Kwiatkowski, Cordelia Langford, John Sillitoe on behalf of the Wellcome Sanger Institute COVID-19 Surveillance Team
EPI_ISL_631064, EPI_ISL_631065, EPI_ISL_631066, EPI_ISL_631067	Lighthouse Lab in Milton Keynes	Wellcome Sanger Institute for the COVID-19 Genomics UK (COG-UK) consortium	The Lighthouse Lab in Milton Keynes and Alex Alderton, Roberto Amato, Sonia Goncalves, Ewan Harrison, David K. Jackson, Ian Johnston, Dominic Kwiatkowski, Cordelia Langford, John Sillitoe on behalf of the Wellcome Sanger Institute COVID-19 Surveillance Team
EPI_ISL_631069	Lighthouse Lab in Cambridge	Wellcome Sanger Institute for the COVID-19 Genomics UK (COG-UK) consortium	Rob Howes, The Lighthouse Lab in Cambridge and Alex Alderton, Roberto Amato, Sonia Goncalves, Ewan Harrison, David K. Jackson, Ian Johnston, Dominic Kwiatkowski, Cordelia Langford, John Sillitoe on behalf of the Wellcome Sanger Institute COVID-19 Surveillance Team
EPI_ISL_631070, EPI_ISL_631071, EPI_ISL_631072, EPI_ISL_631073	Lighthouse Lab in Milton Keynes	Wellcome Sanger Institute for the COVID-19 Genomics UK (COG-UK) consortium	The Lighthouse Lab in Milton Keynes and Alex Alderton, Roberto Amato, Sonia Goncalves, Ewan Harrison, David K. Jackson, Ian Johnston, Dominic Kwiatkowski, Cordelia Langford, John Sillitoe on behalf of the Wellcome Sanger Institute COVID-19 Surveillance Team

EPI_ISL_631074	Lighthouse Lab in Alderley Park	Wellcome Sanger Institute for the COVID-19 Genomics UK (COG-UK) consortium	Jacquelyn Wynn, Mairead Hyland, The Lighthouse Lab in Alderley Park and Alex Alderton, Roberto Amato, Sonia Goncalves, Ewan Harrison, David K. Jackson, Ian Johnston, Dominic Kwiatkowski, Cordelia Langford, John Sillitoe on behalf of the Wellcome Sanger Institute COVID-19 Surveillance Team
EPI_ISL_631075, EPI_ISL_631076, EPI_ISL_631077	Lighthouse Lab in Milton Keynes	Wellcome Sanger Institute for the COVID-19 Genomics UK (COG-UK) consortium	The Lighthouse Lab in Milton Keynes and Alex Alderton, Roberto Amato, Sonia Goncalves, Ewan Harrison, David K. Jackson, Ian Johnston, Dominic Kwiatkowski, Cordelia Langford, John Sillitoe on behalf of the Wellcome Sanger Institute COVID-19 Surveillance Team
EPI_ISL_631078	Lighthouse Lab in Cambridge	Wellcome Sanger Institute for the COVID-19 Genomics UK (COG-UK) consortium	Rob Howes, The Lighthouse Lab in Cambridge and Alex Alderton, Roberto Amato, Sonia Goncalves, Ewan Harrison, David K. Jackson, Ian Johnston, Dominic Kwiatkowski, Cordelia Langford, John Sillitoe on behalf of the Wellcome Sanger Institute COVID-19 Surveillance Team
EPI_ISL_631079	Lighthouse Lab in Milton Keynes	Wellcome Sanger Institute for the COVID-19 Genomics UK (COG-UK) consortium	The Lighthouse Lab in Milton Keynes and Alex Alderton, Roberto Amato, Sonia Goncalves, Ewan Harrison, David K. Jackson, Ian Johnston, Dominic Kwiatkowski, Cordelia Langford, John Sillitoe on behalf of the Wellcome Sanger Institute COVID-19 Surveillance Team
EPI_ISL_631080	Lighthouse Lab in Cambridge	Wellcome Sanger Institute for the COVID-19 Genomics UK (COG-UK) consortium	Rob Howes, The Lighthouse Lab in Cambridge and Alex Alderton, Roberto Amato, Sonia Goncalves, Ewan Harrison, David K. Jackson, Ian Johnston, Dominic Kwiatkowski, Cordelia Langford, John Sillitoe on behalf of the Wellcome Sanger Institute COVID-19 Surveillance Team
EPI_ISL_631082, EPI_ISL_631083	Lighthouse Lab in Milton Keynes	Wellcome Sanger Institute for the COVID-19 Genomics UK (COG-UK) consortium	The Lighthouse Lab in Milton Keynes and Alex Alderton, Roberto Amato, Sonia Goncalves, Ewan Harrison, David K. Jackson, Ian Johnston, Dominic Kwiatkowski, Cordelia Langford, John Sillitoe on behalf of the Wellcome Sanger Institute COVID-19 Surveillance Team
EPI_ISL_631084	Lighthouse Lab in Alderley Park	Wellcome Sanger Institute for the COVID-19 Genomics UK (COG-UK) consortium	Jacquelyn Wynn, Mairead Hyland, The Lighthouse Lab in Alderley Park and Alex Alderton, Roberto Amato, Sonia Goncalves, Ewan Harrison, David K. Jackson, Ian Johnston, Dominic Kwiatkowski, Cordelia Langford, John Sillitoe on behalf of the Wellcome Sanger Institute COVID-19 Surveillance Team
EPI_ISL_631085, EPI_ISL_631087, EPI_ISL_631088	Lighthouse Lab in Milton Keynes	Wellcome Sanger Institute for the COVID-19 Genomics UK (COG-UK) consortium	The Lighthouse Lab in Milton Keynes and Alex Alderton, Roberto Amato, Sonia Goncalves, Ewan Harrison, David K. Jackson, Ian Johnston, Dominic Kwiatkowski, Cordelia Langford, John Sillitoe on behalf of the Wellcome Sanger Institute COVID-19 Surveillance Team
EPI_ISL_631089	Lighthouse Lab in Alderley Park	Wellcome Sanger Institute for the COVID-19 Genomics UK (COG-UK) consortium	Jacquelyn Wynn, Mairead Hyland, The Lighthouse Lab in Alderley Park and Alex Alderton, Roberto Amato, Sonia Goncalves, Ewan Harrison, David K. Jackson, Ian Johnston, Dominic Kwiatkowski, Cordelia Langford, John Sillitoe on behalf of the Wellcome Sanger Institute COVID-19 Surveillance Team
EPI_ISL_631091, EPI_ISL_631092	Lighthouse Lab in Milton Keynes	Wellcome Sanger Institute for the COVID-19 Genomics UK (COG-UK) consortium	The Lighthouse Lab in Milton Keynes and Alex Alderton, Roberto Amato, Sonia Goncalves, Ewan Harrison, David K. Jackson, Ian Johnston, Dominic Kwiatkowski, Cordelia Langford, John Sillitoe on behalf of the Wellcome Sanger Institute COVID-19 Surveillance Team
EPI_ISL_631093	Lighthouse Lab in Alderley Park	Wellcome Sanger Institute for the COVID-19 Genomics UK (COG-UK) consortium	Jacquelyn Wynn, Mairead Hyland, The Lighthouse Lab in Alderley Park and Alex Alderton, Roberto Amato, Sonia Goncalves, Ewan Harrison, David K. Jackson, Ian Johnston, Dominic Kwiatkowski, Cordelia Langford, John Sillitoe on behalf of the Wellcome Sanger Institute COVID-19 Surveillance Team
EPI_ISL_631094	Lighthouse Lab in Cambridge	Wellcome Sanger Institute for the COVID-19 Genomics UK (COG-UK) consortium	Rob Howes, The Lighthouse Lab in Cambridge and Alex Alderton, Roberto Amato, Sonia Goncalves, Ewan Harrison, David K. Jackson, Ian Johnston, Dominic Kwiatkowski, Cordelia Langford, John Sillitoe on behalf of the Wellcome Sanger Institute COVID-19 Surveillance Team
EPI_ISL_631095	Lighthouse Lab in Milton Keynes	Wellcome Sanger Institute for the COVID-19 Genomics UK (COG-UK) consortium	The Lighthouse Lab in Milton Keynes and Alex Alderton, Roberto Amato, Sonia Goncalves, Ewan Harrison, David K. Jackson, Ian Johnston, Dominic Kwiatkowski, Cordelia Langford, John Sillitoe on behalf of the Wellcome Sanger Institute COVID-19 Surveillance Team
EPI_ISL_631096	Lighthouse Lab in Alderley Park	Wellcome Sanger Institute for the COVID-19 Genomics UK (COG-UK) consortium	Jacquelyn Wynn, Mairead Hyland, The Lighthouse Lab in Alderley Park and Alex Alderton, Roberto Amato, Sonia Goncalves, Ewan Harrison, David K. Jackson, Ian Johnston, Dominic Kwiatkowski, Cordelia Langford, John Sillitoe on behalf of the Wellcome Sanger Institute COVID-19 Surveillance Team
EPI_ISL_631098	Lighthouse Lab in Milton Keynes	Wellcome Sanger Institute for the COVID-19 Genomics UK (COG-UK) consortium	The Lighthouse Lab in Milton Keynes and Alex Alderton, Roberto Amato, Sonia Goncalves, Ewan Harrison, David K. Jackson, Ian Johnston, Dominic Kwiatkowski, Cordelia Langford, John Sillitoe on behalf of the Wellcome Sanger Institute COVID-19 Surveillance Team
EPI_ISL_631099	Lighthouse Lab in Alderley Park	Wellcome Sanger Institute for the COVID-19 Genomics UK (COG-UK) consortium	Jacquelyn Wynn, Mairead Hyland, The Lighthouse Lab in Alderley Park and Alex Alderton, Roberto Amato, Sonia Goncalves, Ewan Harrison, David K. Jackson, Ian Johnston, Dominic Kwiatkowski, Cordelia Langford, John Sillitoe on behalf of the Wellcome Sanger Institute COVID-19 Surveillance Team
EPI_ISL_631100	Lighthouse Lab in Milton Keynes	Wellcome Sanger Institute for the COVID-19 Genomics UK (COG-UK) consortium	The Lighthouse Lab in Milton Keynes and Alex Alderton, Roberto Amato, Sonia Goncalves, Ewan Harrison, David K. Jackson, Ian Johnston, Dominic Kwiatkowski, Cordelia Langford, John Sillitoe on behalf of the Wellcome Sanger Institute COVID-19 Surveillance Team
EPI_ISL_631101	Lighthouse Lab in Cambridge	Wellcome Sanger Institute for the COVID-19 Genomics UK (COG-UK) consortium	Rob Howes, The Lighthouse Lab in Cambridge and Alex Alderton, Roberto Amato, Sonia Goncalves, Ewan Harrison, David K. Jackson, Ian Johnston, Dominic Kwiatkowski, Cordelia Langford, John Sillitoe on behalf of the Wellcome Sanger Institute COVID-19 Surveillance Team
EPI_ISL_631103, EPI_ISL_631104	Lighthouse Lab in Milton Keynes	Wellcome Sanger Institute for the COVID-19 Genomics UK (COG-UK) consortium	The Lighthouse Lab in Milton Keynes and Alex Alderton, Roberto Amato, Sonia Goncalves, Ewan Harrison, David K. Jackson, Ian Johnston, Dominic Kwiatkowski, Cordelia Langford, John Sillitoe on behalf of the Wellcome Sanger Institute COVID-19 Surveillance Team
EPI_ISL_631105	Lighthouse Lab in Cambridge	Wellcome Sanger Institute for the COVID-19 Genomics UK (COG-UK) consortium	Rob Howes, The Lighthouse Lab in Cambridge and Alex Alderton, Roberto Amato, Sonia Goncalves, Ewan Harrison, David K. Jackson, Ian Johnston, Dominic Kwiatkowski, Cordelia Langford, John Sillitoe on behalf of the Wellcome Sanger Institute COVID-19 Surveillance Team
EPI_ISL_631106	Lighthouse Lab in Milton Keynes	Wellcome Sanger Institute for the COVID-19 Genomics UK (COG-UK) consortium	The Lighthouse Lab in Milton Keynes and Alex Alderton, Roberto Amato, Sonia Goncalves, Ewan Harrison, David K. Jackson, Ian Johnston, Dominic Kwiatkowski, Cordelia Langford, John Sillitoe on behalf of the Wellcome Sanger Institute COVID-19 Surveillance Team
EPI_ISL_631107	Lighthouse Lab in Alderley Park	Wellcome Sanger Institute for the COVID-19 Genomics UK (COG-UK) consortium	Jacquelyn Wynn, Mairead Hyland, The Lighthouse Lab in Alderley Park and Alex Alderton, Roberto Amato, Sonia Goncalves, Ewan Harrison, David K. Jackson, Ian Johnston, Dominic Kwiatkowski, Cordelia Langford, John Sillitoe on behalf of the Wellcome Sanger Institute COVID-19 Surveillance Team
EPI_ISL_631108	Lighthouse Lab in Milton Keynes	Wellcome Sanger Institute for the COVID-19 Genomics UK (COG-UK) consortium	The Lighthouse Lab in Milton Keynes and Alex Alderton, Roberto Amato, Sonia Goncalves, Ewan Harrison, David K. Jackson, Ian Johnston, Dominic Kwiatkowski, Cordelia Langford, John Sillitoe on behalf of the Wellcome Sanger Institute COVID-19 Surveillance Team
EPI_ISL_631109	Lighthouse Lab in Cambridge	Wellcome Sanger Institute for the COVID-19 Genomics UK (COG-UK) consortium	Rob Howes, The Lighthouse Lab in Cambridge and Alex Alderton, Roberto Amato, Sonia Goncalves, Ewan Harrison, David K. Jackson, Ian Johnston, Dominic Kwiatkowski, Cordelia Langford, John Sillitoe on behalf of the Wellcome Sanger Institute COVID-19 Surveillance Team
EPI_ISL_631110	Lighthouse Lab in Milton Keynes	Wellcome Sanger Institute for the COVID-19 Genomics UK (COG-UK) consortium	The Lighthouse Lab in Milton Keynes and Alex Alderton, Roberto Amato, Sonia Goncalves, Ewan Harrison, David K. Jackson, Ian Johnston, Dominic Kwiatkowski, Cordelia Langford, John Sillitoe on behalf of the Wellcome Sanger Institute COVID-19 Surveillance Team
EPI_ISL_631111, EPI_ISL_631113	Lighthouse Lab in Alderley Park	Wellcome Sanger Institute for the COVID-19 Genomics UK (COG-UK) consortium	Jacquelyn Wynn, Mairead Hyland, The Lighthouse Lab in Alderley Park and Alex Alderton, Roberto Amato, Sonia Goncalves, Ewan Harrison, David K. Jackson, Ian Johnston, Dominic Kwiatkowski, Cordelia Langford, John Sillitoe on behalf of the Wellcome Sanger Institute COVID-19 Surveillance Team
EPI_ISL_631114, EPI_ISL_631115	Lighthouse Lab in Milton Keynes	Wellcome Sanger Institute for the COVID-19 Genomics UK (COG-UK) consortium	The Lighthouse Lab in Milton Keynes and Alex Alderton, Roberto Amato, Sonia Goncalves, Ewan Harrison, David K. Jackson, Ian Johnston, Dominic Kwiatkowski, Cordelia Langford, John Sillitoe on behalf of the Wellcome Sanger Institute COVID-19 Surveillance Team
EPI_ISL_631116	Lighthouse Lab in Alderley Park	Wellcome Sanger Institute for the COVID-19 Genomics UK (COG-UK) consortium	Jacquelyn Wynn, Mairead Hyland, The Lighthouse Lab in Alderley Park and Alex Alderton, Roberto Amato, Sonia Goncalves, Ewan Harrison, David K. Jackson, Ian Johnston, Dominic Kwiatkowski, Cordelia Langford, John Sillitoe on behalf of the Wellcome Sanger Institute COVID-19 Surveillance Team
EPI_ISL_631117	Lighthouse Lab in Cambridge	Wellcome Sanger Institute for the COVID-19 Genomics UK (COG-UK) consortium	Rob Howes, The Lighthouse Lab in Cambridge and Alex Alderton, Roberto Amato, Sonia Goncalves, Ewan Harrison, David K. Jackson, Ian Johnston, Dominic Kwiatkowski, Cordelia Langford, John Sillitoe on behalf of the Wellcome Sanger Institute COVID-19 Surveillance Team
EPI_ISL_631119, EPI_ISL_631120, EPI_ISL_631121, EPI_ISL_631122	Lighthouse Lab in Milton Keynes	Wellcome Sanger Institute for the COVID-19 Genomics UK (COG-UK) consortium	The Lighthouse Lab in Milton Keynes and Alex Alderton, Roberto Amato, Sonia Goncalves, Ewan Harrison, David K. Jackson, Ian Johnston, Dominic Kwiatkowski, Cordelia Langford, John Sillitoe on behalf of the Wellcome Sanger Institute COVID-19 Surveillance Team
EPI_ISL_631123	Lighthouse Lab in Cambridge	Wellcome Sanger Institute for the COVID-19 Genomics UK (COG-UK) consortium	Rob Howes, The Lighthouse Lab in Cambridge and Alex Alderton, Roberto Amato, Sonia Goncalves, Ewan Harrison, David K. Jackson, Ian Johnston, Dominic Kwiatkowski, Cordelia Langford, John Sillitoe on behalf of the Wellcome Sanger Institute COVID-19 Surveillance Team
EPI_ISL_631124, EPI_ISL_631125, EPI_ISL_631126	Lighthouse Lab in Milton Keynes	Wellcome Sanger Institute for the COVID-19 Genomics UK (COG-UK) consortium	The Lighthouse Lab in Milton Keynes and Alex Alderton, Roberto Amato, Sonia Goncalves, Ewan Harrison, David K. Jackson, Ian Johnston, Dominic Kwiatkowski, Cordelia Langford, John Sillitoe on behalf of the Wellcome Sanger Institute COVID-19 Surveillance Team
EPI_ISL_631127, EPI_ISL_631128	Lighthouse Lab in Alderley Park	Wellcome Sanger Institute for the COVID-19 Genomics UK (COG-UK) consortium	Jacquelyn Wynn, Mairead Hyland, The Lighthouse Lab in Alderley Park and Alex Alderton, Roberto Amato, Sonia Goncalves, Ewan Harrison, David K. Jackson, Ian Johnston, Dominic Kwiatkowski, Cordelia Langford, John Sillitoe on behalf of the Wellcome Sanger Institute COVID-19 Surveillance Team
EPI_ISL_631129	Lighthouse Lab in Cambridge	Wellcome Sanger Institute for the COVID-19 Genomics UK (COG-UK) consortium	Rob Howes, The Lighthouse Lab in Cambridge and Alex Alderton, Roberto Amato, Sonia Goncalves, Ewan Harrison, David K. Jackson, Ian Johnston, Dominic Kwiatkowski, Cordelia Langford, John Sillitoe on behalf of the Wellcome Sanger Institute COVID-19 Surveillance Team
EPI_ISL_631130	Lighthouse Lab in Milton Keynes	Wellcome Sanger Institute for the COVID-19 Genomics UK (COG-UK) consortium	The Lighthouse Lab in Milton Keynes and Alex Alderton, Roberto Amato, Sonia Goncalves, Ewan Harrison, David K. Jackson, Ian Johnston, Dominic Kwiatkowski, Cordelia Langford, John Sillitoe on behalf of the Wellcome Sanger Institute COVID-19 Surveillance Team

EPI_ISL_631131, EPI_ISL_631133	Lighthouse Lab in Alderley Park	Wellcome Sanger Institute for the COVID-19 Genomics UK (COG-UK) consortium	Jacquelyn Wynn, Mairead Hyland, The Lighthouse Lab in Alderley Park and Alex Alderton, Roberto Amato, Sonia Goncalves, Ewan Harrison, David K. Jackson, Ian Johnston, Dominic Kwiatkowski, Cordelia Langford, John Sillitoe on behalf of the Wellcome Sanger Institute COVID-19 Surveillance Team
EPI_ISL_631134	Lighthouse Lab in Milton Keynes	Wellcome Sanger Institute for the COVID-19 Genomics UK (COG-UK) consortium	The Lighthouse Lab in Milton Keynes and Alex Alderton, Roberto Amato, Sonia Goncalves, Ewan Harrison, David K. Jackson, Ian Johnston, Dominic Kwiatkowski, Cordelia Langford, John Sillitoe on behalf of the Wellcome Sanger Institute COVID-19 Surveillance Team
EPI_ISL_631135	Lighthouse Lab in Cambridge	Wellcome Sanger Institute for the COVID-19 Genomics UK (COG-UK) consortium	Rob Howes, The Lighthouse Lab in Cambridge and Alex Alderton, Roberto Amato, Sonia Goncalves, Ewan Harrison, David K. Jackson, Ian Johnston, Dominic Kwiatkowski, Cordelia Langford, John Sillitoe on behalf of the Wellcome Sanger Institute COVID-19 Surveillance Team
EPI_ISL_631136	Lighthouse Lab in Milton Keynes	Wellcome Sanger Institute for the COVID-19 Genomics UK (COG-UK) consortium	The Lighthouse Lab in Milton Keynes and Alex Alderton, Roberto Amato, Sonia Goncalves, Ewan Harrison, David K. Jackson, Ian Johnston, Dominic Kwiatkowski, Cordelia Langford, John Sillitoe on behalf of the Wellcome Sanger Institute COVID-19 Surveillance Team
EPI_ISL_631137	Lighthouse Lab in Cambridge	Wellcome Sanger Institute for the COVID-19 Genomics UK (COG-UK) consortium	Rob Howes, The Lighthouse Lab in Cambridge and Alex Alderton, Roberto Amato, Sonia Goncalves, Ewan Harrison, David K. Jackson, Ian Johnston, Dominic Kwiatkowski, Cordelia Langford, John Sillitoe on behalf of the Wellcome Sanger Institute COVID-19 Surveillance Team
EPI_ISL_631138, EPI_ISL_631139, EPI_ISL_631140, EPI_ISL_631141	Lighthouse Lab in Milton Keynes	Wellcome Sanger Institute for the COVID-19 Genomics UK (COG-UK) consortium	The Lighthouse Lab in Milton Keynes and Alex Alderton, Roberto Amato, Sonia Goncalves, Ewan Harrison, David K. Jackson, Ian Johnston, Dominic Kwiatkowski, Cordelia Langford, John Sillitoe on behalf of the Wellcome Sanger Institute COVID-19 Surveillance Team
EPI_ISL_631142	Lighthouse Lab in Cambridge	Wellcome Sanger Institute for the COVID-19 Genomics UK (COG-UK) consortium	Rob Howes, The Lighthouse Lab in Cambridge and Alex Alderton, Roberto Amato, Sonia Goncalves, Ewan Harrison, David K. Jackson, Ian Johnston, Dominic Kwiatkowski, Cordelia Langford, John Sillitoe on behalf of the Wellcome Sanger Institute COVID-19 Surveillance Team
EPI_ISL_631143	Lighthouse Lab in Milton Keynes	Wellcome Sanger Institute for the COVID-19 Genomics UK (COG-UK) consortium	The Lighthouse Lab in Milton Keynes and Alex Alderton, Roberto Amato, Sonia Goncalves, Ewan Harrison, David K. Jackson, Ian Johnston, Dominic Kwiatkowski, Cordelia Langford, John Sillitoe on behalf of the Wellcome Sanger Institute COVID-19 Surveillance Team
EPI_ISL_631144	Lighthouse Lab in Cambridge	Wellcome Sanger Institute for the COVID-19 Genomics UK (COG-UK) consortium	Rob Howes, The Lighthouse Lab in Cambridge and Alex Alderton, Roberto Amato, Sonia Goncalves, Ewan Harrison, David K. Jackson, Ian Johnston, Dominic Kwiatkowski, Cordelia Langford, John Sillitoe on behalf of the Wellcome Sanger Institute COVID-19 Surveillance Team
EPI_ISL_631145, EPI_ISL_631146	Lighthouse Lab in Milton Keynes	Wellcome Sanger Institute for the COVID-19 Genomics UK (COG-UK) consortium	The Lighthouse Lab in Milton Keynes and Alex Alderton, Roberto Amato, Sonia Goncalves, Ewan Harrison, David K. Jackson, Ian Johnston, Dominic Kwiatkowski, Cordelia Langford, John Sillitoe on behalf of the Wellcome Sanger Institute COVID-19 Surveillance Team
EPI_ISL_631148, EPI_ISL_631149	Lighthouse Lab in Cambridge	Wellcome Sanger Institute for the COVID-19 Genomics UK (COG-UK) consortium	Rob Howes, The Lighthouse Lab in Cambridge and Alex Alderton, Roberto Amato, Sonia Goncalves, Ewan Harrison, David K. Jackson, Ian Johnston, Dominic Kwiatkowski, Cordelia Langford, John Sillitoe on behalf of the Wellcome Sanger Institute COVID-19 Surveillance Team
EPI_ISL_631150	Lighthouse Lab in Alderley Park	Wellcome Sanger Institute for the COVID-19 Genomics UK (COG-UK) consortium	Jacquelyn Wynn, Mairead Hyland, The Lighthouse Lab in Alderley Park and Alex Alderton, Roberto Amato, Sonia Goncalves, Ewan Harrison, David K. Jackson, Ian Johnston, Dominic Kwiatkowski, Cordelia Langford, John Sillitoe on behalf of the Wellcome Sanger Institute COVID-19 Surveillance Team
EPI_ISL_631151, EPI_ISL_631152, EPI_ISL_631153	Lighthouse Lab in Milton Keynes	Wellcome Sanger Institute for the COVID-19 Genomics UK (COG-UK) consortium	The Lighthouse Lab in Milton Keynes and Alex Alderton, Roberto Amato, Sonia Goncalves, Ewan Harrison, David K. Jackson, Ian Johnston, Dominic Kwiatkowski, Cordelia Langford, John Sillitoe on behalf of the Wellcome Sanger Institute COVID-19 Surveillance Team
EPI_ISL_631154, EPI_ISL_631155	Lighthouse Lab in Cambridge	Wellcome Sanger Institute for the COVID-19 Genomics UK (COG-UK) consortium	Rob Howes, The Lighthouse Lab in Cambridge and Alex Alderton, Roberto Amato, Sonia Goncalves, Ewan Harrison, David K. Jackson, Ian Johnston, Dominic Kwiatkowski, Cordelia Langford, John Sillitoe on behalf of the Wellcome Sanger Institute COVID-19 Surveillance Team
EPI_ISL_631156	Lighthouse Lab in Milton Keynes	Wellcome Sanger Institute for the COVID-19 Genomics UK (COG-UK) consortium	The Lighthouse Lab in Milton Keynes and Alex Alderton, Roberto Amato, Sonia Goncalves, Ewan Harrison, David K. Jackson, Ian Johnston, Dominic Kwiatkowski, Cordelia Langford, John Sillitoe on behalf of the Wellcome Sanger Institute COVID-19 Surveillance Team
EPI_ISL_631160	Lighthouse Lab in Cambridge	Wellcome Sanger Institute for the COVID-19 Genomics UK (COG-UK) consortium	Rob Howes, The Lighthouse Lab in Cambridge and Alex Alderton, Roberto Amato, Sonia Goncalves, Ewan Harrison, David K. Jackson, Ian Johnston, Dominic Kwiatkowski, Cordelia Langford, John Sillitoe on behalf of the Wellcome Sanger Institute COVID-19 Surveillance Team
EPI_ISL_631161, EPI_ISL_631162	Lighthouse Lab in Milton Keynes	Wellcome Sanger Institute for the COVID-19 Genomics UK (COG-UK) consortium	The Lighthouse Lab in Milton Keynes and Alex Alderton, Roberto Amato, Sonia Goncalves, Ewan Harrison, David K. Jackson, Ian Johnston, Dominic Kwiatkowski, Cordelia Langford, John Sillitoe on behalf of the Wellcome Sanger Institute COVID-19 Surveillance Team
EPI_ISL_631163	Lighthouse Lab in Alderley Park	Wellcome Sanger Institute for the COVID-19 Genomics UK (COG-UK) consortium	Jacquelyn Wynn, Mairead Hyland, The Lighthouse Lab in Alderley Park and Alex Alderton, Roberto Amato, Sonia Goncalves, Ewan Harrison, David K. Jackson, Ian Johnston, Dominic Kwiatkowski, Cordelia Langford, John Sillitoe on behalf of the Wellcome Sanger Institute COVID-19 Surveillance Team
EPI_ISL_631164	Lighthouse Lab in Cambridge	Wellcome Sanger Institute for the COVID-19 Genomics UK (COG-UK) consortium	Rob Howes, The Lighthouse Lab in Cambridge and Alex Alderton, Roberto Amato, Sonia Goncalves, Ewan Harrison, David K. Jackson, Ian Johnston, Dominic Kwiatkowski, Cordelia Langford, John Sillitoe on behalf of the Wellcome Sanger Institute COVID-19 Surveillance Team
EPI_ISL_631165	Lighthouse Lab in Alderley Park	Wellcome Sanger Institute for the COVID-19 Genomics UK (COG-UK) consortium	Jacquelyn Wynn, Mairead Hyland, The Lighthouse Lab in Alderley Park and Alex Alderton, Roberto Amato, Sonia Goncalves, Ewan Harrison, David K. Jackson, Ian Johnston, Dominic Kwiatkowski, Cordelia Langford, John Sillitoe on behalf of the Wellcome Sanger Institute COVID-19 Surveillance Team
EPI_ISL_631166, EPI_ISL_631167, EPI_ISL_631168, EPI_ISL_631169, EPI_ISL_631170	Lighthouse Lab in Milton Keynes	Wellcome Sanger Institute for the COVID-19 Genomics UK (COG-UK) consortium	The Lighthouse Lab in Milton Keynes and Alex Alderton, Roberto Amato, Sonia Goncalves, Ewan Harrison, David K. Jackson, Ian Johnston, Dominic Kwiatkowski, Cordelia Langford, John Sillitoe on behalf of the Wellcome Sanger Institute COVID-19 Surveillance Team
EPI_ISL_631171	Lighthouse Lab in Cambridge	Wellcome Sanger Institute for the COVID-19 Genomics UK (COG-UK) consortium	Rob Howes, The Lighthouse Lab in Cambridge and Alex Alderton, Roberto Amato, Sonia Goncalves, Ewan Harrison, David K. Jackson, Ian Johnston, Dominic Kwiatkowski, Cordelia Langford, John Sillitoe on behalf of the Wellcome Sanger Institute COVID-19 Surveillance Team
EPI_ISL_631172	Lighthouse Lab in Milton Keynes	Wellcome Sanger Institute for the COVID-19 Genomics UK (COG-UK) consortium	The Lighthouse Lab in Milton Keynes and Alex Alderton, Roberto Amato, Sonia Goncalves, Ewan Harrison, David K. Jackson, Ian Johnston, Dominic Kwiatkowski, Cordelia Langford, John Sillitoe on behalf of the Wellcome Sanger Institute COVID-19 Surveillance Team
EPI_ISL_631173	Lighthouse Lab in Alderley Park	Wellcome Sanger Institute for the COVID-19 Genomics UK (COG-UK) consortium	Jacquelyn Wynn, Mairead Hyland, The Lighthouse Lab in Alderley Park and Alex Alderton, Roberto Amato, Sonia Goncalves, Ewan Harrison, David K. Jackson, Ian Johnston, Dominic Kwiatkowski, Cordelia Langford, John Sillitoe on behalf of the Wellcome Sanger Institute COVID-19 Surveillance Team
EPI_ISL_631174	Lighthouse Lab in Cambridge	Wellcome Sanger Institute for the COVID-19 Genomics UK (COG-UK) consortium	Rob Howes, The Lighthouse Lab in Cambridge and Alex Alderton, Roberto Amato, Sonia Goncalves, Ewan Harrison, David K. Jackson, Ian Johnston, Dominic Kwiatkowski, Cordelia Langford, John Sillitoe on behalf of the Wellcome Sanger Institute COVID-19 Surveillance Team
EPI_ISL_631175	Lighthouse Lab in Alderley Park	Wellcome Sanger Institute for the COVID-19 Genomics UK (COG-UK) consortium	Jacquelyn Wynn, Mairead Hyland, The Lighthouse Lab in Alderley Park and Alex Alderton, Roberto Amato, Sonia Goncalves, Ewan Harrison, David K. Jackson, Ian Johnston, Dominic Kwiatkowski, Cordelia Langford, John Sillitoe on behalf of the Wellcome Sanger Institute COVID-19 Surveillance Team
EPI_ISL_631176	Lighthouse Lab in Milton Keynes	Wellcome Sanger Institute for the COVID-19 Genomics UK (COG-UK) consortium	The Lighthouse Lab in Milton Keynes and Alex Alderton, Roberto Amato, Sonia Goncalves, Ewan Harrison, David K. Jackson, Ian Johnston, Dominic Kwiatkowski, Cordelia Langford, John Sillitoe on behalf of the Wellcome Sanger Institute COVID-19 Surveillance Team
EPI_ISL_631177	Lighthouse Lab in Cambridge	Wellcome Sanger Institute for the COVID-19 Genomics UK (COG-UK) consortium	Rob Howes, The Lighthouse Lab in Cambridge and Alex Alderton, Roberto Amato, Sonia Goncalves, Ewan Harrison, David K. Jackson, Ian Johnston, Dominic Kwiatkowski, Cordelia Langford, John Sillitoe on behalf of the Wellcome Sanger Institute COVID-19 Surveillance Team
EPI_ISL_631178, EPI_ISL_631179	Lighthouse Lab in Milton Keynes	Wellcome Sanger Institute for the COVID-19 Genomics UK (COG-UK) consortium	The Lighthouse Lab in Milton Keynes and Alex Alderton, Roberto Amato, Sonia Goncalves, Ewan Harrison, David K. Jackson, Ian Johnston, Dominic Kwiatkowski, Cordelia Langford, John Sillitoe on behalf of the Wellcome Sanger Institute COVID-19 Surveillance Team
EPI_ISL_631180, EPI_ISL_631181	Lighthouse Lab in Cambridge	Wellcome Sanger Institute for the COVID-19 Genomics UK (COG-UK) consortium	Rob Howes, The Lighthouse Lab in Cambridge and Alex Alderton, Roberto Amato, Sonia Goncalves, Ewan Harrison, David K. Jackson, Ian Johnston, Dominic Kwiatkowski, Cordelia Langford, John Sillitoe on behalf of the Wellcome Sanger Institute COVID-19 Surveillance Team
EPI_ISL_631182, EPI_ISL_631183	Lighthouse Lab in Milton Keynes	Wellcome Sanger Institute for the COVID-19 Genomics UK (COG-UK) consortium	The Lighthouse Lab in Milton Keynes and Alex Alderton, Roberto Amato, Sonia Goncalves, Ewan Harrison, David K. Jackson, Ian Johnston, Dominic Kwiatkowski, Cordelia Langford, John Sillitoe on behalf of the Wellcome Sanger Institute COVID-19 Surveillance Team
EPI_ISL_631184	Lighthouse Lab in Alderley Park	Wellcome Sanger Institute for the COVID-19 Genomics UK (COG-UK) consortium	Jacquelyn Wynn, Mairead Hyland, The Lighthouse Lab in Alderley Park and Alex Alderton, Roberto Amato, Sonia Goncalves, Ewan Harrison, David K. Jackson, Ian Johnston, Dominic Kwiatkowski, Cordelia Langford, John Sillitoe on behalf of the Wellcome Sanger Institute COVID-19 Surveillance Team
EPI_ISL_631185	Lighthouse Lab in Cambridge	Wellcome Sanger Institute for the COVID-19 Genomics UK (COG-UK) consortium	Rob Howes, The Lighthouse Lab in Cambridge and Alex Alderton, Roberto Amato, Sonia Goncalves, Ewan Harrison, David K. Jackson, Ian Johnston, Dominic Kwiatkowski, Cordelia Langford, John Sillitoe on behalf of the Wellcome Sanger Institute COVID-19 Surveillance Team
EPI_ISL_631186, EPI_ISL_631187, EPI_ISL_631188	Lighthouse Lab in Milton Keynes	Wellcome Sanger Institute for the COVID-19 Genomics UK (COG-UK) consortium	The Lighthouse Lab in Milton Keynes and Alex Alderton, Roberto Amato, Sonia Goncalves, Ewan Harrison, David K. Jackson, Ian Johnston, Dominic Kwiatkowski, Cordelia Langford, John Sillitoe on behalf of the Wellcome Sanger Institute COVID-19 Surveillance Team
EPI_ISL_631189, EPI_ISL_631190	Lighthouse Lab in Alderley Park	Wellcome Sanger Institute for the COVID-19 Genomics UK (COG-UK) consortium	Jacquelyn Wynn, Mairead Hyland, The Lighthouse Lab in Alderley Park and Alex Alderton, Roberto Amato, Sonia Goncalves, Ewan Harrison, David K. Jackson, Ian Johnston, Dominic Kwiatkowski, Cordelia Langford, John Sillitoe on behalf of the Wellcome Sanger Institute COVID-19 Surveillance Team

EPI_ISL_631191	Lighthouse Lab in Cambridge	Wellcome Sanger Institute for the COVID-19 Genomics UK (COG-UK) consortium	Rob Howes, The Lighthouse Lab in Cambridge and Alex Alderton, Roberto Amato, Sonia Goncalves, Ewan Harrison, David K. Jackson, Ian Johnston, Dominic Kwiatkowski, Cordelia Langford, John Sillitoe on behalf of the Wellcome Sanger Institute COVID-19 Surveillance Team
EPI_ISL_631193	Lighthouse Lab in Milton Keynes	Wellcome Sanger Institute for the COVID-19 Genomics UK (COG-UK) consortium	The Lighthouse Lab in Milton Keynes and Alex Alderton, Roberto Amato, Sonia Goncalves, Ewan Harrison, David K. Jackson, Ian Johnston, Dominic Kwiatkowski, Cordelia Langford, John Sillitoe on behalf of the Wellcome Sanger Institute COVID-19 Surveillance Team
EPI_ISL_631194	Lighthouse Lab in Cambridge	Wellcome Sanger Institute for the COVID-19 Genomics UK (COG-UK) consortium	Rob Howes, The Lighthouse Lab in Cambridge and Alex Alderton, Roberto Amato, Sonia Goncalves, Ewan Harrison, David K. Jackson, Ian Johnston, Dominic Kwiatkowski, Cordelia Langford, John Sillitoe on behalf of the Wellcome Sanger Institute COVID-19 Surveillance Team
EPI_ISL_631196, EPI_ISL_631197, EPI_ISL_631198	Lighthouse Lab in Milton Keynes	Wellcome Sanger Institute for the COVID-19 Genomics UK (COG-UK) consortium	The Lighthouse Lab in Milton Keynes and Alex Alderton, Roberto Amato, Sonia Goncalves, Ewan Harrison, David K. Jackson, Ian Johnston, Dominic Kwiatkowski, Cordelia Langford, John Sillitoe on behalf of the Wellcome Sanger Institute COVID-19 Surveillance Team
EPI_ISL_631199	Lighthouse Lab in Cambridge	Wellcome Sanger Institute for the COVID-19 Genomics UK (COG-UK) consortium	Rob Howes, The Lighthouse Lab in Cambridge and Alex Alderton, Roberto Amato, Sonia Goncalves, Ewan Harrison, David K. Jackson, Ian Johnston, Dominic Kwiatkowski, Cordelia Langford, John Sillitoe on behalf of the Wellcome Sanger Institute COVID-19 Surveillance Team
EPI_ISL_631200, EPI_ISL_631201	Lighthouse Lab in Milton Keynes	Wellcome Sanger Institute for the COVID-19 Genomics UK (COG-UK) consortium	The Lighthouse Lab in Milton Keynes and Alex Alderton, Roberto Amato, Sonia Goncalves, Ewan Harrison, David K. Jackson, Ian Johnston, Dominic Kwiatkowski, Cordelia Langford, John Sillitoe on behalf of the Wellcome Sanger Institute COVID-19 Surveillance Team
EPI_ISL_631202	Lighthouse Lab in Alderley Park	Wellcome Sanger Institute for the COVID-19 Genomics UK (COG-UK) consortium	Jacquelyn Wynn, Mairead Hyland, The Lighthouse Lab in Alderley Park and Alex Alderton, Roberto Amato, Sonia Goncalves, Ewan Harrison, David K. Jackson, Ian Johnston, Dominic Kwiatkowski, Cordelia Langford, John Sillitoe on behalf of the Wellcome Sanger Institute COVID-19 Surveillance Team
EPI_ISL_631204	Lighthouse Lab in Cambridge	Wellcome Sanger Institute for the COVID-19 Genomics UK (COG-UK) consortium	Rob Howes, The Lighthouse Lab in Cambridge and Alex Alderton, Roberto Amato, Sonia Goncalves, Ewan Harrison, David K. Jackson, Ian Johnston, Dominic Kwiatkowski, Cordelia Langford, John Sillitoe on behalf of the Wellcome Sanger Institute COVID-19 Surveillance Team
EPI_ISL_631205, EPI_ISL_631206, EPI_ISL_631207	Lighthouse Lab in Milton Keynes	Wellcome Sanger Institute for the COVID-19 Genomics UK (COG-UK) consortium	The Lighthouse Lab in Milton Keynes and Alex Alderton, Roberto Amato, Sonia Goncalves, Ewan Harrison, David K. Jackson, Ian Johnston, Dominic Kwiatkowski, Cordelia Langford, John Sillitoe on behalf of the Wellcome Sanger Institute COVID-19 Surveillance Team
EPI_ISL_631208	Lighthouse Lab in Cambridge	Wellcome Sanger Institute for the COVID-19 Genomics UK (COG-UK) consortium	Rob Howes, The Lighthouse Lab in Cambridge and Alex Alderton, Roberto Amato, Sonia Goncalves, Ewan Harrison, David K. Jackson, Ian Johnston, Dominic Kwiatkowski, Cordelia Langford, John Sillitoe on behalf of the Wellcome Sanger Institute COVID-19 Surveillance Team
EPI_ISL_631209, EPI_ISL_631210, EPI_ISL_631211	Lighthouse Lab in Milton Keynes	Wellcome Sanger Institute for the COVID-19 Genomics UK (COG-UK) consortium	The Lighthouse Lab in Milton Keynes and Alex Alderton, Roberto Amato, Sonia Goncalves, Ewan Harrison, David K. Jackson, Ian Johnston, Dominic Kwiatkowski, Cordelia Langford, John Sillitoe on behalf of the Wellcome Sanger Institute COVID-19 Surveillance Team
EPI_ISL_631212	Lighthouse Lab in Alderley Park	Wellcome Sanger Institute for the COVID-19 Genomics UK (COG-UK) consortium	Jacquelyn Wynn, Mairead Hyland, The Lighthouse Lab in Alderley Park and Alex Alderton, Roberto Amato, Sonia Goncalves, Ewan Harrison, David K. Jackson, Ian Johnston, Dominic Kwiatkowski, Cordelia Langford, John Sillitoe on behalf of the Wellcome Sanger Institute COVID-19 Surveillance Team
EPI_ISL_631213, EPI_ISL_631214	Lighthouse Lab in Milton Keynes	Wellcome Sanger Institute for the COVID-19 Genomics UK (COG-UK) consortium	The Lighthouse Lab in Milton Keynes and Alex Alderton, Roberto Amato, Sonia Goncalves, Ewan Harrison, David K. Jackson, Ian Johnston, Dominic Kwiatkowski, Cordelia Langford, John Sillitoe on behalf of the Wellcome Sanger Institute COVID-19 Surveillance Team
EPI_ISL_631215	Lighthouse Lab in Alderley Park	Wellcome Sanger Institute for the COVID-19 Genomics UK (COG-UK) consortium	Jacquelyn Wynn, Mairead Hyland, The Lighthouse Lab in Alderley Park and Alex Alderton, Roberto Amato, Sonia Goncalves, Ewan Harrison, David K. Jackson, Ian Johnston, Dominic Kwiatkowski, Cordelia Langford, John Sillitoe on behalf of the Wellcome Sanger Institute COVID-19 Surveillance Team
EPI_ISL_631216	Lighthouse Lab in Milton Keynes	Wellcome Sanger Institute for the COVID-19 Genomics UK (COG-UK) consortium	The Lighthouse Lab in Milton Keynes and Alex Alderton, Roberto Amato, Sonia Goncalves, Ewan Harrison, David K. Jackson, Ian Johnston, Dominic Kwiatkowski, Cordelia Langford, John Sillitoe on behalf of the Wellcome Sanger Institute COVID-19 Surveillance Team
EPI_ISL_631217	Lighthouse Lab in Alderley Park	Wellcome Sanger Institute for the COVID-19 Genomics UK (COG-UK) consortium	Jacquelyn Wynn, Mairead Hyland, The Lighthouse Lab in Alderley Park and Alex Alderton, Roberto Amato, Sonia Goncalves, Ewan Harrison, David K. Jackson, Ian Johnston, Dominic Kwiatkowski, Cordelia Langford, John Sillitoe on behalf of the Wellcome Sanger Institute COVID-19 Surveillance Team
EPI_ISL_631218	Lighthouse Lab in Milton Keynes	Wellcome Sanger Institute for the COVID-19 Genomics UK (COG-UK) consortium	The Lighthouse Lab in Milton Keynes and Alex Alderton, Roberto Amato, Sonia Goncalves, Ewan Harrison, David K. Jackson, Ian Johnston, Dominic Kwiatkowski, Cordelia Langford, John Sillitoe on behalf of the Wellcome Sanger Institute COVID-19 Surveillance Team
EPI_ISL_631219, EPI_ISL_631220, EPI_ISL_631221	Lighthouse Lab in Cambridge	Wellcome Sanger Institute for the COVID-19 Genomics UK (COG-UK) consortium	Rob Howes, The Lighthouse Lab in Cambridge and Alex Alderton, Roberto Amato, Sonia Goncalves, Ewan Harrison, David K. Jackson, Ian Johnston, Dominic Kwiatkowski, Cordelia Langford, John Sillitoe on behalf of the Wellcome Sanger Institute COVID-19 Surveillance Team
EPI_ISL_631222	Lighthouse Lab in Alderley Park	Wellcome Sanger Institute for the COVID-19 Genomics UK (COG-UK) consortium	Jacquelyn Wynn, Mairead Hyland, The Lighthouse Lab in Alderley Park and Alex Alderton, Roberto Amato, Sonia Goncalves, Ewan Harrison, David K. Jackson, Ian Johnston, Dominic Kwiatkowski, Cordelia Langford, John Sillitoe on behalf of the Wellcome Sanger Institute COVID-19 Surveillance Team
EPI_ISL_631223	Lighthouse Lab in Milton Keynes	Wellcome Sanger Institute for the COVID-19 Genomics UK (COG-UK) consortium	The Lighthouse Lab in Milton Keynes and Alex Alderton, Roberto Amato, Sonia Goncalves, Ewan Harrison, David K. Jackson, Ian Johnston, Dominic Kwiatkowski, Cordelia Langford, John Sillitoe on behalf of the Wellcome Sanger Institute COVID-19 Surveillance Team
EPI_ISL_631224, EPI_ISL_631225	Lighthouse Lab in Cambridge	Wellcome Sanger Institute for the COVID-19 Genomics UK (COG-UK) consortium	Rob Howes, The Lighthouse Lab in Cambridge and Alex Alderton, Roberto Amato, Sonia Goncalves, Ewan Harrison, David K. Jackson, Ian Johnston, Dominic Kwiatkowski, Cordelia Langford, John Sillitoe on behalf of the Wellcome Sanger Institute COVID-19 Surveillance Team
EPI_ISL_631226, EPI_ISL_631227	Lighthouse Lab in Milton Keynes	Wellcome Sanger Institute for the COVID-19 Genomics UK (COG-UK) consortium	The Lighthouse Lab in Milton Keynes and Alex Alderton, Roberto Amato, Sonia Goncalves, Ewan Harrison, David K. Jackson, Ian Johnston, Dominic Kwiatkowski, Cordelia Langford, John Sillitoe on behalf of the Wellcome Sanger Institute COVID-19 Surveillance Team
EPI_ISL_631228	Lighthouse Lab in Alderley Park	Wellcome Sanger Institute for the COVID-19 Genomics UK (COG-UK) consortium	Jacquelyn Wynn, Mairead Hyland, The Lighthouse Lab in Alderley Park and Alex Alderton, Roberto Amato, Sonia Goncalves, Ewan Harrison, David K. Jackson, Ian Johnston, Dominic Kwiatkowski, Cordelia Langford, John Sillitoe on behalf of the Wellcome Sanger Institute COVID-19 Surveillance Team
EPI_ISL_631229, EPI_ISL_631230	Lighthouse Lab in Milton Keynes	Wellcome Sanger Institute for the COVID-19 Genomics UK (COG-UK) consortium	The Lighthouse Lab in Milton Keynes and Alex Alderton, Roberto Amato, Sonia Goncalves, Ewan Harrison, David K. Jackson, Ian Johnston, Dominic Kwiatkowski, Cordelia Langford, John Sillitoe on behalf of the Wellcome Sanger Institute COVID-19 Surveillance Team
EPI_ISL_631231	Lighthouse Lab in Alderley Park	Wellcome Sanger Institute for the COVID-19 Genomics UK (COG-UK) consortium	Jacquelyn Wynn, Mairead Hyland, The Lighthouse Lab in Alderley Park and Alex Alderton, Roberto Amato, Sonia Goncalves, Ewan Harrison, David K. Jackson, Ian Johnston, Dominic Kwiatkowski, Cordelia Langford, John Sillitoe on behalf of the Wellcome Sanger Institute COVID-19 Surveillance Team
EPI_ISL_631232, EPI_ISL_631233, EPI_ISL_631234	Lighthouse Lab in Milton Keynes	Wellcome Sanger Institute for the COVID-19 Genomics UK (COG-UK) consortium	The Lighthouse Lab in Milton Keynes and Alex Alderton, Roberto Amato, Sonia Goncalves, Ewan Harrison, David K. Jackson, Ian Johnston, Dominic Kwiatkowski, Cordelia Langford, John Sillitoe on behalf of the Wellcome Sanger Institute COVID-19 Surveillance Team
EPI_ISL_631235	Lighthouse Lab in Cambridge	Wellcome Sanger Institute for the COVID-19 Genomics UK (COG-UK) consortium	Rob Howes, The Lighthouse Lab in Cambridge and Alex Alderton, Roberto Amato, Sonia Goncalves, Ewan Harrison, David K. Jackson, Ian Johnston, Dominic Kwiatkowski, Cordelia Langford, John Sillitoe on behalf of the Wellcome Sanger Institute COVID-19 Surveillance Team
EPI_ISL_631236	Lighthouse Lab in Milton Keynes	Wellcome Sanger Institute for the COVID-19 Genomics UK (COG-UK) consortium	The Lighthouse Lab in Milton Keynes and Alex Alderton, Roberto Amato, Sonia Goncalves, Ewan Harrison, David K. Jackson, Ian Johnston, Dominic Kwiatkowski, Cordelia Langford, John Sillitoe on behalf of the Wellcome Sanger Institute COVID-19 Surveillance Team
EPI_ISL_631237	Lighthouse Lab in Cambridge	Wellcome Sanger Institute for the COVID-19 Genomics UK (COG-UK) consortium	Rob Howes, The Lighthouse Lab in Cambridge and Alex Alderton, Roberto Amato, Sonia Goncalves, Ewan Harrison, David K. Jackson, Ian Johnston, Dominic Kwiatkowski, Cordelia Langford, John Sillitoe on behalf of the Wellcome Sanger Institute COVID-19 Surveillance Team
EPI_ISL_631238, EPI_ISL_631240	Lighthouse Lab in Milton Keynes	Wellcome Sanger Institute for the COVID-19 Genomics UK (COG-UK) consortium	The Lighthouse Lab in Milton Keynes and Alex Alderton, Roberto Amato, Sonia Goncalves, Ewan Harrison, David K. Jackson, Ian Johnston, Dominic Kwiatkowski, Cordelia Langford, John Sillitoe on behalf of the Wellcome Sanger Institute COVID-19 Surveillance Team
EPI_ISL_631241	Lighthouse Lab in Alderley Park	Wellcome Sanger Institute for the COVID-19 Genomics UK (COG-UK) consortium	Jacquelyn Wynn, Mairead Hyland, The Lighthouse Lab in Alderley Park and Alex Alderton, Roberto Amato, Sonia Goncalves, Ewan Harrison, David K. Jackson, Ian Johnston, Dominic Kwiatkowski, Cordelia Langford, John Sillitoe on behalf of the Wellcome Sanger Institute COVID-19 Surveillance Team
EPI_ISL_631242, EPI_ISL_631243, EPI_ISL_631244	Lighthouse Lab in Milton Keynes	Wellcome Sanger Institute for the COVID-19 Genomics UK (COG-UK) consortium	The Lighthouse Lab in Milton Keynes and Alex Alderton, Roberto Amato, Sonia Goncalves, Ewan Harrison, David K. Jackson, Ian Johnston, Dominic Kwiatkowski, Cordelia Langford, John Sillitoe on behalf of the Wellcome Sanger Institute COVID-19 Surveillance Team
EPI_ISL_631245	Lighthouse Lab in Cambridge	Wellcome Sanger Institute for the COVID-19 Genomics UK (COG-UK) consortium	Rob Howes, The Lighthouse Lab in Cambridge and Alex Alderton, Roberto Amato, Sonia Goncalves, Ewan Harrison, David K. Jackson, Ian Johnston, Dominic Kwiatkowski, Cordelia Langford, John Sillitoe on behalf of the Wellcome Sanger Institute COVID-19 Surveillance Team
EPI_ISL_631246, EPI_ISL_631247, EPI_ISL_631248, EPI_ISL_631249	Lighthouse Lab in Milton Keynes	Wellcome Sanger Institute for the COVID-19 Genomics UK (COG-UK) consortium	The Lighthouse Lab in Milton Keynes and Alex Alderton, Roberto Amato, Sonia Goncalves, Ewan Harrison, David K. Jackson, Ian Johnston, Dominic Kwiatkowski, Cordelia Langford, John Sillitoe on behalf of the Wellcome Sanger Institute COVID-19 Surveillance Team
EPI_ISL_631250	Lighthouse Lab in Alderley Park	Wellcome Sanger Institute for the COVID-19 Genomics UK (COG-UK) consortium	Jacquelyn Wynn, Mairead Hyland, The Lighthouse Lab in Alderley Park and Alex Alderton, Roberto Amato, Sonia Goncalves, Ewan Harrison, David K. Jackson, Ian Johnston, Dominic Kwiatkowski, Cordelia Langford, John Sillitoe on behalf of the Wellcome Sanger Institute COVID-19 Surveillance Team

EPI_ISL_631251, EPI_ISL_631252, EPI_ISL_631253	Lighthouse Lab in Milton Keynes	Wellcome Sanger Institute for the COVID-19 Genomics UK (COG-UK) consortium	The Lighthouse Lab in Milton Keynes and Alex Alderton, Roberto Amato, Sonia Goncalves, Ewan Harrison, David K. Jackson, Ian Johnston, Dominic Kwiatkowski, Cordelia Langford, John Sillitoe on behalf of the Wellcome Sanger Institute COVID-19 Surveillance Team
EPI_ISL_631254	Lighthouse Lab in Cambridge	Wellcome Sanger Institute for the COVID-19 Genomics UK (COG-UK) consortium	Rob Howes, The Lighthouse Lab in Cambridge and Alex Alderton, Roberto Amato, Sonia Goncalves, Ewan Harrison, David K. Jackson, Ian Johnston, Dominic Kwiatkowski, Cordelia Langford, John Sillitoe on behalf of the Wellcome Sanger Institute COVID-19 Surveillance Team
EPI_ISL_631255, EPI_ISL_631256	Lighthouse Lab in Milton Keynes	Wellcome Sanger Institute for the COVID-19 Genomics UK (COG-UK) consortium	The Lighthouse Lab in Milton Keynes and Alex Alderton, Roberto Amato, Sonia Goncalves, Ewan Harrison, David K. Jackson, Ian Johnston, Dominic Kwiatkowski, Cordelia Langford, John Sillitoe on behalf of the Wellcome Sanger Institute COVID-19 Surveillance Team
EPI_ISL_631257	Lighthouse Lab in Cambridge	Wellcome Sanger Institute for the COVID-19 Genomics UK (COG-UK) consortium	Rob Howes, The Lighthouse Lab in Cambridge and Alex Alderton, Roberto Amato, Sonia Goncalves, Ewan Harrison, David K. Jackson, Ian Johnston, Dominic Kwiatkowski, Cordelia Langford, John Sillitoe on behalf of the Wellcome Sanger Institute COVID-19 Surveillance Team
EPI_ISL_631258, EPI_ISL_631259, EPI_ISL_631260, EPI_ISL_631261	Lighthouse Lab in Milton Keynes	Wellcome Sanger Institute for the COVID-19 Genomics UK (COG-UK) consortium	The Lighthouse Lab in Milton Keynes and Alex Alderton, Roberto Amato, Sonia Goncalves, Ewan Harrison, David K. Jackson, Ian Johnston, Dominic Kwiatkowski, Cordelia Langford, John Sillitoe on behalf of the Wellcome Sanger Institute COVID-19 Surveillance Team
EPI_ISL_631262	Lighthouse Lab in Cambridge	Wellcome Sanger Institute for the COVID-19 Genomics UK (COG-UK) consortium	Rob Howes, The Lighthouse Lab in Cambridge and Alex Alderton, Roberto Amato, Sonia Goncalves, Ewan Harrison, David K. Jackson, Ian Johnston, Dominic Kwiatkowski, Cordelia Langford, John Sillitoe on behalf of the Wellcome Sanger Institute COVID-19 Surveillance Team
EPI_ISL_631264	Lighthouse Lab in Milton Keynes	Wellcome Sanger Institute for the COVID-19 Genomics UK (COG-UK) consortium	The Lighthouse Lab in Milton Keynes and Alex Alderton, Roberto Amato, Sonia Goncalves, Ewan Harrison, David K. Jackson, Ian Johnston, Dominic Kwiatkowski, Cordelia Langford, John Sillitoe on behalf of the Wellcome Sanger Institute COVID-19 Surveillance Team
EPI_ISL_631265	Lighthouse Lab in Alderley Park	Wellcome Sanger Institute for the COVID-19 Genomics UK (COG-UK) consortium	Jacquelyn Wynn, Mairead Hyland, The Lighthouse Lab in Alderley Park and Alex Alderton, Roberto Amato, Sonia Goncalves, Ewan Harrison, David K. Jackson, Ian Johnston, Dominic Kwiatkowski, Cordelia Langford, John Sillitoe on behalf of the Wellcome Sanger Institute COVID-19 Surveillance Team
EPI_ISL_631266	Lighthouse Lab in Cambridge	Wellcome Sanger Institute for the COVID-19 Genomics UK (COG-UK) consortium	Rob Howes, The Lighthouse Lab in Cambridge and Alex Alderton, Roberto Amato, Sonia Goncalves, Ewan Harrison, David K. Jackson, Ian Johnston, Dominic Kwiatkowski, Cordelia Langford, John Sillitoe on behalf of the Wellcome Sanger Institute COVID-19 Surveillance Team
EPI_ISL_631267	Lighthouse Lab in Milton Keynes	Wellcome Sanger Institute for the COVID-19 Genomics UK (COG-UK) consortium	The Lighthouse Lab in Milton Keynes and Alex Alderton, Roberto Amato, Sonia Goncalves, Ewan Harrison, David K. Jackson, Ian Johnston, Dominic Kwiatkowski, Cordelia Langford, John Sillitoe on behalf of the Wellcome Sanger Institute COVID-19 Surveillance Team
EPI_ISL_631268	Lighthouse Lab in Cambridge	Wellcome Sanger Institute for the COVID-19 Genomics UK (COG-UK) consortium	Rob Howes, The Lighthouse Lab in Cambridge and Alex Alderton, Roberto Amato, Sonia Goncalves, Ewan Harrison, David K. Jackson, Ian Johnston, Dominic Kwiatkowski, Cordelia Langford, John Sillitoe on behalf of the Wellcome Sanger Institute COVID-19 Surveillance Team
EPI_ISL_631269	Lighthouse Lab in Alderley Park	Wellcome Sanger Institute for the COVID-19 Genomics UK (COG-UK) consortium	Jacquelyn Wynn, Mairead Hyland, The Lighthouse Lab in Alderley Park and Alex Alderton, Roberto Amato, Sonia Goncalves, Ewan Harrison, David K. Jackson, Ian Johnston, Dominic Kwiatkowski, Cordelia Langford, John Sillitoe on behalf of the Wellcome Sanger Institute COVID-19 Surveillance Team
EPI_ISL_631270, EPI_ISL_631271, EPI_ISL_631272, EPI_ISL_631273, EPI_ISL_631274	Lighthouse Lab in Cambridge	Wellcome Sanger Institute for the COVID-19 Genomics UK (COG-UK) consortium	Rob Howes, The Lighthouse Lab in Cambridge and Alex Alderton, Roberto Amato, Sonia Goncalves, Ewan Harrison, David K. Jackson, Ian Johnston, Dominic Kwiatkowski, Cordelia Langford, John Sillitoe on behalf of the Wellcome Sanger Institute COVID-19 Surveillance Team
EPI_ISL_631275	Lighthouse Lab in Milton Keynes	Wellcome Sanger Institute for the COVID-19 Genomics UK (COG-UK) consortium	The Lighthouse Lab in Milton Keynes and Alex Alderton, Roberto Amato, Sonia Goncalves, Ewan Harrison, David K. Jackson, Ian Johnston, Dominic Kwiatkowski, Cordelia Langford, John Sillitoe on behalf of the Wellcome Sanger Institute COVID-19 Surveillance Team
EPI_ISL_631277, EPI_ISL_631278, EPI_ISL_631279, EPI_ISL_631280, EPI_ISL_631281, EPI_ISL_631282, EPI_ISL_631283, EPI_ISL_631284, EPI_ISL_631285, EPI_ISL_631286, EPI_ISL_631287, EPI_ISL_631288, EPI_ISL_631289, EPI_ISL_631290, EPI_ISL_631291, EPI_ISL_631292, EPI_ISL_631293, EPI_ISL_631294, EPI_ISL_631295, EPI_ISL_631296, EPI_ISL_631297, EPI_ISL_631298, EPI_ISL_631299, EPI_ISL_631300, EPI_ISL_631301, EPI_ISL_631302, EPI_ISL_631303			
see above	MVZ DIAMEDIS Diagnostische Medizin Sennestadt GmbH	Bielefeld University	David Brandt, Tobias Busche, Markus Haak, Jörn Kalinowski, Levin-Joe Klages, Christiane Scherer, Alexander Sczyrba, Marina Simunovic, Svenja Vinke
EPI_ISL_631304, EPI_ISL_631305, EPI_ISL_631306, EPI_ISL_631307, EPI_ISL_631308, EPI_ISL_631309, EPI_ISL_631310, EPI_ISL_631311, EPI_ISL_631312, EPI_ISL_631313, EPI_ISL_631314, EPI_ISL_631315, EPI_ISL_631316, EPI_ISL_631317, EPI_ISL_631318, EPI_ISL_631319, EPI_ISL_631320, EPI_ISL_631321, EPI_ISL_631322, EPI_ISL_631324, EPI_ISL_631344, EPI_ISL_631345, EPI_ISL_631346, EPI_ISL_631348, EPI_ISL_631349, EPI_ISL_631350, EPI_ISL_631351, EPI_ISL_631352, EPI_ISL_631353, EPI_ISL_631354, EPI_ISL_631355, EPI_ISL_631356, EPI_ISL_631357, EPI_ISL_631358, EPI_ISL_631359, EPI_ISL_631360, EPI_ISL_631361, EPI_ISL_631362, EPI_ISL_631363, EPI_ISL_631364, EPI_ISL_631365, EPI_ISL_631366, EPI_ISL_631367, EPI_ISL_631368, EPI_ISL_631369, EPI_ISL_631370, EPI_ISL_631371, EPI_ISL_631372, EPI_ISL_631373, EPI_ISL_631374, EPI_ISL_631375, EPI_ISL_631376			
see above	ZOTZ KLIMAS MVZ Düsseldorf-Centrum GbR ÜBAG für Labormedizin, Genetik, Zytologie, Pathologie	Center of Medical Microbiology, Virology, and Hospital Hygiene, University of Duesseldorf	Maximilian Damagnez, Alexander Dilthey, Ashley-Jane Duplessis, Patrick Finzer, Katrin Hoffmann, Torsten Houwaart, Lisanna Hülse, Malte Kohns Vasconcelos, Marek Korencak, Nadine Lübke, Jessica Nicolai, Klaus Pfeffer, Daniel Strelow, Jörg Timm, Andreas Walker, Tobias Wienemann, Rainer Zotz
EPI_ISL_631377, EPI_ISL_631378, EPI_ISL_631379, EPI_ISL_631380, EPI_ISL_631381, EPI_ISL_631382, EPI_ISL_631383, EPI_ISL_631384, EPI_ISL_631385, EPI_ISL_631386	University Hospital Cologne	Center of Medical Microbiology, Virology, and Hospital Hygiene, University of Duesseldorf	Maximilian Damagnez, Alexander Dilthey, Ashley-Jane Duplessis, Eva Heger, Torsten Houwaart, Rolf Kaiser, Florian Klein, Elena Knops, Malte Kohns Vasconcelos, Jessica Nicolai, Klaus Pfeffer, Gibran Rubio Quintanares, Saleta Sierra-Aragón, Daniel Strelow, Jörg Timm, Andreas Walker, Tobias Wienemann
EPI_ISL_631387	ZOTZ KLIMAS MVZ Düsseldorf-Centrum GbR ÜBAG für Labormedizin, Genetik, Zytologie, Pathologie	Center of Medical Microbiology, Virology, and Hospital Hygiene, University of Duesseldorf	Maximilian Damagnez, Alexander Dilthey, Ashley-Jane Duplessis, Patrick Finzer, Katrin Hoffmann, Torsten Houwaart, Lisanna Hülse, Malte Kohns Vasconcelos, Marek Korencak, Nadine Lübke, Jessica Nicolai, Klaus Pfeffer, Daniel Strelow, Jörg Timm, Andreas Walker, Tobias Wienemann, Rainer Zotz
EPI_ISL_631388	University Hospital Cologne	Center of Medical Microbiology, Virology, and Hospital Hygiene, University of Duesseldorf	Maximilian Damagnez, Alexander Dilthey, Ashley-Jane Duplessis, Eva Heger, Torsten Houwaart, Rolf Kaiser, Florian Klein, Elena Knops, Malte Kohns Vasconcelos, Jessica Nicolai, Klaus Pfeffer, Gibran Rubio Quintanares, Saleta Sierra-Aragón, Daniel Strelow, Jörg Timm, Andreas Walker, Tobias Wienemann
EPI_ISL_631389, EPI_ISL_631390, EPI_ISL_631391, EPI_ISL_631392, EPI_ISL_631393, EPI_ISL_631394, EPI_ISL_631395, EPI_ISL_631396, EPI_ISL_631397, EPI_ISL_631398, EPI_ISL_631399, EPI_ISL_631400, EPI_ISL_631401, EPI_ISL_631402, EPI_ISL_631404, EPI_ISL_631406, EPI_ISL_631407, EPI_ISL_631408, EPI_ISL_631409, EPI_ISL_631410, EPI_ISL_631412, EPI_ISL_631413, EPI_ISL_631414, EPI_ISL_631415, EPI_ISL_631416, EPI_ISL_631417, EPI_ISL_631418, EPI_ISL_631419, EPI_ISL_631420, EPI_ISL_631421, EPI_ISL_631422, EPI_ISL_631423, EPI_ISL_631424, EPI_ISL_631425, EPI_ISL_631426, EPI_ISL_631427, EPI_ISL_631428, EPI_ISL_631429, EPI_ISL_631430, EPI_ISL_631431, EPI_ISL_631432, EPI_ISL_631433, EPI_ISL_631434, EPI_ISL_631435, EPI_ISL_631436, EPI_ISL_631437, EPI_ISL_631438, EPI_ISL_631439, EPI_ISL_631440, EPI_ISL_631441, EPI_ISL_631442, EPI_ISL_631443, EPI_ISL_631444, EPI_ISL_631445, EPI_ISL_631446, EPI_ISL_631447, EPI_ISL_631448, EPI_ISL_631449, EPI_ISL_631450, EPI_ISL_631451, EPI_ISL_631452, EPI_ISL_631453, EPI_ISL_631454, EPI_ISL_631455, EPI_ISL_631456, EPI_ISL_631457, EPI_ISL_631458, EPI_ISL_631459, EPI_ISL_631460, EPI_ISL_631461, EPI_ISL_631462, EPI_ISL_631463, EPI_ISL_631464, EPI_ISL_631465, EPI_ISL_631466, EPI_ISL_631467, EPI_ISL_631468, EPI_ISL_631469, EPI_ISL_631470, EPI_ISL_631471, EPI_ISL_631472, EPI_ISL_631473, EPI_ISL_631474, EPI_ISL_631475, EPI_ISL_631476, EPI_ISL_631477, EPI_ISL_631478, EPI_ISL_631479, EPI_ISL_631480, EPI_ISL_631481, EPI_ISL_631482, EPI_ISL_631483, EPI_ISL_631484, EPI_ISL_631485, EPI_ISL_631486, EPI_ISL_631487, EPI_ISL_631488, EPI_ISL_631489, EPI_ISL_631490, EPI_ISL_631491, EPI_ISL_631492, EPI_ISL_631493, EPI_ISL_631494, EPI_ISL_631495, EPI_ISL_631496, EPI_ISL_631497, EPI_ISL_631498, EPI_ISL_631499, EPI_ISL_631500, EPI_ISL_631501			
see above	Wisconsin State Laboratory of Hygiene Communicable Disease Division	Wisconsin State Laboratory of Hygiene Communicable Disease Division	Kelsey R. Florek, Abigail C. Shockey
EPI_ISL_631502	New York-Presbyterian-Columbia University Medical Center	New York City Public Health Laboratory	Jade Wang, et al.
EPI_ISL_631504	Wyckoff Heights Medical Center	New York City Public Health Laboratory	Jade Wang, et al.
EPI_ISL_631505	Northwell Health-GoHealth Urgent Care 10025	New York City Public Health Laboratory	Jade Wang, et al.
EPI_ISL_631508	Brooklyn Hospital Center	New York City Public Health Laboratory	Jade Wang, et al.
EPI_ISL_631509	New York Presbyterian Lower Manhattan	New York City Public Health Laboratory	Jade Wang, et al.
EPI_ISL_631510, EPI_ISL_631511	New York Presbyterian/ Weill Cornell Medical Center	New York City Public Health Laboratory	Jade Wang, et al.
EPI_ISL_631512	Mount Sinai Hospital	New York City Public Health Laboratory	Jade Wang, et al.
EPI_ISL_631513	Montefiore Medical Center	New York City Public Health Laboratory	Jade Wang, et al.
EPI_ISL_631514	New York Community Hospital	New York City Public Health Laboratory	Jade Wang, et al.
EPI_ISL_631515	NYU Langone Health	New York City Public Health Laboratory	Jade Wang, et al.
EPI_ISL_631516	Jamaica Hospital Medical Center	New York City Public Health Laboratory	Jade Wang, et al.
EPI_ISL_631517, EPI_ISL_631518, EPI_ISL_631519	New York Presbyterian-Brooklyn Methodist Hospital	New York City Public Health Laboratory	Jade Wang, et al.
EPI_ISL_631520	NYC HH Lincoln Medical And Mental Health Center	New York City Public Health Laboratory	Jade Wang, et al.
EPI_ISL_631522	Robert Kutnick	New York City Public Health Laboratory	Jade Wang, et al.

EPI_ISL_631523	NYU Langone Health	New York City Public Health Laboratory	Jade Wang, et al.
EPI_ISL_631524	Jamaica Hospital Medical Center	New York City Public Health Laboratory	Jade Wang, et al.
EPI_ISL_631525	Mount Sinai Hospital	New York City Public Health Laboratory	Jade Wang, et al.
EPI_ISL_631526, EPI_ISL_631527	NYU Langone Health	New York City Public Health Laboratory	Jade Wang, et al.
EPI_ISL_631528	Maimonides Medical Center	New York City Public Health Laboratory	Jade Wang, et al.
EPI_ISL_631529	St. Johns Episcopal Hospital	New York City Public Health Laboratory	Jade Wang, et al.
EPI_ISL_631530	New York Presbyterian Queens	New York City Public Health Laboratory	Jade Wang, et al.
EPI_ISL_631531	NYU Langone Health	New York City Public Health Laboratory	Jade Wang, et al.
EPI_ISL_631532	Mount Sinai Hospital	New York City Public Health Laboratory	Jade Wang, et al.
EPI_ISL_631533	New York Presbyterian Lower Manhattan	New York City Public Health Laboratory	Jade Wang, et al.
EPI_ISL_631534	Mount Sinai Hospital	New York City Public Health Laboratory	Jade Wang, et al.
EPI_ISL_631536	New York Presbyterian Lower Manhattan	New York City Public Health Laboratory	Jade Wang, et al.
EPI_ISL_631537	Mount Sinai Hospital	New York City Public Health Laboratory	Jade Wang, et al.
EPI_ISL_631538	Maimonides Medical Center	New York City Public Health Laboratory	Jade Wang, et al.
EPI_ISL_631539, EPI_ISL_631540	New York Presbyterian Lower Manhattan	New York City Public Health Laboratory	Jade Wang, et al.
EPI_ISL_631541	NYC Department Of Health And Mental Hygiene	New York City Public Health Laboratory	Jade Wang, et al.
EPI_ISL_631542	New York Presbyterian Lower Manhattan	New York City Public Health Laboratory	Jade Wang, et al.
EPI_ISL_631543	Robert Kutnick	New York City Public Health Laboratory	Jade Wang, et al.
EPI_ISL_631544, EPI_ISL_631545	New York Presbyterian-Brooklyn Methodist Hospital	New York City Public Health Laboratory	Jade Wang, et al.
EPI_ISL_631546	Maimonides Medical Center	New York City Public Health Laboratory	Jade Wang, et al.
EPI_ISL_631547	New York Presbyterian Queens	New York City Public Health Laboratory	Jade Wang, et al.
EPI_ISL_631548	New York Presbyterian-Brooklyn Methodist Hospital	New York City Public Health Laboratory	Jade Wang, et al.
EPI_ISL_631549	Mount Sinai Hospital	New York City Public Health Laboratory	Jade Wang, et al.
EPI_ISL_631550, EPI_ISL_631551	Jamaica Hospital Medical Center	New York City Public Health Laboratory	Jade Wang, et al.
EPI_ISL_631553	Brookdale University Hospital Medical Center	New York City Public Health Laboratory	Jade Wang, et al.
EPI_ISL_631554	Jamaica Hospital Medical Center	New York City Public Health Laboratory	Jade Wang, et al.
EPI_ISL_631555	Richmond University Medical Center	New York City Public Health Laboratory	Jade Wang, et al.
EPI_ISL_631556	NYC HH Jacobi Medical Center	New York City Public Health Laboratory	Jade Wang, et al.
EPI_ISL_631557	NYC HH Lincoln Medical And Mental Health Center	New York City Public Health Laboratory	Jade Wang, et al.
EPI_ISL_631558, EPI_ISL_631559	NYU Langone Health	New York City Public Health Laboratory	Jade Wang, et al.
EPI_ISL_631560, EPI_ISL_631561	Montefiore Medical Center	New York City Public Health Laboratory	Jade Wang, et al.
EPI_ISL_631562	Brookdale University Hospital Medical Center	New York City Public Health Laboratory	Jade Wang, et al.
EPI_ISL_631563	Flushing Hospital Medical Center	New York City Public Health Laboratory	Jade Wang, et al.
EPI_ISL_631564	NYC HH Elmhurst Hospital Medical Center	New York City Public Health Laboratory	Jade Wang, et al.
EPI_ISL_631565	Brookdale University Hospital Medical Center	New York City Public Health Laboratory	Jade Wang, et al.
EPI_ISL_631566, EPI_ISL_631567	New York Presbyterian-Brooklyn Methodist Hospital	New York City Public Health Laboratory	Jade Wang, et al.
EPI_ISL_631568, EPI_ISL_631569	New York Presbyterian Queens	New York City Public Health Laboratory	Jade Wang, et al.
EPI_ISL_631570	OCME Office Of Chief Medical Examiner	New York City Public Health Laboratory	Jade Wang, et al.
EPI_ISL_631571	Jamaica Hospital Medical Center	New York City Public Health Laboratory	Jade Wang, et al.
EPI_ISL_631572	NYC Department Of Health And Mental Hygiene	New York City Public Health Laboratory	Jade Wang, et al.
EPI_ISL_631573	Brookdale University Hospital Medical Center	New York City Public Health Laboratory	Jade Wang, et al.
EPI_ISL_631574	Jamaica Hospital Medical Center	New York City Public Health Laboratory	Jade Wang, et al.
EPI_ISL_631576	Richmond University Medical Center	New York City Public Health Laboratory	Jade Wang, et al.
EPI_ISL_631577	NYC HH Lincoln Medical And Mental Health Center	New York City Public Health Laboratory	Jade Wang, et al.
EPI_ISL_631578	Jamaica Hospital Medical Center	New York City Public Health Laboratory	Jade Wang, et al.
EPI_ISL_631579	Flushing Hospital Medical Center	New York City Public Health Laboratory	Jade Wang, et al.
EPI_ISL_631580	Bellevue Hospital Center	New York City Public Health Laboratory	Jade Wang, et al.
EPI_ISL_631581	SUNY Downstate Medical Center	New York City Public Health Laboratory	Jade Wang, et al.
EPI_ISL_631582	Jamaica Hospital Medical Center	New York City Public Health Laboratory	Jade Wang, et al.
EPI_ISL_631583	Richmond University Medical Center	New York City Public Health Laboratory	Jade Wang, et al.
EPI_ISL_631584	NYC HH Lincoln Medical And Mental Health Center	New York City Public Health Laboratory	Jade Wang, et al.
EPI_ISL_631585	Jamaica Hospital Medical Center	New York City Public Health Laboratory	Jade Wang, et al.
EPI_ISL_631586	Flushing Hospital Medical Center	New York City Public Health Laboratory	Jade Wang, et al.
EPI_ISL_631587	NYC HH Lincoln Medical And Mental Health Center	New York City Public Health Laboratory	Jade Wang, et al.
EPI_ISL_631588	SUNY Downstate Medical Center	New York City Public Health Laboratory	Jade Wang, et al.
EPI_ISL_631590	Jamaica Hospital Medical Center	New York City Public Health Laboratory	Jade Wang, et al.
EPI_ISL_631591	OCME Office Of Chief Medical Examiner	New York City Public Health Laboratory	Jade Wang, et al.
EPI_ISL_631593	Jamaica Hospital Medical Center	New York City Public Health Laboratory	Jade Wang, et al.

EPI_ISL_631594	OCME Office Of Chief Medical Examiner	New York City Public Health Laboratory	Jade Wang, et al.
EPI_ISL_631595	Jamaica Hospital Medical Center	New York City Public Health Laboratory	Jade Wang, et al.
EPI_ISL_631596	James J. Peters VA Medical Center	New York City Public Health Laboratory	Jade Wang, et al.
EPI_ISL_631597	Jamaica Hospital Medical Center	New York City Public Health Laboratory	Jade Wang, et al.
EPI_ISL_631598	OCME Office Of Chief Medical Examiner	New York City Public Health Laboratory	Jade Wang, et al.
EPI_ISL_631599	Jamaica Hospital Medical Center	New York City Public Health Laboratory	Jade Wang, et al.
EPI_ISL_631600	James J. Peters VA Medical Center	New York City Public Health Laboratory	Jade Wang, et al.
EPI_ISL_631601	Jamaica Hospital Medical Center	New York City Public Health Laboratory	Jade Wang, et al.
EPI_ISL_631602	Flushing Hospital Medical Center	New York City Public Health Laboratory	Jade Wang, et al.
EPI_ISL_631603	NYC HH Lincoln Medical And Mental Health Center	New York City Public Health Laboratory	Jade Wang, et al.
EPI_ISL_631604	Jamaica Hospital Medical Center	New York City Public Health Laboratory	Jade Wang, et al.
EPI_ISL_631605	OCME Office Of Chief Medical Examiner	New York City Public Health Laboratory	Jade Wang, et al.
EPI_ISL_631606	Flushing Hospital Medical Center	New York City Public Health Laboratory	Jade Wang, et al.
EPI_ISL_631607	NYC HH Lincoln Medical And Mental Health Center	New York City Public Health Laboratory	Jade Wang, et al.
EPI_ISL_631608, EPI_ISL_631609, EPI_ISL_631610, EPI_ISL_631612, EPI_ISL_631613, EPI_ISL_631615	Jamaica Hospital Medical Center	New York City Public Health Laboratory	Jade Wang, et al.
EPI_ISL_631616	Flushing Hospital Medical Center	New York City Public Health Laboratory	Jade Wang, et al.
EPI_ISL_631617, EPI_ISL_631618, EPI_ISL_631619	OCME Office Of Chief Medical Examiner	New York City Public Health Laboratory	Jade Wang, et al.
EPI_ISL_631620	Flushing Hospital Medical Center	New York City Public Health Laboratory	Jade Wang, et al.
EPI_ISL_631621	Jamaica Hospital Medical Center	New York City Public Health Laboratory	Jade Wang, et al.
EPI_ISL_631623, EPI_ISL_631624, EPI_ISL_631625, EPI_ISL_631626, EPI_ISL_631627, EPI_ISL_631628, EPI_ISL_631629, EPI_ISL_631630, EPI_ISL_631631, EPI_ISL_631632, EPI_ISL_631633			
see above	Richmond University Medical Center	New York City Public Health Laboratory	Jade Wang, et al.
EPI_ISL_631634, EPI_ISL_631636, EPI_ISL_631637	Jamaica Hospital Medical Center	New York City Public Health Laboratory	Jade Wang, et al.
EPI_ISL_631660, EPI_ISL_631661, EPI_ISL_631669, EPI_ISL_631683	Texas Department of State Health Services	Texas Department of State Health Services	Rashmi Tuladhar, Bonnie Oh, Jenny Zhang, Maliha Rahman, Anita Pokharel, Myong Koag, Chung Wang, Rachel Lee, Grace Kubin, Mayela Pedrueza
EPI_ISL_631685	OCME Office Of Chief Medical Examiner	New York City Public Health Laboratory	Jade Wang, et al.
EPI_ISL_631686	NYC HH Lincoln Medical And Mental Health Center	New York City Public Health Laboratory	Jade Wang, et al.
EPI_ISL_631687	OCME Office Of Chief Medical Examiner	New York City Public Health Laboratory	Jade Wang, et al.
EPI_ISL_631688	Wildlife Conservation Society - Bronx Zoo	New York City Public Health Laboratory	Jade Wang, et al.
EPI_ISL_631689, EPI_ISL_631690	Lincoln Medical Center	New York City Public Health Laboratory	Jade Wang, et al.
EPI_ISL_631693	NYC HH Lincoln Medical And Mental Health Center	New York City Public Health Laboratory	Jade Wang, et al.
EPI_ISL_631694, EPI_ISL_631695	OCME Office Of Chief Medical Examiner	New York City Public Health Laboratory	Jade Wang, et al.
EPI_ISL_631697, EPI_ISL_631698	Jamaica Hospital Medical Center	New York City Public Health Laboratory	Jade Wang, et al.
EPI_ISL_631701	Flushing Hospital Medical Center	New York City Public Health Laboratory	Jade Wang, et al.
EPI_ISL_631702, EPI_ISL_631703, EPI_ISL_631704, EPI_ISL_631705, EPI_ISL_631706, EPI_ISL_631709	Jamaica Hospital Medical Center	New York City Public Health Laboratory	Jade Wang, et al.
EPI_ISL_631710	Flushing Hospital Medical Center	New York City Public Health Laboratory	Jade Wang, et al.
EPI_ISL_631711, EPI_ISL_631712, EPI_ISL_631713, EPI_ISL_631716, EPI_ISL_631717, EPI_ISL_631718, EPI_ISL_631719, EPI_ISL_631720, EPI_ISL_631721, EPI_ISL_631722	Jamaica Hospital Medical Center	New York City Public Health Laboratory	Jade Wang, et al.
EPI_ISL_631724, EPI_ISL_631725	Flushing Hospital Medical Center	New York City Public Health Laboratory	Jade Wang, et al.
EPI_ISL_631726, EPI_ISL_631728, EPI_ISL_631730, EPI_ISL_631731	Jamaica Hospital Medical Center	New York City Public Health Laboratory	Jade Wang, et al.
EPI_ISL_631732	Flushing Hospital Medical Center	New York City Public Health Laboratory	Jade Wang, et al.
EPI_ISL_631733, EPI_ISL_631735, EPI_ISL_631736, EPI_ISL_631737, EPI_ISL_631738, EPI_ISL_631739, EPI_ISL_631740, EPI_ISL_631741, EPI_ISL_631742, EPI_ISL_631743, EPI_ISL_631744, EPI_ISL_631745, EPI_ISL_631746, EPI_ISL_631747			
see above	Jamaica Hospital Medical Center	New York City Public Health Laboratory	Jade Wang, et al.
EPI_ISL_631749, EPI_ISL_631750, EPI_ISL_631751	St Barnabas Hospital	New York City Public Health Laboratory	Jade Wang, et al.
EPI_ISL_631752, EPI_ISL_631753, EPI_ISL_631754	NYC HH Lincoln Medical And Mental Health Center	New York City Public Health Laboratory	Jade Wang, et al.
EPI_ISL_631755	Richmond University Medical Center	New York City Public Health Laboratory	Jade Wang, et al.
EPI_ISL_631756	Flushing Hospital Medical Center	New York City Public Health Laboratory	Jade Wang, et al.
EPI_ISL_631757	Richmond University Medical Center	New York City Public Health Laboratory	Jade Wang, et al.
EPI_ISL_631758, EPI_ISL_631759, EPI_ISL_631760, EPI_ISL_631761, EPI_ISL_631762, EPI_ISL_631763	OCME Office Of Chief Medical Examiner	New York City Public Health Laboratory	Jade Wang, et al.
EPI_ISL_631764	Mount Sinai Hospital	New York City Public Health Laboratory	Jade Wang, et al.
EPI_ISL_631766	Flushing Hospital Medical Center	New York City Public Health Laboratory	Jade Wang, et al.
EPI_ISL_631767, EPI_ISL_631768	Jamaica Hospital Medical Center	New York City Public Health Laboratory	Jade Wang, et al.
EPI_ISL_631769, EPI_ISL_631770, EPI_ISL_631771	Flushing Hospital Medical Center	New York City Public Health Laboratory	Jade Wang, et al.
EPI_ISL_631772	Jamaica Hospital Medical Center	New York City Public Health Laboratory	Jade Wang, et al.
EPI_ISL_631773, EPI_ISL_631774	Flushing Hospital Medical Center	New York City Public Health Laboratory	Jade Wang, et al.
EPI_ISL_631775, EPI_ISL_631776, EPI_ISL_631777	Jamaica Hospital Medical Center	New York City Public Health Laboratory	Jade Wang, et al.

EPI_ISL_631778, EPI_ISL_631779, EPI_ISL_631780, EPI_ISL_631781, EPI_ISL_631782, EPI_ISL_631784	St Barnabas Hospital	New York City Public Health Laboratory	Jade Wang, et al.
EPI_ISL_631785, EPI_ISL_631786	Mount Sinai Hospital	New York City Public Health Laboratory	Jade Wang, et al.
EPI_ISL_631787	Flushing Hospital Medical Center	New York City Public Health Laboratory	Jade Wang, et al.
EPI_ISL_631788, EPI_ISL_631789	Jamaica Hospital Medical Center	New York City Public Health Laboratory	Jade Wang, et al.
EPI_ISL_631792	Richmond University Medical Center	New York City Public Health Laboratory	Jade Wang, et al.
EPI_ISL_631793, EPI_ISL_631794	SUNY Downstate Medical Center	New York City Public Health Laboratory	Jade Wang, et al.
EPI_ISL_631796	Maimonides Medical Center	New York City Public Health Laboratory	Jade Wang, et al.
EPI_ISL_631797	OCME Office Of Chief Medical Examiner	New York City Public Health Laboratory	Jade Wang, et al.
EPI_ISL_631800	Brookdale University Hospital Medical Center	New York City Public Health Laboratory	Jade Wang, et al.
EPI_ISL_631801	Richmond University Medical Center	New York City Public Health Laboratory	Jade Wang, et al.
EPI_ISL_631802	SUNY Downstate Medical Center	New York City Public Health Laboratory	Jade Wang, et al.
EPI_ISL_631803	Brookdale University Hospital Medical Center	New York City Public Health Laboratory	Jade Wang, et al.
EPI_ISL_631804	Bellevue Hospital Center	New York City Public Health Laboratory	Jade Wang, et al.
EPI_ISL_631805, EPI_ISL_631806, EPI_ISL_631807	Brookdale University Hospital Medical Center	New York City Public Health Laboratory	Jade Wang, et al.
EPI_ISL_631808	Wyckoff Heights Medical Center	New York City Public Health Laboratory	Jade Wang, et al.
EPI_ISL_631809, EPI_ISL_631810	Jamaica Hospital Medical Center	New York City Public Health Laboratory	Jade Wang, et al.
EPI_ISL_631811	Wyckoff Heights Medical Center	New York City Public Health Laboratory	Jade Wang, et al.
EPI_ISL_631812, EPI_ISL_631813	Jamaica Hospital Medical Center	New York City Public Health Laboratory	Jade Wang, et al.
EPI_ISL_631815, EPI_ISL_631816, EPI_ISL_631817, EPI_ISL_631818	Richmond University Medical Center	New York City Public Health Laboratory	Jade Wang, et al.
EPI_ISL_631819	Brookdale University Hospital Medical Center	New York City Public Health Laboratory	Jade Wang, et al.
EPI_ISL_631820, EPI_ISL_631822, EPI_ISL_631825	Jamaica Hospital Medical Center	New York City Public Health Laboratory	Jade Wang, et al.
EPI_ISL_631826	Flushing Hospital Medical Center	New York City Public Health Laboratory	Jade Wang, et al.
EPI_ISL_631827	NYC HH Lincoln Medical And Mental Health Center	New York City Public Health Laboratory	Jade Wang, et al.
EPI_ISL_631828	Richmond University Medical Center	New York City Public Health Laboratory	Jade Wang, et al.
EPI_ISL_631829	Brookdale University Hospital Medical Center	New York City Public Health Laboratory	Jade Wang, et al.
EPI_ISL_631830	Mount Sinai Hospital	New York City Public Health Laboratory	Jade Wang, et al.
EPI_ISL_631831	VA NY Harbor Healthcare System	New York City Public Health Laboratory	Jade Wang, et al.
EPI_ISL_631832	Metro Urgicare	New York City Public Health Laboratory	Jade Wang, et al.
EPI_ISL_631833, EPI_ISL_631835, EPI_ISL_631836	Jamaica Hospital Medical Center	New York City Public Health Laboratory	Jade Wang, et al.
EPI_ISL_631837	Richmond University Medical Center	New York City Public Health Laboratory	Jade Wang, et al.
EPI_ISL_631838	Bellevue Hospital Center	New York City Public Health Laboratory	Jade Wang, et al.
EPI_ISL_631840, EPI_ISL_631841, EPI_ISL_631843, EPI_ISL_631845	OCME Office Of Chief Medical Examiner	New York City Public Health Laboratory	Jade Wang, et al.
EPI_ISL_631847	Jamaica Hospital Medical Center	New York City Public Health Laboratory	Jade Wang, et al.
EPI_ISL_631848	Flushing Hospital Medical Center	New York City Public Health Laboratory	Jade Wang, et al.
EPI_ISL_631849	Jamaica Hospital Medical Center	New York City Public Health Laboratory	Jade Wang, et al.
EPI_ISL_631851	Flushing Hospital Medical Center	New York City Public Health Laboratory	Jade Wang, et al.
EPI_ISL_631852, EPI_ISL_631853, EPI_ISL_631854	Jamaica Hospital Medical Center	New York City Public Health Laboratory	Jade Wang, et al.
EPI_ISL_631855	Flushing Hospital Medical Center	New York City Public Health Laboratory	Jade Wang, et al.
EPI_ISL_631860	New York Presbyterian/ Weill Cornell Medical Center	New York City Public Health Laboratory	Jade Wang, et al.
EPI_ISL_631861	New York Presbyterian Queens	New York City Public Health Laboratory	Jade Wang, et al.
EPI_ISL_631862	NYC Department Of Health And Mental Hygiene	New York City Public Health Laboratory	Jade Wang, et al.
EPI_ISL_631863	Maimonides Medical Center	New York City Public Health Laboratory	Jade Wang, et al.
EPI_ISL_631864	Brookdale University Hospital Medical Center	New York City Public Health Laboratory	Jade Wang, et al.
EPI_ISL_631866, EPI_ISL_631867	Jamaica Hospital Medical Center	New York City Public Health Laboratory	Jade Wang, et al.
EPI_ISL_631873, EPI_ISL_631875	Richmond University Medical Center	New York City Public Health Laboratory	Jade Wang, et al.
EPI_ISL_631876, EPI_ISL_631879	Jamaica Hospital Medical Center	New York City Public Health Laboratory	Jade Wang, et al.
EPI_ISL_631880	Mount Sinai West	New York City Public Health Laboratory	Jade Wang, et al.
EPI_ISL_631881	Maimonides Medical Center	New York City Public Health Laboratory	Jade Wang, et al.
EPI_ISL_631882, EPI_ISL_631883	Jamaica Hospital Medical Center	New York City Public Health Laboratory	Jade Wang, et al.
EPI_ISL_631885	Flushing Hospital Medical Center	New York City Public Health Laboratory	Jade Wang, et al.
EPI_ISL_631887	Jamaica Hospital Medical Center	New York City Public Health Laboratory	Jade Wang, et al.
EPI_ISL_631888	St Barnabas Hospital	New York City Public Health Laboratory	Jade Wang, et al.
EPI_ISL_631889, EPI_ISL_631890	Jamaica Hospital Medical Center	New York City Public Health Laboratory	Jade Wang, et al.
EPI_ISL_631891	Flushing Hospital Medical Center	New York City Public Health Laboratory	Jade Wang, et al.
EPI_ISL_631892	Richmond University Medical Center	New York City Public Health Laboratory	Jade Wang, et al.
EPI_ISL_631893, EPI_ISL_631894	Flushing Hospital Medical Center	New York City Public Health Laboratory	Jade Wang, et al.
EPI_ISL_631895, EPI_ISL_631896	Jamaica Hospital Medical Center	New York City Public Health Laboratory	Jade Wang, et al.

EPI_ISL_631897	Richmond University Medical Center	New York City Public Health Laboratory	Jade Wang, et al.
EPI_ISL_631898	OCME Office Of Chief Medical Examiner	New York City Public Health Laboratory	Jade Wang, et al.
EPI_ISL_631902	Jamaica Hospital Medical Center	New York City Public Health Laboratory	Jade Wang, et al.
EPI_ISL_631903	Richmond University Medical Center	New York City Public Health Laboratory	Jade Wang, et al.
EPI_ISL_631904	Jamaica Hospital Medical Center	New York City Public Health Laboratory	Jade Wang, et al.
EPI_ISL_631905	Richmond University Medical Center	New York City Public Health Laboratory	Jade Wang, et al.
EPI_ISL_631906, EPI_ISL_631907	Flushing Hospital Medical Center	New York City Public Health Laboratory	Jade Wang, et al.
EPI_ISL_631909	Jamaica Hospital Medical Center	New York City Public Health Laboratory	Jade Wang, et al.
EPI_ISL_631912	St Barnabas Hospital	New York City Public Health Laboratory	Jade Wang, et al.
EPI_ISL_631914	Mount Sinai Hospital	New York City Public Health Laboratory	Jade Wang, et al.
EPI_ISL_631915	Maimonides Medical Center	New York City Public Health Laboratory	Jade Wang, et al.
EPI_ISL_631917	St Barnabas Hospital	New York City Public Health Laboratory	Jade Wang, et al.
EPI_ISL_631918	Flushing Hospital Medical Center	New York City Public Health Laboratory	Jade Wang, et al.
EPI_ISL_631919, EPI_ISL_631920	Richmond University Medical Center	New York City Public Health Laboratory	Jade Wang, et al.
EPI_ISL_631921, EPI_ISL_631922	Jamaica Hospital Medical Center	New York City Public Health Laboratory	Jade Wang, et al.
EPI_ISL_631923	Flushing Hospital Medical Center	New York City Public Health Laboratory	Jade Wang, et al.
EPI_ISL_631924	St Barnabas Hospital	New York City Public Health Laboratory	Jade Wang, et al.
EPI_ISL_631925, EPI_ISL_631926, EPI_ISL_631927, EPI_ISL_631928	Jamaica Hospital Medical Center	New York City Public Health Laboratory	Jade Wang, et al.
EPI_ISL_631929, EPI_ISL_631931	Flushing Hospital Medical Center	New York City Public Health Laboratory	Jade Wang, et al.
EPI_ISL_631932	OCME Office Of Chief Medical Examiner	New York City Public Health Laboratory	Jade Wang, et al.
EPI_ISL_631933	St Barnabas Hospital	New York City Public Health Laboratory	Jade Wang, et al.
EPI_ISL_631934	OCME Office Of Chief Medical Examiner	New York City Public Health Laboratory	Jade Wang, et al.
EPI_ISL_631935	St Barnabas Hospital	New York City Public Health Laboratory	Jade Wang, et al.
EPI_ISL_631936, EPI_ISL_631937, EPI_ISL_631938	OCME Office Of Chief Medical Examiner	New York City Public Health Laboratory	Jade Wang, et al.
EPI_ISL_631939, EPI_ISL_631940, EPI_ISL_631942, EPI_ISL_631943, EPI_ISL_631944, EPI_ISL_631945, EPI_ISL_631946, EPI_ISL_631949	St Barnabas Hospital	New York City Public Health Laboratory	Jade Wang, et al.
EPI_ISL_631950, EPI_ISL_631951	Jamaica Hospital Medical Center	New York City Public Health Laboratory	Jade Wang, et al.
EPI_ISL_631952	St Barnabas Hospital	New York City Public Health Laboratory	Jade Wang, et al.
EPI_ISL_631953	NYC HH Lincoln Medical And Mental Health Center	New York City Public Health Laboratory	Jade Wang, et al.
EPI_ISL_631954	Richmond University Medical Center	New York City Public Health Laboratory	Jade Wang, et al.
EPI_ISL_631955	Flushing Hospital Medical Center	New York City Public Health Laboratory	Jade Wang, et al.
EPI_ISL_631956	OCME Office Of Chief Medical Examiner	New York City Public Health Laboratory	Jade Wang, et al.
EPI_ISL_631957, EPI_ISL_631958	St Barnabas Hospital	New York City Public Health Laboratory	Jade Wang, et al.
EPI_ISL_631959, EPI_ISL_631960, EPI_ISL_631961	Flushing Hospital Medical Center	New York City Public Health Laboratory	Jade Wang, et al.
EPI_ISL_631962	Richmond University Medical Center	New York City Public Health Laboratory	Jade Wang, et al.
EPI_ISL_631963	Jamaica Hospital Medical Center	New York City Public Health Laboratory	Jade Wang, et al.
EPI_ISL_631964	Flushing Hospital Medical Center	New York City Public Health Laboratory	Jade Wang, et al.
EPI_ISL_631965	Richmond University Medical Center	New York City Public Health Laboratory	Jade Wang, et al.
EPI_ISL_631966	Jamaica Hospital Medical Center	New York City Public Health Laboratory	Jade Wang, et al.
EPI_ISL_631967	Richmond University Medical Center	New York City Public Health Laboratory	Jade Wang, et al.
EPI_ISL_631968	Flushing Hospital Medical Center	New York City Public Health Laboratory	Jade Wang, et al.
EPI_ISL_631969, EPI_ISL_631970	Jamaica Hospital Medical Center	New York City Public Health Laboratory	Jade Wang, et al.
EPI_ISL_631971	St Barnabas Hospital	New York City Public Health Laboratory	Jade Wang, et al.
EPI_ISL_631972	Richmond University Medical Center	New York City Public Health Laboratory	Jade Wang, et al.
EPI_ISL_631973	Jamaica Hospital Medical Center	New York City Public Health Laboratory	Jade Wang, et al.
EPI_ISL_631974	NYC HH Coney Island Hospital	New York City Public Health Laboratory	Jade Wang, et al.
EPI_ISL_631975	Richmond University Medical Center	New York City Public Health Laboratory	Jade Wang, et al.
EPI_ISL_631976	Brookdale University Hospital Medical Center	New York City Public Health Laboratory	Jade Wang, et al.
EPI_ISL_631977	SUNY Downstate Medical Center	New York City Public Health Laboratory	Jade Wang, et al.
EPI_ISL_631978	Brookdale University Hospital Medical Center	New York City Public Health Laboratory	Jade Wang, et al.
EPI_ISL_631979	Jamaica Hospital Medical Center	New York City Public Health Laboratory	Jade Wang, et al.
EPI_ISL_631980	St Barnabas Hospital	New York City Public Health Laboratory	Jade Wang, et al.
EPI_ISL_631981	Brookdale University Hospital Medical Center	New York City Public Health Laboratory	Jade Wang, et al.
EPI_ISL_631982	Montefiore Medical Center	New York City Public Health Laboratory	Jade Wang, et al.
EPI_ISL_631983	Mount Sinai Hospital	New York City Public Health Laboratory	Jade Wang, et al.
EPI_ISL_631984	Brookdale University Hospital Medical Center	New York City Public Health Laboratory	Jade Wang, et al.
EPI_ISL_631985	St Barnabas Hospital	New York City Public Health Laboratory	Jade Wang, et al.

EPI_ISL_631986	Flushing Hospital Medical Center	New York City Public Health Laboratory	Jade Wang, et al.
EPI_ISL_631987, EPI_ISL_631988	St Barnabas Hospital	New York City Public Health Laboratory	Jade Wang, et al.
EPI_ISL_631989	Flushing Hospital Medical Center	New York City Public Health Laboratory	Jade Wang, et al.
EPI_ISL_631990	St Barnabas Hospital	New York City Public Health Laboratory	Jade Wang, et al.
EPI_ISL_631991	Flushing Hospital Medical Center	New York City Public Health Laboratory	Jade Wang, et al.
EPI_ISL_631992	Richmond University Medical Center	New York City Public Health Laboratory	Jade Wang, et al.
EPI_ISL_631993	Maimonides Medical Center	New York City Public Health Laboratory	Jade Wang, et al.
EPI_ISL_631994	Jamaica Hospital Medical Center	New York City Public Health Laboratory	Jade Wang, et al.
EPI_ISL_631995	Wyckoff Heights Medical Center	New York City Public Health Laboratory	Jade Wang, et al.
EPI_ISL_631997	Jamaica Hospital Medical Center	New York City Public Health Laboratory	Jade Wang, et al.
EPI_ISL_631998, EPI_ISL_631999, EPI_ISL_632000, EPI_ISL_632001, EPI_ISL_632002	Richmond University Medical Center	New York City Public Health Laboratory	Jade Wang, et al.
EPI_ISL_632003	Flushing Hospital Medical Center	New York City Public Health Laboratory	Jade Wang, et al.
EPI_ISL_632005	Jamaica Hospital Medical Center	New York City Public Health Laboratory	Jade Wang, et al.
EPI_ISL_632006	OCME Office Of Chief Medical Examiner	New York City Public Health Laboratory	Jade Wang, et al.
EPI_ISL_632007, EPI_ISL_632008, EPI_ISL_632009	Jamaica Hospital Medical Center	New York City Public Health Laboratory	Jade Wang, et al.
EPI_ISL_632010	Flushing Hospital Medical Center	New York City Public Health Laboratory	Jade Wang, et al.
EPI_ISL_632011, EPI_ISL_632012	Jamaica Hospital Medical Center	New York City Public Health Laboratory	Jade Wang, et al.
EPI_ISL_632013, EPI_ISL_632014, EPI_ISL_632015, EPI_ISL_632016	Richmond University Medical Center	New York City Public Health Laboratory	Jade Wang, et al.
EPI_ISL_632017	Jamaica Hospital Medical Center	New York City Public Health Laboratory	Jade Wang, et al.
EPI_ISL_632018, EPI_ISL_632019, EPI_ISL_632020	Flushing Hospital Medical Center	New York City Public Health Laboratory	Jade Wang, et al.
EPI_ISL_632021	Jamaica Hospital Medical Center	New York City Public Health Laboratory	Jade Wang, et al.
EPI_ISL_632022	Flushing Hospital Medical Center	New York City Public Health Laboratory	Jade Wang, et al.
EPI_ISL_632023	Jamaica Hospital Medical Center	New York City Public Health Laboratory	Jade Wang, et al.
EPI_ISL_632024	Flushing Hospital Medical Center	New York City Public Health Laboratory	Jade Wang, et al.
EPI_ISL_632025	Jamaica Hospital Medical Center	New York City Public Health Laboratory	Jade Wang, et al.
EPI_ISL_632026	NYC HH Lincoln Medical And Mental Health Center	New York City Public Health Laboratory	Jade Wang, et al.
EPI_ISL_632027	Flushing Hospital Medical Center	New York City Public Health Laboratory	Jade Wang, et al.
EPI_ISL_632028, EPI_ISL_632029	Jamaica Hospital Medical Center	New York City Public Health Laboratory	Jade Wang, et al.
EPI_ISL_632030, EPI_ISL_632031, EPI_ISL_632032	Richmond University Medical Center	New York City Public Health Laboratory	Jade Wang, et al.
EPI_ISL_632034	Flushing Hospital Medical Center	New York City Public Health Laboratory	Jade Wang, et al.
EPI_ISL_632035	Jamaica Hospital Medical Center	New York City Public Health Laboratory	Jade Wang, et al.
EPI_ISL_632036	Richmond University Medical Center	New York City Public Health Laboratory	Jade Wang, et al.
EPI_ISL_632037	Jamaica Hospital Medical Center	New York City Public Health Laboratory	Jade Wang, et al.
EPI_ISL_632038	New York Presbyterian Lower Manhattan	New York City Public Health Laboratory	Jade Wang, et al.
EPI_ISL_632039	NYC Department Of Health And Mental Hygiene	New York City Public Health Laboratory	Jade Wang, et al.
EPI_ISL_632040	New York Presbyterian Queens	New York City Public Health Laboratory	Jade Wang, et al.
EPI_ISL_632041, EPI_ISL_632042	New York Presbyterian Lower Manhattan	New York City Public Health Laboratory	Jade Wang, et al.
EPI_ISL_632043, EPI_ISL_632044	NYU Langone Health	New York City Public Health Laboratory	Jade Wang, et al.
EPI_ISL_632045	New York Presbyterian Lower Manhattan	New York City Public Health Laboratory	Jade Wang, et al.
EPI_ISL_632046	New York Presbyterian-Brooklyn Methodist Hospital	New York City Public Health Laboratory	Jade Wang, et al.
EPI_ISL_632047	New York Presbyterian Lower Manhattan	New York City Public Health Laboratory	Jade Wang, et al.
EPI_ISL_632048	New York Presbyterian-Brooklyn Methodist Hospital	New York City Public Health Laboratory	Jade Wang, et al.
EPI_ISL_632049	New York Presbyterian Queens	New York City Public Health Laboratory	Jade Wang, et al.
EPI_ISL_632050	Long Island Jewish Medical Center	New York City Public Health Laboratory	Jade Wang, et al.
EPI_ISL_632051	Jamaica Hospital Medical Center	New York City Public Health Laboratory	Jade Wang, et al.
EPI_ISL_632052, EPI_ISL_632053	New York Presbyterian Queens	New York City Public Health Laboratory	Jade Wang, et al.
EPI_ISL_632054	New York Presbyterian-Brooklyn Methodist Hospital	New York City Public Health Laboratory	Jade Wang, et al.
EPI_ISL_632055, EPI_ISL_632056	New York Community Hospital	New York City Public Health Laboratory	Jade Wang, et al.
EPI_ISL_632057	New York-Presbyterian-Columbia University Medical Center	New York City Public Health Laboratory	Jade Wang, et al.
EPI_ISL_632058, EPI_ISL_632059	Proximedical Urgent Care	New York City Public Health Laboratory	Jade Wang, et al.
EPI_ISL_632060	NYC HH Queens Hospital Center	New York City Public Health Laboratory	Jade Wang, et al.
EPI_ISL_632061	Jamaica Hospital Medical Center	New York City Public Health Laboratory	Jade Wang, et al.
EPI_ISL_632062	NYC Department Of Health And Mental Hygiene	New York City Public Health Laboratory	Jade Wang, et al.
EPI_ISL_632063, EPI_ISL_632064	New York Presbyterian Queens	New York City Public Health Laboratory	Jade Wang, et al.
EPI_ISL_632065, EPI_ISL_632066	Jamaica Hospital Medical Center	New York City Public Health Laboratory	Jade Wang, et al.
EPI_ISL_632067	NYU Langone Health	New York City Public Health Laboratory	Jade Wang, et al.
EPI_ISL_632068, EPI_ISL_632069	Bellevue Hospital Center	New York City Public Health Laboratory	Jade Wang, et al.

EPI_ISL_632070, EPI_ISL_632071	New York Presbyterian Queens	New York City Public Health Laboratory	Jade Wang, et al.
EPI_ISL_632072	NYC HH Lincoln Medical And Mental Health Center	New York City Public Health Laboratory	Jade Wang, et al.
EPI_ISL_632073	OCME Office Of Chief Medical Examiner	New York City Public Health Laboratory	Jade Wang, et al.
EPI_ISL_632074	NYC Department Of Health And Mental Hygiene	New York City Public Health Laboratory	Jade Wang, et al.
EPI_ISL_632075	Jamaica Hospital Medical Center	New York City Public Health Laboratory	Jade Wang, et al.
EPI_ISL_632076	NYU Langone Health	New York City Public Health Laboratory	Jade Wang, et al.
EPI_ISL_632077, EPI_ISL_632078	Brookdale University Hospital Medical Center	New York City Public Health Laboratory	Jade Wang, et al.
EPI_ISL_632079	NYU Langone Health	New York City Public Health Laboratory	Jade Wang, et al.
EPI_ISL_632080, EPI_ISL_632091, EPI_ISL_632092	SUNY Downstate Medical Center	New York City Public Health Laboratory	Jade Wang, et al.
EPI_ISL_632093, EPI_ISL_632094, EPI_ISL_632095, EPI_ISL_632096	Jamaica Hospital Medical Center	New York City Public Health Laboratory	Jade Wang, et al.
EPI_ISL_632097	Flushing Hospital Medical Center	New York City Public Health Laboratory	Jade Wang, et al.
EPI_ISL_632098	Bellevue Hospital Center	New York City Public Health Laboratory	Jade Wang, et al.
EPI_ISL_632099	VA NY Harbor Healthcare System	New York City Public Health Laboratory	Jade Wang, et al.
EPI_ISL_632100	Brookdale University Hospital Medical Center	New York City Public Health Laboratory	Jade Wang, et al.
EPI_ISL_632101, EPI_ISL_632102	Mount Sinai Queens	New York City Public Health Laboratory	Jade Wang, et al.
EPI_ISL_632103, EPI_ISL_632104, EPI_ISL_632105	Flushing Hospital Medical Center	New York City Public Health Laboratory	Jade Wang, et al.
EPI_ISL_632106, EPI_ISL_632107, EPI_ISL_632108, EPI_ISL_632109, EPI_ISL_632110	Jamaica Hospital Medical Center	New York City Public Health Laboratory	Jade Wang, et al.
EPI_ISL_632111	Montefiore Medical Center	New York City Public Health Laboratory	Jade Wang, et al.
EPI_ISL_632112, EPI_ISL_632113	Richmond University Medical Center	New York City Public Health Laboratory	Jade Wang, et al.
EPI_ISL_632114	Brookdale University Hospital Medical Center	New York City Public Health Laboratory	Jade Wang, et al.
EPI_ISL_632115	Richmond University Medical Center	New York City Public Health Laboratory	Jade Wang, et al.
EPI_ISL_632116	NYC HH Lincoln Medical And Mental Health Center	New York City Public Health Laboratory	Jade Wang, et al.
EPI_ISL_632117, EPI_ISL_632118	Richmond University Medical Center	New York City Public Health Laboratory	Jade Wang, et al.
EPI_ISL_632119, EPI_ISL_632120	Flushing Hospital Medical Center	New York City Public Health Laboratory	Jade Wang, et al.
EPI_ISL_632121	Bellevue Hospital Center	New York City Public Health Laboratory	Jade Wang, et al.
EPI_ISL_632122	Flushing Hospital Medical Center	New York City Public Health Laboratory	Jade Wang, et al.
EPI_ISL_632123	Brookdale University Hospital Medical Center	New York City Public Health Laboratory	Jade Wang, et al.
EPI_ISL_632124, EPI_ISL_632125, EPI_ISL_632126, EPI_ISL_632127	Flushing Hospital Medical Center	New York City Public Health Laboratory	Jade Wang, et al.
EPI_ISL_632128	Jamaica Hospital Medical Center	New York City Public Health Laboratory	Jade Wang, et al.
EPI_ISL_632129	Wyckoff Heights Medical Center	New York City Public Health Laboratory	Jade Wang, et al.
EPI_ISL_632130	Jamaica Hospital Medical Center	New York City Public Health Laboratory	Jade Wang, et al.
EPI_ISL_632131	NYC HH Lincoln Medical And Mental Health Center	New York City Public Health Laboratory	Jade Wang, et al.
EPI_ISL_632132, EPI_ISL_632133, EPI_ISL_632134	Flushing Hospital Medical Center	New York City Public Health Laboratory	Jade Wang, et al.
EPI_ISL_632135	SUNY Downstate Medical Center	New York City Public Health Laboratory	Jade Wang, et al.
EPI_ISL_632136	Bellevue Hospital Center	New York City Public Health Laboratory	Jade Wang, et al.
EPI_ISL_632137, EPI_ISL_632138, EPI_ISL_632139, EPI_ISL_632140	Flushing Hospital Medical Center	New York City Public Health Laboratory	Jade Wang, et al.
EPI_ISL_632141	Wyckoff Heights Medical Center	New York City Public Health Laboratory	Jade Wang, et al.
EPI_ISL_632142	Brookdale University Hospital Medical Center	New York City Public Health Laboratory	Jade Wang, et al.
EPI_ISL_632143	Jamaica Hospital Medical Center	New York City Public Health Laboratory	Jade Wang, et al.
EPI_ISL_632144	Flushing Hospital Medical Center	New York City Public Health Laboratory	Jade Wang, et al.
EPI_ISL_632145	Jamaica Hospital Medical Center	New York City Public Health Laboratory	Jade Wang, et al.
EPI_ISL_632146, EPI_ISL_632147	NYC HH Lincoln Medical And Mental Health Center	New York City Public Health Laboratory	Jade Wang, et al.
EPI_ISL_632148	SUNY Downstate Medical Center	New York City Public Health Laboratory	Jade Wang, et al.
EPI_ISL_632149, EPI_ISL_632150, EPI_ISL_632151, EPI_ISL_632152	Jamaica Hospital Medical Center	New York City Public Health Laboratory	Jade Wang, et al.
EPI_ISL_632153	SUNY Downstate Medical Center	New York City Public Health Laboratory	Jade Wang, et al.
EPI_ISL_632154	Richmond University Medical Center	New York City Public Health Laboratory	Jade Wang, et al.
EPI_ISL_632155, EPI_ISL_632156	Brookdale University Hospital Medical Center	New York City Public Health Laboratory	Jade Wang, et al.
EPI_ISL_632157	Wyckoff Heights Medical Center	New York City Public Health Laboratory	Jade Wang, et al.
EPI_ISL_632158	SUNY Downstate Medical Center	New York City Public Health Laboratory	Jade Wang, et al.
EPI_ISL_632159	Wyckoff Heights Medical Center	New York City Public Health Laboratory	Jade Wang, et al.
EPI_ISL_632160, EPI_ISL_632161	SUNY Downstate Medical Center	New York City Public Health Laboratory	Jade Wang, et al.
EPI_ISL_632162	Jamaica Hospital Medical Center	New York City Public Health Laboratory	Jade Wang, et al.
EPI_ISL_632163, EPI_ISL_632164, EPI_ISL_632165	Richmond University Medical Center	New York City Public Health Laboratory	Jade Wang, et al.
EPI_ISL_632166, EPI_ISL_632167, EPI_ISL_632168, EPI_ISL_632169, EPI_ISL_632170, EPI_ISL_632171, EPI_ISL_632172	Jamaica Hospital Medical Center	New York City Public Health Laboratory	Jade Wang, et al.

EPI_ISL_632173	Richmond University Medical Center	New York City Public Health Laboratory	Jade Wang, et al.
EPI_ISL_632174	Flushing Hospital Medical Center	New York City Public Health Laboratory	Jade Wang, et al.
EPI_ISL_632175, EPI_ISL_632176	OCME Office Of Chief Medical Examiner	New York City Public Health Laboratory	Jade Wang, et al.
EPI_ISL_632177	NYC HH Lincoln Medical And Mental Health Center	New York City Public Health Laboratory	Jade Wang, et al.
EPI_ISL_632178	OCME Office Of Chief Medical Examiner	New York City Public Health Laboratory	Jade Wang, et al.
EPI_ISL_632179	James J. Peters VA Medical Center	New York City Public Health Laboratory	Jade Wang, et al.
EPI_ISL_632180	Jamaica Hospital Medical Center	New York City Public Health Laboratory	Jade Wang, et al.
EPI_ISL_632181	Flushing Hospital Medical Center	New York City Public Health Laboratory	Jade Wang, et al.
EPI_ISL_632182	Jamaica Hospital Medical Center	New York City Public Health Laboratory	Jade Wang, et al.
EPI_ISL_632183	OCME Office Of Chief Medical Examiner	New York City Public Health Laboratory	Jade Wang, et al.
EPI_ISL_632184	Jamaica Hospital Medical Center	New York City Public Health Laboratory	Jade Wang, et al.
EPI_ISL_632185	Flushing Hospital Medical Center	New York City Public Health Laboratory	Jade Wang, et al.
EPI_ISL_632186, EPI_ISL_632187	NYC HH Lincoln Medical And Mental Health Center	New York City Public Health Laboratory	Jade Wang, et al.
EPI_ISL_632188, EPI_ISL_632189	Jamaica Hospital Medical Center	New York City Public Health Laboratory	Jade Wang, et al.
EPI_ISL_632190	Flushing Hospital Medical Center	New York City Public Health Laboratory	Jade Wang, et al.
EPI_ISL_632191	Jamaica Hospital Medical Center	New York City Public Health Laboratory	Jade Wang, et al.
EPI_ISL_632192, EPI_ISL_632193	NYC HH Lincoln Medical And Mental Health Center	New York City Public Health Laboratory	Jade Wang, et al.
EPI_ISL_632194	Flushing Hospital Medical Center	New York City Public Health Laboratory	Jade Wang, et al.
EPI_ISL_632195	Jamaica Hospital Medical Center	New York City Public Health Laboratory	Jade Wang, et al.
EPI_ISL_632196, EPI_ISL_632197	Flushing Hospital Medical Center	New York City Public Health Laboratory	Jade Wang, et al.
EPI_ISL_632198	Jamaica Hospital Medical Center	New York City Public Health Laboratory	Jade Wang, et al.
EPI_ISL_632199	Richmond University Medical Center	New York City Public Health Laboratory	Jade Wang, et al.
EPI_ISL_632200	James J. Peters VA Medical Center	New York City Public Health Laboratory	Jade Wang, et al.
EPI_ISL_632201	Richmond University Medical Center	New York City Public Health Laboratory	Jade Wang, et al.
EPI_ISL_632202	OCME Office Of Chief Medical Examiner	New York City Public Health Laboratory	Jade Wang, et al.
EPI_ISL_632203	Richmond University Medical Center	New York City Public Health Laboratory	Jade Wang, et al.
EPI_ISL_632204	Jamaica Hospital Medical Center	New York City Public Health Laboratory	Jade Wang, et al.
EPI_ISL_632205, EPI_ISL_632206, EPI_ISL_632207, EPI_ISL_632208	Richmond University Medical Center	New York City Public Health Laboratory	Jade Wang, et al.
EPI_ISL_632209, EPI_ISL_632210	Jamaica Hospital Medical Center	New York City Public Health Laboratory	Jade Wang, et al.
EPI_ISL_632211, EPI_ISL_632212, EPI_ISL_632213	Flushing Hospital Medical Center	New York City Public Health Laboratory	Jade Wang, et al.
EPI_ISL_632214	Jamaica Hospital Medical Center	New York City Public Health Laboratory	Jade Wang, et al.
EPI_ISL_632215, EPI_ISL_632216, EPI_ISL_632217, EPI_ISL_632218	Richmond University Medical Center	New York City Public Health Laboratory	Jade Wang, et al.
EPI_ISL_632219	Jamaica Hospital Medical Center	New York City Public Health Laboratory	Jade Wang, et al.
EPI_ISL_632220	Flushing Hospital Medical Center	New York City Public Health Laboratory	Jade Wang, et al.
EPI_ISL_632221	Jamaica Hospital Medical Center	New York City Public Health Laboratory	Jade Wang, et al.
EPI_ISL_632222, EPI_ISL_632223, EPI_ISL_632224, EPI_ISL_632225, EPI_ISL_632226	Flushing Hospital Medical Center	New York City Public Health Laboratory	Jade Wang, et al.
EPI_ISL_632227, EPI_ISL_632228, EPI_ISL_632229	Jamaica Hospital Medical Center	New York City Public Health Laboratory	Jade Wang, et al.
EPI_ISL_632230, EPI_ISL_632231, EPI_ISL_632232, EPI_ISL_632233, EPI_ISL_632234	Richmond University Medical Center	New York City Public Health Laboratory	Jade Wang, et al.
EPI_ISL_632235	NYC HH Lincoln Medical And Mental Health Center	New York City Public Health Laboratory	Jade Wang, et al.
EPI_ISL_632237	Flushing Hospital Medical Center	New York City Public Health Laboratory	Jade Wang, et al.
EPI_ISL_632238	OCME Office Of Chief Medical Examiner	New York City Public Health Laboratory	Jade Wang, et al.
EPI_ISL_632239, EPI_ISL_632240, EPI_ISL_632241	Jamaica Hospital Medical Center	New York City Public Health Laboratory	Jade Wang, et al.
EPI_ISL_632242, EPI_ISL_632247, EPI_ISL_632248, EPI_ISL_632249	NHSGGC West of Scotland Specialist Virology Centre	MRC-University of Glasgow Centre for Virus Research	Ana da Silva Filipe, Natasha Johnson, Kathy Smollett, Daniel Mair, Stephen Carmichael, Lily Tong, Jenna Nichols, Kyriaki Nomikou; Richard Orton, Joseph Hughes, Sreenu Vattipally, David L Robertson; Alasdair MacLean, Rory Gunson; Kathy Li, Natasha Jesudason, Rajiv Shah, James Shepherd, Antonia Ho, Emma Thomson; Alex Alderton, Roberto Amato, Sonia Goncalves, Ewan Harrison, David K. Jackson, Ian Johnston, Dominic Kwiatkowski, Cordelia Langford, John Sillitoe on behalf of the Wellcome Sanger Institute COVID-19 Surveillance Team (http://www.sanger.ac.uk/covid-team)
EPI_ISL_632250, EPI_ISL_632251, EPI_ISL_632252, EPI_ISL_632253, EPI_ISL_632254, EPI_ISL_632255	Communicable Disease Laboratory, Public Health Directorate	Communicable Disease Laboratory, Public Health Directorate	AlWasti,H., Altaif,Z., AlHujairi,Z., AlAbbas,Z.
EPI_ISL_632256, EPI_ISL_632257, EPI_ISL_632258	Communicable Disease Laboratory, Public Health Directorate	Communicable Disease Laboratory, Public Health Directorate	AlHujairi,Z., Altaif,Z., AlWasti,H., AlAbbas,Z.
EPI_ISL_632259, EPI_ISL_632260	Communicable Disease Laboratory, Public Health Directorate	Communicable Disease Laboratory, Public Health Directorate	AlTaif,Z., AlWasti,H., AlHujairi,Z., AlAbbas,Z.
EPI_ISL_632261, EPI_ISL_632262, EPI_ISL_632263, EPI_ISL_632264, EPI_ISL_632265, EPI_ISL_632266, EPI_ISL_632267, EPI_ISL_632268, EPI_ISL_632269, EPI_ISL_632270, EPI_ISL_632271, EPI_ISL_632272, EPI_ISL_632273, EPI_ISL_632274, EPI_ISL_632275, EPI_ISL_632276, EPI_ISL_632277, EPI_ISL_632278, EPI_ISL_632279, EPI_ISL_632280, EPI_ISL_632281, EPI_ISL_632282			
see above	Communicable Disease Laboratory, Public Health Directorate	Communicable Disease Laboratory, Public Health Directorate	AlWasti,H., AlTaif,Z., AlHujairi,Z., AlAbbas,Z.
EPI_ISL_632287	Environmental and Global Health, University of Florida	Environmental and Global Health, University of Florida	Alam,M.M., Lauzardo,M., Elbadry,M.A., Stephenson,C.J., Gibson,J.C., Morris,J.G. and Lednicky,J.A.
EPI_ISL_632288, EPI_ISL_632289, EPI_ISL_632290, EPI_ISL_632291, EPI_ISL_632292, EPI_ISL_632293, EPI_ISL_632294, EPI_ISL_632295, EPI_ISL_632296, EPI_ISL_632297, EPI_ISL_632298, EPI_ISL_632299, EPI_ISL_632300, EPI_ISL_632301, EPI_ISL_632302, EPI_ISL_632303, EPI_ISL_632304, EPI_ISL_632305, EPI_ISL_632306, EPI_ISL_632307, EPI_ISL_632308, EPI_ISL_632309			

see above	Brigham and Women's Hospital	Jonathan Li laboratory	Choudhary MC, Esmaeilzadeh E, Etemad B, Mohammadi A, Regan J, Li JZ
EPI_ISL_632310	1-Laboratory of Microbiology, National Reference Lab, Charles Nicolle Hospital; 2-University of Tunis ElManar, Faculty of Medicine of Tunis, LR99ES09, Tunis, Tunisia	1-Clinical and Experimental Pharmacology Lab, LR16SP02, National Center of Pharmacovigilance, University of Tunis El Manar, Tunis, Tunisia. 2-Neurodegenerative diseases and psychiatric troubles, LR18SP03, Razi Hospital, University of Tunis El Manar, Tunis, Tunisia. 3- Ministry of Health, National Observatory of New and Emerging Diseases, 1006, Tunis, Tunisia	Ilhem Boutiba-Ben Boubaker, Sameh Trabelsi, Nissaf Ben Alaya, Maher Kharrat, Alia Ben Kahla, Jalila Ben Khelil, Salma Abid, Sana Ferjani, Mouna Ben Sassi, Mouna Safer, Imen Mkada, Imen Kacem, Gaies Emna, Soumaya Rammeh, Riadh Daghfous, Riadh Gouider.
EPI_ISL_632311	MVZ DIAMEDIS Diagnostische Medizin Sennestadt GmbH	Bielefeld University	David Brandt, Tobias Busche, Markus Haak, Jörn Kalinowski, Levin-Joe Klages, Christiane Scherer, Alexander Sczyrba, Marina Simunovic, Svenja Vinke
EPI_ISL_632312	NU-sjukvården	Clinical microbiology, Sahlgrenska University Hospital	Johan Ringlander, Josefin Olausson, Hedvig Engström Jakobsson, Magnus Lindh
EPI_ISL_632313	NU-sjukvården	Clinical Microbiology, Sahlgrenska University Hospital,	Johan Ringlander, Josefin Olausson, Hedvig Engström Jakobsson, Magnus Lindh
EPI_ISL_632389, EPI_ISL_632390, EPI_ISL_632391, EPI_ISL_632431, EPI_ISL_632432, EPI_ISL_632433, EPI_ISL_632440, EPI_ISL_632441, EPI_ISL_632442, EPI_ISL_632443, EPI_ISL_632444, EPI_ISL_632445, EPI_ISL_632448, EPI_ISL_632460, EPI_ISL_632461, EPI_ISL_632479, EPI_ISL_632480, EPI_ISL_632481, EPI_ISL_632482, EPI_ISL_632494, EPI_ISL_632495, EPI_ISL_632496, EPI_ISL_632497, EPI_ISL_632498, EPI_ISL_632499, EPI_ISL_632500, EPI_ISL_632501, EPI_ISL_632502, EPI_ISL_632503, EPI_ISL_632504, EPI_ISL_632505, EPI_ISL_632506, EPI_ISL_632507, EPI_ISL_632508, EPI_ISL_632509, EPI_ISL_632510, EPI_ISL_632511, EPI_ISL_632512, EPI_ISL_632513, EPI_ISL_632514, EPI_ISL_632515, EPI_ISL_632516, EPI_ISL_632517, EPI_ISL_632518, EPI_ISL_632519, EPI_ISL_632520, EPI_ISL_632521, EPI_ISL_632522, EPI_ISL_632523, EPI_ISL_632524, EPI_ISL_632525, EPI_ISL_632526, EPI_ISL_632527, EPI_ISL_632528, EPI_ISL_632529, EPI_ISL_632530, EPI_ISL_632531, EPI_ISL_632532, EPI_ISL_632533, EPI_ISL_632534, EPI_ISL_632535, EPI_ISL_632536, EPI_ISL_632537, EPI_ISL_632538, EPI_ISL_632539, EPI_ISL_632540, EPI_ISL_632541, EPI_ISL_632542, EPI_ISL_632543, EPI_ISL_632544, EPI_ISL_632545, EPI_ISL_632546, EPI_ISL_632547, EPI_ISL_632548, EPI_ISL_632549, EPI_ISL_632550, EPI_ISL_632551, EPI_ISL_632552, EPI_ISL_632553, EPI_ISL_632554, EPI_ISL_632555, EPI_ISL_632556, EPI_ISL_632557, EPI_ISL_632558, EPI_ISL_632559, EPI_ISL_632560, EPI_ISL_632561, EPI_ISL_632562, EPI_ISL_632563, EPI_ISL_632564, EPI_ISL_632565, EPI_ISL_632566, EPI_ISL_632567, EPI_ISL_632568, EPI_ISL_632569, EPI_ISL_632570, EPI_ISL_632571, EPI_ISL_632572, EPI_ISL_632573, EPI_ISL_632574, EPI_ISL_632575, EPI_ISL_632576, EPI_ISL_632577, EPI_ISL_632578, EPI_ISL_632579, EPI_ISL_632580, EPI_ISL_632581, EPI_ISL_632582, EPI_ISL_632583, EPI_ISL_632584, EPI_ISL_632585, EPI_ISL_632586, EPI_ISL_632587, EPI_ISL_632588, EPI_ISL_632589, EPI_ISL_632590, EPI_ISL_632591, EPI_ISL_632592, EPI_ISL_632593, EPI_ISL_632594, EPI_ISL_632595, EPI_ISL_632596, EPI_ISL_632597, EPI_ISL_632598, EPI_ISL_632599, EPI_ISL_632600, EPI_ISL_632601, EPI_ISL_632602, EPI_ISL_632603, EPI_ISL_632604, EPI_ISL_632605, EPI_ISL_632606, EPI_ISL_632607, EPI_ISL_632608, EPI_ISL_632609, EPI_ISL_632610, EPI_ISL_632611, EPI_ISL_632612, EPI_ISL_632613, EPI_ISL_632614, EPI_ISL_632615, EPI_ISL_632616, EPI_ISL_632617, EPI_ISL_632618, EPI_ISL_632619, EPI_ISL_632620, EPI_ISL_632621, EPI_ISL_632622, EPI_ISL_632623, EPI_ISL_632624, EPI_ISL_632625, EPI_ISL_632626, EPI_ISL_632627, EPI_ISL_632628, EPI_ISL_632629, EPI_ISL_632630, EPI_ISL_632631, EPI_ISL_632632, EPI_ISL_632633, EPI_ISL_632634, EPI_ISL_632635, EPI_ISL_632636, EPI_ISL_632637, EPI_ISL_632638, EPI_ISL_632639, EPI_ISL_632640, EPI_ISL_632641, EPI_ISL_632642, EPI_ISL_632643, EPI_ISL_632644, EPI_ISL_632645, EPI_ISL_632646, EPI_ISL_632647, EPI_ISL_632648, EPI_ISL_632649, EPI_ISL_632650, EPI_ISL_632651, EPI_ISL_632652, EPI_ISL_632653, EPI_ISL_632654, EPI_ISL_632655, EPI_ISL_632656, EPI_ISL_632657, EPI_ISL_632658, EPI_ISL_632659, EPI_ISL_632660, EPI_ISL_632661, EPI_ISL_632662, EPI_ISL_632663, EPI_ISL_632664, EPI_ISL_632665, EPI_ISL_632666, EPI_ISL_632667, EPI_ISL_632668, EPI_ISL_632669, EPI_ISL_632670, EPI_ISL_632671, EPI_ISL_632672, EPI_ISL_632673, EPI_ISL_632674, EPI_ISL_632675, EPI_ISL_632676, EPI_ISL_632677, EPI_ISL_632678, EPI_ISL_632679, EPI_ISL_632680, EPI_ISL_632681, EPI_ISL_632682, EPI_ISL_632683, EPI_ISL_632684, EPI_ISL_632685, EPI_ISL_632686, EPI_ISL_632687, EPI_ISL_632688, EPI_ISL_632689, EPI_ISL_632690, EPI_ISL_632691, EPI_ISL_632692, EPI_ISL_632693, EPI_ISL_632694, EPI_ISL_632695, EPI_ISL_632696, EPI_ISL_632697, EPI_ISL_632698, EPI_ISL_632699, EPI_ISL_632700, EPI_ISL_632701, EPI_ISL_632702, EPI_ISL_632703, EPI_ISL_632704, EPI_ISL_632705, EPI_ISL_632706, EPI_ISL_632707, EPI_ISL_632708, EPI_ISL_632709, EPI_ISL_632710, EPI_ISL_632711, EPI_ISL_632712, EPI_ISL_632713, EPI_ISL_632714, EPI_ISL_632715, EPI_ISL_632716, EPI_ISL_632717, EPI_ISL_632718, EPI_ISL_632719, EPI_ISL_632720, EPI_ISL_632721, EPI_ISL_632722, EPI_ISL_632723, EPI_ISL_632724, EPI_ISL_632725, EPI_ISL_632726, EPI_ISL_632727, EPI_ISL_632728, EPI_ISL_632729, EPI_ISL_632730, EPI_ISL_632731, EPI_ISL_632732, EPI_ISL_632733, EPI_ISL_632734, EPI_ISL_632735, EPI_ISL_632736, EPI_ISL_632737, EPI_ISL_632738, EPI_ISL_632739, EPI_ISL_632740, EPI_ISL_632741, EPI_ISL_632742, EPI_ISL_632743, EPI_ISL_632744, EPI_ISL_632745, EPI_ISL_632746, EPI_ISL_632747, EPI_ISL_632748, EPI_ISL_632749, EPI_ISL_632750, EPI_ISL_632751, EPI_ISL_632752, EPI_ISL_632753, EPI_ISL_632754, EPI_ISL_632755, EPI_ISL_632756, EPI_ISL_632757, EPI_ISL_632758, EPI_ISL_632759, EPI_ISL_632760, EPI_ISL_632761, EPI_ISL_632762, EPI_ISL_632763, EPI_ISL_632764, EPI_ISL_632765, EPI_ISL_632766, EPI_ISL_632767, EPI_ISL_632768, EPI_ISL_632769, EPI_ISL_632770, EPI_ISL_632771, EPI_ISL_632772, EPI_ISL_632773, EPI_ISL_632774, EPI_ISL_632775, EPI_ISL_632776, EPI_ISL_632777, EPI_ISL_632778, EPI_ISL_632779, EPI_ISL_632780, EPI_ISL_632781, EPI_ISL_632782, EPI_ISL_632783, EPI_ISL_632784, EPI_ISL_632785, EPI_ISL_632786, EPI_ISL_632787, EPI_ISL_632788, EPI_ISL_632789, EPI_ISL_632790, EPI_ISL_632791, EPI_ISL_632792, EPI_ISL_632793, EPI_ISL_632794, EPI_ISL_632795			
see above	Dutch COVID-19 response team	Erasmus Medical Center	Bas Oude Munnink, David Nieuwenhuijse, Reina Sikkema, Claudia Schapendonk, Irina Chestakova, Anne van der Linden, Theo Bestebroer, Stefan van Nieuwkoop, Mark Pronk, Pascal Lexmond, Corien Swaan, Manon Haverkate, Madelief Mollers, Mart Stein, Sandra Kengne Kamga Mobou, Jeroen van Kampen, Jolanda Voermans, Aura Timen, Corine GeurtsvanKessel, Annemiek van der Eijk, Richard Molenkamp, Marion Koopmans, on behalf of the Dutch national COVID-19 response team.
EPI_ISL_632796	Respiratory Virus Unit, Microbiology Services Colindale, Public Health England	Respiratory Virus Unit, Microbiology Services Colindale, Public Health England	PHE Covid Sequencing Team
EPI_ISL_632797, EPI_ISL_632798, EPI_ISL_632799, EPI_ISL_632800, EPI_ISL_632801, EPI_ISL_632802, EPI_ISL_632803, EPI_ISL_632804, EPI_ISL_632805, EPI_ISL_632806, EPI_ISL_632807, EPI_ISL_632808, EPI_ISL_632809, EPI_ISL_632810, EPI_ISL_632811, EPI_ISL_632812, EPI_ISL_632813, EPI_ISL_632815, EPI_ISL_632816, EPI_ISL_632817, EPI_ISL_632818, EPI_ISL_632819, EPI_ISL_632820, EPI_ISL_632821, EPI_ISL_632822			
see above	Microbiology, Department of Pathology, St. Bernard's Hospital, Gibraltar Health Authority	Respiratory Virus Unit, Microbiology Services Colindale, Public Health England	PHE Covid Sequencing Team, Dr Nicholas Cortes (Gibraltar), Charlotte Gillborn-Jones (Gibraltar)
EPI_ISL_632823, EPI_ISL_632824, EPI_ISL_632825, EPI_ISL_632826, EPI_ISL_632827, EPI_ISL_632828, EPI_ISL_632831, EPI_ISL_632832, EPI_ISL_632834, EPI_ISL_632836, EPI_ISL_632837, EPI_ISL_632839, EPI_ISL_632841, EPI_ISL_632843, EPI_ISL_632844, EPI_ISL_632845			
see above	New Mexico Department of Health Scientific Laboratory	New Mexico Department of Health Scientific Laboratory	Ellie Johnson, Anastacia Griego-Fisher, D'Eldra Malone
EPI_ISL_632847, EPI_ISL_632848, EPI_ISL_632849, EPI_ISL_632850, EPI_ISL_632851, EPI_ISL_632852, EPI_ISL_632853, EPI_ISL_632856, EPI_ISL_632857, EPI_ISL_632858, EPI_ISL_632859, EPI_ISL_632861, EPI_ISL_632863, EPI_ISL_632864, EPI_ISL_632865, EPI_ISL_632868, EPI_ISL_632870, EPI_ISL_632871, EPI_ISL_632872, EPI_ISL_632873, EPI_ISL_632876, EPI_ISL_632878, EPI_ISL_632879, EPI_ISL_632880, EPI_ISL_632881, EPI_ISL_632882, EPI_ISL_632883, EPI_ISL_632885, EPI_ISL_632886			
see above	Idaho Bureau of Laboratories	Center for Global Health, University of New Mexico Health Sciences Center	Daryl Domman, Kurt Schwalm, Matthew Burns, Robert Voermans, Christopher Ball, Darrell Dinwiddie
EPI_ISL_632891, EPI_ISL_632892, EPI_ISL_632893, EPI_ISL_632895, EPI_ISL_632896, EPI_ISL_632897, EPI_ISL_632898	University of Wisconsin-Madison AIDS Vaccine Research Laboratories	University of Wisconsin-Madison AIDS Vaccine Research Laboratories	Gage Moreno, Katarina Braun, et al. AIDS Vaccine Research Laboratories
EPI_ISL_632899, EPI_ISL_632900, EPI_ISL_632901, EPI_ISL_632902, EPI_ISL_632903	Communicable Disease Laboratory, Public Health Directorate	Communicable Disease Laboratory, Public Health Directorate	AlAbbas,Z., Altaif,Z., AlWasti,H., Alhujairi,Z.
EPI_ISL_632904	Communicable Disease Laboratory, Public Health Directorate	Communicable Disease Laboratory, Public Health Directorate	AlTaif,Z., AlHujairi,Z., AlWasti,H., AlAbbas,Z.
EPI_ISL_632905	Communicable Disease Laboratory, Public Health Directorate	Communicable Disease Laboratory, Public Health Directorate	AlHujairi,Z., Altaif,Z., AlWasti,H., AlAbbas,Z.
EPI_ISL_632906, EPI_ISL_632907	Communicable Disease Laboratory, Public Health Directorate	Communicable Disease Laboratory, Public Health Directorate	AlAbbas,Z., Altaif,Z., AlWasti,H., Alhujairi,Z.
EPI_ISL_632908	Genomic Sciences, Rehman Medical Institute	Genomic Sciences, Rehman Medical Institute	Ali,J., Afridi,U.K., Haider,S.A., Sabiha,B., Jan,H. and Jehanzeb,V.
EPI_ISL_632909, EPI_ISL_632910, EPI_ISL_632911, EPI_ISL_632912, EPI_ISL_632913, EPI_ISL_632914, EPI_ISL_632915, EPI_ISL_632916, EPI_ISL_632917, EPI_ISL_632918, EPI_ISL_632919, EPI_ISL_632920, EPI_ISL_632921, EPI_ISL_632922, EPI_ISL_632923, EPI_ISL_632924, EPI_ISL_632925, EPI_ISL_632926, EPI_ISL_632927, EPI_ISL_632928, EPI_ISL_632929, EPI_ISL_632930, EPI_ISL_632931, EPI_ISL_632932, EPI_ISL_632933			
see above	Cadham Provincial laboratory	Cadham Provincial laboratory	Anna Majer, Shari Tyson, Grace Seo, Philip Mabon, Elsie Grudeski, Rhiannon Huzarewich, Russell Mandes, Anneliese Landgraff, Jennifer Tanner, Natalie Knox, Morag Graham, Gary Van Domselaar, Paul Van Caeseele, Jared Bullard, David Alexander, Kerry Dust, Nathalie Bastien, Yan Li, Timothy Booth, Darian Hole, Madison Chapel, CanCOGeN's metadata curation team, Public Health Agency of Canada CanCOGeN team
EPI_ISL_632934	Department of Acute Infectious Diseases Control and Prevention,Yunnan Provincial Center for Disease Control and Prevention	Department of Acute Infectious Diseases Control and Prevention,Yunnan Provincial Center for Disease Control and Prevention	Meiling Zhang,Jienan Zhou,Senquan Jia,Xiaonan Zhao,Xiaoqing Fu
EPI_ISL_632936	Puskesmas Mlati 1 Sleman	Genetics Working Group (Pokja Genetik) Faculty of Medicine, Public Health and Nursing Universitas Gadjah Mada (FK-KMK UGM); Disease Investigation Center Wates Ministry of Agriculture Indonesia; Department of Microbiology FK-KMK UGM; Laboratorium Diagnostik Yayasan Tahija World Mosquito Program (WMP) Yogyakarta Center for Tropical Medicine FK-KMK UGM;	Gunadi, Hendra Wibawa, Marcellus, Mohamad S. Hakim, Edwin W. Daniwijaya, Ludhang P. Rizki, Endah Supriyati, Eggi Arguni, Titik Nuryastuti, Tri Wibawa, Dwi AA Nugrahaningsih, Afiahayati, Siswanto, Kristy Iskandar, Nungki Anggorowati, Ika Trisnawati, Sumardi, Eko Budiono, Bambang Sigit Riyanto, Heni Retnowulan, Munawar Gani, Satria Maulana, Nur Rahmi Ananda, Riat El Khair, Yunika Puspadewi, Osman Sianipar, Umi Solekhah Intansari, Elizabeth Henny Herningtiyas, Ira Puspitawati, Nur Imma Fatimah Harahap, Ernawati, Untung Wirawan , William Widitjiarso

		Integrated Research Center FK-KMK UGM; Department of Computer Science and Electronics FMIPA UGM; RSUP Dr. Sardjito	
EPI_ISL_632937	RSUD Saptosari Gunung Kidul	Genetics Working Group (Pokja Genetik) Faculty of Medicine, Public Health and Nursing Universitas Gadjah Mada (FK-KMK UGM); Disease Investigation Center Wates Ministry of Agriculture Indonesia; Department of Microbiology FK-KMK UGM; Laboratorium Diagnostik Yayasan Tahija World Mosquito Program (WMP) Yogyakarta Center for Tropical Medicine FK-KMK UGM; Integrated Research Center FK-KMK UGM; Department of Computer Science and Electronics FMIPA UGM; Balai Besar Teknik Kesehatan Lingkungan dan Pengendalian Penyakit (BBTKLPP) Yogyakarta	Gunadi, Hendra Wibawa, Marcellus, Mohamad S. Hakim, Edwin W. Daniwijaya, Ludhang P. Rizki, Endah Supriyati, Eggi Arguni, Titik Nuryastuti, Tri Wibawa, Dwi AA Nugrahaningsih, Afiahayati, Siswanto, Kristy Iskandar, Nungki Anggorowati, Irene, Indaryati, Havid Setyawan, Eko Darmawan, Maria Patricia Inggriani, Audric Kenny Tedja
EPI_ISL_632939	Jamaica Hospital Medical Center	New York City Public Health Laboratory	Jade Wang, et al.
EPI_ISL_632940	Richmond University Medical Center	New York City Public Health Laboratory	Jade Wang, et al.
EPI_ISL_632941	Jamaica Hospital Medical Center	New York City Public Health Laboratory	Jade Wang, et al.
EPI_ISL_632942	Richmond University Medical Center	New York City Public Health Laboratory	Jade Wang, et al.
EPI_ISL_632944	Jamaica Hospital Medical Center	New York City Public Health Laboratory	Jade Wang, et al.
EPI_ISL_632945, EPI_ISL_632946	OCME Office Of Chief Medical Examiner	New York City Public Health Laboratory	Jade Wang, et al.
EPI_ISL_632947	Flushing Hospital Medical Center	New York City Public Health Laboratory	Jade Wang, et al.
EPI_ISL_632948	Richmond University Medical Center	New York City Public Health Laboratory	Jade Wang, et al.
EPI_ISL_632949, EPI_ISL_632950	Jamaica Hospital Medical Center	New York City Public Health Laboratory	Jade Wang, et al.
EPI_ISL_632951, EPI_ISL_632952	Flushing Hospital Medical Center	New York City Public Health Laboratory	Jade Wang, et al.
EPI_ISL_632953	Jamaica Hospital Medical Center	New York City Public Health Laboratory	Jade Wang, et al.
EPI_ISL_632954, EPI_ISL_632955, EPI_ISL_632956	Flushing Hospital Medical Center	New York City Public Health Laboratory	Jade Wang, et al.
EPI_ISL_632957, EPI_ISL_632958	Jamaica Hospital Medical Center	New York City Public Health Laboratory	Jade Wang, et al.
EPI_ISL_632959	Richmond University Medical Center	New York City Public Health Laboratory	Jade Wang, et al.
EPI_ISL_632960	Flushing Hospital Medical Center	New York City Public Health Laboratory	Jade Wang, et al.
EPI_ISL_632961	Jamaica Hospital Medical Center	New York City Public Health Laboratory	Jade Wang, et al.
EPI_ISL_632962	Richmond University Medical Center	New York City Public Health Laboratory	Jade Wang, et al.
EPI_ISL_632963	NYC Department Of Health And Mental Hygiene	New York City Public Health Laboratory	Jade Wang, et al.
EPI_ISL_632964	Department of Homeless Services	New York City Public Health Laboratory	Jade Wang, et al.
EPI_ISL_632965	Richmond University Medical Center	New York City Public Health Laboratory	Jade Wang, et al.
EPI_ISL_632966, EPI_ISL_632967, EPI_ISL_632968	Flushing Hospital Medical Center	New York City Public Health Laboratory	Jade Wang, et al.
EPI_ISL_632969, EPI_ISL_632970, EPI_ISL_632971, EPI_ISL_632972, EPI_ISL_632974	Jamaica Hospital Medical Center	New York City Public Health Laboratory	Jade Wang, et al.
EPI_ISL_632976, EPI_ISL_632977, EPI_ISL_632979	Empire City Laboratories	New York City Public Health Laboratory	Jade Wang, et al.
EPI_ISL_632980, EPI_ISL_632981, EPI_ISL_632982, EPI_ISL_632984, EPI_ISL_632985, EPI_ISL_632986, EPI_ISL_632987, EPI_ISL_632989, EPI_ISL_632990, EPI_ISL_632991, EPI_ISL_632992, EPI_ISL_632993			
see above	DOHMH Corona	New York City Public Health Laboratory	Jade Wang, et al.
EPI_ISL_632994, EPI_ISL_632995, EPI_ISL_632996, EPI_ISL_632997, EPI_ISL_632998	DOHMH Crown Heights	New York City Public Health Laboratory	Jade Wang, et al.
EPI_ISL_632999, EPI_ISL_633000, EPI_ISL_633001, EPI_ISL_633002, EPI_ISL_633003, EPI_ISL_633004, EPI_ISL_633005, EPI_ISL_633006, EPI_ISL_633007, EPI_ISL_633008	DOHMH Jamaica	New York City Public Health Laboratory	Jade Wang, et al.
EPI_ISL_633009, EPI_ISL_633010	DOHMH Central Harlem	New York City Public Health Laboratory	Jade Wang, et al.
EPI_ISL_633011	DOHMH PHL	New York City Public Health Laboratory	Jade Wang, et al.
EPI_ISL_633012	DOHMH Riverside	New York City Public Health Laboratory	Jade Wang, et al.
EPI_ISL_633013	DOHMH PHL	New York City Public Health Laboratory	Jade Wang, et al.
EPI_ISL_633014	DOHMH Chelsea	New York City Public Health Laboratory	Jade Wang, et al.
EPI_ISL_633015	DOHMH Morrisania	New York City Public Health Laboratory	Jade Wang, et al.
EPI_ISL_633016, EPI_ISL_633017	DOHMH Chelsea	New York City Public Health Laboratory	Jade Wang, et al.
EPI_ISL_633019	DOHMH Morrisania	New York City Public Health Laboratory	Jade Wang, et al.
EPI_ISL_633025	DOHMH Chelsea	New York City Public Health Laboratory	Jade Wang, et al.
EPI_ISL_633027, EPI_ISL_633029, EPI_ISL_633030	DOHMH Morrisania	New York City Public Health Laboratory	Jade Wang, et al.
EPI_ISL_633031	DOHMH Chelsea	New York City Public Health Laboratory	Jade Wang, et al.
EPI_ISL_633032	DOHMH Morrisania	New York City Public Health Laboratory	Jade Wang, et al.
EPI_ISL_633033	DOHMH Chelsea	New York City Public Health Laboratory	Jade Wang, et al.
EPI_ISL_633034	DOHMH Riverside	New York City Public Health Laboratory	Jade Wang, et al.
EPI_ISL_633035	DOHMH PHL	New York City Public Health Laboratory	Jade Wang, et al.
EPI_ISL_633036	DOHMH Morrisania	New York City Public Health Laboratory	Jade Wang, et al.
EPI_ISL_633037	DOHMH Central Harlem	New York City Public Health Laboratory	Jade Wang, et al.
EPI_ISL_633038	DOHMH PHL	New York City Public Health Laboratory	Jade Wang, et al.

EPI_ISL_633039	DOHMH Chelsea	New York City Public Health Laboratory	Jade Wang, et al.
EPI_ISL_633041, EPI_ISL_633042	DOHMH Riverside	New York City Public Health Laboratory	Jade Wang, et al.
EPI_ISL_633044	DOHMH Morrisania	New York City Public Health Laboratory	Jade Wang, et al.
EPI_ISL_633045, EPI_ISL_633055	DOHMH PHL	New York City Public Health Laboratory	Jade Wang, et al.
EPI_ISL_633059	DOHMH Corona	New York City Public Health Laboratory	Jade Wang, et al.
EPI_ISL_633062	DOHMH Jamaica	New York City Public Health Laboratory	Jade Wang, et al.
EPI_ISL_633067	DOHMH Morrisania	New York City Public Health Laboratory	Jade Wang, et al.
EPI_ISL_633071	DOHMH PHL	New York City Public Health Laboratory	Jade Wang, et al.
EPI_ISL_633072	DOHMH Riverside	New York City Public Health Laboratory	Jade Wang, et al.
EPI_ISL_633073	DOHMH Fort Greene	New York City Public Health Laboratory	Jade Wang, et al.
EPI_ISL_633074	DOHMH Corona	New York City Public Health Laboratory	Jade Wang, et al.
EPI_ISL_633075	DOHMH Morrisania	New York City Public Health Laboratory	Jade Wang, et al.
EPI_ISL_633076, EPI_ISL_633078	DOHMH Chelsea	New York City Public Health Laboratory	Jade Wang, et al.
EPI_ISL_633079	DOHMH PHL	New York City Public Health Laboratory	Jade Wang, et al.
EPI_ISL_633080, EPI_ISL_633081, EPI_ISL_633082	DOHMH Morrisania	New York City Public Health Laboratory	Jade Wang, et al.
EPI_ISL_633083, EPI_ISL_633084, EPI_ISL_633085, EPI_ISL_633086, EPI_ISL_633087, EPI_ISL_633088, EPI_ISL_633089, EPI_ISL_633090, EPI_ISL_633092, EPI_ISL_633093, EPI_ISL_633094, EPI_ISL_633095, EPI_ISL_633096, EPI_ISL_633097, EPI_ISL_633098, EPI_ISL_633099			
see above	MD PHL	MD PHL	Maryland Department of Health Laboratories Administration
EPI_ISL_633100, EPI_ISL_633101, EPI_ISL_633102	Lighthouse Lab in Cambridge	Wellcome Sanger Institute for the COVID-19 Genomics UK (COG-UK) consortium	Rob Howes, The Lighthouse Lab in Cambridge and Alex Alderton, Roberto Amato, Sonia Goncalves, Ewan Harrison, David K. Jackson, Ian Johnston, Dominic Kwiatkowski, Cordelia Langford, John Sillitoe on behalf of the Wellcome Sanger Institute COVID-19 Surveillance Team
EPI_ISL_633103	Lighthouse Lab in Glasgow	Wellcome Sanger Institute for the COVID-19 Genomics UK (COG-UK) consortium	Harper VanSteenhouse, Yumi Kasai, David Gray, Carol Clugston, Anna Dominiczak and Alex Alderton, Roberto Amato, Sonia Goncalves, Ewan Harrison, David K. Jackson, Ian Johnston, Dominic Kwiatkowski, Cordelia Langford, John Sillitoe on behalf of the Wellcome Sanger Institute COVID-19 Surveillance Team
EPI_ISL_633104, EPI_ISL_633105, EPI_ISL_633106, EPI_ISL_633107, EPI_ISL_633109, EPI_ISL_633110	Lighthouse Lab in Cambridge	Wellcome Sanger Institute for the COVID-19 Genomics UK (COG-UK) consortium	Rob Howes, The Lighthouse Lab in Cambridge and Alex Alderton, Roberto Amato, Sonia Goncalves, Ewan Harrison, David K. Jackson, Ian Johnston, Dominic Kwiatkowski, Cordelia Langford, John Sillitoe on behalf of the Wellcome Sanger Institute COVID-19 Surveillance Team
EPI_ISL_633111, EPI_ISL_633112, EPI_ISL_633113, EPI_ISL_633115	Lighthouse Lab in Glasgow	Wellcome Sanger Institute for the COVID-19 Genomics UK (COG-UK) consortium	Harper VanSteenhouse, Yumi Kasai, David Gray, Carol Clugston, Anna Dominiczak and Alex Alderton, Roberto Amato, Sonia Goncalves, Ewan Harrison, David K. Jackson, Ian Johnston, Dominic Kwiatkowski, Cordelia Langford, John Sillitoe on behalf of the Wellcome Sanger Institute COVID-19 Surveillance Team
EPI_ISL_633116, EPI_ISL_633117	Lighthouse Lab in Cambridge	Wellcome Sanger Institute for the COVID-19 Genomics UK (COG-UK) consortium	Rob Howes, The Lighthouse Lab in Cambridge and Alex Alderton, Roberto Amato, Sonia Goncalves, Ewan Harrison, David K. Jackson, Ian Johnston, Dominic Kwiatkowski, Cordelia Langford, John Sillitoe on behalf of the Wellcome Sanger Institute COVID-19 Surveillance Team
EPI_ISL_633119	Lighthouse Lab in Glasgow	Wellcome Sanger Institute for the COVID-19 Genomics UK (COG-UK) consortium	Harper VanSteenhouse, Yumi Kasai, David Gray, Carol Clugston, Anna Dominiczak and Alex Alderton, Roberto Amato, Sonia Goncalves, Ewan Harrison, David K. Jackson, Ian Johnston, Dominic Kwiatkowski, Cordelia Langford, John Sillitoe on behalf of the Wellcome Sanger Institute COVID-19 Surveillance Team
EPI_ISL_633120, EPI_ISL_633121, EPI_ISL_633122, EPI_ISL_633123, EPI_ISL_633124	Lighthouse Lab in Cambridge	Wellcome Sanger Institute for the COVID-19 Genomics UK (COG-UK) consortium	Rob Howes, The Lighthouse Lab in Cambridge and Alex Alderton, Roberto Amato, Sonia Goncalves, Ewan Harrison, David K. Jackson, Ian Johnston, Dominic Kwiatkowski, Cordelia Langford, John Sillitoe on behalf of the Wellcome Sanger Institute COVID-19 Surveillance Team
EPI_ISL_633125	Lighthouse Lab in Glasgow	Wellcome Sanger Institute for the COVID-19 Genomics UK (COG-UK) consortium	Harper VanSteenhouse, Yumi Kasai, David Gray, Carol Clugston, Anna Dominiczak and Alex Alderton, Roberto Amato, Sonia Goncalves, Ewan Harrison, David K. Jackson, Ian Johnston, Dominic Kwiatkowski, Cordelia Langford, John Sillitoe on behalf of the Wellcome Sanger Institute COVID-19 Surveillance Team
EPI_ISL_633126, EPI_ISL_633127, EPI_ISL_633128, EPI_ISL_633129, EPI_ISL_633130, EPI_ISL_633131	Lighthouse Lab in Cambridge	Wellcome Sanger Institute for the COVID-19 Genomics UK (COG-UK) consortium	Rob Howes, The Lighthouse Lab in Cambridge and Alex Alderton, Roberto Amato, Sonia Goncalves, Ewan Harrison, David K. Jackson, Ian Johnston, Dominic Kwiatkowski, Cordelia Langford, John Sillitoe on behalf of the Wellcome Sanger Institute COVID-19 Surveillance Team
EPI_ISL_633132	Lighthouse Lab in Glasgow	Wellcome Sanger Institute for the COVID-19 Genomics UK (COG-UK) consortium	Harper VanSteenhouse, Yumi Kasai, David Gray, Carol Clugston, Anna Dominiczak and Alex Alderton, Roberto Amato, Sonia Goncalves, Ewan Harrison, David K. Jackson, Ian Johnston, Dominic Kwiatkowski, Cordelia Langford, John Sillitoe on behalf of the Wellcome Sanger Institute COVID-19 Surveillance Team
EPI_ISL_633135, EPI_ISL_633136	Lighthouse Lab in Cambridge	Wellcome Sanger Institute for the COVID-19 Genomics UK (COG-UK) consortium	Rob Howes, The Lighthouse Lab in Cambridge and Alex Alderton, Roberto Amato, Sonia Goncalves, Ewan Harrison, David K. Jackson, Ian Johnston, Dominic Kwiatkowski, Cordelia Langford, John Sillitoe on behalf of the Wellcome Sanger Institute COVID-19 Surveillance Team
EPI_ISL_633137, EPI_ISL_633138, EPI_ISL_633139	Lighthouse Lab in Glasgow	Wellcome Sanger Institute for the COVID-19 Genomics UK (COG-UK) consortium	Harper VanSteenhouse, Yumi Kasai, David Gray, Carol Clugston, Anna Dominiczak and Alex Alderton, Roberto Amato, Sonia Goncalves, Ewan Harrison, David K. Jackson, Ian Johnston, Dominic Kwiatkowski, Cordelia Langford, John Sillitoe on behalf of the Wellcome Sanger Institute COVID-19 Surveillance Team
EPI_ISL_633140, EPI_ISL_633141, EPI_ISL_633142	Lighthouse Lab in Cambridge	Wellcome Sanger Institute for the COVID-19 Genomics UK (COG-UK) consortium	Rob Howes, The Lighthouse Lab in Cambridge and Alex Alderton, Roberto Amato, Sonia Goncalves, Ewan Harrison, David K. Jackson, Ian Johnston, Dominic Kwiatkowski, Cordelia Langford, John Sillitoe on behalf of the Wellcome Sanger Institute COVID-19 Surveillance Team
EPI_ISL_633143, EPI_ISL_633144, EPI_ISL_633145, EPI_ISL_633146	Lighthouse Lab in Glasgow	Wellcome Sanger Institute for the COVID-19 Genomics UK (COG-UK) consortium	Harper VanSteenhouse, Yumi Kasai, David Gray, Carol Clugston, Anna Dominiczak and Alex Alderton, Roberto Amato, Sonia Goncalves, Ewan Harrison, David K. Jackson, Ian Johnston, Dominic Kwiatkowski, Cordelia Langford, John Sillitoe on behalf of the Wellcome Sanger Institute COVID-19 Surveillance Team
EPI_ISL_633147, EPI_ISL_633148, EPI_ISL_633149, EPI_ISL_633150	Lighthouse Lab in Cambridge	Wellcome Sanger Institute for the COVID-19 Genomics UK (COG-UK) consortium	Rob Howes, The Lighthouse Lab in Cambridge and Alex Alderton, Roberto Amato, Sonia Goncalves, Ewan Harrison, David K. Jackson, Ian Johnston, Dominic Kwiatkowski, Cordelia Langford, John Sillitoe on behalf of the Wellcome Sanger Institute COVID-19 Surveillance Team
EPI_ISL_633151, EPI_ISL_633152	Lighthouse Lab in Glasgow	Wellcome Sanger Institute for the COVID-19 Genomics UK (COG-UK) consortium	Harper VanSteenhouse, Yumi Kasai, David Gray, Carol Clugston, Anna Dominiczak and Alex Alderton, Roberto Amato, Sonia Goncalves, Ewan Harrison, David K. Jackson, Ian Johnston, Dominic Kwiatkowski, Cordelia Langford, John Sillitoe on behalf of the Wellcome Sanger Institute COVID-19 Surveillance Team
EPI_ISL_633153	Lighthouse Lab in Cambridge	Wellcome Sanger Institute for the COVID-19 Genomics UK (COG-UK) consortium	Rob Howes, The Lighthouse Lab in Cambridge and Alex Alderton, Roberto Amato, Sonia Goncalves, Ewan Harrison, David K. Jackson, Ian Johnston, Dominic Kwiatkowski, Cordelia Langford, John Sillitoe on behalf of the Wellcome Sanger Institute COVID-19 Surveillance Team
EPI_ISL_633154	Lighthouse Lab in Glasgow	Wellcome Sanger Institute for the COVID-19 Genomics UK (COG-UK) consortium	Harper VanSteenhouse, Yumi Kasai, David Gray, Carol Clugston, Anna Dominiczak and Alex Alderton, Roberto Amato, Sonia Goncalves, Ewan Harrison, David K. Jackson, Ian Johnston, Dominic Kwiatkowski, Cordelia Langford, John Sillitoe on behalf of the Wellcome Sanger Institute COVID-19 Surveillance Team
EPI_ISL_633155, EPI_ISL_633156, EPI_ISL_633157	Lighthouse Lab in Cambridge	Wellcome Sanger Institute for the COVID-19 Genomics UK (COG-UK) consortium	Rob Howes, The Lighthouse Lab in Cambridge and Alex Alderton, Roberto Amato, Sonia Goncalves, Ewan Harrison, David K. Jackson, Ian Johnston, Dominic Kwiatkowski, Cordelia Langford, John Sillitoe on behalf of the Wellcome Sanger Institute COVID-19 Surveillance Team
EPI_ISL_633158	Lighthouse Lab in Glasgow	Wellcome Sanger Institute for the COVID-19 Genomics UK (COG-UK) consortium	Harper VanSteenhouse, Yumi Kasai, David Gray, Carol Clugston, Anna Dominiczak and Alex Alderton, Roberto Amato, Sonia Goncalves, Ewan Harrison, David K. Jackson, Ian Johnston, Dominic Kwiatkowski, Cordelia Langford, John Sillitoe on behalf of the Wellcome Sanger Institute COVID-19 Surveillance Team
EPI_ISL_633159	Lighthouse Lab in Cambridge	Wellcome Sanger Institute for the COVID-19 Genomics UK (COG-UK) consortium	Rob Howes, The Lighthouse Lab in Cambridge and Alex Alderton, Roberto Amato, Sonia Goncalves, Ewan Harrison, David K. Jackson, Ian Johnston, Dominic Kwiatkowski, Cordelia Langford, John Sillitoe on behalf of the Wellcome Sanger Institute COVID-19 Surveillance Team

EPI_ISL_633160	Lighthouse Lab in Glasgow	Wellcome Sanger Institute for the COVID-19 Genomics UK (COG-UK) consortium	Harper VanSteenhouse, Yumi Kasai, David Gray, Carol Clugston, Anna Dominiczak and Alex Alderton, Roberto Amato, Sonia Goncalves, Ewan Harrison, David K. Jackson, Ian Johnston, Dominic Kwiatkowski, Cordelia Langford, John Sillitoe on behalf of the Wellcome Sanger Institute COVID-19 Surveillance Team
EPI_ISL_633161	Lighthouse Lab in Cambridge	Wellcome Sanger Institute for the COVID-19 Genomics UK (COG-UK) consortium	Rob Howes, The Lighthouse Lab in Cambridge and Alex Alderton, Roberto Amato, Sonia Goncalves, Ewan Harrison, David K. Jackson, Ian Johnston, Dominic Kwiatkowski, Cordelia Langford, John Sillitoe on behalf of the Wellcome Sanger Institute COVID-19 Surveillance Team
EPI_ISL_633162	Lighthouse Lab in Glasgow	Wellcome Sanger Institute for the COVID-19 Genomics UK (COG-UK) consortium	Harper VanSteenhouse, Yumi Kasai, David Gray, Carol Clugston, Anna Dominiczak and Alex Alderton, Roberto Amato, Sonia Goncalves, Ewan Harrison, David K. Jackson, Ian Johnston, Dominic Kwiatkowski, Cordelia Langford, John Sillitoe on behalf of the Wellcome Sanger Institute COVID-19 Surveillance Team
EPI_ISL_633163, EPI_ISL_633164	Lighthouse Lab in Cambridge	Wellcome Sanger Institute for the COVID-19 Genomics UK (COG-UK) consortium	Rob Howes, The Lighthouse Lab in Cambridge and Alex Alderton, Roberto Amato, Sonia Goncalves, Ewan Harrison, David K. Jackson, Ian Johnston, Dominic Kwiatkowski, Cordelia Langford, John Sillitoe on behalf of the Wellcome Sanger Institute COVID-19 Surveillance Team
EPI_ISL_633166, EPI_ISL_633167, EPI_ISL_633168, EPI_ISL_633169	Lighthouse Lab in Glasgow	Wellcome Sanger Institute for the COVID-19 Genomics UK (COG-UK) consortium	Harper VanSteenhouse, Yumi Kasai, David Gray, Carol Clugston, Anna Dominiczak and Alex Alderton, Roberto Amato, Sonia Goncalves, Ewan Harrison, David K. Jackson, Ian Johnston, Dominic Kwiatkowski, Cordelia Langford, John Sillitoe on behalf of the Wellcome Sanger Institute COVID-19 Surveillance Team
EPI_ISL_633170	Lighthouse Lab in Cambridge	Wellcome Sanger Institute for the COVID-19 Genomics UK (COG-UK) consortium	Rob Howes, The Lighthouse Lab in Cambridge and Alex Alderton, Roberto Amato, Sonia Goncalves, Ewan Harrison, David K. Jackson, Ian Johnston, Dominic Kwiatkowski, Cordelia Langford, John Sillitoe on behalf of the Wellcome Sanger Institute COVID-19 Surveillance Team
EPI_ISL_633171	Lighthouse Lab in Glasgow	Wellcome Sanger Institute for the COVID-19 Genomics UK (COG-UK) consortium	Harper VanSteenhouse, Yumi Kasai, David Gray, Carol Clugston, Anna Dominiczak and Alex Alderton, Roberto Amato, Sonia Goncalves, Ewan Harrison, David K. Jackson, Ian Johnston, Dominic Kwiatkowski, Cordelia Langford, John Sillitoe on behalf of the Wellcome Sanger Institute COVID-19 Surveillance Team
EPI_ISL_633173	Lighthouse Lab in Cambridge	Wellcome Sanger Institute for the COVID-19 Genomics UK (COG-UK) consortium	Rob Howes, The Lighthouse Lab in Cambridge and Alex Alderton, Roberto Amato, Sonia Goncalves, Ewan Harrison, David K. Jackson, Ian Johnston, Dominic Kwiatkowski, Cordelia Langford, John Sillitoe on behalf of the Wellcome Sanger Institute COVID-19 Surveillance Team
EPI_ISL_633174, EPI_ISL_633175	Lighthouse Lab in Glasgow	Wellcome Sanger Institute for the COVID-19 Genomics UK (COG-UK) consortium	Harper VanSteenhouse, Yumi Kasai, David Gray, Carol Clugston, Anna Dominiczak and Alex Alderton, Roberto Amato, Sonia Goncalves, Ewan Harrison, David K. Jackson, Ian Johnston, Dominic Kwiatkowski, Cordelia Langford, John Sillitoe on behalf of the Wellcome Sanger Institute COVID-19 Surveillance Team
EPI_ISL_633176, EPI_ISL_633177	Lighthouse Lab in Cambridge	Wellcome Sanger Institute for the COVID-19 Genomics UK (COG-UK) consortium	Rob Howes, The Lighthouse Lab in Cambridge and Alex Alderton, Roberto Amato, Sonia Goncalves, Ewan Harrison, David K. Jackson, Ian Johnston, Dominic Kwiatkowski, Cordelia Langford, John Sillitoe on behalf of the Wellcome Sanger Institute COVID-19 Surveillance Team
EPI_ISL_633178, EPI_ISL_633179, EPI_ISL_633180, EPI_ISL_633181, EPI_ISL_633182	Lighthouse Lab in Glasgow	Wellcome Sanger Institute for the COVID-19 Genomics UK (COG-UK) consortium	Harper VanSteenhouse, Yumi Kasai, David Gray, Carol Clugston, Anna Dominiczak and Alex Alderton, Roberto Amato, Sonia Goncalves, Ewan Harrison, David K. Jackson, Ian Johnston, Dominic Kwiatkowski, Cordelia Langford, John Sillitoe on behalf of the Wellcome Sanger Institute COVID-19 Surveillance Team
EPI_ISL_633183	Lighthouse Lab in Cambridge	Wellcome Sanger Institute for the COVID-19 Genomics UK (COG-UK) consortium	Rob Howes, The Lighthouse Lab in Cambridge and Alex Alderton, Roberto Amato, Sonia Goncalves, Ewan Harrison, David K. Jackson, Ian Johnston, Dominic Kwiatkowski, Cordelia Langford, John Sillitoe on behalf of the Wellcome Sanger Institute COVID-19 Surveillance Team
EPI_ISL_633184, EPI_ISL_633185	Lighthouse Lab in Glasgow	Wellcome Sanger Institute for the COVID-19 Genomics UK (COG-UK) consortium	Harper VanSteenhouse, Yumi Kasai, David Gray, Carol Clugston, Anna Dominiczak and Alex Alderton, Roberto Amato, Sonia Goncalves, Ewan Harrison, David K. Jackson, Ian Johnston, Dominic Kwiatkowski, Cordelia Langford, John Sillitoe on behalf of the Wellcome Sanger Institute COVID-19 Surveillance Team
EPI_ISL_633186, EPI_ISL_633187, EPI_ISL_633188, EPI_ISL_633189	Lighthouse Lab in Cambridge	Wellcome Sanger Institute for the COVID-19 Genomics UK (COG-UK) consortium	Rob Howes, The Lighthouse Lab in Cambridge and Alex Alderton, Roberto Amato, Sonia Goncalves, Ewan Harrison, David K. Jackson, Ian Johnston, Dominic Kwiatkowski, Cordelia Langford, John Sillitoe on behalf of the Wellcome Sanger Institute COVID-19 Surveillance Team
EPI_ISL_633190, EPI_ISL_633191	Lighthouse Lab in Glasgow	Wellcome Sanger Institute for the COVID-19 Genomics UK (COG-UK) consortium	Harper VanSteenhouse, Yumi Kasai, David Gray, Carol Clugston, Anna Dominiczak and Alex Alderton, Roberto Amato, Sonia Goncalves, Ewan Harrison, David K. Jackson, Ian Johnston, Dominic Kwiatkowski, Cordelia Langford, John Sillitoe on behalf of the Wellcome Sanger Institute COVID-19 Surveillance Team
EPI_ISL_633192	Lighthouse Lab in Cambridge	Wellcome Sanger Institute for the COVID-19 Genomics UK (COG-UK) consortium	Rob Howes, The Lighthouse Lab in Cambridge and Alex Alderton, Roberto Amato, Sonia Goncalves, Ewan Harrison, David K. Jackson, Ian Johnston, Dominic Kwiatkowski, Cordelia Langford, John Sillitoe on behalf of the Wellcome Sanger Institute COVID-19 Surveillance Team
EPI_ISL_633193	Lighthouse Lab in Glasgow	Wellcome Sanger Institute for the COVID-19 Genomics UK (COG-UK) consortium	Harper VanSteenhouse, Yumi Kasai, David Gray, Carol Clugston, Anna Dominiczak and Alex Alderton, Roberto Amato, Sonia Goncalves, Ewan Harrison, David K. Jackson, Ian Johnston, Dominic Kwiatkowski, Cordelia Langford, John Sillitoe on behalf of the Wellcome Sanger Institute COVID-19 Surveillance Team
EPI_ISL_633194	Lighthouse Lab in Cambridge	Wellcome Sanger Institute for the COVID-19 Genomics UK (COG-UK) consortium	Rob Howes, The Lighthouse Lab in Cambridge and Alex Alderton, Roberto Amato, Sonia Goncalves, Ewan Harrison, David K. Jackson, Ian Johnston, Dominic Kwiatkowski, Cordelia Langford, John Sillitoe on behalf of the Wellcome Sanger Institute COVID-19 Surveillance Team
EPI_ISL_633196	Lighthouse Lab in Glasgow	Wellcome Sanger Institute for the COVID-19 Genomics UK (COG-UK) consortium	Harper VanSteenhouse, Yumi Kasai, David Gray, Carol Clugston, Anna Dominiczak and Alex Alderton, Roberto Amato, Sonia Goncalves, Ewan Harrison, David K. Jackson, Ian Johnston, Dominic Kwiatkowski, Cordelia Langford, John Sillitoe on behalf of the Wellcome Sanger Institute COVID-19 Surveillance Team
EPI_ISL_633197, EPI_ISL_633199, EPI_ISL_633200	Lighthouse Lab in Cambridge	Wellcome Sanger Institute for the COVID-19 Genomics UK (COG-UK) consortium	Rob Howes, The Lighthouse Lab in Cambridge and Alex Alderton, Roberto Amato, Sonia Goncalves, Ewan Harrison, David K. Jackson, Ian Johnston, Dominic Kwiatkowski, Cordelia Langford, John Sillitoe on behalf of the Wellcome Sanger Institute COVID-19 Surveillance Team
EPI_ISL_633202, EPI_ISL_633203, EPI_ISL_633204	Lighthouse Lab in Glasgow	Wellcome Sanger Institute for the COVID-19 Genomics UK (COG-UK) consortium	Harper VanSteenhouse, Yumi Kasai, David Gray, Carol Clugston, Anna Dominiczak and Alex Alderton, Roberto Amato, Sonia Goncalves, Ewan Harrison, David K. Jackson, Ian Johnston, Dominic Kwiatkowski, Cordelia Langford, John Sillitoe on behalf of the Wellcome Sanger Institute COVID-19 Surveillance Team
EPI_ISL_633205	Lighthouse Lab in Cambridge	Wellcome Sanger Institute for the COVID-19 Genomics UK (COG-UK) consortium	Rob Howes, The Lighthouse Lab in Cambridge and Alex Alderton, Roberto Amato, Sonia Goncalves, Ewan Harrison, David K. Jackson, Ian Johnston, Dominic Kwiatkowski, Cordelia Langford, John Sillitoe on behalf of the Wellcome Sanger Institute COVID-19 Surveillance Team
EPI_ISL_633206, EPI_ISL_633207	Lighthouse Lab in Glasgow	Wellcome Sanger Institute for the COVID-19 Genomics UK (COG-UK) consortium	Harper VanSteenhouse, Yumi Kasai, David Gray, Carol Clugston, Anna Dominiczak and Alex Alderton, Roberto Amato, Sonia Goncalves, Ewan Harrison, David K. Jackson, Ian Johnston, Dominic Kwiatkowski, Cordelia Langford, John Sillitoe on behalf of the Wellcome Sanger Institute COVID-19 Surveillance Team
EPI_ISL_633208	Lighthouse Lab in Cambridge	Wellcome Sanger Institute for the COVID-19 Genomics UK (COG-UK) consortium	Rob Howes, The Lighthouse Lab in Cambridge and Alex Alderton, Roberto Amato, Sonia Goncalves, Ewan Harrison, David K. Jackson, Ian Johnston, Dominic Kwiatkowski, Cordelia Langford, John Sillitoe on behalf of the Wellcome Sanger Institute COVID-19 Surveillance Team
EPI_ISL_633209, EPI_ISL_633210	Lighthouse Lab in Glasgow	Wellcome Sanger Institute for the COVID-19 Genomics UK (COG-UK) consortium	Harper VanSteenhouse, Yumi Kasai, David Gray, Carol Clugston, Anna Dominiczak and Alex Alderton, Roberto Amato, Sonia Goncalves, Ewan Harrison, David K. Jackson, Ian Johnston, Dominic Kwiatkowski, Cordelia Langford, John Sillitoe on behalf of the Wellcome Sanger Institute COVID-19 Surveillance Team
EPI_ISL_633211, EPI_ISL_633212	Lighthouse Lab in Cambridge	Wellcome Sanger Institute for the COVID-19 Genomics UK (COG-UK) consortium	Rob Howes, The Lighthouse Lab in Cambridge and Alex Alderton, Roberto Amato, Sonia Goncalves, Ewan Harrison, David K. Jackson, Ian Johnston, Dominic Kwiatkowski, Cordelia Langford, John Sillitoe on behalf of the Wellcome Sanger Institute COVID-19 Surveillance Team
EPI_ISL_633213	Lighthouse Lab in Glasgow	Wellcome Sanger Institute for the COVID-19 Genomics UK (COG-UK) consortium	Harper VanSteenhouse, Yumi Kasai, David Gray, Carol Clugston, Anna Dominiczak and Alex Alderton, Roberto Amato, Sonia Goncalves, Ewan Harrison, David K. Jackson, Ian Johnston, Dominic Kwiatkowski, Cordelia Langford, John Sillitoe on behalf of the Wellcome Sanger Institute COVID-19 Surveillance Team
EPI_ISL_633214, EPI_ISL_633215, EPI_ISL_633216, EPI_ISL_633217, EPI_ISL_633219, EPI_ISL_633220	Lighthouse Lab in Cambridge	Wellcome Sanger Institute for the COVID-19 Genomics UK (COG-UK) consortium	Rob Howes, The Lighthouse Lab in Cambridge and Alex Alderton, Roberto Amato, Sonia Goncalves, Ewan Harrison, David K. Jackson, Ian Johnston, Dominic Kwiatkowski, Cordelia Langford, John Sillitoe on behalf of the Wellcome Sanger Institute COVID-19 Surveillance Team
EPI_ISL_633222	Lighthouse Lab in Glasgow	Wellcome Sanger Institute for the COVID-19 Genomics UK (COG-UK) consortium	Harper VanSteenhouse, Yumi Kasai, David Gray, Carol Clugston, Anna Dominiczak and Alex Alderton, Roberto Amato, Sonia Goncalves, Ewan Harrison, David K. Jackson, Ian Johnston, Dominic Kwiatkowski, Cordelia Langford, John Sillitoe on behalf of the Wellcome Sanger Institute COVID-19 Surveillance Team

EPI_ISL_633223, EPI_ISL_633224, EPI_ISL_633225	Lighthouse Lab in Cambridge	Wellcome Sanger Institute for the COVID-19 Genomics UK (COG-UK) consortium	Rob Howes, The Lighthouse Lab in Cambridge and Alex Alderton, Roberto Amato, Sonia Goncalves, Ewan Harrison, David K. Jackson, Ian Johnston, Dominic Kwiatkowski, Cordelia Langford, John Sillitoe on behalf of the Wellcome Sanger Institute COVID-19 Surveillance Team
EPI_ISL_633226	Lighthouse Lab in Glasgow	Wellcome Sanger Institute for the COVID-19 Genomics UK (COG-UK) consortium	Harper VanSteenhouse, Yumi Kasai, David Gray, Carol Clugston, Anna Dominiczak and Alex Alderton, Roberto Amato, Sonia Goncalves, Ewan Harrison, David K. Jackson, Ian Johnston, Dominic Kwiatkowski, Cordelia Langford, John Sillitoe on behalf of the Wellcome Sanger Institute COVID-19 Surveillance Team
EPI_ISL_633227	Lighthouse Lab in Cambridge	Wellcome Sanger Institute for the COVID-19 Genomics UK (COG-UK) consortium	Rob Howes, The Lighthouse Lab in Cambridge and Alex Alderton, Roberto Amato, Sonia Goncalves, Ewan Harrison, David K. Jackson, Ian Johnston, Dominic Kwiatkowski, Cordelia Langford, John Sillitoe on behalf of the Wellcome Sanger Institute COVID-19 Surveillance Team
EPI_ISL_633228	Lighthouse Lab in Glasgow	Wellcome Sanger Institute for the COVID-19 Genomics UK (COG-UK) consortium	Harper VanSteenhouse, Yumi Kasai, David Gray, Carol Clugston, Anna Dominiczak and Alex Alderton, Roberto Amato, Sonia Goncalves, Ewan Harrison, David K. Jackson, Ian Johnston, Dominic Kwiatkowski, Cordelia Langford, John Sillitoe on behalf of the Wellcome Sanger Institute COVID-19 Surveillance Team
EPI_ISL_633229	Lighthouse Lab in Cambridge	Wellcome Sanger Institute for the COVID-19 Genomics UK (COG-UK) consortium	Rob Howes, The Lighthouse Lab in Cambridge and Alex Alderton, Roberto Amato, Sonia Goncalves, Ewan Harrison, David K. Jackson, Ian Johnston, Dominic Kwiatkowski, Cordelia Langford, John Sillitoe on behalf of the Wellcome Sanger Institute COVID-19 Surveillance Team
EPI_ISL_633230	Lighthouse Lab in Glasgow	Wellcome Sanger Institute for the COVID-19 Genomics UK (COG-UK) consortium	Harper VanSteenhouse, Yumi Kasai, David Gray, Carol Clugston, Anna Dominiczak and Alex Alderton, Roberto Amato, Sonia Goncalves, Ewan Harrison, David K. Jackson, Ian Johnston, Dominic Kwiatkowski, Cordelia Langford, John Sillitoe on behalf of the Wellcome Sanger Institute COVID-19 Surveillance Team
EPI_ISL_633231	Lighthouse Lab in Cambridge	Wellcome Sanger Institute for the COVID-19 Genomics UK (COG-UK) consortium	Rob Howes, The Lighthouse Lab in Cambridge and Alex Alderton, Roberto Amato, Sonia Goncalves, Ewan Harrison, David K. Jackson, Ian Johnston, Dominic Kwiatkowski, Cordelia Langford, John Sillitoe on behalf of the Wellcome Sanger Institute COVID-19 Surveillance Team
EPI_ISL_633232, EPI_ISL_633233	Lighthouse Lab in Glasgow	Wellcome Sanger Institute for the COVID-19 Genomics UK (COG-UK) consortium	Harper VanSteenhouse, Yumi Kasai, David Gray, Carol Clugston, Anna Dominiczak and Alex Alderton, Roberto Amato, Sonia Goncalves, Ewan Harrison, David K. Jackson, Ian Johnston, Dominic Kwiatkowski, Cordelia Langford, John Sillitoe on behalf of the Wellcome Sanger Institute COVID-19 Surveillance Team
EPI_ISL_633234, EPI_ISL_633235, EPI_ISL_633236	Lighthouse Lab in Cambridge	Wellcome Sanger Institute for the COVID-19 Genomics UK (COG-UK) consortium	Rob Howes, The Lighthouse Lab in Cambridge and Alex Alderton, Roberto Amato, Sonia Goncalves, Ewan Harrison, David K. Jackson, Ian Johnston, Dominic Kwiatkowski, Cordelia Langford, John Sillitoe on behalf of the Wellcome Sanger Institute COVID-19 Surveillance Team
EPI_ISL_633237	Lighthouse Lab in Glasgow	Wellcome Sanger Institute for the COVID-19 Genomics UK (COG-UK) consortium	Harper VanSteenhouse, Yumi Kasai, David Gray, Carol Clugston, Anna Dominiczak and Alex Alderton, Roberto Amato, Sonia Goncalves, Ewan Harrison, David K. Jackson, Ian Johnston, Dominic Kwiatkowski, Cordelia Langford, John Sillitoe on behalf of the Wellcome Sanger Institute COVID-19 Surveillance Team
EPI_ISL_633239	Lighthouse Lab in Cambridge	Wellcome Sanger Institute for the COVID-19 Genomics UK (COG-UK) consortium	Rob Howes, The Lighthouse Lab in Cambridge and Alex Alderton, Roberto Amato, Sonia Goncalves, Ewan Harrison, David K. Jackson, Ian Johnston, Dominic Kwiatkowski, Cordelia Langford, John Sillitoe on behalf of the Wellcome Sanger Institute COVID-19 Surveillance Team
EPI_ISL_633240, EPI_ISL_633241	Lighthouse Lab in Glasgow	Wellcome Sanger Institute for the COVID-19 Genomics UK (COG-UK) consortium	Harper VanSteenhouse, Yumi Kasai, David Gray, Carol Clugston, Anna Dominiczak and Alex Alderton, Roberto Amato, Sonia Goncalves, Ewan Harrison, David K. Jackson, Ian Johnston, Dominic Kwiatkowski, Cordelia Langford, John Sillitoe on behalf of the Wellcome Sanger Institute COVID-19 Surveillance Team
EPI_ISL_633242	Lighthouse Lab in Cambridge	Wellcome Sanger Institute for the COVID-19 Genomics UK (COG-UK) consortium	Rob Howes, The Lighthouse Lab in Cambridge and Alex Alderton, Roberto Amato, Sonia Goncalves, Ewan Harrison, David K. Jackson, Ian Johnston, Dominic Kwiatkowski, Cordelia Langford, John Sillitoe on behalf of the Wellcome Sanger Institute COVID-19 Surveillance Team
EPI_ISL_633243, EPI_ISL_633244	Lighthouse Lab in Glasgow	Wellcome Sanger Institute for the COVID-19 Genomics UK (COG-UK) consortium	Harper VanSteenhouse, Yumi Kasai, David Gray, Carol Clugston, Anna Dominiczak and Alex Alderton, Roberto Amato, Sonia Goncalves, Ewan Harrison, David K. Jackson, Ian Johnston, Dominic Kwiatkowski, Cordelia Langford, John Sillitoe on behalf of the Wellcome Sanger Institute COVID-19 Surveillance Team
EPI_ISL_633245, EPI_ISL_633246, EPI_ISL_633247, EPI_ISL_633248, EPI_ISL_633249, EPI_ISL_633250	Lighthouse Lab in Cambridge	Wellcome Sanger Institute for the COVID-19 Genomics UK (COG-UK) consortium	Rob Howes, The Lighthouse Lab in Cambridge and Alex Alderton, Roberto Amato, Sonia Goncalves, Ewan Harrison, David K. Jackson, Ian Johnston, Dominic Kwiatkowski, Cordelia Langford, John Sillitoe on behalf of the Wellcome Sanger Institute COVID-19 Surveillance Team
EPI_ISL_633251	Lighthouse Lab in Glasgow	Wellcome Sanger Institute for the COVID-19 Genomics UK (COG-UK) consortium	Harper VanSteenhouse, Yumi Kasai, David Gray, Carol Clugston, Anna Dominiczak and Alex Alderton, Roberto Amato, Sonia Goncalves, Ewan Harrison, David K. Jackson, Ian Johnston, Dominic Kwiatkowski, Cordelia Langford, John Sillitoe on behalf of the Wellcome Sanger Institute COVID-19 Surveillance Team
EPI_ISL_633252, EPI_ISL_633253, EPI_ISL_633254, EPI_ISL_633255	Lighthouse Lab in Cambridge	Wellcome Sanger Institute for the COVID-19 Genomics UK (COG-UK) consortium	Rob Howes, The Lighthouse Lab in Cambridge and Alex Alderton, Roberto Amato, Sonia Goncalves, Ewan Harrison, David K. Jackson, Ian Johnston, Dominic Kwiatkowski, Cordelia Langford, John Sillitoe on behalf of the Wellcome Sanger Institute COVID-19 Surveillance Team
EPI_ISL_633256	Lighthouse Lab in Glasgow	Wellcome Sanger Institute for the COVID-19 Genomics UK (COG-UK) consortium	Harper VanSteenhouse, Yumi Kasai, David Gray, Carol Clugston, Anna Dominiczak and Alex Alderton, Roberto Amato, Sonia Goncalves, Ewan Harrison, David K. Jackson, Ian Johnston, Dominic Kwiatkowski, Cordelia Langford, John Sillitoe on behalf of the Wellcome Sanger Institute COVID-19 Surveillance Team
EPI_ISL_633257, EPI_ISL_633258, EPI_ISL_633260	Lighthouse Lab in Cambridge	Wellcome Sanger Institute for the COVID-19 Genomics UK (COG-UK) consortium	Rob Howes, The Lighthouse Lab in Cambridge and Alex Alderton, Roberto Amato, Sonia Goncalves, Ewan Harrison, David K. Jackson, Ian Johnston, Dominic Kwiatkowski, Cordelia Langford, John Sillitoe on behalf of the Wellcome Sanger Institute COVID-19 Surveillance Team
EPI_ISL_633261	Lighthouse Lab in Glasgow	Wellcome Sanger Institute for the COVID-19 Genomics UK (COG-UK) consortium	Harper VanSteenhouse, Yumi Kasai, David Gray, Carol Clugston, Anna Dominiczak and Alex Alderton, Roberto Amato, Sonia Goncalves, Ewan Harrison, David K. Jackson, Ian Johnston, Dominic Kwiatkowski, Cordelia Langford, John Sillitoe on behalf of the Wellcome Sanger Institute COVID-19 Surveillance Team
EPI_ISL_633263, EPI_ISL_633264, EPI_ISL_633265, EPI_ISL_633266, EPI_ISL_633267	Lighthouse Lab in Cambridge	Wellcome Sanger Institute for the COVID-19 Genomics UK (COG-UK) consortium	Rob Howes, The Lighthouse Lab in Cambridge and Alex Alderton, Roberto Amato, Sonia Goncalves, Ewan Harrison, David K. Jackson, Ian Johnston, Dominic Kwiatkowski, Cordelia Langford, John Sillitoe on behalf of the Wellcome Sanger Institute COVID-19 Surveillance Team
EPI_ISL_633268, EPI_ISL_633269	Lighthouse Lab in Glasgow	Wellcome Sanger Institute for the COVID-19 Genomics UK (COG-UK) consortium	Harper VanSteenhouse, Yumi Kasai, David Gray, Carol Clugston, Anna Dominiczak and Alex Alderton, Roberto Amato, Sonia Goncalves, Ewan Harrison, David K. Jackson, Ian Johnston, Dominic Kwiatkowski, Cordelia Langford, John Sillitoe on behalf of the Wellcome Sanger Institute COVID-19 Surveillance Team
EPI_ISL_633270	Lighthouse Lab in Cambridge	Wellcome Sanger Institute for the COVID-19 Genomics UK (COG-UK) consortium	Rob Howes, The Lighthouse Lab in Cambridge and Alex Alderton, Roberto Amato, Sonia Goncalves, Ewan Harrison, David K. Jackson, Ian Johnston, Dominic Kwiatkowski, Cordelia Langford, John Sillitoe on behalf of the Wellcome Sanger Institute COVID-19 Surveillance Team
EPI_ISL_633271, EPI_ISL_633272	Lighthouse Lab in Glasgow	Wellcome Sanger Institute for the COVID-19 Genomics UK (COG-UK) consortium	Harper VanSteenhouse, Yumi Kasai, David Gray, Carol Clugston, Anna Dominiczak and Alex Alderton, Roberto Amato, Sonia Goncalves, Ewan Harrison, David K. Jackson, Ian Johnston, Dominic Kwiatkowski, Cordelia Langford, John Sillitoe on behalf of the Wellcome Sanger Institute COVID-19 Surveillance Team
EPI_ISL_633273	Lighthouse Lab in Cambridge	Wellcome Sanger Institute for the COVID-19 Genomics UK (COG-UK) consortium	Rob Howes, The Lighthouse Lab in Cambridge and Alex Alderton, Roberto Amato, Sonia Goncalves, Ewan Harrison, David K. Jackson, Ian Johnston, Dominic Kwiatkowski, Cordelia Langford, John Sillitoe on behalf of the Wellcome Sanger Institute COVID-19 Surveillance Team
EPI_ISL_633274	Lighthouse Lab in Glasgow	Wellcome Sanger Institute for the COVID-19 Genomics UK (COG-UK) consortium	Harper VanSteenhouse, Yumi Kasai, David Gray, Carol Clugston, Anna Dominiczak and Alex Alderton, Roberto Amato, Sonia Goncalves, Ewan Harrison, David K. Jackson, Ian Johnston, Dominic Kwiatkowski, Cordelia Langford, John Sillitoe on behalf of the Wellcome Sanger Institute COVID-19 Surveillance Team
EPI_ISL_633275	Lighthouse Lab in Cambridge	Wellcome Sanger Institute for the COVID-19 Genomics UK (COG-UK) consortium	Rob Howes, The Lighthouse Lab in Cambridge and Alex Alderton, Roberto Amato, Sonia Goncalves, Ewan Harrison, David K. Jackson, Ian Johnston, Dominic Kwiatkowski, Cordelia Langford, John Sillitoe on behalf of the Wellcome Sanger Institute COVID-19 Surveillance Team
EPI_ISL_633276	Lighthouse Lab in Glasgow	Wellcome Sanger Institute for the COVID-19 Genomics UK (COG-UK) consortium	Harper VanSteenhouse, Yumi Kasai, David Gray, Carol Clugston, Anna Dominiczak and Alex Alderton, Roberto Amato, Sonia Goncalves, Ewan Harrison, David K. Jackson, Ian Johnston, Dominic Kwiatkowski, Cordelia Langford, John Sillitoe on behalf of the Wellcome Sanger Institute COVID-19 Surveillance Team
EPI_ISL_633277, EPI_ISL_633278	Lighthouse Lab in Cambridge	Wellcome Sanger Institute for the COVID-19 Genomics UK (COG-UK) consortium	Rob Howes, The Lighthouse Lab in Cambridge and Alex Alderton, Roberto Amato, Sonia Goncalves, Ewan Harrison, David K. Jackson, Ian Johnston, Dominic Kwiatkowski, Cordelia Langford, John Sillitoe on behalf of the Wellcome Sanger Institute COVID-19 Surveillance Team
EPI_ISL_633279, EPI_ISL_633280	Lighthouse Lab in Glasgow	Wellcome Sanger Institute for the COVID-19 Genomics	Harper VanSteenhouse, Yumi Kasai, David Gray, Carol Clugston, Anna Dominiczak and Alex Alderton, Roberto Amato, Sonia Goncalves, Ewan Harrison,

		UK (COG-UK) consortium	David K. Jackson, Ian Johnston, Dominic Kwiatkowski, Cordelia Langford, John Sillitoe on behalf of the Wellcome Sanger Institute COVID-19 Surveillance Team
EPI_ISL_633281	Lighthouse Lab in Cambridge	Wellcome Sanger Institute for the COVID-19 Genomics UK (COG-UK) consortium	Rob Howes, The Lighthouse Lab in Cambridge and Alex Alderton, Roberto Amato, Sonia Goncalves, Ewan Harrison, David K. Jackson, Ian Johnston, Dominic Kwiatkowski, Cordelia Langford, John Sillitoe on behalf of the Wellcome Sanger Institute COVID-19 Surveillance Team
EPI_ISL_633283	Lighthouse Lab in Glasgow	Wellcome Sanger Institute for the COVID-19 Genomics UK (COG-UK) consortium	Harper VanSteenhouse, Yumi Kasai, David Gray, Carol Clugston, Anna Dominiczak and Alex Alderton, Roberto Amato, Sonia Goncalves, Ewan Harrison, David K. Jackson, Ian Johnston, Dominic Kwiatkowski, Cordelia Langford, John Sillitoe on behalf of the Wellcome Sanger Institute COVID-19 Surveillance Team
EPI_ISL_633284	Lighthouse Lab in Cambridge	Wellcome Sanger Institute for the COVID-19 Genomics UK (COG-UK) consortium	Rob Howes, The Lighthouse Lab in Cambridge and Alex Alderton, Roberto Amato, Sonia Goncalves, Ewan Harrison, David K. Jackson, Ian Johnston, Dominic Kwiatkowski, Cordelia Langford, John Sillitoe on behalf of the Wellcome Sanger Institute COVID-19 Surveillance Team
EPI_ISL_633285	Lighthouse Lab in Glasgow	Wellcome Sanger Institute for the COVID-19 Genomics UK (COG-UK) consortium	Harper VanSteenhouse, Yumi Kasai, David Gray, Carol Clugston, Anna Dominiczak and Alex Alderton, Roberto Amato, Sonia Goncalves, Ewan Harrison, David K. Jackson, Ian Johnston, Dominic Kwiatkowski, Cordelia Langford, John Sillitoe on behalf of the Wellcome Sanger Institute COVID-19 Surveillance Team
EPI_ISL_633286	Lighthouse Lab in Cambridge	Wellcome Sanger Institute for the COVID-19 Genomics UK (COG-UK) consortium	Rob Howes, The Lighthouse Lab in Cambridge and Alex Alderton, Roberto Amato, Sonia Goncalves, Ewan Harrison, David K. Jackson, Ian Johnston, Dominic Kwiatkowski, Cordelia Langford, John Sillitoe on behalf of the Wellcome Sanger Institute COVID-19 Surveillance Team
EPI_ISL_633287	Lighthouse Lab in Glasgow	Wellcome Sanger Institute for the COVID-19 Genomics UK (COG-UK) consortium	Harper VanSteenhouse, Yumi Kasai, David Gray, Carol Clugston, Anna Dominiczak and Alex Alderton, Roberto Amato, Sonia Goncalves, Ewan Harrison, David K. Jackson, Ian Johnston, Dominic Kwiatkowski, Cordelia Langford, John Sillitoe on behalf of the Wellcome Sanger Institute COVID-19 Surveillance Team
EPI_ISL_633288	Lighthouse Lab in Cambridge	Wellcome Sanger Institute for the COVID-19 Genomics UK (COG-UK) consortium	Rob Howes, The Lighthouse Lab in Cambridge and Alex Alderton, Roberto Amato, Sonia Goncalves, Ewan Harrison, David K. Jackson, Ian Johnston, Dominic Kwiatkowski, Cordelia Langford, John Sillitoe on behalf of the Wellcome Sanger Institute COVID-19 Surveillance Team
EPI_ISL_633289	Lighthouse Lab in Glasgow	Wellcome Sanger Institute for the COVID-19 Genomics UK (COG-UK) consortium	Harper VanSteenhouse, Yumi Kasai, David Gray, Carol Clugston, Anna Dominiczak and Alex Alderton, Roberto Amato, Sonia Goncalves, Ewan Harrison, David K. Jackson, Ian Johnston, Dominic Kwiatkowski, Cordelia Langford, John Sillitoe on behalf of the Wellcome Sanger Institute COVID-19 Surveillance Team
EPI_ISL_633291, EPI_ISL_633292	Lighthouse Lab in Cambridge	Wellcome Sanger Institute for the COVID-19 Genomics UK (COG-UK) consortium	Rob Howes, The Lighthouse Lab in Cambridge and Alex Alderton, Roberto Amato, Sonia Goncalves, Ewan Harrison, David K. Jackson, Ian Johnston, Dominic Kwiatkowski, Cordelia Langford, John Sillitoe on behalf of the Wellcome Sanger Institute COVID-19 Surveillance Team
EPI_ISL_633293	Lighthouse Lab in Glasgow	Wellcome Sanger Institute for the COVID-19 Genomics UK (COG-UK) consortium	Harper VanSteenhouse, Yumi Kasai, David Gray, Carol Clugston, Anna Dominiczak and Alex Alderton, Roberto Amato, Sonia Goncalves, Ewan Harrison, David K. Jackson, Ian Johnston, Dominic Kwiatkowski, Cordelia Langford, John Sillitoe on behalf of the Wellcome Sanger Institute COVID-19 Surveillance Team
EPI_ISL_633294, EPI_ISL_633295	Lighthouse Lab in Cambridge	Wellcome Sanger Institute for the COVID-19 Genomics UK (COG-UK) consortium	Rob Howes, The Lighthouse Lab in Cambridge and Alex Alderton, Roberto Amato, Sonia Goncalves, Ewan Harrison, David K. Jackson, Ian Johnston, Dominic Kwiatkowski, Cordelia Langford, John Sillitoe on behalf of the Wellcome Sanger Institute COVID-19 Surveillance Team
EPI_ISL_633296	Lighthouse Lab in Glasgow	Wellcome Sanger Institute for the COVID-19 Genomics UK (COG-UK) consortium	Harper VanSteenhouse, Yumi Kasai, David Gray, Carol Clugston, Anna Dominiczak and Alex Alderton, Roberto Amato, Sonia Goncalves, Ewan Harrison, David K. Jackson, Ian Johnston, Dominic Kwiatkowski, Cordelia Langford, John Sillitoe on behalf of the Wellcome Sanger Institute COVID-19 Surveillance Team
EPI_ISL_633298, EPI_ISL_633299, EPI_ISL_633300	Lighthouse Lab in Cambridge	Wellcome Sanger Institute for the COVID-19 Genomics UK (COG-UK) consortium	Rob Howes, The Lighthouse Lab in Cambridge and Alex Alderton, Roberto Amato, Sonia Goncalves, Ewan Harrison, David K. Jackson, Ian Johnston, Dominic Kwiatkowski, Cordelia Langford, John Sillitoe on behalf of the Wellcome Sanger Institute COVID-19 Surveillance Team
EPI_ISL_633301	Lighthouse Lab in Glasgow	Wellcome Sanger Institute for the COVID-19 Genomics UK (COG-UK) consortium	Harper VanSteenhouse, Yumi Kasai, David Gray, Carol Clugston, Anna Dominiczak and Alex Alderton, Roberto Amato, Sonia Goncalves, Ewan Harrison, David K. Jackson, Ian Johnston, Dominic Kwiatkowski, Cordelia Langford, John Sillitoe on behalf of the Wellcome Sanger Institute COVID-19 Surveillance Team
EPI_ISL_633302	Lighthouse Lab in Cambridge	Wellcome Sanger Institute for the COVID-19 Genomics UK (COG-UK) consortium	Rob Howes, The Lighthouse Lab in Cambridge and Alex Alderton, Roberto Amato, Sonia Goncalves, Ewan Harrison, David K. Jackson, Ian Johnston, Dominic Kwiatkowski, Cordelia Langford, John Sillitoe on behalf of the Wellcome Sanger Institute COVID-19 Surveillance Team
EPI_ISL_633303, EPI_ISL_633304, EPI_ISL_633305, EPI_ISL_633306, EPI_ISL_633307	Lighthouse Lab in Glasgow	Wellcome Sanger Institute for the COVID-19 Genomics UK (COG-UK) consortium	Harper VanSteenhouse, Yumi Kasai, David Gray, Carol Clugston, Anna Dominiczak and Alex Alderton, Roberto Amato, Sonia Goncalves, Ewan Harrison, David K. Jackson, Ian Johnston, Dominic Kwiatkowski, Cordelia Langford, John Sillitoe on behalf of the Wellcome Sanger Institute COVID-19 Surveillance Team
EPI_ISL_633308	Lighthouse Lab in Cambridge	Wellcome Sanger Institute for the COVID-19 Genomics UK (COG-UK) consortium	Rob Howes, The Lighthouse Lab in Cambridge and Alex Alderton, Roberto Amato, Sonia Goncalves, Ewan Harrison, David K. Jackson, Ian Johnston, Dominic Kwiatkowski, Cordelia Langford, John Sillitoe on behalf of the Wellcome Sanger Institute COVID-19 Surveillance Team
EPI_ISL_633309, EPI_ISL_633310	Lighthouse Lab in Glasgow	Wellcome Sanger Institute for the COVID-19 Genomics UK (COG-UK) consortium	Harper VanSteenhouse, Yumi Kasai, David Gray, Carol Clugston, Anna Dominiczak and Alex Alderton, Roberto Amato, Sonia Goncalves, Ewan Harrison, David K. Jackson, Ian Johnston, Dominic Kwiatkowski, Cordelia Langford, John Sillitoe on behalf of the Wellcome Sanger Institute COVID-19 Surveillance Team
EPI_ISL_633311	Lighthouse Lab in Cambridge	Wellcome Sanger Institute for the COVID-19 Genomics UK (COG-UK) consortium	Rob Howes, The Lighthouse Lab in Cambridge and Alex Alderton, Roberto Amato, Sonia Goncalves, Ewan Harrison, David K. Jackson, Ian Johnston, Dominic Kwiatkowski, Cordelia Langford, John Sillitoe on behalf of the Wellcome Sanger Institute COVID-19 Surveillance Team
EPI_ISL_633312	Lighthouse Lab in Glasgow	Wellcome Sanger Institute for the COVID-19 Genomics UK (COG-UK) consortium	Harper VanSteenhouse, Yumi Kasai, David Gray, Carol Clugston, Anna Dominiczak and Alex Alderton, Roberto Amato, Sonia Goncalves, Ewan Harrison, David K. Jackson, Ian Johnston, Dominic Kwiatkowski, Cordelia Langford, John Sillitoe on behalf of the Wellcome Sanger Institute COVID-19 Surveillance Team
EPI_ISL_633313, EPI_ISL_633314, EPI_ISL_633315	Lighthouse Lab in Cambridge	Wellcome Sanger Institute for the COVID-19 Genomics UK (COG-UK) consortium	Rob Howes, The Lighthouse Lab in Cambridge and Alex Alderton, Roberto Amato, Sonia Goncalves, Ewan Harrison, David K. Jackson, Ian Johnston, Dominic Kwiatkowski, Cordelia Langford, John Sillitoe on behalf of the Wellcome Sanger Institute COVID-19 Surveillance Team
EPI_ISL_633316, EPI_ISL_633317, EPI_ISL_633318	Lighthouse Lab in Glasgow	Wellcome Sanger Institute for the COVID-19 Genomics UK (COG-UK) consortium	Harper VanSteenhouse, Yumi Kasai, David Gray, Carol Clugston, Anna Dominiczak and Alex Alderton, Roberto Amato, Sonia Goncalves, Ewan Harrison, David K. Jackson, Ian Johnston, Dominic Kwiatkowski, Cordelia Langford, John Sillitoe on behalf of the Wellcome Sanger Institute COVID-19 Surveillance Team
EPI_ISL_633319	Lighthouse Lab in Cambridge	Wellcome Sanger Institute for the COVID-19 Genomics UK (COG-UK) consortium	Rob Howes, The Lighthouse Lab in Cambridge and Alex Alderton, Roberto Amato, Sonia Goncalves, Ewan Harrison, David K. Jackson, Ian Johnston, Dominic Kwiatkowski, Cordelia Langford, John Sillitoe on behalf of the Wellcome Sanger Institute COVID-19 Surveillance Team
EPI_ISL_633320	Lighthouse Lab in Glasgow	Wellcome Sanger Institute for the COVID-19 Genomics UK (COG-UK) consortium	Harper VanSteenhouse, Yumi Kasai, David Gray, Carol Clugston, Anna Dominiczak and Alex Alderton, Roberto Amato, Sonia Goncalves, Ewan Harrison, David K. Jackson, Ian Johnston, Dominic Kwiatkowski, Cordelia Langford, John Sillitoe on behalf of the Wellcome Sanger Institute COVID-19 Surveillance Team
EPI_ISL_633321, EPI_ISL_633322	Lighthouse Lab in Cambridge	Wellcome Sanger Institute for the COVID-19 Genomics UK (COG-UK) consortium	Rob Howes, The Lighthouse Lab in Cambridge and Alex Alderton, Roberto Amato, Sonia Goncalves, Ewan Harrison, David K. Jackson, Ian Johnston, Dominic Kwiatkowski, Cordelia Langford, John Sillitoe on behalf of the Wellcome Sanger Institute COVID-19 Surveillance Team
EPI_ISL_633323	Lighthouse Lab in Glasgow	Wellcome Sanger Institute for the COVID-19 Genomics UK (COG-UK) consortium	Harper VanSteenhouse, Yumi Kasai, David Gray, Carol Clugston, Anna Dominiczak and Alex Alderton, Roberto Amato, Sonia Goncalves, Ewan Harrison, David K. Jackson, Ian Johnston, Dominic Kwiatkowski, Cordelia Langford, John Sillitoe on behalf of the Wellcome Sanger Institute COVID-19 Surveillance Team
EPI_ISL_633324, EPI_ISL_633325	Lighthouse Lab in Cambridge	Wellcome Sanger Institute for the COVID-19 Genomics UK (COG-UK) consortium	Rob Howes, The Lighthouse Lab in Cambridge and Alex Alderton, Roberto Amato, Sonia Goncalves, Ewan Harrison, David K. Jackson, Ian Johnston, Dominic Kwiatkowski, Cordelia Langford, John Sillitoe on behalf of the Wellcome Sanger Institute COVID-19 Surveillance Team
EPI_ISL_633326, EPI_ISL_633327, EPI_ISL_633328	Lighthouse Lab in Glasgow	Wellcome Sanger Institute for the COVID-19 Genomics UK (COG-UK) consortium	Harper VanSteenhouse, Yumi Kasai, David Gray, Carol Clugston, Anna Dominiczak and Alex Alderton, Roberto Amato, Sonia Goncalves, Ewan Harrison, David K. Jackson, Ian Johnston, Dominic Kwiatkowski, Cordelia Langford, John Sillitoe on behalf of the Wellcome Sanger Institute COVID-19 Surveillance Team
EPI_ISL_633329, EPI_ISL_633330	Lighthouse Lab in Cambridge	Wellcome Sanger Institute for the COVID-19 Genomics	Rob Howes, The Lighthouse Lab in Cambridge and Alex Alderton, Roberto Amato, Sonia Goncalves, Ewan Harrison, David K. Jackson, Ian Johnston,

		UK (COG-UK) consortium	Dominic Kwiatkowski, Cordelia Langford, John Sillitoe on behalf of the Wellcome Sanger Institute COVID-19 Surveillance Team
EPI_ISL_633332	Lighthouse Lab in Glasgow	Wellcome Sanger Institute for the COVID-19 Genomics UK (COG-UK) consortium	Harper VanSteenhouse, Yumi Kasai, David Gray, Carol Clugston, Anna Dominiczak and Alex Alderton, Roberto Amato, Sonia Goncalves, Ewan Harrison, David K. Jackson, Ian Johnston, Dominic Kwiatkowski, Cordelia Langford, John Sillitoe on behalf of the Wellcome Sanger Institute COVID-19 Surveillance Team
EPI_ISL_633333, EPI_ISL_633334	Lighthouse Lab in Cambridge	Wellcome Sanger Institute for the COVID-19 Genomics UK (COG-UK) consortium	Rob Howes, The Lighthouse Lab in Cambridge and Alex Alderton, Roberto Amato, Sonia Goncalves, Ewan Harrison, David K. Jackson, Ian Johnston, Dominic Kwiatkowski, Cordelia Langford, John Sillitoe on behalf of the Wellcome Sanger Institute COVID-19 Surveillance Team
EPI_ISL_633335	Lighthouse Lab in Glasgow	Wellcome Sanger Institute for the COVID-19 Genomics UK (COG-UK) consortium	Harper VanSteenhouse, Yumi Kasai, David Gray, Carol Clugston, Anna Dominiczak and Alex Alderton, Roberto Amato, Sonia Goncalves, Ewan Harrison, David K. Jackson, Ian Johnston, Dominic Kwiatkowski, Cordelia Langford, John Sillitoe on behalf of the Wellcome Sanger Institute COVID-19 Surveillance Team
EPI_ISL_633336, EPI_ISL_633337	Lighthouse Lab in Cambridge	Wellcome Sanger Institute for the COVID-19 Genomics UK (COG-UK) consortium	Rob Howes, The Lighthouse Lab in Cambridge and Alex Alderton, Roberto Amato, Sonia Goncalves, Ewan Harrison, David K. Jackson, Ian Johnston, Dominic Kwiatkowski, Cordelia Langford, John Sillitoe on behalf of the Wellcome Sanger Institute COVID-19 Surveillance Team
EPI_ISL_633338, EPI_ISL_633339	Lighthouse Lab in Glasgow	Wellcome Sanger Institute for the COVID-19 Genomics UK (COG-UK) consortium	Harper VanSteenhouse, Yumi Kasai, David Gray, Carol Clugston, Anna Dominiczak and Alex Alderton, Roberto Amato, Sonia Goncalves, Ewan Harrison, David K. Jackson, Ian Johnston, Dominic Kwiatkowski, Cordelia Langford, John Sillitoe on behalf of the Wellcome Sanger Institute COVID-19 Surveillance Team
EPI_ISL_633340	Lighthouse Lab in Cambridge	Wellcome Sanger Institute for the COVID-19 Genomics UK (COG-UK) consortium	Rob Howes, The Lighthouse Lab in Cambridge and Alex Alderton, Roberto Amato, Sonia Goncalves, Ewan Harrison, David K. Jackson, Ian Johnston, Dominic Kwiatkowski, Cordelia Langford, John Sillitoe on behalf of the Wellcome Sanger Institute COVID-19 Surveillance Team
EPI_ISL_633342, EPI_ISL_633343	Lighthouse Lab in Glasgow	Wellcome Sanger Institute for the COVID-19 Genomics UK (COG-UK) consortium	Harper VanSteenhouse, Yumi Kasai, David Gray, Carol Clugston, Anna Dominiczak and Alex Alderton, Roberto Amato, Sonia Goncalves, Ewan Harrison, David K. Jackson, Ian Johnston, Dominic Kwiatkowski, Cordelia Langford, John Sillitoe on behalf of the Wellcome Sanger Institute COVID-19 Surveillance Team
EPI_ISL_633344, EPI_ISL_633345, EPI_ISL_633346, EPI_ISL_633347	Lighthouse Lab in Cambridge	Wellcome Sanger Institute for the COVID-19 Genomics UK (COG-UK) consortium	Rob Howes, The Lighthouse Lab in Cambridge and Alex Alderton, Roberto Amato, Sonia Goncalves, Ewan Harrison, David K. Jackson, Ian Johnston, Dominic Kwiatkowski, Cordelia Langford, John Sillitoe on behalf of the Wellcome Sanger Institute COVID-19 Surveillance Team
EPI_ISL_633348	Lighthouse Lab in Glasgow	Wellcome Sanger Institute for the COVID-19 Genomics UK (COG-UK) consortium	Harper VanSteenhouse, Yumi Kasai, David Gray, Carol Clugston, Anna Dominiczak and Alex Alderton, Roberto Amato, Sonia Goncalves, Ewan Harrison, David K. Jackson, Ian Johnston, Dominic Kwiatkowski, Cordelia Langford, John Sillitoe on behalf of the Wellcome Sanger Institute COVID-19 Surveillance Team
EPI_ISL_633349	Lighthouse Lab in Cambridge	Wellcome Sanger Institute for the COVID-19 Genomics UK (COG-UK) consortium	Rob Howes, The Lighthouse Lab in Cambridge and Alex Alderton, Roberto Amato, Sonia Goncalves, Ewan Harrison, David K. Jackson, Ian Johnston, Dominic Kwiatkowski, Cordelia Langford, John Sillitoe on behalf of the Wellcome Sanger Institute COVID-19 Surveillance Team
EPI_ISL_633351, EPI_ISL_633352, EPI_ISL_633353, EPI_ISL_633354, EPI_ISL_633355	Lighthouse Lab in Glasgow	Wellcome Sanger Institute for the COVID-19 Genomics UK (COG-UK) consortium	Harper VanSteenhouse, Yumi Kasai, David Gray, Carol Clugston, Anna Dominiczak and Alex Alderton, Roberto Amato, Sonia Goncalves, Ewan Harrison, David K. Jackson, Ian Johnston, Dominic Kwiatkowski, Cordelia Langford, John Sillitoe on behalf of the Wellcome Sanger Institute COVID-19 Surveillance Team
EPI_ISL_633356, EPI_ISL_633357	Lighthouse Lab in Cambridge	Wellcome Sanger Institute for the COVID-19 Genomics UK (COG-UK) consortium	Rob Howes, The Lighthouse Lab in Cambridge and Alex Alderton, Roberto Amato, Sonia Goncalves, Ewan Harrison, David K. Jackson, Ian Johnston, Dominic Kwiatkowski, Cordelia Langford, John Sillitoe on behalf of the Wellcome Sanger Institute COVID-19 Surveillance Team
EPI_ISL_633358	Lighthouse Lab in Glasgow	Wellcome Sanger Institute for the COVID-19 Genomics UK (COG-UK) consortium	Harper VanSteenhouse, Yumi Kasai, David Gray, Carol Clugston, Anna Dominiczak and Alex Alderton, Roberto Amato, Sonia Goncalves, Ewan Harrison, David K. Jackson, Ian Johnston, Dominic Kwiatkowski, Cordelia Langford, John Sillitoe on behalf of the Wellcome Sanger Institute COVID-19 Surveillance Team
EPI_ISL_633359, EPI_ISL_633360	Lighthouse Lab in Cambridge	Wellcome Sanger Institute for the COVID-19 Genomics UK (COG-UK) consortium	Rob Howes, The Lighthouse Lab in Cambridge and Alex Alderton, Roberto Amato, Sonia Goncalves, Ewan Harrison, David K. Jackson, Ian Johnston, Dominic Kwiatkowski, Cordelia Langford, John Sillitoe on behalf of the Wellcome Sanger Institute COVID-19 Surveillance Team
EPI_ISL_633362	Lighthouse Lab in Glasgow	Wellcome Sanger Institute for the COVID-19 Genomics UK (COG-UK) consortium	Harper VanSteenhouse, Yumi Kasai, David Gray, Carol Clugston, Anna Dominiczak and Alex Alderton, Roberto Amato, Sonia Goncalves, Ewan Harrison, David K. Jackson, Ian Johnston, Dominic Kwiatkowski, Cordelia Langford, John Sillitoe on behalf of the Wellcome Sanger Institute COVID-19 Surveillance Team
EPI_ISL_633363	Lighthouse Lab in Cambridge	Wellcome Sanger Institute for the COVID-19 Genomics UK (COG-UK) consortium	Rob Howes, The Lighthouse Lab in Cambridge and Alex Alderton, Roberto Amato, Sonia Goncalves, Ewan Harrison, David K. Jackson, Ian Johnston, Dominic Kwiatkowski, Cordelia Langford, John Sillitoe on behalf of the Wellcome Sanger Institute COVID-19 Surveillance Team
EPI_ISL_633364	Lighthouse Lab in Glasgow	Wellcome Sanger Institute for the COVID-19 Genomics UK (COG-UK) consortium	Harper VanSteenhouse, Yumi Kasai, David Gray, Carol Clugston, Anna Dominiczak and Alex Alderton, Roberto Amato, Sonia Goncalves, Ewan Harrison, David K. Jackson, Ian Johnston, Dominic Kwiatkowski, Cordelia Langford, John Sillitoe on behalf of the Wellcome Sanger Institute COVID-19 Surveillance Team
EPI_ISL_633365, EPI_ISL_633366	Lighthouse Lab in Cambridge	Wellcome Sanger Institute for the COVID-19 Genomics UK (COG-UK) consortium	Rob Howes, The Lighthouse Lab in Cambridge and Alex Alderton, Roberto Amato, Sonia Goncalves, Ewan Harrison, David K. Jackson, Ian Johnston, Dominic Kwiatkowski, Cordelia Langford, John Sillitoe on behalf of the Wellcome Sanger Institute COVID-19 Surveillance Team
EPI_ISL_633367	Lighthouse Lab in Glasgow	Wellcome Sanger Institute for the COVID-19 Genomics UK (COG-UK) consortium	Harper VanSteenhouse, Yumi Kasai, David Gray, Carol Clugston, Anna Dominiczak and Alex Alderton, Roberto Amato, Sonia Goncalves, Ewan Harrison, David K. Jackson, Ian Johnston, Dominic Kwiatkowski, Cordelia Langford, John Sillitoe on behalf of the Wellcome Sanger Institute COVID-19 Surveillance Team
EPI_ISL_633369, EPI_ISL_633370	Lighthouse Lab in Cambridge	Wellcome Sanger Institute for the COVID-19 Genomics UK (COG-UK) consortium	Rob Howes, The Lighthouse Lab in Cambridge and Alex Alderton, Roberto Amato, Sonia Goncalves, Ewan Harrison, David K. Jackson, Ian Johnston, Dominic Kwiatkowski, Cordelia Langford, John Sillitoe on behalf of the Wellcome Sanger Institute COVID-19 Surveillance Team
EPI_ISL_633371	Lighthouse Lab in Glasgow	Wellcome Sanger Institute for the COVID-19 Genomics UK (COG-UK) consortium	Harper VanSteenhouse, Yumi Kasai, David Gray, Carol Clugston, Anna Dominiczak and Alex Alderton, Roberto Amato, Sonia Goncalves, Ewan Harrison, David K. Jackson, Ian Johnston, Dominic Kwiatkowski, Cordelia Langford, John Sillitoe on behalf of the Wellcome Sanger Institute COVID-19 Surveillance Team
EPI_ISL_633372, EPI_ISL_633373, EPI_ISL_633374, EPI_ISL_633375	Lighthouse Lab in Cambridge	Wellcome Sanger Institute for the COVID-19 Genomics UK (COG-UK) consortium	Rob Howes, The Lighthouse Lab in Cambridge and Alex Alderton, Roberto Amato, Sonia Goncalves, Ewan Harrison, David K. Jackson, Ian Johnston, Dominic Kwiatkowski, Cordelia Langford, John Sillitoe on behalf of the Wellcome Sanger Institute COVID-19 Surveillance Team
EPI_ISL_633376, EPI_ISL_633377	Lighthouse Lab in Glasgow	Wellcome Sanger Institute for the COVID-19 Genomics UK (COG-UK) consortium	Harper VanSteenhouse, Yumi Kasai, David Gray, Carol Clugston, Anna Dominiczak and Alex Alderton, Roberto Amato, Sonia Goncalves, Ewan Harrison, David K. Jackson, Ian Johnston, Dominic Kwiatkowski, Cordelia Langford, John Sillitoe on behalf of the Wellcome Sanger Institute COVID-19 Surveillance Team
EPI_ISL_633378	Lighthouse Lab in Cambridge	Wellcome Sanger Institute for the COVID-19 Genomics UK (COG-UK) consortium	Rob Howes, The Lighthouse Lab in Cambridge and Alex Alderton, Roberto Amato, Sonia Goncalves, Ewan Harrison, David K. Jackson, Ian Johnston, Dominic Kwiatkowski, Cordelia Langford, John Sillitoe on behalf of the Wellcome Sanger Institute COVID-19 Surveillance Team
EPI_ISL_633379	Lighthouse Lab in Glasgow	Wellcome Sanger Institute for the COVID-19 Genomics UK (COG-UK) consortium	Harper VanSteenhouse, Yumi Kasai, David Gray, Carol Clugston, Anna Dominiczak and Alex Alderton, Roberto Amato, Sonia Goncalves, Ewan Harrison, David K. Jackson, Ian Johnston, Dominic Kwiatkowski, Cordelia Langford, John Sillitoe on behalf of the Wellcome Sanger Institute COVID-19 Surveillance Team
EPI_ISL_633380	Lighthouse Lab in Cambridge	Wellcome Sanger Institute for the COVID-19 Genomics UK (COG-UK) consortium	Rob Howes, The Lighthouse Lab in Cambridge and Alex Alderton, Roberto Amato, Sonia Goncalves, Ewan Harrison, David K. Jackson, Ian Johnston, Dominic Kwiatkowski, Cordelia Langford, John Sillitoe on behalf of the Wellcome Sanger Institute COVID-19 Surveillance Team
EPI_ISL_633381, EPI_ISL_633382	Lighthouse Lab in Glasgow	Wellcome Sanger Institute for the COVID-19 Genomics UK (COG-UK) consortium	Harper VanSteenhouse, Yumi Kasai, David Gray, Carol Clugston, Anna Dominiczak and Alex Alderton, Roberto Amato, Sonia Goncalves, Ewan Harrison, David K. Jackson, Ian Johnston, Dominic Kwiatkowski, Cordelia Langford, John Sillitoe on behalf of the Wellcome Sanger Institute COVID-19 Surveillance Team
EPI_ISL_633383	Lighthouse Lab in Cambridge	Wellcome Sanger Institute for the COVID-19 Genomics UK (COG-UK) consortium	Rob Howes, The Lighthouse Lab in Cambridge and Alex Alderton, Roberto Amato, Sonia Goncalves, Ewan Harrison, David K. Jackson, Ian Johnston, Dominic Kwiatkowski, Cordelia Langford, John Sillitoe on behalf of the Wellcome Sanger Institute COVID-19 Surveillance Team
EPI_ISL_633384	Lighthouse Lab in Glasgow	Wellcome Sanger Institute for the COVID-19 Genomics UK (COG-UK) consortium	Harper VanSteenhouse, Yumi Kasai, David Gray, Carol Clugston, Anna Dominiczak and Alex Alderton, Roberto Amato, Sonia Goncalves, Ewan Harrison, David K. Jackson, Ian Johnston, Dominic Kwiatkowski, Cordelia Langford, John Sillitoe on behalf of the Wellcome Sanger Institute COVID-19 Surveillance

			Team
EPI_ISL_633385	Lighthouse Lab in Cambridge	Wellcome Sanger Institute for the COVID-19 Genomics UK (COG-UK) consortium	Rob Howes, The Lighthouse Lab in Cambridge and Alex Alderton, Roberto Amato, Sonia Goncalves, Ewan Harrison, David K. Jackson, Ian Johnston, Dominic Kwiatkowski, Cordelia Langford, John Sillitoe on behalf of the Wellcome Sanger Institute COVID-19 Surveillance Team
EPI_ISL_633386, EPI_ISL_633387	Lighthouse Lab in Glasgow	Wellcome Sanger Institute for the COVID-19 Genomics UK (COG-UK) consortium	Harper VanSteenhouse, Yumi Kasai, David Gray, Carol Clugston, Anna Dominiczak and Alex Alderton, Roberto Amato, Sonia Goncalves, Ewan Harrison, David K. Jackson, Ian Johnston, Dominic Kwiatkowski, Cordelia Langford, John Sillitoe on behalf of the Wellcome Sanger Institute COVID-19 Surveillance Team
EPI_ISL_633388, EPI_ISL_633389, EPI_ISL_633390	Lighthouse Lab in Cambridge	Wellcome Sanger Institute for the COVID-19 Genomics UK (COG-UK) consortium	Rob Howes, The Lighthouse Lab in Cambridge and Alex Alderton, Roberto Amato, Sonia Goncalves, Ewan Harrison, David K. Jackson, Ian Johnston, Dominic Kwiatkowski, Cordelia Langford, John Sillitoe on behalf of the Wellcome Sanger Institute COVID-19 Surveillance Team
EPI_ISL_633391, EPI_ISL_633392	Lighthouse Lab in Glasgow	Wellcome Sanger Institute for the COVID-19 Genomics UK (COG-UK) consortium	Harper VanSteenhouse, Yumi Kasai, David Gray, Carol Clugston, Anna Dominiczak and Alex Alderton, Roberto Amato, Sonia Goncalves, Ewan Harrison, David K. Jackson, Ian Johnston, Dominic Kwiatkowski, Cordelia Langford, John Sillitoe on behalf of the Wellcome Sanger Institute COVID-19 Surveillance Team
EPI_ISL_633393	Lighthouse Lab in Cambridge	Wellcome Sanger Institute for the COVID-19 Genomics UK (COG-UK) consortium	Rob Howes, The Lighthouse Lab in Cambridge and Alex Alderton, Roberto Amato, Sonia Goncalves, Ewan Harrison, David K. Jackson, Ian Johnston, Dominic Kwiatkowski, Cordelia Langford, John Sillitoe on behalf of the Wellcome Sanger Institute COVID-19 Surveillance Team
EPI_ISL_633394	Lighthouse Lab in Glasgow	Wellcome Sanger Institute for the COVID-19 Genomics UK (COG-UK) consortium	Harper VanSteenhouse, Yumi Kasai, David Gray, Carol Clugston, Anna Dominiczak and Alex Alderton, Roberto Amato, Sonia Goncalves, Ewan Harrison, David K. Jackson, Ian Johnston, Dominic Kwiatkowski, Cordelia Langford, John Sillitoe on behalf of the Wellcome Sanger Institute COVID-19 Surveillance Team
EPI_ISL_633395	Lighthouse Lab in Cambridge	Wellcome Sanger Institute for the COVID-19 Genomics UK (COG-UK) consortium	Rob Howes, The Lighthouse Lab in Cambridge and Alex Alderton, Roberto Amato, Sonia Goncalves, Ewan Harrison, David K. Jackson, Ian Johnston, Dominic Kwiatkowski, Cordelia Langford, John Sillitoe on behalf of the Wellcome Sanger Institute COVID-19 Surveillance Team
EPI_ISL_633396	Lighthouse Lab in Glasgow	Wellcome Sanger Institute for the COVID-19 Genomics UK (COG-UK) consortium	Harper VanSteenhouse, Yumi Kasai, David Gray, Carol Clugston, Anna Dominiczak and Alex Alderton, Roberto Amato, Sonia Goncalves, Ewan Harrison, David K. Jackson, Ian Johnston, Dominic Kwiatkowski, Cordelia Langford, John Sillitoe on behalf of the Wellcome Sanger Institute COVID-19 Surveillance Team
EPI_ISL_633397, EPI_ISL_633398, EPI_ISL_633399	Lighthouse Lab in Cambridge	Wellcome Sanger Institute for the COVID-19 Genomics UK (COG-UK) consortium	Rob Howes, The Lighthouse Lab in Cambridge and Alex Alderton, Roberto Amato, Sonia Goncalves, Ewan Harrison, David K. Jackson, Ian Johnston, Dominic Kwiatkowski, Cordelia Langford, John Sillitoe on behalf of the Wellcome Sanger Institute COVID-19 Surveillance Team
EPI_ISL_633400, EPI_ISL_633401, EPI_ISL_633402	Lighthouse Lab in Glasgow	Wellcome Sanger Institute for the COVID-19 Genomics UK (COG-UK) consortium	Harper VanSteenhouse, Yumi Kasai, David Gray, Carol Clugston, Anna Dominiczak and Alex Alderton, Roberto Amato, Sonia Goncalves, Ewan Harrison, David K. Jackson, Ian Johnston, Dominic Kwiatkowski, Cordelia Langford, John Sillitoe on behalf of the Wellcome Sanger Institute COVID-19 Surveillance Team
EPI_ISL_633403	Lighthouse Lab in Cambridge	Wellcome Sanger Institute for the COVID-19 Genomics UK (COG-UK) consortium	Rob Howes, The Lighthouse Lab in Cambridge and Alex Alderton, Roberto Amato, Sonia Goncalves, Ewan Harrison, David K. Jackson, Ian Johnston, Dominic Kwiatkowski, Cordelia Langford, John Sillitoe on behalf of the Wellcome Sanger Institute COVID-19 Surveillance Team
EPI_ISL_633404	Lighthouse Lab in Glasgow	Wellcome Sanger Institute for the COVID-19 Genomics UK (COG-UK) consortium	Harper VanSteenhouse, Yumi Kasai, David Gray, Carol Clugston, Anna Dominiczak and Alex Alderton, Roberto Amato, Sonia Goncalves, Ewan Harrison, David K. Jackson, Ian Johnston, Dominic Kwiatkowski, Cordelia Langford, John Sillitoe on behalf of the Wellcome Sanger Institute COVID-19 Surveillance Team
EPI_ISL_633405	Lighthouse Lab in Cambridge	Wellcome Sanger Institute for the COVID-19 Genomics UK (COG-UK) consortium	Rob Howes, The Lighthouse Lab in Cambridge and Alex Alderton, Roberto Amato, Sonia Goncalves, Ewan Harrison, David K. Jackson, Ian Johnston, Dominic Kwiatkowski, Cordelia Langford, John Sillitoe on behalf of the Wellcome Sanger Institute COVID-19 Surveillance Team
EPI_ISL_633406	Lighthouse Lab in Glasgow	Wellcome Sanger Institute for the COVID-19 Genomics UK (COG-UK) consortium	Harper VanSteenhouse, Yumi Kasai, David Gray, Carol Clugston, Anna Dominiczak and Alex Alderton, Roberto Amato, Sonia Goncalves, Ewan Harrison, David K. Jackson, Ian Johnston, Dominic Kwiatkowski, Cordelia Langford, John Sillitoe on behalf of the Wellcome Sanger Institute COVID-19 Surveillance Team
EPI_ISL_633407, EPI_ISL_633408	Lighthouse Lab in Cambridge	Wellcome Sanger Institute for the COVID-19 Genomics UK (COG-UK) consortium	Rob Howes, The Lighthouse Lab in Cambridge and Alex Alderton, Roberto Amato, Sonia Goncalves, Ewan Harrison, David K. Jackson, Ian Johnston, Dominic Kwiatkowski, Cordelia Langford, John Sillitoe on behalf of the Wellcome Sanger Institute COVID-19 Surveillance Team
EPI_ISL_633409, EPI_ISL_633410, EPI_ISL_633411, EPI_ISL_633412, EPI_ISL_633413, EPI_ISL_633414, EPI_ISL_633415, EPI_ISL_633416, EPI_ISL_633417, EPI_ISL_633418, EPI_ISL_633419, EPI_ISL_633420, EPI_ISL_633422, EPI_ISL_633423, EPI_ISL_633424, EPI_ISL_633425, EPI_ISL_633426, EPI_ISL_633427, EPI_ISL_633428, EPI_ISL_633429, EPI_ISL_633430, EPI_ISL_633431, EPI_ISL_633433, EPI_ISL_633434, EPI_ISL_633435, EPI_ISL_633436, EPI_ISL_633437, EPI_ISL_633438, EPI_ISL_633439, EPI_ISL_633440, EPI_ISL_633441, EPI_ISL_633442, EPI_ISL_633444, EPI_ISL_633445, EPI_ISL_633446, EPI_ISL_633447, EPI_ISL_633448, EPI_ISL_633449, EPI_ISL_633450, EPI_ISL_633451, EPI_ISL_633452, EPI_ISL_633453, EPI_ISL_633454, EPI_ISL_633455, EPI_ISL_633456, EPI_ISL_633457, EPI_ISL_633458, EPI_ISL_633459, EPI_ISL_633460, EPI_ISL_633461, EPI_ISL_633462, EPI_ISL_633464, EPI_ISL_633466, EPI_ISL_633467, EPI_ISL_633468, EPI_ISL_633469, EPI_ISL_633470, EPI_ISL_633471, EPI_ISL_633472, EPI_ISL_633473, EPI_ISL_633475, EPI_ISL_633476, EPI_ISL_633477, EPI_ISL_633478, EPI_ISL_633479, EPI_ISL_633480, EPI_ISL_633481, EPI_ISL_633482, EPI_ISL_633483, EPI_ISL_633484, EPI_ISL_633485, EPI_ISL_633486, EPI_ISL_633487, EPI_ISL_633488, EPI_ISL_633490, EPI_ISL_633491, EPI_ISL_633493, EPI_ISL_633494, EPI_ISL_633495, EPI_ISL_633496, EPI_ISL_633497, EPI_ISL_633499, EPI_ISL_633500, EPI_ISL_633501, EPI_ISL_633502, EPI_ISL_633503, EPI_ISL_633504, EPI_ISL_633505, EPI_ISL_633506, EPI_ISL_633507, EPI_ISL_633508, EPI_ISL_633509, EPI_ISL_633510, EPI_ISL_633511, EPI_ISL_633512, EPI_ISL_633513, EPI_ISL_633514, EPI_ISL_633515, EPI_ISL_633516, EPI_ISL_633517, EPI_ISL_633518, EPI_ISL_633519, EPI_ISL_633520, EPI_ISL_633521, EPI_ISL_633522, EPI_ISL_633523, EPI_ISL_633524, EPI_ISL_633525, EPI_ISL_633526, EPI_ISL_633527, EPI_ISL_633528, EPI_ISL_633529, EPI_ISL_633530, EPI_ISL_633531, EPI_ISL_633532, EPI_ISL_633533, EPI_ISL_633534, EPI_ISL_633535, EPI_ISL_633536, EPI_ISL_633537, EPI_ISL_633538, EPI_ISL_633539, EPI_ISL_633541, EPI_ISL_633542, EPI_ISL_633543, EPI_ISL_633544, EPI_ISL_633545, EPI_ISL_633546, EPI_ISL_633547, EPI_ISL_633548, EPI_ISL_633549, EPI_ISL_633550, EPI_ISL_633551, EPI_ISL_633552, EPI_ISL_633553, EPI_ISL_633554, EPI_ISL_633555, EPI_ISL_633556, EPI_ISL_633557, EPI_ISL_633558, EPI_ISL_633559, EPI_ISL_633560, EPI_ISL_633561, EPI_ISL_633562, EPI_ISL_633563, EPI_ISL_633564, EPI_ISL_633565, EPI_ISL_633566, EPI_ISL_633570, EPI_ISL_633571, EPI_ISL_633572, EPI_ISL_633573, EPI_ISL_633574, EPI_ISL_633575, EPI_ISL_633576, EPI_ISL_633578, EPI_ISL_633579, EPI_ISL_633580, EPI_ISL_633581, EPI_ISL_633582, EPI_ISL_633583, EPI_ISL_633584, EPI_ISL_633586, EPI_ISL_633587, EPI_ISL_633588, EPI_ISL_633591, EPI_ISL_633592, EPI_ISL_633593, EPI_ISL_633594, EPI_ISL_633595, EPI_ISL_633596, EPI_ISL_633597, EPI_ISL_633598, EPI_ISL_633599, EPI_ISL_633600, EPI_ISL_633601, EPI_ISL_633602, EPI_ISL_633603, EPI_ISL_633604, EPI_ISL_633605, EPI_ISL_633606, EPI_ISL_633607, EPI_ISL_633608, EPI_ISL_633609, EPI_ISL_633610, EPI_ISL_633611, EPI_ISL_633612, EPI_ISL_633613, EPI_ISL_633614, EPI_ISL_633616, EPI_ISL_633617, EPI_ISL_633618, EPI_ISL_633619, EPI_ISL_633620, EPI_ISL_633621, EPI_ISL_633622, EPI_ISL_633623, EPI_ISL_633624, EPI_ISL_633625, EPI_ISL_633626, EPI_ISL_633627, EPI_ISL_633628, EPI_ISL_633629, EPI_ISL_633630, EPI_ISL_633631, EPI_ISL_633632, EPI_ISL_633633, EPI_ISL_633634, EPI_ISL_633635, EPI_ISL_633636, EPI_ISL_633637, EPI_ISL_633638, EPI_ISL_633639, EPI_ISL_633640, EPI_ISL_633641, EPI_ISL_633643, EPI_ISL_633644, EPI_ISL_633645, EPI_ISL_633647, EPI_ISL_633648, EPI_ISL_633649, EPI_ISL_633650, EPI_ISL_633651, EPI_ISL_633652, EPI_ISL_633654, EPI_ISL_633655, EPI_ISL_633656, EPI_ISL_633657, EPI_ISL_633658, EPI_ISL_633659, EPI_ISL_633660, EPI_ISL_633661, EPI_ISL_633662, EPI_ISL_633663, EPI_ISL_633664, EPI_ISL_633665, EPI_ISL_633666, EPI_ISL_633667, EPI_ISL_633668, EPI_ISL_633669, EPI_ISL_633670, EPI_ISL_633671, EPI_ISL_633672, EPI_ISL_633673, EPI_ISL_633674, EPI_ISL_633675, EPI_ISL_633676, EPI_ISL_633677, EPI_ISL_633678, EPI_ISL_633679, EPI_ISL_633680, EPI_ISL_633681, EPI_ISL_633682, EPI_ISL_633683, EPI_ISL_633684, EPI_ISL_633685, EPI_ISL_633686, EPI_ISL_633687, EPI_ISL_633688, EPI_ISL_633689, EPI_ISL_633690, EPI_ISL_633691, EPI_ISL_633692, EPI_ISL_633694, EPI_ISL_633695, EPI_ISL_633698, EPI_ISL_633699, EPI_ISL_633701, EPI_ISL_633702, EPI_ISL_633703, EPI_ISL_633704, EPI_ISL_633705, EPI_ISL_633707, EPI_ISL_633709, EPI_ISL_633710, EPI_ISL_633711, EPI_ISL_633712, EPI_ISL_633713, EPI_ISL_633714, EPI_ISL_633715, EPI_ISL_633716, EPI_ISL_633717, EPI_ISL_633718, EPI_ISL_633719, EPI_ISL_633720, EPI_ISL_633721, EPI_ISL_633722, EPI_ISL_633723, EPI_ISL_633724, EPI_ISL_633725, EPI_ISL_633726, EPI_ISL_633727, EPI_ISL_633728, EPI_ISL_633729, EPI_ISL_633731, EPI_ISL_633732, EPI_ISL_633733, EPI_ISL_633734, EPI_ISL_633735, EPI_ISL_633736, EPI_ISL_633737, EPI_ISL_633738, EPI_ISL_633739, EPI_ISL_633740, EPI_ISL_633741, EPI_ISL_633742, EPI_ISL_633743, EPI_ISL_633744, EPI_ISL_633745, EPI_ISL_633746, EPI_ISL_633747, EPI_ISL_633748, EPI_ISL_633749, EPI_ISL_633750, EPI_ISL_633752, EPI_ISL_633753, EPI_ISL_633754, EPI_ISL_633755, EPI_ISL_633756, EPI_ISL_633757, EPI_ISL_633758, EPI_ISL_633761, EPI_ISL_633762, EPI_ISL_633763, EPI_ISL_633764, EPI_ISL_633765, EPI_ISL_633766, EPI_ISL_633767, EPI_ISL_633768, EPI_ISL_633769, EPI_ISL_633770, EPI_ISL_633771, EPI_ISL_633772, EPI_ISL_633773, EPI_ISL_633774, EPI_ISL_633775, EPI_ISL_633776, EPI_ISL_633778, EPI_ISL_633779, EPI_ISL_633780, EPI_ISL_633783, EPI_ISL_633784, EPI_ISL_633785, EPI_ISL_633787, EPI_ISL_633789, EPI_ISL_633790, EPI_ISL_633791, EPI_ISL_633792, EPI_ISL_633793, EPI_ISL_633795, EPI_ISL_633796, EPI_ISL_633797, EPI_ISL_633798, EPI_ISL_633801, EPI_ISL_633802, EPI_ISL_633803, EPI_ISL_633804, EPI_ISL_633805, EPI_ISL_633807, EPI_ISL_633808, EPI_ISL_633810, EPI_ISL_633811, EPI_ISL_633812, EPI_ISL_633816, EPI_ISL_633817, EPI_ISL_633818, EPI_ISL_633819, EPI_ISL_633820, EPI_ISL_633822, EPI_ISL_633823, EPI_ISL_633824, EPI_ISL_633826, EPI_ISL_633827, EPI_ISL_633829, EPI_ISL_633830, EPI_ISL_633832, EPI_ISL_633834, EPI_ISL_633836, EPI_ISL_633837, EPI_ISL_633838, EPI_ISL_633839, EPI_ISL_633840, EPI_ISL_633841, EPI_ISL_633842, EPI_ISL_633843, EPI_ISL_633844, EPI_ISL_633846, EPI_ISL_633847, EPI_ISL_633848, EPI_ISL_633850, EPI_ISL_633852, EPI_ISL_633853, EPI_ISL_633854, EPI_ISL_633855, EPI_ISL_633856, EPI_ISL_633857, EPI_ISL_633858, EPI_ISL_633859, EPI_ISL_633861, EPI_ISL_633862, EPI_ISL_633863, EPI_ISL_633864, EPI_ISL_633865, EPI_ISL_633866, EPI_ISL_633867, EPI_ISL_633868, EPI_ISL_633869, EPI_ISL_633870, EPI_ISL_633871, EPI_ISL_633873, EPI_ISL_633874, EPI_ISL_633875, EPI_ISL_633876, EPI_ISL_633879, EPI_ISL_633880, EPI_ISL_633881, EPI_ISL_633882, EPI_ISL_633883, EPI_ISL_633884, EPI_ISL_633885, EPI_ISL_633888, EPI_ISL_633890, EPI_ISL_633891, EPI_ISL_633892, EPI_ISL_633894, EPI_ISL_633895, EPI_ISL_633896, EPI_ISL_633897, EPI_ISL_633898, EPI_ISL_633899, EPI_ISL_633901, EPI_ISL_633902, EPI_ISL_633904, EPI_ISL_633906, EPI_ISL_633908, EPI_ISL_633909, EPI_ISL_633911, EPI_ISL_633912, EPI_ISL_633913, EPI_ISL_633915, EPI_ISL_633917, EPI_ISL_633918, EPI_ISL_633919, EPI_ISL_633920, EPI_ISL_633921, EPI_ISL_633924, EPI_ISL_633925, EPI_ISL_633926, EPI_ISL_633927, EPI_ISL_633928, EPI_ISL_633929, EPI_ISL_633930, EPI_ISL_633931, EPI_ISL_633932, EPI_ISL_633933, EPI_ISL_633934, EPI_ISL_633935, EPI_ISL_633936, EPI_ISL_633938, EPI_ISL_633939, EPI_ISL_633940, EPI_ISL_633941, EPI_ISL_633942, EPI_ISL_633944, EPI_ISL_633945, EPI_ISL_633946, EPI_ISL_633947, EPI_ISL_633948, EPI_ISL_633949, EPI_ISL_633950, EPI_ISL_633951, EPI_ISL_633952, EPI_ISL_633953, EPI_ISL_633954, EPI_ISL_633955, EPI_ISL_633956, EPI_ISL_633957, EPI_ISL_633958, EPI_ISL_633959, EPI_ISL_633960, EPI_ISL_633961, EPI_ISL_633962, EPI_ISL_633963, EPI_ISL_633964, EPI_ISL_633965, EPI_ISL_633966, EPI_ISL_633967, EPI_ISL_633968, EPI_ISL_633969, EPI_ISL_633970, EPI_ISL_633971, EPI_ISL_633973, EPI_ISL_633974, EPI_ISL_633975, EPI_ISL_633976, EPI_ISL_633977, EPI_ISL_633978, EPI_ISL_633979, EPI_ISL_633980, EPI_ISL_633981, EPI_ISL_633982, EPI_ISL_633983, EPI_ISL_633984, EPI_ISL_633985, EPI_ISL_633986, EPI_ISL_633987, EPI_ISL_633989, EPI_ISL_633990, EPI_ISL_633991, EPI_ISL_633992, EPI_ISL_633993, EPI_ISL_633994, EPI_ISL_633995, EPI_ISL_633996, EPI_ISL_633997, EPI_ISL_633998, EPI_ISL_633999, EPI_ISL_634000, EPI_ISL_634001, EPI_ISL_634002, EPI_ISL_634003, EPI_ISL_634004, EPI_ISL_634005, EPI_ISL_634006, EPI_ISL_634007, EPI_ISL_634008, EPI_ISL_634009, EPI_ISL_634010, EPI_ISL_634011, EPI_ISL_634012, EPI_ISL_634013, EPI_ISL_634014, EPI_ISL_634015, EPI_ISL_634016, EPI_ISL_634017, EPI_ISL_634019, EPI_ISL_634020, EPI_ISL_634021, EPI_ISL_634022, EPI_ISL_634023, EPI_ISL_634024, EPI_ISL_634025, EPI_ISL_634026, EPI_ISL_634027, EPI_ISL_634028, EPI_ISL_634029, EPI_ISL_634030, EPI_ISL_634031, EPI_ISL_634032, EPI_ISL_634033, EPI_ISL_634034, EPI_ISL_634035, EPI_ISL_634036, EPI_ISL_634037, EPI_ISL_634038, EPI_ISL_634039, EPI_ISL_634040, EPI_ISL_634042, EPI_ISL_634044, EPI_ISL_634045, EPI_ISL_634046, EPI_ISL_634047, EPI_ISL_634048, EPI_ISL_634049, EPI_ISL_634050, EPI_ISL_634051, EPI_ISL_634052, EPI_ISL_634054, EPI_ISL_634055, EPI_ISL_634056, EPI_ISL_634057, EPI_ISL_634058, EPI_ISL_634059, EPI_ISL_634060, EPI_ISL_634061, EPI_ISL_634062, EPI_ISL_634064, EPI_ISL_634065, EPI_ISL_634066, EPI_ISL_634067, EPI_ISL_634068, EPI_ISL_634069, EPI_ISL_634070, EPI_ISL_634071, EPI_ISL_634073, EPI_ISL_634074, EPI_ISL_634075, EPI_ISL_634076, EPI_ISL_634077, EPI_ISL_634078, EPI_ISL_634079, EPI_ISL_634080, EPI_ISL_634081, EPI_ISL_634082, EPI_ISL_634083, EPI_ISL_634084, EPI_ISL_634085, EPI_ISL_634086, EPI_ISL_634087, EPI_ISL_634088, EPI_ISL_634089, EPI_ISL_634090, EPI_ISL_634091, EPI_ISL_634093, EPI_ISL_634095, EPI_ISL_634096, EPI_ISL_634097, EPI_ISL_634099, EPI_ISL_634100, EPI_ISL_634101, EPI_ISL_634102, EPI_ISL_634103, EPI_ISL_634104, EPI_ISL_634105, EPI_ISL_634106, EPI_ISL_634110, EPI_ISL_634111, EPI_ISL_634112, EPI_ISL_634114, EPI_ISL_634115, EPI_ISL_634116, EPI_ISL_634117, EPI_ISL_634118, EPI_ISL_634119, EPI_ISL_634120, EPI_ISL_634121, EPI_ISL_634122, EPI_ISL_634124, EPI_ISL_634125, EPI_ISL_634126, EPI_ISL_634128, EPI_ISL_634129, EPI_ISL_634130, EPI_ISL_634131, EPI_ISL_634132, EPI_ISL_634133, EPI_ISL_634134, EPI_ISL_634135, EPI_ISL_634136, EPI_ISL_634137, EPI_ISL_634138, EPI_ISL_634139, EPI_ISL_634141, EPI_ISL_634142, EPI_ISL_634143, EPI_ISL_634144, EPI_ISL_634145, EPI_ISL_634146, EPI_ISL_634147, EPI_ISL_634150, EPI_ISL_634151, EPI_ISL_634152, EPI_ISL_634153, EPI_ISL_634154, EPI_ISL_634155, EPI_ISL_634157, EPI_ISL_634158, EPI_ISL_634159, EPI_ISL_634160, EPI_ISL_634161, EPI_ISL_634162, EPI_ISL_634163, EPI_ISL_634165, EPI_ISL_634166, EPI_ISL_634167, EPI_ISL_634168, EPI_ISL_634169, EPI_ISL_634170, EPI_ISL_634171, EPI_ISL_634172, EPI_ISL_634173, EPI_ISL_634174, EPI_ISL_634175, EPI_ISL_634176, EPI_ISL_634179, EPI_ISL_634180, EPI_ISL_634181, EPI_ISL_634182, EPI_ISL_634183, EPI_ISL_634184, EPI_ISL_634185, EPI_ISL_634186, EPI_ISL_634188, EPI_ISL_634189, EPI_ISL_634190, EPI_ISL_634191, EPI_ISL_634192, EPI_ISL_634193, EPI_ISL_634195, EPI_ISL_634196, EPI_ISL_634197, EPI_ISL_634198, EPI_ISL_634199, EPI_ISL_634200, EPI_ISL_634203, EPI_ISL_634204, EPI_ISL_634205, EPI_ISL_634206, EPI_ISL_634207, EPI_ISL_634208, EPI_ISL_634209, EPI_ISL_634210, EPI_ISL_634211, EPI_ISL_634212, EPI_ISL_634213, EPI_ISL_634214, EPI_ISL_634215, EPI_ISL_634216, EPI_ISL_634217, EPI_ISL_634218, EPI_ISL_634219, EPI_ISL_634220, EPI_ISL_634222, EPI_ISL_634223, EPI_ISL_634226, EPI_ISL_634227, EPI_ISL_634228, EPI_ISL_634229, EPI_ISL_634231, EPI_ISL_634232, EPI_ISL_634233, EPI_ISL_634234, EPI_ISL_634235, EPI_ISL_634236, EPI_ISL_634237, EPI_ISL_634239, EPI_ISL_634240, EPI_ISL_634241, EPI_ISL_634243, EPI_ISL_634244, EPI_ISL_634245, EPI_ISL_634246, EPI_ISL_634247, EPI_ISL_634248, EPI_ISL_634249, EPI_ISL_634250, EPI_ISL_634252			

see above	Lighthouse Lab in Glasgow	Wellcome Sanger Institute for the COVID-19 Genomics UK (COG-UK) consortium	Harper VanSteenhouse, Yumi Kasai, David Gray, Carol Clugston, Anna Dominiczak and Alex Alderton, Roberto Amato, Sonia Goncalves, Ewan Harrison, David K. Jackson, Ian Johnston, Dominic Kwiatkowski, Cordelia Langford, John Sillitoe on behalf of the Wellcome Sanger Institute COVID-19 Surveillance Team
EPI_ISL_634256, EPI_ISL_634258, EPI_ISL_634259, EPI_ISL_634261, EPI_ISL_634262, EPI_ISL_634263, EPI_ISL_634264, EPI_ISL_634265, EPI_ISL_634267, EPI_ISL_634268, EPI_ISL_634269, EPI_ISL_634270, EPI_ISL_634271, EPI_ISL_634272, EPI_ISL_634273, EPI_ISL_634274, EPI_ISL_634275, EPI_ISL_634276, EPI_ISL_634277, EPI_ISL_634278, EPI_ISL_634280, EPI_ISL_634281, EPI_ISL_634282, EPI_ISL_634283, EPI_ISL_634284, EPI_ISL_634285, EPI_ISL_634286, EPI_ISL_634287, EPI_ISL_634288, EPI_ISL_634289, EPI_ISL_634290, EPI_ISL_634291, EPI_ISL_634292, EPI_ISL_634293, EPI_ISL_634294, EPI_ISL_634295, EPI_ISL_634296, EPI_ISL_634297, EPI_ISL_634298, EPI_ISL_634299, EPI_ISL_634300, EPI_ISL_634303, EPI_ISL_634305, EPI_ISL_634306, EPI_ISL_634307, EPI_ISL_634308, EPI_ISL_634309, EPI_ISL_634310, EPI_ISL_634312, EPI_ISL_634313, EPI_ISL_634314, EPI_ISL_634315, EPI_ISL_634316, EPI_ISL_634317, EPI_ISL_634319, EPI_ISL_634321, EPI_ISL_634322, EPI_ISL_634323, EPI_ISL_634324, EPI_ISL_634325, EPI_ISL_634327, EPI_ISL_634328, EPI_ISL_634329, EPI_ISL_634330, EPI_ISL_634331, EPI_ISL_634332, EPI_ISL_634334, EPI_ISL_634335, EPI_ISL_634336, EPI_ISL_634340, EPI_ISL_634342, EPI_ISL_634343, EPI_ISL_634344, EPI_ISL_634345, EPI_ISL_634346, EPI_ISL_634347, EPI_ISL_634348, EPI_ISL_634349, EPI_ISL_634350, EPI_ISL_634351, EPI_ISL_634352, EPI_ISL_634354, EPI_ISL_634355, EPI_ISL_634356, EPI_ISL_634357, EPI_ISL_634358, EPI_ISL_634359, EPI_ISL_634360, EPI_ISL_634361, EPI_ISL_634362, EPI_ISL_634364, EPI_ISL_634365, EPI_ISL_634367, EPI_ISL_634368, EPI_ISL_634369, EPI_ISL_634370, EPI_ISL_634371, EPI_ISL_634372, EPI_ISL_634373, EPI_ISL_634374, EPI_ISL_634375, EPI_ISL_634378, EPI_ISL_634379, EPI_ISL_634380, EPI_ISL_634381, EPI_ISL_634382, EPI_ISL_634383, EPI_ISL_634386, EPI_ISL_634387, EPI_ISL_634388, EPI_ISL_634389, EPI_ISL_634390, EPI_ISL_634392, EPI_ISL_634393, EPI_ISL_634394, EPI_ISL_634396, EPI_ISL_634397, EPI_ISL_634398, EPI_ISL_634399, EPI_ISL_634400, EPI_ISL_634401, EPI_ISL_634402, EPI_ISL_634403, EPI_ISL_634404, EPI_ISL_634405, EPI_ISL_634407, EPI_ISL_634409, EPI_ISL_634410, EPI_ISL_634411, EPI_ISL_634412, EPI_ISL_634413, EPI_ISL_634414, EPI_ISL_634415, EPI_ISL_634416, EPI_ISL_634417, EPI_ISL_634418, EPI_ISL_634419, EPI_ISL_634420, EPI_ISL_634421, EPI_ISL_634422, EPI_ISL_634423, EPI_ISL_634424, EPI_ISL_634425, EPI_ISL_634426, EPI_ISL_634427, EPI_ISL_634429, EPI_ISL_634430, EPI_ISL_634431, EPI_ISL_634432, EPI_ISL_634433, EPI_ISL_634434, EPI_ISL_634435, EPI_ISL_634436, EPI_ISL_634437, EPI_ISL_634438, EPI_ISL_634439, EPI_ISL_634440, EPI_ISL_634441, EPI_ISL_634442, EPI_ISL_634443, EPI_ISL_634444, EPI_ISL_634446, EPI_ISL_634448, EPI_ISL_634449, EPI_ISL_634450, EPI_ISL_634451, EPI_ISL_634452, EPI_ISL_634453, EPI_ISL_634455, EPI_ISL_634456, EPI_ISL_634457, EPI_ISL_634458, EPI_ISL_634459, EPI_ISL_634461, EPI_ISL_634463, EPI_ISL_634464, EPI_ISL_634466, EPI_ISL_634467, EPI_ISL_634468, EPI_ISL_634469, EPI_ISL_634470, EPI_ISL_634471, EPI_ISL_634472, EPI_ISL_634473, EPI_ISL_634474, EPI_ISL_634476, EPI_ISL_634477, EPI_ISL_634478, EPI_ISL_634479, EPI_ISL_634481, EPI_ISL_634482, EPI_ISL_634484, EPI_ISL_634486, EPI_ISL_634487, EPI_ISL_634488, EPI_ISL_634489, EPI_ISL_634490, EPI_ISL_634491, EPI_ISL_634494, EPI_ISL_634495, EPI_ISL_634497, EPI_ISL_634498, EPI_ISL_634500, EPI_ISL_634501, EPI_ISL_634502, EPI_ISL_634503, EPI_ISL_634504, EPI_ISL_634505, EPI_ISL_634506, EPI_ISL_634507, EPI_ISL_634508, EPI_ISL_634509, EPI_ISL_634511, EPI_ISL_634512, EPI_ISL_634513, EPI_ISL_634514, EPI_ISL_634515, EPI_ISL_634516, EPI_ISL_634518, EPI_ISL_634519			
see above	Lighthouse Lab in Milton Keynes	Wellcome Sanger Institute for the COVID-19 Genomics UK (COG-UK) consortium	The Lighthouse Lab in Milton Keynes and Alex Alderton, Roberto Amato, Sonia Goncalves, Ewan Harrison, David K. Jackson, Ian Johnston, Dominic Kwiatkowski, Cordelia Langford, John Sillitoe on behalf of the Wellcome Sanger Institute COVID-19 Surveillance Team
EPI_ISL_634520, EPI_ISL_634521, EPI_ISL_634522, EPI_ISL_634523, EPI_ISL_634524, EPI_ISL_634525, EPI_ISL_634526, EPI_ISL_634527, EPI_ISL_634528, EPI_ISL_634529, EPI_ISL_634530, EPI_ISL_634531, EPI_ISL_634532, EPI_ISL_634533, EPI_ISL_634535, EPI_ISL_634536, EPI_ISL_634538, EPI_ISL_634539, EPI_ISL_634541, EPI_ISL_634542, EPI_ISL_634543, EPI_ISL_634544, EPI_ISL_634545, EPI_ISL_634546, EPI_ISL_634547, EPI_ISL_634548, EPI_ISL_634549, EPI_ISL_634550, EPI_ISL_634551, EPI_ISL_634552, EPI_ISL_634553, EPI_ISL_634554, EPI_ISL_634555, EPI_ISL_634556, EPI_ISL_634557, EPI_ISL_634558, EPI_ISL_634559, EPI_ISL_634560, EPI_ISL_634561, EPI_ISL_634562, EPI_ISL_634563, EPI_ISL_634564, EPI_ISL_634565, EPI_ISL_634566, EPI_ISL_634567, EPI_ISL_634568, EPI_ISL_634569, EPI_ISL_634570, EPI_ISL_634571, EPI_ISL_634572, EPI_ISL_634573, EPI_ISL_634574, EPI_ISL_634575, EPI_ISL_634576, EPI_ISL_634578, EPI_ISL_634579, EPI_ISL_634580, EPI_ISL_634581, EPI_ISL_634582, EPI_ISL_634583, EPI_ISL_634584, EPI_ISL_634585, EPI_ISL_634586, EPI_ISL_634587, EPI_ISL_634588, EPI_ISL_634589, EPI_ISL_634591, EPI_ISL_634592, EPI_ISL_634593, EPI_ISL_634594, EPI_ISL_634595, EPI_ISL_634596, EPI_ISL_634597, EPI_ISL_634598, EPI_ISL_634599			
see above	Lighthouse Lab in Glasgow	Wellcome Sanger Institute for the COVID-19 Genomics UK (COG-UK) consortium	Harper VanSteenhouse, Yumi Kasai, David Gray, Carol Clugston, Anna Dominiczak and Alex Alderton, Roberto Amato, Sonia Goncalves, Ewan Harrison, David K. Jackson, Ian Johnston, Dominic Kwiatkowski, Cordelia Langford, John Sillitoe on behalf of the Wellcome Sanger Institute COVID-19 Surveillance Team
EPI_ISL_634600, EPI_ISL_634601, EPI_ISL_634602, EPI_ISL_634603, EPI_ISL_634605, EPI_ISL_634606, EPI_ISL_634607, EPI_ISL_634608, EPI_ISL_634609, EPI_ISL_634610, EPI_ISL_634611, EPI_ISL_634612, EPI_ISL_634614, EPI_ISL_634615, EPI_ISL_634616, EPI_ISL_634617, EPI_ISL_634618, EPI_ISL_634619, EPI_ISL_634620, EPI_ISL_634621, EPI_ISL_634622, EPI_ISL_634623, EPI_ISL_634624, EPI_ISL_634625, EPI_ISL_634626, EPI_ISL_634627, EPI_ISL_634628, EPI_ISL_634629, EPI_ISL_634630, EPI_ISL_634631, EPI_ISL_634632, EPI_ISL_634633, EPI_ISL_634634, EPI_ISL_634635, EPI_ISL_634636, EPI_ISL_634638, EPI_ISL_634639, EPI_ISL_634640, EPI_ISL_634641, EPI_ISL_634642, EPI_ISL_634643, EPI_ISL_634644, EPI_ISL_634645, EPI_ISL_634646, EPI_ISL_634647, EPI_ISL_634648, EPI_ISL_634649, EPI_ISL_634650, EPI_ISL_634651, EPI_ISL_634652, EPI_ISL_634653, EPI_ISL_634654, EPI_ISL_634655, EPI_ISL_634656, EPI_ISL_634657, EPI_ISL_634658, EPI_ISL_634659, EPI_ISL_634660, EPI_ISL_634661, EPI_ISL_634662, EPI_ISL_634663, EPI_ISL_634664, EPI_ISL_634666, EPI_ISL_634667, EPI_ISL_634668, EPI_ISL_634669, EPI_ISL_634670, EPI_ISL_634671, EPI_ISL_634672, EPI_ISL_634673, EPI_ISL_634674, EPI_ISL_634675, EPI_ISL_634676, EPI_ISL_634677, EPI_ISL_634679, EPI_ISL_634680, EPI_ISL_634681, EPI_ISL_634682, EPI_ISL_634683, EPI_ISL_634684, EPI_ISL_634685, EPI_ISL_634686, EPI_ISL_634687, EPI_ISL_634688, EPI_ISL_634689, EPI_ISL_634690, EPI_ISL_634691, EPI_ISL_634692, EPI_ISL_634693, EPI_ISL_634694, EPI_ISL_634695, EPI_ISL_634696, EPI_ISL_634697, EPI_ISL_634698, EPI_ISL_634699, EPI_ISL_634700, EPI_ISL_634701, EPI_ISL_634702, EPI_ISL_634703, EPI_ISL_634705, EPI_ISL_634706, EPI_ISL_634708, EPI_ISL_634709, EPI_ISL_634710, EPI_ISL_634711, EPI_ISL_634712, EPI_ISL_634714, EPI_ISL_634715, EPI_ISL_634716, EPI_ISL_634717, EPI_ISL_634718, EPI_ISL_634720, EPI_ISL_634721, EPI_ISL_634722, EPI_ISL_634723, EPI_ISL_634725, EPI_ISL_634726, EPI_ISL_634727, EPI_ISL_634728, EPI_ISL_634729, EPI_ISL_634730, EPI_ISL_634731, EPI_ISL_634732, EPI_ISL_634733, EPI_ISL_634735, EPI_ISL_634736, EPI_ISL_634738, EPI_ISL_634739, EPI_ISL_634740, EPI_ISL_634741, EPI_ISL_634742, EPI_ISL_634744, EPI_ISL_634745, EPI_ISL_634746, EPI_ISL_634747, EPI_ISL_634748, EPI_ISL_634749, EPI_ISL_634750, EPI_ISL_634751, EPI_ISL_634752, EPI_ISL_634753			
see above	Lighthouse Lab in Glasgow	Wellcome Sanger Institute for the COVID-19 Genomics UK (COG-UK) consortium	Harper VanSteenhouse, Yumi Kasai, David Gray, Carol Clugston, Anna Dominiczak and Alex Alderton, Roberto Amato, Sonia Goncalves, Ewan Harrison, David K. Jackson, Ian Johnston, Dominic Kwiatkowski, Cordelia Langford, John Sillitoe on behalf of the Wellcome Sanger Institute COVID-19 Surveillance Team (http://www.sanger.ac.uk/covid-team)
EPI_ISL_634754, EPI_ISL_634755, EPI_ISL_634756, EPI_ISL_634758, EPI_ISL_634759, EPI_ISL_634760, EPI_ISL_634761, EPI_ISL_634762, EPI_ISL_634763, EPI_ISL_634764, EPI_ISL_634766, EPI_ISL_634767, EPI_ISL_634768, EPI_ISL_634769, EPI_ISL_634770, EPI_ISL_634771, EPI_ISL_634772, EPI_ISL_634773, EPI_ISL_634774, EPI_ISL_634775, EPI_ISL_634776, EPI_ISL_634777, EPI_ISL_634778, EPI_ISL_634781, EPI_ISL_634782, EPI_ISL_634783, EPI_ISL_634784, EPI_ISL_634785, EPI_ISL_634786, EPI_ISL_634788			
see above	Lighthouse Lab in Alderley Park	Wellcome Sanger Institute for the COVID-19 Genomics UK (COG-UK) consortium	Jacquelyn Wynn, Mairead Hyland, The Lighthouse Lab in Alderley Park and Alex Alderton, Roberto Amato, Sonia Goncalves, Ewan Harrison, David K. Jackson, Ian Johnston, Dominic Kwiatkowski, Cordelia Langford, John Sillitoe on behalf of the Wellcome Sanger Institute COVID-19 Surveillance Team (http://www.sanger.ac.uk/covid-team)
EPI_ISL_634790, EPI_ISL_634791, EPI_ISL_634792, EPI_ISL_634793, EPI_ISL_634794, EPI_ISL_634795, EPI_ISL_634796, EPI_ISL_634797, EPI_ISL_634798, EPI_ISL_634799, EPI_ISL_634800, EPI_ISL_634801, EPI_ISL_634802, EPI_ISL_634803, EPI_ISL_634804, EPI_ISL_634805, EPI_ISL_634806, EPI_ISL_634807, EPI_ISL_634808, EPI_ISL_634809, EPI_ISL_634810, EPI_ISL_634811, EPI_ISL_634812, EPI_ISL_634813, EPI_ISL_634814, EPI_ISL_634815, EPI_ISL_634816			
see above	Lighthouse Lab in Cambridge	Wellcome Sanger Institute for the COVID-19 Genomics UK (COG-UK) consortium	Rob Howes, The Lighthouse Lab in Cambridge and Alex Alderton, Roberto Amato, Sonia Goncalves, Ewan Harrison, David K. Jackson, Ian Johnston, Dominic Kwiatkowski, Cordelia Langford, John Sillitoe on behalf of the Wellcome Sanger Institute COVID-19 Surveillance Team (http://www.sanger.ac.uk/covid-team)
EPI_ISL_634817	Laboratoire de virologie, CHU de Grenoble - CS 10217 - 38043 Grenoble cedex 9	CNR Virus des Infections Respiratoires - France SUD	Antonin Bal, Gregory Destras, Gwendolyne Burfin, Hadrien Règue, Quentin Semanas, Martine Valette, Bruno Lina, Sylvie Larrat, Laurence Josset
EPI_ISL_634818	Laboratoire de virologie, CHU de Grenoble - CS 10217 - 38043 Grenoble cedex 10	CNR Virus des Infections Respiratoires - France SUD	Antonin Bal, Gregory Destras, Gwendolyne Burfin, Hadrien Règue, Quentin Semanas, Martine Valette, Bruno Lina, Sylvie Larrat, Laurence Josset
EPI_ISL_634819	Laboratoire de virologie, CHU de Grenoble - CS 10217 - 38043 Grenoble cedex 11	CNR Virus des Infections Respiratoires - France SUD	Antonin Bal, Gregory Destras, Gwendolyne Burfin, Hadrien Règue, Quentin Semanas, Martine Valette, Bruno Lina, Sylvie Larrat, Laurence Josset
EPI_ISL_634820	Laboratoire de virologie, CHU de Grenoble - CS 10217 - 38043 Grenoble cedex 12	CNR Virus des Infections Respiratoires - France SUD	Antonin Bal, Gregory Destras, Gwendolyne Burfin, Hadrien Règue, Quentin Semanas, Martine Valette, Bruno Lina, Sylvie Larrat, Laurence Josset
EPI_ISL_634821	Laboratoire de virologie, CHU de Grenoble - CS 10217 - 38043 Grenoble cedex 13	CNR Virus des Infections Respiratoires - France SUD	Antonin Bal, Gregory Destras, Gwendolyne Burfin, Hadrien Règue, Quentin Semanas, Martine Valette, Bruno Lina, Sylvie Larrat, Laurence Josset
EPI_ISL_634822	Laboratoire de virologie, CHU de Grenoble - CS 10217 - 38043 Grenoble cedex 14	CNR Virus des Infections Respiratoires - France SUD	Antonin Bal, Gregory Destras, Gwendolyne Burfin, Hadrien Règue, Quentin Semanas, Martine Valette, Bruno Lina, Sylvie Larrat, Laurence Josset
EPI_ISL_634823	Laboratoire de virologie, CHU de Grenoble - CS 10217 - 38043 Grenoble cedex 15	CNR Virus des Infections Respiratoires - France SUD	Antonin Bal, Gregory Destras, Gwendolyne Burfin, Hadrien Règue, Quentin Semanas, Martine Valette, Bruno Lina, Sylvie Larrat, Laurence Josset
EPI_ISL_634824	Laboratoire de virologie, CHU de Grenoble - CS 10217 - 38043 Grenoble cedex 16	CNR Virus des Infections Respiratoires - France SUD	Antonin Bal, Gregory Destras, Gwendolyne Burfin, Hadrien Règue, Quentin Semanas, Martine Valette, Bruno Lina, Sylvie Larrat, Laurence Josset
EPI_ISL_634825	Laboratoire de virologie, CHU de Grenoble - CS 10217 - 38043 Grenoble cedex 17	CNR Virus des Infections Respiratoires - France SUD	Antonin Bal, Gregory Destras, Gwendolyne Burfin, Hadrien Règue, Quentin Semanas, Martine Valette, Bruno Lina, Sylvie Larrat, Laurence Josset
EPI_ISL_634826	Laboratoire de virologie, CHU de Grenoble - CS 10217 - 38043 Grenoble cedex 18	CNR Virus des Infections Respiratoires - France SUD	Antonin Bal, Gregory Destras, Gwendolyne Burfin, Hadrien Règue, Quentin Semanas, Martine Valette, Bruno Lina, Sylvie Larrat, Laurence Josset
EPI_ISL_634827	Laboratoire de virologie, CHU de Grenoble - CS 10217 - 38043 Grenoble cedex 19	CNR Virus des Infections Respiratoires - France SUD	Antonin Bal, Gregory Destras, Gwendolyne Burfin, Hadrien Règue, Quentin Semanas, Martine Valette, Bruno Lina, Sylvie Larrat, Laurence Josset
EPI_ISL_634828	Laboratoire de virologie, CHU de Grenoble - CS 10217 - 38043 Grenoble cedex 20	CNR Virus des Infections Respiratoires - France SUD	Antonin Bal, Gregory Destras, Gwendolyne Burfin, Hadrien Règue, Quentin Semanas, Martine Valette, Bruno Lina, Sylvie Larrat, Laurence Josset
EPI_ISL_634829	Laboratoire de virologie, CHU de Grenoble - CS 10217 - 38043 Grenoble cedex 21	CNR Virus des Infections Respiratoires - France SUD	Antonin Bal, Gregory Destras, Gwendolyne Burfin, Hadrien Règue, Quentin Semanas, Martine Valette, Bruno Lina, Sylvie Larrat, Laurence Josset
EPI_ISL_634830	Laboratoire de virologie, CHU de Grenoble - CS 10217 - 38043 Grenoble cedex 22	CNR Virus des Infections Respiratoires - France SUD	Antonin Bal, Gregory Destras, Gwendolyne Burfin, Hadrien Règue, Quentin Semanas, Martine Valette, Bruno Lina, Sylvie Larrat, Laurence Josset

EPI_ISL_634831	Laboratoire de virologie, CHU de Grenoble - CS 10217 - 38043 Grenoble cedex 23	CNR Virus des Infections Respiratoires - France SUD	Antonin Bal, Gregory Destras, Gwendolyne Burfin, Hadrien Règue, Quentin Semanas, Martine Valette, Bruno Lina, Sylvie Larrat, Laurence Josset
EPI_ISL_634832	Laboratoire de virologie, CHU de Grenoble - CS 10217 - 38043 Grenoble cedex 24	CNR Virus des Infections Respiratoires - France SUD	Antonin Bal, Gregory Destras, Gwendolyne Burfin, Hadrien Règue, Quentin Semanas, Martine Valette, Bruno Lina, Sylvie Larrat, Laurence Josset
EPI_ISL_634833	Laboratoire de virologie, CHU de Grenoble - CS 10217 - 38043 Grenoble cedex 25	CNR Virus des Infections Respiratoires - France SUD	Antonin Bal, Gregory Destras, Gwendolyne Burfin, Hadrien Règue, Quentin Semanas, Martine Valette, Bruno Lina, Sylvie Larrat, Laurence Josset
EPI_ISL_634834	Laboratoire de virologie, CHU de Grenoble - CS 10217 - 38043 Grenoble cedex 26	CNR Virus des Infections Respiratoires - France SUD	Antonin Bal, Gregory Destras, Gwendolyne Burfin, Hadrien Règue, Quentin Semanas, Martine Valette, Bruno Lina, Sylvie Larrat, Laurence Josset
EPI_ISL_634835	Laboratoire de virologie, CHU de Grenoble - CS 10217 - 38043 Grenoble cedex 27	CNR Virus des Infections Respiratoires - France SUD	Antonin Bal, Gregory Destras, Gwendolyne Burfin, Hadrien Règue, Quentin Semanas, Martine Valette, Bruno Lina, Sylvie Larrat, Laurence Josset
EPI_ISL_634836	Laboratoire de virologie, CHU de Grenoble - CS 10217 - 38043 Grenoble cedex 28	CNR Virus des Infections Respiratoires - France SUD	Antonin Bal, Gregory Destras, Gwendolyne Burfin, Hadrien Règue, Quentin Semanas, Martine Valette, Bruno Lina, Sylvie Larrat, Laurence Josset
EPI_ISL_634837	Laboratoire de virologie, CHU de Grenoble - CS 10217 - 38043 Grenoble cedex 29	CNR Virus des Infections Respiratoires - France SUD	Antonin Bal, Gregory Destras, Gwendolyne Burfin, Hadrien Règue, Quentin Semanas, Martine Valette, Bruno Lina, Sylvie Larrat, Laurence Josset
EPI_ISL_634838, EPI_ISL_634839, EPI_ISL_634840, EPI_ISL_634841, EPI_ISL_634842, EPI_ISL_634843, EPI_ISL_634844, EPI_ISL_634845, EPI_ISL_634846, EPI_ISL_634847, EPI_ISL_634848, EPI_ISL_634849, EPI_ISL_634850, EPI_ISL_634851, EPI_ISL_634852, EPI_ISL_634853, EPI_ISL_634854, EPI_ISL_634855, EPI_ISL_634856			
see above	Minnesota Department of Health, Public Health Laboratory	Minnesota Department of Health, Public Health Laboratory	Matt Plumb, Jacob Garfin, Alexandra Lorentz, and Xiong Wang
EPI_ISL_634857	Minneapolis VA Health Care System	Minnesota Department of Health, Public Health Laboratory	Matt Plumb, Jacob Garfin, Alexandra Lorentz, and Xiong Wang
EPI_ISL_634858, EPI_ISL_634859, EPI_ISL_634860, EPI_ISL_634861, EPI_ISL_634862, EPI_ISL_634863	Minnesota Department of Health, Public Health Laboratory	Minnesota Department of Health, Public Health Laboratory	Matt Plumb, Jacob Garfin, Alexandra Lorentz, and Xiong Wang
EPI_ISL_634864, EPI_ISL_634865	HealthPartners Central Lab	Minnesota Department of Health, Public Health Laboratory	Matt Plumb, Jacob Garfin, Alexandra Lorentz, and Xiong Wang
EPI_ISL_634866, EPI_ISL_634867, EPI_ISL_634868, EPI_ISL_634869, EPI_ISL_634870, EPI_ISL_634871, EPI_ISL_634872, EPI_ISL_634873, EPI_ISL_634874, EPI_ISL_634875	Minnesota Department of Health, Public Health Laboratory	Minnesota Department of Health, Public Health Laboratory	Matt Plumb, Jacob Garfin, Alexandra Lorentz, and Xiong Wang
EPI_ISL_634878, EPI_ISL_634879, EPI_ISL_634880, EPI_ISL_634881, EPI_ISL_634882, EPI_ISL_634883, EPI_ISL_634884, EPI_ISL_634885	Lab voor klinische biologie	Onderzoeksgroep Virologie	Laurens Lambrechts, Nick Vereecke, Marthe Pauwels, Bruno Verhasselt, Linos Vandekerckhove, Hans Nauwynck, Sebastiaan Theuns
EPI_ISL_634886, EPI_ISL_634887, EPI_ISL_634888, EPI_ISL_634889, EPI_ISL_634890, EPI_ISL_634891, EPI_ISL_634892, EPI_ISL_634893, EPI_ISL_634894, EPI_ISL_634895	Lab voor klinische biologie	Onderzoeksgroep Virologie	Nick Vereecke, Laurens Lambrechts, Marthe Pauwels, Bruno Verhasselt, Linos Vandekerckhove, Hans Nauwynck, Sebastiaan Theuns
EPI_ISL_634896, EPI_ISL_634898, EPI_ISL_634900, EPI_ISL_634901, EPI_ISL_634902, EPI_ISL_634903, EPI_ISL_634904, EPI_ISL_634905, EPI_ISL_634906, EPI_ISL_634907, EPI_ISL_634908, EPI_ISL_634909, EPI_ISL_634913, EPI_ISL_634914, EPI_ISL_634915, EPI_ISL_634916, EPI_ISL_634919, EPI_ISL_634921, EPI_ISL_634922, EPI_ISL_634924, EPI_ISL_634925, EPI_ISL_634926, EPI_ISL_634927, EPI_ISL_634928, EPI_ISL_634929, EPI_ISL_634931, EPI_ISL_634932			
see above	Utah Public Health Laboratory	Utah Public Health Laboratory	Erin L. Young, Kelly F. Oakeson
EPI_ISL_634977	1-Laboratory of Microbiology, National Reference Lab, Charles Nicolle Hospital; 2-University of Tunis ElManar, Faculty of Medicine of Tunis, LR99ES09, Tunis, Tunisia	1-Clinical and Experimental Pharmacology Lab, LR16SP02, National Center of Pharmacovigilance, University of Tunis El Manar, Tunis, Tunisia. 2-Neurodegenerative diseases and psychiatric troubles, LR18SP03, Razi Hospital, University of Tunis El Manar, Tunis, Tunisia. 3- Ministry of Health, National Observatory of New and Emerging Diseases, 1006, Tunis, Tunisia	Ilhem Boutiba-Ben Boubaker, Sameh Trabelsi, Nissaf Ben Alaya, Maher Kharrat, Alia Ben Kahla, Jalila Ben Khelil, Salma Abid, Sana Ferjani, Mouna Ben Sassi, Mouna Safer, Imen Mkada, Imen Kacem, Gaies Emna, Soumaya Rammeh, Riadh Daghfous, Riadh Gouider.
EPI_ISL_634978, EPI_ISL_634979, EPI_ISL_634981, EPI_ISL_634983, EPI_ISL_634984, EPI_ISL_634985, EPI_ISL_634986, EPI_ISL_634987, EPI_ISL_634989, EPI_ISL_634990, EPI_ISL_634993, EPI_ISL_634994, EPI_ISL_634995, EPI_ISL_634996, EPI_ISL_634997, EPI_ISL_634998, EPI_ISL_634999, EPI_ISL_635000, EPI_ISL_635001, EPI_ISL_635002, EPI_ISL_635003, EPI_ISL_635004, EPI_ISL_635005, EPI_ISL_635006, EPI_ISL_635007, EPI_ISL_635008, EPI_ISL_635009, EPI_ISL_635010, EPI_ISL_635011, EPI_ISL_635012, EPI_ISL_635013, EPI_ISL_635014, EPI_ISL_635015, EPI_ISL_635016, EPI_ISL_635017, EPI_ISL_635018, EPI_ISL_635019, EPI_ISL_635021, EPI_ISL_635022, EPI_ISL_635024, EPI_ISL_635025, EPI_ISL_635026, EPI_ISL_635027, EPI_ISL_635028, EPI_ISL_635029, EPI_ISL_635030, EPI_ISL_635031, EPI_ISL_635032, EPI_ISL_635033, EPI_ISL_635034, EPI_ISL_635035, EPI_ISL_635036, EPI_ISL_635037, EPI_ISL_635038, EPI_ISL_635039, EPI_ISL_635040, EPI_ISL_635041, EPI_ISL_635043, EPI_ISL_635045, EPI_ISL_635046, EPI_ISL_635047, EPI_ISL_635048, EPI_ISL_635049, EPI_ISL_635050, EPI_ISL_635051, EPI_ISL_635052, EPI_ISL_635053, EPI_ISL_635054, EPI_ISL_635055, EPI_ISL_635056, EPI_ISL_635057, EPI_ISL_635058			
see above	National Health Laboratory Service - Inkosi Albert Luthuli Central Hospital (NHLS-IALCH)	KRISP, KZN Research Innovation and Sequencing Platform	Giandhari J, Pillay S, Lessells R, Mdlalose K, York D, Khan S, Tegally H, Wilkinson E, de Oliveira T
EPI_ISL_635061, EPI_ISL_635062	1-Laboratory of Microbiology, National Reference Lab, Charles Nicolle Hospital; 2-University of Tunis ElManar, Faculty of Medicine of Tunis, LR99ES09, Tunis, Tunisia	1-Clinical and Experimental Pharmacology Lab, LR16SP02, National Center of Pharmacovigilance, University of Tunis El Manar, Tunis, Tunisia. 2-Neurodegenerative diseases and psychiatric troubles, LR18SP03, Razi Hospital, University of Tunis El Manar, Tunis, Tunisia. 3- Ministry of Health, National Observatory of New and Emerging Diseases, 1006, Tunis, Tunisia	Ilhem Boutiba-Ben Boubaker, Sameh Trabelsi, Nissaf Ben Alaya, Maher Kharrat, Alia Ben Kahla, Jalila Ben Khelil, Salma Abid, Sana Ferjani, Mouna Ben Sassi, Mouna Safer, Imen Mkada, Imen Kacem, Gaies Emna, Soumaya Rammeh, Riadh Daghfous, Riadh Gouider.
EPI_ISL_635063, EPI_ISL_635064, EPI_ISL_635065	Foerde Hospital, Department of Microbiology	Norwegian Institute of Public Health, Department of Virology	Kathrine Stene-Johansen, Kamilla Heddeland Instefjord, Hilde Elshaug, Marie Paulsen Madsen, Rasmus Riis Kopperud, Hilde Vollan, Karoline Bragstad, Olav Hungnes
EPI_ISL_635066, EPI_ISL_635067	Norwegian Institute of Public Health, Department of Virology	Norwegian Institute of Public Health, Department of Virology	Kathrine Stene-Johansen, Kamilla Heddeland Instefjord, Hilde Elshaug, Marie Paulsen Madsen, Rasmus Riis Kopperud, Hilde Vollan, Karoline Bragstad, Olav Hungnes
EPI_ISL_635068, EPI_ISL_635069, EPI_ISL_635070, EPI_ISL_635071, EPI_ISL_635072, EPI_ISL_635073	University Hospital of Northern Norway, Department for Microbiology and Infectious Disease Control	Norwegian Institute of Public Health, Department of Virology	Kathrine Stene-Johansen, Kamilla Heddeland Instefjord, Hilde Elshaug, Marie Paulsen Madsen, Rasmus Riis Kopperud, Hilde Vollan, Karoline Bragstad, Olav Hungnes
EPI_ISL_635074, EPI_ISL_635075, EPI_ISL_635076, EPI_ISL_635077, EPI_ISL_635078, EPI_ISL_635079	Norwegian Institute of Public Health, Department of Virology	Norwegian Institute of Public Health, Department of Virology	Kathrine Stene-Johansen, Kamilla Heddeland Instefjord, Hilde Elshaug, Marie Paulsen Madsen, Rasmus Riis Kopperud, Hilde Vollan, Karoline Bragstad, Olav Hungnes
EPI_ISL_635081	Haukeland University Hospital, Department of Medical Microbiology	Norwegian Institute of Public Health, Department of Virology	Kathrine Stene-Johansen, Kamilla Heddeland Instefjord, Hilde Elshaug, Marie Paulsen Madsen, Rasmus Riis Kopperud, Hilde Vollan, Karoline Bragstad, Olav Hungnes
EPI_ISL_635085	Oslo University Hospital, Department of Medical Microbiology	Norwegian Institute of Public Health, Department of Virology	Kathrine Stene-Johansen, Kamilla Heddeland Instefjord, Hilde Elshaug, Marie Paulsen Madsen, Rasmus Riis Kopperud, Hilde Vollan, Karoline Bragstad, Olav Hungnes
EPI_ISL_635086, EPI_ISL_635087	Furst Medical Laboratory	Norwegian Institute of Public Health, Department of Virology	Kathrine Stene-Johansen, Kamilla Heddeland Instefjord, Hilde Elshaug, Marie Paulsen Madsen, Rasmus Riis Kopperud, Hilde Vollan, Karoline Bragstad, Olav Hungnes

EPI_ISL_635095	Oslo University Hospital, Department of Medical Microbiology	Norwegian Institute of Public Health, Department of Virology	Kathrine Stene-Johansen, Kamilla Heddeland Instefjord, Hilde Elshaug, Marie Paulsen Madsen, Rasmus Riis Kopperud, Hilde Vollan, Karoline Bragstad, Olav Hungnes
EPI_ISL_635096	Department of Medical Microbiology, St. Olavs hospital	Norwegian Institute of Public Health, Department of Virology	Kathrine Stene-Johansen, Kamilla Heddeland Instefjord, Hilde Elshaug, Marie Paulsen Madsen, Rasmus Riis Kopperud, Hilde Vollan, Karoline Bragstad, Olav Hungnes
EPI_ISL_635097	Hospital of Southern Norway - Kristiansand, Department of Medical Microbiology	Norwegian Institute of Public Health, Department of Virology	Kathrine Stene-Johansen, Kamilla Heddeland Instefjord, Hilde Elshaug, Marie Paulsen Madsen, Rasmus Riis Kopperud, Hilde Vollan, Karoline Bragstad, Olav Hungnes
EPI_ISL_635098	Foerde Hospital, Department of Microbiology	Norwegian Institute of Public Health, Department of Virology	Kathrine Stene-Johansen, Kamilla Heddeland Instefjord, Hilde Elshaug, Marie Paulsen Madsen, Rasmus Riis Kopperud, Hilde Vollan, Karoline Bragstad, Olav Hungnes
EPI_ISL_635099	Medical Microbiology Unit, Department for Laboratory Medicine, Drammen Hospital, Vestre Viken Health Trust,	Norwegian Institute of Public Health, Department of Virology	Kathrine Stene-Johansen, Kamilla Heddeland Instefjord, Hilde Elshaug, Marie Paulsen Madsen, Rasmus Riis Kopperud, Hilde Vollan, Karoline Bragstad, Olav Hungnes
EPI_ISL_635100	Department of Medical Microbiology, St. Olavs hospital	Norwegian Institute of Public Health, Department of Virology	Kathrine Stene-Johansen, Kamilla Heddeland Instefjord, Hilde Elshaug, Marie Paulsen Madsen, Rasmus Riis Kopperud, Hilde Vollan, Karoline Bragstad, Olav Hungnes
EPI_ISL_635101	Medical Microbiology Unit, Department for Laboratory Medicine, Drammen Hospital, Vestre Viken Health Trust,	Norwegian Institute of Public Health, Department of Virology	Kathrine Stene-Johansen, Kamilla Heddeland Instefjord, Hilde Elshaug, Marie Paulsen Madsen, Rasmus Riis Kopperud, Hilde Vollan, Karoline Bragstad, Olav Hungnes
EPI_ISL_635102	Akershus University Hospital, Department for Microbiology and Infectious Disease Control	Norwegian Institute of Public Health, Department of Virology	Kathrine Stene-Johansen, Kamilla Heddeland Instefjord, Hilde Elshaug, Marie Paulsen Madsen, Rasmus Riis Kopperud, Hilde Vollan, Karoline Bragstad, Olav Hungnes
EPI_ISL_635103	Vestfold Hospital, Toensberg Department of Microbiology	Norwegian Institute of Public Health, Department of Virology	Kathrine Stene-Johansen, Kamilla Heddeland Instefjord, Hilde Elshaug, Marie Paulsen Madsen, Rasmus Riis Kopperud, Hilde Vollan, Karoline Bragstad, Olav Hungnes
EPI_ISL_635104	Unilabs Laboratory Medicine	Norwegian Institute of Public Health, Department of Virology	Kathrine Stene-Johansen, Kamilla Heddeland Instefjord, Hilde Elshaug, Marie Paulsen Madsen, Rasmus Riis Kopperud, Hilde Vollan, Karoline Bragstad, Olav Hungnes
EPI_ISL_635105, EPI_ISL_635106	Vestfold Hospital, Toensberg Department of Microbiology	Norwegian Institute of Public Health, Department of Virology	Kathrine Stene-Johansen, Kamilla Heddeland Instefjord, Hilde Elshaug, Marie Paulsen Madsen, Rasmus Riis Kopperud, Hilde Vollan, Karoline Bragstad, Olav Hungnes
EPI_ISL_635107, EPI_ISL_635108, EPI_ISL_635109	Ostfold Hospital Trust - Kalnes, Centre for Laboratory Medicine, Section for gene technology and infection serology	Norwegian Institute of Public Health, Department of Virology	Kathrine Stene-Johansen, Kamilla Heddeland Instefjord, Hilde Elshaug, Marie Paulsen Madsen, Rasmus Riis Kopperud, Hilde Vollan, Karoline Bragstad, Olav Hungnes
EPI_ISL_635110, EPI_ISL_635111	Department of Medical Microbiology, St. Olavs hospital	Norwegian Institute of Public Health, Department of Virology	Kathrine Stene-Johansen, Kamilla Heddeland Instefjord, Hilde Elshaug, Marie Paulsen Madsen, Rasmus Riis Kopperud, Hilde Vollan, Karoline Bragstad, Olav Hungnes
EPI_ISL_635112	Vestfold Hospital, Toensberg Department of Microbiology	Norwegian Institute of Public Health, Department of Virology	Kathrine Stene-Johansen, Kamilla Heddeland Instefjord, Hilde Elshaug, Marie Paulsen Madsen, Rasmus Riis Kopperud, Hilde Vollan, Karoline Bragstad, Olav Hungnes
EPI_ISL_635113, EPI_ISL_635114, EPI_ISL_635115	University Hospital of Northern Norway, Department for Microbiology and Infectious Disease Control	Norwegian Institute of Public Health, Department of Virology	Kathrine Stene-Johansen, Kamilla Heddeland Instefjord, Hilde Elshaug, Marie Paulsen Madsen, Rasmus Riis Kopperud, Hilde Vollan, Karoline Bragstad, Olav Hungnes
EPI_ISL_635116	Ostfold Hospital Trust - Kalnes, Centre for Laboratory Medicine, Section for gene technology and infection serology	Norwegian Institute of Public Health, Department of Virology	Kathrine Stene-Johansen, Kamilla Heddeland Instefjord, Hilde Elshaug, Marie Paulsen Madsen, Rasmus Riis Kopperud, Hilde Vollan, Karoline Bragstad, Olav Hungnes
EPI_ISL_635117, EPI_ISL_635118	University Hospital of Northern Norway, Department for Microbiology and Infectious Disease Control	Norwegian Institute of Public Health, Department of Virology	Kathrine Stene-Johansen, Kamilla Heddeland Instefjord, Hilde Elshaug, Marie Paulsen Madsen, Rasmus Riis Kopperud, Hilde Vollan, Karoline Bragstad, Olav Hungnes
EPI_ISL_635119	Oslo University Hospital, Department of Medical Microbiology	Norwegian Institute of Public Health, Department of Virology	Kathrine Stene-Johansen, Kamilla Heddeland Instefjord, Hilde Elshaug, Marie Paulsen Madsen, Rasmus Riis Kopperud, Hilde Vollan, Karoline Bragstad, Olav Hungnes
EPI_ISL_635120	Dept. of Medical Microbiology, Stavanger University Hospital, Helse Stavanger HF	Norwegian Institute of Public Health, Department of Virology	Kathrine Stene-Johansen, Kamilla Heddeland Instefjord, Hilde Elshaug, Marie Paulsen Madsen, Rasmus Riis Kopperud, Hilde Vollan, Karoline Bragstad, Olav Hungnes
EPI_ISL_635121, EPI_ISL_635122, EPI_ISL_635123	Hospital of Southern Norway - Kristiansand, Department of Medical Microbiology	Norwegian Institute of Public Health, Department of Virology	Kathrine Stene-Johansen, Kamilla Heddeland Instefjord, Hilde Elshaug, Marie Paulsen Madsen, Rasmus Riis Kopperud, Hilde Vollan, Karoline Bragstad, Olav Hungnes
EPI_ISL_635124, EPI_ISL_635125	Innlandet Hospital Trust, Division Lillehammer, Department for Medical Microbiology	Norwegian Institute of Public Health, Department of Virology	Kathrine Stene-Johansen, Kamilla Heddeland Instefjord, Hilde Elshaug, Marie Paulsen Madsen, Rasmus Riis Kopperud, Hilde Vollan, Karoline Bragstad, Olav Hungnes
EPI_ISL_635126	Department of Medical Microbiology, St. Olavs hospital	Norwegian Institute of Public Health, Department of Virology	Kathrine Stene-Johansen, Kamilla Heddeland Instefjord, Hilde Elshaug, Marie Paulsen Madsen, Rasmus Riis Kopperud, Hilde Vollan, Karoline Bragstad, Olav Hungnes
EPI_ISL_635127	Foerde Hospital, Department of Microbiology	Norwegian Institute of Public Health, Department of Virology	Kathrine Stene-Johansen, Kamilla Heddeland Instefjord, Hilde Elshaug, Marie Paulsen Madsen, Rasmus Riis Kopperud, Hilde Vollan, Karoline Bragstad, Olav Hungnes
EPI_ISL_635128	Haukeland University Hospital, Dept. of Microbiology	Norwegian Institute of Public Health, Department of Virology	Kathrine Stene-Johansen, Kamilla Heddeland Instefjord, Hilde Elshaug, Marie Paulsen Madsen, Rasmus Riis Kopperud, Hilde Vollan, Karoline Bragstad, Olav Hungnes
EPI_ISL_635129	Oslo University Hospital, Department of Medical Microbiology	Norwegian Institute of Public Health, Department of Virology	Kathrine Stene-Johansen, Kamilla Heddeland Instefjord, Hilde Elshaug, Marie Paulsen Madsen, Rasmus Riis Kopperud, Hilde Vollan, Karoline Bragstad, Olav Hungnes
EPI_ISL_635130, EPI_ISL_635131	Innlandet Hospital Trust, Division Lillehammer, Department for Medical Microbiology	Norwegian Institute of Public Health, Department of Virology	Kathrine Stene-Johansen, Kamilla Heddeland Instefjord, Hilde Elshaug, Marie Paulsen Madsen, Rasmus Riis Kopperud, Hilde Vollan, Karoline Bragstad, Olav Hungnes
EPI_ISL_635132	Unilabs Laboratory Medicine	Norwegian Institute of Public Health, Department of Virology	Kathrine Stene-Johansen, Kamilla Heddeland Instefjord, Hilde Elshaug, Marie Paulsen Madsen, Rasmus Riis Kopperud, Hilde Vollan, Karoline Bragstad, Olav Hungnes
EPI_ISL_635133	Haukeland University Hospital, Department of Medical Microbiology	Norwegian Institute of Public Health, Department of Virology	Kathrine Stene-Johansen, Kamilla Heddeland Instefjord, Hilde Elshaug, Marie Paulsen Madsen, Rasmus Riis Kopperud, Hilde Vollan, Karoline Bragstad, Olav Hungnes
EPI_ISL_635134	Medical Microbiology Unit, Department for Laboratory Medicine, Drammen Hospital, Vestre Viken Health Trust,	Norwegian Institute of Public Health, Department of Virology	Kathrine Stene-Johansen, Kamilla Heddeland Instefjord, Hilde Elshaug, Marie Paulsen Madsen, Rasmus Riis Kopperud, Hilde Vollan, Karoline Bragstad, Olav Hungnes
EPI_ISL_635135, EPI_ISL_635136	Department of Medical Microbiology, St. Olavs hospital	Norwegian Institute of Public Health, Department of Virology	Kathrine Stene-Johansen, Kamilla Heddeland Instefjord, Hilde Elshaug, Marie Paulsen Madsen, Rasmus Riis Kopperud, Hilde Vollan, Karoline Bragstad, Olav Hungnes
EPI_ISL_635137	University Hospital of Northern Norway, Department for Microbiology and Infectious Disease Control	Norwegian Institute of Public Health, Department of Virology	Kathrine Stene-Johansen, Kamilla Heddeland Instefjord, Hilde Elshaug, Marie Paulsen Madsen, Rasmus Riis Kopperud, Hilde Vollan, Karoline Bragstad, Olav Hungnes
EPI_ISL_635138	Hospital of Southern Norway - Kristiansand, Department of Medical Microbiology	Norwegian Institute of Public Health, Department of Virology	Kathrine Stene-Johansen, Kamilla Heddeland Instefjord, Hilde Elshaug, Marie Paulsen Madsen, Rasmus Riis Kopperud, Hilde Vollan, Karoline Bragstad, Olav Hungnes
EPI_ISL_635139, EPI_ISL_635140	Vestfold Hospital, Toensberg Department of Microbiology	Norwegian Institute of Public Health, Department of Virology	Kathrine Stene-Johansen, Kamilla Heddeland Instefjord, Hilde Elshaug, Marie Paulsen Madsen, Rasmus Riis Kopperud, Hilde Vollan, Karoline Bragstad, Olav Hungnes

EPI_ISL_635141	University Hospital of Northern Norway, Department for Microbiology and Infectious Disease Control	Norwegian Institute of Public Health, Department of Virology	Kathrine Stene-Johansen, Kamilla Heddeland Instefjord, Hilde Elshaug, Marie Paulsen Madsen, Rasmus Riis Kopperud, Hilde Vollan, Karoline Bragstad, Olav Hungnes
EPI_ISL_635142	Nordland Hospital - Bodo, Laboratory Department, Molecular Biology Unit	Norwegian Institute of Public Health, Department of Virology	Kathrine Stene-Johansen, Kamilla Heddeland Instefjord, Hilde Elshaug, Marie Paulsen Madsen, Rasmus Riis Kopperud, Hilde Vollan, Karoline Bragstad, Olav Hungnes
EPI_ISL_635143	Hospital of Southern Norway - Kristiansand, Department of Medical Microbiology	Norwegian Institute of Public Health, Department of Virology	Kathrine Stene-Johansen, Kamilla Heddeland Instefjord, Hilde Elshaug, Marie Paulsen Madsen, Rasmus Riis Kopperud, Hilde Vollan, Karoline Bragstad, Olav Hungnes
EPI_ISL_635144	Dept. of Medical Microbiology, Stavanger University Hospital, Helse Stavanger HF	Norwegian Institute of Public Health, Department of Virology	Kathrine Stene-Johansen, Kamilla Heddeland Instefjord, Hilde Elshaug, Marie Paulsen Madsen, Rasmus Riis Kopperud, Hilde Vollan, Karoline Bragstad, Olav Hungnes
EPI_ISL_635145	Nordland Hospital - Bodo, Laboratory Department, Molecular Biology Unit	Norwegian Institute of Public Health, Department of Virology	Kathrine Stene-Johansen, Kamilla Heddeland Instefjord, Hilde Elshaug, Marie Paulsen Madsen, Rasmus Riis Kopperud, Hilde Vollan, Karoline Bragstad, Olav Hungnes
EPI_ISL_635146	Dept. of Medical Microbiology, Stavanger University Hospital, Helse Stavanger HF	Norwegian Institute of Public Health, Department of Virology	Kathrine Stene-Johansen, Kamilla Heddeland Instefjord, Hilde Elshaug, Marie Paulsen Madsen, Rasmus Riis Kopperud, Hilde Vollan, Karoline Bragstad, Olav Hungnes
EPI_ISL_635147, EPI_ISL_635148	University Hospital of Northern Norway, Department for Microbiology and Infectious Disease Control	Norwegian Institute of Public Health, Department of Virology	Kathrine Stene-Johansen, Kamilla Heddeland Instefjord, Hilde Elshaug, Marie Paulsen Madsen, Rasmus Riis Kopperud, Hilde Vollan, Karoline Bragstad, Olav Hungnes
EPI_ISL_635149, EPI_ISL_635150, EPI_ISL_635151, EPI_ISL_635152	Ostfold Hospital Trust - Kalnes, Centre for Laboratory Medicine, Section for gene technology and infection serology	Norwegian Institute of Public Health, Department of Virology	Kathrine Stene-Johansen, Kamilla Heddeland Instefjord, Hilde Elshaug, Marie Paulsen Madsen, Rasmus Riis Kopperud, Hilde Vollan, Karoline Bragstad, Olav Hungnes
EPI_ISL_635153, EPI_ISL_635154	Unilabs Laboratory Medicine	Norwegian Institute of Public Health, Department of Virology	Kathrine Stene-Johansen, Kamilla Heddeland Instefjord, Hilde Elshaug, Marie Paulsen Madsen, Rasmus Riis Kopperud, Hilde Vollan, Karoline Bragstad, Olav Hungnes
EPI_ISL_635155	Oslo University Hospital, Department of Medical Microbiology	Norwegian Institute of Public Health, Department of Virology	Kathrine Stene-Johansen, Kamilla Heddeland Instefjord, Hilde Elshaug, Marie Paulsen Madsen, Rasmus Riis Kopperud, Hilde Vollan, Karoline Bragstad, Olav Hungnes
EPI_ISL_635156, EPI_ISL_635157	University Hospital of Northern Norway, Department for Microbiology and Infectious Disease Control	Norwegian Institute of Public Health, Department of Virology	Kathrine Stene-Johansen, Kamilla Heddeland Instefjord, Hilde Elshaug, Marie Paulsen Madsen, Rasmus Riis Kopperud, Hilde Vollan, Karoline Bragstad, Olav Hungnes
EPI_ISL_635158	Unilabs Laboratory Medicine	Norwegian Institute of Public Health, Department of Virology	Kathrine Stene-Johansen, Kamilla Heddeland Instefjord, Hilde Elshaug, Marie Paulsen Madsen, Rasmus Riis Kopperud, Hilde Vollan, Karoline Bragstad, Olav Hungnes
EPI_ISL_635159	Medical Microbiology Unit, Department for Laboratory Medicine, Drammen Hospital, Vestre Viken Health Trust,	Norwegian Institute of Public Health, Department of Virology	Kathrine Stene-Johansen, Kamilla Heddeland Instefjord, Hilde Elshaug, Marie Paulsen Madsen, Rasmus Riis Kopperud, Hilde Vollan, Karoline Bragstad, Olav Hungnes
EPI_ISL_635160	University Hospital of Northern Norway, Department for Microbiology and Infectious Disease Control	Norwegian Institute of Public Health, Department of Virology	Kathrine Stene-Johansen, Kamilla Heddeland Instefjord, Hilde Elshaug, Marie Paulsen Madsen, Rasmus Riis Kopperud, Hilde Vollan, Karoline Bragstad, Olav Hungnes
EPI_ISL_635161	Hospital of Southern Norway - Kristiansand, Department of Medical Microbiology	Norwegian Institute of Public Health, Department of Virology	Kathrine Stene-Johansen, Kamilla Heddeland Instefjord, Hilde Elshaug, Marie Paulsen Madsen, Rasmus Riis Kopperud, Hilde Vollan, Karoline Bragstad, Olav Hungnes
EPI_ISL_635162	Unilabs Laboratory Medicine	Norwegian Institute of Public Health, Department of Virology	Kathrine Stene-Johansen, Kamilla Heddeland Instefjord, Hilde Elshaug, Marie Paulsen Madsen, Rasmus Riis Kopperud, Hilde Vollan, Karoline Bragstad, Olav Hungnes
EPI_ISL_635163	University Hospital of Northern Norway, Department for Microbiology and Infectious Disease Control	Norwegian Institute of Public Health, Department of Virology	Kathrine Stene-Johansen, Kamilla Heddeland Instefjord, Hilde Elshaug, Marie Paulsen Madsen, Rasmus Riis Kopperud, Hilde Vollan, Karoline Bragstad, Olav Hungnes
EPI_ISL_635164	Ostfold Hospital Trust - Kalnes, Centre for Laboratory Medicine, Section for gene technology and infection serology	Norwegian Institute of Public Health, Department of Virology	Kathrine Stene-Johansen, Kamilla Heddeland Instefjord, Hilde Elshaug, Marie Paulsen Madsen, Rasmus Riis Kopperud, Hilde Vollan, Karoline Bragstad, Olav Hungnes
EPI_ISL_635165	Department of Medical Microbiology, St. Olavs hospital	Norwegian Institute of Public Health, Department of Virology	Kathrine Stene-Johansen, Kamilla Heddeland Instefjord, Hilde Elshaug, Marie Paulsen Madsen, Rasmus Riis Kopperud, Hilde Vollan, Karoline Bragstad, Olav Hungnes
EPI_ISL_635166	Dept. of Medical Microbiology, Stavanger University Hospital, Helse Stavanger HF	Norwegian Institute of Public Health, Department of Virology	Kathrine Stene-Johansen, Kamilla Heddeland Instefjord, Hilde Elshaug, Marie Paulsen Madsen, Rasmus Riis Kopperud, Hilde Vollan, Karoline Bragstad, Olav Hungnes
EPI_ISL_635167	Foerde Hospital, Department of Microbiology	Norwegian Institute of Public Health, Department of Virology	Kathrine Stene-Johansen, Kamilla Heddeland Instefjord, Hilde Elshaug, Marie Paulsen Madsen, Rasmus Riis Kopperud, Hilde Vollan, Karoline Bragstad, Olav Hungnes
EPI_ISL_635168	Vestfold Hospital, Toensberg Department of Microbiology	Norwegian Institute of Public Health, Department of Virology	Kathrine Stene-Johansen, Kamilla Heddeland Instefjord, Hilde Elshaug, Marie Paulsen Madsen, Rasmus Riis Kopperud, Hilde Vollan, Karoline Bragstad, Olav Hungnes
EPI_ISL_635169	Medical Microbiology Unit, Department for Laboratory Medicine, Drammen Hospital, Vestre Viken Health Trust,	Norwegian Institute of Public Health, Department of Virology	Kathrine Stene-Johansen, Kamilla Heddeland Instefjord, Hilde Elshaug, Marie Paulsen Madsen, Rasmus Riis Kopperud, Hilde Vollan, Karoline Bragstad, Olav Hungnes
EPI_ISL_635170	Foerde Hospital, Department of Microbiology	Norwegian Institute of Public Health, Department of Virology	Kathrine Stene-Johansen, Kamilla Heddeland Instefjord, Hilde Elshaug, Marie Paulsen Madsen, Rasmus Riis Kopperud, Hilde Vollan, Karoline Bragstad, Olav Hungnes
EPI_ISL_635171	Vestfold Hospital, Toensberg Department of Microbiology	Norwegian Institute of Public Health, Department of Virology	Kathrine Stene-Johansen, Kamilla Heddeland Instefjord, Hilde Elshaug, Marie Paulsen Madsen, Rasmus Riis Kopperud, Hilde Vollan, Karoline Bragstad, Olav Hungnes
EPI_ISL_635172	Oslo University Hospital, Department of Medical Microbiology	Norwegian Institute of Public Health, Department of Virology	Kathrine Stene-Johansen, Kamilla Heddeland Instefjord, Hilde Elshaug, Marie Paulsen Madsen, Rasmus Riis Kopperud, Hilde Vollan, Karoline Bragstad, Olav Hungnes
EPI_ISL_635173	Foerde Hospital, Department of Microbiology	Norwegian Institute of Public Health, Department of Virology	Kathrine Stene-Johansen, Kamilla Heddeland Instefjord, Hilde Elshaug, Marie Paulsen Madsen, Rasmus Riis Kopperud, Hilde Vollan, Karoline Bragstad, Olav Hungnes
EPI_ISL_635174, EPI_ISL_635175	Ostfold Hospital Trust - Kalnes, Centre for Laboratory Medicine, Section for gene technology and infection serology	Norwegian Institute of Public Health, Department of Virology	Kathrine Stene-Johansen, Kamilla Heddeland Instefjord, Hilde Elshaug, Marie Paulsen Madsen, Rasmus Riis Kopperud, Hilde Vollan, Karoline Bragstad, Olav Hungnes
EPI_ISL_635176, EPI_ISL_635177	Foerde Hospital, Department of Microbiology	Norwegian Institute of Public Health, Department of Virology	Kathrine Stene-Johansen, Kamilla Heddeland Instefjord, Hilde Elshaug, Marie Paulsen Madsen, Rasmus Riis Kopperud, Hilde Vollan, Karoline Bragstad, Olav Hungnes
EPI_ISL_635178	Norwegian Institute of Public Health, Department of Virology	Norwegian Institute of Public Health, Department of Virology	Kathrine Stene-Johansen, Kamilla Heddeland Instefjord, Hilde Elshaug, Marie Paulsen Madsen, Rasmus Riis Kopperud, Hilde Vollan, Karoline Bragstad, Olav Hungnes
EPI_ISL_635179, EPI_ISL_635180, EPI_ISL_635181	University Hospital of Northern Norway, Department for Microbiology and Infectious Disease Control	Norwegian Institute of Public Health, Department of Virology	Kathrine Stene-Johansen, Kamilla Heddeland Instefjord, Hilde Elshaug, Marie Paulsen Madsen, Rasmus Riis Kopperud, Hilde Vollan, Karoline Bragstad, Olav Hungnes
EPI_ISL_635182	Nordland Hospital - Bodo, Laboratory Department, Molecular Biology Unit	Norwegian Institute of Public Health, Department of Virology	Kathrine Stene-Johansen, Kamilla Heddeland Instefjord, Hilde Elshaug, Marie Paulsen Madsen, Rasmus Riis Kopperud, Hilde Vollan, Karoline Bragstad, Olav Hungnes
EPI_ISL_635183	Innlandet Hospital Trust, Division Lillehammer, Department for Medical Microbiology	Norwegian Institute of Public Health, Department of Virology	Kathrine Stene-Johansen, Kamilla Heddeland Instefjord, Hilde Elshaug, Marie Paulsen Madsen, Rasmus Riis Kopperud, Hilde Vollan, Karoline Bragstad, Olav Hungnes

EPI_ISL_635184	Haukeland University Hospital, Department of Medical Microbiology	Norwegian Institute of Public Health, Department of Virology	Kathrine Stene-Johansen, Kamilla Heddeland Instefjord, Hilde Elshaug, Marie Paulsen Madsen, Rasmus Riis Kopperud, Hilde Vollan, Karoline Bragstad, Olav Hungnes
EPI_ISL_635185	Ostfold Hospital Trust - Kalnes, Centre for Laboratory Medicine, Section for gene technology and infection serology	Norwegian Institute of Public Health, Department of Virology	Kathrine Stene-Johansen, Kamilla Heddeland Instefjord, Hilde Elshaug, Marie Paulsen Madsen, Rasmus Riis Kopperud, Hilde Vollan, Karoline Bragstad, Olav Hungnes
EPI_ISL_635186	University Hospital of Northern Norway, Department for Microbiology and Infectious Disease Control	Norwegian Institute of Public Health, Department of Virology	Kathrine Stene-Johansen, Kamilla Heddeland Instefjord, Hilde Elshaug, Marie Paulsen Madsen, Rasmus Riis Kopperud, Hilde Vollan, Karoline Bragstad, Olav Hungnes
EPI_ISL_635187, EPI_ISL_635188	Norwegian Institute of Public Health, Department of Virology	Norwegian Institute of Public Health, Department of Virology	Kathrine Stene-Johansen, Kamilla Heddeland Instefjord, Hilde Elshaug, Marie Paulsen Madsen, Rasmus Riis Kopperud, Hilde Vollan, Karoline Bragstad, Olav Hungnes
EPI_ISL_635189	Hospital of Southern Norway - Kristiansand, Department of Medical Microbiology	Norwegian Institute of Public Health, Department of Virology	Kathrine Stene-Johansen, Kamilla Heddeland Instefjord, Hilde Elshaug, Marie Paulsen Madsen, Rasmus Riis Kopperud, Hilde Vollan, Karoline Bragstad, Olav Hungnes
EPI_ISL_635190	University Hospital of Northern Norway, Department for Microbiology and Infectious Disease Control	Norwegian Institute of Public Health, Department of Virology	Kathrine Stene-Johansen, Kamilla Heddeland Instefjord, Hilde Elshaug, Marie Paulsen Madsen, Rasmus Riis Kopperud, Hilde Vollan, Karoline Bragstad, Olav Hungnes
EPI_ISL_635191	Unilabs Laboratory Medicine	Norwegian Institute of Public Health, Department of Virology	Kathrine Stene-Johansen, Kamilla Heddeland Instefjord, Hilde Elshaug, Marie Paulsen Madsen, Rasmus Riis Kopperud, Hilde Vollan, Karoline Bragstad, Olav Hungnes
EPI_ISL_635192, EPI_ISL_635193	Medical Microbiology Unit, Department for Laboratory Medicine, Drammen Hospital, Vestre Viken Health Trust,	Norwegian Institute of Public Health, Department of Virology	Kathrine Stene-Johansen, Kamilla Heddeland Instefjord, Hilde Elshaug, Marie Paulsen Madsen, Rasmus Riis Kopperud, Hilde Vollan, Karoline Bragstad, Olav Hungnes
EPI_ISL_635194	Akershus University Hospital, Department for Microbiology and Infectious Disease Control	Norwegian Institute of Public Health, Department of Virology	Kathrine Stene-Johansen, Kamilla Heddeland Instefjord, Hilde Elshaug, Marie Paulsen Madsen, Rasmus Riis Kopperud, Hilde Vollan, Karoline Bragstad, Olav Hungnes
EPI_ISL_635195	Vestfold Hospital, Toensberg Department of Microbiology	Norwegian Institute of Public Health, Department of Virology	Kathrine Stene-Johansen, Kamilla Heddeland Instefjord, Hilde Elshaug, Marie Paulsen Madsen, Rasmus Riis Kopperud, Hilde Vollan, Karoline Bragstad, Olav Hungnes
EPI_ISL_635196	Dept. of Medical Microbiology, Stavanger University Hospital, Helse Stavanger HF	Norwegian Institute of Public Health, Department of Virology	Kathrine Stene-Johansen, Kamilla Heddeland Instefjord, Hilde Elshaug, Marie Paulsen Madsen, Rasmus Riis Kopperud, Hilde Vollan, Karoline Bragstad, Olav Hungnes
EPI_ISL_635197	Oslo University Hospital, Department of Medical Microbiology	Norwegian Institute of Public Health, Department of Virology	Kathrine Stene-Johansen, Kamilla Heddeland Instefjord, Hilde Elshaug, Marie Paulsen Madsen, Rasmus Riis Kopperud, Hilde Vollan, Karoline Bragstad, Olav Hungnes
EPI_ISL_635198	University Hospital of Northern Norway, Department for Microbiology and Infectious Disease Control	Norwegian Institute of Public Health, Department of Virology	Kathrine Stene-Johansen, Kamilla Heddeland Instefjord, Hilde Elshaug, Marie Paulsen Madsen, Rasmus Riis Kopperud, Hilde Vollan, Karoline Bragstad, Olav Hungnes
EPI_ISL_635201, EPI_ISL_635206, EPI_ISL_635207, EPI_ISL_635208, EPI_ISL_635209, EPI_ISL_635210, EPI_ISL_635211, EPI_ISL_635212, EPI_ISL_635213, EPI_ISL_635214, EPI_ISL_635215, EPI_ISL_635216, EPI_ISL_635217, EPI_ISL_635218, EPI_ISL_635219, EPI_ISL_635220, EPI_ISL_635221, EPI_ISL_635222, EPI_ISL_635223, EPI_ISL_635224, EPI_ISL_635225, EPI_ISL_635226, EPI_ISL_635227, EPI_ISL_635228, EPI_ISL_635229, EPI_ISL_635230, EPI_ISL_635231, EPI_ISL_635232, EPI_ISL_635233, EPI_ISL_635234, EPI_ISL_635235, EPI_ISL_635236, EPI_ISL_635237, EPI_ISL_635238, EPI_ISL_635239, EPI_ISL_635240, EPI_ISL_635241, EPI_ISL_635242, EPI_ISL_635243, EPI_ISL_635244, EPI_ISL_635245, EPI_ISL_635246, EPI_ISL_635248			
see above	Institute of Microbiology and Immunology, Faculty of Medicine, University of Ljubljana	Institute of Microbiology and Immunology, Faculty of Medicine, University of Ljubljana	Samo Zakotnik, Tomaž Mark Zorec, Miša Korva, Mario Poljak, Tatjana Avši - Županc
EPI_ISL_635250, EPI_ISL_635251, EPI_ISL_635252, EPI_ISL_635253, EPI_ISL_635254, EPI_ISL_635255, EPI_ISL_635256, EPI_ISL_635257, EPI_ISL_635258, EPI_ISL_635259, EPI_ISL_635260, EPI_ISL_635261, EPI_ISL_635262, EPI_ISL_635263, EPI_ISL_635264, EPI_ISL_635265, EPI_ISL_635266, EPI_ISL_635267, EPI_ISL_635269, EPI_ISL_635270, EPI_ISL_635271, EPI_ISL_635272, EPI_ISL_635274, EPI_ISL_635275, EPI_ISL_635276, EPI_ISL_635277, EPI_ISL_635278, EPI_ISL_635281, EPI_ISL_635282, EPI_ISL_635283, EPI_ISL_635284, EPI_ISL_635285, EPI_ISL_635286, EPI_ISL_635287, EPI_ISL_635288, EPI_ISL_635289, EPI_ISL_635290, EPI_ISL_635291, EPI_ISL_635292, EPI_ISL_635293, EPI_ISL_635294, EPI_ISL_635295, EPI_ISL_635296, EPI_ISL_635297, EPI_ISL_635299			
see above	Institute of Microbiology and Immunology, Faculty of Medicine, University of Ljubljana	Institute of Microbiology and Immunology, Faculty of Medicine, University of Ljubljana	Tomaž Mark Zorec, Samo Zakotnik, Miša Korva, Tatjana Avši - Županc, Mario Poljak
EPI_ISL_635300, EPI_ISL_635301, EPI_ISL_635302, EPI_ISL_635303, EPI_ISL_635304, EPI_ISL_635305, EPI_ISL_635306, EPI_ISL_635308, EPI_ISL_635309, EPI_ISL_635310, EPI_ISL_635311, EPI_ISL_635312, EPI_ISL_635313, EPI_ISL_635314, EPI_ISL_635315, EPI_ISL_635316, EPI_ISL_635317, EPI_ISL_635318, EPI_ISL_635319, EPI_ISL_635320, EPI_ISL_635321, EPI_ISL_635322, EPI_ISL_635323, EPI_ISL_635324, EPI_ISL_635325, EPI_ISL_635326, EPI_ISL_635327, EPI_ISL_635328, EPI_ISL_635329, EPI_ISL_635330, EPI_ISL_635331, EPI_ISL_635332, EPI_ISL_635333, EPI_ISL_635334, EPI_ISL_635335, EPI_ISL_635336, EPI_ISL_635337, EPI_ISL_635338, EPI_ISL_635340, EPI_ISL_635341, EPI_ISL_635342, EPI_ISL_635343, EPI_ISL_635344, EPI_ISL_635345, EPI_ISL_635346, EPI_ISL_635347, EPI_ISL_635348, EPI_ISL_635349, EPI_ISL_635350, EPI_ISL_635352, EPI_ISL_635353, EPI_ISL_635354, EPI_ISL_635355, EPI_ISL_635356, EPI_ISL_635357, EPI_ISL_635358, EPI_ISL_635359, EPI_ISL_635360, EPI_ISL_635361, EPI_ISL_635363, EPI_ISL_635364, EPI_ISL_635366, EPI_ISL_635369, EPI_ISL_635370, EPI_ISL_635371, EPI_ISL_635372, EPI_ISL_635373, EPI_ISL_635374, EPI_ISL_635375, EPI_ISL_635376, EPI_ISL_635377, EPI_ISL_635378, EPI_ISL_635379, EPI_ISL_635380, EPI_ISL_635381, EPI_ISL_635382, EPI_ISL_635383, EPI_ISL_635385, EPI_ISL_635386, EPI_ISL_635387, EPI_ISL_635388, EPI_ISL_635389, EPI_ISL_635390, EPI_ISL_635391, EPI_ISL_635392, EPI_ISL_635393, EPI_ISL_635394, EPI_ISL_635395, EPI_ISL_635396, EPI_ISL_635397, EPI_ISL_635398, EPI_ISL_635399, EPI_ISL_635401, EPI_ISL_635402, EPI_ISL_635403, EPI_ISL_635404, EPI_ISL_635405, EPI_ISL_635406, EPI_ISL_635407, EPI_ISL_635412, EPI_ISL_635413, EPI_ISL_635414, EPI_ISL_635416, EPI_ISL_635417, EPI_ISL_635418, EPI_ISL_635420, EPI_ISL_635421, EPI_ISL_635422, EPI_ISL_635423, EPI_ISL_635424, EPI_ISL_635425, EPI_ISL_635427, EPI_ISL_635428, EPI_ISL_635429, EPI_ISL_635430, EPI_ISL_635432, EPI_ISL_635433, EPI_ISL_635435, EPI_ISL_635436, EPI_ISL_635437, EPI_ISL_635438, EPI_ISL_635439, EPI_ISL_635440, EPI_ISL_635441, EPI_ISL_635442, EPI_ISL_635444, EPI_ISL_635445, EPI_ISL_635446, EPI_ISL_635447, EPI_ISL_635448, EPI_ISL_635449, EPI_ISL_635451, EPI_ISL_635452, EPI_ISL_635453, EPI_ISL_635454, EPI_ISL_635455, EPI_ISL_635456, EPI_ISL_635457, EPI_ISL_635458, EPI_ISL_635459, EPI_ISL_635460, EPI_ISL_635462, EPI_ISL_635463, EPI_ISL_635466, EPI_ISL_635467, EPI_ISL_635468, EPI_ISL_635469, EPI_ISL_635470, EPI_ISL_635471, EPI_ISL_635472, EPI_ISL_635473, EPI_ISL_635474, EPI_ISL_635475, EPI_ISL_635476, EPI_ISL_635477, EPI_ISL_635478			
see above	San Diego County Public Health Laboratory	Andersen lab at Scripps Research	SEARCH Alliance San Diego with Tracy Basler, Jovan Shephard, Brett Austin
EPI_ISL_635480, EPI_ISL_635481, EPI_ISL_635483, EPI_ISL_635484, EPI_ISL_635486, EPI_ISL_635489, EPI_ISL_635491, EPI_ISL_635492, EPI_ISL_635493, EPI_ISL_635494, EPI_ISL_635495, EPI_ISL_635496, EPI_ISL_635497, EPI_ISL_635498, EPI_ISL_635499, EPI_ISL_635500, EPI_ISL_635501, EPI_ISL_635502, EPI_ISL_635504, EPI_ISL_635505, EPI_ISL_635506, EPI_ISL_635507, EPI_ISL_635508, EPI_ISL_635510, EPI_ISL_635511, EPI_ISL_635512, EPI_ISL_635513, EPI_ISL_635515, EPI_ISL_635516, EPI_ISL_635517, EPI_ISL_635518, EPI_ISL_635519, EPI_ISL_635520, EPI_ISL_635521, EPI_ISL_635522, EPI_ISL_635523, EPI_ISL_635524, EPI_ISL_635525, EPI_ISL_635526, EPI_ISL_635527, EPI_ISL_635528, EPI_ISL_635529, EPI_ISL_635530, EPI_ISL_635532, EPI_ISL_635533, EPI_ISL_635534, EPI_ISL_635535, EPI_ISL_635536, EPI_ISL_635537, EPI_ISL_635538, EPI_ISL_635539, EPI_ISL_635540, EPI_ISL_635541, EPI_ISL_635542, EPI_ISL_635543, EPI_ISL_635544, EPI_ISL_635545, EPI_ISL_635546, EPI_ISL_635547, EPI_ISL_635548, EPI_ISL_635549, EPI_ISL_635550, EPI_ISL_635551, EPI_ISL_635552, EPI_ISL_635553, EPI_ISL_635554, EPI_ISL_635555, EPI_ISL_635556, EPI_ISL_635557, EPI_ISL_635558, EPI_ISL_635559, EPI_ISL_635560, EPI_ISL_635561, EPI_ISL_635562, EPI_ISL_635563, EPI_ISL_635564, EPI_ISL_635565, EPI_ISL_635566, EPI_ISL_635567, EPI_ISL_635568, EPI_ISL_635569, EPI_ISL_635570, EPI_ISL_635571, EPI_ISL_635572, EPI_ISL_635573, EPI_ISL_635574, EPI_ISL_635575, EPI_ISL_635576			
see above	Centro de Diagnstico COVID-19 UABC Tijuana	Andersen lab at Scripps Research	SEARCH Alliance San Diego with Idanya Rubí Serafín Higuera, Manuel Sánchez Alavez, Jorge Luis Jiménez Niebla, Germán Ibarra, Jonathan Vincent Baena, Oscar Efrén Zazueta Fierro
EPI_ISL_635577, EPI_ISL_635578, EPI_ISL_635579, EPI_ISL_635580, EPI_ISL_635582, EPI_ISL_635583, EPI_ISL_635584, EPI_ISL_635585, EPI_ISL_635586, EPI_ISL_635587, EPI_ISL_635588, EPI_ISL_635589, EPI_ISL_635591, EPI_ISL_635592, EPI_ISL_635593, EPI_ISL_635594, EPI_ISL_635595, EPI_ISL_635596, EPI_ISL_635597, EPI_ISL_635598, EPI_ISL_635600, EPI_ISL_635601, EPI_ISL_635602, EPI_ISL_635603, EPI_ISL_635604, EPI_ISL_635605, EPI_ISL_635606, EPI_ISL_635607, EPI_ISL_635608, EPI_ISL_635609, EPI_ISL_635610, EPI_ISL_635611, EPI_ISL_635612, EPI_ISL_635613, EPI_ISL_635614, EPI_ISL_635615, EPI_ISL_635616, EPI_ISL_635617, EPI_ISL_635618, EPI_ISL_635619, EPI_ISL_635620, EPI_ISL_635621, EPI_ISL_635622, EPI_ISL_635623, EPI_ISL_635624, EPI_ISL_635625, EPI_ISL_635626, EPI_ISL_635627, EPI_ISL_635628, EPI_ISL_635629, EPI_ISL_635630, EPI_ISL_635632, EPI_ISL_635633, EPI_ISL_635634, EPI_ISL_635635, EPI_ISL_635636, EPI_ISL_635637, EPI_ISL_635638, EPI_ISL_635639, EPI_ISL_635640, EPI_ISL_635642, EPI_ISL_635643, EPI_ISL_635644, EPI_ISL_635646, EPI_ISL_635648, EPI_ISL_635649, EPI_ISL_635651, EPI_ISL_635652, EPI_ISL_635653, EPI_ISL_635654, EPI_ISL_635655, EPI_ISL_635657, EPI_ISL_635658, EPI_ISL_635660, EPI_ISL_635661, EPI_ISL_635663, EPI_ISL_635664, EPI_ISL_635668, EPI_ISL_635669, EPI_ISL_635670, EPI_ISL_635671, EPI_ISL_635672, EPI_ISL_635673, EPI_ISL_635674, EPI_ISL_635675, EPI_ISL_635676, EPI_ISL_635677, EPI_ISL_635678, EPI_ISL_635680, EPI_ISL_635681, EPI_ISL_635683, EPI_ISL_635684, EPI_ISL_635685, EPI_ISL_635686, EPI_ISL_635688, EPI_ISL_635689, EPI_ISL_635690, EPI_ISL_635691, EPI_ISL_635692, EPI_ISL_635693, EPI_ISL_635694, EPI_ISL_635695, EPI_ISL_635696, EPI_ISL_635697, EPI_ISL_635698, EPI_ISL_635699, EPI_ISL_635701, EPI_ISL_635702, EPI_ISL_635703, EPI_ISL_635704, EPI_ISL_635705, EPI_ISL_635706, EPI_ISL_635708, EPI_ISL_635709, EPI_ISL_635710, EPI_ISL_635711, EPI_ISL_635712, EPI_ISL_635713, EPI_ISL_635714, EPI_ISL_635716, EPI_ISL_635719, EPI_ISL_635720, EPI_ISL_635721, EPI_ISL_635722, EPI_ISL_635724, EPI_ISL_635725, EPI_ISL_635726, EPI_ISL_635727, EPI_ISL_635729, EPI_ISL_635730, EPI_ISL_635731, EPI_ISL_635732, EPI_ISL_635733, EPI_ISL_635734, EPI_ISL_635736, EPI_ISL_635737, EPI_ISL_635739, EPI_ISL_635740, EPI_ISL_635741, EPI_ISL_635742, EPI_ISL_635743, EPI_ISL_635744, EPI_ISL_635745, EPI_ISL_635746, EPI_ISL_635747, EPI_ISL_635748, EPI_ISL_635749, EPI_ISL_635750, EPI_ISL_635751, EPI_ISL_635752, EPI_ISL_635753, EPI_ISL_635754, EPI_ISL_635755, EPI_ISL_635756, EPI_ISL_635757, EPI_ISL_635758, EPI_ISL_635759, EPI_ISL_635760, EPI_ISL_635761, EPI_ISL_635762, EPI_ISL_635763, EPI_ISL_635764, EPI_ISL_635765, EPI_ISL_635766, EPI_ISL_635767, EPI_ISL_635768, EPI_ISL_635769, EPI_ISL_635770, EPI_ISL_635771, EPI_ISL_635772, EPI_ISL_635773, EPI_ISL_635774, EPI_ISL_635775, EPI_ISL_635776			
see above	San Diego County Public Health Laboratory	Andersen lab at Scripps Research	SEARCH Alliance San Diego with Tracy Basler, Jovan Shephard, Brett Austin
EPI_ISL_635777, EPI_ISL_635778, EPI_ISL_635779, EPI_ISL_635780, EPI_ISL_635781, EPI_ISL_635782	Biolab Diagnostic Laboratories	Andersen lab at Scripps Research	Issa Abu-Dayyeh, Ahmad Tibi, Lama Hussein, Lina Mohammad, Zein Naber, Amid Abdelnour with SEARCH Alliance San Diego
EPI_ISL_635786, EPI_ISL_635787, EPI_ISL_635788, EPI_ISL_635790, EPI_ISL_635791, EPI_ISL_635792, EPI_ISL_635793, EPI_ISL_635794, EPI_ISL_635795, EPI_ISL_635796, EPI_ISL_635797, EPI_ISL_635799, EPI_ISL_635801, EPI_ISL_635802, EPI_ISL_635803, EPI_ISL_635804, EPI_ISL_635807, EPI_ISL_635808, EPI_ISL_635809, EPI_ISL_635810, EPI_ISL_635811, EPI_ISL_635812, EPI_ISL_635813, EPI_ISL_635814, EPI_ISL_635815, EPI_ISL_635816, EPI_ISL_635817, EPI_ISL_635818, EPI_ISL_635819, EPI_ISL_635820, EPI_ISL_635821, EPI_ISL_635822, EPI_ISL_635823, EPI_ISL_635824, EPI_ISL_635825, EPI_ISL_635826, EPI_ISL_635827, EPI_ISL_635828, EPI_ISL_635829, EPI_ISL_635830, EPI_ISL_635832, EPI_ISL_635833, EPI_ISL_635834, EPI_ISL_635835, EPI_ISL_635836, EPI_ISL_635837, EPI_ISL_635838, EPI_ISL_635840, EPI_ISL_635841, EPI_ISL_635842, EPI_ISL_635843, EPI_ISL_635844, EPI_ISL_635845, EPI_ISL_635846, EPI_ISL_635847, EPI_ISL_635848, EPI_ISL_635849, EPI_ISL_635852, EPI_ISL_635853, EPI_ISL_635854, EPI_ISL_635855, EPI_ISL_635856, EPI_ISL_635857, EPI_ISL_635858, EPI_ISL_635859, EPI_ISL_635860, EPI_ISL_635861, EPI_ISL_635862, EPI_ISL_635863, EPI_ISL_635864, EPI_ISL_635865, EPI_ISL_635866, EPI_ISL_635867, EPI_ISL_635868, EPI_ISL_635869, EPI_ISL_635870, EPI_ISL_635871, EPI_ISL_635873, EPI_ISL_635874, EPI_ISL_635875, EPI_ISL_635876, EPI_ISL_635877, EPI_ISL_635878, EPI_ISL_635879, EPI_ISL_635880, EPI_ISL_635881, EPI_ISL_635882, EPI_ISL_635883, EPI_ISL_635885, EPI_ISL_635886, EPI_ISL_635887, EPI_ISL_635888, EPI_ISL_635889, EPI_ISL_635890, EPI_ISL_635891, EPI_ISL_635892, EPI_ISL_635893, EPI_ISL_635894, EPI_ISL_635895, EPI_ISL_635896, EPI_ISL_635897, EPI_ISL_635898, EPI_ISL_635899, EPI_ISL_635900, EPI_ISL_635901, EPI_ISL_635902, EPI_ISL_635903, EPI_ISL_635904, EPI_ISL_635905, EPI_ISL_635906, EPI_ISL_635907, EPI_ISL_635908, EPI_ISL_635909, EPI_ISL_635912, EPI_ISL_635913, EPI_ISL_635914, EPI_ISL_635917, EPI_ISL_635918, EPI_ISL_635919, EPI_ISL_635920, EPI_ISL_635921, EPI_ISL_635922, EPI_ISL_635923, EPI_ISL_635924, EPI_ISL_635925, EPI_ISL_635929,			

EPI_ISL_635930, EPI_ISL_635931, EPI_ISL_635932, EPI_ISL_635933, EPI_ISL_635934, EPI_ISL_635935, EPI_ISL_635936, EPI_ISL_635937, EPI_ISL_635939, EPI_ISL_635940, EPI_ISL_635941, EPI_ISL_635942, EPI_ISL_635943, EPI_ISL_635944, EPI_ISL_635945, EPI_ISL_635946, EPI_ISL_635947, EPI_ISL_635948, EPI_ISL_635949, EPI_ISL_635950, EPI_ISL_635951, EPI_ISL_635952, EPI_ISL_635953, EPI_ISL_635954, EPI_ISL_635956, EPI_ISL_635957, EPI_ISL_635958, EPI_ISL_635959, EPI_ISL_635960, EPI_ISL_635961, EPI_ISL_635962, EPI_ISL_635963, EPI_ISL_635964, EPI_ISL_635965, EPI_ISL_635966, EPI_ISL_635967, EPI_ISL_635968, EPI_ISL_635969, EPI_ISL_635972, EPI_ISL_635973, EPI_ISL_635974, EPI_ISL_635975, EPI_ISL_635976, EPI_ISL_635977, EPI_ISL_635980, EPI_ISL_635981, EPI_ISL_635982, EPI_ISL_635983, EPI_ISL_635984, EPI_ISL_635985, EPI_ISL_635986, EPI_ISL_635987, EPI_ISL_635988, EPI_ISL_635989, EPI_ISL_635990, EPI_ISL_635991, EPI_ISL_635992, EPI_ISL_635993, EPI_ISL_635994, EPI_ISL_635996, EPI_ISL_635997, EPI_ISL_635998, EPI_ISL_635999, EPI_ISL_636001, EPI_ISL_636002, EPI_ISL_636003, EPI_ISL_636004, EPI_ISL_636005, EPI_ISL_636006, EPI_ISL_636010, EPI_ISL_636011, EPI_ISL_636012, EPI_ISL_636014, EPI_ISL_636016, EPI_ISL_636017, EPI_ISL_636018, EPI_ISL_636019, EPI_ISL_636022, EPI_ISL_636023, EPI_ISL_636024, EPI_ISL_636025, EPI_ISL_636026, EPI_ISL_636027, EPI_ISL_636028, EPI_ISL_636029, EPI_ISL_636031, EPI_ISL_636032, EPI_ISL_636033, EPI_ISL_636034, EPI_ISL_636035, EPI_ISL_636036, EPI_ISL_636037, EPI_ISL_636038, EPI_ISL_636039, EPI_ISL_636040, EPI_ISL_636041, EPI_ISL_636042, EPI_ISL_636043, EPI_ISL_636044, EPI_ISL_636045, EPI_ISL_636046, EPI_ISL_636047, EPI_ISL_636048, EPI_ISL_636049, EPI_ISL_636050, EPI_ISL_636051, EPI_ISL_636052, EPI_ISL_636053, EPI_ISL_636054, EPI_ISL_636056, EPI_ISL_636057, EPI_ISL_636059, EPI_ISL_636060, EPI_ISL_636061, EPI_ISL_636062, EPI_ISL_636063, EPI_ISL_636065, EPI_ISL_636066, EPI_ISL_636067, EPI_ISL_636068, EPI_ISL_636069, EPI_ISL_636070, EPI_ISL_636071, EPI_ISL_636072, EPI_ISL_636073, EPI_ISL_636074, EPI_ISL_636075, EPI_ISL_636076, EPI_ISL_636077, EPI_ISL_636078, EPI_ISL_636079, EPI_ISL_636080, EPI_ISL_636081, EPI_ISL_636082, EPI_ISL_636084, EPI_ISL_636085, EPI_ISL_636086, EPI_ISL_636087, EPI_ISL_636088, EPI_ISL_636090, EPI_ISL_636091, EPI_ISL_636092, EPI_ISL_636093, EPI_ISL_636094, EPI_ISL_636095, EPI_ISL_636096, EPI_ISL_636097, EPI_ISL_636098, EPI_ISL_636100, EPI_ISL_636101, EPI_ISL_636102, EPI_ISL_636104, EPI_ISL_636105, EPI_ISL_636106, EPI_ISL_636107, EPI_ISL_636108, EPI_ISL_636109, EPI_ISL_636110, EPI_ISL_636111, EPI_ISL_636112			
see above	San Diego County Public Health Laboratory	Andersen lab at Scripps Research	SEARCH Alliance San Diego with Tracy Basler, Jovan Shephard, Brett Austin
EPI_ISL_636113, EPI_ISL_636114, EPI_ISL_636115, EPI_ISL_636116, EPI_ISL_636117, EPI_ISL_636118, EPI_ISL_636119	Sharp HealthCare Laboratory	Andersen lab at Scripps Research	SEARCH Alliance San Diego with Aaron Harding, Jacquelyn Berumen, Cathy Woerle, Liam McGinnis
EPI_ISL_636121, EPI_ISL_636122, EPI_ISL_636123, EPI_ISL_636124, EPI_ISL_636125, EPI_ISL_636126, EPI_ISL_636127, EPI_ISL_636128, EPI_ISL_636129, EPI_ISL_636130, EPI_ISL_636131, EPI_ISL_636132, EPI_ISL_636133, EPI_ISL_636134, EPI_ISL_636135, EPI_ISL_636136, EPI_ISL_636137, EPI_ISL_636138, EPI_ISL_636139, EPI_ISL_636140, EPI_ISL_636141, EPI_ISL_636143, EPI_ISL_636144, EPI_ISL_636145, EPI_ISL_636146, EPI_ISL_636147, EPI_ISL_636148, EPI_ISL_636149, EPI_ISL_636150, EPI_ISL_636151, EPI_ISL_636152, EPI_ISL_636153, EPI_ISL_636154, EPI_ISL_636156, EPI_ISL_636157, EPI_ISL_636158, EPI_ISL_636160, EPI_ISL_636161, EPI_ISL_636162, EPI_ISL_636163, EPI_ISL_636165, EPI_ISL_636166, EPI_ISL_636167, EPI_ISL_636168, EPI_ISL_636169, EPI_ISL_636170, EPI_ISL_636172, EPI_ISL_636173, EPI_ISL_636174, EPI_ISL_636175, EPI_ISL_636177, EPI_ISL_636178, EPI_ISL_636179, EPI_ISL_636180, EPI_ISL_636181, EPI_ISL_636182, EPI_ISL_636183, EPI_ISL_636184, EPI_ISL_636185, EPI_ISL_636186, EPI_ISL_636187, EPI_ISL_636188, EPI_ISL_636189, EPI_ISL_636190, EPI_ISL_636192, EPI_ISL_636193, EPI_ISL_636194, EPI_ISL_636195, EPI_ISL_636196, EPI_ISL_636197, EPI_ISL_636198, EPI_ISL_636199, EPI_ISL_636200, EPI_ISL_636201, EPI_ISL_636202, EPI_ISL_636203, EPI_ISL_636204, EPI_ISL_636205, EPI_ISL_636206, EPI_ISL_636207, EPI_ISL_636208, EPI_ISL_636209, EPI_ISL_636210, EPI_ISL_636211, EPI_ISL_636212, EPI_ISL_636213, EPI_ISL_636214, EPI_ISL_636215, EPI_ISL_636216, EPI_ISL_636217, EPI_ISL_636218, EPI_ISL_636219, EPI_ISL_636220, EPI_ISL_636221, EPI_ISL_636222, EPI_ISL_636223, EPI_ISL_636224, EPI_ISL_636225, EPI_ISL_636226, EPI_ISL_636227, EPI_ISL_636228, EPI_ISL_636229, EPI_ISL_636230, EPI_ISL_636231, EPI_ISL_636232, EPI_ISL_636233, EPI_ISL_636234, EPI_ISL_636235, EPI_ISL_636236, EPI_ISL_636237, EPI_ISL_636239, EPI_ISL_636240, EPI_ISL_636241, EPI_ISL_636242, EPI_ISL_636243, EPI_ISL_636244, EPI_ISL_636245, EPI_ISL_636246, EPI_ISL_636247, EPI_ISL_636248, EPI_ISL_636250, EPI_ISL_636251, EPI_ISL_636252, EPI_ISL_636253, EPI_ISL_636254, EPI_ISL_636255, EPI_ISL_636256, EPI_ISL_636258, EPI_ISL_636260, EPI_ISL_636261, EPI_ISL_636262, EPI_ISL_636263, EPI_ISL_636264, EPI_ISL_636265, EPI_ISL_636266			
see above	San Diego County Public Health Laboratory	Andersen lab at Scripps Research	SEARCH Alliance San Diego with Tracy Basler, Jovan Shephard, Brett Austin
EPI_ISL_636267, EPI_ISL_636268, EPI_ISL_636269, EPI_ISL_636270, EPI_ISL_636271, EPI_ISL_636272, EPI_ISL_636273, EPI_ISL_636274, EPI_ISL_636275, EPI_ISL_636276, EPI_ISL_636277, EPI_ISL_636278, EPI_ISL_636279, EPI_ISL_636280, EPI_ISL_636281, EPI_ISL_636282, EPI_ISL_636283, EPI_ISL_636284, EPI_ISL_636285, EPI_ISL_636286, EPI_ISL_636287, EPI_ISL_636288, EPI_ISL_636289, EPI_ISL_636290, EPI_ISL_636291, EPI_ISL_636292, EPI_ISL_636293, EPI_ISL_636294, EPI_ISL_636295, EPI_ISL_636296, EPI_ISL_636297, EPI_ISL_636298, EPI_ISL_636299, EPI_ISL_636300, EPI_ISL_636301, EPI_ISL_636302, EPI_ISL_636303, EPI_ISL_636304, EPI_ISL_636305, EPI_ISL_636306, EPI_ISL_636307, EPI_ISL_636308, EPI_ISL_636309, EPI_ISL_636310, EPI_ISL_636311, EPI_ISL_636312, EPI_ISL_636313, EPI_ISL_636314, EPI_ISL_636315, EPI_ISL_636316, EPI_ISL_636317, EPI_ISL_636318, EPI_ISL_636319, EPI_ISL_636320, EPI_ISL_636321, EPI_ISL_636322, EPI_ISL_636323, EPI_ISL_636324, EPI_ISL_636325, EPI_ISL_636326, EPI_ISL_636327, EPI_ISL_636328, EPI_ISL_636329, EPI_ISL_636330, EPI_ISL_636331, EPI_ISL_636332, EPI_ISL_636333, EPI_ISL_636334, EPI_ISL_636335, EPI_ISL_636336, EPI_ISL_636337, EPI_ISL_636338, EPI_ISL_636339, EPI_ISL_636340, EPI_ISL_636341, EPI_ISL_636342, EPI_ISL_636343, EPI_ISL_636344, EPI_ISL_636345, EPI_ISL_636346, EPI_ISL_636347, EPI_ISL_636348, EPI_ISL_636349, EPI_ISL_636350, EPI_ISL_636351, EPI_ISL_636352, EPI_ISL_636353, EPI_ISL_636354, EPI_ISL_636355, EPI_ISL_636356, EPI_ISL_636357, EPI_ISL_636358, EPI_ISL_636359, EPI_ISL_636360, EPI_ISL_636362, EPI_ISL_636363, EPI_ISL_636364, EPI_ISL_636365, EPI_ISL_636366, EPI_ISL_636367, EPI_ISL_636368, EPI_ISL_636369, EPI_ISL_636370, EPI_ISL_636371, EPI_ISL_636372, EPI_ISL_636373, EPI_ISL_636374, EPI_ISL_636375, EPI_ISL_636376, EPI_ISL_636377, EPI_ISL_636378, EPI_ISL_636379, EPI_ISL_636380, EPI_ISL_636381, EPI_ISL_636382, EPI_ISL_636383, EPI_ISL_636384, EPI_ISL_636385, EPI_ISL_636386, EPI_ISL_636388, EPI_ISL_636389, EPI_ISL_636391, EPI_ISL_636392, EPI_ISL_636393, EPI_ISL_636394, EPI_ISL_636395, EPI_ISL_636396, EPI_ISL_636397, EPI_ISL_636398, EPI_ISL_636399, EPI_ISL_636400, EPI_ISL_636401, EPI_ISL_636402, EPI_ISL_636403, EPI_ISL_636404, EPI_ISL_636405, EPI_ISL_636406, EPI_ISL_636407, EPI_ISL_636408, EPI_ISL_636409, EPI_ISL_636410, EPI_ISL_636411, EPI_ISL_636412, EPI_ISL_636413, EPI_ISL_636414, EPI_ISL_636415, EPI_ISL_636416, EPI_ISL_636417, EPI_ISL_636418, EPI_ISL_636419, EPI_ISL_636420, EPI_ISL_636421, EPI_ISL_636422, EPI_ISL_636423, EPI_ISL_636424, EPI_ISL_636425, EPI_ISL_636426, EPI_ISL_636427, EPI_ISL_636428, EPI_ISL_636429, EPI_ISL_636430, EPI_ISL_636431, EPI_ISL_636432, EPI_ISL_636433, EPI_ISL_636434, EPI_ISL_636435, EPI_ISL_636436, EPI_ISL_636437, EPI_ISL_636438, EPI_ISL_636439, EPI_ISL_636440, EPI_ISL_636441, EPI_ISL_636442, EPI_ISL_636443, EPI_ISL_636444, EPI_ISL_636445, EPI_ISL_636446, EPI_ISL_636447, EPI_ISL_636448, EPI_ISL_636449, EPI_ISL_636450, EPI_ISL_636451, EPI_ISL_636452, EPI_ISL_636453, EPI_ISL_636454, EPI_ISL_636455, EPI_ISL_636456, EPI_ISL_636457, EPI_ISL_636458			
see above	Biolab Diagnostic Laboratories	Andersen lab at Scripps Research	Issa Abu-Dayyeh, Ahmad Tibi, Lama Hussein, Lina Mohammad, Zein Naber, Amid Abdelnour with SEARCH Alliance San Diego
EPI_ISL_636462, EPI_ISL_636463	ULSS6 Euganea	Istituto Zooprofilattico Sperimentale delle Venezie	Adelaide Milani, Alessia Schivo, Annalisa Salviato, Erika Giorgia Quaranta, Gianpiero Zamperin, Ambra Pastori, Bianca Zecchin, Alice Fusaro, Calogero Terregino, Antonia Ricci
EPI_ISL_636464	ULSS6 Piove di Sacco	Istituto Zooprofilattico Sperimentale delle Venezie	Adelaide Milani, Alessia Schivo, Annalisa Salviato, Erika Giorgia Quaranta, Gianpiero Zamperin, Ambra Pastori, Bianca Zecchin, Alice Fusaro, Calogero Terregino, Antonia Ricci
EPI_ISL_636467, EPI_ISL_636468, EPI_ISL_636469, EPI_ISL_636470	ULSS6 Distretto Padova Terme Colli	Istituto Zooprofilattico Sperimentale delle Venezie	Adelaide Milani, Alessia Schivo, Annalisa Salviato, Erika Giorgia Quaranta, Gianpiero Zamperin, Ambra Pastori, Bianca Zecchin, Alice Fusaro, Calogero Terregino, Antonia Ricci
EPI_ISL_636471, EPI_ISL_636472, EPI_ISL_636473	ULSS6 Piove di Sacco	Istituto Zooprofilattico Sperimentale delle Venezie	Adelaide Milani, Alessia Schivo, Annalisa Salviato, Erika Giorgia Quaranta, Gianpiero Zamperin, Ambra Pastori, Bianca Zecchin, Alice Fusaro, Calogero Terregino, Antonia Ricci
EPI_ISL_636474, EPI_ISL_636475	ULSS6 Distretto Padova Terme Colli	Istituto Zooprofilattico Sperimentale delle Venezie	Adelaide Milani, Alessia Schivo, Annalisa Salviato, Erika Giorgia Quaranta, Gianpiero Zamperin, Ambra Pastori, Bianca Zecchin, Alice Fusaro, Calogero Terregino, Antonia Ricci
EPI_ISL_636476, EPI_ISL_636477	CNR Virus des Infections Respiratoires - France SUD	CNR Virus des Infections Respiratoires - France SUD	Antonin Bal, Gregory Destras, Gwendolyne Burfin, Hadrien Règue, Alexandre Gaymard, Maude Bouscambert-Duchamp, Florence Morfin-Sherpa, Martine Valette, Bruno Lina, Laurence Josset
EPI_ISL_636478	Centre Hospitalier Pierre Oudot	CNR Virus des Infections Respiratoires - France SUD	Antonin Bal, Gregory Destras, Gwendolyne Burfin, Hadrien Règue, Alexandre Gaymard, Maude Bouscambert-Duchamp, Florence Morfin-Sherpa, Martine Valette, Bruno Lina, Laurence Josset
EPI_ISL_636479	CNR Virus des Infections Respiratoires - France SUD	CNR Virus des Infections Respiratoires - France SUD	Antonin Bal, Gregory Destras, Gwendolyne Burfin, Hadrien Règue, Alexandre Gaymard, Maude Bouscambert-Duchamp, Florence Morfin-Sherpa, Martine Valette, Bruno Lina, Laurence Josset
EPI_ISL_636480	Centre Hospitalier Pierre Oudot	CNR Virus des Infections Respiratoires - France SUD	Antonin Bal, Gregory Destras, Gwendolyne Burfin, Hadrien Règue, Alexandre Gaymard, Maude Bouscambert-Duchamp, Florence Morfin-Sherpa, Martine Valette, Bruno Lina, Laurence Josset
EPI_ISL_636481	Centre Hospitalier de Bourg en Bresse	CNR Virus des Infections Respiratoires - France SUD	Antonin Bal, Gregory Destras, Gwendolyne Burfin, Hadrien Règue, Alexandre Gaymard, Maude Bouscambert-Duchamp, Florence Morfin-Sherpa, Martine Valette, Bruno Lina, Laurence Josset
EPI_ISL_636482, EPI_ISL_636483	Centre Hospitalier Pierre Oudot	CNR Virus des Infections Respiratoires - France SUD	Antonin Bal, Gregory Destras, Gwendolyne Burfin, Hadrien Règue, Alexandre Gaymard, Maude Bouscambert-Duchamp, Florence Morfin-Sherpa, Martine Valette, Bruno Lina, Laurence Josset
EPI_ISL_636484, EPI_ISL_636485	CNR Virus des Infections Respiratoires - France SUD	CNR Virus des Infections Respiratoires - France SUD	Antonin Bal, Gregory Destras, Gwendolyne Burfin, Hadrien Règue, Alexandre Gaymard, Maude Bouscambert-Duchamp, Florence Morfin-Sherpa, Martine Valette, Bruno Lina, Laurence Josset
EPI_ISL_636486	Centre Hospitalier Pierre Oudot	CNR Virus des Infections Respiratoires - France SUD	Antonin Bal, Gregory Destras, Gwendolyne Burfin, Hadrien Règue, Alexandre Gaymard, Maude Bouscambert-Duchamp, Florence Morfin-Sherpa, Martine Valette, Bruno Lina, Laurence Josset
EPI_ISL_636487	CNR Virus des Infections Respiratoires - France SUD	CNR Virus des Infections Respiratoires - France SUD	Antonin Bal, Gregory Destras, Gwendolyne Burfin, Hadrien Règue, Alexandre Gaymard, Maude Bouscambert-Duchamp, Florence Morfin-Sherpa, Martine Valette, Bruno Lina, Laurence Josset
EPI_ISL_636488	ULSS9 Scaligera	Istituto Zooprofilattico Sperimentale delle Venezie	Adelaide Milani, Alessia Schivo, Annalisa Salviato, Erika Giorgia Quaranta, Gianpiero Zamperin, Ambra Pastori, Bianca Zecchin, Alice Fusaro, Calogero Terregino, Antonia Ricci
EPI_ISL_636491, EPI_ISL_636492, EPI_ISL_636493, EPI_ISL_636494, EPI_ISL_636495, EPI_ISL_636496, EPI_ISL_636497, EPI_ISL_636498, EPI_ISL_636499, EPI_ISL_636500, EPI_ISL_636501, EPI_ISL_636502, EPI_ISL_636503, EPI_ISL_636504, EPI_ISL_636505, EPI_ISL_636506, EPI_ISL_636507, EPI_ISL_636508, EPI_ISL_636509, EPI_ISL_636510, EPI_ISL_636511, EPI_ISL_636512, EPI_ISL_636513, EPI_ISL_636514, EPI_ISL_636515, EPI_ISL_636516, EPI_ISL_636517, EPI_ISL_636518, EPI_ISL_636519, EPI_ISL_636520, EPI_ISL_636521, EPI_ISL_636522, EPI_ISL_636523, EPI_ISL_636524, EPI_ISL_636525, EPI_ISL_636526, EPI_ISL_636527, EPI_ISL_636528, EPI_ISL_636529, EPI_ISL_636530, EPI_ISL_636531, EPI_ISL_636532, EPI_ISL_636533, EPI_ISL_636534, EPI_ISL_636535, EPI_ISL_636536, EPI_ISL_636537, EPI_ISL_636538, EPI_ISL_636539, EPI_ISL_636540, EPI_ISL_636541, EPI_ISL_636542, EPI_ISL_636543, EPI_ISL_636544, EPI_ISL_636545, EPI_ISL_636546, EPI_ISL_636547, EPI_ISL_636548, EPI_ISL_636549, EPI_ISL_636550, EPI_ISL_636551, EPI_ISL_636552, EPI_ISL_636553, EPI_ISL_636554, EPI_ISL_636555, EPI_ISL_636556, EPI_ISL_636557, EPI_ISL_636558, EPI_ISL_636559, EPI_ISL_636560, EPI_ISL_636561, EPI_ISL_636562, EPI_ISL_636563, EPI_ISL_636564, EPI_ISL_636565, EPI_ISL_636566, EPI_ISL_636567, EPI_ISL_636568, EPI_ISL_636569, EPI_ISL_636570, EPI_ISL_636571, EPI_ISL_636572, EPI_ISL_636573, EPI_ISL_636574, EPI_ISL_636575, EPI_ISL_636576, EPI_ISL_636577, EPI_ISL_636578, EPI_ISL_636579, EPI_ISL_636580, EPI_ISL_636581, EPI_ISL_636582, EPI_ISL_636583, EPI_ISL_636584, EPI_ISL_636585, EPI_ISL_636586, EPI_ISL_636587, EPI_ISL_636588, EPI_ISL_636589, EPI_ISL_636590, EPI_ISL_636591, EPI_ISL_636592, EPI_ISL_636593, EPI_ISL_636594, EPI_ISL_636595, EPI_ISL_636596, EPI_ISL_636597, EPI_ISL_636598, EPI_ISL_636599, EPI_ISL_636600, EPI_ISL_636601, EPI_ISL_636602, EPI_ISL_636603			
see above	Dutch COVID-19 response team	National Institute for Public Health and the Environment (RIVM)	Adam Meijer, Harry Vennema, Jeroen Cremer, Sharon van den Brink, Bas van der Veer, AnneMarie van den Brandt, Florian Zwagemaker, Dennis Schmitz, Chantal Reusken, on behalf of the national COVID-19 response team
EPI_ISL_636604	Lithuanian University of Health Sciences Hospital, Department of Laboratory Medicine	Lithuanian University of Health Sciences, Molecular cardiology lab.	Lukas Zemaitis, Ingrida Olendrait, Arnoldas Pautienius, Kamile Tamusauskaite, Dovydas Gecys, Laura Pareckaite, Vaiva Lesauskaite, Astra Vitkauskiene

EPI_ISL_636605, EPI_ISL_636606, EPI_ISL_636607, EPI_ISL_636608, EPI_ISL_636609, EPI_ISL_636610, EPI_ISL_636611, EPI_ISL_636612, EPI_ISL_636613, EPI_ISL_636614, EPI_ISL_636615, EPI_ISL_636616, EPI_ISL_636617, EPI_ISL_636619, EPI_ISL_636620, EPI_ISL_636621, EPI_ISL_636622, EPI_ISL_636623, EPI_ISL_636624, EPI_ISL_636625, EPI_ISL_636626, EPI_ISL_636627, EPI_ISL_636628, EPI_ISL_636629, EPI_ISL_636630, EPI_ISL_636631, EPI_ISL_636632, EPI_ISL_636633, EPI_ISL_636634, EPI_ISL_636635, EPI_ISL_636636, EPI_ISL_636637, EPI_ISL_636638, EPI_ISL_636639, EPI_ISL_636640, EPI_ISL_636641, EPI_ISL_636642, EPI_ISL_636643, EPI_ISL_636644, EPI_ISL_636645, EPI_ISL_636646, EPI_ISL_636647, EPI_ISL_636648, EPI_ISL_636649, EPI_ISL_636650, EPI_ISL_636651, EPI_ISL_636652, EPI_ISL_636653, EPI_ISL_636654, EPI_ISL_636655, EPI_ISL_636656, EPI_ISL_636657, EPI_ISL_636658, EPI_ISL_636659, EPI_ISL_636660, EPI_ISL_636661, EPI_ISL_636662, EPI_ISL_636663, EPI_ISL_636664, EPI_ISL_636665, EPI_ISL_636666, EPI_ISL_636667, EPI_ISL_636668, EPI_ISL_636669, EPI_ISL_636670, EPI_ISL_636671, EPI_ISL_636672, EPI_ISL_636673, EPI_ISL_636674, EPI_ISL_636675, EPI_ISL_636676, EPI_ISL_636677, EPI_ISL_636679, EPI_ISL_636680, EPI_ISL_636681, EPI_ISL_636682, EPI_ISL_636684, EPI_ISL_636685			
see above	Department of Clinical Microbiology	GIGA Medical Genomics	Keith Durkin, Maria Artesi, Sébastien Bontems, Raphaël Boreux, Bouchra Boujemla, Cécile Meex, Pierrette Melin, Marie-Pierre Hayette, Vincent Bours
EPI_ISL_636686, EPI_ISL_636687, EPI_ISL_636688, EPI_ISL_636689, EPI_ISL_636690, EPI_ISL_636691, EPI_ISL_636692, EPI_ISL_636693, EPI_ISL_636694, EPI_ISL_636695, EPI_ISL_636696, EPI_ISL_636697, EPI_ISL_636699, EPI_ISL_636700, EPI_ISL_636701, EPI_ISL_636702, EPI_ISL_636703, EPI_ISL_636705, EPI_ISL_636706, EPI_ISL_636707, EPI_ISL_636708, EPI_ISL_636709, EPI_ISL_636710, EPI_ISL_636711, EPI_ISL_636712, EPI_ISL_636714, EPI_ISL_636715, EPI_ISL_636716, EPI_ISL_636718, EPI_ISL_636719, EPI_ISL_636720, EPI_ISL_636721, EPI_ISL_636722, EPI_ISL_636723, EPI_ISL_636724, EPI_ISL_636728, EPI_ISL_636729, EPI_ISL_636730, EPI_ISL_636731, EPI_ISL_636732, EPI_ISL_636733, EPI_ISL_636734			
see above	Respiratory Virus Unit, Microbiology Services Colindale, Public Health England	Respiratory Virus Unit, Microbiology Services Colindale, Public Health England	PHE Covid Sequencing Team
EPI_ISL_636737	Laboratório de Imunofarmacologia - Instituto Oswaldo Cruz	Laboratório de Imunofarmacologia - Instituto Oswaldo Cruz	Souza,T.M., Fintelman-Rodrigues,N., De Paula,A.D., Saraiva,F.B., Ferreira,M.A. and Sacramento,C.Q.
EPI_ISL_636740, EPI_ISL_636742, EPI_ISL_636743, EPI_ISL_636746, EPI_ISL_636747, EPI_ISL_636748, EPI_ISL_636750, EPI_ISL_636752, EPI_ISL_636753, EPI_ISL_636754, EPI_ISL_636755, EPI_ISL_636756, EPI_ISL_636757, EPI_ISL_636759, EPI_ISL_636760, EPI_ISL_636761, EPI_ISL_636762, EPI_ISL_636766, EPI_ISL_636770, EPI_ISL_636774, EPI_ISL_636776, EPI_ISL_636777, EPI_ISL_636780, EPI_ISL_636781, EPI_ISL_636787, EPI_ISL_636788, EPI_ISL_636795, EPI_ISL_636801, EPI_ISL_636802, EPI_ISL_636804, EPI_ISL_636805, EPI_ISL_636809, EPI_ISL_636811, EPI_ISL_636813, EPI_ISL_636815, EPI_ISL_636816, EPI_ISL_636817, EPI_ISL_636818, EPI_ISL_636819, EPI_ISL_636820, EPI_ISL_636821, EPI_ISL_636822, EPI_ISL_636823, EPI_ISL_636824, EPI_ISL_636826, EPI_ISL_636828, EPI_ISL_636829, EPI_ISL_636830, EPI_ISL_636831			
see above	National Centre for Disease control (NCDC)	NCDC/CSIR-IGIB	Mahesh S. Dhar1*, Bharathram Uppili2*, Robin Marwal1*, Pooja Sharma2*, RadhaKrishnan VS, Vivekanand A, Nishu Tyagi, Shaista Khan, Simmi Tiwari, Manish Kumar, Ajit Shewale, Ishtaq Ahmed, Asangla Kamai, Aparna Swaminathan, Saruchi Wadhwa, Tushar Nale, Sandhya Kabra, Sujeet Singh, Mohammed Faruq#, Anurag Agrawal#, Partha Rakshit#
EPI_ISL_636834, EPI_ISL_636835, EPI_ISL_636837	Laboratório de Imunofarmacologia - Instituto Oswaldo Cruz	Laboratório de Imunofarmacologia - Instituto Oswaldo Cruz	Souza,T.M., Fintelman-Rodrigues,N., De Paula,A.D., Saraiva,F.B., Ferreira,M.A. and Sacramento,C.Q.
EPI_ISL_636839, EPI_ISL_636840, EPI_ISL_636841, EPI_ISL_636842, EPI_ISL_636843, EPI_ISL_636844, EPI_ISL_636845, EPI_ISL_636846, EPI_ISL_636847, EPI_ISL_636848, EPI_ISL_636849, EPI_ISL_636850, EPI_ISL_636851, EPI_ISL_636852, EPI_ISL_636853, EPI_ISL_636854, EPI_ISL_636855, EPI_ISL_636856, EPI_ISL_636857, EPI_ISL_636858, EPI_ISL_636859, EPI_ISL_636860, EPI_ISL_636861, EPI_ISL_636863, EPI_ISL_636864, EPI_ISL_636865, EPI_ISL_636866, EPI_ISL_636867, EPI_ISL_636868, EPI_ISL_636869, EPI_ISL_636870, EPI_ISL_636872, EPI_ISL_636873, EPI_ISL_636874, EPI_ISL_636875, EPI_ISL_636876, EPI_ISL_636877, EPI_ISL_636878, EPI_ISL_636879, EPI_ISL_636880, EPI_ISL_636881, EPI_ISL_636882, EPI_ISL_636884, EPI_ISL_636885, EPI_ISL_636886, EPI_ISL_636887, EPI_ISL_636888, EPI_ISL_636889, EPI_ISL_636890, EPI_ISL_636891, EPI_ISL_636892, EPI_ISL_636893, EPI_ISL_636894, EPI_ISL_636895, EPI_ISL_636896, EPI_ISL_636897, EPI_ISL_636898, EPI_ISL_636899, EPI_ISL_636900, EPI_ISL_636901, EPI_ISL_636902, EPI_ISL_636903, EPI_ISL_636904, EPI_ISL_636905, EPI_ISL_636906			
see above	Lithuanian University of Health Sciences Hospital, Department of Laboratory Medicine	Lithuanian University of Health Sciences, Molecular cardiology lab.	Lukas Zemaitis, Ingrida Olendrait, Arnoldas Pautienius, Kamile Tamusauskaite, Dovydas Gecys, Laura Pareckaite, Vaiva Lesauskaite, Astra Vitkauskiene
EPI_ISL_636907, EPI_ISL_636908, EPI_ISL_636909, EPI_ISL_636910, EPI_ISL_636911, EPI_ISL_636912, EPI_ISL_636913, EPI_ISL_636914, EPI_ISL_636915, EPI_ISL_636916, EPI_ISL_636917, EPI_ISL_636918, EPI_ISL_636919, EPI_ISL_636920, EPI_ISL_636921, EPI_ISL_636922, EPI_ISL_636923, EPI_ISL_636924, EPI_ISL_636925, EPI_ISL_636927, EPI_ISL_636928, EPI_ISL_636929, EPI_ISL_636930, EPI_ISL_636931, EPI_ISL_636932, EPI_ISL_636933, EPI_ISL_636934, EPI_ISL_636935, EPI_ISL_636936, EPI_ISL_636937, EPI_ISL_636938, EPI_ISL_636939, EPI_ISL_636940, EPI_ISL_636941, EPI_ISL_636942, EPI_ISL_636943, EPI_ISL_636944, EPI_ISL_636945, EPI_ISL_636946, EPI_ISL_636947, EPI_ISL_636949, EPI_ISL_636950, EPI_ISL_636951, EPI_ISL_636952, EPI_ISL_636953, EPI_ISL_636954, EPI_ISL_636955, EPI_ISL_636956, EPI_ISL_636957, EPI_ISL_636958, EPI_ISL_636959			
see above	Public Health Ontario Laboratory	Public Health Ontario Laboratory	Vanessa G Allen, Philip Banh, Richard de Borja, Yao Chen, Alireza Eshaghi, Nahuel Fittipaldi, Christine Frantz, Jonathan B Gubbay, Jennifer L Guthrie, Lawrence Heisler, Esha Joshi, Michael Laszloffy, Aimin Li, Michael CY Li, Dean Maxwell, Sandeep Nagra, Samir N Patel, Heather Rilkoff, Jared Simpson, Karthikeyan Sivaraman, Yogi Sundaravadanam, Sarah Teatero, Andre Villegas, Sandra Zittermann
EPI_ISL_636964	Pathogen Genomics Lab King Abdullah University of Science and Technology(KAUST)	Pathogen Genomics Lab King Abdullah University of Science and Technology(KAUST)	Rahul P Salunke, Sharif Hala, Raeece Naeem, Sara Mfarrej, Amit Kumar Subudhi, Amanda Ooi, Luke Esau, Fadwa Alofi, Fathia Ben Rached, Afrah Alsomali, Asim Khogeer, Ahmad Bakur Mahmoud, Anwar Hashem, Naif Almontashiri, Arnab Pain
EPI_ISL_636965	Pathogen Genomics Lab King Abdullah University of Science and Technology(KAUST)	Pathogen Genomics Lab King Abdullah University of Science and Technology(KAUST)	Raeece Naeem, Rahul P Salunke, Sharif Hala, Sara Mfarrej, Amit Kumar Subudhi, Fadwa Alofi, Fathia Ben Rached, Afrah Alsomali, Asim Khogeer, Ahmad Bakur Mahmoud, Anwar Hashem, Naif Almontashiri, Arnab Pain
EPI_ISL_636966, EPI_ISL_636967	Pathogen Genomics Lab King Abdullah University of Science and Technology(KAUST)	Pathogen Genomics Lab King Abdullah University of Science and Technology(KAUST)	Fathia Ben Rached, Raeece Naeem, Sharif Hala, Fadwa Alofi, Rahul P Salunke, Sara Mfarrej, Amit Kumar Subudhi, Afrah Alsomali, Asim Khogeer, Ahmad Bakur Mahmoud, Anwar Hashem, Naif Almontashiri, Arnab Pain
EPI_ISL_636969, EPI_ISL_636970, EPI_ISL_636971	Etlik Veterinary Control Central Research Institute	Etlik Veterinary Control Central Research Institute	Sabri Hacioglu, Ahu Pakdemirli, Dilek Dulger, Erdem Danyer, Ummu Sena Sari, Cevdet Yarali, Ozcan Yildirim
EPI_ISL_636972	Pathogen Genomics Lab King Abdullah University of Science and Technology (KAUST)	Pathogen Genomics Lab King Abdullah University of Science and Technology (KAUST)	Afrah Alsomali, Fathia Ben Rached, Raeece Naeem, Sharif Hala,Rahul P Salunke, Amanda Ooi, Luke Esau, Sara Mfarrej, Amit Kumar Subudhi, Fadwa Alofi, Asim Khogeer, Kahled Algithami, Anwar Hashem, Naif Almontashiri, Arnab Pain
EPI_ISL_636973	Public Health Lab	Public Health Lab	Alwasti, H
EPI_ISL_636977	HP Pemba	KRISP, KZN Research Innovation and Sequencing Platform	Ismael N, Giandhari J, Pillay S, Tegally H, Wilkinson E, de Oliveira T, Nadia Sitoe, Paulo Arnaldo, Nedio Mabunda
EPI_ISL_636980	CS Xai Xai	KRISP, KZN Research Innovation and Sequencing Platform	Ismael N, Giandhari J, Pillay S, Tegally H, Wilkinson E, de Oliveira T, Nadia Sitoe, Paulo Arnaldo, Nedio Mabunda
EPI_ISL_636982, EPI_ISL_636983, EPI_ISL_636984, EPI_ISL_636985, EPI_ISL_636986	Virology Lab, National Institute for Biomedical Research (INRB)	Project group Epidemology of Highly Pathogenic Microorganisms, Robert Koch-Institute	Jean-Jacques Muyembe Tamfum, Steve Ahuka-Mundeke, Eddy Kinganda-Lusamaki, Gabriel Mbunsu, Sheila Makiala, Essia Belarbi, Jasmin Schlotterbeck, Grit Schubert, Fabian Leendertz
EPI_ISL_639739	Centre of Nanotechnologies, INCD IMT-Bucuresti (National Institute for Research and Development in Microtechnologies - Bucharest)	Centre of Nanotechnologies, INCD IMT-Bucuresti (National Institute for Research and Development in Microtechnologies - Bucharest)	Salceanu,A., Gogianu,L. and Baisan,M.
EPI_ISL_649126, EPI_ISL_649127, EPI_ISL_649128, EPI_ISL_649129, EPI_ISL_649130, EPI_ISL_649131, EPI_ISL_649132, EPI_ISL_649133, EPI_ISL_649134, EPI_ISL_649135, EPI_ISL_649136, EPI_ISL_649137, EPI_ISL_649138, EPI_ISL_649139, EPI_ISL_649140, EPI_ISL_649141, EPI_ISL_649142, EPI_ISL_649143, EPI_ISL_649144			
see above	Tokyo Metropolitan Institute of Public Health, Department of Microbiology	Tokyo Metropolitan Institute of Public Health, Department of Microbiology	Asakura,H., Kumagai,R., Yoshida,I., Nagashima,M., Chiba,T. and Sadamasu,K.
